# Multiple roles of aryloxide leaving groups in enantioselective annulations employing α,β-unsaturated acyl ammonium catalysis†
†Electronic supplementary information (ESI) available: Experimental procedures, product characterisation data (mpt, NMR, IR, HRMS, [*α*]_D_, HPLC), traces (NMR, HPLC) and X-ray crystallographic data. CCDC 1827462 and 1827463. For ESI and crystallographic data in CIF or other electronic format see DOI: 10.1039/c8sc01324a


**DOI:** 10.1039/c8sc01324a

**Published:** 2018-05-04

**Authors:** Mark D. Greenhalgh, Shen Qu, Alexandra M. Z. Slawin, Andrew D. Smith

**Affiliations:** a EaStCHEM , School of Chemistry , University of St Andrews , North Haugh , St Andrews KY16 9ST , UK . Email: ads10@st-andrews.ac.uk

## Abstract

An isothiourea-catalysed Michael addition–annulation process using β-fluoroalkyl-substituted α,β-unsaturated aryl esters and a range of 2-acylbenzazoles is reported for the enantioselective synthesis of dihydropyranone and dihydropyridinone products bearing polyfluorinated stereocenters (29 examples, up to 98% yield, >99 : 1 er). The choice of aryl group of the aryl ester proved essential in determining reaction enantioselectivity and dihydropyranone : dihydropyridinone product selectivity. The aryloxide leaving group is shown to play a number of essential additional roles, operating (i) as a Brønsted base, circumventing the need for an auxiliary base; and (ii) as a Lewis base to catalyse the isomerisation of dihydropyranone products into thermodynamically-favoured dihydropyridinones. After optimisation, this isomerisation process was exploited for the selective synthesis of dihydropyridinone products using acylbenzothiazoles, and either dihydropyranone or dihydropyridinone products using acylbenzoxazoles. Finally, the phenol derivative, produced following protonation of the aryloxide, is proposed to act as a Brønsted acid, which promotes an isothiourea-catalysed kinetic resolution of benzoxazole-derived dihydropyranones.

## Abstract 2

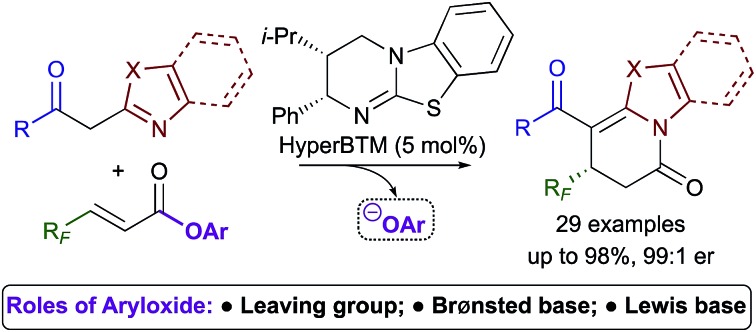
Aryloxides play an essential role as: (i) leaving group; (ii) Brønsted base; and (iii) Lewis base; in enantioselective isothiourea-catalysed annulations.

## 1. Introduction

**Scheme 1:**
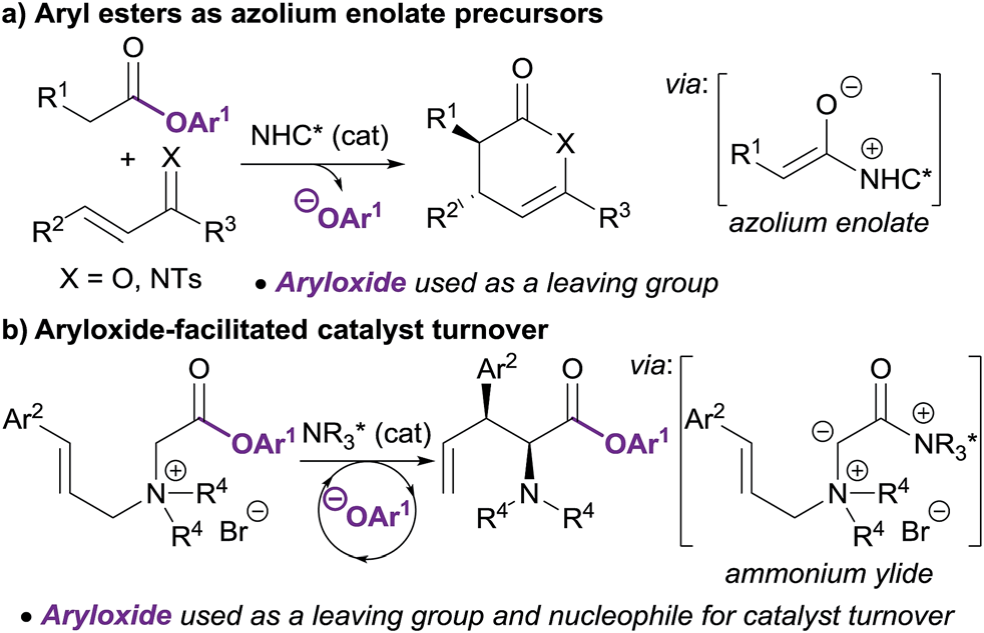
Previous uses of aryl ester substrates in NHC and tertiary amine catalysis.

Lewis base organocatalysis is now firmly established as a cornerstone of modern organic synthesis, with a variety of distinct reactivity modes allowing the construction of complex products with high levels of regio-, chemo- and stereocontrol.^1^ A critical feature for the widespread adoption of any synthetic procedure is the ease of access and handling of the required substrates. A current focus in the fields of tertiary amine and N-heterocyclic carbene (NHC) catalysis is the use of bench-stable ammonium/azolium enolate and α,β-unsaturated acyl ammonium/azolium precursors.^2^ This has resulted in the replacement of notoriously-unstable ketenes and acid chlorides in these processes with homoanhydrides, *in situ* formed mixed anhydrides, and aryl esters. The use of isolated, bench-stable aryl esters as starting materials is of particular interest due to the simplicity of reaction set-up and improved atom economy relative to methods using anhydrides. Chi first introduced aryl esters as azolium enolate precursors in 2012,^3^ demonstrating that aryl esters bearing electron withdrawing substituents were essential to achieve sufficient nucleofugality of the aryloxide leaving group (Scheme 1a). Chi subsequently applied electron-deficient aryl esters in a range of NHC-catalysed formal cycloaddition and domino cascade processes involving azolium (homo)enolate and α,β-unsaturated acyl azolium intermediates.^4^ In each case, the aryloxide was only considered as a simple leaving group.^5^

The use of electron-deficient aryl esters in enantioselective tertiary amine catalysis was first reported by our research group in 2014 for the 2,3-rearrangement of allylic ammonium ylides (Scheme 1b).^6^,^7^ This method represented a conceptually different approach, with the aryloxide released from the substrate also required to facilitate intermolecular catalyst turnover.^8^ This approach, in which the aryloxide performs a dual role, has since been successfully applied in ammonium enolate and α,β-unsaturated acyl ammonium catalysis.^9^

**Scheme 2:**
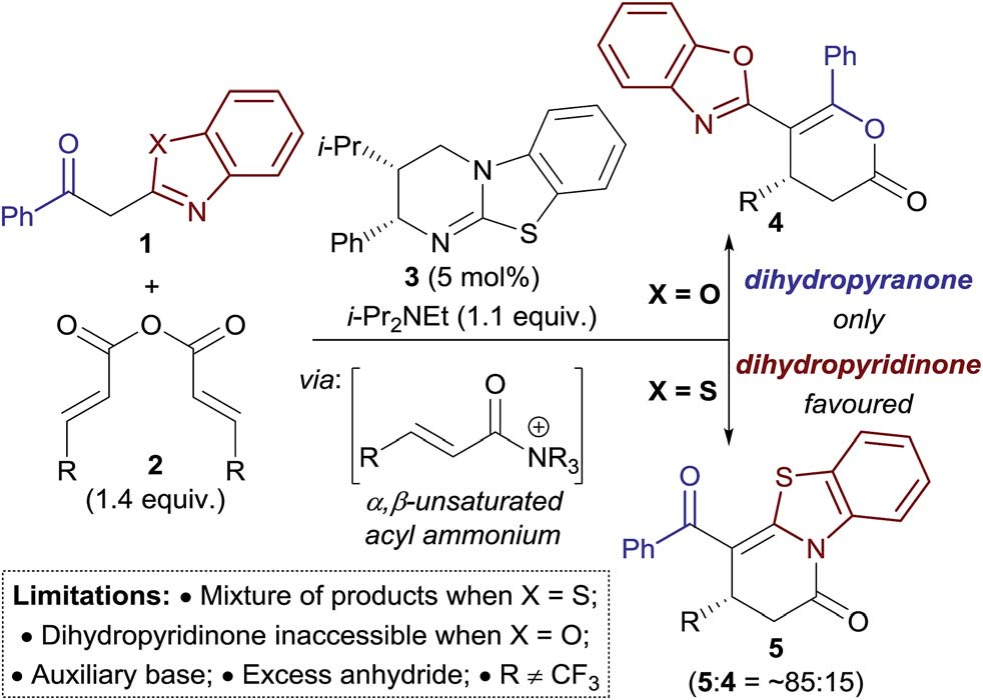
Isothiourea-catalysed Michael addition–annulation of homoanhydrides with 2-acylbenzazole derivatives.

The field of enantioselective α,β-unsaturated acyl ammonium catalysis has seen a recent rise in popularity.^10^,^11^ Following seminal work in 2006 by Fu on [3 + 2] annulations using a planar-chiral DMAP catalyst,^12^ little attention was given to this field until publications by Lupton, Romo and ourselves using isothiourea catalysis.^13^ Since 2013 a range of highly enantioselective Michael addition–annulation, formal cycloaddition and complex cascade methodologies have been developed.^14^ For example, we reported recently an isothiourea-catalysed Michael addition–annulation process using 2-acylbenzazole pro-nucleophiles **1** and homoanhydrides **2** as α,β-unsaturated acyl ammonium precursors (Scheme 2).14e,^15^ In this work, the selectivity of annulation depended upon the identity of the 2-acylbenzazole substrate. 2-Acylbenzoxazole substrates (X = O) exclusively gave dihydropyranones **4**, whilst 2-acylbenzothiazoles (X = S) preferentially gave the corresponding dihydropyridinone **5** (typically in ∼85 : 15 ratio of **5** : **4**). Experimental and computational studies showed the selectivity of annulation to be kinetically-derived, with non-covalent C–H···O and S···O interactions present in the respective annulation transition states implicated in determining product selectivity.

Due to general widespread interest in the formation of products containing fluorinated substituents at stereogenic centres,^16^ we sought to apply this method to prepare fluorinated heterocycles in enantiopure form. However, the prohibitive instability of fluorinated homoanhydrides (*e.g.* R = CF_3_) led us to investigate β-polyfluoroalkyl-substituted α,β-unsaturated aryl esters as alternative acyl ammonium precursors. Herein, we report the development of this process, during which the aryloxide leaving group (ArO^–^) has been identified as playing a number of additional key roles in determining catalytic efficiency and selectivity. In this manuscript we show that the *in situ* generated aryloxide (ArO^–^) acts as (i) a Brønsted base, circumventing the previous requirement for an auxiliary base; (ii) a Lewis base, which can be exploited to selectively catalyse the isomerisation of dihydropyranones into thermodynamically-favoured dihydropyridinones. Additionally, it was found that ArOH, produced upon protonation of the aryloxide, can act as a Brønsted acid that promotes an isothiourea-catalysed kinetic resolution of benzoxazole-derived dihydropyranones.

## 2. Results and discussion

### 2.1 Reaction optimisation

Initial studies focused on the Michael addition–annulation of 2-phenacylbenzothiazole **6** and β-CF_3_-substituted α,β-unsaturated *para*-nitrophenyl (PNP) ester **7** using HyperBTM **3** as catalyst. In the presence of i-Pr_2_NEt as auxiliary base and 5 mol% HyperBTM, dihydropyridinone **10** was obtained as the sole product in quantitative yield and 86 : 14 er (Table 1, entry 1). In the absence of i-Pr_2_NEt full conversion was still observed, however a mixture of dihydropyridinone **10** and dihydropyranone **11** was obtained as a 3 : 1 ratio (entry 2). This preservation of reactivity is consistent with the released aryloxide operating as the Brønsted base in this case. Significantly, the absence of i-Pr_2_NEt also led to vastly improved enantioselectivity (95 : 5 er). This difference in enantioselectivity can be attributed to a competitive base-mediated background reaction in the presence of i-Pr_2_NEt (entry 3). Alternative isothiourea catalysts, solvents and reaction temperatures did not improve the er,^17^ so attention turned to the use of different α,β-unsaturated aryl esters. 3,5-Bis(trifluoromethyl)phenyl (BCF_3_P) ester **8** provided a mixture of dihydropyridinone **10** and dihydropyranone **11** in a similar ratio and er to PNP ester **7** (entry 4), however 2,4,6-trichlorophenyl (TCP) ester **9** gave **10** and **11** in close to a 1 : 1 ratio, but with excellent enantioselectivity (97 : 3 er, entry 5). Lowering the catalyst loading to 1 mol% resulted in a slight drop in conversion; however both products were still obtained with excellent enantiocontrol (entry 6).

**Table 1:** Reaction optimisation and controls

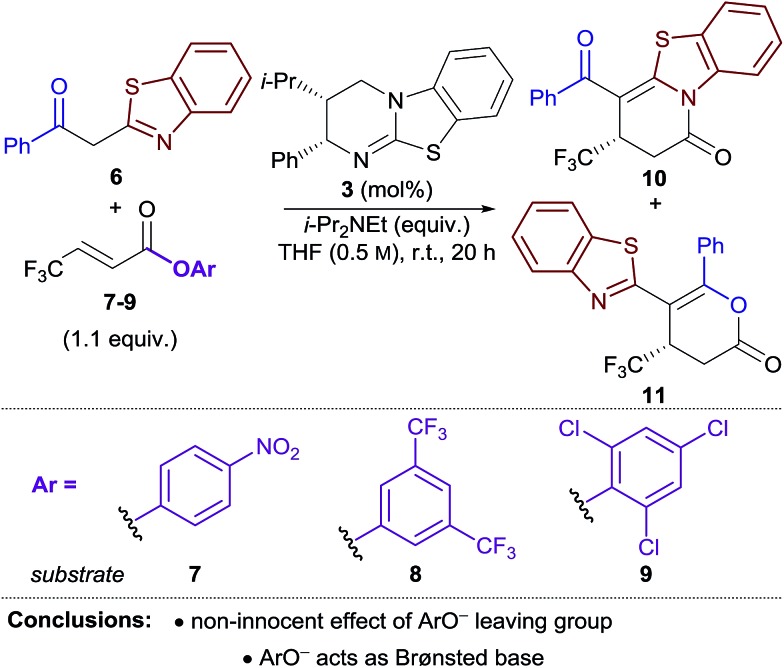							
Entry	**3** (mol%)	Substrate	i-Pr_2_NEt (equiv.)	**10**		**11**	
				%^*a*^	er^*b*^	%^*a*^	er^*b*^
1	5	**7**	1.2	96	86 : 14	0	NA^*c*^
2	5	**7**	0	75	95 : 5	23	ND^*d*^
3	0	**7**	1.2	75	NA^*c*^	13	NA^*c*^
4	5	**8**	0	73	94 : 6	21	94 : 6
5	5	**9**	0	54	97 : 3	42	97 : 3
6	1	**9**	0	47	97 : 3	38	97 : 3
7	0	**7**, **8** or **9**	0	0	NA^*c*^	0	NA^*c*^
8	0	**7**	0.1	63	NA^*c*^	15	NA^*c*^
9	0	**8**	0.1	45	NA^*c*^	21	NA^*c*^
10	0	**9**	0.1	17	NA^*c*^	7	NA^*c*^

^*a*^Determined by ^1^H NMR spectroscopy using 1,3,5-trimethoxybenzene as internal standard.

^*b*^Determined by chiral HPLC analysis.

^*c*^NA = not applicable.

^*d*^ND = not determined.

The differences in product ratio and enantioselectivity using different aryl esters provided the first indication that the aryloxide ‘leaving group’ was performing additional roles in the reaction. First, the differences in enantioselectivity were investigated. Control reactions between 2-phenacylbenzothiazole **6** and aryl esters **7–9** in the absence of HyperBTM resulted in no conversion in each case (entry 7). The addition of a substoichiometric amount of i-Pr_2_NEt (0.1 equiv.) successfully promoted the reaction, with high conversion obtained when using PNP and BCF_3_P esters **7** and **8** (66–78%) (entries 8, 9). In contrast, only modest conversion was observed when using TCP ester **9** (24%) (entry 10). These experiments indicate that the 0.1 equiv. of base served to initiate the reaction, with conversions of >10% consistent with the released aryloxide acting as a Brønsted base to propagate the reaction. The lower enantioselectivities obtained using PNP and BCF_3_P esters **7** and **8** in the Michael addition–annulation reaction may therefore be attributed to an enhanced base-mediated background reaction promoted by the released aryloxide.

**Fig. 1:**
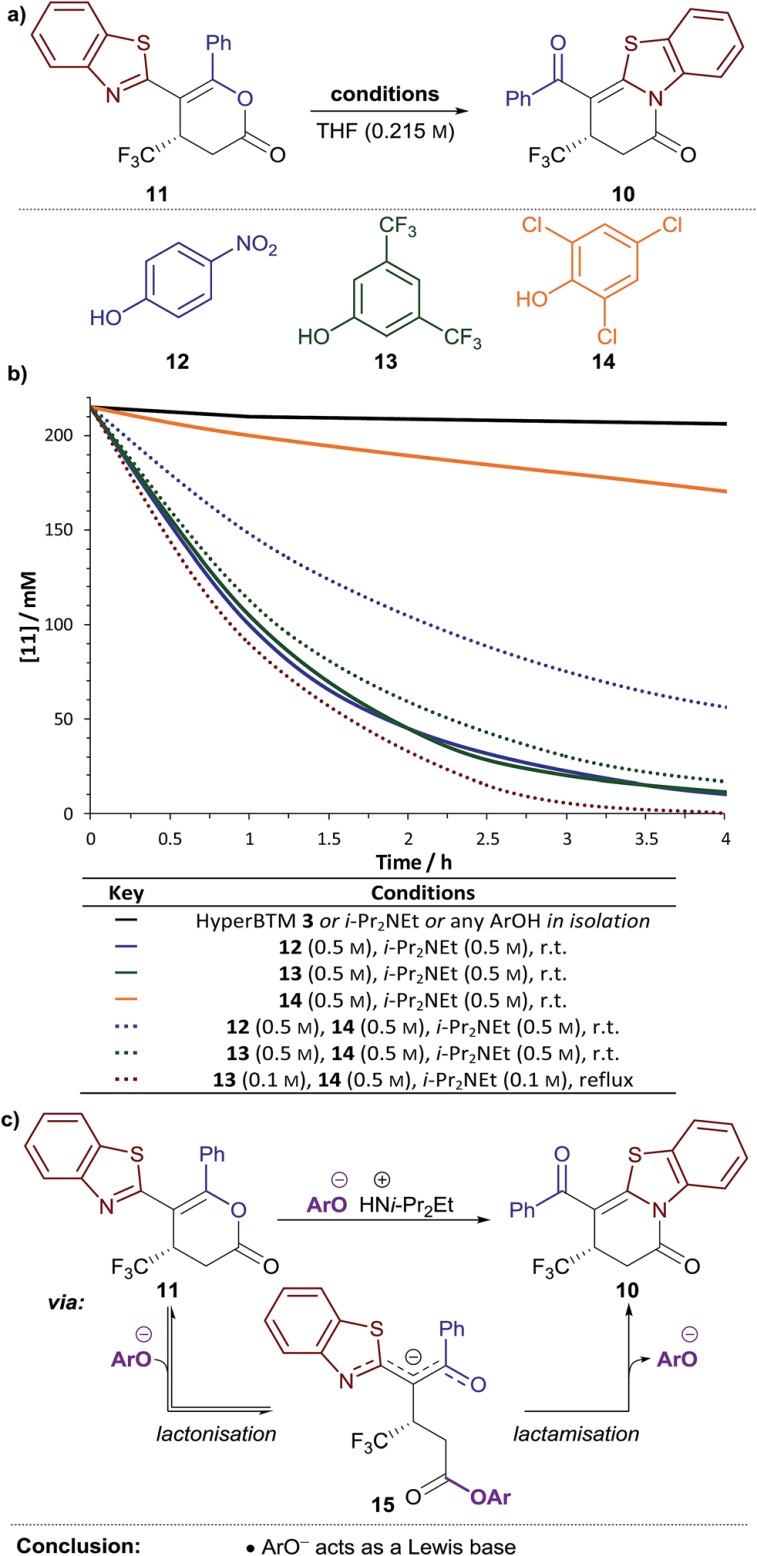
Isomerisation of dihydropyranone **11** to dihydropyridinone: (a) general reaction scheme; (b) temporal change in the concentration of **11** under different reaction conditions; (c) proposed mechanism for isomerisation.

Next, the variation in the ratio of dihydropyridinone and dihydropyranone products was probed. It was hypothesised this variation may arise from isomerisation of dihydropyranone **11** to give the thermodynamically-favoured dihydropyridinone **10** under the reaction conditions. The isomerisation of dihydropyranone **11** was therefore investigated in isolation under various conditions (Fig. 1a). In the presence of either HyperBTM **3**, i-Pr_2_NEt or a substituted phenol derivative alone, essentially no isomerisation of dihydropyranone **11** was observed (<5% in 5 h, Fig. 1b 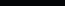). However, a combination of i-Pr_2_NEt (2.2 equiv.) and either *para*-nitrophenol **12** (PNPOH, 2.2 equiv., Fig. 1b 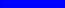) or 3,5-bis(trifluoromethyl)phenol **13** (BCF_3_POH, 2.2 equiv., Fig. 1b 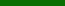) promoted effective isomerisation (*t*_1/2_ ≈ 1 h). This is consistent with the aryloxide, formed upon deprotonation of the phenol derivative, catalysing this isomerisation. In contrast, a combination of 2,4,6-trichlorophenol **14** (TCPOH, 2.2 equiv.) and i-Pr_2_NEt (2.2 equiv.) (Fig. 1b 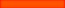) resulted in much slower isomerisation (*t*_1/2_ ≈ 12 h). These differences in the rate of dihydropyranone isomerisation in the presence of each aryloxide are consistent with the variation in product selectivity observed during reaction optimisation (Table 1, entries 2, 4, 5). A more extensive study of aryloxide derivatives found that those bearing *ortho*-substituents were uniformly ineffective for the isomerisation of dihydropyranone **11**.^17^ This trend in reactivity is synonymous with the aryloxide operating as a Lewis base in this process (Fig. 1c). Nucleophilic attack of the aryloxide on the dihydropyranone **11** would result in ring-opening to give aryl ester intermediate **15**, which may undergo lactonisation to reform the dihydropyranone **11** or lactamisation to give the thermodynamically-favoured dihydropyridinone **10**.

We envisioned that this isomerisation process could be applied following the Michael addition–annulation reaction to provide a single dihydropyridinone product. The highest enantioselectivity was obtained using TCP ester **9**, however isomerisation was most efficient using aryloxides derived from PNPOH **12** or BCF_3_POH **13**. Therefore the addition of either PNPO^–^ or BCF_3_PO^–^ at the end of the Michael addition–annulation process would be required. As a stoichiometric amount of TCPOH **14** would also be present at this stage in the process, the efficiency of dihydropyranone isomerisation using either PNPO^–^ or BCF_3_PO^–^ in the presence of an equivalent of TCPOH **14** was tested. The rate of dihydropyranone isomerisation using a combination of BCF_3_POH **13** and i-Pr_2_NEt (2.2 equiv. of each) (Fig. 1b 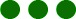) was essentially unaffected by the additional TCPOH **14** (2.2 equiv.); however a significant retardation in the rate of isomerisation was observed when using PNPOH **12** and i-Pr_2_NEt (2.2 equiv. of each) (Fig. 1b 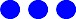). This confirmed the combination of BCF_3_POH **13** and i-Pr_2_NEt to be optimal for use in a telescoped Michael addition–annulation–isomerisation sequence. Further studies found that substoichiometric BCF_3_POH **13** and i-Pr_2_NEt could be used to affect efficient isomerisation by heating the reaction at reflux (Fig. 1b 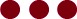).

**Scheme 3:**
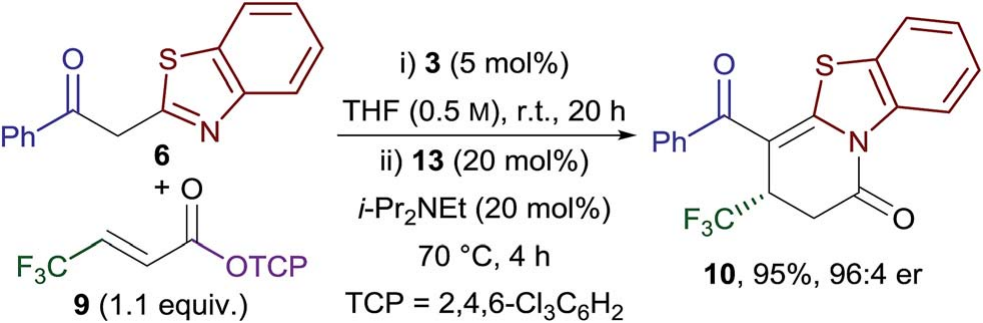
Optimised Michael addition–annulation–isomerisation reaction.

Combining the Michael addition–annulation and isomerisation processes, 2-phenylbenzothiazole, β-trifluoromethyl-substituted α,β-unsaturated TCP ester **9** and HyperBTM **3** (5 mol%) were reacted in THF at room temperature for 20 h, followed by the addition of BCF_3_POH **13** (20 mol%) and i-Pr_2_NEt (20 mol%) and heating at reflux for a further 4 h. This sequence provided dihydropyridinone **10** as the sole reaction product in 95% yield and 96 : 4 er (Scheme 3).

### 2.2 Reaction scope: benzothiazoles

The generality of this method was investigated for a range of 2-acylbenzothiazole derivatives and β-fluoroalkyl-substituted α,β-unsaturated TCP esters (Table 2). Substitution of the phenacyl group with both electron-donating and moderately electron-withdrawing groups provided dihydropyridinones **16–18** in excellent yield and with high enantioselectivity (Table 2a).^18^ Incorporation of a strong electron-withdrawing group (NO_2_) provided **19** in excellent yield, but with diminished enantioselectivity (87 : 13 er). *ortho*-Substitution of the aryl group was also tolerated, with 2-iodophenyl- and 1-naphthyl-functionalised products **20** and **21** obtained in excellent yield and enantiocontrol. The scope was extended to include heteroaromatic and alkyl-substituted ketones, with **22–24** all obtained in excellent yield and enantiocontrol. Next, variation of the benzothiazole unit was investigated (Table 2b). Substitution with fluoro, bromo, and methoxy groups was tolerated to give **25–27** in equally high yield and enantiocontrol. In addition, the use of 2-phenacylthiazole proved effective in giving dihydropyridinone **28** in high yield and enantiocontrol. The scope of the process was extended to different β-fluoroalkyl-substituted α,β-unsaturated TCP esters (Table 2c). Difluoromethyl substituents, which have experienced significant recent interest in drug design,16b,c,^19^ were successfully incorporated. A small scope including different (hetero)aryl- and alkyl-substituted ketones was demonstrated giving dihydropyridinones **29–32** in excellent yield and with good to high enantioselectivity. The incorporation of a pentafluoroethyl group at the stereogenic centre was also successful, with **33** obtained in excellent yield and enantiocontrol. The series of dihydropyridinones **10**, **29** and **33**, bearing different polyfluoroalkyl groups at the stereogenic centre, reveals a trend of improved enantioselectivity with increasing fluorine substitution.

**Table 2:** Michael addition–annulation–isomerisation using 2-acyl(benzo)thiazoles: ketone, (benzo)thiazole and fluoroalkyl variation

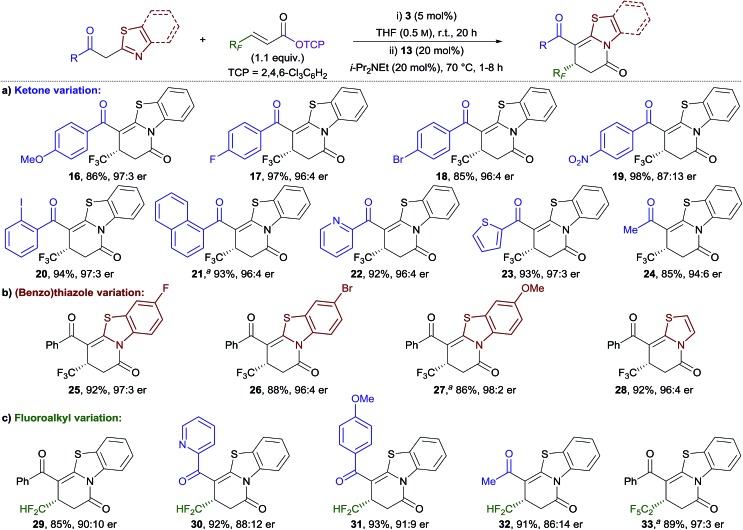

^*a*^10 mol% **3** used.

### 2.3 Reaction scope: benzoxazoles

In our previously-reported Michael addition–annulation methodology,14*e* the reaction of 2-phenacylbenzoxazole with homoanhydrides provided dihydropyranones as the sole reaction products. We postulated that the newly developed isomerisation process could allow selective access to either dihydropyranone or dihydropyridinone products, broadening the scope of this process. In the absence of an isomerisation step, the Michael addition–annulation between 2-phenacylbenzoxazole and β-trifluoromethyl-substituted α,β-unsaturated TCP ester **9** using HyperBTM **3** (10 mol%) at room temperature provided dihydropyranone **34** as the sole reaction product with exceptional enantioselectivity (>99 : 1 er) (Table 3, left). The scope of the acyl group was further investigated, with 3-pyridyl and 3-thienyl substituents providing dihydropyranones **35** and **36** in good yield and excellent enantiocontrol. Substitution of the acyl group with electron-withdrawing groups resulted in the formation of dihydropyranones **37** and **38** in improved yield, and with excellent enantioselectivity, albeit with reduced selectivity for the dihydropyranone product (∼90 : 10 dihydropyranone : dihydropyridinone). Interestingly, in these examples, the minor dihydropyridinone products **42** and **43** were obtained with lower enantioselectivity (∼92 : 8 er) in comparison to the major dihydropyranone products (>99 : 1 er). This effect is discussed in more detail in Section 2.5.

**Table 3:** Michael addition–annulation using 2-acylbenzoxazoles: selective formation of dihydropyranone or dihydropyridinone products

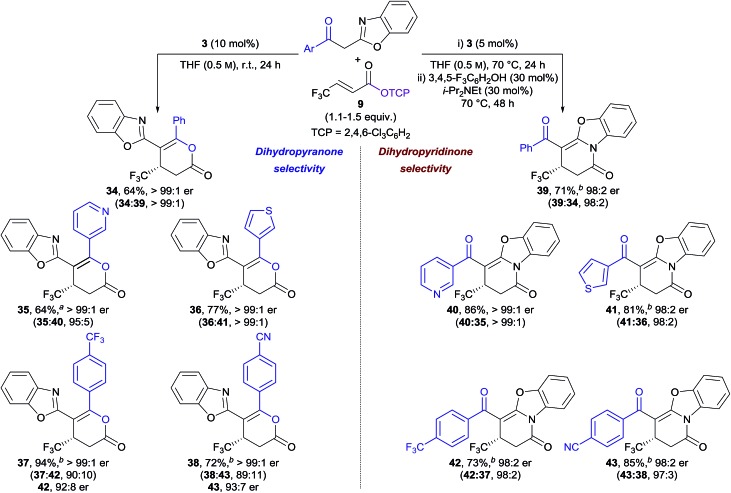

^*a*^Isolated as a single constitutional isomer.

^*b*^Isolated as a mixture of constitutional isomers.

The applicability of the telescoped Michael addition–annulation–isomerisation sequence was next investigated for the synthesis of dihydropyridinones. In this case, a combination of 3,4,5-trifluorophenol (30 mol%) and i-Pr_2_NEt (30 mol%), in addition to longer reaction times, proved optimal for complete conversion to the corresponding dihydropyridinones (Table 3, right). The isothiourea catalyst loading could be reduced to 5 mol% by heating the Michael addition–annulation step at reflux. Under these conditions the Michael addition–annulation proceeded in higher yield over shorter reaction times with excellent enantioselectivities (∼98 : 2 er). While reduced dihydropyranone : dihydropyridinone ratios were obtained, this product ratio was considered inconsequential due to the subsequent isomerisation step. The same five 2-acylbenzoxazole derivatives were applied in the Michael addition–annulation–isomerisation sequence giving dihydropyridinones **39–43** in good yield, excellent dihydropyridinone selectivity, and with high enantioselectivity (≥98 : 2 er) in each case.^20^

### 2.4 Extension of the isomerisation protocol

Finally, we were interested to see if the isomerisation protocol could be applied to our previously-reported Michael addition–annulation reaction using 2-acylbenzothiazoles and homoanhydrides.14*e* Although good to excellent yields and enantioselectivities had been reported, product isolation was complicated by the concurrent formation of dihydropyridinone and dihydropyranone products as a kinetically-determined and sometimes inseparable mixture (typically ∼85 : 15 ratio). As the original Michael addition–annulation process using homoanhydrides required a small excess of i-Pr_2_NEt (1.3 equiv.), isomerisation was attempted by the addition of 3,5-bis(trifluoromethyl)phenol **13** (40 mol%) after 6 h, followed by heating the reaction at reflux. This method proved successful, with the generality of the process demonstrated for aryl, alkyl, heteroaryl and alkenyl-substituted derivatives (Table 4). Dihydropyridinones **44–47** were obtained as the exclusive reaction products in excellent yield and with comparable enantioselectivity to the previously-reported method. This simple protocol improves the synthetic utility of the original method, and highlights the potential for the more widespread application of aryloxides as Lewis base catalysts.^21^

**Table 4:** Michael addition–annulation–isomerisation protocol using homoanhydrides

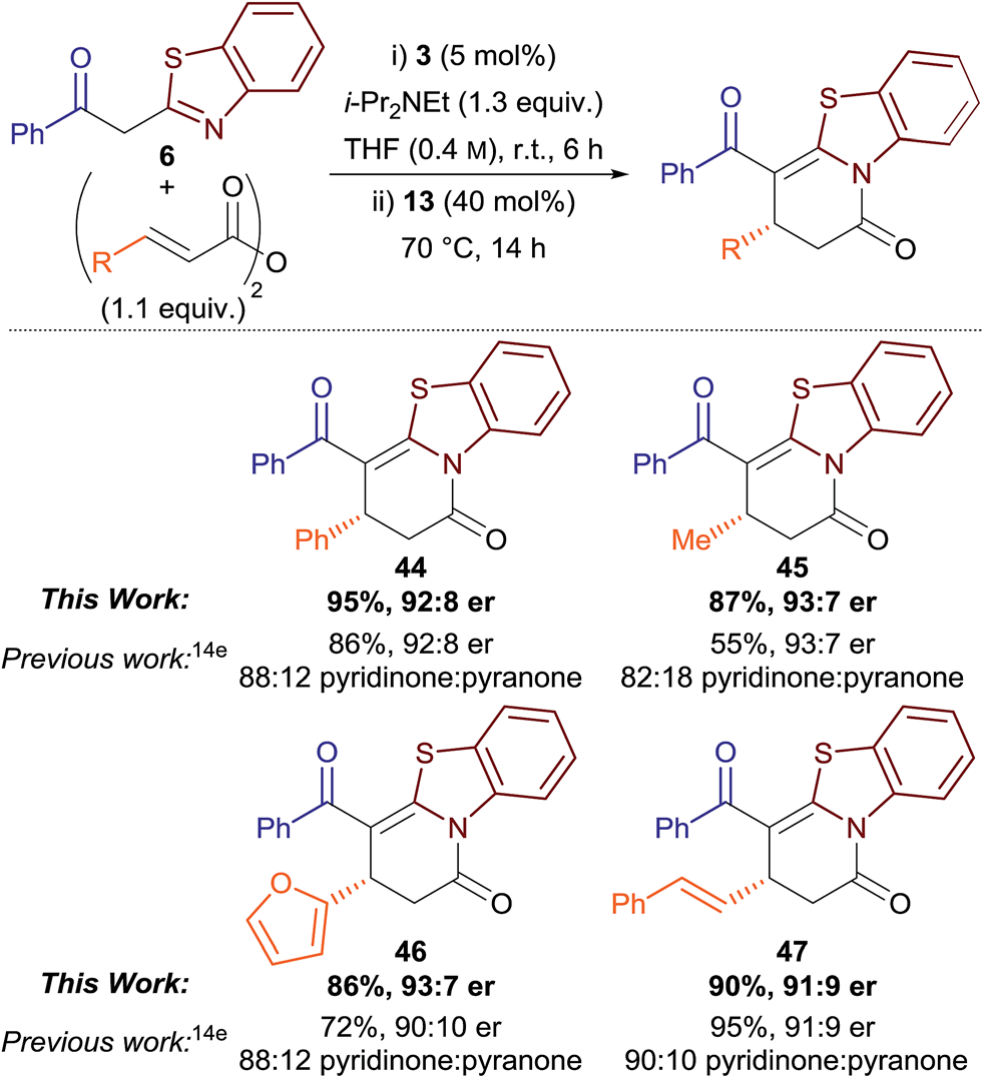

### 2.5 Kinetic resolution

Whilst exploring the selective synthesis of benzoxazole-derived dihydropyranones outlined in Section 2.3 (Table 3, left column), dihydropyranones **37** and **38** were obtained in essentially enantiopure form (>99 : 1 er), whilst the minor dihydropyridinone products, **42** and **43**, were obtained with significantly lower enantioenrichment (∼92 : 8 er). These differences in product er prompted further investigation. Reaction of racemic dihydropyranone (±)-**37** with HyperBTM **3** (10 mol%) gave a mixture of enantioenriched (*R*)-dihydropyridinone **42** and (*S*)-dihydropyranone **37** (Table 5, entry 1). This demonstrates that HyperBTM **3** is capable of affecting the isomerisation of benzoxazole-derived dihydropyranone **37**. This is in contrast to the isomerisation studies using benzothiazole-derived dihydropyranone **11**, in which HyperBTM was inactive (see Fig. 1b 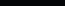). The observed formation of enantioenriched (*R*)-dihydropyridinone **42** and (*S*)-dihydropyranone **37** in this process suggests it can be simplistically described as a kinetic resolution.^22^ While the selectivity factor metric, *s*, is commonly used to report the efficiency of kinetic resolutions; in this case *s* was found to be dependent on reaction conversion, and was therefore not considered a valid descriptor.^17^ The enantioselectivity of this process, however, can be used to rationalise the differences in enantioenrichment observed between the dihydropyranone and dihydropyridinone products formed in the Michael addition–annulation process (Table 3). Significantly, (2*S*,3*R*)-HyperBTM **3** produces dihydropyranone (*S*)-**37** as the major product in the Michael addition–annulation reaction, but is more efficient at catalysing the isomerisation of (*R*)-**37** to give dihydropyridinone **42**. This larger rate constant for the isomerisation of (*R*)-**37** leads to further enrichment of the dihydropyranone product in (*S*)-**37**, whilst consequentially producing dihydropyridinone **42** with a lower level of enantioenrichment.

**Table 5:** Kinetic resolution of (±)-**37**

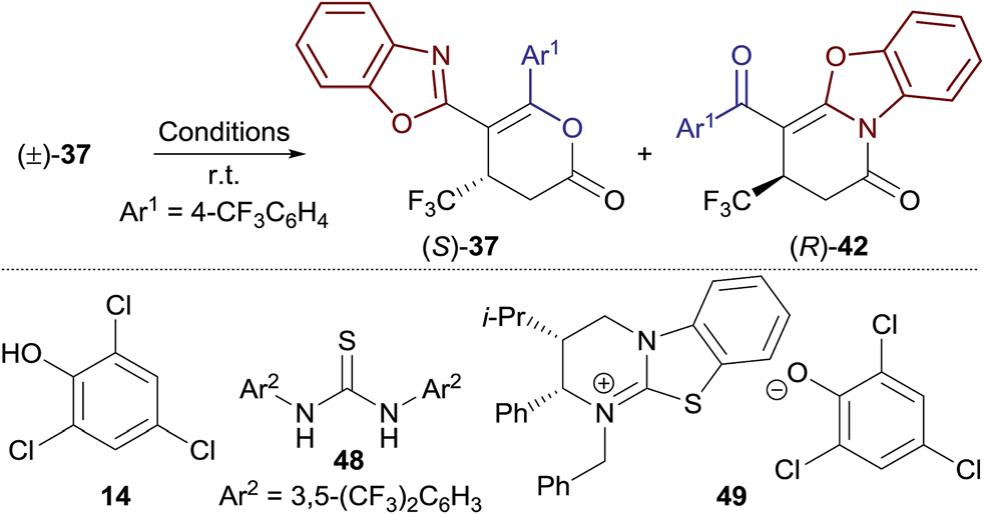				
Entry	Conditions^*a*^	Conv. (%)	**37** er	**42** er
1	**3** (10 mol%)	55	77 : 23	74 : 26
2	**3** (10 mol%) + **14** (1 equiv.)	56	92 : 8	85 : 15
3	**3** (10 mol%) + PhCO_2_H (10 mol%)	53	92 : 8	89 : 11
4	**3** (10 mol%) + **48** (10 mol%)	56	92 : 8	83 : 17
5	**49** (10 mol%)	64	50 : 50	50 : 50

^*a*^See ESI for reaction times.

To better simulate this kinetic resolution under the reaction conditions of the Michael addition–annulation process, the isomerisation of (±)-**37** was next investigated using a combination of HyperBTM **3** (10 mol%) and trichlorophenol **14** (1 equiv.) (entry 2). Improved enantioenrichment of both **37** (92 : 8 er) and **42** (85 : 15 er) was observed at a similar reaction conversion, indicating that the phenol additive has a beneficial effect on the kinetic resolution process. It was hypothesised that trichlorophenol could be either: (i) operating as a Brønsted acid/hydrogen bond donor to activate the dihydropyranone to nucleophilic attack by HyperBTM **3**; or (ii) deprotonated by HyperBTM **3** to produce an isothiouronium aryloxide ion pair, where the aryloxide acts as a nucleophile and enantioselectivity is induced by the chiral counterion HyperBTM-H^+^. To test the first hypothesis alternative non-nucleophilic Brønsted acids/hydrogen bond donors were applied. A combination of HyperBTM **3** and either benzoic acid or Schreiner's thiourea **48**^23^ resulted in similar or improved enantioselectivity relative to the use of trichlorophenol **14** (entries 3, 4). The beneficial effect of using benzoic acid in the planar-chiral DMAP catalysed dynamic kinetic resolution of azlactones has been previously noted, however the origin of this effect was not discussed.^24^ The second scenario was simulated using *N*-benzylisothiouronium trichlorophenoxide **49**, which catalysed the isomerisation, but gave both dihydropyridinone **42** and dihydropyranone **37** as racemates (entry 5).

**Scheme 4:**
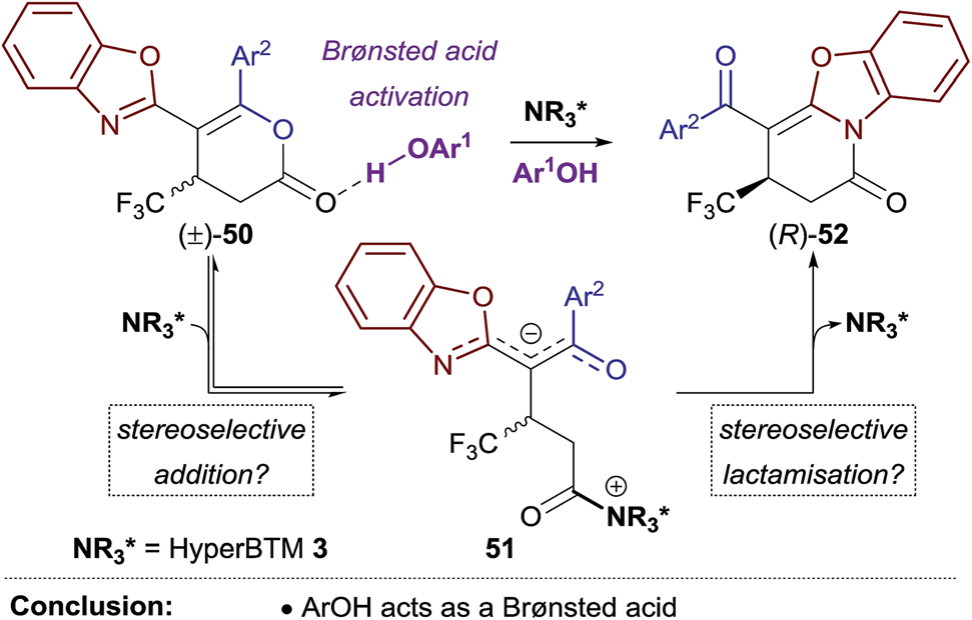
Proposed mechanism for the kinetic resolution of (±)-**50**.

**Scheme 5:**
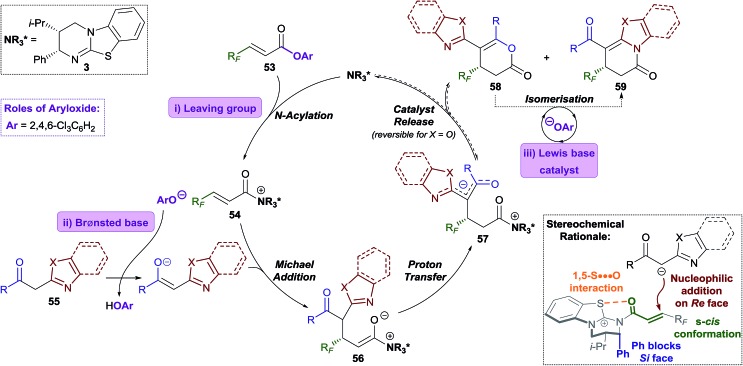
Proposed mechanism and stereochemical rationale.

These experiments are consistent with the phenol additive providing Brønsted acid activation of the dihydropyranone, and HyperBTM acting as a chiral nucleophile (Scheme 4). Nucleophilic attack of HyperBTM **3** onto racemic dihydropyranone **50** would produce two diastereomeric zwitterionic acyl isothiouronium intermediates **51**, which may undergo lactonisation to reform dihydropyranone **50**, or undergo lactamisation to give dihydropyridinone product **52**. The nucleophilic addition of HyperBTM to dihydropyranone **50** is expected to be reversible as zwitterionic acyl isothiouronium intermediate **51** is a proposed intermediate in the Michael addition–annulation process, in which dihydropyranone **50** is originally generated (see Scheme 5). The enantioselectivity observed within this process may therefore originate either from the preferential nucleophilic addition of HyperBTM **3** to (*R*)-**50**, and/or through the differential rates of lactamisation from each diastereomeric zwitterionic acyl isothiouronium intermediate **51**. We cannot currently differentiate these possibilities.^25^

### 2.6 Proposed mechanism

The Michael addition–annulation process is proposed to begin with *N*-acylation of HyperBTM **3** by α,β-unsaturated TCP ester **53** to give α,β-unsaturated acyl isothiouronium trichlorophenoxide ion pair **54** (Scheme 5).9*c* Deprotonation of the 2-acylbenzazole pro-nucleophile **55** by trichlorophenoxide provides trichlorophenol and a stabilised enolate, which undergoes Michael addition to α,β-unsaturated acyl isothiouronium **54** to give ammonium enolate **56**. Proton transfer, likely facilitated by trichlorophenol, gives zwitterionic intermediate **57**, which may undergo cyclisation through oxygen or nitrogen, regenerating the catalyst and giving dihydropyranone **58** or dihydropyridinone **59**, respectively. Trichlorophenoxide present in the reaction can facilitate isomerisation of dihydropyranone **58** to give the thermodynamically-favoured dihydropyridinone **59**. This isomerisation is most facile for benzothiazole-derived dihydropyranones (X = S), and presumably takes place through nucleophilic ring-opening of the dihydropyranone **58**, followed by lactamisation (see Fig. 1c). In the optimised protocol this isomerisation step was most efficiently catalysed by the addition of less sterically-hindered aryloxides, such as 3,5-bis(trifluoromethyl)phenoxide or 3,4,5-trifluorophenoxide. For benzoxazole-derived dihydropyranones, a second isomerisation pathway is possible, which is catalysed by HyperBTM **3** operating as a Lewis base, and trichlorophenol acting as a Brønsted acid.

The stereochemical outcome of the reaction can be rationalised by the α,β-unsaturated acyl isothiouronium **54** adopting an *s-cis* conformation, with a *syn*-coplanar non-covalent 1,5-S···O interaction between the acyl O and catalyst S providing a conformational lock.6c,13b,14a,e,i,^26^ Michael addition of the acylbenzazole-derived enolate to α,β-unsaturated acyl isothiouronium **54** then takes place *anti*- to the stereodirecting *pseudo*-axial phenyl substituent of the isothiourea catalyst (Scheme 5, bottom).

## 3. Conclusions

The isothiourea-catalysed enantioselective synthesis of a range of polyfluorinated dihydropyranone and dihydropyridinone products was achieved *via* a Michael addition–annulation process using α,β-unsaturated acyl ammonium catalysis (29 examples, up to 98%, >99 : 1 er). β-Fluoroalkyl-substituted α,β-unsaturated trichlorophenyl esters were used as the α,β-unsaturated acyl ammonium precursors, and a range of 2-acyl(benz)azoles used as the nucleophilic reaction partner. Significantly, the trichlorophenoxide leaving group was shown to play a variety of other roles in the reaction, including acting as (i) a Brønsted base, circumventing the need for the addition of an auxiliary base; and (ii) a Lewis base, catalysing the isomerisation of dihydropyranone products into thermodynamically-favoured dihydropyridinones. The isomerisation process was most efficient using less sterically-hindered aryloxide catalysts bearing electron-withdrawing groups, such as 3,5-bis(trifluoromethyl)phenoxide, 3,4,5-trifluorophenoxide or *para*-nitrophenoxide. These findings led to the development of a sequential Michael addition–annulation–isomerisation protocol for the synthesis of a range of benzothiazole-derived dihydropyridinone products as the only constitutional isomer in excellent yield and enantiocontrol. The method could also be applied when using 2-acylbenzoxazole pro-nucleophiles, with the selective formation of either dihydropyranones or dihydropyridinones achieved by including or omitting the isomerisation step. The aryloxide-promoted isomerisation protocol was further applied to our previously-reported Michael addition–annulation process using homoanhydrides, demonstrating the wide applicability of the method. Finally, the phenol derivative produced upon protonation of the aryloxide during the reaction was shown to act as a Brønsted acid, which promoted an isothiourea-catalysed kinetic resolution of benzoxazole-derived dihydropyranones. Overall, this work provides a concise and efficient method for the synthesis of polyfluorinated heterocyclic products in high yield and enantioselectivity. Identification of the multiple roles of the aryloxide leaving group in this process should inform future work in this area and provide inspiration for new reaction design.^27^

## Conflicts of interest

No conflicts of interest to declare.

## Supplementary Material

Supplementary information

Crystal structure data

Crystal structure data
