# Supplementary material for: Multiple roles of aryloxide leaving groups in enantioselective annulations employing α,β-unsaturated acyl ammonium catalysis
Source: Chem Sci. 2018 May 4;9(21):4909–18. doi: 10.1039/c8sc01324a (PMC5982221; doi:10.1039/c8sc01324a)
Supplement: Supplementary file 1 [file SC-009-C8SC01324A-s001.pdf]

Electronic Supplementary Material (ESI) for Chemical Science.
This journal is © The Royal Society of Chemistry 2018

Multiple roles of aryloxide leaving groups in enantioselective annulations employing α,β-unsaturated acyl ammonium catalysis

Mark D. Greenhalgh, Shen Qu, Alexandra M. Z. Slawin and Andrew D. Smith*

EaStCHEM, School of Chemistry, University of St Andrews, North Haugh, St Andrews, KY16 9ST, U.K.

Email: ads10@st-andrews.ac.uk

Supplementary Information

Table of Contents

General Experimental S2

General Procedures S4

Synthesis of β-Fluoroalkyl-Substituted α,β-Unsaturated Aryl Ester Derivatives S6

Synthesis of α,β-Unsaturated Anhydrides S9

Synthesis of Acyl (Benz)azole Derivatives S9

Reaction Optimisation and Isomerisation Studies S16

Isothiourea-Catalysed Enantioselective Synthesis of Fluorinated Dihydropyridinones and Dihydropyranones S19

Kinetic Resolution Studies S41

Single Crystal X-ray Analysis: Absolute Configuration of (*S*)-18 and (*S*)-41 S45

NMR traces of novel compounds S46

HPLC traces S181

References S218

General Experimental

Reactions involving moisture sensitive reagents were carried out in flame-dried glassware under an argon or nitrogen atmosphere using standard vacuum line techniques, and using anhydrous solvents. Anhydrous solvents (THF and toluene) were obtained from an anhydrous solvent system (purified using an alumina column, Mbraun SPS-800). All other reactions were performed in standard glassware with no precautions to exclude air or moisture. Solvents and commercial reagents were used as supplied without further purification unless otherwise stated.

Room temperature (rt) refers to 20-25 °C. Temperatures of 0 °C and −78 °C were obtained using ice/water and CO_2(s)/acetone baths respectively. Reflux conditions were obtained using a DrySyn, oil bath or sand bath equipped with a contact thermocouple.

In vacuo refers to the use either a Büchi Rotavapor R-200 with a Büchi V-491 heating bath and Büchi V-800 vacuum controller; a Büchi Rotavapor R-210 with a Büchi V-491 heating bath and Büchi V-850 vacuum controller; a Heidolph Laborota 4001 with vacuum controller; an IKA RV10 rotary evaporator with an IKA HB10 heating bath and ILMVAC vacuum controller; or an IKA RV10 rotary evaporator with an IKA HB10 heating bath and Vacuubrand CVC3000 vacuum controller. Rotary evaporator condensers are fitted to Julabo FL601 Recirculating Coolers filled with ethylene glycol and set to –5 °C.

Analytical thin layer chromatography was performed on pre-coated aluminium plates (Kieselgel 60 F_{254} silica). TLC visualisation was carried out with ultraviolet light (254 nm), followed by staining with a 1% aqueous $KMnO_4$ solution. Flash column chromatography was performed on Kieselgel 60 silica in the solvent system stated. Automated chromatography was performed on a Biotage Isolera Four running Biotage OS578 with a UV/Vis detector using the method stated and cartridges filled with Kieselgel 60 silica.

Melting points were recorded on an Electrothermal 9100 melting point apparatus and are uncorrected.

Optical rotations were measured on a Perkin Elmer Precisly/Model-341 polarimeter operating at the sodium D line with a 100 mm path cell at 20 °C.

HPLC analyses were obtained using either a Shimadzu HPLC consisting of a DGU-20A5 degassing unit, LC-20AT liquid chromatography pump, SIL-20AHT autosampler, CMB-20A communications bus module, SPD-M20A diode array detector and a CTO-20A column oven; or a Shimadzu HPLC consisting of a DGU-20A5R degassing unit, LC-20AD liquid chromatography pump, SIL-20AHT autosampler, SPD-20A UV/Vis detector and a CTO-20A column oven. Separation was achieved using DAICEL CHIRALCEL OD-H and OJ-H columns or DAICEL CHIRALPAK AD-H and IC columns using the method stated. HPLC traces of enantiomerically enriched compounds were compared with authentic racemic spectra.

^{1}H, ^{13}C{^{1}H} and ^{19}F{^{1}H} nuclear magnetic resonance (NMR) spectra were acquired on either a Bruker Avance II 400 (^{1}H 400 MHz; ^{19}F{^{1}H} 376 MHz) or a Bruker Avance II 500 (^{1}H 500 MHz; ^{13}C{^{1}H} 126 MHz) spectrometer at ambient temperature in the deuterated solvent stated. All chemical shifts are quoted in parts per million (ppm) and referenced to the residual solvent peak. All coupling constants, *J*, are quoted in Hz. Multiplicities are indicated by: s (singlet), d (doublet), t (triplet), q (quartet), sept

(septet), dd (doublet of doublets), dt (doublet of triplets), dq (doublet of quartets), td (triplet of doublets), tt (triplet of triplets), ddd (doublet of doublet of doublets), ddt (doublet of doublet of triplets) dddd (doublet of doublet of doublet of doublets), dddt (doublet of doublet of doublet of triplets), and m (multiplet). The abbreviation Ar is used to denote aromatic, Ph to denote phenyl, br to denote broad and app to denote apparent. NMR peak assignments were confirmed using 2D $^{1}H–^{13}C$ heteronuclear single quantum coherence (HSQC) and 2D $^{1}H–^{13}C$ heteronuclear multiple-bond correlation spectroscopy (HMBC) where necessary.

Infrared spectra were recorded on a Shimadzu IRAffinity-1 Fourier transform IR spectrophotometer fitted with a Specac Quest ATR accessory (diamond puck). Spectra were recorded of either thin films or solids, with characteristic absorption wavenumbers (ν_{max}) reported in cm^{-1}.

Mass spectrometry (*m/z*) data were acquired by electrospray ionization (ESI), atmospheric pressure chemical ionization (APCI) or nanospray ionization (NSI) either at the University of St Andrews or the EPSRC National Mass Spectrometry Facility, Swansea.

General procedures

General procedure A: *Synthesis of β-fluoroalkyl-substituted α,β-unsaturated aryl ester derivatives*

Oxalyl chloride (1.05 equiv.) and DMF (cat.) were added to a solution of a β-fluoroalkyl-substituted α,β-unsaturated acid (1.0 equiv.) in anhydrous CH_2Cl_2 (0.33 M) at r.t. under a N_2 atmosphere and allowed to stir for 1 h. A solution of a phenol derivative (1.0 equiv.) and *i*-Pr_2NEt (2.0 equiv.) in anhydrous CH_2Cl_2 (0.33 M) was added over 2-3 minutes and the solution allowed to stir overnight. The solvent was removed *in vacuo* and the product purified as specified.

General procedure B: *Synthesis of 2-(benzo)thiazole and -benzoxazole-substituted acetophenone derivatives from 2-methyl(benzo)thiazole or 2-methylbenzoxazole and an acid chloride*

Triethylamine (3.6 equiv.) was added to a solution of a 2-methylheterocycle derivative (1 equiv.) and an acid chloride (3 equiv.) in MeCN (0.25 M) at r.t. under a nitrogen atmosphere, and the reaction heated at reflux overnight. The reaction was cooled to r.t. and MeCN removed *in vacuo*. The residue was dissolved in CH_2Cl_2 and washed with saturated aqueous $NaHCO_3$ and brine, dried ($MgSO_4$), filtered and concentrated *in vacuo*. The residue was dissolved in the solvent system stated, KOH (2.5 equiv.) added and the reaction stirred overnight. The solvent was removed, the residue dissolved in EtOAc and washed sequentially with 1 M HCl (2 times), saturated aqueous $NaHCO_3$ and brine, dried ($MgSO_4$), filtered and concentrated *in vacuo* to give the crude product, which was purified as specified.

General procedure C: *Synthesis of 2-benzothiazole-substituted acetophenone derivatives from 2-chlorobenzothiazole and an acetophenone derivative*

NaHMDS (2 M solution in THF, 3 equiv.) was added dropwise to a solution of 2-chlorobenzothiazole (1 equiv.) and an acetophenone derivative (3 equiv.) in anhydrous toluene (0.33 M) at 0 °C under a nitrogen atmosphere. The reaction was allowed to stir for 3 h at 0 °C, and then allowed to warm to r.t. overnight. Saturated aqueous NH_4Cl was added, the layers separated, and the aqueous phase extracted with EtOAc (3 times). The combined organic fractions were washed (brine), dried ($MgSO_4$), filtered and concentrated *in vacuo*. The residue was dissolved in a minimal amount of boiling EtOH, and cooled in a freezer (−20 °C) overnight. The precipitate was filtered, washed sequentially with ice-cold EtOH and hexane, and dried under high vacuum to give the product.

General procedure D: *Synthesis of 2-benzoxazole-substituted acetophenone derivatives from 2-methylbenzoxazole and a carboxylic acid*

Oxalyl chloride (1.1 equiv.) and DMF (cat.) were added to a solution of a benzoic acid derivative (1.0 equiv.) in anhydrous CH_2Cl_2 (0.36 M) at r.t. under a N_2 atmosphere and allowed to stir for 2 h. The solvent was removed *in vacuo* and the acid chloride obtained (assuming quantitative conversion) was used according to **General Procedure B**.

General procedure E: *Isothiourea-catalysed enantioselective synthesis of fluorinated dihydropyridinones from acylbenzothiazoles and β-fluoroalkyl-substituted α,β-unsaturated aryl esters*

A β-fluoroalkyl-substituted α,β-unsaturated aryl ester (1.1 equiv.), acylbenzothiazole derivative (1 equiv.), HyperBTM (0.05 equiv.) and THF (0.5 M) were allowed to stir for 20 h at r.t. in a sealed vial. 3,5-Bis(trifluoromethyl)phenol (0.2 equiv.) and *i*-Pr_2NEt (0.2 equiv.) were added and the reaction heated at reflux for the time stated (between 1–8 h). The reaction was allowed to cool to r.t. and the product isolated by Biotage® Isolera 4 chromatography using the solvent system stated.

General procedure F: *Isothiourea-catalysed enantioselective synthesis of CF_3-substituted dihydropyranones from acylbenzoxazoles and a β-CF_3-substituted α,β-unsaturated aryl ester*

2,4,6-Trichlorophenyl (*E*)-4,4,4-trifluorobut-2-enoate (1.5 equiv.), acylbenzoxazole derivative (1 equiv.), HyperBTM (0.1 equiv.) and THF (0.5 M) were allowed to stir for 24 h at r.t. in a sealed vial. The product was isolated by Biotage® Isolera 4 chromatography using the solvent system stated.

General procedure G: *Isothiourea-catalysed enantioselective synthesis of CF_3-substituted dihydropyridinones from acylbenzoxazoles and a β-CF_3-substituted α,β-unsaturated aryl ester*

2,4,6-Trichlorophenyl (*E*)-4,4,4-trifluorobut-2-enoate (1.1 equiv.), acylbenzoxazole derivative (1 equiv.), HyperBTM (0.05 equiv.) and THF (0.5 M) were allowed to stir for 24 h at reflux in a sealed vial. 3,4,5-Trifluorophenol (0.3 equiv.) and *i*-Pr_2NEt (0.3 equiv.) were added and the reaction heated at reflux for a further 48 h. The reaction was allowed to cool to r.t. and the product isolated by Biotage® Isolera 4 chromatography using the solvent system stated.

General procedure H: *Isothiourea-catalysed enantioselective synthesis of dihydropyridinones from acylbenzothiazoles and α,β-unsaturated homoanhydrides*

An α,β-unsaturated homoanhydride (1.1 equiv.), 2-(benzo[*d*]thiazol-2-yl)-1-phenylethan-1-one (1 equiv.), *i*-Pr_2NEt (1.3 equiv.), HyperBTM (0.05 equiv.) and THF (0.4 M) were allowed to stir for 6 h at r.t. in a sealed vial. 3,5-Bis(trifluoromethyl)phenol (0.4 equiv.) was added and the reaction heated at reflux for 16 h. The reaction was allowed to cool to r.t. and the product isolated by Biotage® Isolera 4 chromatography using the solvent system stated.

Starting Material Synthesis

Synthesis of β-fluoroalkyl-substituted α,β-unsaturated aryl ester derivatives

(*E*)-4,4,4-Trifluorobut-2-enoic acid **S1**

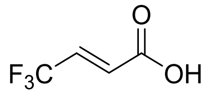

Aqueous NaOH (1 M, 60 mL, 60 mmol) was added to a solution of ethyl (*E*)-4,4,4-trifluorobut-2-enoate (8 mL, 53.5 mmol) in THF (80 mL), and was allowed to stir for 3 h at r.t. The solution was acidified to pH 2 with aqueous HCl (1 M). The volume was reduced *in vacuo* to remove excess THF, and the resulting solution was diluted with brine and extracted with diethyl ether (3 × 50 mL). The combined organic layers were dried ($MgSO_4$), filtered and concentrated *in vacuo* to give (*E*)-4,4,4-trifluorobut-2-enoic acid **S1** as colourless crystals (5.57 g, 39.8 mmol, 74%).

m.p. 53-55 °C {Lit.[1] 54-55 °C} **^{1}H NMR** (500 MHz, $CDCl_3$) δ_H: 6.53 (1H, dq, *J* 15.8, 1.9, C*H*CO_2H), 6.90 (1H, dq, *J* 15.8, 6.4, C*H*CF_3), 11.38 (1H, br s, O*H*); **^{19}F{^{1}H} NMR** (376 MHz, $CDCl_3$) δ_F: −65.8.

Data were in accordance with those previously reported.[2]

4-Nitrophenyl (*E*)-4,4,4-trifluorobut-2-enoate **7**

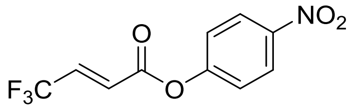

According to **General Procedure A**, oxalyl chloride (2.22 mL, 26.3 mmol), DMF (3 drops) and (*E*)-4,4,4-trifluorobut-2-enoic acid (3.50 g, 25 mmol) in CH_2Cl_2 (75 mL), followed by 4-nitrophenol (3.48 g, 25 mmol) and *i*-Pr_2NEt (8.70 mL, 50 mmol) in CH_2Cl_2 (75 mL) gave a brown solid which was recrystallized from hexane to give *4-nitrophenyl (E)-4,4,4-triluorobut-2-enoate* **7** as pale yellow crystals (5.50 g, 21.1 mmol, 84%).

m.p. 93-95 °C (hexane); **^{1}H NMR** (400 MHz, $CDCl_3$) δ_H: 6.73 (1H, dq, *J* 15.8, 2.0, C*H*CO_2H), 7.02 (1H, dq, *J* 15.8, 6.4, C*H*CF_3), 7.34-7.39 (2H, m, ArC(2,6)*H*), 8.29-8.35 (2H, m, ArC(3,5)*H*); **^{13}C{^{1}H} NMR** (126 MHz, $CDCl_3$) δ_C: 121.6 (q, $^1J_{CF}$ 270.9, *C*F_3), 122.2 (Ar*C*(2,6)H), 125.4 (Ar*C*(3,5)H), 127.4 (q, $^3J_{CF}$ 6.2, *C*(2)H), 134.2 (q, $^2J_{CF}$ 35.9, *C*(3)H), 145.8 (Ar*C*(4)), 154.5 (Ar*C*(1)) 161.4 (C=O); **^{19}F{^{1}H} NMR** (376 MHz, CDCl3) δ_F: −65.7; **IR** (neat) ν_{max} cm^{-1} 1742 (C=O), 1618, 1595, 1526, 1306, 1128, 972, 770; **HRMS** $(APCI)^+$ calculated for $C_{10}H_7NO_4F_3^+$ ($[M+H]^+$) requires 262.0322; found 262.0331 (+3.4 ppm).

3,5-Bis(trifluoromethyl)phenyl (*E*)-4,4,4-trifluorobut-2-enoate **8**

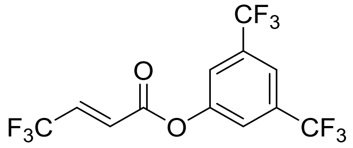

According to **General Procedure A**, oxalyl chloride (0.18 mL, 2.1 mmol), DMF (1 drop) and (*E*)-4,4,4-trifluorobut-2-enoic acid (280 mg, 2.0 mmol) in CH_2Cl_2 (6 mL), followed by 3,5-bis(trifluoromethyl)phenol (0.30 mL, 2.0 mmol) and *i*-Pr_2NEt (0.7 mL, 4.0 mmol) in CH_2Cl_2 (6 mL) gave

a brown solid which was triturated using Et_2O. The colourless solid (*i*-$Pr_2NEt·HCl$) was filtered and washed with Et_2O. The filtrate was concentrated *in vacuo* to give a brown oil with was purified by Biotage® Isolera 4 chromatography [eluent: 0%→10% Et_2O in petrol] to give *3,5-bis(trifluoromethyl)phenyl (E)-4,4,4-trifluorobut-2-enoate* **8** as a colourless volatile oil (356 mg, 1.0 mmol, 51%).

^{1}H NMR (500 MHz, $CDCl_3$) δ_H: 6.73 (1H, dq, *J* 15.8, 1.9, C*H*CO_2H), 7.03 (1H, dq, *J* 15.8, 6.4, C*H*CF_3), 7.67 (2H, s, ArC(2,6)*H*), 7.82 (1H, s, ArC(4)*H*); **^{13}C{^{1}H} NMR** (126 MHz, $CDCl_3$) δ_C: 120.6 (sept., $^3J_{CF}$ 3.8, Ar*C*(4)H), 122.4 (q, $^3J_{CF}$ 3.1, Ar*C*(2,6)H), 122.7 (q, $^1J_{CF}$ 272.9, ArC(3,5)*C*F_3), 121.7 (q, $^1J_{CF}$ 270.3, *C*(4)F_3), 127.2 (q, $^3J_{CF}$ 6.1, *C*(2)H), 133.4 (q, $^2J_{CF}$ 34.2, Ar*C*(3,5)), 134.6 (q, $^2J_{CF}$ 36.1, *C*(3)H), 150.6 (Ar*C*(1)), 161.6 (*C*=O); **^{19}F{^{1}H} NMR** (376 MHz, $CDCl_3$) δ_F: −63.0, −65.8; **IR** (neat) ν_{max} cm^{-1} 3100, 1763 (C=O), 1462, 1371, 1304, 1275, 1121 (C-F), 1105, 968. **HRMS** (APCI)$^+$ calculated for $C_{12}H_5O_2F_8^+$ ($[M-F]^+$) requires 333.0156; found 333.0157 (+0.3 ppm).

2,4,6-Trichlorophenyl (*E*)-4,4,4-trifluorobut-2-enoate **9**

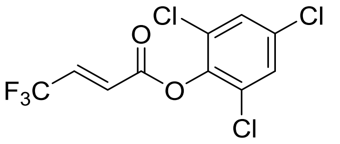

According to **General Procedure A**, oxalyl chloride (1.78 mL, 21 mmol), DMF (3 drops) and (*E*)-4,4,4-trifluorobut-2-enoic acid (2.80 g, 20 mmol) in CH_2Cl_2 (60 mL), followed by 2,4,6-trichlorophenol (3.95 g, 20 mmol) and *i*-Pr_2NEt (6.95 mL, 40 mmol) in CH_2Cl_2 (60 mL) gave a brown solid which was triturated using Et_2O. The colourless solid (*i*-$Pr_2NEt·HCl$) was filtered and washed with Et_2O. The filtrate was concentrated *in vacuo* to give a brown oil with was purified by Biotage® Isolera 4 chromatography [eluent: 0%→10% Et_2O in petrol; Rf = 0.79 (9:1 petrol:Et_2O)] to give *2,4,6-trichlorophenyl (E)-4,4,4-triluorobut-2-enoate* **9** as colourless crystals (5.78 g, 18.1 mmol, 90%).

m.p. 29-30 °C; **^{1}H NMR** (500 MHz, $CDCl_3$) δ_H: 6.76 (1H, dq, *J* 15.8, 1.9, C*H*CO_2H), 7.06 (1H, dq, *J* 15.8, 6.4, C*H*CF_3), 7.42 (2H, s, ArC(3,5)*H*); **^{13}C{^{1}H} NMR** (126 MHz, $CDCl_3$) δ_C: 121.7 (q, $^1J_{CF}$ 271.2, *C*F_3), 126.6 (q, $^3J_{CF}$ 6.2, *C*(2)H), 128.9 (Ar*C*(3,5)H), 129.5 (Ar*C*(2,6)), 132.9 (Ar*C*(4)), 134.6 (q, $^2J_{CF}$ 36.1, *C*(3)H), 142.3 (Ar*C*(1)), 160.2 (*C*=O); **^{19}F{^{1}H} NMR** (376 MHz, $CDCl_3$) δ_F: −65.8; **IR** (neat) ν_{max} cm^{-1} 1765 (C=O), 1566, 1447, 1307, 1233, 1217, 1119 (C-F), 957. **HRMS** (APCI)$^+$ calculated for $C_{10}H_5O_2F_3{}^{35}Cl_3^+$ ($[M+H]^+$) requires 318.9302; found 318.9303 (−0.3 ppm).

2,4,6-Trichlorophenyl (*E*)-4,4-difluorobut-2-enoate **S2**

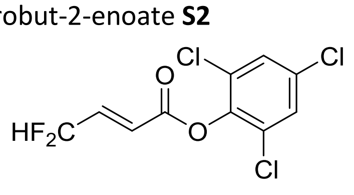

According to **General Procedure A**, oxalyl chloride (0.26 mL, 3.0 mmol), DMF (1 drop) and (*E*)-4,4-difluorobut-2-enoic acid [349 mg, 2.86 mmol (synthesised from ethyl (*E*)-4,4-difluorobut-2-enoate according to a literature procedure)[3] in CH_2Cl_2 (7 mL), followed by 2,4,6-trichlorophenol (565 mg, 2.86 mmol) and *i*-Pr_2NEt (1.0 mL, 5.72 mmol) in CH_2Cl_2 (7 mL) gave a brown solid which was triturated using Et_2O. The colourless solid (*i*-$Pr_2NEt·HCl$) was filtered and washed with Et_2O. The filtrate was

concentrated *in vacuo* to give a brown oil with was purified by Biotage® Isolera 4 chromatography [eluent: 0%→10% Et_2O in petrol] to give *2,4,6-trichlorophenyl (E)-4,4-difluorobut-2-enoate* **S2** as a colourless oil (705 mg, 2.34 mmol, 82%).

^{1}H NMR (500 MHz, $CDCl_3$) δ_H: 6.34 (1H, tdd, *J* 54.6, 3.8, 1.0, C*H*F_2), 6.56 (1H, dtd, *J* 15.9, 2.9, 1.0, C*H*CO_2H), 7.10 (1H, dtd, *J* 15.9, 10.4, 3.8, C*H*CHF_2), 7.41 (2H, s, ArC(3,5)*H*); **^{13}C{^{1}H} NMR** (126 MHz, $CDCl_3$) δ_C: 112.0 (t, $^1J_{CF}$ 238.3, *C*HF_2), 126.6 (t, $^3J_{CF}$ 10.4, *C*(2)H), 128.9 (Ar*C*(3,5)H), 129.6 (Ar*C*(2,6)), 132.7 (Ar*C*(4)), 140.0 (t, $^2J_{CF}$ 24.0, *C*(3)H), 142.5 (Ar*C*(1)), 160.9 (*C*=O); **^{19}F{^{1}H} NMR** (376 MHz, $CDCl_3$) δ_F: −117.1; **IR** (neat) ν_{max} cm^{-1} 3084, 2974, 1759 (C=O), 1566, 1447, 1385, 1223, 1121, 1040. **HRMS** $(APCI)^+$ calculated for $C_{10}H_6O_2F_2{}^{35}Cl_3{}^+$ ($[M+H]^+$) requires 300.9396; found 300.9400 (+1.3 ppm).

2,4,6-Trichlorophenyl (*E*)-4,4,5,5,5-pentafluoropent-2-enoate **S3**

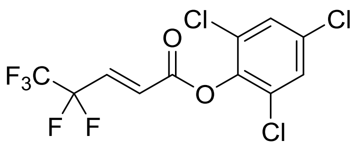

According to **General Procedure A**, oxalyl chloride (0.11 mL, 1.27 mmol), DMF (1 drop) and (*E*)-4,4,5,5,5-pentafluoropent-2-enoic acid [230 mg, 1.21 mmol (synthesised from ethyl (*E*)-4,4,5,5,5-pentafluoropent-2-enoate according to a literature procedure)[3] in CH_2Cl_2 (4 mL), followed by 2,4,6-trichlorophenol (239 mg, 1.21 mmol) and *i*-Pr_2NEt (0.42 mL, 2.42 mmol) in CH_2Cl_2 (4 mL) gave a brown solid which was triturated using Et_2O. The colourless solid (*i*-Pr_2NEt·HCl) was filtered and washed with Et_2O. The filtrate was concentrated *in vacuo* to give a brown oil with was purified by Biotage® Isolera 4 chromatography [eluent: 0%→10% Et_2O in petrol] to give *2,4,6-trichlorophenyl (E)-4,4,5,5,5-pentafluoropent-2-enoate* **S3** as a colourless oil (307 mg, 0.83 mmol, 69%).

^{1}H NMR (500 MHz, $CDCl_3$) δ_H: 6.83 (1H, dt, *J* 15.9, 2.0, C*H*CO_2H), 7.09 (1H, dt, *J* 15.9, 11.5, C*H*C_2F_5), 7.42 (2H, s, ArC(3,5)*H*); **^{13}C{^{1}H} NMR** (126 MHz, $CDCl_3$) δ_C: 111.4 (tq, *J* 253.5, 38.8, *C*F_2CF_3), 118.5 (qt, *J* 285.9, 36.2, CF_2*C*F_3), 128.5 (t, *J* 8.4, *C*(2)H), 128.9 (Ar*C*(3,5)H), 129.4 (Ar*C*(2,6)), 132.9 (Ar*C*(4)), 133.8 (t, *J* 24.1, *C*(3)H), 142.3 (Ar*C*(1)), 160.0 (*C*=O); **^{19}F{^{1}H} NMR** (376 MHz, $CDCl_3$) δ_F: −84.4 (t, *J* 2.1), −117.4 (q, *J* 2.1); **IR** (neat) ν_{max} cm^{-1} 3088, 2928, 1769 (C=O), 1566, 1449, 1281, 1200, 1121, 1042. **HRMS** $(APCI)^+$ calculated for $C_{11}H_5O_2F_5{}^{35}Cl_3{}^+$ ($[M+H]^+$) requires 368.9270; found 368.9270 (−0.0 ppm).

Synthesis of α,β-unsaturated anhydrides

(*E*)-Cinnamic anhydride, (*E*)-3-(furan-2-yl)acrylic anhydride and (*E*)-but-2-enoic anhydride were synthesized as previously reported.[4]

(*E*,*E*)-5-Phenylpenta-2,4-dienoic anhydride **S4**

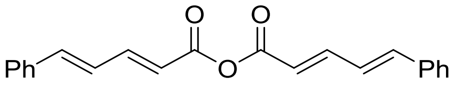

N-(3-Dimethylaminopropyl)-*N*′-ethylcarbodiimide hydrochloride (479 mg, 2.5 mmol) was added to a solution of (*E*,*E*)-5-phenylpenta-2,4-dienoic acid (610 mg, 3.5 mmol) in CH_2Cl_2 (5 mL) at r.t. and allowed to stir for 2h. CH_2Cl_2 (20 mL) and water (20 mL) was added and the layers separated. The organic layer was washed sequentially with water, saturated aqueous $NaHCO_3$ and brine, dried ($MgSO_4$), filtered and concentrated *in vacuo* to give *(E,E)-5-phenylpenta-2,4-dienoic anhydride* **S4** as a pale yellow solid (412 mg, 1.25 mmol, 71%).

m.p. 94-95 °C; **^{1}H NMR** (500 MHz, $CDCl_3$) δ_H: 6.07 (2H, d, *J* 15.2, C(2)*H*), 6.94 (2H, dd, *J* 15.6, 10.7, C(4)*H*), 7.01 (2H, d, *J* 15.6, C(5)*H*), 7.32-7.42 (6H, m, PhC(3,4,5)*H*), 7.47-7.52 (4H, m, PhC(2,6)*H*), 7.60 (2H, dd, *J* 15.2, 10.7, C(3)*H*); **^{13}C{^{1}H} NMR** (126 MHz, $CDCl_3$) δ_C: 119.7 (*C*(2)H), 125.9 (*C*(4)H), 127.6 (Ph*C*(3,5)H), 129.0 (Ph*C*(2,6)H), 129.8 (Ph*C*(4)H), 135.7 (Ph*C*(1)), 143.0 (*C*(5)H), 148.6 (*C*(3)H), 162.7 (C=O); **IR** (neat) ν_{max} cm^{-1} 3059, 3024, 1767, 1703, 1614, 1591, 1449, 1344, 1315, 1308, 1296, 1219, 1047, 1003; **HRMS** (APCI)$^+$ calculated for $C_{22}H_{19}O_3^+$ ($[M+H]^+$) requires 331.1329; found 331.1327 (−0.6 ppm).

Synthesis of acyl(benz)azole derivatives

2-(Benzo[*d*]thiazol-2-yl)-1-phenylethan-1-one **6**, 2-(benzo[*d*]thiazol-2-yl)-1-(4-fluorophenyl)ethan-1-one, 2-(benzo[*d*]thiazol-2-yl)-1-(4-bromophenyl)ethan-1-one, 2-(benzo[*d*]thiazol-2-yl)-1-(4-methoxyphenyl)ethan-1-one, 2-(6-fluorobenzo[*d*]thiazol-2-yl)-1-phenylethan-1-one, 2-(6-bromobenzo[*d*]thiazol-2-yl)-1-phenylethan-1-one, 2-(6-methoxybenzo[*d*]thiazol-2-yl)-1-phenylethan-1-one, 1-(benzo[*d*]thiazol-2-yl)propan-2-one and 2-(benzo[*d*]oxazol-2-yl)-1-phenylethan-1-one were synthesised as previously reported.[4-7]

2-(Benzo[*d*]thiazol-2-yl)-1-(4-nitrophenyl)ethan-1-one **S5**

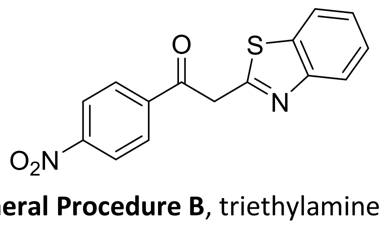

According to *a modification to* **General Procedure B**, triethylamine (1.5 mL, 10.8 mmol) was added to a solution of a 2-methylbenzothiazole (0.38 mL, 3 mmol) and 4-nitrobenzoyl chloride (1.67 g, 9 mmol) in MeCN (12 mL) and the reaction heated at reflux overnight under a nitrogen atmosphere. Upon cooling to r.t. a precipitate formed, which was collected by filtration and transferred to a round-bottomed flask. DMF (5 mL) and 1-butanol (5 mL) were added and the mixture heated at reflux overnight. Upon cooling to r.t. an orange precipitate formed, which was collected by filtration, suspended in hot EtOH and cooled in a freezer (−20 °C) overnight. The orange solid was collected by

filtration, washed with ice-cold EtOH and dried under high vacuum to give *2-(benzo[d]thiazol-2-yl)-1-(4-nitrophenyl)ethan-1-one* **S5** as a bright orange solid (582 mg, 1.95 mmol, 65%).

m.p. 205-207 °C (EtOH); **^{1}H NMR** (400 MHz, $CDCl_3$ – mixture of tautomers *enol*:*keto* = 22:1) *major (enol) tautomer* δ_H: 6.49 (enol-C*H*), 7.34-7.40 (1H, m, ArC*H*), 7.46-7.53 (1H, m, ArC*H*), 7.81-7.86 (1H, m, ArC*H*), 7.86-7.90 (1H, m, ArC*H*), 8.01-8.06 (2H, m, Ar_{NO2}C(2,6)*H*), 8.27-8.33 (2H, m, Ar_{NO2}C(3,5)*H*); *minor (keto) tautomer (selected)* δ_H: 4.85 (2H, s, CH_2); **^{13}C{^{1}H} NMR** (126 MHz, d_6-DMSO - *only enol tautomer observed*) δ_C: 88.1 (enol-*C*H), 112.4 (Ar*C*H), 123.1 (Ar*C*H), 123.3 (Ar*C*H), 124.2 (2 × Ar*C*H), 126.7 (Ar*C*), 127.5 (Ar*C*H), 128.4 (2 × Ar*C*H), 139.2 (Ar*C*), 145.3 (Ar*C*), 148.8 (Ar*C*), 164.1 (Ar*C*), 180.2 (enol-*C*OH); **IR** (neat) ν_{max} cm^{-1} 2893, 2826, 1564, 1504, 1456, 1427, 1389, 1339, 1294, 1202, 1109; **HRMS** (NSI)$^+$ calculated for $C_{15}H_{11}O_3N_2S^+$ ([M+H]$^+$) requires 299.0485; found 299.0488 (+1.0 ppm).

2-(Benzo[*d*]thiazol-2-yl)-1-(2-iodophenyl)ethan-1-one **S6**

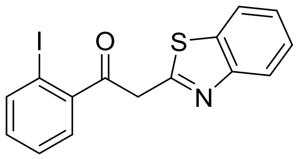

According to **General Procedure B**, triethylamine (1.0 mL, 7.2 mmol), 2-methylbenzothiazole (0.25 mL, 2.0 mmol), 2-iodobenzoyl chloride (1.6 g, 6.0 mmol) and MeCN (8 mL), followed by KOH (280 mg, 5.0 mmol), THF (5 mL) and MeOH (5 mL) gave the crude product which was purified by recrystallisation from hot EtOH: the crude product was dissolved in a minimal amount of boiling EtOH and then cooled in a freezer (−20 °C) overnight. The precipitate was collected by filtration, washed with ice-cold EtOH and dried under high vacuum to give *2-(benzo[d]thiazol-2-yl)-1-(2-iodophenyl)ethan-1-one* **S6** as brown needles (431 mg, 1.14 mmol, 57%).

m.p. 153-154 °C (EtOH); **^{1}H NMR** (400 MHz, $CDCl_3$ – mixture of tautomers *enol*:*keto* = 6:1) *major (enol) tautomer* δ_H: 6.00 (enol-C*H*), 7.09 (1H, app td, *J* 7.7, 1.7, ArC*H*), 7.28-7.34 (1H, m, ArC*H*), 7.37-7.53 (3H, m, ArC*H*), 7.73-7.80 (2H, m, ArC*H*), 7.94 (1H, dd, *J* 8.0, 1.1, ArC*H*); *minor (keto) tautomer* δ_H: 4.78 (2H, s, CH_2), 7.17 (1H, app td, *J* 7.9, 1.7, ArC*H*), 7.37-7.53 (3H, m, ArC*H*), 7.59 (1H, app dd, *J* 7.7, 1.6, ArC*H*), 7.89 (1H, app d, *J* 8.0, ArC*H*), 7.97 (1H, app dd, *J* 7.7, 0.9, ArC*H*), 8.02 (1H, app d, *J* 8.2, ArC*H*); **^{13}C{^{1}H} NMR** (126 MHz, d_6-DMSO - *only enol tautomer observed*) δ_C: 90.5 (enol-*C*H), 93.9 (Ar*C*), 112.0 (Ar*C*H), 123.0 (Ar*C*H), 126.5 (Ar*C*), 127.4 (Ar*C*H), 128.6 (Ar*C*H), 130.9 (Ar*C*H), 139.0 (Ar*C*), 140.0 (Ar*C*H), 147.3 (Ar*C*), 162.4 (Ar*C*), 187.0 (enol-*C*OH); **IR** (neat) ν_{max} cm^{-1} 1609, 1570, 1555, 1472, 1431, 1346, 1234, 1204, 1121, 1094, 1013; **HRMS** (APCI)$^+$ calculated for $C_{15}H_{11}ONSI^+$ ([M+H]$^+$) requires 379.9601; found 379.9601 (−0.0 ppm).

2-(Benzo[*d*]thiazol-2-yl)-1-(naphthalen-1-yl)ethan-1-one **S7**

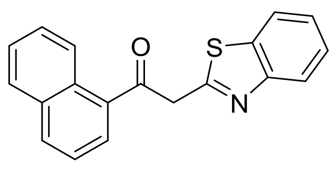

According to **General Procedure C**, NaHMDS (6 mL, 12 mmol), 1-(naphthalen-1-yl)ethan-1-one (1.82 mL, 12 mmol), 2-chlorobenzothiazole (0.52 mL, 4 mmol) and toluene (12 mL) gave *2-(benzo[d]thiazol-2-yl)-1-(naphthalen-1-yl)ethan-1-one* **S7** as a yellow solid (397 mg, 1.31 mmol, 33%).

m.p. 134-135 °C (EtOH); ^{1}**H NMR** (500 MHz, $CDCl_3$ – mixture of tautomers *enol*:*keto* = 2.7:1) *major (enol) tautomer* δ_H: 6.18 (1H, s, enol-C*H*), 7.31-7.36 (1H, m, Ar*H*), 7.45-7.59 (4H, m, Ar*H*), 7.75 (1H, dd, *J* 7.1, 1.3, Ar*H*), 7.79-7.83 (1H, m, Ar*H*), 7.83-7.95 (3H, m, Ar*H*), 8.46-8.53 (1H, m, Ar*H*); *minor (keto) tautomer* δ_H: 4.93 (2H, s, CH_2), 7.36-7.42 (1H, m, Ar*H*), 7.45-7.59 (3H, m, Ar*H*), 7.60-7.66 (1H, m, Ar*H*), 7.83-7.95 (2H, m, Ar*H*), 8.01-8.07 (2H, m, Ar*H*), 8.16 (1H, dd, *J* 7.3, 1.2, Ar*H*), 8.76-8.81 (1H, m, Ar*H*); ^{13}**C{^{1}H} NMR** (126 MHz, $CDCl_3$ – mixture of tautomers *enol*:*keto* = 2.7:1) *major (enol) tautomer* δ_C: 95.8 (enol-*C*H), 119.9 (Ar*C*H), 121.7 (Ar*C*H), 124.4 (Ar*C*H), 125.2 (Ar*C*H), 126.0 (Ar*C*H), 126.3 (Ar*C*H), 126.6 (Ar*C*H), 126.75 (Ar*C*H), 126.83 (Ar*C*H), 128.5 (Ar*C*H), 130.4 (Ar*C*H), 130.8 (Ar*C*), 131.2 (Ar*C*), 133.9 (Ar*C*), 134.4 (Ar*C*), 150.0 (Ar*C*), 168.0 (Ar*C*), 169.3 (enol-*C*OH); *minor (keto) tautomer (selected)* δ_C: 47.0 (*C*H_2), 121.7 (Ar*C*H), 123.1 (Ar*C*H), 124.5 (Ar*C*H), 125.3 (Ar*C*H), 126.0 (Ar*C*H), 126.2 (Ar*C*H), 126.9 (Ar*C*H), 128.7 (Ar*C*H), 129.4 (Ar*C*H), 134.2 (Ar*C*H); **IR** (neat) ν_{max} cm^{-1} 1558, 15006, 1450, 1416, 1383, 1256, 1223; **HRMS** (NSI)$^{+}$ calculated for $C_{19}H_{14}ONS^{+}$ ([M+H]$^{+}$) requires 304.0791; found 304.0794 (+1.1 ppm).

2-(Benzo[*d*]thiazol-2-yl)-1-(pyridin-2-yl)ethan-1-one **S8**

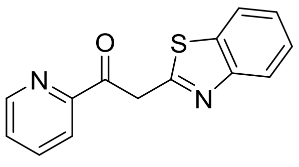

According to a *modification of* **General Procedure C**, NaHMDS (6 mL, 12 mmol), 1-(pyridin-2-yl)ethan-1-one (1.35 mL, 12 mmol), 2-chlorobenzothiazole (0.52 mL, 4 mmol) and toluene (12 mL) *were heated at reflux overnight* to give *2-(benzo[d]thiazol-2-yl)-1-(naphthalen-1-yl)ethan-1-one* **S8** as a yellow/brown solid (420 mg, 1.65 mmol, 41%).

m.p. 120-122 °C (EtOH); ^{1}**H NMR** (500 MHz, $CDCl_3$ – mixture of tautomers *enol*:*keto* = 4.5:1) *major (enol) tautomer* δ_H: 7.12 (1H, s, enol-C*H*), 7.30-7.36 (2H, m, Ar*H*), 7.43-7.49 (1H, m, Ar*H*), 7.78-7.90 (3H, m, Ar*H*), 7.99-8.06 (1H, m, Ar*H*), 8.65 (1H, ddd, *J* 4.7, 1.6, 0.8, Ar*H*); *minor (keto) tautomer* δ_H: 5.11 (2H, s, CH_2), 7.36-7.40 (1H, m, Ar*H*), 7.43-7.49 (1H, m, Ar*H*), 7.53 (1H, ddd, *J* 7.6, 4.8, 1.2, Ar*H*), 7.78-7.90 (2H, m, Ar*H*), 8.99-8.06 (1H, m, Ar*H*), 8.13 (1H, app dt, *J* 7.9, 1.0, Ar*H*), 8.74 (1H, ddd, *J* 4.7, 1.6, 0.9, Ar*H*); ^{13}**C{^{1}H} NMR** (126 MHz, $CDCl_3$ – mixture of tautomers *enol*:*keto* = 4.5:1) *major (enol) tautomer* δ_C: 92.6 (enol-*C*H), 120.3 (Ar*C*H), 121.0 (Ar*C*H), 121.6 (Ar*C*H), 124.5 (Ar*C*H), 124.6 (Ar*C*H), 126.6 (Ar*C*H), 132.0 (Ar*C*), 137.1 (Ar*C*H), 149.3 (Ar*C*H), 150.5 (Ar*C*), 152.2 (Ar*C*), 162.9 (Ar*C*), 168.1

(enol-*C*OH); *minor (keto) tautomer (selected)* δ_C: 42.8 (*C*H_2), 121.6 (Ar*C*H), 122.5 (Ar*C*H), 123.0 (Ar*C*H), 125.0 (Ar*C*H), 126.0 (Ar*C*H), 127.8 (Ar*C*H), 136.0 (Ar*C*), 137.2 (Ar*C*H), 152.3 (Ar*C*), 153.0 (Ar*C*), 164.0 (Ar*C*), 195.9 (C=O); **IR** (neat) ν_{max} cm^{-1} 1626, 1576, 1558, 1456, 1437, 1362, 1275, 1238, 1121, 1070, 1063; **HRMS** (ESI)$^+$ calculated for $C_{14}H_{10}ON_2SNa^+$ ([M+Na]$^+$) requires 277.0406; found 277.0396 (−3.6 ppm).

2-(Benzo[*d*]thiazol-2-yl)-1-(thiophen-2-yl)ethan-1-one **S9**

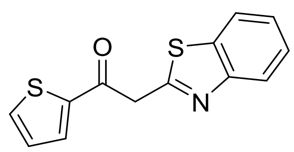

According to **General Procedure C**, NaHMDS (6 mL, 12 mmol), 1-(thiophen-2-yl)ethan-1-one (1.30 mL, 12 mmol), 2-chlorobenzothiazole (0.52 mL, 4 mmol) and toluene (12 mL) gave *2-(benzo[d]thiazol-2-yl)-1-(thiophen-2-yl)ethan-1-one* **S9** as a green/brown solid (943 mg, 3.64 mmol, 91%).

m.p. 117-118 °C (EtOH); **^{1}H NMR** (500 MHz, $CDCl_3$ – mixture of tautomers *enol*:*keto* = 1:2.4) *major (keto) tautomer* δ_H: 4.73 (2H, s, C*H*$_2$), 7.15 (1H, dd, *J* 5.0, 3.9, thiopheneC(4)*H*), 7.34-7.48 (2H, m, Ar*H*), 7.70 (1H, dd, *J* 5.0, 1.0, thiopheneC(3)*H*), 7.84-7.88 (1H, m, Ar*H*), 7.91 (1H, dd, *J* 3.9, 1.0, thiopheneC(5)*H*), 7.97-8.01 (1H, m, Ar*H*); *minor (enol) tautomer* δ_H: 6.24 (1H, s, enol-C*H*), 7.09 (1H, dd, *J* 5.0, 3.7, thiopheneC(4)*H*), 7.24 (1H, app td, *J* 7.6, 1.1, ArC*H*), 7.34-7.48 (2H, m, Ar*H*), 7.57 (1H, dd, *J* 3.7, 1.1, thiopheneC(5)*H*), 7.61-7.65 (1H, m, Ar*H*), 7.67-7.70 (1H, m, Ar*H*); **^{13}C{^{1}H} NMR** (126 MHz, $CDCl_3$ – mixture of tautomers *enol*:*keto* = 1:2.4) *major (keto) tautomer* δ_C: 44.6 (*C*H_2), 121.6 (Ar*C*H), 122.9 (Ar*C*H), 125.2 (Ar*C*H), 126.1 (Ar*C*H), 128.5 (Ar*C*H), 133.7 (Ar*C*H), 135.3 (Ar*C*H), 136.0 (Ar*C*), 142.9 (Ar*C*), 152.7 (Ar*C*), 163.1 (Ar*C*), 186.8 (C=O); *minor (enol) tautomer (selected)* δ_C: 89.2 (enol-*C*H), 117.8 (Ar*C*H), 121.6 (Ar*C*H), 123.9 (Ar*C*H), 126.7 (Ar*C*H), 127.0 (Ar*C*H), 128.0 (Ar*C*H), 129.0 (Ar*C*H), 129.4 (Ar*C*), 141.2 (Ar*C*), 147.3 (Ar*C*), 165.7 (*C*), 166.7 (*C*); **IR** (neat) ν_{max} cm^{-1} 3144, 3075, 1605, 1584, 1489, 1474, 1458, 1437, 1337, 1308, 1263, 1233, 1192; **HRMS** (NSI)$^+$ calculated for $C_{13}H_{10}ONS_2^+$ ([M+H]$^+$) requires 260.0198; found 260.0202 (+1.4 ppm).

1-Phenyl-2-(thiazol-2-yl)ethan-1-one **S10**

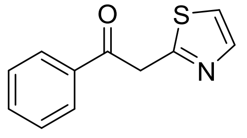

According to **General Procedure B**, triethylamine (2.0 mL, 14.4 mmol), 2-methylthiazole (0.36 mL, 4.0 mmol), benzoyl chloride (1.39 mL, 12.0 mmol) and MeCN (16 mL), followed by KOH (494 mg, 8.8 mmol), and MeOH (16 mL) gave the crude product which was purified by Biotage® Isolera 4 chromatography [eluent: 0%→15% EtOAc in petrol] to give 1-phenyl-2-(thiazol-2-yl)ethan-1-one **S10** as a green oil (772 mg, 3.80 mmol, 95%).

^{1}H NMR (400 MHz, $CDCl_3$ – mixture of tautomers *enol*:*keto* = 1:1.9) *major (keto) tautomer* δ_H: 4.75 (2H, s, C*H*$_2$), 7.32 (1H, d, *J* 3.3, thiazoleC(5)*H*), 7.45-7.53 (2H, m, PhC(3,5)*H*), 7.56-7.62 (1H, m, PhC(4)*H*), 7.77 (1H, d, *J* 3.3, thiazoleC(4)*H*), 8.03-8.11 (2H, m, PhC(2,6)*H*); *minor(enol) tautomer* δ_H:

6.35 (enol-C*H*), 7.07 (1H, d, *J* 3.4, thiazoleC(5)*H*), 7.36-7.45 (3H, m, PhC(3,4,5)*H*), 7.68 (1H, d, *J* 3.4, thiazoleC(4)*H*), 7.79-7.84 (2H, m, PhC(2,6)*H*); **HRMS** (NSI)$^+$ calculated for $C_{11}H_{10}ONS^+$ ($[M+H]^+$) requires 204.0478; found 204.0477 (−0.3 ppm).

Data were in accordance with those previously reported.[8]

2-(Benzo[*d*]oxazol-2-yl)-1-(pyridin-3-yl)ethan-1-one **S11**

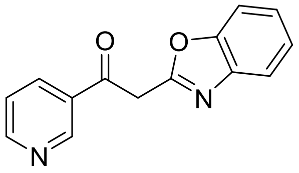

n-BuLi (2.5 M in hexane, 0.88 mL, 2.2 mmol) was added dropwise over 10 mins to a solution of 2-methylbenzoxazole (0.24 mL, 2 mmol) in anhydrous THF (10 mL) at −78 °C under a nitrogen atmosphere, and the reaction allowed to stir for 1 h. A solution of methyl nicotinate (165 mg, 1.2 mmol) in anhydrous THF (2 mL) was added over 10 mins and the reaction allowed to stir for a further 1 h at −78 °C, then warmed to r.t. Saturated aqueous $NaHCO_3$ and Et_2O were added and the layers separated. The aqueous was extracted with Et_2O (2 × 30 mL) and the combined organic fractions washed (brine), dried ($MgSO_4$), filtered and concentrated *in vacuo*. The residue was dissolved in a minimal amount of boiling EtOH and then cooled in a freezer (−20 °C) overnight. The precipitate was collected by filtration, washed sequentially with ice-cold EtOH and hexane and dried under high vacuum to give *2-(benzo[d]oxazol-2-yl)-1-(pyridin-3-yl)ethan-1-one* **S11** as an orange solid (171 mg, 0.72 mmol, 60%).

m.p. 125-127 °C (EtOH); **^{1}H NMR** (500 MHz, $CDCl_3$ – mixture of tautomers *enol*:*keto* = 4:1) *major (enol) tautomer* δ_H: 6.25 (2H, s, enol-C*H*), 7.28-7.38 (2H, m, ArC*H*), 7.40 (1H, ddd, *J* 8.0, 4.8, 0.8, PyC(5)*H*), 7.49-7.55 (1H, m, ArC*H*), 7.61-7.67 (1H, m, ArC*H*), 8.15 (1H, ddd, *J* 8.0, 2.2, 1.7, PyC(4)C*H*), 8.68 (1H, dd, *J* 4.8, 1.7, PyC(6)*H*), 9.11 (1H, dd, *J* 2.2, 0.8, PyC(2)*H*); *minor (keto) tautomer* δ_H: 4.65 (2H, s, CH_2), 7.28-7.38 (2H, m, ArC*H*), 7.47 (1H, ddd, *J* 8.0, 4.8, 0.8, PyC(5)*H*), 7.49-7.55 (1H, m, ArC*H*), 7.69-7.75 (1H, m, ArC*H*), 8.33 (1H, ddd, *J* 8.0, 2.1, 1.7, PyC(4)C*H*), 8.83 (1H, dd, *J* 4.8, 1.7, PyC(6)*H*), 9.11 (1H, dd, *J* 2.1, 0.8, PyC(2)*H*); **^{13}C{^{1}H} NMR** (126 MHz, $CDCl_3$ – mixture of tautomers *enol*:*keto* = 4:1) *major (enol) tautomer* δ_C: 85.0 (enol-*C*H), 110.6 (Ar*C*H), 118.2 (Ar*C*H), 123.6 (Ar*C*H), 124.7 (Ar*C*H), 125.1 (Ar*C*H), 133.4 (Py*C*(4)H), 147.5 (Py*C*(2)H), 151.4 (Py*C*(6)H), 130.2 (Ar*C*), 139.7 (Ar*C*), 148.9 (Ar*C*), 164.0 (*C*), 165.3 (*C*); *minor (keto) tautomer (selected)* δ_C: 40.0 (*C*H_2), 110.9 (Ar*C*H), 120.3 (Ar*C*H), 124.1 (Ar*C*H), 125.5 (Ar*C*H), 136.2 (Py*C*(4)H), 150.2 (Py*C*(2)H), 154.5 (Py*C*(6)H), 131.3 (Ar*C*), 141.4 (Ar*C*), 159.7 (Ar*C*), 191.6 (*C*=O); **IR** (neat) ν_{max} cm^{-1} 1624, 1568, 1530, 1454, 1283, 1246, 1165, 1072; **HRMS** (NSI)$^+$ calculated for $C_{14}H_{11}O_2N_2^+$ ($[M+H]^+$) requires 239.0815; found 239.0817 (+0.8 ppm).

2-(Benzo[*d*]oxazol-2-yl)-1-(thiophen-3-yl)ethan-1-one **S12**

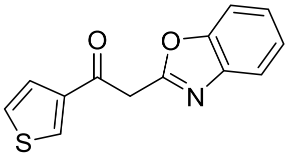

According to **General Procedure D**, oxalyl chloride (0.84 mL, 9.9 mmol), DMF (5 drops), thiophene-3-carboxylic acid (1.15 g, 9 mmol) and CH_2Cl_2 (25 mL); followed by triethylamine (1.5 mL, 10.8 mmol), 2-

methylbenzoxazole (0.36 mL, 3.0 mmol), and MeCN (12 mL); followed by KOH (420 mg, 7.5 mmol), and MeOH (9 mL) gave the crude product which was purified by Biotage® Isolera 4 chromatography [eluent: 0%→15% EtOAc in petrol] to give *2-(benzo[d]oxazol-2-yl)-1-(thiophen-3-yl)ethan-1-one* **S12** as a green solid (360 mg, 1.48 mmol, 49%).

m.p. 130-133 °C (EtOH); **^{1}H NMR** (500 MHz, $CDCl_3$ – mixture of tautomers *enol*:*keto* = 1:1.5) *major (keto) tautomer* δ_H: 4.53 (2H, s, C*H*$_2$), 7.24-7.38 (3H, m, ArC*H*), 7.49-7.54 (1H, m, ArC*H*), 7.57-7.63 (1H, m, ArC*H*), 7.69-7.74 (1H, m, ArC*H*), 8.24 (1H, app dd, *J* 2.7, 1.0, ArC*H*); *minor (enol) tautomer* δ_H: 6.05 (1H, s, enol-C*H*), 7.24-7.38 (3H, m, ArC*H*), 7.42 (1H, app dd, *J* 5.1, 0.9, ArC*H*), 7.48 (1H, app d, *J* 8.1, ArC*H*), 7.57-7.63 (1H, m, ArC*H*), 7.90 (1H, app dd, *J* 3.0, 1.0, ArC*H*); **^{13}C{^{1}H} NMR** (126 MHz, $CDCl_3$ – mixture of tautomers *enol*:*keto* = 1:1.5) *major (keto) tautomer (selected)* δ_C: 40.9 (*C*H$_2$), 110.8 (Ar*C*H), 120.1 (Ar*C*H), 124.5 (Ar*C*H), 125.2 (Ar*C*H), 127.0 (Ar*C*H), 127.2 (Ar*C*H), 133.9 (Ar*C*H), 140.9 (Ar*C*), 141.3 (Ar*C*), 151.3 (Ar*C*), 162.3 (Ar*C*), 186.4 (C=O); *minor (enol) tautomer* δ_C: 83.9 (enol-*C*H), 110.3 (Ar*C*H), 117.9 (Ar*C*H), 124.1 (Ar*C*H), 124.7 (Ar*C*H), 125.0 (Ar*C*H), 126.0 (Ar*C*H), 126.6 (Ar*C*H), 137.0 (Ar*C*), 140.0 (Ar*C*), 148.8 (Ar*C*), 160.3 (Ar*C*), 165.8 (enol-*C*OH); **IR** (neat) ν_{max} cm^{-1} 1626, 1533, 1512, 1454, 1418, 1273, 1248, 1221, 1155; **HRMS** $(NSI)^+$ calculated for $C_{13}H_{10}O_2NS^+$ ($[M+H]^+$) requires 244.0427; found 244.0428 (+0.5 ppm).

2-(Benzo[*d*]oxazol-2-yl)-1-(4-(trifluoromethyl)phenyl)ethan-1-one **S13**

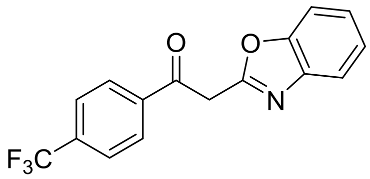

According to **General Procedure B**, triethylamine (2.0 mL, 14.4 mmol), 2-methylbenzoxazole (0.48 mL, 4.0 mmol), 4-(trifluoromethyl)benzoyl chloride (1.78 mL, 12.0 mmol) and MeCN (16 mL), followed by KOH (560 mg, 10 mmol), and MeOH (15 mL) gave the crude product which was purified by recrystallisation from hot EtOH: the crude product was dissolved in a minimal amount of boiling EtOH and then cooled in a freezer (−20 °C) overnight. The precipitate was collected by filtration, washed with ice-cold EtOH and dried under high vacuum to give *2-(benzo[d]oxazol-2-yl)-1-(4-(trifluoromethyl)phenyl)ethan-1-one* **S13** as shiny pale yellow/green plates (1.01 g, 3.31 mmol, 83%).

m.p. 134-136 °C (EtOH); **^{1}H NMR** (500 MHz, $CDCl_3$ – mixture of tautomers *enol*:*keto* = 5.5:1) *major (enol) tautomer* δ_H: 6.26 (enol-C*H*), 7.28-7.38 (2H, m, ArC*H*), 7.51 (1H, app d, *J* 7.9, ArC*H*), 7.64 (1H, app d, *J* 7.8, ArC*H*), 7.70 (2H, app d, *J* 8.2, Ar_{CF3}C(2,6)*H*), 7.97 (2H, app d, *J* 8.2, Ar_{CF3}C(3,5)*H*); *minor (keto) tautomer (selected)* δ_H: 4.66 (2H, s, C*H*$_2$), 7.77 (2H, app d, *J* 8.0, Ar_{CF3}C(2,6)*H*), 8.17 (2H, app d, *J* 8.0, Ar_{CF3}C(3,5)*H*); **^{13}C{^{1}H} NMR** (126 MHz, $CDCl_3$ – mixture of tautomers *enol*:*keto* = 5.5:1) *major (enol) tautomer* δ_C: 85.2 (enol-*C*H), 110.4 (Ar*C*H), 118.1 (Ar*C*H), 123.9 (q, $^1J_{CF}$ 271.7, *C*F$_3$), 124.6 (Ar*C*H), 124.9 (Ar*C*H), 125.6 (q, $^3J_{CF}$ 3.6, Ar_{CF3}*C*(3,5)H), 126.1 (Ar_{CF3}*C*(2,6)H), 132.0 (q, $^2J_{CF}$ 32.6, Ar_{CF3}*C*(4)), 137.4 (Ar*C*), 139.6 (Ar*C*), 148.8 (Ar*C*), 164.3 (*C*), 165.1 (*C*); *minor (keto) tautomer (selected)* δ_C: 39.9 (*C*H$_2$), 110.7 (Ar*C*H), 120.1 (Ar*C*H), 125.3 (Ar*C*H), 126.0 (q, $^3J_{CF}$ 3.6, Ar_{CF3}*C*(3,5)H), 129.0 (Ar_{CF3}*C*(2,6)H), 135.2 (q, $^2J_{CF}$ 33.3, Ar_{CF3}*C*(4)), 138.2 (Ar*C*), 141.2 (Ar*C*), 151.2 (Ar*C*), 159.7 (Ar*C*), 191.6 (C=O); **^{19}F{^{1}H} NMR** (376 MHz, $CDCl_3$ – mixture of tautomers *enol*:*keto* = 5.5:1) *major (enol) tautomer* δ_F: −62.8; *minor (keto) tautomer* δ_F: −63.2; **IR** (neat) ν_{max} cm^{-1} 1622, 1614, 1528, 1514, 1454, 1281, 1248, 1169, 1105,

1069, 1061; **HRMS** (NSI)$^+$ calculated for $C_{16}H_{11}O_2NF_3^+$ ([M+H]$^+$) requires 306.0736; found 306.0739 (+0.9 ppm).

4-(2-(Benzo[*d*]oxazol-2-yl)acetyl)benzonitrile **S14**

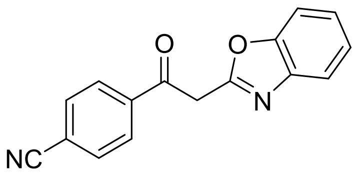

According to **General Procedure D**, oxalyl chloride (1.12 mL, 13.2 mmol), DMF (5 drops), 4-cyanobenzoic acid (1.76 g, 12 mmol) and CH_2Cl_2 (30 mL); followed by triethylamine (2.0 mL, 14.4 mmol), 2-methylbenzoxazole (0.48 mL, 4.0 mmol), and MeCN (16 mL); followed by KOH (560 mg, 7.5 mmol), THF (20 mL) and MeOH (20 mL) gave the crude product which was purified by recrystallisation from hot EtOH: the crude product was dissolved in a minimal amount of boiling EtOH and then cooled in a freezer (−20 °C) for 5 h. The precipitate was collected by filtration, washed with ice-cold EtOH and dried under high vacuum to give *4-(2-(benzo[d]oxazol-2-yl)acetyl)benzonitrile* **S14** as a yellow solid (689 mg, 2.63 mmol, 66%).

m.p. 178-180 °C (EtOH); **^{1}H NMR** (500 MHz, $CDCl_3$ – mixture of tautomers *enol*:*keto* = 11:1) *major (enol) tautomer* δ_H: 6.29 (2H, s, enol-C*H*), 7.30-7.39 (2H, m, ArC*H*), 7.53 (1H, app d, *J* 7.9, ArC*H*), 7.65 (1H, app d, *J* 7.7, ArC*H*), 7.74 (2H, app d, *J* 8.4, Ar_{CN}C(3,5)*H*), 7.97 (2H, app d, *J* 8.4, Ar_{CN}C(2,6)*H*); *minor (keto) tautomer (selected)* δ_H: 4.65 (2H, s, CH_2), 7.82 (2H, app d, *J* 8.4, Ar_{CN}C(3,5)*H*), 8.15 (2H, app d, *J* 8.4, Ar_{CN}C(2,6)*H*); **^{13}C{^{1}H} NMR** (126 MHz, $CDCl_3$ – mixture of tautomers *enol*:*keto* = 11:1) *major (enol) tautomer* δ_C: 86.2 (enol-*C*H), 110.7 (Ar*C*H), 113.9 (Ar_{CN}*C*(4)), 118.5 (Ar*C*H), 118.7 (*C*N), 125.0 (Ar*C*H), 125.2 (Ar*C*H), 126.6 (Ar_{CN}*C*(2,6)H), 132.6 (Ar_{CN}*C*(3,5)H), 138.5 (Ar*C*), 139.7 (Ar*C*), 149.1 (Ar*C*), 163.9 (*C*), 165.1 (*C*); *minor (keto) tautomer (selected)* δ_C: 40.1 (*C*H_2), 111.0 (Ar*C*H), 120.4 (Ar*C*H), 124.9 (Ar*C*H), 125.6 (Ar*C*H), 129.3 (Ar_{CN}*C*(2,6)H), 133.0 (Ar_{CN}*C*(3,5)H); **IR** (neat) ν_{max} cm^{-1} 2226 (CN), 1634, 1528n 1454, 1335, 1291, 1248, 1161, 1065; **HRMS** (NSI)$^+$ calculated for $C_{16}H_{11}O_2N_2^+$ ([M+H]$^+$) requires 263.0815; found 263.0817 (+0.7 ppm).

Reaction Optimisation

Optimisation aimed to find conditions to give highest enantioselectivity and conversion. A high ratio between dihydropyranone and dihydropyridinone products was not needed due to subsequent isomerisation. Very high substrate conversion was targeted to limit erosion of enantioselectivity through racemic, base-mediated, product formation during isomerisation step.

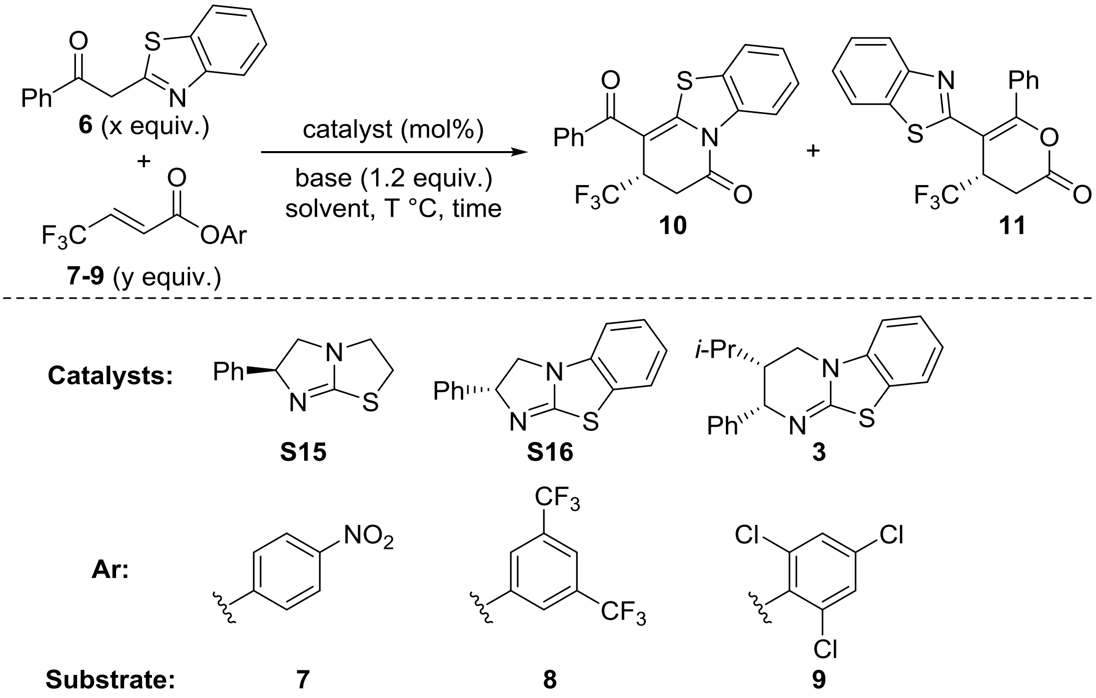

x	y	Cat (mol%)	Substrate	base	solvent	T/°C	t/h	10		11	
								%	er	%	er
1.0	1.1	**S15** (15)	**7**	*i*-Pr_2NEt	THF	r.t.	20	94	16:84	0	-
1.0	1.1	**S16** (15)	**7**	*i*-Pr_2NEt	THF	r.t.	20	73	91:9	0	-
1.0	1.1	**3** (15)	**7**	*i*-Pr_2NEt	THF	r.t.	20	96	93:7	0	-
1.0	1.1	**3** (15)	**7**	*i*-Pr_2NEt	THF	r.t.	2	85	94:6	8	ND
1.0	1.1	**3** (15)	**7**	*i*-Pr_2NEt	THF	0	2	84	94:6	10	ND
1.0	1.1	**3** (15)	**7**	*i*-Pr_2NEt	THF	−78	2	71	88:12	14	ND
1.0	1.1	**3** (15)	**7**	*i*-Pr_2NEt	CH_2Cl_2	r.t.	20	100	85:15	0	-
1.0	1.1	**3** (15)	**7**	*i*-Pr_2NEt	PhMe	r.t.	20	89	84:16	0	-
1.0	1.1	**3** (15)	**7**	*i*-Pr_2NEt	MeCN	r.t.	20	79	90:10	0	-
1.0	1.1	**3** (15)	**7**	*i*-Pr_2NEt	EtOAc	r.t.	20	99	92:8	0	-
1.0	1.1	none	**7**	*i*-Pr_2NEt	THF	r.t.	2	75	-	13	-
1.0	1.1	**3** (5)	**7**	none	THF	r.t.	20	75	95:5	23	ND
1.0	1.1	**3** (5)	**9**	none	THF	r.t.	20	54	97:3	42	97:3
1.0	1.1	**3** (5)	**8**	none	THF	r.t.	20	73	94:6	21	94:6
1.0	1.1	**3** (1)	**9**	none	THF	r.t.	20	47	97:3	38	97:3
1.0	1.1	**3** (3)	**9**	none	THF	r.t.	20	50	97:3	42	97:3
1.0	1.25	**3** (5)	**9**	none	THF	r.t.	20	54	97:3	41	97:3
1.25	1.0	**3** (5)	**9**	none	THF	r.t.	20	54	97:3	40	97:3

Dihydropyranone to Dihydropyridinone Isomerisation Studies

A mixture of (*S*)-4-benzoyl-3-(trifluoromethyl)-2,3-dihydro-1*H*-benzo[4,5]thiazolo[3,2-*a*]pyridin-1-one **10** and (*S*)-5-(benzo[*d*]thiazol-2-yl)-6-phenyl-4-(trifluoromethyl)-3,4-dihydro-2*H*-pyran-2-one **11** (53:47 ratio, 38 mg, 0.1 mmol) and 1,3,5-trimethoxybenzene (3.4 mg, 0.02 mmol) were allowed to stir in anhydrous THF (0.2 mL) at r.t. in the presence of various reagent combinations. 1,3,5-trimethoxybenzene was used as an internal standard. Periodically a small sample was removed, added to an NMR tube and blown to near-dryness using compressed air. $CDCl_3$ (~0.5 mL) was added to the NMR tube and ^{1}H NMR spectroscopic analysis was used to quantify the amount of dihydropyranone **11** and dihydropyridinone **10**. In all cases where isomerisation was observed, the total amount of **11** and **10** remained constant, with a decrease in concentration of **11** concomitant an increase in concentration of **10**. For simplicity only the concentration of **11** is provided in the following plots.

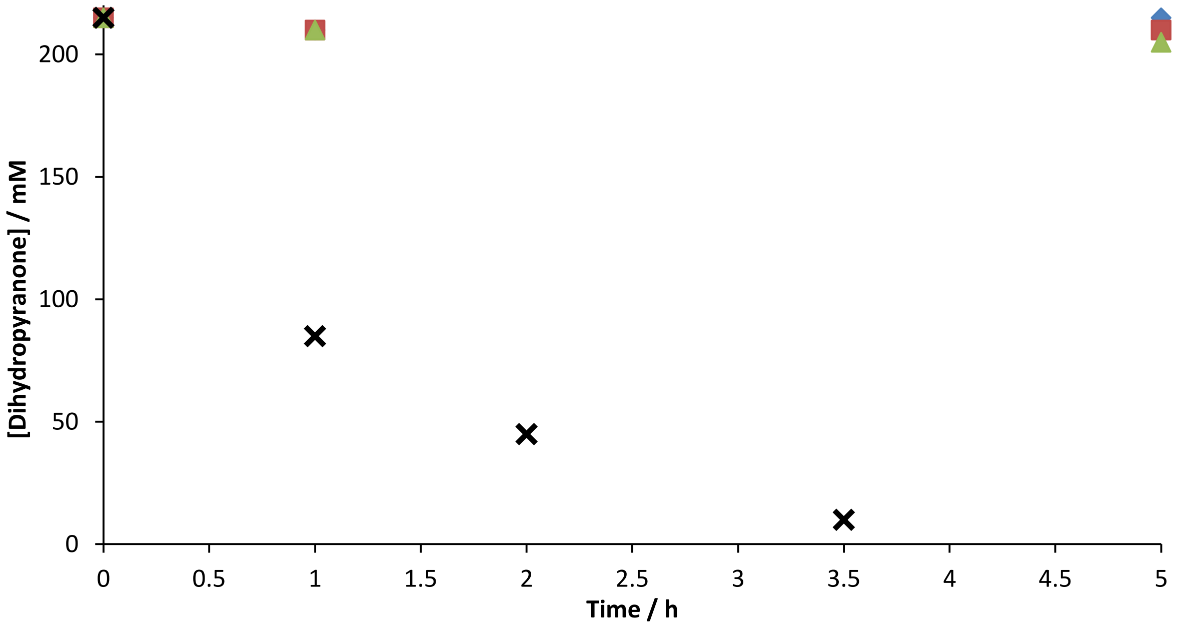

Reagent combination used: ◆ HyperBTM (25 mM); ■ *i*-Pr_2NEt (500 mM); ▲ *p*-Nitrophenol (500 mM); ✕ *p*-Nitrophenol (500 mM), *i*-Pr_2NEt (500 mM).

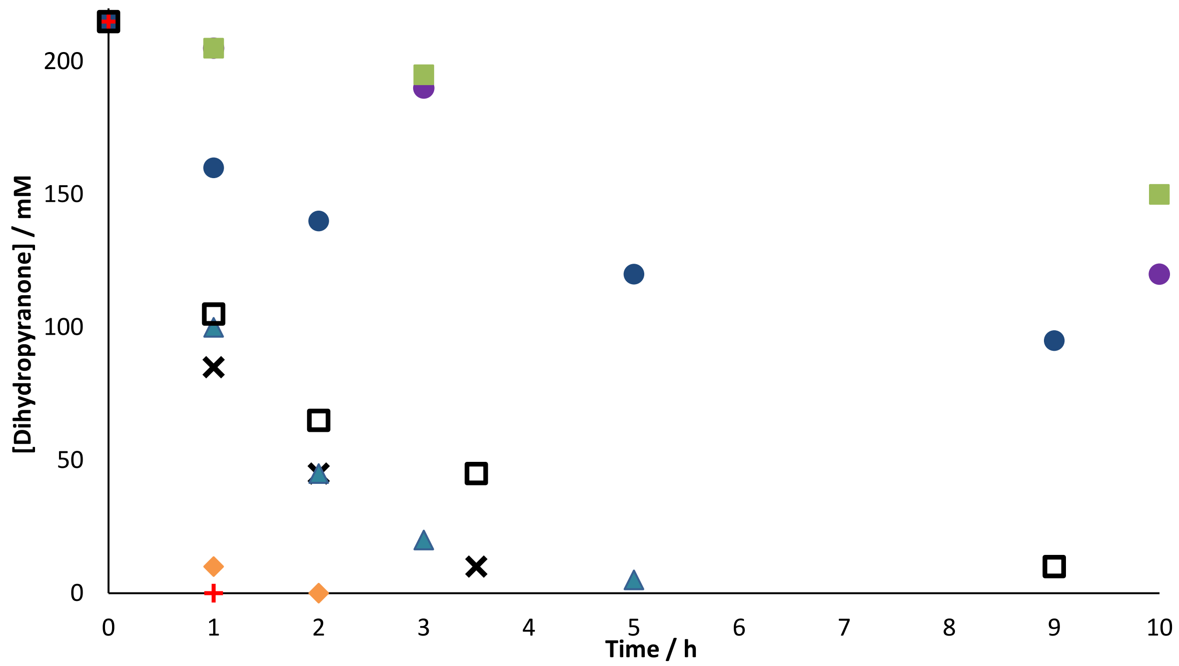

Reagent combination used: ✕ *p*-Nitrophenol (500 mM), *i*-Pr_2NEt (500 mM); ● 2,4,6-Trichlorophenol (500 mM), *i*-Pr_2NEt (500 mM); ◆ 3,4,5-Trifluorophenol (500 mM), *i*-Pr_2NEt (500 mM); ■ Pentafluorophenol (500 mM), *i*-Pr_2NEt (500 mM); ▲ 3,5-Bis(trifluoromethyl)phenol (500 mM), *i*-Pr_2NEt (500 mM); ● Phenol (500 mM), *i*-Pr_2NEt (500 mM); ✚ Thiophenol (500 mM), *i*-Pr_2NEt (500 mM); ☐ Hexafluoroisopropanol (500 mM), *i*-Pr_2NEt (500 mM).

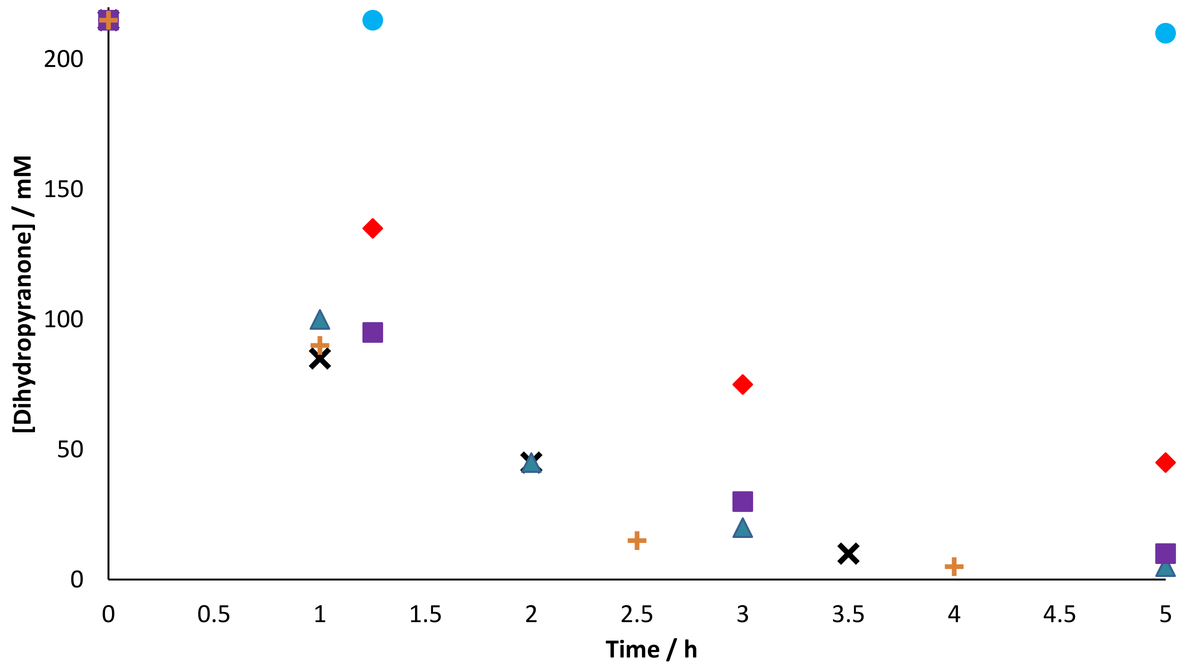

Reagent combination used: ✕ *p*-Nitrophenol (500 mM), *i*-Pr_2NEt (500 mM); ▲ 3,5-Bis(trifluoromethyl)phenol (500 mM), *i*-Pr_2NEt (500 mM); ◆ *p*-Nitrophenol (500 mM), 2,4,6-Trichlorophenol (500 mM), *i*-Pr_2NEt (500 mM);
■ 3,5-Bis(trifluoromethyl)phenol (500 mM), 2,4,6-Trichlorophenol (500 mM), *i*-Pr_2NEt (500 mM); ✚ 3,5-Bis(trifluoromethyl)phenol (100 mM), 2,4,6-Trichlorophenol (500 mM), *i*-Pr_2NEt (100 mM), reflux; ● 2,4,6-Trichlorophenol (500 mM), HyperBTM (25 mM).

Isothiourea-catalysed synthesis of fluorinated dihydropyridinones and dihydropyranones

All racemic samples were prepared by analogous methods using rac-HyperBTM as catalyst.

(*S*)-4-Benzoyl-3-(trifluoromethyl)-2,3-dihydro-1*H*-benzo[4,5]thiazolo[3,2-*a*]pyridin-1-one **10**

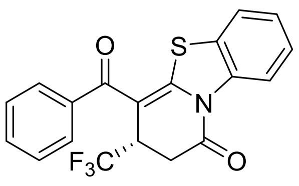

According to **General Procedure E**, 2,4,6-trichlorophenyl (*E*)-4,4,4-trifluorobut-2-enoate (70 mg, 0.22 mmol), 2-(benzo[*d*]thiazol-2-yl)-1-phenylethan-1-one (51 mg, 0.2 mmol), HyperBTM (3.1 mg, 0.01 mmol) and THF (0.4 mL); followed by 3,5-bis(trifluoromethyl)phenol (6 μL, 0.04 mmol) and *i*-Pr_2NEt (7 μL, 0.04 mmol) for 6 h (reflux), was purified [eluent: 0%→20% EtOAc in petrol; R_f = 0.23 (4:1 pet:EtOAc)] to give *(S)-4-benzoyl-3-(trifluoromethyl)-2,3-dihydro-1H-benzo[4,5]thiazolo[3,2-a]pyridin-1-one* **10** as a yellow solid (71 mg, 0.19 mmol, 95%).

m.p. 149-151 °C; $[\alpha]_D^{20}$ = +202 (*c* 1.0, $CHCl_3$); **Chiral HPLC analysis**: CHIRALPAK AD-H (20:80 *i*-PrOH:hexane, flow rate 1.0 mL min^{-1}, 254 nm, 30 °C), t_R (*R*): 12.3 min, t_R (*S*): 18.3 min, 96.6:3.4 (*S*:*R*) er; **^{1}H NMR** (500 MHz, $CDCl_3$) δ_H: 3.21 (1H, dd, *J* 17.3, 2.5, C(2)H_AH_B), 3.25 (1H, dd, *J* 17.3, 6.6, C(2)H_AH_B), 3.91-4.01 (1H, m, C(3)*H*), 7.35 (1H, app td, *J* 7.6, 1.2, ArC(7)*H*), 7.38-7.52 (6H, m, ArC(8)*H* & PhC*H*), 7.58 (1H, app dd, *J* 7.6, 1.3, ArC(6)*H*), 8.54 (1H, app d, *J* 8.4, ArC(9)*H*); **^{13}C{^{1}H} NMR** (126 MHz, $CDCl_3$) δ_C: 32.3 (*C*(2)H_2), 38.0 (q, $^2J_{CF}$ 29.0, *C*(3)HCF_3), 97.5 (*C*(4)), 117.8 (Ar*C*(9)H), 122.0 (Ar*C*(6)H), 126.2 (q, $^1J_{CF}$ 283.2, *C*F_3), 126.4 (Ar*C*(7)H), 126.9 (*C*(6a)), 127.0 (Ph*C*(3,5)H), 127.6 (Ar*C*(8)H), 128.8 (Ph*C*(2,6)H), 130.5 (Ph*C*(4)H), 136.0 (*C*(9a)), 139.5 (Ph*C*(1)), 159.4 (*C*(5)), 166.1 (N*C*(1)=O), 191.6 (*C*=O); **^{19}F{^{1}H} NMR** (376 MHz, $CDCl_3$) δ_F: −72.9; **IR** (neat) ν_{max} cm^{-1} 1736 (C=O), 1612, 1575, 1487, 1219, 1113 (C-F), 959, 750; **HRMS** $(NSI)^+$ calculated for $C_{19}H_{13}O_2NF_3S^+$ ($[M+H]^+$) requires 376.0614; found 376.0602 (−3.2 ppm).

(*S*)-5-(Benzo[*d*]thiazol-2-yl)-6-phenyl-4-(trifluoromethyl)-3,4-dihydro-2*H*-pyran-2-one **11**

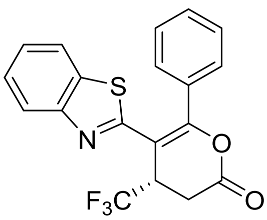

Following the procedure above with the exception of the isomerisation step using 3,5-bis(trifluoromethyl)phenol and *i*-Pr_2NEt, gave a crude material, which was purified by column chromatography (eluent: 0%→20% EtOAc in petrol) to give *(S)-5-(benzo[d]thiazol-2-yl)-6-phenyl-4-(trifluoromethyl)-3,4-dihydro-2H-pyran-2-one* **11** as a yellow solid (22 mg, 0.06 mmol, 29%).

m.p. 122-124 °C; **Chiral HPLC analysis**: CHIRALPAK AD-H (20:80 *i*-PrOH:hexane, flow rate 1.0 mL min^{-1}, 254 nm, 30 °C), t_R (*R*): 6.1 min, t_R (*S*): 24.4min, 96.6:3.4 (*S*:*R*) er; **^{1}H NMR** (500 MHz, $CDCl_3$) δ_H: 3.11 (1H, dd, *J* 17.0, 7.5, C(3)H_AH_B), 3.23 (1H, dd, *J* 17.0, 1.6, C(3)H_AH_B), 4.65-4.76 (1H, m, C(4)*H*), 7.30-7.37 (1H, m, ArC(5)*H*), 7.41-7.58 (6H, m, ArC(6)*H* & PhC*H*), 7.66 (1H, ddd, *J* 8.1, 1.1, 0.6, ArC(4)*H*), 7.98 (1H, ddd, *J* 8.2, 0.9, 0.6, ArC(7)*H*); **^{13}C{^{1}H} NMR** (126 MHz, $CDCl_3$) δ_C: 28.5 (*C*(3)H_2), 39.9 (q, $^2J_{CF}$ 29.2, *C*(4)HCF_3), 107.3 (*C*(5)), 121.4 (Ar*C*(4)H), 123.2 (Ar*C*(7)H), 125.8 (Ar*C*(5)H), 126.1 (q, $^1J_{CF}$ 282.4, *C*F_3), 126.4 (Ar*C*(6)H), 129.2 (Ph*C*(3,5)H), 130.0 (Ph*C*(2,6)H), 131.2 (Ph*C*(1)), 131.4 (Ph*C*(4)H), 135.8 (Ar*C*(3a)), 152.2 (Ar*C*(7a)), 157.4 (*C*(6)), 163.2 (Ar*C*(2)), 164.5 (*C*=O); **^{19}F{^{1}H} NMR** (376 MHz, $CDCl_3$) δ_F: −71.2; **IR** (neat) ν_{max} cm^{-1} 1790 (C=O), 1651, 1346, 1215, 1193, 995, 937; **HRMS** $(NSI)^+$ calculated for $C_{19}H_{13}O_2NF_3S^+$ ($[M+H]^+$) requires 376.0614; found 376.0610 (−1.1 ppm).

(*S*)-4-(4-Methoxybenzoyl)-3-(trifluoromethyl)-2,3-dihydro-1*H*-benzo[4,5]thiazolo[3,2-*a*]pyridin-1-one **16**

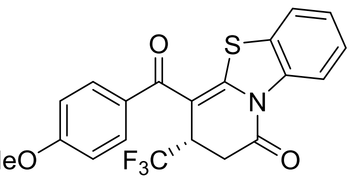

According to **General Procedure E**, 2,4,6-trichlorophenyl (*E*)-4,4,4-trifluorobut-2-enoate (35 mg, 0.11 mmol), 2-(benzo[*d*]thiazol-2-yl)-1-(4-methoxyphenyl)ethan-1-one (28 mg, 0.1 mmol), HyperBTM (3.1 mg, 0.01 mmol) and THF (0.2 mL); followed by 3,5-bis(trifluoromethyl)phenol (3 μL, 0.02 mmol) and *i*-Pr_2NEt (4 μL, 0.02 mmol) for 10 h (reflux), was purified [eluent: 0%→20% EtOAc in petrol] to give *(S)-4-(4-methoxybenzoyl)-3-(trifluoromethyl)-2,3-dihydro-1H-benzo[4,5]thiazolo[3,2-a]pyridin-1-one* **16** as a yellow solid (35 mg, 0.09 mmol, 86%).

m.p. 173-177 °C; $[\alpha]_D^{20}$ = +151 (*c* 0.5, $CHCl_3$); **Chiral HPLC analysis**: CHIRALCEL OJ-H (20:80 *i*-PrOH:hexane, flow rate 1.5 mL min^{-1}, 211 nm, 40 °C), t_R (*S*): 30.9 min, t_R (*R*): 43.9 min, 97.4:2.6 (*S*:*R*) er; **^{1}H NMR** (500 MHz, $CDCl_3$) δ_H: 3.20 (1H, dd, *J* 17.4, 2.5, C(2)H_AH_B), 3.23 (1H, dd, *J* 17.4, 6.6, C(2)H_AH_B), 3.85 (3H, s, OCH_3), 3.98-4.07 (1H, m, C(3)*H*), 6.92-6.97 (2H, m, Ar_{OMe}C(3,5)*H*), 7.32 (1H, app td, *J* 7.6, 1.1, ArC(7)*H*), 7.35-7.40 (1H, m, ArC(8)*H*), 7.41-7.45 (2H, m, Ar_{OMe}C(2,6)*H*), 7.53 (1H, app dd, *J* 7.8, 1.3, ArC(6)*H*), 8.51 (1H, app d, *J* 8.3, ArC(9)*H*); **^{13}C{^{1}H} NMR** (126 MHz, $CDCl_3$) δ_C: 32.3 (*C*(2)H_2), 38.1 (q, $^2J_{CF}$ 29.2, *C*(3)HCF_3), 55.5 (O*C*H_3), 97.6 (*C*(4)), 114.1 (Ar_{OMe}*C*(3,5)H), 117.8 (Ar*C*(9)H), 122.0 (Ar*C*(6)H), 126.2 (q, $^1J_{CF}$ 282.9, *C*F_3), 126.3 (Ar*C*(7)H), 126.9 (*C*(6a)), 127.4 (Ar*C*(8)H), 129.6 (Ar_{OMe}*C*(2,6)H), 131.8 (Ar_{OMe}*C*(1)), 136.0 (*C*(9a)), 158.9 (*C*(5)), 161.5 (Ar_{OMe}*C*(4)), 166.1 (N*C*(1)=O), 191.6 (*C*=O); **^{19}F{^{1}H} NMR** (376 MHz, $CDCl_3$) δ_F: −73.0; **IR** (neat) ν_{max} cm^{-1} 2974, 2928, 1744 (C=O), 1599, 1472, 1456, 1368, 1250, 1217, 1163, 1103; **HRMS** $(NSI)^+$ calculated for $C_{20}H_{15}O_3NF_3S^+$ ($[M+H]^+$) requires 406.0719; found 406.0721 (+0.4 ppm).

(*S*)-4-(4-Fluorobenzoyl)-3-(trifluoromethyl)-2,3-dihydro-1*H*-benzo[4,5]thiazolo[3,2-*a*]pyridin-1-one **17**

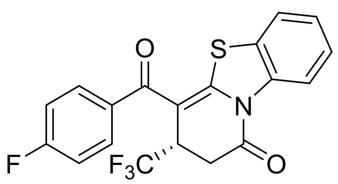

According to **General Procedure E**, 2,4,6-trichlorophenyl (*E*)-4,4,4-trifluorobut-2-enoate (70 mg, 0.22 mmol), 2-(benzo[*d*]thiazol-2-yl)-1-(4-fluorophenyl)ethan-1-one (55 mg, 0.2 mmol), HyperBTM (3.1 mg, 0.01 mmol) and THF (0.4 mL); followed by 3,5-bis(trifluoromethyl)phenol (6 μL, 0.04 mmol) and *i*-Pr_2NEt (7 μL, 0.04 mmol) for 6 h (reflux), was purified [eluent: 0%→20% EtOAc in petrol] to give *(S)-4-(4-fluorobenzoyl)-3-(trifluoromethyl)-2,3-dihydro-1H-benzo[4,5]thiazolo[3,2-a]pyridin-1-one* **17** as a yellow solid (76 mg, 0.19 mmol, 97%).

m.p. 190-193 °C; $[\alpha]_D^{20}$ = +186 (*c* 0.5, $CHCl_3$); **Chiral HPLC analysis**: CHIRALCEL OJ-H (20:80 *i*-PrOH:hexane, flow rate 1.5 mL min^{-1}, 254 nm, 40 °C), t_R (*S*): 14.5 min, t_R (*R*): 20.1 min, 96.1:3.9 (*S*:*R*) er; **^{1}H NMR** (500 MHz, $CDCl_3$) δ_H: 3.18-3.30 (2H, m, C(2)H_2), 3.87-3.98 (1H, m, C(3)*H*), 7.12-7.20 (2H, m, Ar_FC(3,5)*H*), 7.36 (1H, app td, *J* 7.6, 1.3, ArC(7)*H*), 7.39-7.50 (3H, m, ArC(8)*H* & Ar_FC(2,6)*H*), 7.56-7.61 (1H, m, ArC(6)*H*), 8.52-8.57 (1H, m, ArC(9)*H*); **^{13}C{^{1}H} NMR** (126 MHz, $CDCl_3$) δ_C: 32.3 (*C*(2)H_2), 38.1 (q, $^2J_{CF}$ 29.2, *C*(3)HCF_3), 97.2 (*C*(4)), 116.0 (d, $^2J_{CF}$ 21.8, Ar_F*C*(3,5)H), 117.9 (Ar*C*(9)H), 122.1 (Ar*C*(6)H), 126.1 (q, $^1J_{CF}$ 282.8, *C*F_3), 126.4 (Ar*C*(7)H), 126.8 (*C*(6a)), 127.7 (Ar*C*(8)H), 129.3 (d, $^3J_{CF}$ 8.7, Ar_F*C*(2,6)H), 135.6 (d, $^4J_{CF}$ 3.4, Ar_F*C*(1)), 135.9 (*C*(9a)), 159.9 (*C*(5)), 163.9 (d, $^1J_{CF}$ 250.9, Ar_F*C*(4)), 165.9 (N*C*(1)=O), 190.4 (*C*=O); **^{19}F{^{1}H} NMR** (376 MHz, $CDCl_3$) δ_F: −109.1 (Ar*F*), −72.9 (CF_3); **IR** (neat) ν_{max} cm^{-1} 2988, 1726 (C=O), 1609, 1481, 1456, 1375, 1348, 1331, 1261, 1215, 1163, 1155, 1111; **HRMS** $(NSI)^+$ calculated for $C_{19}H_{12}O_2NF_4S^+$ ($[M+H]^+$) requires 394.0519; found 394.0521 (+0.4 ppm).

(*S*)-4-(4-Bromobenzoyl)-3-(trifluoromethyl)-2,3-dihydro-1*H*-benzo[4,5]thiazolo[3,2-*a*]pyridin-1-one **18**

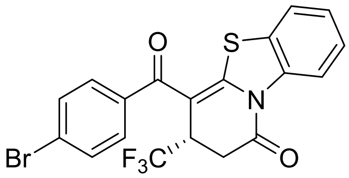

According to **General Procedure E**, 2,4,6-trichlorophenyl (*E*)-4,4,4-trifluorobut-2-enoate (70 mg, 0.22 mmol), 2-(benzo[*d*]thiazol-2-yl)-1-(4-bromophenyl)ethan-1-one (66 mg, 0.2 mmol), HyperBTM (3.1 mg, 0.01 mmol) and THF (0.4 mL); followed by 3,5-bis(trifluoromethyl)phenol (6 μL, 0.04 mmol) and *i*-Pr_2NEt (7 μL, 0.04 mmol) for 6 h (reflux), was purified [eluent: 0%→20% EtOAc in petrol] to give *(S)-4-(4-bromobenzoyl)-3-(trifluoromethyl)-2,3-dihydro-1H-benzo[4,5]thiazolo[3,2-a]pyridin-1-one* **18** as a yellow solid (77 mg, 0.17 mmol, 85%).

m.p. 119-127 °C; $[\alpha]_D^{20}$ = +143 (*c* 0.5, $CHCl_3$); **Chiral HPLC analysis**: CHIRALPAK AD-H (20:80 *i*-PrOH:hexane, flow rate 1.0 mL min^{-1}, 211 nm, 30 °C), t_R (*S*): 21.0 min, t_R (*R*): 24.5 min, 95.6:4.4 (*S*:*R*) er; **^{1}H NMR** (500 MHz, $CDCl_3$) δ_H: 3.18-3.29 (2H, m, C(2)H_2), 3.84-3.93 (1H, m, C(3)*H*), 7.30-7.34 (2H, m, Ar_{Br}C(2,6)*H*), 7.37 (1H, app td, *J* 7.6, 1.1, ArC(7)*H*), 7.43 (1H, app td, *J* 7.5, 1.3, ArC(8)*H*), 7.56-7.63

(3H, m, ArC(6)*H* & Ar_FC(3,5)*H*), 8.54 (1H, app d, *J* 8.2, ArC(9)*H*); **^{13}C{^{1}H} NMR** (126 MHz, $CDCl_3$) δ_C: 32.3 (*C*(2)H_2), 38.0 (q, $^2J_{CF}$ 29.2, *C*(3)HCF_3), 97.1 (*C*(4)), 117.9 (Ar*C*(9)H), 122.1 (Ar*C*(6)H), 124.9 (Ar_{Br}*C*(4)), 126.1 (q, $^1J_{CF}$ 282.8, *C*F_3), 126.5 (Ar*C*(7)H), 126.7 (*C*(6a)), 127.7 (Ar*C*(8)H), 128.6 (Ar_{Br}*C*(2,6)H), 132.1 (Ar_{Br}*C*(3,5)H), 135.9 (*C*(9a)), 138.2 (Ar_{Br}*C*(1)), 160.1 (*C*(5)), 165.9 (N*C*(1)=O), 190.3 (*C*=O); **^{19}F{^{1}H} NMR** (376 MHz, $CDCl_3$) δ_F: −72.9; **IR** (neat) ν_{max} cm^{-1} 2976, 2934, 1734 (C=O), 1603, 1473, 1456, 1373, 1261, 1213, 1159, 1115; **HRMS** $(APCI)^+$ calculated for $C_{19}H_{12}O_2NF_3{}^{79}BrS^+$ ($[M+H]^+$) requires 453.9719; found 453.9725 (+1.3 ppm).

(*S*)-4-(4-Nitrobenzoyl)-3-(trifluoromethyl)-2,3-dihydro-1*H*-benzo[4,5]thiazolo[3,2-*a*]pyridin-1-one **19**

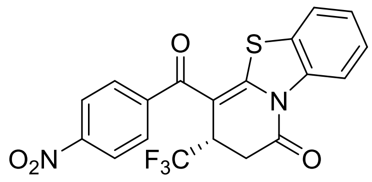

According to *a modification to* **General Procedure E**, 2,4,6-trichlorophenyl (*E*)-4,4,4-trifluorobut-2-enoate (70 mg, 0.22 mmol), 2-(benzo[*d*]thiazol-2-yl)-1-(4-nitrophenyl)ethan-1-one (60 mg, 0.2 mmol), HyperBTM (3.1 mg, 0.01 mmol) THF (0.4 mL) and DMF (5 drops); followed by 3,5-bis(trifluoromethyl)phenol (6 μL, 0.04 mmol) and *i*-Pr_2NEt (7 μL, 0.04 mmol) for 1.5 h (reflux). Upon completion and cooling to r.t., NaOH (1 M, 2 mL) added and mixture stirred for 10 min. EtOAc (20 mL) and NaOH (1 M, 10 mL) added and layers separated. The organic layer was washed sequentially with NaOH (1 M, 3 × 10 mL), saturated aqueous NH_4Cl (2 × 10 mL) and brine (10 mL), dried ($MgSO_4$), filtered and concentrated *in vacuo* to give *(S)-4-(4-nitrobenzoyl)-3-(trifluoromethyl)-2,3-dihydro-1H-benzo[4,5]thiazolo[3,2-a]pyridin-1-one* **19** as an yellow solid (82 mg, 0.2 mmol, 98%).

m.p. 250-254 °C; $[\alpha]_D^{20}$ = +96.4 (*c* 0.5, $CHCl_3$); **Chiral HPLC analysis**: CHIRALPAK AD-H (20:80 *i*-PrOH:hexane, flow rate 1.5 mL min^{-1}, 254 nm, 40 °C), t_R (*S*): 17.6 min, t_R (*R*): 34.7 min, 87.0:13.0 (*S*:*R*) er; **^{1}H NMR** (500 MHz, $CDCl_3$) δ_H: 3.20-3.33 (2H, m, C(2)H_2), 3.71-3.84 (1H, m, C(3)*H*), 7.40 (1H, app td, *J* 7.6, 1.0, ArC(7)*H*), 7.47 (1H, app td, *J* 7.7, 1.2, ArC(8)*H*), 7.58-7.67 (3H, m, ArC(6)*H* & Ar_{NO2}C(2,6)*H*), 8.31-8.38 (2H, m, Ar_{NO2}C(3,5)*H*), 8.57 (1H, app d, *J* 8.2, ArC(9)*H*); **^{13}C{^{1}H} NMR** (126 MHz, $CDCl_3$) δ_C: 32.3 (*C*(2)H_2), 38.0 (q, $^2J_{CF}$ 29.2, *C*(3)HCF_3), 96.7 (*C*(4)), 118.0 (Ar*C*(9)H), 122.2 (Ar*C*(6)H), 124.2 (Ar_{NO2}*C*(3,5)H), 126.0 (q, $^1J_{CF}$ 283.0, *C*F_3), 126.7 (Ar*C*(7)H), 126.6 (*C*(6a)), 128.01 (Ar*C*(8)H), 128.03 (Ar_{NO2}*C*(2,6)H), 135.9 (*C*(9a)), 145.4 (Ar_{NO2}*C*(1)), 148.7 (Ar_{NO2}*C*(4)), 161.1 (*C*(5)), 165.6 (N*C*(1)=O), 188.9 (*C*=O); **^{19}F{^{1}H} NMR** (376 MHz, $CDCl_3$) δ_F: −72.8; **IR** (neat) ν_{max} cm^{-1} 2965, 2928, 1742 (C=O), 1595, 1516, 1470, 1452, 1277, 1258, 1128; **HRMS** $(APCI)^+$ calculated for $C_{19}H_{12}O_4N_2F_3S^+$ ($[M+H]^+$) requires 421.0464; found 421.0468 (+1.0 ppm).

(*S*)-4-(2-Iodobenzoyl)-3-(trifluoromethyl)-2,3-dihydro-1*H*-benzo[4,5]thiazolo[3,2-*a*]pyridin-1-one **20**

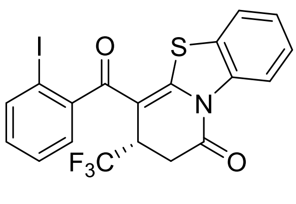

According to **General Procedure E**, 2,4,6-trichlorophenyl (*E*)-4,4,4-trifluorobut-2-enoate (70 mg, 0.22 mmol), 2-(benzo[*d*]thiazol-2-yl)-1-(2-iodophenyl)ethan-1-one (76 mg, 0.2 mmol), HyperBTM (3.1 mg, 0.01 mmol) and THF (0.4 mL); followed by 3,5-bis(trifluoromethyl)phenol (6 μL, 0.04 mmol) and *i*-Pr_2NEt (7 μL, 0.04 mmol) for 5 h (reflux), was purified [eluent: 0%→20% EtOAc in petrol] to give *(S)-4-(2-iodobenzoyl)-3-(trifluoromethyl)-2,3-dihydro-1H-benzo[4,5]thiazolo[3,2-a]pyridin-1-one* **20** as a yellow solid (94 mg, 0.19 mmol, 94%).

m.p. 210-213 °C; $[\alpha]_D^{20}$ = +260 (*c* 0.5, $CHCl_3$); **Chiral HPLC analysis**: CHIRALPAK AD-H (10:90 *i*-PrOH:hexane, flow rate 1.5 mL min^{-1}, 211 nm, 40 °C), t_R (*R*): 7.2 min, t_R (*S*): 11.8 min, 96.7:3.3 (*S*:*R*) er; **^{1}H NMR** (500 MHz, $CDCl_3$) δ_H: 3.19 (1H, app d, *J* 15.6, C(2)H_AH_B), 3.31-3.57 (2H, m, C(2)H_AH_B & C(3)*H*), 7.15 (1H, app td, *J* 7.5, 1.7, Ar_IC(4)*H*), 7.28-7.51 (4H, m, ArC(7,8)*H* & Ar_IC(5,6)*H*), 7.61 (1H, app dd, *J* 7.8, 1.1, ArC(6)*H*), 7.85 (1H, app d, *J* 7.9, Ar_IC(3)*H*), 8.54 (1H, app dd, *J* 8.2, 1.1, ArC(9)*H*); **^{13}C{^{1}H} NMR** (126 MHz, $CDCl_3$) *some peaks weak/broad (noted) – restricted rotation?* δ_C: 32.7 (*C*(2)H_2), 38.5 (q, $^2J_{CF}$ 29.0, *C*(3)HCF_3), 92.3 (br, Ar_I*C*(2)) 97.3 (br, *C*(4)), 117.9 (Ar*C*(9)H), 122.2 (Ar*C*(6)H), 126.1 (q, $^1J_{CF}$ 283.0, *C*F_3), 126.4 (Ar*C*(7)H), 127.0 (*C*(6a)), 127.7 (Ar*C*(8)H), 128.9 (br, Ar_I*C*(5)H), 129.3 (br, Ar_I*C*(6)H), 131.0 (Ar_I*C*(4)H), 136.0 (*C*(9a)), 138.7 (br, Ar_I*C*(3)H), 144.2 (br, Ar_I*C*(1)), 162.3 (br, *C*(5)), 166.2 (N*C*(1)=O), 191.3 (br, *C*=O); **^{19}F{^{1}H} NMR** (376 MHz, $CDCl_3$) δ_F: −72.2; **IR** (neat) ν_{max} cm^{-1} 2963, 1732 (C=O), 1618, 1489, 1456, 1368, 1346, 1260, 1217, 1163, 1136, 1105; **HRMS** $(APCI)^+$ calculated for $C_{19}H_{12}O_2NF_3IS^+$ ($[M+H]^+$) requires 501.9580; found 501.9588 (+1.6 ppm).

(*S*)-4-(1-Naphthoyl)-3-(trifluoromethyl)-2,3-dihydro-1*H*-benzo[4,5]thiazolo[3,2-*a*]pyridin-1-one **21**

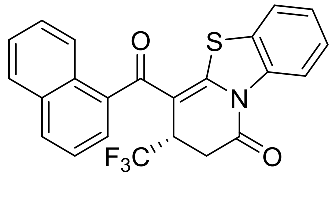

According to **General Procedure E**, 2,4,6-trichlorophenyl (*E*)-4,4,4-trifluorobut-2-enoate (70 mg, 0.22 mmol), 2-(benzo[*d*]thiazol-2-yl)-1-(naphthalen-1-yl)ethan-1-one (61 mg, 0.2 mmol), HyperBTM (6.2 mg, 0.02 mmol) and THF (0.4 mL); followed by 3,5-bis(trifluoromethyl)phenol (6 μL, 0.04 mmol) and *i*-Pr_2NEt (7 μL, 0.04 mmol) for 8 h (reflux), was purified [eluent: 0%→20% EtOAc in petrol] to give *(S)-4-(1-naphthoyl)-3-(trifluoromethyl)-2,3-dihydro-1H-benzo[4,5]thiazolo[3,2-a]pyridin-1-one* **21** as a pale yellow solid (79 mg, 0.19 mmol, 93%).

m.p. 203-205 °C; $[\alpha]_D^{20}$ = +308 (*c* 0.5, $CHCl_3$); **Chiral HPLC analysis**: CHIRALPAK AD-H (10:90 *i*-PrOH:hexane, flow rate 1.5 mL min^{-1}, 254 nm, 40 °C), t_R (*R*): 11.4 min, t_R (*S*): 17.6 min, 96.0:4.0 (*S*:*R*) er; **^{1}H NMR** (500 MHz, $CDCl_3$) δ_H: 3.01-3.29 (2H, m, C(2)H_2), 3.54 (1H, br, s C(3)*H*), 7.37 (1H, app td, *J*

7.6, 1.2, ArC(7)*H*), 7.40-7.58 (5H, m, ArC(8)*H* & 4 × NpC*H*), 7.61 (1H, app dd, *J* 7.7, 1.2, ArC(6)*H*), 7.80 (1H, br s, NpC*H*), 7.86-7.98 (2H, m, 2 × NpC*H*), 8.55 (1H, app dd, *J* 8.2, 0.8, ArC(9)*H*); **^{13}C{^{1}H} NMR** (126 MHz, $CDCl_3$) *some peaks missing/very broad – restricted rotation?* δ_C: 32.5 (*C*(2)H_2), 99.3 (br, *C*(4)), 117.9 (Ar*C*(9)H), 122.1 (Ar*C*(6)H), 126.0 (q, $^{1}J_{CF}$ 282.9, *C*F_3), 126.4 (Ar*C*(7)H), 126.6 (br, Np*C*H), 126.9 (*C*(6a)), 127.3 (Np*C*H), 127.6 (Ar*C*(8)H), 128.9 (br, Np*C*H), 129.4 (br, Np*C*), 130.2 (br, Np*C*H), 136.0 (*C*(9a)), 136.9 (br, Np*C*), 166.2 (N*C*(1)=O), 191.9 (*C*=O); **^{19}F{^{1}H} NMR** (376 MHz, $CDCl_3$) δ_F: −72.5 (very broad); **IR** (neat) ν_{max} cm^{-1} 3057, 2972, 1734 (C=O), 1575, 1558, 1506, 1472, 1456, 1341, 1267, 1206, 1153, 1140, 1099; **HRMS** (APCI)$^+$ calculated for $C_{23}H_{15}O_2NF_3S^+$ ([M+H]$^+$) requires 426.0770; found 426.0770 (−0.0 ppm).

(*S*)-4-Picolinoyl-3-(trifluoromethyl)-2,3-dihydro-1*H*-benzo[4,5]thiazolo[3,2-*a*]pyridin-1-one **22**

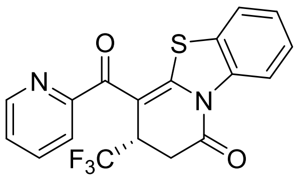

According to **General Procedure E**, 2,4,6-trichlorophenyl (*E*)-4,4,4-trifluorobut-2-enoate (53 mg, 0.165 mmol), 2-(benzo[*d*]thiazol-2-yl)-1-(pyridin-2-yl)ethan-1-one (38 mg, 0.15 mmol), HyperBTM (2.3 mg, 0.0075 mmol) and THF (0.3 mL); followed by 3,5-bis(trifluoromethyl)phenol (4.5 μL, 0.03 mmol) and *i*-Pr_2NEt (5 μL, 0.03 mmol) for 1.5 h (reflux), was purified [eluent: 0%→60% EtOAc in petrol] to give *(S)-4-picolinoyl-3-(trifluoromethyl)-2,3-dihydro-1H-benzo[4,5]thiazolo[3,2-a]pyridin-1-one* **22** as a yellow/brown solid (52 mg, 0.14 mmol, 92%).

m.p. 188-190 °C; $[\alpha]_D^{20}$ = +189 (*c* 0.5, $CHCl_3$); **Chiral HPLC analysis**: CHIRALPAK AD-H (15:85 *i*-PrOH:hexane, flow rate 1.5 mL min^{-1}, 254 nm, 40 °C), t_R (*R*): 5.4 min, t_R (*S*): 9.6 min, 96.2:3.8 (*S*:*R*) er; **^{1}H NMR** (500 MHz, $CDCl_3$) δ_H: 3.24 (1H, dd, *J* 17.4, 1.5, C(2)H_AH_B), 3.35 (1H, dd, *J* 17.4, 7.6, C(2)H_AH_B), 5.83-5.96 (1H, m, C(3)*H*), 7.35 (1H, app td, *J* 7.6, 1.2, ArC(7)*H*), 7.39-7.45 (2H, m, ArC(8)*H* & PyC5*H*), 7.60 (1H, app dd, *J* 7.6, 1.2, ArC(6)*H*), 7.88 (1H, app td, *J* 7.7, 1.8, PyC(4)*H*), 8.01 (1H, app dt, *J* 7.9, 1.0, PyC(3)*H*), 8.55-8.62 (2H, m, ArC(9)*H* & PyC(6)*H*); **^{13}C{^{1}H} NMR** (126 MHz, $CDCl_3$) δ_C: 32.1 (*C*(2)H_2), 35.8 (q, $^{2}J_{CF}$ 28.8, *C*(3)HCF_3), 98.6 (*C*(4)), 117.8 (Ar*C*(9)H), 122.0 (Ar*C*(6)H), 124.6 (Py*C*(3)H), 125.6 (Py*C*(5)H), 126.2 (Ar*C*(7)H), 126.7 (q, $^{1}J_{CF}$ 282.5, *C*F_3), 127.3 (*C*(6a)), 127.6 (Ar*C*(8)H), 136.1 (*C*(9a)), 137.5 (Py*C*(4)H), 147.6 (Py*C*(6)H), 156.8 (Py*C*(2)), 160.4 (*C*(5)), 166.8 (N*C*(1)=O), 185.5 (*C*=O); **^{19}F{^{1}H} NMR** (376 MHz, $CDCl_3$) δ_F: −72.8; **IR** (neat) ν_{max} cm^{-1} 2967, 2924, 1732 (C=O), 1603, 1580, 1560, 1472, 1456, 1368, 1287, 1221, 1159, 1111; **HRMS** (NSI)$^+$ calculated for $C_{18}H_{12}O_2N_2F_3S^+$ ([M+H]$^+$) requires 377.0566; found 377.0568 (+0.5 ppm).

(*S*)-4-(Thiophene-2-carbonyl)-3-(trifluoromethyl)-2,3-dihydro-1*H*-benzo[4,5]thiazolo[3,2-*a*]pyridin-1-one **23**

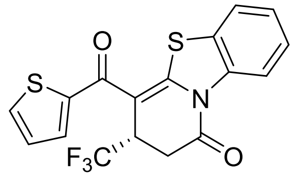

According to **General Procedure E**, 2,4,6-trichlorophenyl (*E*)-4,4,4-trifluorobut-2-enoate (70 mg, 0.22 mmol), 2-(benzo[*d*]thiazol-2-yl)-1-(thiophen-2-yl)ethan-1-one (52 mg, 0.2 mmol), HyperBTM (3.1 mg, 0.01 mmol) and THF (0.4 mL); followed by 3,5-bis(trifluoromethyl)phenol (6 μL, 0.04 mmol) and *i*-Pr_2NEt (7 μL, 0.04 mmol) for 2 h (reflux), was purified [eluent: 0%→20% EtOAc in petrol] to give *(S)-4-(thiophene-2-carbonyl)-3-(trifluoromethyl)-2,3-dihydro-1H-benzo[4,5]thiazolo[3,2-a]pyridin-1-one* **23** as a yellow solid (71 mg, 0.19 mmol, 93%).

m.p. 157-159 °C; $[\alpha]_D^{20}$ = +141 (*c* 0.5, $CHCl_3$); **Chiral HPLC analysis**: CHIRALPAK AD-H (20:80 *i*-PrOH:hexane, flow rate 1.5 mL min^{-1}, 254 nm, 40 °C), t_R (*R*): 6.0 min, t_R (*S*): 8.4 min, 97.0:3.0 (*S*:*R*) er; **^{1}H NMR** (500 MHz, $CDCl_3$) δ_H: 3.19-3.33 (2H, m, C(2)H_2), 4.37-4.48 (1H, m, C(3)*H*), 7.15 (1H, dd, *J* 5.0, 3.8, ThC(4)*H*), 7.35 (1H, app td, *J* 7.6, 1.2, ArC(7)*H*), 7.39-7.45 (1H, m, ArC(8)*H*), 7.55-7.60 (2H, m, ArC(6)*H* & ThC(3)*H*), 7.61 (1H, app dd, *J* 5.0, 1.0, ThC(5)H), 8.54 (1H, app dd, *J* 8.3, 0.9, ArC(9)*H*); **^{13}C{^{1}H} NMR** (126 MHz, $CDCl_3$) δ_C: 32.5 (*C*(2)H_2), 37.8 (q, $^2J_{CF}$ 29.4, *C*(3)HCF_3), 97.6 (*C*(4)), 117.9 (Ar*C*(9)H), 122.0 (Ar*C*(6)H), 126.4 (q, $^1J_{CF}$ 282.7, *C*F_3), 126.3 (Ar*C*(7)H), 127.4 (*C*(6a)), 127.6 (Ar*C*(8)H), 127.9 (Th*C*(4)H), 129.7 (Th*C*(3)H), 131.6 (Th*C*(5)H), 136.0 (*C*(9a)), 143.2 (Th*C*(2)), 150.5 (*C*(5)), 166.0 (N*C*(1)=O), 180.3 (*C*=O); **^{19}F{^{1}H} NMR** (376 MHz, $CDCl_3$) δ_F: −71.5; **IR** (neat) ν_{max} cm^{-1} 2984, 2911, 1730 (C=O), 1578, 1472, 1456, 1417, 1373, 1279, 1271, 1217, 1163, 1107; **HRMS** (NSI)$^+$ calculated for $C_{17}H_{11}O_2NF_3S_2^+$ ([M+H]$^+$) requires 382.0178; found 382.0178 (+0.0 ppm).

(*S*)-4-Acetyl-3-(trifluoromethyl)-2,3-dihydro-1*H*-benzo[4,5]thiazolo[3,2-*a*]pyridin-1-one **24**

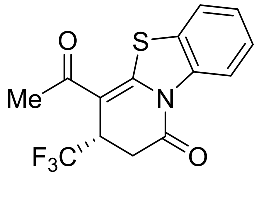

According to **General Procedure E**, 2,4,6-trichlorophenyl (*E*)-4,4,4-trifluorobut-2-enoate (70 mg, 0.22 mmol), 1-(benzo[*d*]thiazol-2-yl)propan-2-one (38 mg, 0.2 mmol), HyperBTM (3.1 mg, 0.01 mmol) and THF (0.4 mL); followed by 3,5-bis(trifluoromethyl)phenol (6 μL, 0.04 mmol) and *i*-Pr_2NEt (7 μL, 0.04 mmol) for 14 h (reflux), was purified [eluent: 0%→20% EtOAc in petrol] to give *(S)-4-acetyl-3-(trifluoromethyl)-2,3-dihydro-1H-benzo[4,5]thiazolo[3,2-a]pyridin-1-one* **24** as a yellow solid (53 mg, 0.17 mmol, 85%).

m.p. 150-151 °C; $[\alpha]_D^{20}$ = +65.0 (*c* 0.5, $CHCl_3$); **Chiral HPLC analysis**: CHIRALCEL OJ-H (20:80 *i*-PrOH:hexane, flow rate 1.5 mL min^{-1}, 254 nm, 40 °C), t_R (*R*): 9.2 min, t_R (*S*): 11.0 min, 94.2:5.8 (*S*:*R*) er; **^{1}H NMR** (400 MHz, $CDCl_3$) δ_H: 2.39 (3H, s, CH_3), 3.16 (1H, dd, *J* 17.2, 7.1, C(2)H_AH_B), 3.23 (1H, dd, *J*

17.2, 1.7, C(2)H_AH_B), 3.70-3.83 (1H, m, C(3)*H*), 7.31 (1H, app td, *J* 7.6, 1.0, ArC(7)*H*), 7.37 (1H, app td, *J* 7.4, 1.1, ArC(8)*H*), 7.52 (1H, app dd, *J* 7.6, 0.8, ArC(6)*H*), 8.48 (1H, app d, *J* 8.2, ArC(9)*H*); **^{13}C{^{1}H} NMR** (126 MHz, $CDCl_3$) δ_C: 26.2 (*C*H_3), 32.3 (*C*(2)H_2), 37.8 (q, $^2J_{CF}$ 29.4, *C*(3)HCF_3), 98.5 (*C*(4)), 117.6 (Ar*C*(9)H), 121.9 (Ar*C*(6)H), 126.4 (q, $^1J_{CF}$ 282.3, *C*F_3), 126.1 (Ar*C*(7)H), 127.1 (*C*(6a)), 127.3 (Ar*C*(8)H), 135.5 (*C*(9a)), 157.5 (*C*(5)), 165.9 (N*C*(1)=O), 192.3 (*C*=O); **^{19}F{^{1}H} NMR** (376 MHz, $CDCl_3$) δ_F: −72.4; **IR** (neat) ν_{max} cm^{-1} 2963, 2936, 1717 (C=O), 1628, 1489, 1458, 1373, 1348, 1267, 1256, 1217, 1163, 1109; **HRMS** (NSI)$^+$ calculated for $C_{14}H_{11}O_2NF_3S^+$ ([M+H]$^+$) requires 314.0457; found 314.0458 (+0.3 ppm).

(*S*)-4-Benzoyl-7-fluoro-3-(trifluoromethyl)-2,3-dihydro-1*H*-benzo[4,5]thiazolo[3,2-*a*]pyridin-1-one **25**

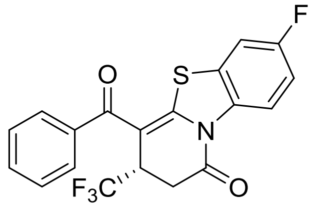

According to **General Procedure E**, 2,4,6-trichlorophenyl (*E*)-4,4,4-trifluorobut-2-enoate (70 mg, 0.22 mmol), 2-(6-fluorobenzo[*d*]thiazol-2-yl)-1-phenylethan-1-one (54 mg, 0.2 mmol), HyperBTM (3.1 mg, 0.01 mmol) and THF (0.4 mL); followed by 3,5-bis(trifluoromethyl)phenol (6 μL, 0.04 mmol) and *i*-Pr_2NEt (7 μL, 0.04 mmol) for 4 h (reflux), was purified [eluent: 0%→20% EtOAc in petrol] to give *(S)-4-benzoyl-7-fluoro-3-(trifluoromethyl)-2,3-dihydro-1H-benzo[4,5]thiazolo[3,2-a]pyridin-1-one* **25** as a pale yellow solid (72 mg, 0.18 mmol, 92%).

m.p. 202-204 °C; $[\alpha]_D^{20}$ = +203 (*c* 0.5, $CHCl_3$); **Chiral HPLC analysis**: CHIRALCEL OJ-H (20:80 *i*-PrOH:hexane, flow rate 1.5 mL min^{-1}, 254 nm, 40 °C), t_R (*S*): 24.6 min, t_R (*R*): 28.7 min, 97.0:3.0 (*S*:*R*) er; **^{1}H NMR** (400 MHz, $CDCl_3$) δ_H: 3.17-3.30 (2H, m, C(2)H_2), 3.91-4.03 (1H, m, C(3)*H*), 7.11 (1H, app td, *J* 8.7, 2.7, ArC(8)*H*), 7.29 (1H, dd, *J* 7.4, 2.6, ArC(6)*H*), 7.42-7.53 (5H, m, PhC*H*), 8.51 (1H, d, *J* 9.2, 4.6, ArC(9)*H*); **^{13}C{^{1}H} NMR** (126 MHz, $CDCl_3$) δ_C: 32.1 (*C*(2)H_2), 38.0 (q, $^2J_{CF}$ 29.3, *C*(3)HCF_3), 97.8 (*C*(4)), 109.2 (d, $^2J_{CF}$ 26.6, Ar*C*(6)H), 114.5 (d, $^2J_{CF}$ 23.4, Ar*C*(8)H), 119.0 (d, $^3J_{CF}$ 8.4, Ar*C*(9)H), 126.1 (q, $^1J_{CF}$ 283.2, *C*F_3), 127.0 (Ph*C*(3,5)H), 128.8 (Ph*C*(2,6)H), 129.0 (d, $^3J_{CF}$ 9.2, *C*(6a)), 130.6 (Ph*C*(4)H), 132.3 (d, $^4J_{CF}$ 2.0, *C*(9a)), 139.2 (Ph*C*(1)), 159.4 (*C*(5)), 160.6 (d, $^1J_{CF}$ 248.0, Ar*C*(7)), 165.8 (N*C*(1)=O), 191.6 (*C*=O); **^{19}F{^{1}H} NMR** (376 MHz, $CDCl_3$) δ_F: −114.4 (Ar*F*), −72.9 (C*F*$_3$); **IR** (neat) ν_{max} cm^{-1} 2956, 2922, 1719 (C=O), 1622, 1595, 1506, 1489, 1472, 1368, 1352, 1325, 1292, 1256, 1227, 1163, 1132, 1107; **HRMS** (NSI)$^+$ calculated for $C_{19}H_{12}O_3NF_4S^+$ ([M+H]$^+$) requires 394.0519; found 394.0520 (+0.2 ppm).

(S)-4-Benzoyl-7-bromo-3-(trifluoromethyl)-2,3-dihydro-1*H*-benzo[4,5]thiazolo[3,2-*a*]pyridin-1-one **26**

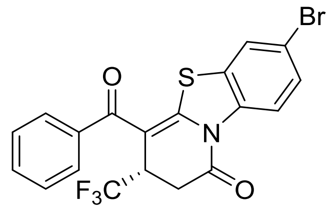

According to **General Procedure E**, 2,4,6-trichlorophenyl (*E*)-4,4,4-trifluorobut-2-enoate (70 mg, 0.22 mmol), 2-(6-bromobenzo[*d*]thiazol-2-yl)-1-phenylethan-1-one (66 mg, 0.2 mmol), HyperBTM (3.1 mg, 0.01 mmol) and THF (0.4 mL); followed by 3,5-bis(trifluoromethyl)phenol (6 μL, 0.04 mmol) and *i*-Pr_2NEt (7 μL, 0.04 mmol) for 4 h (reflux), was purified [eluent: 0%→20% EtOAc in petrol] to give *(S)-4-benzoyl-7-bromo-3-(trifluoromethyl)-2,3-dihydro-1H-benzo[4,5]thiazolo[3,2-a]pyridin-1-one* **26** as a pale yellow solid (80 mg, 0.18 mmol, 88%).

m.p. 206-209 °C; $[\alpha]_D^{20}$ = +144 (*c* 0.5, $CHCl_3$); **Chiral HPLC analysis**: CHIRALCEL OJ-H (20:80 *i*-PrOH:hexane, flow rate 1.5 mL min^{-1}, 254 nm, 40 °C), t_R (*S*): 27.1 min, t_R (*R*): 36.2 min, 96.5:3.5 (*S*:*R*) er; **^{1}H NMR** (400 MHz, $CDCl_3$) δ_H: 3.16-3.30 (2H, m, C(2)H_2), 3.92-4.04 (1H, m, C(3)*H*), 7.41-7.55 (6H, m, PhC*H* & ArC(8)*H*), 7.70 (1H, dd, *J* 2.0, 0.3, ArC(6)*H*), 8.40 (1H, dd, *J* 8.9, 0.3, ArC(9)*H*); **^{13}C{^{1}H} NMR** (126 MHz, $CDCl_3$) δ_C: 32.2 (*C*(2)H_2), 38.0 (q, $^2J_{CF}$ 29.3, *C*(3)HCF_3), 97.9 (*C*(4)), 118.9 (Ar*C*(9)H), 119.4 (Ar*C*(7)), 124.7 (Ar*C*(6)H), 126.0 (q, $^1J_{CF}$ 283.2, *C*F_3), 127.0 (Ph*C*(3,5)H), 128.9 (Ph*C*(2,6)H), 129.3 (*C*(6a)), 130.6 (Ar*C*(8)H), 130.7 (Ph*C*(4)H), 135.1 (*C*(9a)), 139.1 (Ph*C*(1)), 158.8 (*C*(5)), 165.9 (N*C*(1)=O), 191.7 (*C*=O); **^{19}F{^{1}H} NMR** (376 MHz, $CDCl_3$) δ_F: −72.9; **IR** (neat) ν_{max} cm^{-1} 3069, 2970, 2924, 1734 (C=O), 1717, 1622, 1489, 1456, 1369, 1350, 1325, 1258, 1215, 1155, 1117; **HRMS** $(APCI)^+$ calculated for $C_{19}H_{12}O_3NF_3{}^{79}BrS^+$ ($[M+H]^+$) requires 453.9719; found 453.9724 (+1.1 ppm).

(S)-4-Benzoyl-7-methoxy-3-(trifluoromethyl)-2,3-dihydro-1*H*-benzo[4,5]thiazolo[3,2-*a*]pyridin-1-one **27**

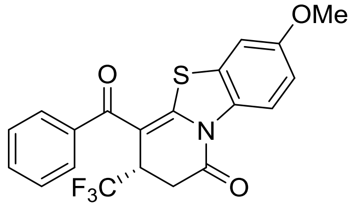

According to **General Procedure E**, 2,4,6-trichlorophenyl (*E*)-4,4,4-trifluorobut-2-enoate (70 mg, 0.22 mmol), 2-(6-methoxybenzo[*d*]thiazol-2-yl)-1-phenylethan-1-one (57 mg, 0.2 mmol), HyperBTM (6.2 mg, 0.02 mmol) and THF (0.4 mL); followed by 3,5-bis(trifluoromethyl)phenol (6 μL, 0.04 mmol) and *i*-Pr_2NEt (7 μL, 0.04 mmol) for 10 h (reflux), was purified [eluent: 0%→20% EtOAc in petrol] to give *(S)-4-benzoyl-7-methoxy-3-(trifluoromethyl)-2,3-dihydro-1H-benzo[4,5]thiazolo[3,2-a]pyridin-1-one* **27** as a yellow solid (70 mg, 0.17 mmol, 86%).

m.p. 193-195 °C; $[\alpha]_D^{20}$ = +203 (*c* 0.5, $CHCl_3$); **Chiral HPLC analysis**: CHIRALCEL OJ-H (20:80 *i*-PrOH:hexane, flow rate 1.5 mL min^{-1}, 254 nm, 40 °C), t_R (*S*): 30.1 min, t_R (*R*): 44.7 min, 97.5:2.5 (*S*:*R*)

er; **^{1}H NMR** (400 MHz, $CDCl_3$) δ_H: 3.14-3.28 (2H, m, C(2)H_2), 3.87 (3H, s, OCH_3), 3.89-4.00 (1H, m, C(3)*H*), 6.94 (1H, dd, *J* 9.2, 2.6, ArC(8)*H*), 7.07 (1H, d, *J* 2.6, ArC(6)*H*), 7.42-7.50 (5H, m, PhC*H*), 8.43 (1H, d, *J* 9.2, ArC(9)*H*); **^{13}C{^{1}H} NMR** (126 MHz, $CDCl_3$) δ_C: 32.2 (*C*(2)H_2), 38.1 (q, $^2J_{CF}$ 29.2, *C*(3)HCF_3), 56.0 (OCH_3), 97.4 (*C*(4)), 106.5 (Ar*C*(6)H), 113.9 (Ar*C*(8)H), 118.6 (Ar*C*(9)H), 126.2 (q, $^1J_{CF}$ 283.0, CF_3), 127.0 (Ph*C*(3,5)H), 128.5 (*C*(6a)), 128.8 (Ph*C*(2,6)H), 129.8 (*C*(9a)), 130.4 (Ph*C*(4)H), 139.5 (Ph*C*(1)), 158.1 (Ar*C*(7)), 159.7 (*C*(5)), 165.7 (N*C*(1)=O), 191.4 (*C*=O); **^{19}F{^{1}H} NMR** (376 MHz, $CDCl_3$) δ_F: −72.9; **IR** (neat) ν_{max} cm^{-1} 2970, 1734 (C=O), 1717, 1599, 1489, 1373, 1352, 1296, 1265, 1250, 1213, 1113; **HRMS** (APCI)$^+$ calculated for $C_{20}H_{15}O_3NF_3S^+$ ([M+H]$^+$) requires 406.0719; found 406.0722 (+0.7 ppm).

(*S*)-8-Benzoyl-7-(trifluoromethyl)-6,7-dihydro-5*H*-thiazolo[3,2-*a*]pyridin-5-one **28**

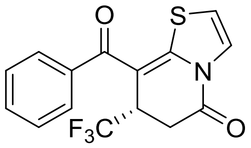

According to **General Procedure E**, 2,4,6-trichlorophenyl (*E*)-4,4,4-trifluorobut-2-enoate (70 mg, 0.22 mmol), 1-phenyl-2-(thiazol-2-yl)ethan-1-one (41 mg, 0.2 mmol), HyperBTM (3.1 mg, 0.01 mmol) and THF (0.4 mL); followed by 3,5-bis(trifluoromethyl)phenol (6 μL, 0.04 mmol) and *i*-Pr_2NEt (7 μL, 0.04 mmol) for 4 h (reflux), was purified [eluent: 0%→25% EtOAc in petrol] to give *(S)-8-benzoyl-7-(trifluoromethyl)-6,7-dihydro-5H-thiazolo[3,2-a]pyridin-5-one* **28** as a yellow solid (60 mg, 0.18 mmol, 92%).

m.p. 65-70 °C; $[\alpha]_D^{20}$ = +189 (*c* 1.0, $CHCl_3$); **Chiral HPLC analysis**: CHIRALCEL OJ-H (20:80 *i*-PrOH:hexane, flow rate 1.5 mL min^{-1}, 254 nm, 40 °C), t_R (*S*): 18.3 min, t_R (*R*): 25.3 min, 96.3:3.7 (*S*:*R*) er; **^{1}H NMR** (400 MHz, $CDCl_3$) δ_H: 3.08-3.21 (2H, m, C(6)H_2), 3.90-4.02 (1H, m, C(7)*H*), 6.52 (d, *J* 4.7, C(2)*H*), 7.39-7.49 (5H, m, PhC*H*), 7.66 (d, *J* 4.7, C(3)*H*); **^{13}C{^{1}H} NMR** (126 MHz, $CDCl_3$) δ_C: 31.0 (*C*(6)H_2), 38.0 (q, $^2J_{CF}$ 29.2, *C*(7)HCF_3), 95.6 (*C*(8)), 111.2 (*C*(2)H), 121.7 (*C*(3)H), 126.2 (q, $^1J_{CF}$ 283.2, CF_3), 127.0 (Ph*C*(3,5)H), 128.7 (Ph*C*(2,6)H), 130.2 (Ph*C*(4)H), 139.6 (Ph*C*(1)), 159.7 (*C*(9)), 164.3 (N*C*(5)=O), 189.9 (*C*=O); **^{19}F{^{1}H} NMR** (376 MHz, $CDCl_3$) δ_F: −73.4; **IR** (neat) ν_{max} cm^{-1} 2959, 2913, 1734 (C=O), 1595, 1474, 1373, 1352, 1233, 1177, 1105, 1094; **HRMS** (APCI)$^+$ calculated for $C_{15}H_{11}O_2NF_3S^+$ ([M+H]$^+$) requires 326.0457; found 326.0460 (+0.9 ppm).

(*S*)-4-Benzoyl-3-(difluoromethyl)-2,3-dihydro-1*H*-benzo[4,5]thiazolo[3,2-*a*]pyridin-1-one **29**

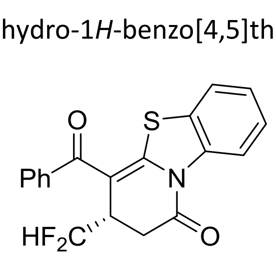

According to **General Procedure E**, 2,4,6-trichlorophenyl (*E*)-4,4-difluorobut-2-enoate (66 mg, 0.22 mmol), 2-(benzo[*d*]thiazol-2-yl)-1-phenylethan-1-one (51 mg, 0.2 mmol), HyperBTM (3.1 mg, 0.01 mmol) and THF (0.4 mL); followed by 3,5-bis(trifluoromethyl)phenol (6 μL, 0.04 mmol) and *i*-Pr_2NEt (7

μL, 0.04 mmol) for 2 h (reflux), was purified [eluent: 0%→20% EtOAc in petrol] to give *(S)-4-benzoyl-3-(difluoromethyl)-2,3-dihydro-1H-benzo[4,5]thiazolo[3,2-a]pyridin-1-one* **29** as a colourless solid (61 mg, 0.17 mmol, 85%).

m.p. 150-151 °C; $[\alpha]_D^{20}$ = +110 (*c* 0.5, $CHCl_3$); **Chiral HPLC analysis**: CHIRALPAK AD-H (10:90 *i*-PrOH:hexane, flow rate 1.5 mL min^{-1}, 254 nm, 40 °C), t_R (*R*): 22.1 min, t_R (*S*): 38.2 min, 89.9:10.1 (*S*:*R*) er; **^{1}H NMR** (400 MHz, $CDCl_3$) δ_H: 3.12 (1H, dd, *J* 17.2, 7.2, C(2)H_AH_B), 3.19 (1H, dd, *J* 17.2, 1.9, C(2)H_AH_B), 3.56-3.72 (1H, m, C(3)*H*), 5.61 (1H, app td, *J* 55.7, 2.2, C*H*F_2), 7.33 (1H, app td, *J* 7.6, 1.1, ArC(7)*H*), 7.40 (1H, app td, *J* 7.6, 1.3, ArC(8)*H*), 7.43-7.52 (5H, m, PhC*H*), 7.56 (1H, app dd, *J* 7.6, 1.0, ArC(6)*H*), 8.55 (1H, app d, *J* 8.2, ArC(9)*H*); **^{13}C{^{1}H} NMR** (126 MHz, $CDCl_3$) δ_C: 31.1 (*C*(2)H_2), 37.4 (app t, $^2J_{CF}$ 21.5, *C*(3)H), 99.4 (*C*(4)), 116.1 (dd, $^1J_{CF}$ 248.9, 247.2, *C*HF_2), 117.8 (Ar*C*(9)H), 122.0 (Ar*C*(6)H), 126.2 (Ar*C*(7)H), 126.9 (Ph*C*(3,5)H), 127.1 (*C*(6a)), 127.5 (Ar*C*(8)H), 128.9 (Ph*C*(2,6)H), 130.5 (Ph*C*(4)H), 136.1 (*C*(9a)), 139.6 (Ph*C*(1)), 159.0 (*C*(5)), 167.1 (N*C*(1)=O), 191.3 (*C*=O); **^{19}F{^{1}H} NMR** (376 MHz, $CDCl_3$) δ_F: −127.6 (d, *J* 278.5, CHF_AF_B), −119.8 (d, *J* 278.5, CHF_AF_B); **IR** (neat) ν_{max} cm^{-1} 2968, 2901, 1716 (C=O), 1599, 1472, 1456, 1356, 1300, 1277, 1215, 1182, 1173, 1144, 1117; **HRMS** $(NSI)^+$ calculated for $C_{19}H_{14}O_2NF_2S^+$ ($[M+H]^+$) requires 358.0708; found 358.0709 (+0.3 ppm).

(*S*)-3-(Difluoromethyl)-4-picolinoyl-2,3-dihydro-1*H*-benzo[4,5]thiazolo[3,2-*a*]pyridin-1-one **30**

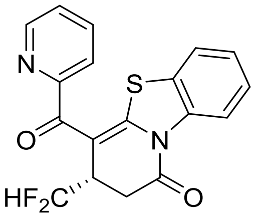

According to **General Procedure E**, 2,4,6-trichlorophenyl (*E*)-4,4-difluorobut-2-enoate (33 mg, 0.11 mmol), 2-(benzo[*d*]thiazol-2-yl)-1-(pyridin-2-yl)ethan-1-one (25 mg, 0.1 mmol), HyperBTM (1.5 mg, 0.005 mmol) and THF (0.2 mL); followed by 3,5-bis(trifluoromethyl)phenol (3 μL, 0.02 mmol) and *i*-Pr_2NEt (4 μL, 0.02 mmol) for 2 h (reflux), was purified [eluent: 0%→40% EtOAc in petrol] to give *(S)-3-(difluoromethyl)-4-picolinoyl-2,3-dihydro-1H-benzo[4,5]thiazolo[3,2-a]pyridin-1-one* **30** as a yellow solid (33 mg, 0.09 mmol, 92%).

m.p. 184-187 °C; $[\alpha]_D^{20}$ = −79.2 (*c* 0.5, $CHCl_3$); **Chiral HPLC analysis**: CHIRALPAK AD-H (15:85 *i*-PrOH:hexane, flow rate 1.5 mL min^{-1}, 254 nm, 40 °C), t_R (*R*): 8.8 min, t_R (*S*): 11.0 min, 88.4:11.6 (*S*:*R*) er; **^{1}H NMR** (400 MHz, $CDCl_3$) δ_H: 3.01 (1H, dd, *J* 17.0, 7.7, C(2)H_AH_B), 3.27 (1H, dd, *J* 17.0, 1.5, C(2)H_AH_B), 4.48-4.65 (1H, m, C(3)*H*), 6.36-6.71 (1H, m, C*H*F_2), 7.33 (1H, app td, *J* 7.5, 1.1, ArC(7)*H*), 7.37-7.44 (2H, m, ArC(8)*H* & PyC5*H*), 7.58 (1H, app dd, *J* 7.7, 1.0, ArC(6)*H*), 7.88 (1H, app td, *J* 7.8, 1.7, PyC(4)*H*), 8.08 (1H, app dt, *J* 7.9, 1.0, PyC(3)*H*), 8.55-8.63 (2H, m, ArC(9)*H* & PyC(6)*H*); **^{13}C{^{1}H} NMR** (126 MHz, $CDCl_3$) δ_C: 30.8 (*C*(2)H_2), 36.6 (dd, $^2J_{CF}$ 23.6, 19.5, *C*(3)H), 101.3 (d, $^3J_{CF}$ 10.6, *C*(4)), 117.8 (Ar*C*(9)H), 118.2 (dd, $^1J_{CF}$ 249.8, 244.1, *C*HF_2), 121.9 (Ar*C*(6)H), 124.8 (Py*C*(3)H), 125.7 (Py*C*(5)H), 126.0 (Ar*C*(7)H), 127.5 (*C*(6a)), 127.5 (Ar*C*(8)H), 136.2 (*C*(9a)), 137.5 (Py*C*(4)H), 147.7 (Py*C*(6)H), 156.1 (Py*C*(4)), 160.6 (*C*(5)), 168.1 (N*C*(1)=O), 184.7 (*C*=O); **^{19}F{^{1}H} NMR** (376 MHz, $CDCl_3$) δ_F: −130.4 (d, *J* 277.3, CHF_AF_B), −120.1 (d, *J* 277.3, CHF_AF_B); **IR** (neat) ν_{max} cm^{-1} 2974, 2920, 1719 (C=O), 1609, 1558,

1474, 1456, 1360, 1215, 1190, 1146, 1109, 1011; **HRMS** (NSI)$^+$ calculated for $C_{18}H_{13}O_2N_2F_2S^+$ ([M+H]$^+$) requires 359.0660; found 359.0662 (+0.5 ppm).

(*S*)-3-(Difluoromethyl)-4-(4-methoxybenzoyl)-2,3-dihydro-1*H*-benzo[4,5]thiazolo[3,2-*a*]pyridin-1-one **31**

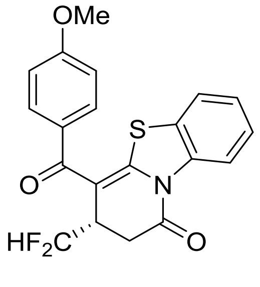

According to **General Procedure E**, 2,4,6-trichlorophenyl (*E*)-4,4-difluorobut-2-enoate (33 mg, 0.11 mmol), 2-(benzo[*d*]thiazol-2-yl)-1-(4-methoxyphenyl)ethan-1-one (28 mg, 0.1 mmol), HyperBTM (1.5 mg, 0.005 mmol) and THF (0.2 mL); followed by 3,5-bis(trifluoromethyl)phenol (3 μL, 0.02 mmol) and *i*-Pr_2NEt (4 μL, 0.02 mmol) for 7 h (reflux), was purified [eluent: 0%→20% EtOAc in petrol] to give *(S)-3-(difluoromethyl)-4-(4-methoxybenzoyl)-2,3-dihydro-1H-benzo[4,5]thiazolo[3,2-a]pyridin-1-one* **31** as a colourless solid (36 mg, 0.09 mmol, 93%).

m.p. 145-147 °C; $[\alpha]_D^{20}$ = +73.6 (*c* 0.5, $CHCl_3$); **Chiral HPLC analysis**: CHIRALPAK IC (20:80 *i*-PrOH:hexane, flow rate 1.5 mL min^{-1}, 254 nm, 40 °C), t_R (*R*): 19.1 min, t_R (*S*): 21.8 min, 91.1:8.9 (*S*:*R*) er; **^{1}H NMR** (500 MHz, $CDCl_3$) δ_H: 3.12 (1H, dd, *J* 17.2, 7.2, C(2)H_AH_B), 3.19 (1H, dd, *J* 17.2, 2.0, C(2)H_AH_B), 3.66-3.81 (1H, m, C(3)*H*), 3.86 (3H, s, OCH_3), 5.64 (1H, app td, *J* 55.8, 2.5, C*H*F_2), 6.94-6.99 (2H, m, Ar_{OMe}C(3,5)*H*), 7.30 (1H, app td, *J* 7.6, 1.2, ArC(7)*H*), 7.34-7.40 (1H, m, ArC(8)*H*), 7.44-7.45 (2H, m, Ar_{OMe}C(2,6)*H*), 7.52 (1H, app dd, *J* 7.6, 1.2, ArC(6)*H*), 8.52 (1H, app dd, *J* 8.3, 0.9, ArC(9)*H*); **^{13}C{^{1}H} NMR** (126 MHz, $CDCl_3$) δ_C: 30.9 (*C*(2)H_2), 37.5 (app t, $^2J_{CF}$ 21.5, *C*(3)H), 55.4 (O*C*H_3), 99.6 (d, $^3J_{CF}$ 6.7, *C*(4)), 114.1 (Ar_{OMe}*C*(3,5)H), 116.0 (app t, $^1J_{CF}$ 248.0, *C*HF_2), 117.7 (Ar*C*(9)H), 121.7 (Ar*C*(6)H), 126.0 (Ar*C*(7)H), 126.9 (*C*(6a)), 127.2 (Ar*C*(8)H), 129.1 (Ar_{OMe}*C*(2,6)H), 131.7 (Ar_{OMe}*C*(1)), 136.0 (*C*(9a)), 158.3 (*C*(5)), 161.4 (Ar_{OMe}*C*(4)), 167.0 (N*C*(1)=O), 190.5 (*C*=O); **^{19}F{^{1}H} NMR** (376 MHz, $CDCl_3$) δ_F: −127.7 (d, *J* 278.5, CHF_AF_B), −119.8 (d, *J* 278.5, CHF_AF_B); **IR** (neat) ν_{max} cm^{-1} 2970, 2930, 1719 (C=O), 1584, 1558, 1472, 1456, 1360, 1339, 1300, 1258, 1169, 1015; **HRMS** (NSI)$^+$ calculated for $C_{20}H_{16}O_3NF_2S^+$ ([M+H]$^+$) requires 388.0813; found 388.0814 (+0.1 ppm).

(*S*)-4-Acetyl-3-(difluoromethyl)-2,3-dihydro-1*H*-benzo[4,5]thiazolo[3,2-*a*]pyridin-1-one **32**

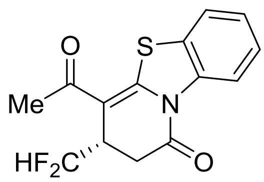

According to **General Procedure E**, 2,4,6-trichlorophenyl (*E*)-4,4-difluorobut-2-enoate (33 mg, 0.11 mmol), 1-(benzo[*d*]thiazol-2-yl)propan-2-one (19 mg, 0.1 mmol), HyperBTM (1.5 mg, 0.005 mmol) and THF (0.2 mL); followed by 3,5-bis(trifluoromethyl)phenol (3 μL, 0.02 mmol) and *i*-Pr_2NEt (4 μL, 0.02 mmol) for 6 h (reflux), was purified [eluent: 0%→20% EtOAc in petrol] to give *(S)-4-acetyl-3-(difluoromethyl)-2,3-dihydro-1H-benzo[4,5]thiazolo[3,2-a]pyridin-1-one* **32** as a yellow solid (27 mg, 0.19 mmol, 91%).

m.p. 111-115 °C; $[\alpha]_D^{20}$ = +37.8 (*c* 0.5, $CHCl_3$); **Chiral HPLC analysis**: CHIRALCEL OD-H (15:85 *i*-PrOH:hexane, flow rate 1.5 mL min^{-1}, 254 nm, 40 °C), t_R (*R*): 10.9 min, t_R (*S*): 23.1 min, 86.5:13.5 (*S*:*R*) er; **^{1}H NMR** (400 MHz, $CDCl_3$) δ_H: 2.39 (3H, s, C*H₃*), 3.07 (1H, dd, *J* 17.1, 7.4, C(2)H_AH_B), 3.17 (1H, dd, *J* 17.1, 1.7, C(2)H_AH_B), 3.42-3.57 (1H, m, C(3)*H*), 5.87 (1H, app td, *J* 55.8, 3.4, C*H*F_2), 7.30 (1H, app td, *J* 7.6, 1.3, ArC(7)*H*), 7.34-7.40 (1H, m, ArC(8)*H*), 7.52 (1H, app dd, *J* 7.6, 1.4, ArC(6)*H*), 8.47-8.52 (1H, m, ArC(9)*H*); **^{13}C{^{1}H} NMR** (126 MHz, $CDCl_3$) δ_C: 26.4 (*C*H_3), 31.7 (app t, $^3J_{CF}$ 4.0, *C*(2)H_2), 37.3 (app t, $^2J_{CF}$ 21.8, *C*(3)H), 100.1 (dd, $^3J_{CF}$ 4.9, 1.9, *C*(4)), 115.9 (app t, $^1J_{CF}$ 247.3, *C*HF_2), 117.6 (Ar*C*(9)H), 121.8 (Ar*C*(6)H), 125.9 (Ar*C*(7)H), 127.1 (Ar*C*(8)H), 127.2 (*C*(6a)), 135.6 (*C*(9a)), 156.5 (*C*(5)), 166.9 (N*C*(1)=O), 192.3 (*C*=O); **^{19}F{^{1}H} NMR** (376 MHz, $CDCl_3$) δ_F: −123.8 (d, *J* 280.7, CHF_AF_B), −120.4 (d, *J* 280.7, CHF_AF_B); **IR** (neat) ν_{max} cm^{-1} 2972, 2922, 1713 (C=O), 1630, 1506, 1456, 1373, 1298, 1271, 1252, 1217, 1171, 1053; **HRMS** (NSI)$^+$ calculated for $C_{14}H_{12}O_2NF_2S^+$ ($[M+H]^+$) requires 296.0551; found 296.0554 (+0.9 ppm).

(*S*)-4-Benzoyl-3-(perfluoroethyl)-2,3-dihydro-1*H*-benzo[4,5]thiazolo[3,2-*a*]pyridin-1-one **33**

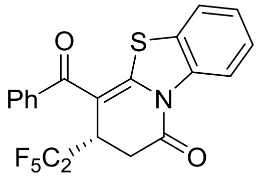

According to **General Procedure E**, 2,4,6-trichlorophenyl (*E*)-4,4,5,5,5-pentafluoropent-2-enoate (41 mg, 0.11 mmol), 2-(benzo[*d*]thiazol-2-yl)-1-phenylethan-1-one (25 mg, 0.1 mmol), HyperBTM (3.1 mg, 0.01 mmol) and THF (0.2 mL); followed by 3,4,5-trifluorophenol (4.4 mg, 0.03 mmol) and *i*-Pr_2NEt (5 μL, 0.03 mmol) for 11 h (reflux), was purified [eluent: 0%→20% EtOAc in petrol] to give *(S)-4-benzoyl-3-(perfluoroethyl)-2,3-dihydro-1H-benzo[4,5]thiazolo[3,2-a]pyridin-1-one* **33** as a pale yellow solid (38 mg, 0.09 mmol, 89%).

m.p. 176-178 °C; $[\alpha]_D^{20}$ = +202 (*c* 0.5, $CHCl_3$); **Chiral HPLC analysis**: CHIRALPAK AD-H (10:90 *i*-PrOH:hexane, flow rate 1.5 mL min^{-1}, 254 nm, 40 °C), t_R (*R*): 11.0 min, t_R (*S*): 14.3 min, 97.4:2.6 (*S*:*R*) er; **^{1}H NMR** (500 MHz, $CDCl_3$) δ_H: 3.22-3.34 (2H, m, C(2)*H₂*), 4.02-4.14 (1H, m, C(3)*H*), 7.34 (1H, app td,

J 7.6, 1.2, ArC(7)H), 7.37-7.42 (1H, m, ArC(8)H), 7.38-7.52 (5H, m, PhCH), 7.57 (1H, app dd, J 7.6, 1.2, ArC(6)H), 8.52 (1H, app d, J 8.2, ArC(9)H); **^{13}C{^{1}H} NMR** (126 MHz, $CDCl_3$) δ_C: 32.3 (C(2)H_2), 35.7 (app t, $^{2}J_{CF}$ 22.0, C(3)H), 97.0 (C(4)), 115.2 (app tq, J 259.1, 36.9, CF_2CF_3), 117.8 (ArC(9)H), 118.9 (app qt, J 286.9, 36.3, CF_3), 122.0 (ArC(6)H), 126.4 (ArC(7)H), 126.9 (C(6a)), 127.1 (PhC(3,5)H), 127.5 (ArC(8)H), 128.7 (PhC(2,6)H), 130.6 (PhC(4)H), 135.9 (C(9a)), 139.1 (PhC(1)), 159.6 (C(5)), 166.2 (NC(1)=O), 191.5 (C=O); **^{19}F{^{1}H} NMR** (376 MHz, $CDCl_3$) δ_F: : −120.7 (d, J 272.9, CF_AF_B), −117.8 (d, J 272.9, CF_AF_B), −82.2 (CF_3); **IR** (neat) ν_{max} cm^{-1} 1734 (C=O), 1616, 1489, 1354, 1304, 1200; **HRMS** (APCI)$^+$ calculated for $C_{20}H_{13}O_2NF_5S^+$ ([M+H]$^+$) requires 426.0582; found 426.0587 (+1.2 ppm).

(*S*)-5-(Benzo[*d*]oxazol-2-yl)-6-phenyl-4-(trifluoromethyl)-3,4-dihydro-2*H*-pyran-2-one **34**

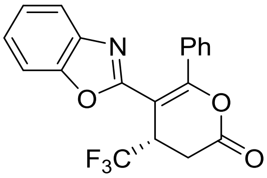

According to **General Procedure F**, 2,4,6-trichlorophenyl (*E*)-4,4,4-trifluorobut-2-enoate (96 mg, 0.3 mmol), 2-(benzo[*d*]oxazol-2-yl)-1-phenylethan-1-one (47 mg, 0.2 mmol), HyperBTM (6.2 mg, 0.02 mmol) and THF (0.4 mL) gave after purification [eluent: 0%→20% EtOAc in petrol] *(S)-5-(benzo[d]oxazol-2-yl)-6-phenyl-4-(trifluoromethyl)-3,4-dihydro-2H-pyran-2-one* **34** as a colourless oil (46 mg, 0.13 mmol, 64%).

$[\alpha]_D^{20}$ = +77.6 (c 0.5, $CHCl_3$); **Chiral HPLC analysis**: CHIRALPAK AD-H (10:90 *i*-PrOH:hexane, flow rate 1.5 mL min^{-1}, 254 nm, 40 °C), t_R (*R*): 5.6 min, t_R (*S*): 14.8 min, 99.5:0.5 (*S*:*R*) er; **^{1}H NMR** (400 MHz, $CDCl_3$) δ_H: 3.10 (1H, dd, J 17.0, 7.6, C(3)H_AH_B), 3.23 (1H, d, J 17.0, C(3)H_AH_B), 4.40 (1H, app quin., J 8.1, C(4)H), 7.24 (1H, app d, J 8.0, benzoxazoleC(7)H), 7.27-7.36 (2H, m, benzoxazoleC(5,6)H), 7.36-7.42 (2H, m, PhC(3,5)H), 7.43-7.52 (3H, m, PhC(2,4,6)H), 7.70 (1H, app d, J 7.8, benzoxazoleC(4)H); **^{13}C{^{1}H} NMR** (126 MHz, $CDCl_3$) δ_C: 28.1 (C(3)H_2), 39.7 (q, $^{2}J_{CF}$ 29.6, C(4)HCF_3), 99.2 (C(5)), 110.7 (benzoxazole$C$(7)H), 120.1 (benzoxazole$C$(4)H), 124.9 (benzoxazole$C$(6)H), 125.8 (benzoxazole$C$(5)H), 125.9 (q, $^{1}J_{CF}$ 282.2, CF_3), 128.4 (PhC(3,5)H), 129.1 (PhC(2,6)H), 131.1 (PhC(4)H), 132.0 (PhC(1)), 141.4 (benzoxazole$C$(3a)), 150.4 (benzoxazole$C$(7a)), 159.0 (C(6)), 160.2 (benzoxazole$C$(2)), 164.0 (C(2)=O); **^{19}F NMR** (376 MHz, $CDCl_3$) δ_F: −71.7; **IR** (neat) ν_{max} cm^{-1} 1790 (C=O), 1654, 1452, 1360, 1273, 1246, 1227, 1171, 1119; **HRMS** (NSI)$^+$ calculated for $C_{19}H_{16}O_3N_2F_3^+$ ([M+NH$_4$]$^+$) requires 377.1108; found 377.1113 (+1.4 ppm).

(S)-5-(Benzo[d]oxazol-2-yl)-6-(pyridin-3-yl)-4-(trifluoromethyl)-3,4-dihydro-2H-pyran-2-one **35**

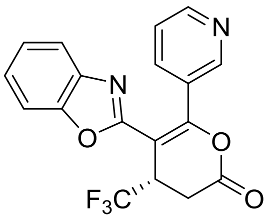

According to **General Procedure F**, 2,4,6-trichlorophenyl (*E*)-4,4,4-trifluorobut-2-enoate (96 mg, 0.3 mmol), 2-(benzo[*d*]oxazol-2-yl)-1-(pyridin-3-yl)ethan-1-one (48 mg, 0.2 mmol), HyperBTM (6.2 mg, 0.02 mmol) and THF (0.4 mL) gave after purification [eluent: 0%→60% EtOAc in petrol] *(S)-5-(benzo[d]oxazol-2-yl)-6-(pyridin-3-yl)-4-(trifluoromethyl)-3,4-dihydro-2H-pyran-2-one* **35** as a yellow oil (46 mg, 0.13 mmol, 64%).

$[\alpha]_D^{20}$ = +46.6 (*c* 0.5, $CHCl_3$); **Chiral HPLC analysis**: CHIRALPAK AD-H (15:85 *i*-PrOH:hexane, flow rate 1.5 mL min^{-1}, 254 nm, 40 °C), t_R (*R*): 9.0 min, t_R (*S*): 23.3 min, 99.9:0.1 (*S*:*R*) er; **^{1}H NMR** (400 MHz, $CDCl_3$) δ_H: 3.12 (1H, dd, *J* 17.1, 7.3, C(3)H_AH_B), 3.27 (1H, dd, *J* 17.1, 1.3, C(3)H_AH_B), 4.41-4.52 (1H, m, C(4)*H*), 7.23-7.27 (1H, m, benzoxazoleC(7)*H*), 7.28-7.37 (2H, m, benzoxazoleC(5,6)*H*), 7.41 (ddd, *J* 8.0, 4.9, 0.8, PyC(4)*H*), 7.67-7.71 (1H, app d, *J* 7.8, benzoxazoleC(4)*H*), 7.86 (ddd, *J* 8.0, 2.2, 1.8, PyC(5)*H*), 8.67 (dd, *J* 2.2, 0.8, PyC(2)*H*), 8.73 (dd, *J* 4.9, 1.8, PyC(6)*H*); **^{13}C{^{1}H} NMR** (126 MHz, $CDCl_3$) δ_C: 27.9 (*C*(3)H_2), 39.3 (q, $^2J_{CF}$ 29.8, *C*(4)HCF_3), 100.8 (*C*(5)), 110.7 (benzoxazole*C*(7)H), 120.1 (benzoxazole*C*(4)H), 123.1 (Py*C*(5)H), 125.1 (benzoxazole*C*(6)H), 125.6 (q, $^1J_{CF}$ 282.4, *C*F_3), 126.1 (benzoxazole*C*(5)H), 128.3 (Py*C*(3)), 136.4 (Py*C*(4)H), 141.1 (benzoxazole*C*(3a)), 149.7 (Py*C*(2)H), 150.2 (benzoxazole*C*(7a)), 151.6 (Py*C*(6)H), 156.0 (*C*(6)), 159.1 (benzoxazole*C*(2)), 163.3 (*C*(2)=O); **^{19}F{^{1}H} NMR** (376 MHz, $CDCl_3$) δ_F: −71.5; **IR** (neat) ν_{max} cm^{-1} 2963, 2926, 1792 (C=O), 1653, 1452, 1362, 1227, 1171, 1115; **HRMS** (ESI)$^+$ calculated for $C_{18}H_{11}O_3N_2F_3^+$ ($[M+H]^+$) requires 361.0795; found 361.0787 (−2.1 ppm).

(S)-5-(Benzo[d]oxazol-2-yl)-6-(thiophen-3-yl)-4-(trifluoromethyl)-3,4-dihydro-2H-pyran-2-one **36**

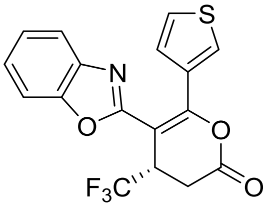

According to **General Procedure F**, 2,4,6-trichlorophenyl (*E*)-4,4,4-trifluorobut-2-enoate (96 mg, 0.3 mmol), 2-(benzo[*d*]oxazol-2-yl)-1-(thiophen-3-yl)ethan-1-one (49 mg, 0.2 mmol), HyperBTM (6.2 mg, 0.02 mmol) and THF (0.4 mL) gave after purification [eluent: 0%→20% EtOAc in petrol] *(S)-5-(benzo[d]oxazol-2-yl)-6-(thiophen-3-yl)-4-(trifluoromethyl)-3,4-dihydro-2H-pyran-2-one* **36** as a yellow oil (56 mg, 0.15 mmol, 77%).

$[\alpha]_D^{20}$ = +48.6 (*c* 1.0, $CHCl_3$); **Chiral HPLC analysis**: CHIRALPAK AD-H (5:95 *i*-PrOH:hexane, flow rate 1.5 mL min^{-1}, 254 nm, 40 °C), t_R (*R*): 10.0 min, t_R (*S*): 19.2 min, 99.4:0.6 (*S*:*R*) er; **^{1}H NMR** (400 MHz, $CDCl_3$) δ_H: 3.06 (1H, dd, *J* 16.9, 7.5, C(3)H_AH_B), 3.21 (1H, dd, *J* 16.9, 1.2, C(3)H_AH_B), 4.26-4.38 (1H, m, C(4)*H*),

6.99 (dd, *J* 5.1, 1.3, ThC(4)*H*), 7.28 (dd, *J* 5.1, 3.0, ThC(5)*H*), 7.33-7.43 (3H, m, benzoxazoleC(5,6,7)*H*), 7.71-7.76 (1H, m, benzoxazoleC(4)*H*), 7.78 (dd, *J* 3.0, 1.3, ThC(2)*H*); **^{13}C{^{1}H} NMR** (126 MHz, $CDCl_3$) δ_C: 28.0 (*C*(3)H_2), 40.0 (q, $^2J_{CF}$ 29.6, *C*(4)HCF_3), 98.0 (*C*(5)), 110.9 (benzoxazole*C*(7)H), 120.2 (benzoxazole*C*(4)H), 125.1 (benzoxazole*C*(6)H), 125.8 (q, $^1J_{CF}$ 282.4, *C*F_3), 125.8 (Th*C*(5)H), 126.0 (benzoxazole*C*(5)H), 127.4 (Th*C*(4)H), 129.7 (Th*C*(2)H), 132.2 (Th*C*(3)), 141.4 (benzoxazole*C*(3a)), 150.4 (benzoxazole*C*(7a)), 153.7 (*C*(6)), 160.1 (benzoxazole*C*(2)), 164.0 (*C*(2)=O); **^{19}F{^{1}H} NMR** (376 MHz, $CDCl_3$) δ_F: −71.7; **IR** (neat) ν_{max} cm^{-1} 3113, 2930, 1790 (C=O), 1647, 1452, 1358, 1269, 1229, 1171, 1119; **HRMS** (NSI)$^+$ calculated for $C_{17}H_{11}O_3NF_3S^+$ ([M+H]$^+$) requires 366.0406; found 366.0408 (+0.5 ppm).

(*S*)-5-(Benzo[*d*]oxazol-2-yl)-4-(trifluoromethyl)-6-(4-(trifluoromethyl)phenyl)-3,4-dihydro-2*H*-pyran-2-one **37**

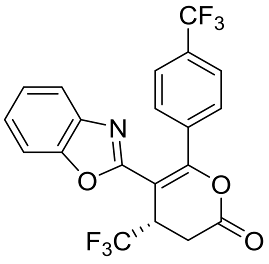

According to **General Procedure F**, 2,4,6-trichlorophenyl (*E*)-4,4,4-trifluorobut-2-enoate (96 mg, 0.3 mmol), 2-(benzo[*d*]oxazol-2-yl)-1-(4-(trifluoromethyl)phenyl)ethan-1-one (61 mg, 0.2 mmol), HyperBTM (6.2 mg, 0.02 mmol) and THF (0.4 mL) gave after purification [eluent: 0%→20% EtOAc in petrol] *(S)-5-(benzo[d]oxazol-2-yl)-4-(trifluoromethyl)-6-(4-(trifluoromethyl)phenyl)-3,4-dihydro-2H-pyran-2-one* **37** and *(S)-3-(trifluoromethyl)-4-(4-(trifluoromethyl)benzoyl)-2,3-dihydro-1H-benzo[4,5]oxazolo[3,2-a]pyridin-1-one* **42** (90:10 inseparable mixture) as a colourless solid (80 mg, 0.19 mmol, 94%).

m.p. 46-47 °C; $[\alpha]_D^{20}$ = +70.6 (*c* 0.5, $CHCl_3$); **Chiral HPLC analysis (37)**: CHIRALPAK AD-H (5:95 *i*-PrOH:hexane, flow rate 1.5 mL min^{-1}, 254 nm, 40 °C), t_R (*R*): 5.7 min, t_R (*S*): 7.3 min, 99.8:0.2 (*S*:*R*) er; **^{1}H NMR** (400 MHz, $CDCl_3$) δ_H: 3.12 (1H, dd, *J* 17.1, 7.5, C(3)H_AH_B), 3.26 (1H, dd, *J* 17.1, 1.1, C(3)H_AH_B), 4.38-4.49 (1H, m, C(4)*H*), 7.23-7.28 (1H, m, benzoxazoleC(7)*H*), 7.29-7.38 (2H, m, benzoxazoleC(5,6)*H*), 7.56-7.63 (2H, m, ArC(2,6)*H*), 7.64-7.74 (3H, m, ArC(3,5)*H* & benzoxazoleC(4)*H*); **^{13}C{^{1}H} NMR** (126 MHz, $CDCl_3$) δ_C: 27.8 (*C*(3)H_2), 39.4 (q, $^2J_{CF}$ 29.8, *C*(4)HCF_3), 100.5 (*C*(5)), 110.7 (benzoxazole*C*(7)H), 120.1 (benzoxazole*C*(4)H), 123.6 (q, $^1J_{CF}$ 272.5, Ar*C*F_3), 125.1 (benzoxazole*C*(6)H), 125.3 (q, $^3J_{CF}$ 3.3, Ar*C*(3,5)H), 125.6 (q, $^1J_{CF}$ 282.1, C(4)*C*F_3), 126.1 (benzoxazole*C*(5)H), 129.5 (Ar*C*(2,6)H), 132.7 (q, $^2J_{CF}$ 33.1, Ar*C*(4)), 135.4 (Ar*C*(1)), 141.1 (benzoxazole*C*(3a)), 150.2 (benzoxazole*C*(7a)), 157.2 (*C*(6)), 159.2 (benzoxazole*C*(2)), 163.3 (*C*(2)=O); **^{19}F{^{1}H} NMR** (376 MHz, $CDCl_3$) δ_F: −71.6 (C(4)C*F*$_3$), −63.0 (ArC*F*$_3$); **IR** (neat) ν_{max} cm^{-1} 1792 (C=O), 1653, 1618, 1452, 1410, 1360, 1321, 1225, 1167, 1105, 1069; **HRMS** (NSI)$^+$ calculated for $C_{20}H_{12}O_3NF_6^+$ ([M+H]$^+$) requires 428.0716; found 428.0719 (+0.7 ppm).

Chiral HPLC analysis for minor dihydropyranone product (42): CHIRALPAK AD-H (5:95 *i*-PrOH:hexane, flow rate 1.5 mL min^{-1}, 254 nm, 40 °C), t_R (*S*): 8.2 min, t_R (*R*): 9.6 min, 92.3:7.8 (*S*:*R*) er.

(*S*)-4-(5-(Benzo[*d*]oxazol-2-yl)-2-oxo-4-(trifluoromethyl)-3,4-dihydro-2*H*-pyran-6-yl)benzonitrile **38**

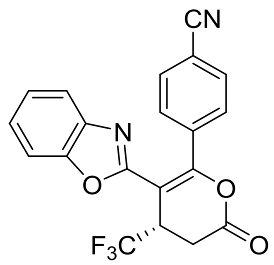

According to **General Procedure F**, 2,4,6-trichlorophenyl (*E*)-4,4,4-trifluorobut-2-enoate (96 mg, 0.3 mmol), 4-(2-(benzo[*d*]oxazol-2-yl)acetyl)benzonitrile (53 mg, 0.2 mmol), HyperBTM (6.2 mg, 0.02 mmol) and THF (0.4 mL) gave after purification [eluent: 0%→20% EtOAc in petrol] *(S)-4-(5-(benzo[d]oxazol-2-yl)-2-oxo-4-(trifluoromethyl)-3,4-dihydro-2H-pyran-6-yl)benzonitrile* **38** and *(S)-4-(1-oxo-3-(trifluoromethyl)-2,3-dihydro-1H-benzo[4,5]oxazolo[3,2-a]pyridine-4-carbonyl)benzonitrile* **43** (89:11 inseparable mixture) as a yellow oil (55 mg, 0.19 mmol, 94%).

$[\alpha]_D^{20}$ = +59.4 (*c* 0.5, $CHCl_3$); **Chiral HPLC analysis (38)**: CHIRALPAK IC (20:80 *i*-PrOH:hexane, flow rate 1.5 mL min^{-1}, 254 nm, 40 °C), t_R (*S*): 5.4 min, t_R (*R*): 6.3 min, 99.8:0.2 (*S*:*R*) er; **^{1}H NMR** (400 MHz, $CDCl_3$) δ_H: 3.11 (1H, dd, *J* 17.1, 7.6, C(3)H_AH_B), 3.26 (1H, dd, *J* 17.1, 1.2, C(3)H_AH_B), 4.38-4.49 (1H, m, C(4)*H*), 7.24-7.29 (1H, m, benzoxazoleC(7)*H*), 7.31-7.39 (2H, m, benzoxazoleC(5,6)*H*), 7.56-7.61 (2H, m, ArC(2,6)*H*), 7.68-7.73 (3H, m, ArC(3,5)*H* & benzoxazoleC(4)*H*); **^{13}C{^{1}H} NMR** (126 MHz, $CDCl_3$) δ_C: 27.9 (*C*(3)H_2), 39.6 (q, $^2J_{CF}$ 29.9, *C*(4)HCF_3), 101.1 (*C*(5)), 110.7 (benzoxazole*C*(7)H), 114.7 (Ar*C*(4)), 118.2 (*C*N), 120.4 (benzoxazole*C*(4)H), 125.3 (benzoxazole*C*(6)H), 125.7 (q, $^1J_{CF}$ 282.1, C(4)*C*F_3), 126.4 (benzoxazole*C*(5)H), 129.9 (Ar*C*(2,6)H), 132.1 (Ar*C*(3,5)H), 136.3 (Ar*C*(1)), 141.2 (benzoxazole*C*(3a)), 150.3 (benzoxazole*C*(7a)), 156.6 (*C*(6)), 159.0 (benzoxazole*C*(2)), 163.1 (*C*(2)=O); **^{19}F{^{1}H} NMR** (376 MHz, $CDCl_3$) δ_F: −71.5; **IR** (neat) ν_{max} cm^{-1} 2232 (CN), 1796 (C=O), 1653, 1609, 1360, 1225, 117, 1121; **HRMS** (NSI)$^+$ calculated for $C_{20}H_{12}O_3N_2F_3^+$ ([M+H]$^+$) requires 385.0795; found 385.0797 (+0.6 ppm).

Chiral HPLC analysis (for minor dihydropyranone product (43): CHIRALPAK IC (20:80 *i*-PrOH:hexane, flow rate 1.5 mL min^{-1}, 254 nm, 40 °C), t_R (*S*): 9.2 min, t_R (*R*): 11.0 min, 92.8:7.2 (*S*:*R*).

(*S*)-4-Benzoyl-3-(trifluoromethyl)-2,3-dihydro-1*H*-benzo[4,5]oxazolo[3,2-*a*]pyridin-1-one **39**

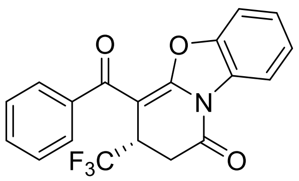

According to **General Procedure G**, 2,4,6-trichlorophenyl (*E*)-4,4,4-trifluorobut-2-enoate (70 mg, 0.22 mmol), 2-(benzo[*d*]oxazol-2-yl)-1-phenylethan-1-one (47 mg, 0.2 mmol), HyperBTM (3.1 mg, 0.01 mmol) and THF (0.4 mL); followed by 3,4,5-trifluorophenol (9 mg, 0.06 mmol) and *i*-Pr_2NEt (11 μL, 0.06 mmol), gave after purification [eluent: 0%→20% EtOAc in petrol] *(S)-4-benzoyl-3-(trifluoromethyl)-2,3-dihydro-1H-benzo[4,5]oxazolo[3,2-a]pyridin-1-one* **39** as a colourless solid (51 mg, 0.14 mmol, 71%).

m.p. 177-180 °C; $[\alpha]_D^{20}$ = +181 (*c* 0.5, $CHCl_3$); **Chiral HPLC analysis**: CHIRALPAK AD-H (10:90 *i*-PrOH:hexane, flow rate 1.5 mL min^{-1}, 254 nm, 40 °C), t_R (*S*): 7.3 min, t_R (*R*): 11.2 min, 97.8:2.2 (*S*:*R*) er; **^{1}H NMR** (400 MHz, $CDCl_3$) δ_H: 3.03 (1H, dd, *J* 17.9, 8.1, C(2)H_AH_B), 3.16 (1H, dd, *J* 17.9, 1.2, C(2)H_AH_B), 4.30-4.44 (1H, m, C(3)*H*), 6.89-6.95 (1H, m, ArC(6)*H*), 7.17 (1H, app td, *J* 8.0, 1.6, ArC(7)*H*), 7.22 (1H, app td, *J* 7.7, 1.2, ArC(8)*H*), 7.42-7.49 (2H, m, PhC(3,5)*H*), 7.52-7.58 (1H, m, PhC(4)*H*), 7.64-7.70 (2H, m, PhC(2,6)*H*), 7.98 (1H, app dd, *J* 7.8, 1.4, ArC(9)*H*); **^{13}C{^{1}H} NMR** (126 MHz, $CDCl_3$) δ_C: 31.0 (*C*(2)H_2), 35.5 (q, $^2J_{CF}$ 29.4, *C*(3)HCF_3), 85.2 (*C*(4)), 110.0 (Ar*C*(6)H), 115.0 (Ar*C*(9)H), 125.2 (Ar*C*(7)H), 125.7 (Ar*C*(8)H), 126.6 (*C*(9a)), 126.7 (q, $^1J_{CF}$ 282.4, CF_3), 128.1 (Ph*C*(3,5)H), 128.1 (Ph*C*(2,6)H), 131.6 (Ph*C*(4)H), 140.1 (Ph*C*(1)), 146.3 (*C*(6a)), 156.5 (*C*(5)), 165.2 (N*C*(1)=O), 190.8 (*C*=O); **^{19}F{^{1}H} NMR** (376 MHz, $CDCl_3$) δ_F: −73.6; **IR** (neat) ν_{max} cm^{-1} 2924, 1701 (C=O), 1684 (C=O), 1616, 1558, 1476, 1383, 1356, 1229, 1173, 1130, 1105, 1011; **HRMS** $(NSI)^+$ calculated for $C_{19}H_{13}O_3NF_3^+$ ($[M+H]^+$) requires 360.0845; found 360.0842 (+0.8 ppm).

(*S*)-4-Nicotinoyl-3-(trifluoromethyl)-2,3-dihydro-1*H*-benzo[4,5]oxazolo[3,2-*a*]pyridin-1-one **40**

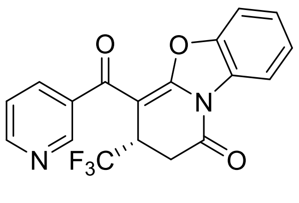

According to **General Procedure G**, 2,4,6-trichlorophenyl (*E*)-4,4,4-trifluorobut-2-enoate (35 mg, 0.11 mmol), 2-(benzo[*d*]oxazol-2-yl)-1-(pyridin-3-yl)ethan-1-one (24 mg, 0.1 mmol), HyperBTM (1.5 mg, 0.005 mmol) and THF (0.2 mL); followed by 3,4,5-trifluorophenol (4 mg, 0.03 mmol) and *i*-Pr_2NEt (5 µL, 0.03 mmol), gave after purification [eluent: 0%→60% EtOAc in petrol] *(S)-4-nicotinoyl-3-(trifluoromethyl)-2,3-dihydro-1H-benzo[4,5]oxazolo[3,2-a]pyridin-1-one* **40** as a pale yellow solid (31 mg, 0.09 mmol, 86%).

m.p. 184-185 °C; $[\alpha]_D^{20}$ = +150 (*c* 0.5, $CHCl_3$); **Chiral HPLC analysis**: CHIRALPAK AD-H (15:85 *i*-PrOH:hexane, flow rate 1.5 mL min^{-1}, 211 nm, 40 °C), t_R (*S*): 11.5 min, t_R (*R*): 15.7 min, 99.7:0.3 (*S*:*R*) er; **^{1}H NMR** (400 MHz, $CDCl_3$) δ_H: 3.04 (1H, dd, *J* 17.9, 8.0, C(2)H_AH_B), 3.20 (1H, dd, *J* 17.9, 1.3, C(2)H_AH_B), 4.30-4.43 (1H, m, C(3)*H*), 6.93-6.97 (1H, m, ArC(6)*H*), 7.20 (1H, app td, *J* 7.9, 1.5, ArC(7)*H*), 7.25 (1H, app td, *J* 7.7, 1.1, ArC(8)*H*), 7.42 (1H, ddd, *J* 7.8, 4.9, 0.5, PyC(5)*H*), 7.95 (1H, app dt, *J* 7.8, 1.9, PyC(4)*H*), 8.00 (1H, app dd, *J* 7.7, 1.4, ArC(9)*H*), 8.78 (1H, app d, *J* 4.9, PyC(6)*H*), 8.86 (1H, app s, PyC(2)*H*); **^{13}C{^{1}H} NMR** (126 MHz, $CDCl_3$) δ_C: 31.1 (*C*(2)H_2), 35.4 (q, $^2J_{CF}$ 29.4, *C*(3)HCF_3), 85.6 (*C*(4)), 110.3 (Ar*C*(6)H), 115.3 (Ar*C*(9)H), 123.2 (Py*C*(5)H), 125.7 (Ar*C*(7)H), 126.1 (Ar*C*(8)H), 126.5 (*C*(9a)), 126.7 (q, $^1J_{CF}$ 282.2, CF_3), 135.6 (Py*C*(4)H), 136.1 (Py*C*(3)), 146.2 (*C*(6a)), 149.0 (Py*C*(2)H), 152.1 (Py*C*(6)H), 157.5 (*C*(5)), 165.2 (N*C*(1)=O), 188.4 (*C*=O); **^{19}F{^{1}H} NMR** (376 MHz, $CDCl_3$) δ_F: −73.5; **IR** (neat) ν_{max} cm^{-1} 3034, 2963, 1732 (C=O), 1674 (C=O), 1603, 1479, 1362, 1223, 1167, 1150, 1138, 1107; **HRMS** $(NSI)^+$ calculated for $C_{18}H_{12}O_3N_2F_3^+$ ($[M+H]^+$) requires 361.0795; found 361.0790 (−1.3 ppm).

(*S*)-4-(Thiophene-3-carbonyl)-3-(trifluoromethyl)-2,3-dihydro-1*H*-benzo[4,5]oxazolo[3,2-*a*]pyridin-1-one **41**

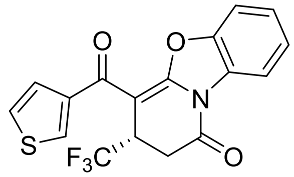

According to **General Procedure G**, 2,4,6-trichlorophenyl (*E*)-4,4,4-trifluorobut-2-enoate (70 mg, 0.22 mmol), 2-(benzo[*d*]oxazol-2-yl)-1-(thiophen-3-yl)ethan-1-one (49 mg, 0.2 mmol), HyperBTM (3.1 mg, 0.01 mmol) and THF (0.4 mL); followed by 3,4,5-trifluorophenol (9 mg, 0.06 mmol) and *i*-Pr_2NEt (11 µL, 0.06 mmol), gave after purification [eluent: 0%→20% EtOAc in petrol] *(S)-4-(thiophene-3-carbonyl)-3-(trifluoromethyl)-2,3-dihydro-1H-benzo[4,5]oxazolo[3,2-a]pyridin-1-one* **41** as a colourless solid (59 mg, 0.16 mmol, 81%).

m.p. 158-160 °C; $[\alpha]_D^{20}$ = +165 (*c* 0.5, $CHCl_3$); **Chiral HPLC analysis**: CHIRALPAK AD-H (5:95 *i*-PrOH:hexane, flow rate 1.5 mL min^{-1}, 254 nm, 40 °C), t_R (*S*): 14.6 min, t_R (*R*): 16.5 min, 98.1:1.9 (*S*:*R*) er; **^{1}H NMR** (400 MHz, $CDCl_3$) δ_H: 3.01 (1H, dd, *J* 17.8, 8.1, C(2)H_AH_B), 3.14 (1H, dd, *J* 17.8, 1.3, C(2)H_AH_B), 4.28-4.40 (1H, m, C(3)*H*), 7.02-7.08 (1H, m, ArC(6)*H*), 7.17-7.28 (2H, m, ArC(7,8)*H*), 7.33 (1H, dd, *J* 5.0, 2.9, ThC(5)*H*), 7.45 (1H, dd, *J* 5.0, 1.3, ThC(4)*H*), 7.92 (1H, dd, *J* 2.9, 1.3, ThC(2)*H*), 7.97-8.02 (1H, m, ArC(9)*H*); **^{13}C{^{1}H} NMR** (126 MHz, $CDCl_3$) δ_C: 31.0 (*C*(2)H_2), 35.7 (q, $^2J_{CF}$ 29.4, *C*(3)HCF_3), 85.9 (*C*(4)), 110.1 (Ar*C*(6)H), 115.2 (Ar*C*(9)H), 125.4 (Ar*C*(7)H), 125.4 (Th*C*(5)H), 125.9 (Ar*C*(8)H), 126.7 (*C*(9a)), 126.7 (q, $^1J_{CF}$ 282.2, *C*F_3), 127.8 (Th*C*(4)H), 130.9 (Th*C*(2)H), 142.2 (Th*C*(3)), 146.4 (*C*(6a)), 155.9 (*C*(5)), 165.3 (N*C*(1)=O), 184.2 (*C*=O); **^{19}F{^{1}H} NMR** (376 MHz, $CDCl_3$) δ_F: −73.6; **IR** (neat) ν_{max} cm^{-1} 2988, 1705 (C=O), 1682 (C=O), 1607, 1477, 1396, 1383, 1352, 1261, 1250, 1231, 1171, 1128, 1105, 1013; **HRMS** (NSI)$^+$ calculated for $C_{17}H_{11}O_3NF_3S^+$ ($[M+H]^+$) requires 366.0406; found 366.0408 (+0.5 ppm).

(*S*)-3-(Trifluoromethyl)-4-(4-(trifluoromethyl)benzoyl)-2,3-dihydro-1*H*-benzo[4,5]oxazolo[3,2-*a*]pyridin-1-one **42**

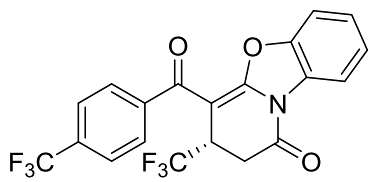

According to **General Procedure G**, 2,4,6-trichlorophenyl (*E*)-4,4,4-trifluorobut-2-enoate (70 mg, 0.22 mmol), 2-(benzo[*d*]oxazol-2-yl)-1-(4-(trifluoromethyl)phenyl)ethan-1-one (61 mg, 0.2 mmol), HyperBTM (3.1 mg, 0.01 mmol) and THF (0.4 mL); followed by 3,4,5-trifluorophenol (9 mg, 0.06 mmol) and *i*-Pr_2NEt (11 µL, 0.06 mmol), gave after purification [eluent: 0%→20% EtOAc in petrol] *(S)-3-(trifluoromethyl)-4-(4-(trifluoromethyl)benzoyl)-2,3-dihydro-1H-benzo[4,5]oxazolo[3,2-a]pyridin-1-one* **42** as a colourless solid (62 mg, 0.15 mmol, 73%).

m.p. 192-194 °C; $[\alpha]_D^{20}$ = +126 (*c* 0.5, $CHCl_3$); **Chiral HPLC analysis**: CHIRALPAK AD-H (5:95 *i*-PrOH:hexane, flow rate 1.5 mL min^{-1}, 211 nm, 40 °C), t_R (*S*): 8.2 min, t_R (*R*): 9.6 min, 97.8:2.2 (*S*:*R*) er; **^{1}H NMR** (400 MHz, $CDCl_3$) δ_H: 3.04 (1H, dd, *J* 17.8, 8.0, C(2)H_AH_B), 3.20 (1H, dd, *J* 17.8, 1.2, C(2)H_AH_B), 4.29-4.42 (1H, m, C(3)*H*), 6.90-6.95 (1H, m, ArC(6)*H*), 7.20 (1H, app td, *J* 8.0, 1.6, ArC(7)*H*), 7.26 (1H, app td, *J* 7.7, 1.3, ArC(8)*H*), 7.69-7.77 (4H, m, Ar_{CF3}C(2,3,5,6)*H*), 7.97-8.03 (1H, m, ArC(9)*H*); **^{13}C{^{1}H} NMR** (126 MHz, $CDCl_3$) δ_C: 31.0 (*C*(2)H_2), 35.3 (q, $^2J_{CF}$ 29.6, *C*(3)HCF_3), 85.3 (*C*(4)), 110.1 (Ar*C*(6)H), 115.2 (Ar*C*(9)H), 123.9 (q, $^1J_{CF}$ 272.7, Ar*C*F_3), 125.2 (q, $^3J_{CF}$ 3.7, Ar_{CF3}*C*(3,5)H), 125.6 (Ar*C*(7)H), 126.0 (Ar*C*(8)H), 126.4 (*C*(9a)), 126.7 (q, $^1J_{CF}$ 282.3, C(3)*C*F_3), 128.1 (Ar_{CF3}*C*(2,6)H), 132.9 (q, $^2J_{CF}$ 32.6, Ar_{CF3}*C*(4)), 143.7 (Ar_{CF3}*C*(1)), 146.2 (*C*(6a)), 157.6 (*C*(5)), 165.2 (N*C*(1)=O), 189.6 (*C*=O); **^{19}F{^{1}H} NMR** (376 MHz, $CDCl_3$) δ_F: −73.6 (C(3)C*F_3*), −62.8 (ArC*F_3*); **IR** (neat) ν_{max} cm^{-1} 1730 (C=O), 1671 (C=O), 1601, 1476, 1375, 1362, 1323, 1254, 1223, 1126, 1103, 1065; **HRMS** $(NSI)^+$ calculated for $C_{20}H_{12}O_3NF_6^+$ ($[M+H]^+$) requires 428.0716; found 428.0714 (−0.4 ppm).

(*S*)-4-(1-Oxo-3-(trifluoromethyl)-2,3-dihydro-1*H*-benzo[4,5]oxazolo[3,2-*a*]pyridine-4-carbonyl)benzonitrile **43**

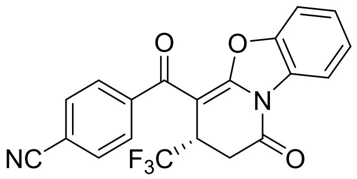

According to **General Procedure G**, 2,4,6-trichlorophenyl (*E*)-4,4,4-trifluorobut-2-enoate (70 mg, 0.22 mmol), 4-(2-(benzo[*d*]oxazol-2-yl)acetyl)benzonitrile (53 mg, 0.2 mmol), HyperBTM (3.1 mg, 0.01 mmol) and THF (0.4 mL); followed by 3,4,5-trifluorophenol (9 mg, 0.06 mmol) and *i*-Pr_2NEt (11 μL, 0.06 mmol), gave after purification [eluent: 0%→20% EtOAc in petrol] *(S)-4-(1-oxo-3-(trifluoromethyl)-2,3-dihydro-1H-benzo[4,5]oxazolo[3,2-a]pyridine-4-carbonyl)benzonitrile* **43** as a pale yellow solid (65 mg, 0.17 mmol, 85%).

m.p. 137-142 °C; $[\alpha]_D^{20}$ = +147 (*c* 1.0, $CHCl_3$); **Chiral HPLC analysis**: CHIRALPAK IC (20:80 *i*-PrOH:hexane, flow rate 1.5 mL min^{-1}, 211 nm, 40 °C), t_R (*S*): 9.2 min, t_R (*R*): 11.0 min, 97.5:2.5 (*S*:*R*) er; **^{1}H NMR** (400 MHz, $CDCl_3$) δ_H: 3.03 (1H, dd, *J* 17.9, 8.1, C(2)H_AH_B), 3.20 (1H, dd, *J* 17.9, 1.2, C(2)H_AH_B), 4.28-4.41 (1H, m, C(3)*H*), 6.92-6.97 (1H, m, ArC(6)*H*), 7.22 (1H, app td, *J* 8.0, 1.6, ArC(7)*H*), 7.27 (1H, app td, *J* 7.7, 1.3, ArC(8)*H*), 7.68-7.73 (2H, m, Ar_{CN}C(2,6)*H*), 7.74-7.79 (2H, m, Ar_{CN}C(3,5)*H*), 7.97-8.02 (1H, m, ArC(9)*H*); **^{13}C{^{1}H} NMR** (126 MHz, $CDCl_3$) δ_C: 30.9 (*C*(2)H_2), 35.2 (q, $^2J_{CF}$ 29.6, *C*(3)HCF_3), 85.2 (*C*(4)), 110.1 (Ar*C*(6)H), 114.5 (Ar_{CN}*C*(4)), 115.2 (Ar*C*(9)H), 118.4 (*C*N), 125.7 (Ar*C*(7)H), 126.1 (Ar*C*(8)H), 126.3 (*C*(9a)), 126.6 (q, $^1J_{CF}$ 281.9, C(3)*C*F_3), 128.3 (Ar_{CN}*C*(2,6)H), 132.0 (Ar_{CN}*C*(3,5)H), 144.4 (Ar_{CN}*C*(1)), 146.0 (*C*(6a)), 157.6 (*C*(5)), 164.9 (N*C*(1)=O), 188.7 (*C*=O); **^{19}F{^{1}H} NMR** (376 MHz, $CDCl_3$) δ_F: −73.5; **IR** (neat) ν_{max} cm^{-1} 2230 (CN), 1734 (C=O), 1663 (C=O), 1597, 1476, 1373, 1254, 1225, 1169, 1011; **HRMS** $(NSI)^+$ calculated for $C_{20}H_{12}O_3N_2F_3^+$ ($[M+H]^+$) requires 385.0795; found 385.0795 (+0.1 ppm).

(*R*)-4-Benzoyl-3-phenyl-2,3-dihydro-1*H*-benzo[4,5]thiazolo[3,2-*a*]pyridin-1-one **44**

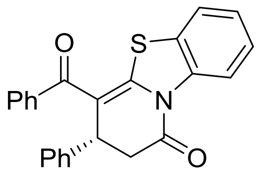

According to **General Procedure H**, (*E*)-cinnamic anhydride (61 mg, 0.22 mmol), 2-(benzo[*d*]thiazol-2-yl)-1-phenylethan-1-one (51 mg, 0.2 mmol), *i*-Pr_2NEt (45 μL, 0.26 mmol), HyperBTM (3.1 mg, 0.01 mmol) and THF (0.4 mL); followed by 3,5-bis(trifluoromethyl)phenol (12 μL, 0.08 mmol) gave after purification [eluent: 40%→80% CH_2Cl_2 in petrol] (*R*)-4-benzoyl-3-phenyl-2,3-dihydro-1*H*-benzo[4,5]thiazolo[3,2-*a*]pyridin-1-one **44** as a yellow solid (73 mg, 0.19 mmol, 95%).

m.p. 137-140 °C {Lit: 168-171 °C}; $[\alpha]_D^{20}$ = −112 (*c* 1.0, $CHCl_3$) {Lit: −148.5 (*c* 1.0 in $CHCl_3$, 98:2 er)}; **Chiral HPLC analysis**: CHIRALPAK AD-H (20:80 *i*-PrOH:hexane, flow rate 1.0 mL min^{-1}, 211 nm, 30 °C), t_R (*S*): 13.2 min, t_R (*R*): 22.4 min, 92.3:7.7 (*R*:*S*) er; **^{1}H NMR** (400 MHz, $CDCl_3$) δ_H: 3.04 (1H, dd, *J* 15.9, 2.2, C(2)H_AH_B), 3.27 (1H, dd, *J* 15.9, 6.9, C(2)H_AH_B), 4.34 (1H, dd, *J* 6.9, 2.2, C(3)*H*), 7.06-7.11 (2H, m, C(3)PhC(2,6)*H*), 7.21-7.42 (10H, m, ArC(7,8)*H* & C(3)PhC(3,4,5)*H* & COPhC*H*), 7.58-7.64 (1H, m, ArC(6)*H*), 8.43-8.48 (1H, m, ArC(9)*H*).

Data were in accordance with those previously reported.[4]

(*S*)-4-Benzoyl-3-methyl-2,3-dihydro-1*H*-benzo[4,5]thiazolo[3,2-*a*]pyridin-1-one **45**

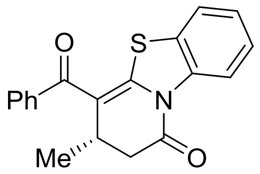

According to **General Procedure H**, (*E*)-but-2-enoic anhydride (34 mg, 0.22 mmol), 2-(benzo[*d*]thiazol-2-yl)-1-phenylethan-1-one (51 mg, 0.2 mmol), *i*-Pr_2NEt (45 μL, 0.26 mmol), HyperBTM (3.1 mg, 0.01 mmol) and THF (0.4 mL); followed by 3,5-bis(trifluoromethyl)phenol (12 μL, 0.08 mmol) gave after purification [eluent: 40%→80% CH_2Cl_2 in petrol] (*S*)-4-benzoyl-3-methyl-2,3-dihydro-1*H*-benzo[4,5]thiazolo[3,2-*a*]pyridin-1-one **45** as a yellow solid (56 mg, 0.17 mmol, 87%).

m.p. 148-149 °C {Lit: 156-157 °C}; $[\alpha]_D^{20}$ = +102 (*c* 1.0, $CHCl_3$) {Lit: +93.2 (*c* 0.5 in CH_2Cl_2, 93:7 er)}; **Chiral HPLC analysis**: CHIRALPAK AD-H (20:80 *i*-PrOH:hexane, flow rate 1.0 mL min^{-1}, 211 nm, 30 °C), t_R (*R*): 11.4 min, t_R (*S*): 14.4 min, 93.2:6.8 (*S*:*R*) er; **^{1}H NMR** (400 MHz, $CDCl_3$) δ_H: 1.11 (3H, d, *J* 6.9, C*H$_3$*), 2.73 (1H, dd, *J* 16.0, 2.2, C(2)H_AH_B), 3.05 (1H, dd, *J* 16.0, 6.3, C(2)H_AH_B), 3.30 (1H, app quin.d, *J* 6.9, 2.2, C(3)*H*), 7.31 (1H, app td, *J* 7.6, 1.2, ArC(7)*H*), 7.38 (1H, app td, *J* 7.5, 1.4, ArC(8)*H*), 7.42-7.54 (5H, m, PhC*H*), 7.55 (1H, app dd, *J* 7.6, 1.2, ArC(6)*H*), 8.55 (1H, app d, *J* 8.2, ArC(9)*H*).

Data were in accordance with those previously reported.[4]

(S)-4-Benzoyl-3-(furan-2-yl)-2,3-dihydro-1*H*-benzo[4,5]thiazolo[3,2-*a*]pyridin-1-one **46**

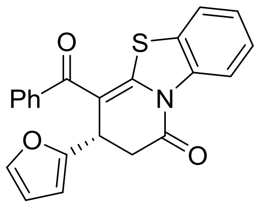

According to **General Procedure H**, (*E*)-3-(furan-2-yl)acrylic anhydride (57 mg, 0.22 mmol), 2-(benzo[*d*]thiazol-2-yl)-1-phenylethan-1-one (51 mg, 0.2 mmol), *i*-Pr_2NEt (45 μL, 0.26 mmol), HyperBTM (3.1 mg, 0.01 mmol) and THF (0.4 mL); followed by 3,5-bis(trifluoromethyl)phenol (12 μL, 0.08 mmol) gave after purification [eluent: 40%→80% CH_2Cl_2 in petrol] (*S*)-4-benzoyl-3-(furan-2-yl)-2,3-dihydro-1*H*-benzo[4,5]thiazolo[3,2-*a*]pyridin-1-one **46** as a yellow solid (64 mg, 0.17 mmol, 86%).

m.p. 126-130 °C {Lit: 127-128 °C}; $[\alpha]_D^{20}$ = −27.2 (*c* 1.0, $CHCl_3$) {Lit: −30.3 (*c* 1.0 in $CHCl_3$, 90:10 er)}; **Chiral HPLC analysis**: CHIRALPAK AD-H (20:80 *i*-PrOH:hexane, flow rate 1.0 mL min^{-1}, 211 nm, 30 °C), t_R (*R*): 15.0 min, t_R (*S*): 20.2 min, 93.4:6.6 (*S*:*R*) er; **^{1}H NMR** (400 MHz, $CDCl_3$) δ_H: 3.18 (1H, dd, *J* 16.1, 6.2, C(2)H_AH_B), 3.26 (1H, dd, *J* 16.1, 2.4, C(2)H_AH_B), 4.45 (1H, ddd, *J* 6.2, 2.4, 0.7, C(3)*H*), 6.00 (1H, app dt, *J* 3.2, 0.9, FurC(3)*H*), 6.25 (1H, dd, *J* 3.2, 1.9, FurC(4)*H*), 7.32-7.50 (8H, m, ArC(7,8)*H* & FurC(3)*H* & PhC*H*), 7.59-7.63 (1H, m, ArC(6)*H*), 8.50-8.55 (1H, m, ArC(9)*H*).

Data were in accordance with those previously reported.[4]

(*R*,*E*)-4-Benzoyl-3-styryl-2,3-dihydro-1*H*-benzo[4,5]thiazolo[3,2-*a*]pyridin-1-one **47**

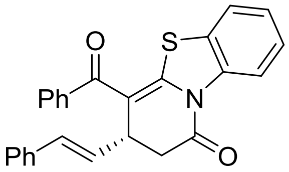

According to **General Procedure H**, (*E*,*E*)-5-phenylpenta-2,4-dienoic anhydride (73 mg, 0.22 mmol), 2-(benzo[*d*]thiazol-2-yl)-1-phenylethan-1-one (51 mg, 0.2 mmol), *i*-Pr_2NEt (45 μL, 0.26 mmol), HyperBTM (3.1 mg, 0.01 mmol) and THF (0.4 mL); followed by 3,5-bis(trifluoromethyl)phenol (12 μL, 0.08 mmol) gave after purification [eluent: 0%→25% EtOAc in petrol] *(R,E)-4-benzoyl-3-styryl-2,3-dihydro-1H-benzo[4,5]thiazolo[3,2-a]pyridin-1-one* **47** as a yellow solid (74 mg, 0.18 mmol, 90%).

m.p. 110-115 °C; $[\alpha]_D^{20}$ = −93.7 (*c* 1.0, $CHCl_3$); **Chiral HPLC analysis**: CHIRALPAK AD-H (20:80 *i*-PrOH:hexane, flow rate 1.0 mL min^{-1}, 211 nm, 30 °C), t_R (*S*): 13.1 min, t_R (*S*): 18.3 min, 91.2:8.8 (*R*:*S*) er; **^{1}H NMR** (400 MHz, $CDCl_3$) δ_H: 2.97 (1H, dd, *J* 16.0, 2.2, C(2)H_AH_B), 3.14 (1H, dd, *J* 16.0, 6.5, C(2)H_AH_B), 3.92-3.99 (1H, m, C(3)*H*), 6.19 (1H, dd, *J* 16.0, 5.7, C(3)C*H*=CH), 6.28 (1H, dd, *J* 16.0, 1.0, C(3)CH=C*H*), 7.18-7.25 (1H, m,), 7.26-7.29 (4H, m,), 7.30-7.50 (5H, m, ArC(7,8)*H* &), 7.56-7.61 (3H, m, ArC(6)*H* &), 8.49-8.53 (1H, m, ArC(9)*H*); **^{13}C{^{1}H} NMR** (126 MHz, $CDCl_3$) δ_C: 35.9 (*C*(3)H), 38.8 (*C*(2)H_2), 106.9 (*C*(4)), 117.7 (Ar*C*(9)H), 122.0 (Ar*C*(6)H), 125.9 (Ar*C*(7)H), 126.5 (CHPh*C*(2,6)H), 127.1 (COPh*C*(2,6)H), 127.1 (Ar*C*(8)H), 127.7 (*C*(6a)), 127.9 (CHPh*C*(4)H), 128.0, (C(3)*C*H=CH), 128.3 (COPh*C*(3,5)H), 128.6 (CHPh*C*(3,5)H), 130.3 (COPh*C*(4)H), 131.7 (C(3)CH=*C*H), 136.1 (*C*(9a)), 136.2

(CHPh*C*(1)), 139.6 (COPh*C*(1)), 155.7 (*C*(5)), 168.2 (N*C*(1)=O), 191.1 (*C*=O); **IR** (neat) ν_{max} cm^{-1} 3061, 3024, 1724 (C=O), 1611, 1485, 1358, 1335, 1296, 1277, 1173, 1134; **HRMS** $(NSI)^+$ calculated for $C_{26}H_{20}O_2NS^+$ ($[M+H]^+$) requires 410.1209; found 410.1202 (−1.8 ppm).

Kinetic Resolution Studies

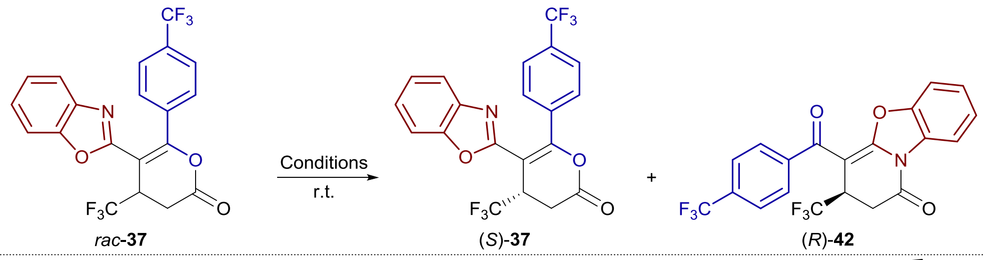

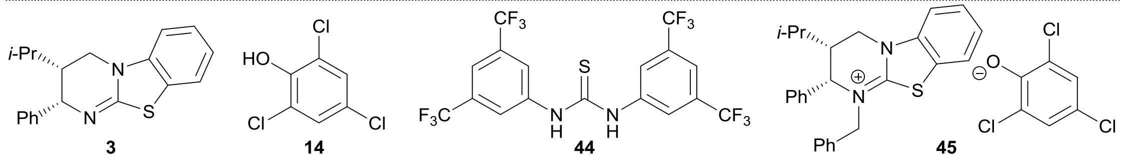

Entry	Conditions	Conv. (%)	37 er	42 er
1	**3** (10 mol%)	55	77:23	74:26
2	**3** (10 mol%) + **14** (1 equiv.)	56	92:8	85:15
3	**3** (10 mol%) + $PhCO_2H$ (10 mol%)	53	92:8	89:11
4	**3** (10 mol%) + **44** (10 mol%)	56	92:8	83:17
5	**45** (10 mol%)	64	50:50	50:50

Procedure:

A racemic sample of 5-(benzo[*d*]oxazol-2-yl)-4-(trifluoromethyl)-6-(4-(trifluoromethyl)phenyl)-3,4-dihydro-2*H*-pyran-2-one (±)-**37** (21.4 mg, 0.05 mmol) and the additives stated in the table were allowed to stir in THF (0.2 mL). Samples were removed periodically, reduced to dryness under a flow of compressed air and dissolved in $CDCl_3$ for analysis by ^1H NMR spectroscopy. The $CDCl_3$ was then removed under a flow of compressed air and the sample dissolved in *i*-PrOH for analysis by chiral HPLC.

Selectivity factors (*s*) were calculated using the following equations, with %ees determined by chiral HPLC analysis. Conversion was measured by ^1H NMR spectroscopy and calculated using HPLC analysis (see below). See reference 9 for the derivation, and alternative forms, of these equations.

$$s = \frac{\ln[(1\text{-conv})(1\text{-}\%ee_{alcohol})]}{\ln[(1\text{-conv})(1^{+}\%ee_{alcohol})]}$$

where both ee and conv are given as between 0 and 1

$$\text{and,} \quad \text{conv} = \frac{\%ee_{alcohol}}{\%ee_{alcohol} + \%ee_{ester}}$$

Data for Kinetic Resolutions (Table 4):

Entry 1: [HyperBTM (2*S*,3*R*)-**3** (10 mol%)]

Time (h)	%ee$_{lactone}$	%ee$_{lactam}$	conv (NMR)	conv (HPLC)	*s*
3	0.818	67.896	1	1.19	5.3
8	2.188	76.14	3	2.79	7.5
23	5.952	78.044	8	7.09	8.6
47	21.036	62.718	27	25.12	5.3
71	38.046	54.35	42	41.18	4.8
100	53.443	47.058	55	53.18	4.6
144	69.654	38.9	65	64.17	4.5
198	78.718	28.746	73	73.25	3.9
360	74.186	13.362	86	84.74	2.4

Entry 2: [HyperBTM (2*S*,3*R*)-**3** (10 mol%) + Trichlorophenol **14** (1 equiv.)]

Time (h)	%ee$_{lactone}$	%ee$_{lactam}$	conv (NMR)	conv (HPLC)	*s*
3	2.972	78.946	4	3.63	8.8
8	7.74	86.554	10	8.21	15.0
23	17.612	88.316	17	16.63	19.1
47	40.224	85.29	33	32.05	18.7
71	58.416	82.904	42	41.34	19.2
100	73.61	78.096	49	48.52	17.8
144	84.508	69.036	56	55.04	14.3
198	89.918	58.102	61	60.75	11.0
360	87.842	31.442	74	73.64	4.9

Entry 3: [HyperBTM (2*S*,3*R*)-**3** (10 mol%) + $PhCO_2H$ (10 mol%)]

Time (h)	%ee$_{lactone}$	%ee$_{lactam}$	conv (NMR)	conv (HPLC)	*s*
5	3.528	78.942	4	4.28	8.8
23	19.29	89.742	17	17.69	22.3
54	38.332	88.954	31	30.11	24.9
78	53.582	87.144	39	38.08	24.8
102	64.786	85.35	45	43.15	24.6
170	84.974	78.502	53	51.98	22.2
270	90.92	67.782	58	57.29	15.9

Entry 4: [HyperBTM (2*S*,3*R*)-**3** (10 mol%) + **44** (10 mol%)]

Time (h)	%ee$_{lactone}$	%ee$_{lactam}$	conv (NMR)	conv (HPLC)	*s*
6	3.328	68.58	5	4.63	5.5
25	13.632	80.632	16	14.46	10.7
52	28.076	80.27	25	25.91	12.0
97	49.324	75.336	41	39.57	11.5
166	70.576	71.948	49	49.52	12.8
240	84.428	65.116	56	56.46	12.3
360	89.396	55.282	63	61.79	9.9

Entry 5: [**45** (10 mol%)]

Time (h)	%ee$_{lactone}$	%ee$_{lactam}$	conv (NMR)	conv (HPLC)	*s*
1	0.012	2.17	7	0.55	1.0
6	0.526	0.234	29	69.21	1.0
24	0.232	0.006	64	97.48	1.0
48	0.396	0.056	79	87.61	1.0
77	0.514	0.094	88	84.54	1.0
102	0.14	0.102	90	57.85	1.0

(2*S*,3*R*)-1-Benzyl-3-isopropyl-2-phenyl-3,4-dihydro-2*H*-benzo[4,5]thiazolo[3,2-*a*]pyrimidin-1-ium bromide **S15**

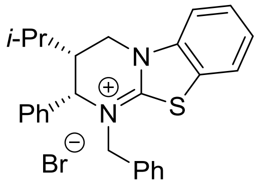

Benzyl bromide (60 μL, 0.5 mmol, 1.4 equiv.) was added to a solution of (2*S*,3*R*)-3-isopropyl-2-phenyl-3,4-dihydro-2*H*-benzo[4,5]thiazolo[3,2-*a*]pyrimidine (HyperBTM) **3** (108 mg, 0.35 mmol) in anhydrous CH_2Cl_2 (1 mL) under a nitrogen atmosphere and allowed to stir at r.t. for 16 h. Et_2O (15 mL) was added and the precipitate filtered and dried under high vacuum to give *(2S,3R)-1-benzyl-3-isopropyl-2-phenyl-3,4-dihydro-2H-benzo[4,5]thiazolo[3,2-a]pyrimidin-1-ium bromide* **S15** as a colourless solid (151 mg, 0.32 mmol, 90%). *Note: NMR chemical shifts of salts can vary with concentration, therefore slight variation in the reported shifts may be observed depending on sample concentration.*

m.p. 105-112 °C; $[\alpha]_D^{20}$ = +225 (*c* 1.0, $CHCl_3$); **^{1}H NMR** (400 MHz, CD_2Cl_2) δ_H: 0.92 (3H, d, *J* 6.7, $CH(CH_3)_A(CH_3)_B$), 1.12 (3H, d, *J* 6.5, $CH(CH_3)_A(CH_3)_B$), 1.32-1.42 (1H, m, $CH(CH_3)_2$), 2.49-2.61 (1H, m, C(3)*H*), 3.84 (1H, app t, *J* 12.8, C(4)H_AH_B), 4.58 (1H, ddd, *J* 13.1, 5.2, 1.2, C(4)H_AH_B), 4.72 (1H, d, *J* 15.6, NCH_AH_BPh), 4.99 (1H, d, *J* 15.6, NCH_AH_BPh), 5.12 (1H, app d, *J* 4.6, C(2)*H*), 7.06-7.13 (2H, m, C(2)PhC(2,6)*H*), 7.26-7.33 (2H, m, CH_2PhC(2,6)*H*), 7.33-7.44 (6H, m, CH_2PhC(3,4,5)*H* & C(2)PhC(3,4,5)*H*), 7.46-7.52 (1H, m, ArC(8)*H*), 7.62-7.70 (1H, m, ArC(7)*H*), 7.76 (1H, app d, *J* 8.3,

ArC(6)*H*), 8.16 (1H, dd, *J* 8.1, 0.7, ArC(9)*H*); **^{13}C{^{1}H} NMR** (126 MHz, CD_2Cl_2) δ_C: 20.0 (CH(*C*H_3)$_A$(CH_3)$_B$), 21.7 (CH(CH_3)$_A$(*C*H_3)$_B$), 27.3 (*C*H(CH_3)$_2$), 41.7 (*C*(3)H), 43.8 (*C*(4)H_2), 59.4 (N*C*H_2Ph), 63.8 (*C*(2)H), 113.8 (Ar*C*(6)H), 122.3 (*C*(9a)), 124.6 (Ar*C*(9)H), 126.7 (Ar*C*(8)H), 128.3 (C(2)Ph*C*(2,6)H), 129.0 (CH_2Ph*C*(2,6)H), 129.3 (Ar*C*(7)H), 129.7 (CH_2Ph*C*(3,5)H), 129.8 (CH_2Ph*C*(4)H), 129.9 (C(2)Ph*C*(3,5)H), 130.0 (C(2)Ph*C*(4)H), 132.0 (CH_2Ph*C*(1)), 134.7 (C(2)Ph*C*(1)), 139.4 (*C*(5a)), 165.6 (*C*(10a)); **IR** (neat) ν_{max} cm^{-1} 2911, 1605, 1589, 1582, 1454, 1412, 1373, 1321, 1244; **HRMS** (NSI)$^+$ calculated for $C_{26}H_{27}N_2S^+$ ([M−Br]$^+$) requires 399.1889; found 399.1883 (−1.6 ppm).

(2*S*,3*R*)-1-Benzyl-3-isopropyl-2-phenyl-3,4-dihydro-2*H*-benzo[4,5]thiazolo[3,2-*a*]pyrimidin-1-ium 2,4,6-trichlorophenolate **49**

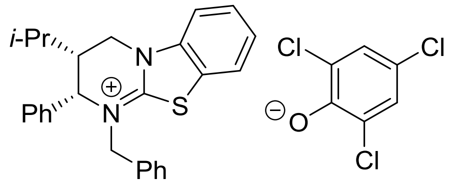

Amberlyst® A26 (hydroxide form) (300 mg) was added to a solution of (2*S*,3*R*)-1-benzyl-3-isopropyl-2-phenyl-3,4-dihydro-2*H*-benzo[4,5]thiazolo[3,2-*a*]pyrimidin-1-ium bromide **S15** (144 mg, 0.3 mmol) in anhydrous MeOH (3 mL) under a nitrogen atmosphere and allowed to stir at r.t. for 14 h. The mixture was filtered and the solids washed with anhydrous MeOH (2 mL). Trichlorophenol (593 mg, 0.3 mmol) was added to the combined filtrates and the solution was allowed to stir for 15 min. The solvent was removed *in vacuo*. *n*-Hexane (5 mL) was added and the solvent removed *in vacuo*. This process was repeated 3 times, and the residue dried under high vacuum. The residue was dissolved in CH_2Cl_2, and Et_2O added to give a precipitate, which was filtered and dried under high vacuum to give *(2S,3R)-1-benzyl-3-isopropyl-2-phenyl-3,4-dihydro-2H-benzo[4,5]thiazolo[3,2-a]pyrimidin-1-ium 2,4,6-trichlorophenolate* **49** as a pale yellow solid (103 mg, 0.17 mmol, 58%). *Note: NMR chemical shifts of salts can vary with concentration, therefore slight variation in the reported shifts may be observed depending on sample concentration.*

$[\alpha]_D^{20}$ = +139 (*c* 0.1, $CHCl_3$); **^{1}H NMR** (500 MHz, $CDCl_3$) δ_H: 0.89 (3H, d, *J* 6.8, CH(C*H*$_3$)$_A$(CH_3)$_B$), 1.06 (3H, d, *J* 6.5, CH(CH_3)$_A$(C*H*$_3$)$_B$), 1.24-1.35 (1H, m, C*H*(CH_3)$_2$), 2.56-2.65 (1H, m, C(3)*H*), 3.77 (1H, app t, *J* 12.8, C(4)H_AH_B), 4.56 (1H, dd, *J* 12.8, 4.8, C(4)H_AH_B), 4.68 (1H, d, *J* 15.6, NCH_AH_BPh), 5.02 (1H, d, *J* 15.6, NCH_AH_BPh), 5.05 (1H, d, *J* 4.4, C(2)*H*), 6.92 (2H, s, Ar_{Cl3}C(3,5)*H*), 6.97-7.03 (2H, m, C(2)PhC(2,6)*H*), 7.15-7.20 (2H, m, CH_2PhC(2,6)*H*), 7.22-7.28 (3H, m, CH_2PhC(3,4,5)*H*), 7.28-7.35 (3H, m, C(2)PhC(3,4,5)*H*), 7.37 (1H, app t, *J* 7.8, ArC(8)*H*), 7.54 (1H, app t, *J* 7.8, ArC(7)*H*), 7.67 (1H, app d, *J* 8.3, ArC(6)*H*), 8.01 (1H, app d, *J* 8.1, ArC(9)*H*); **^{13}C{^{1}H} NMR** (126 MHz, $CDCl_3$) δ_C: 19.8 (CH(*C*H_3)$_A$(CH_3)$_B$), 21.4 (CH(CH_3)$_A$(*C*H_3)$_B$), 26.8 (*C*H(CH_3)$_2$), 40.9 (*C*(3)H), 43.3 (*C*(4)H_2), 59.3 (N*C*H_2Ph), 63.5 (*C*(2)H), 113.2 (Ar*C*(6)H), 118.6 (Ar_{Cl3}*C*(4)), 121.9 (*C*(9a)), 124.0 (Ar*C*(9)H), 124.1 (Ar_{Cl3}*C*(2,6)), 126.2 (Ar*C*(8)H), 127.4 (Ar_{Cl3}*C*(3,5)H), 127.8 (C(2)Ph*C*(2,6)H), 128.76 (CH_2Ph*C*(2,6)H), 128.84 (Ar*C*(7)H), 129.26 (CH_2Ph*C*(3,5)H), 129.34 (CH_2Ph*C*(4)H), 129.6 (C(2)Ph*C*(3,4,5)H), 131.4 (CH_2Ph*C*(1)), 134.5 (C(2)Ph*C*(1)), 138.7 (*C*(5a)), 153.7 (Ar_{Cl3}*C*(1)), 165.1 (*C*(10a)); **IR** (neat) ν_{max} cm^{-1} 2963, 2924, 1634, 1605, 1582, 1495, 1472, 1454, 1371, 1246, 1217; **HRMS** (NSI)$^+$ calculated for $C_{20}H_{12}O_3N_2F_3^+$ $C_{26}H_{27}N_2S^+$ ([M−$OC_6H_2Cl_3$]$^+$) requires 399.1889; found 399.1880 (−2.4 ppm).

Single Crystal X-ray Analysis: Absolute Configuration of (*S*)-18 and (*S*)-41

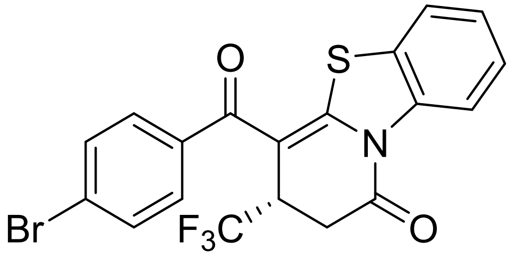

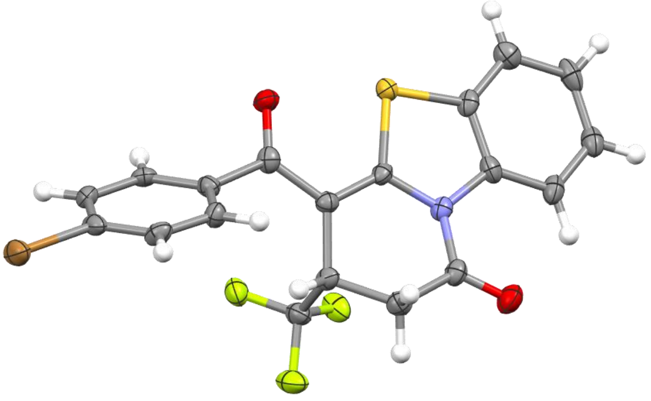

(*S*)-**18**

Crystallographic data have been deposited
with the Cambridge Crystallographic Data Centre (CCDC **1827462**)

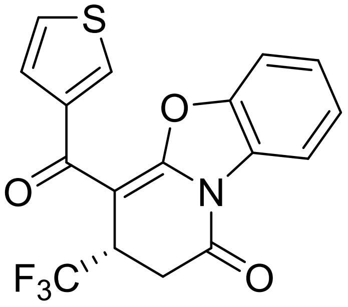

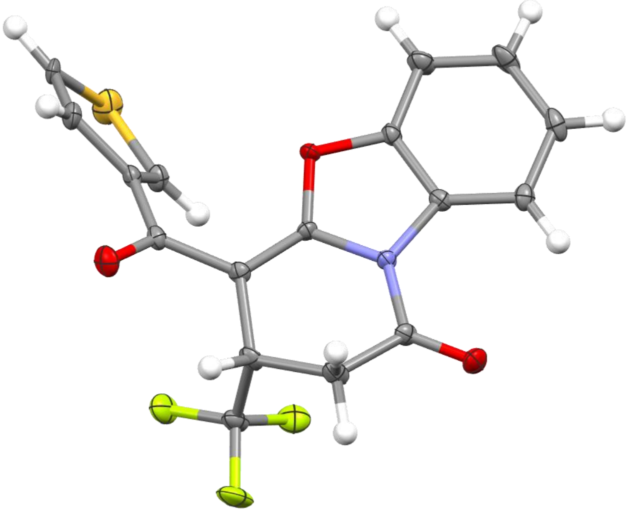

(*S*)-**41**

Crystallographic data have been deposited
with the Cambridge Crystallographic Data Centre (CCDC **1827463**)

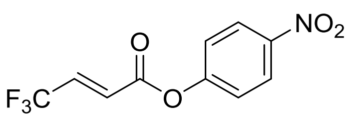

7

^{1}H, 400 MHz, $CDCl_3$

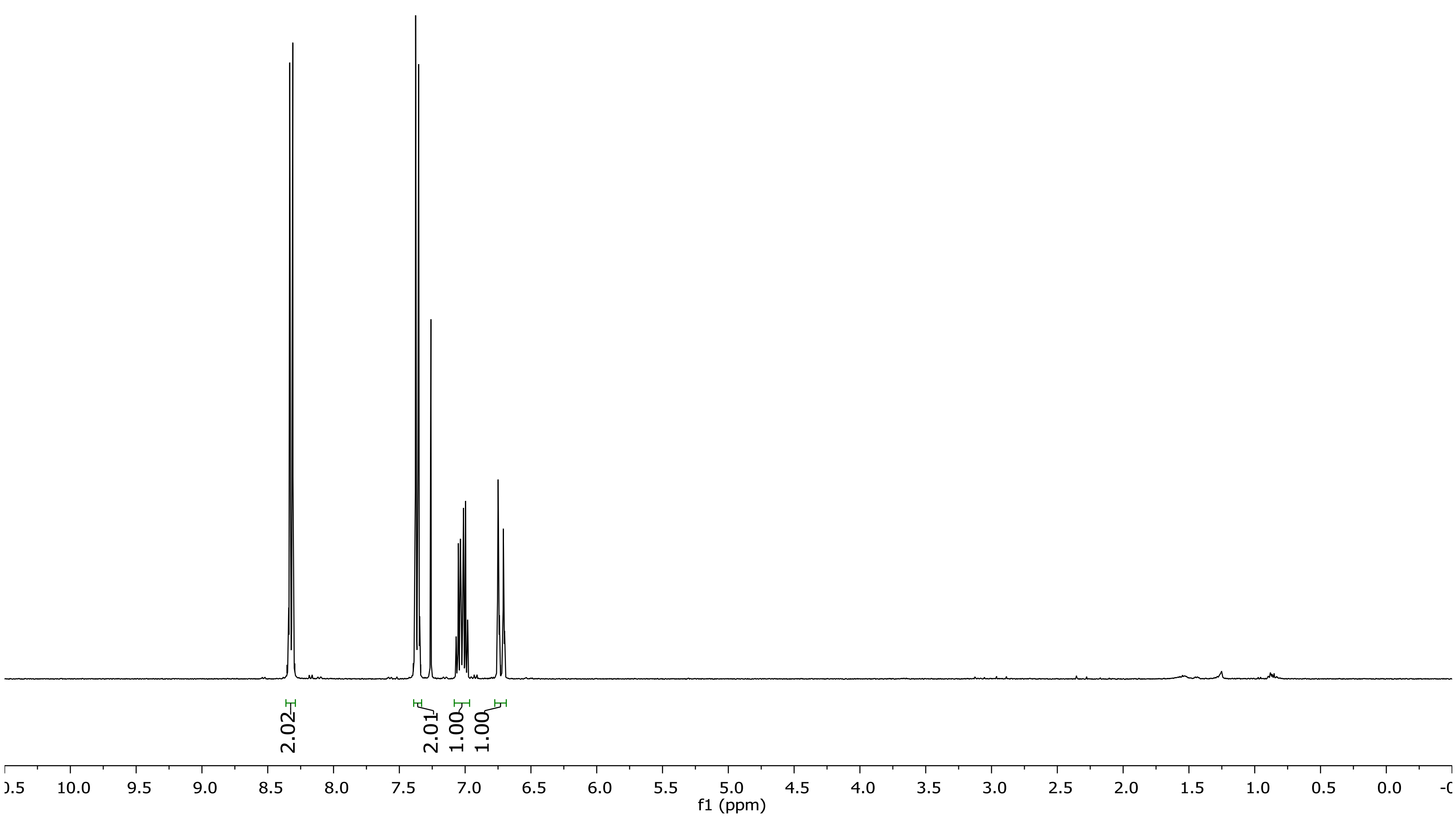

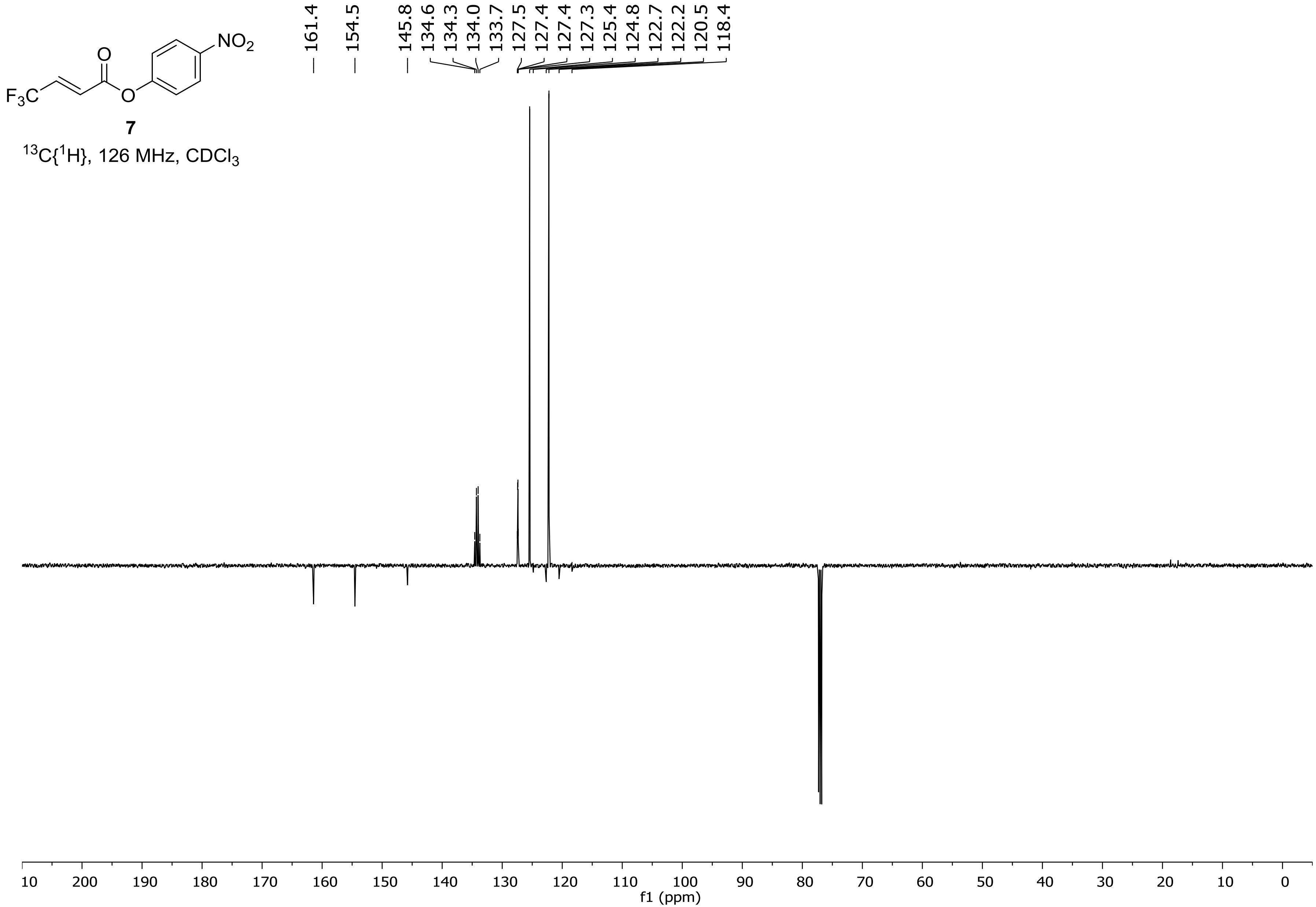
F3C
O
O
NO2
7
13C{1H}, 126 MHz, CDCl3
161.4
154.5
145.8
134.6
134.3
134.0
133.7
127.5
127.4
127.4
127.3
125.4
124.8
122.7
122.2
120.5
118.4
10
200
190
180
170
160
150
140
130
120
110
100
90
80
70
60
50
40
30
20
10
0
f1 (ppm)

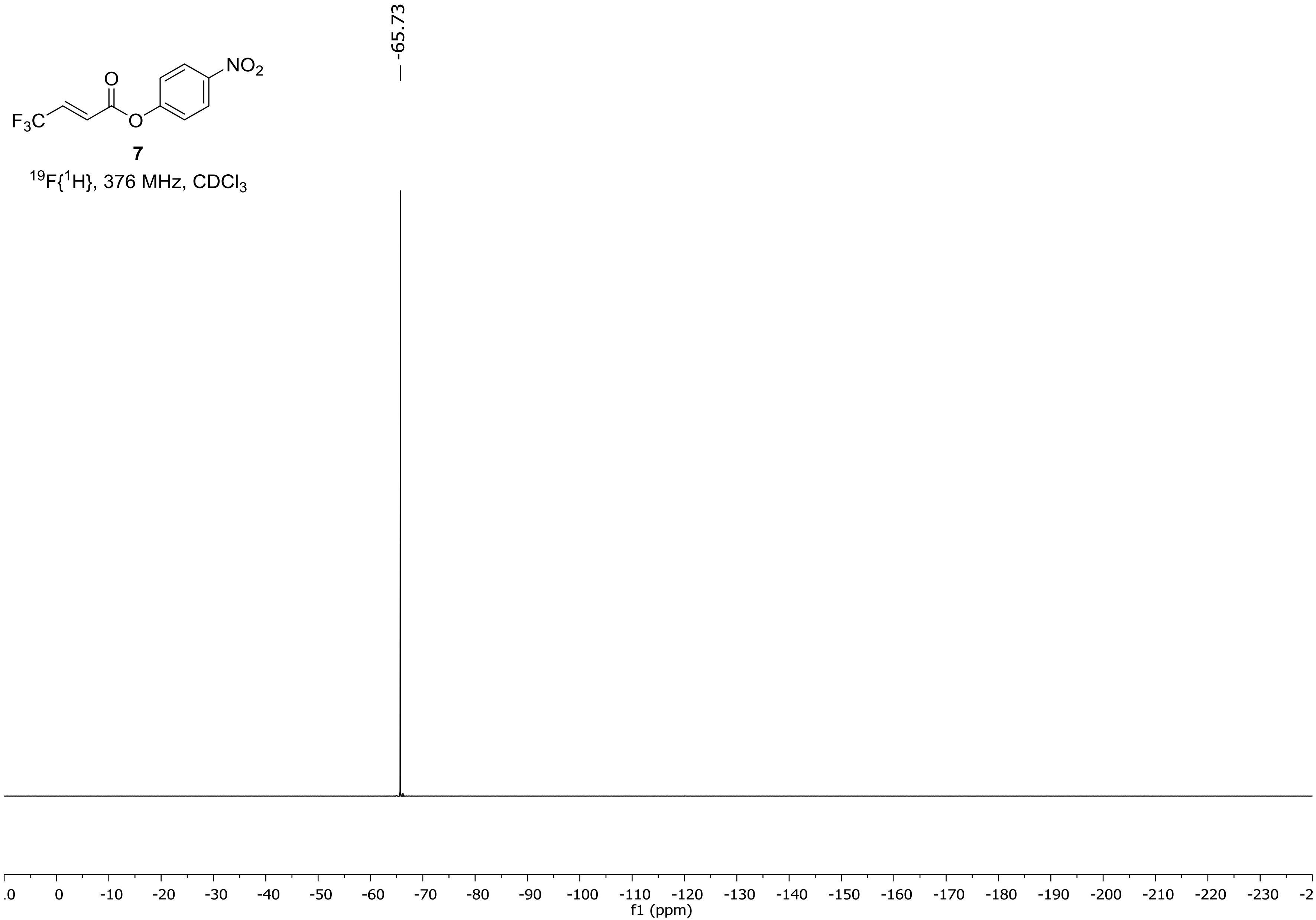

-65.73
NO2
O
F3C
O
7
19F{1H}, 376 MHz, CDCl3
.0 0 -10 -20 -30 -40 -50 -60 -70 -80 -90 -100 -110 -120 -130 -140 -150 -160 -170 -180 -190 -200 -210 -220 -230 -2
f1 (ppm)

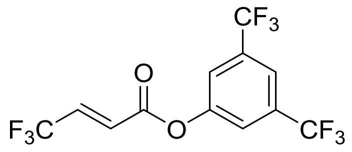

8

^{1}H, 500 MHz, $CDCl_3$

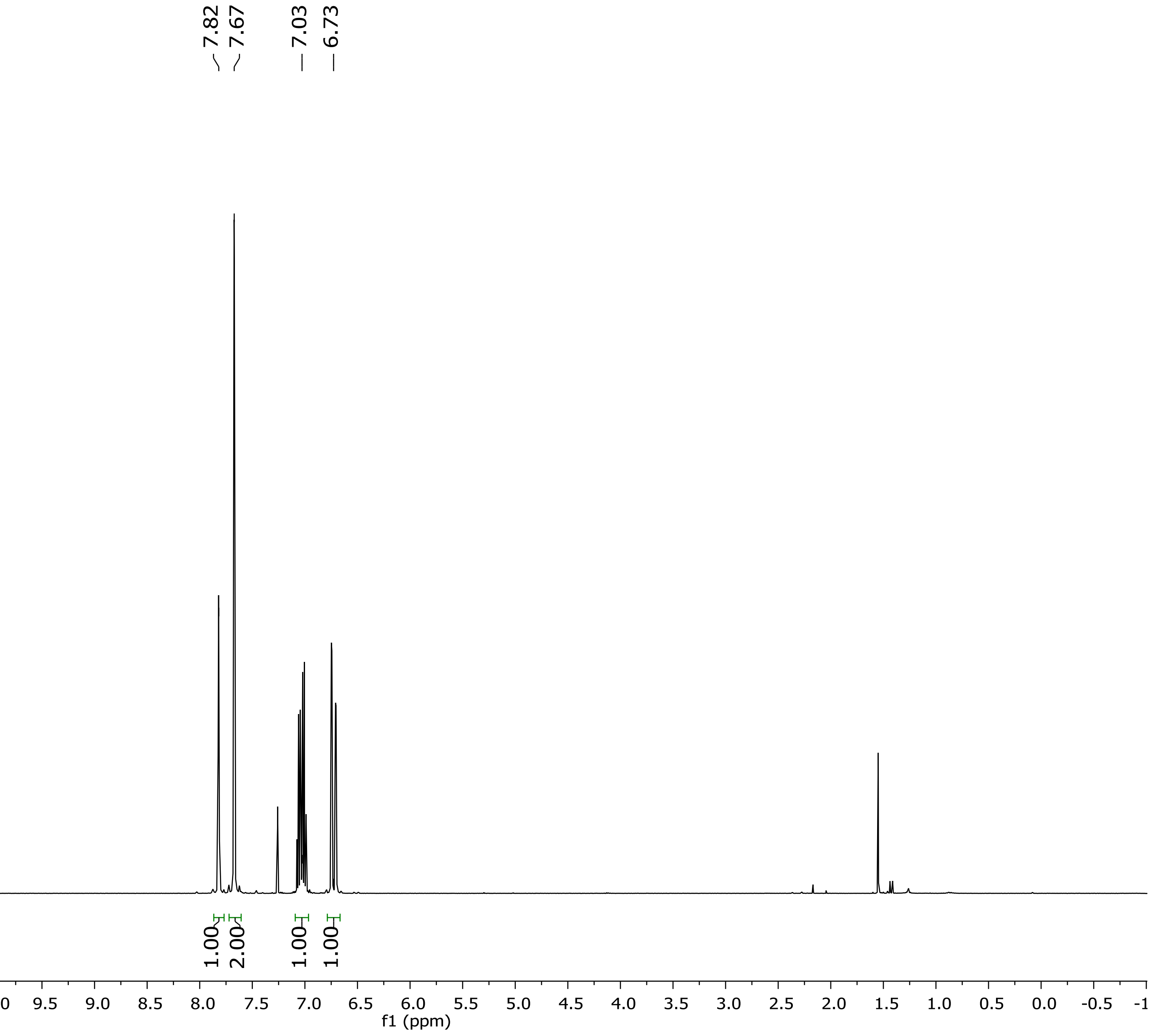

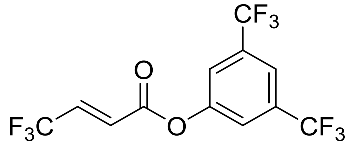

8

$^{13}C\{^{1}H\}$, 126 MHz, $CDCl_3$

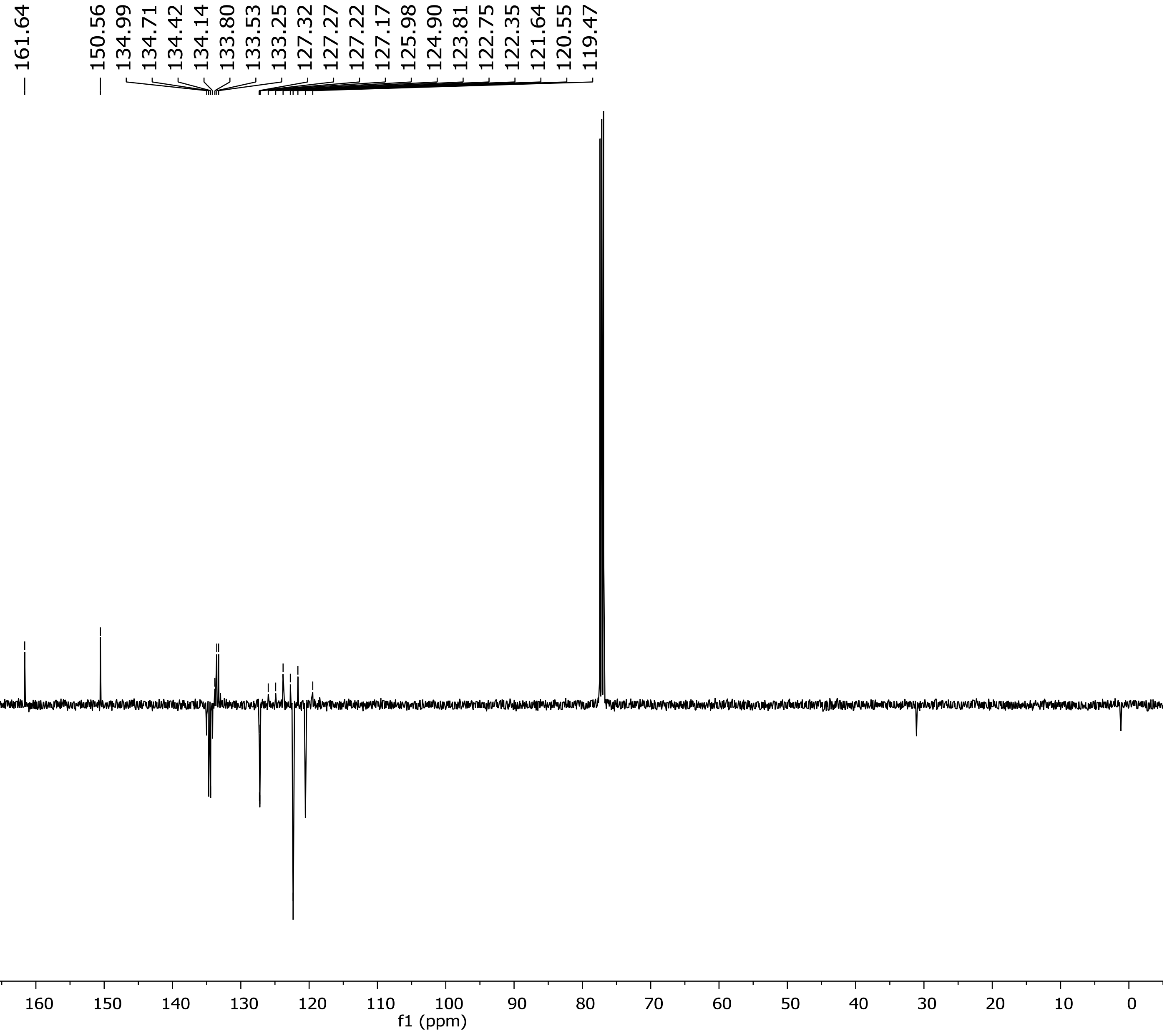

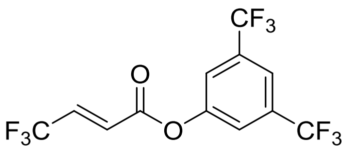

8

$^{19}F\{^{1}H\}$, 376 MHz, $CDCl_3$

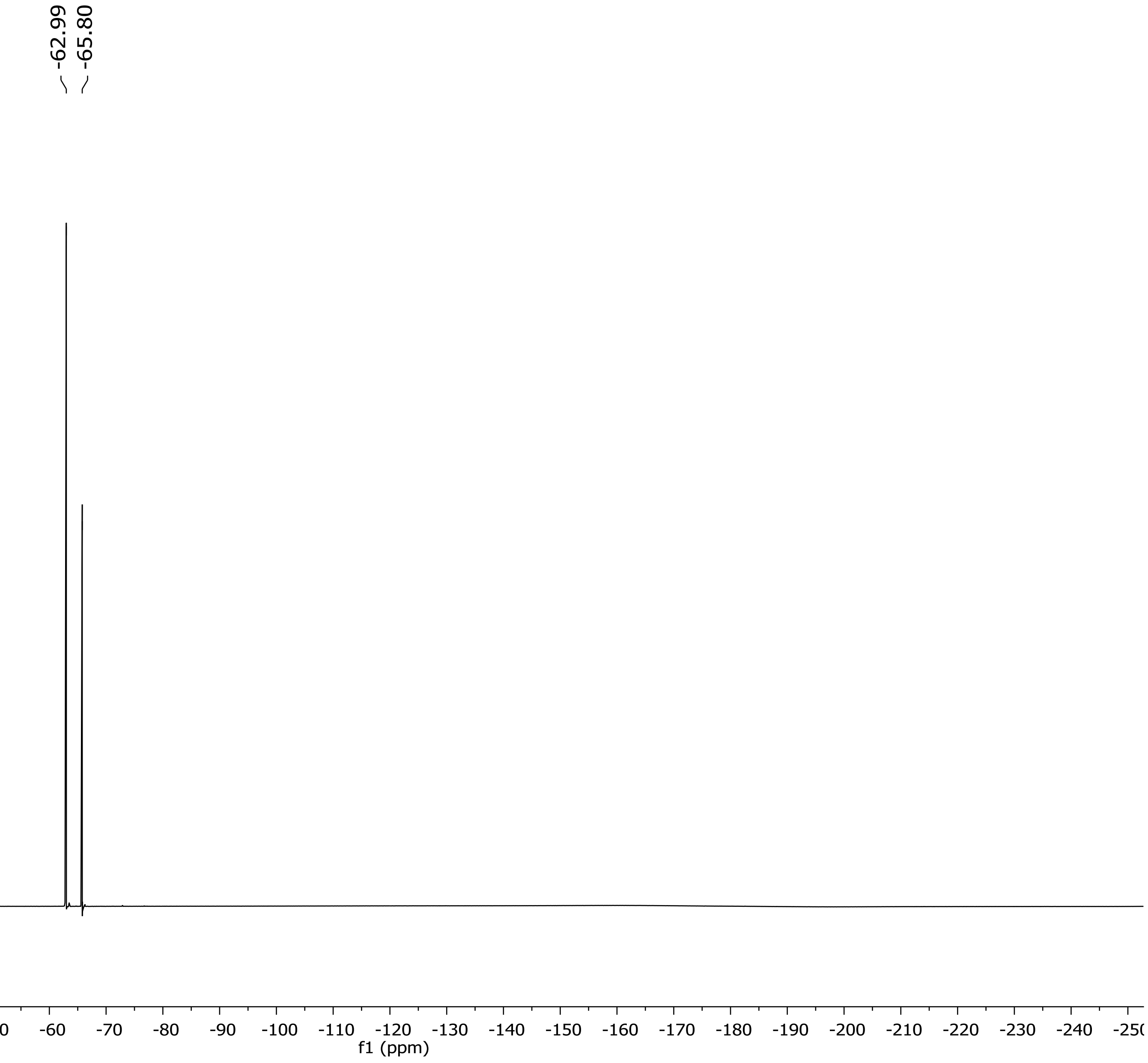

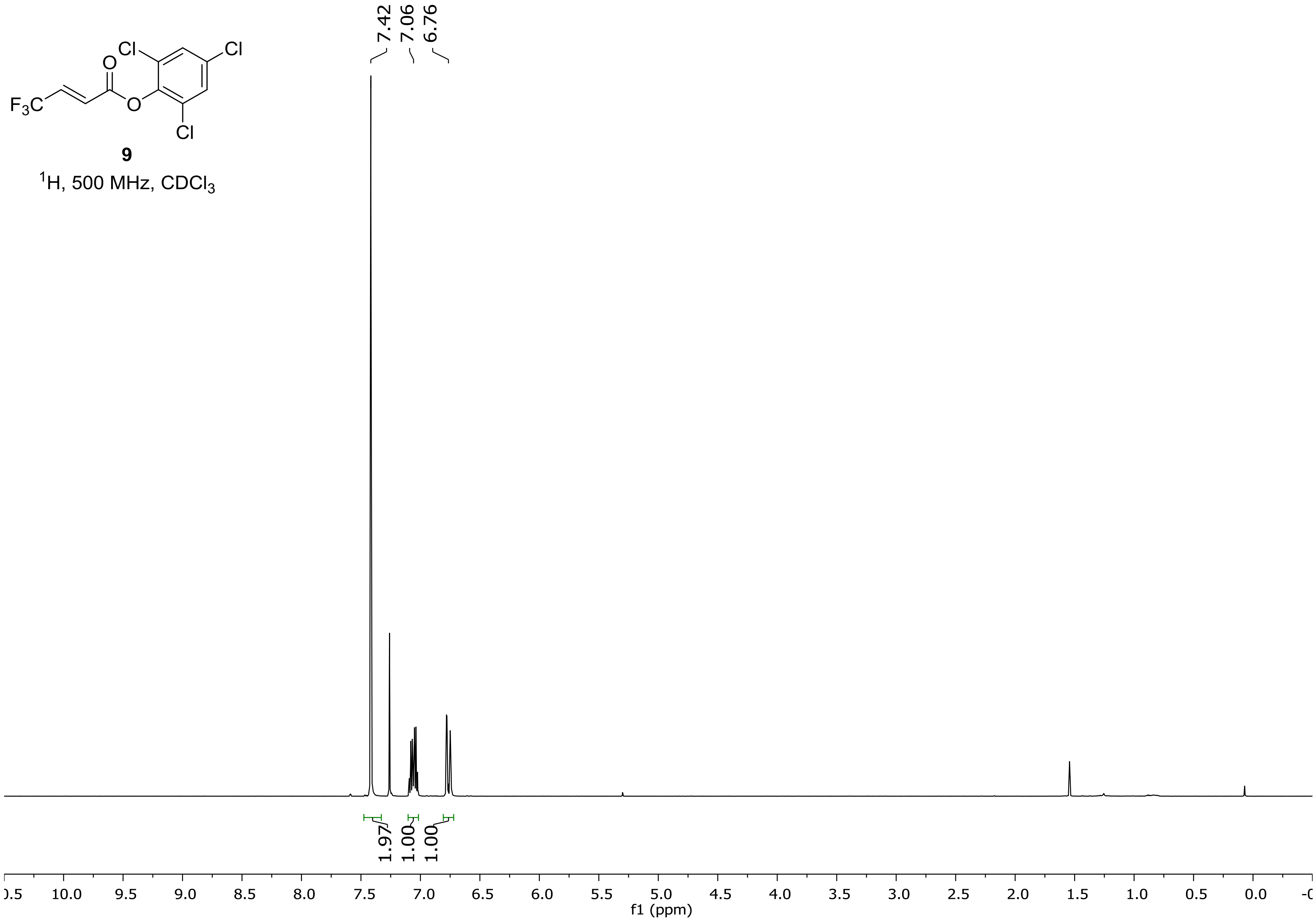

Cl
Cl
O
F3C
O
Cl
9
1H, 500 MHz, CDCl3
7.42
7.06
6.76
1.97
1.00
1.00
10.0
9.5
9.0
8.5
8.0
7.5
7.0
6.5
6.0
5.5
5.0
4.5
4.0
3.5
3.0
2.5
2.0
1.5
1.0
0.5
0.0
f1 (ppm)

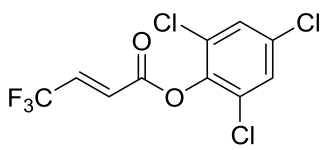

9

$^{13}C\{^{1}H\}$, 126 MHz, $CDCl_3$

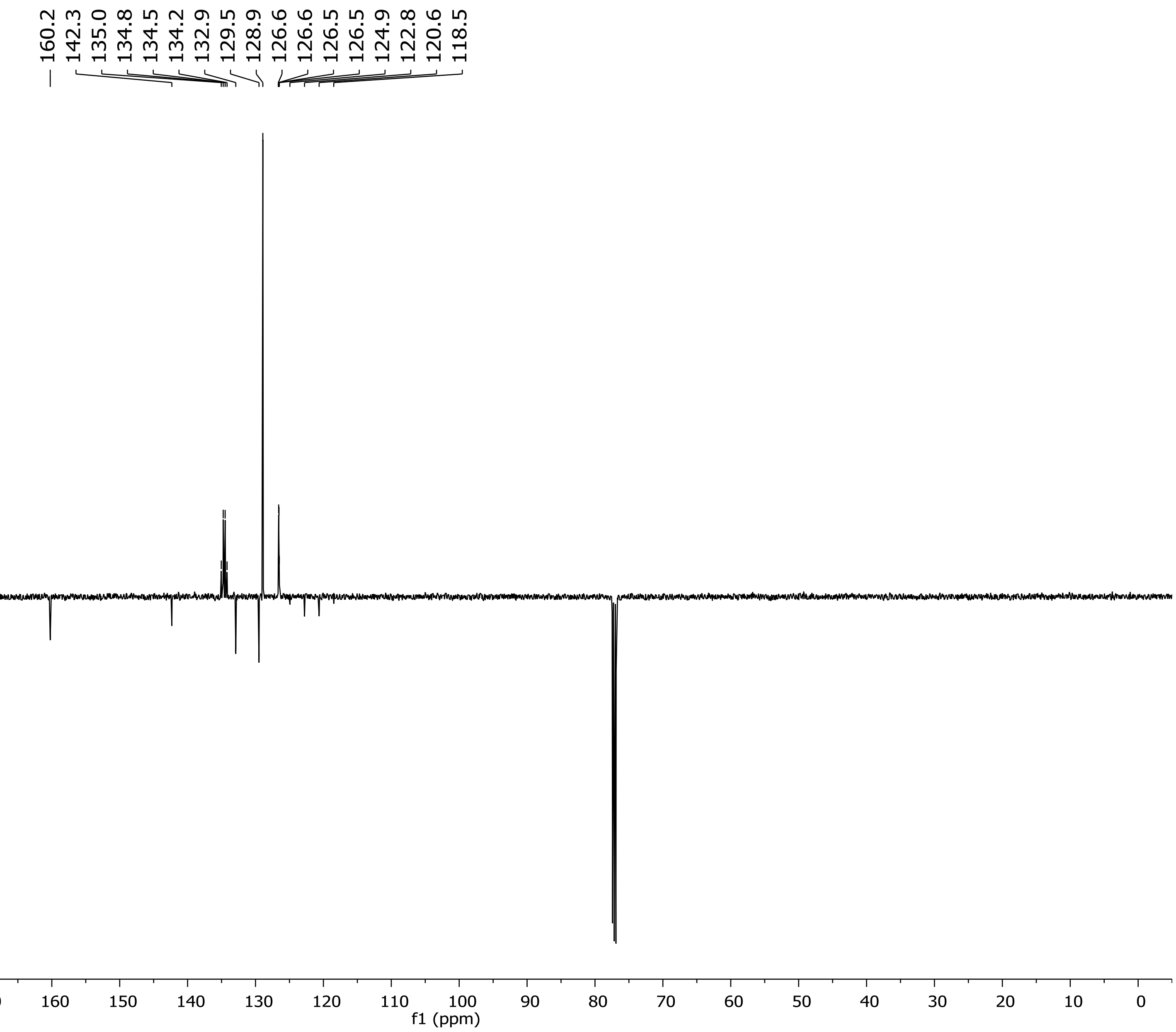

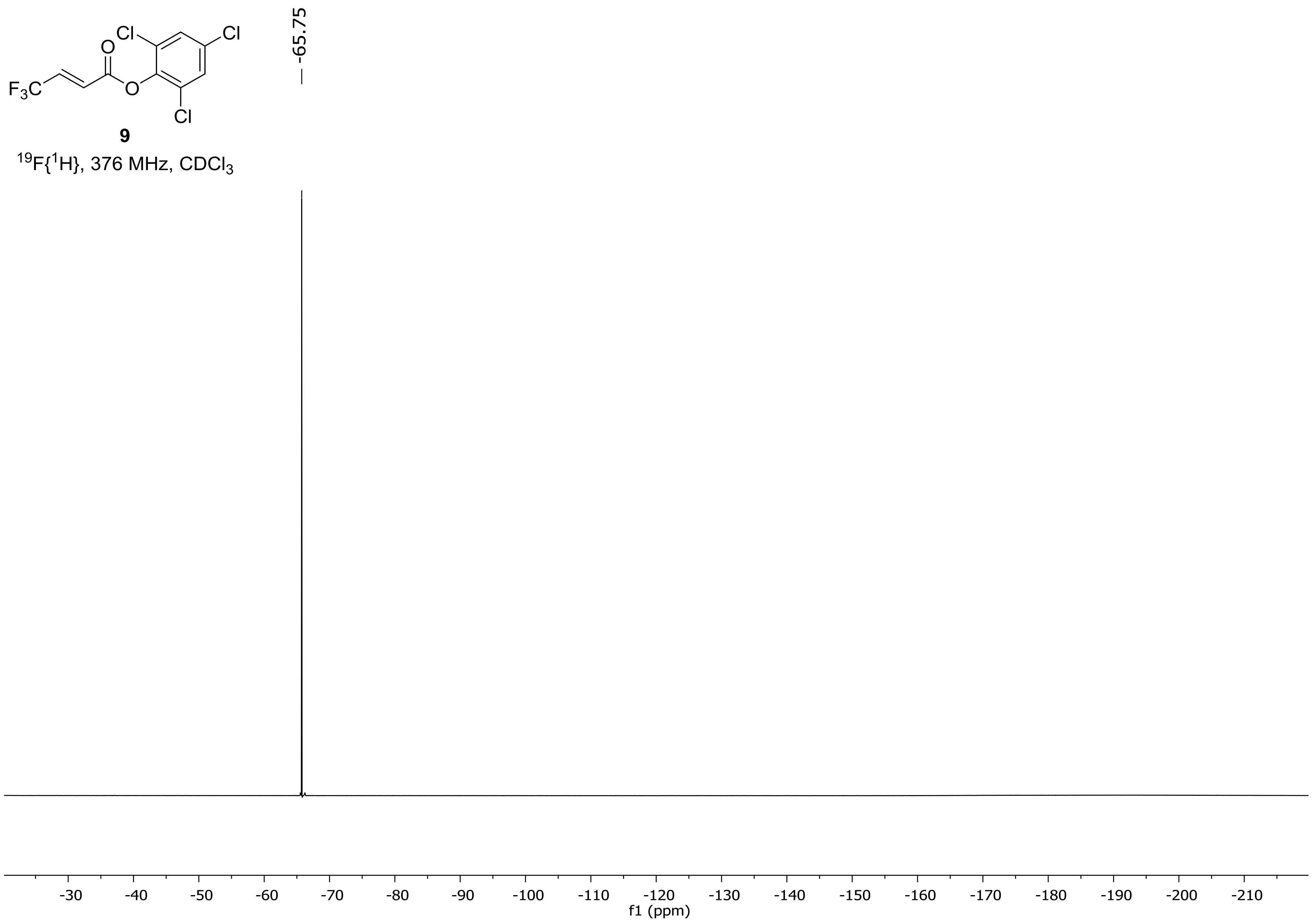

9
$^{19}F\{^1H\}$, 376 MHz, $CDCl_3$
-65.75
-30
-40
-50
-60
-70
-80
-90
-100
-110
-120
-130
-140
-150
-160
-170
-180
-190
-200
-210
f1 (ppm)

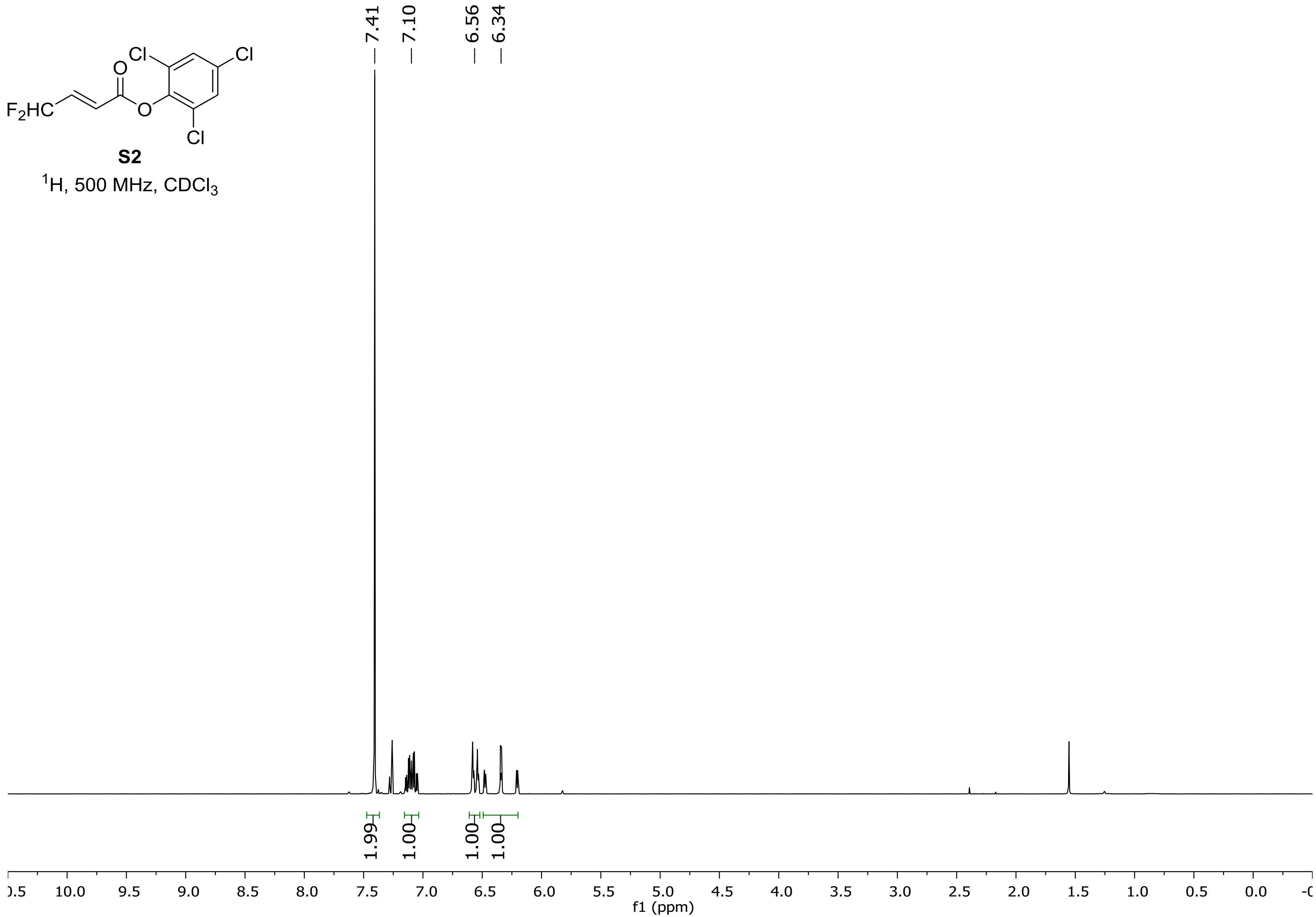
F2HC
O
Cl
Cl
Cl
S2
1H, 500 MHz, CDCl3
7.41
7.10
6.56
6.34
1.99
1.00
1.00
1.00
f1 (ppm)

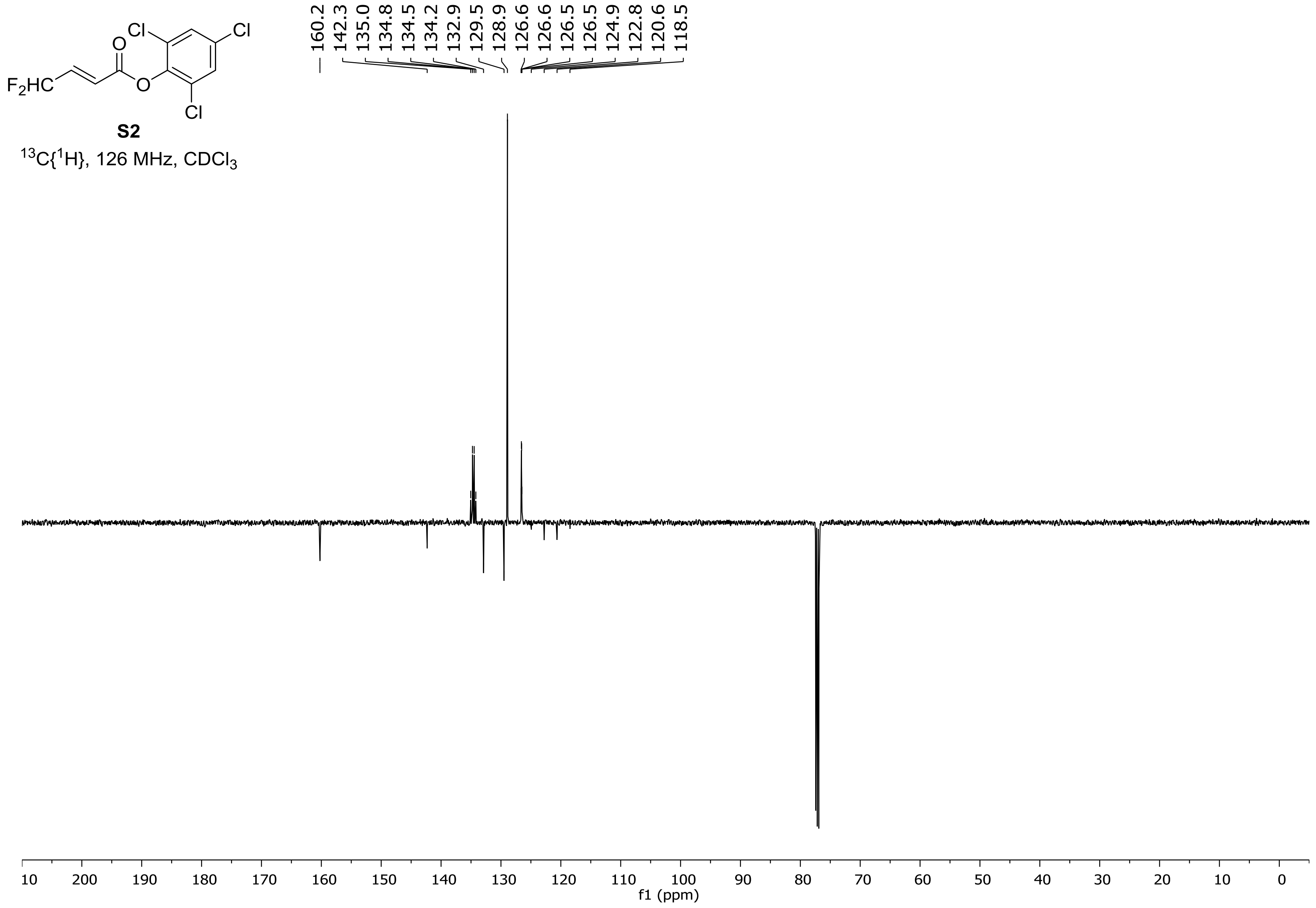

Cl
Cl
O
F2HC
O
Cl
S2
13C{1H}, 126 MHz, CDCl3
160.2
142.3
135.0
134.8
134.5
134.2
132.9
129.5
128.9
126.6
126.6
126.5
126.5
124.9
122.8
120.6
118.5
10
200
190
180
170
160
150
140
130
120
110
100
90
80
70
60
50
40
30
20
10
0
f1 (ppm)

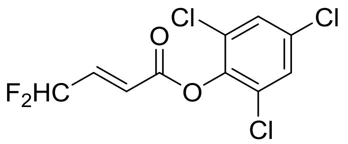

S2

$^{19}F\{^{1}H\}$, 376 MHz, $CDCl_3$

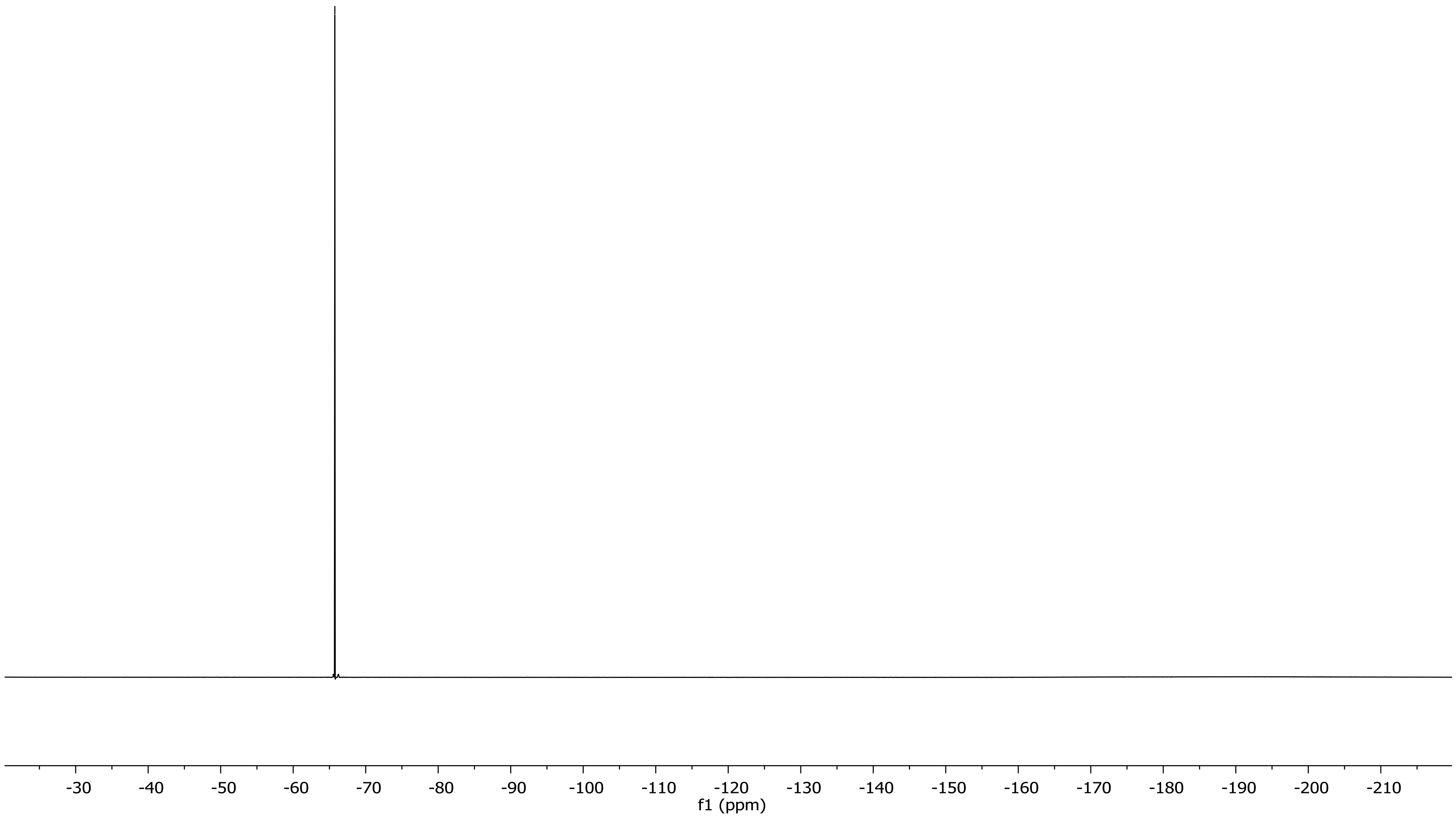

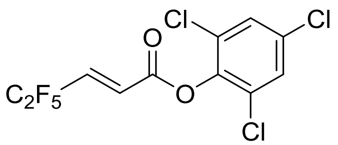

S3

^1H, 500 MHz, $CDCl_3$

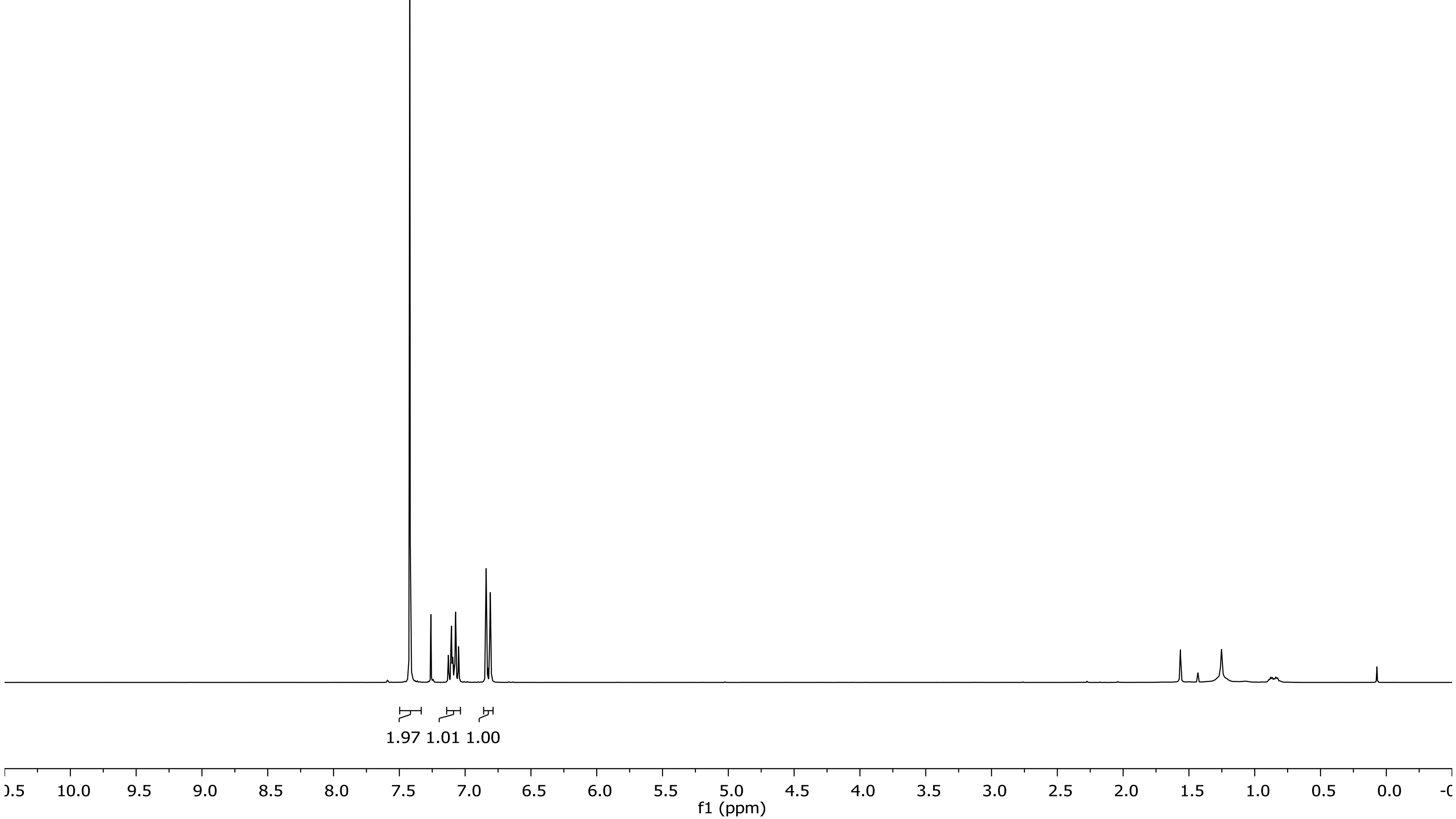

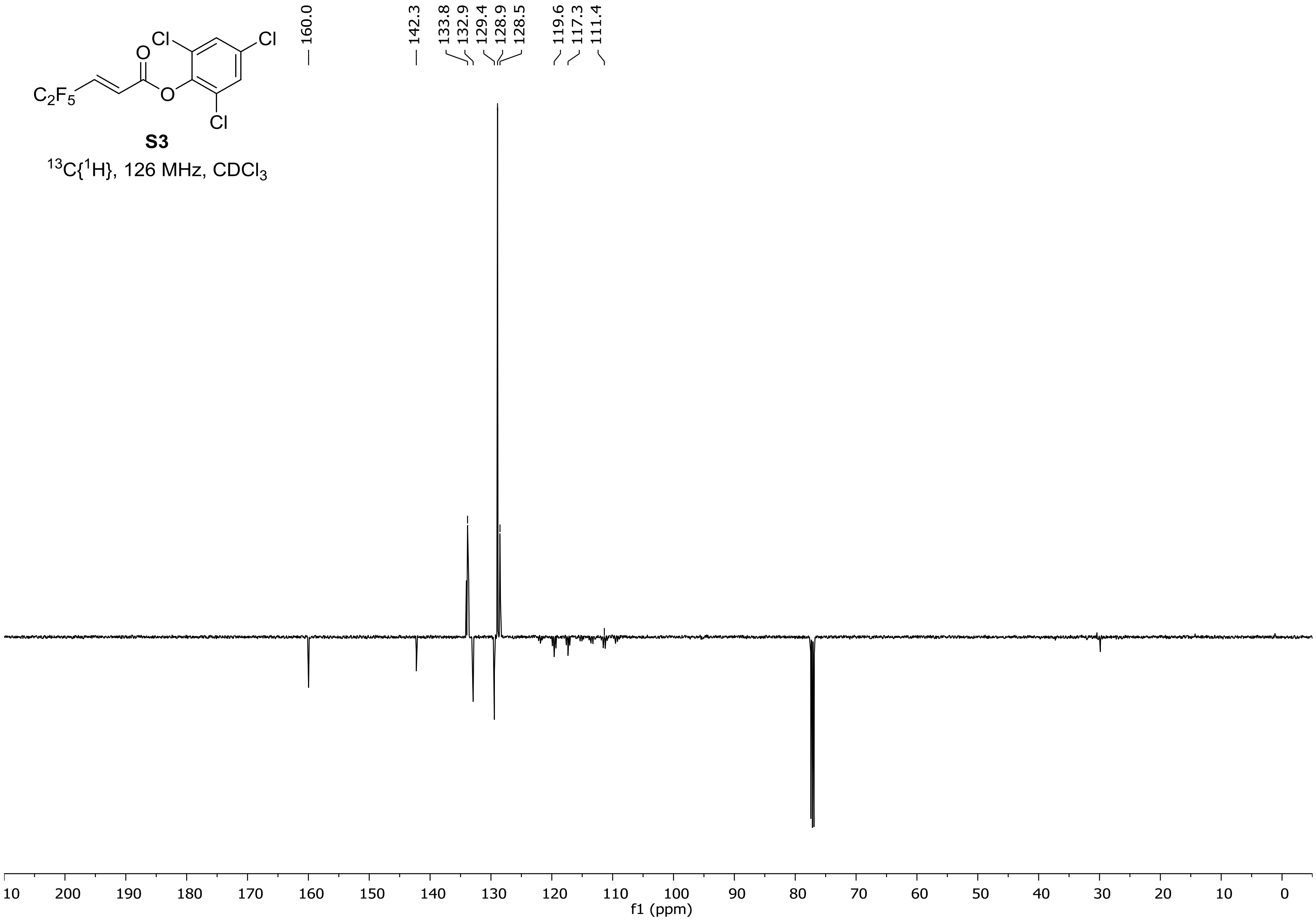

C2F5
O
O
Cl
Cl
Cl
S3
13C{1H}, 126 MHz, CDCl3
160.0
142.3
133.8
132.9
129.4
128.9
128.5
119.6
117.3
111.4
f1 (ppm)

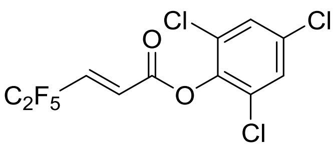

S3

$^{19}F\{^{1}H\}$, 376 MHz, $CDCl_3$

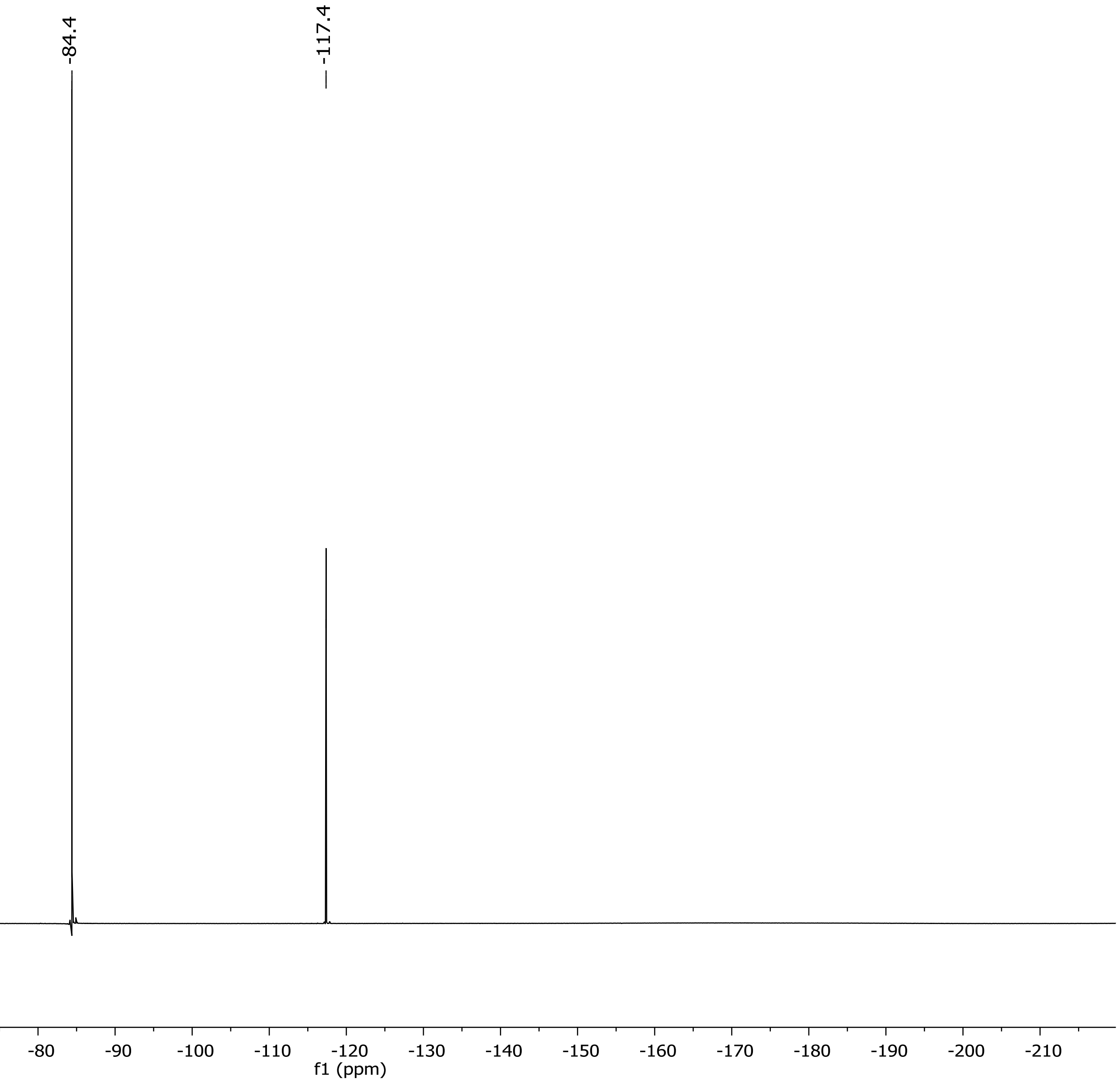

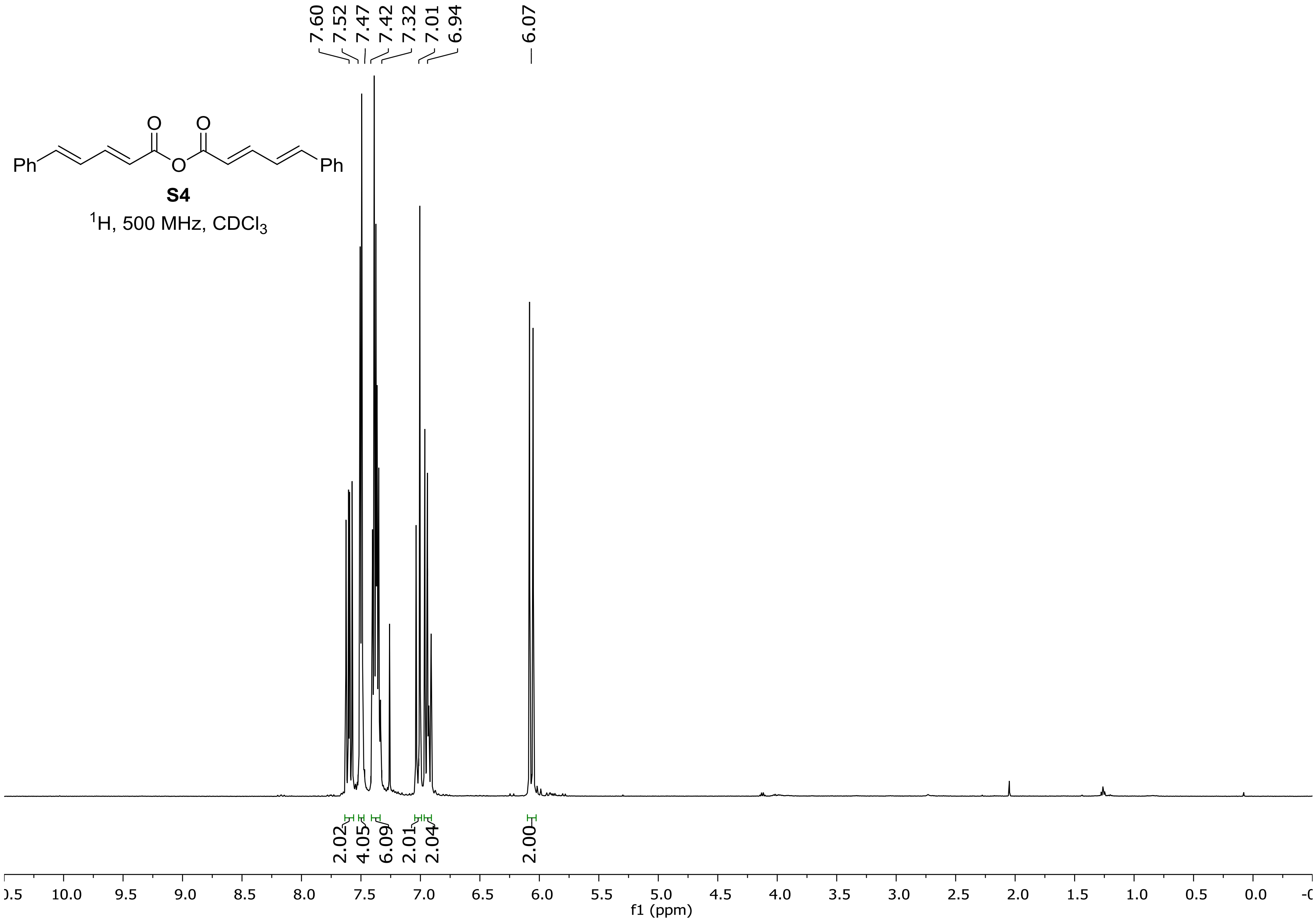

Ph
O
O
O
Ph
S4
1H, 500 MHz, CDCl3
7.60
7.52
7.47
7.42
7.32
7.01
6.94
6.07
2.02
4.05
6.09
2.01
2.04
2.00
f1 (ppm)

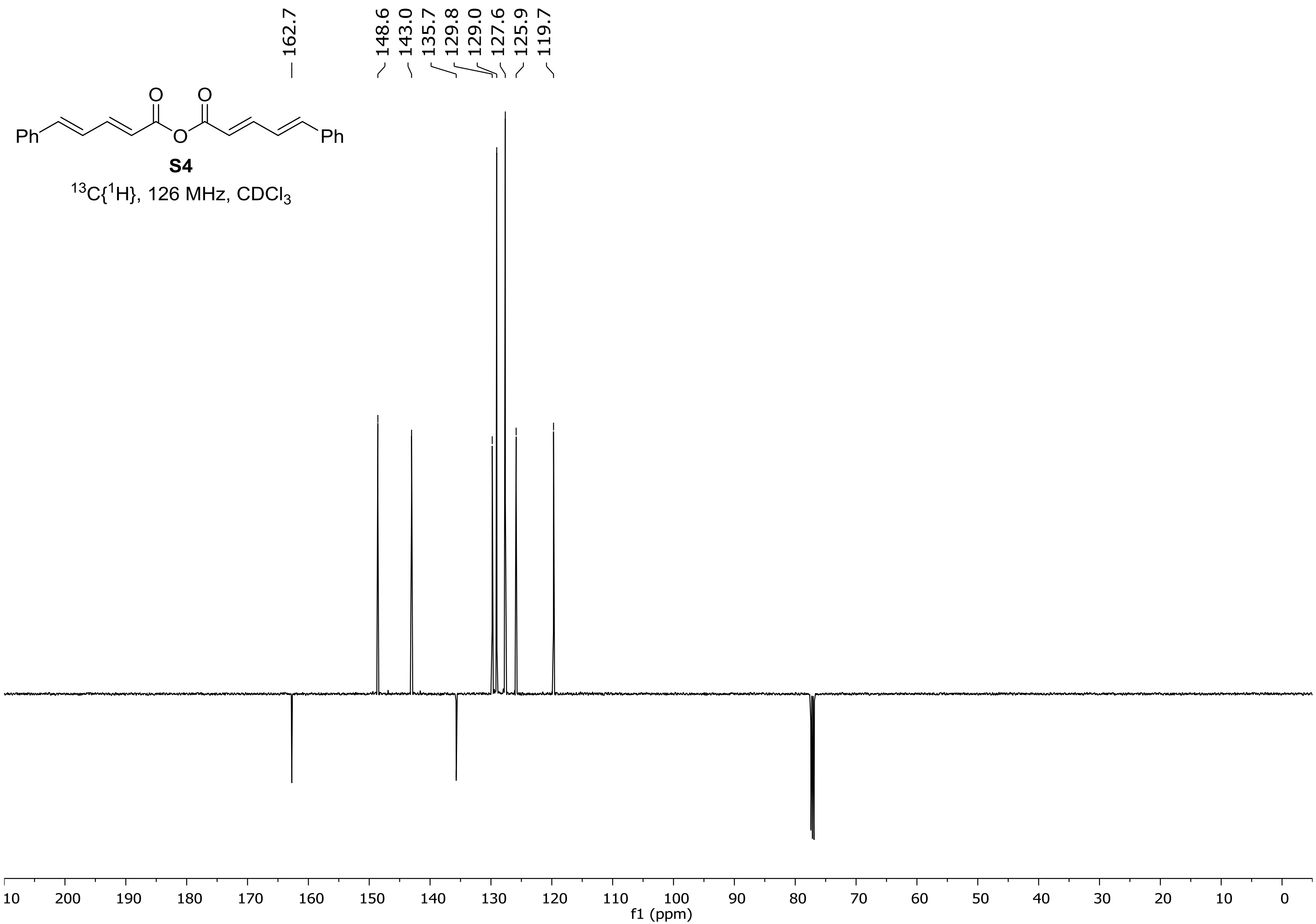

162.7
148.6
143.0
135.7
129.8
129.0
127.6
125.9
119.7
Ph
O
O
O
Ph
S4
13C{1H}, 126 MHz, CDCl3
10
200
190
180
170
160
150
140
130
120
110
100
90
80
70
60
50
40
30
20
10
0
f1 (ppm)

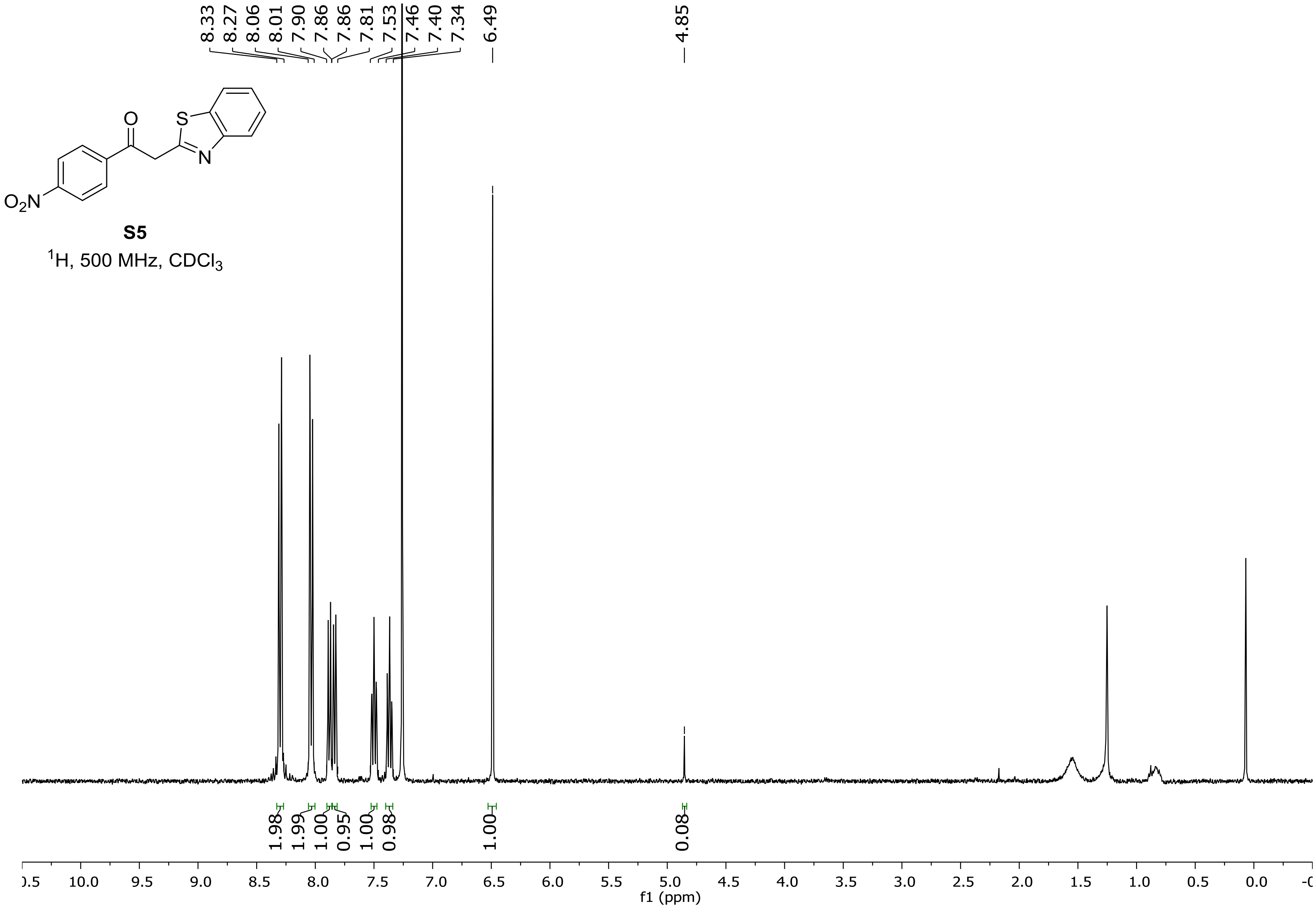

S5

^{1}H, 500 MHz, $CDCl_3$

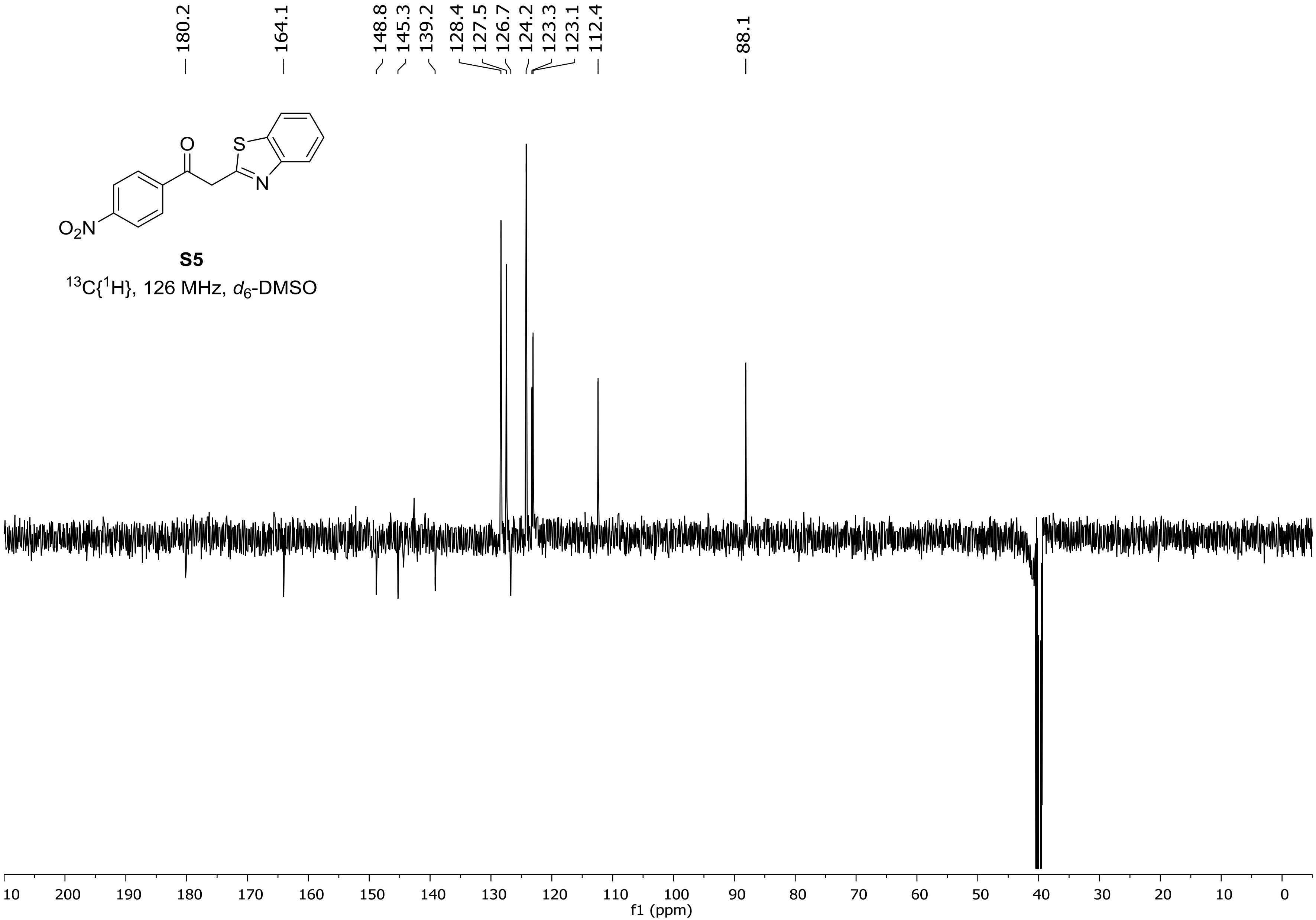

S5

$^{13}C\{^1H\}$, 126 MHz, d_6-DMSO

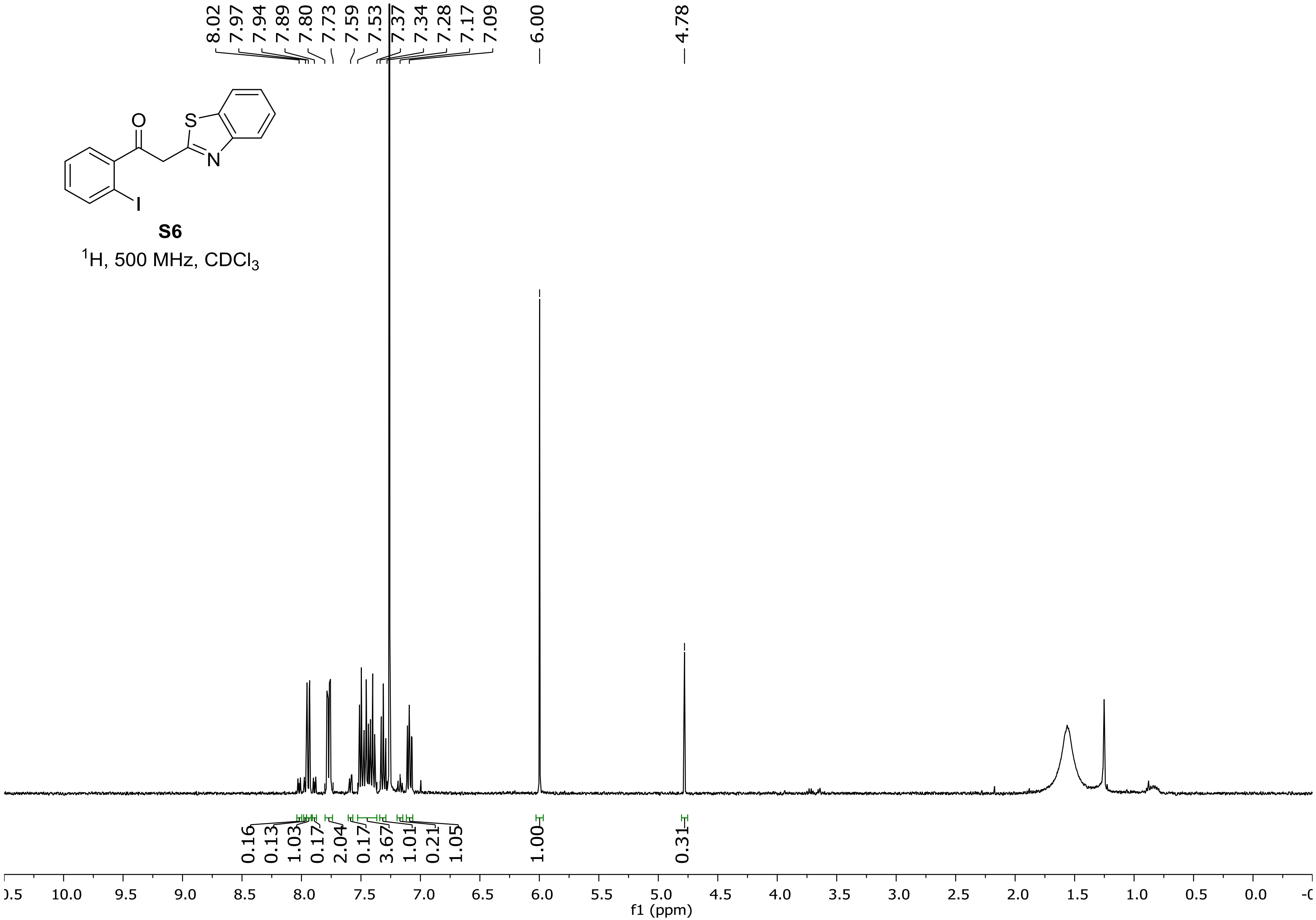
8.02
7.97
7.94
7.89
7.80
7.73
7.59
7.53
7.37
7.34
7.28
7.17
7.09
6.00
4.78
O
S
N
I
S6
¹H, 500 MHz, CDCl3
0.16
0.13
1.03
0.17
2.04
0.17
3.67
1.01
0.21
1.05
1.00
0.31
f1 (ppm)

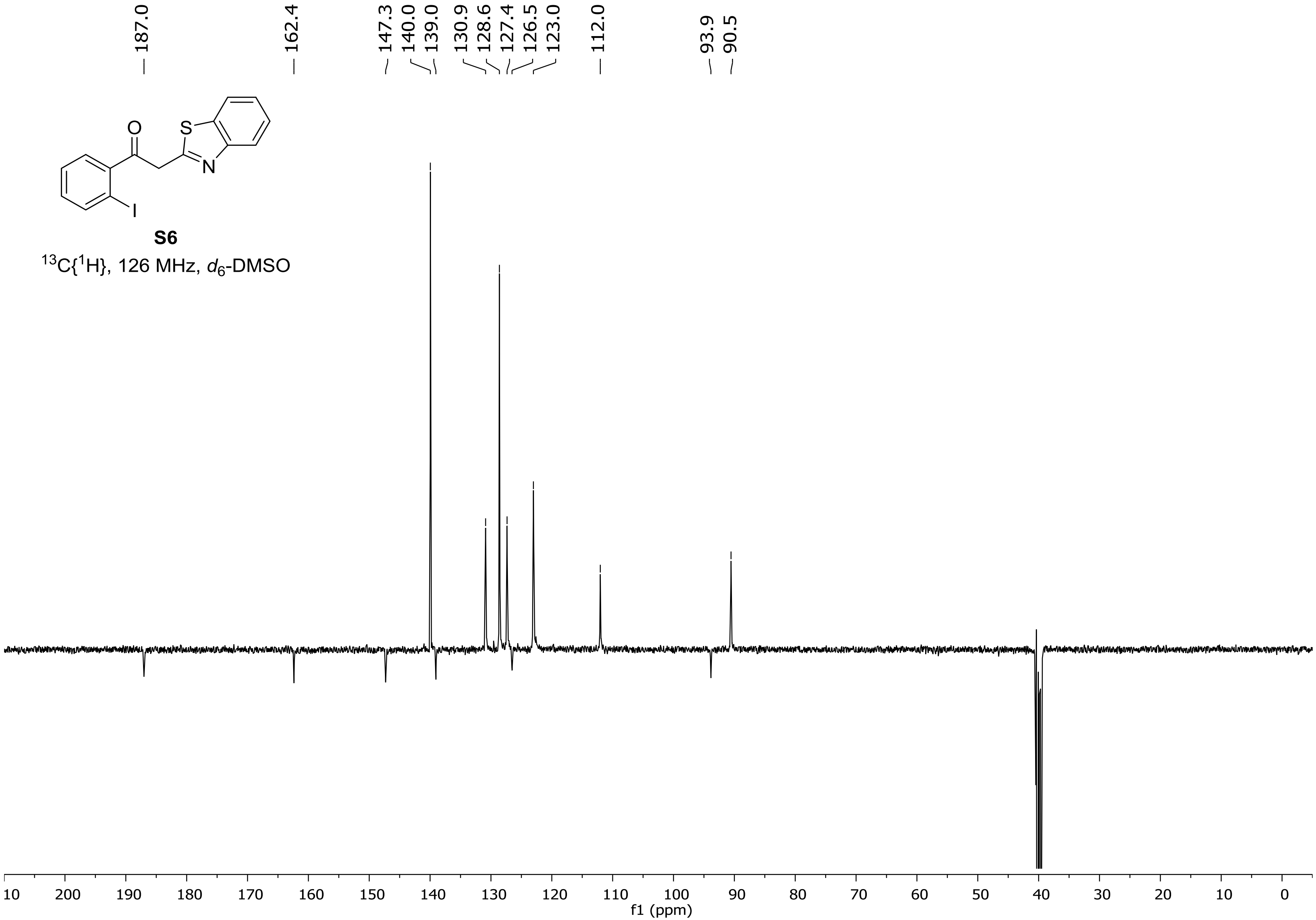
187.0
162.4
147.3
140.0
139.0
130.9
128.6
127.4
126.5
123.0
112.0
93.9
90.5
S6
$^{13}C\{^{1}H\}$, 126 MHz, d_6-DMSO
10 200 190 180 170 160 150 140 130 120 110 100 90 80 70 60 50 40 30 20 10 0
f1 (ppm)

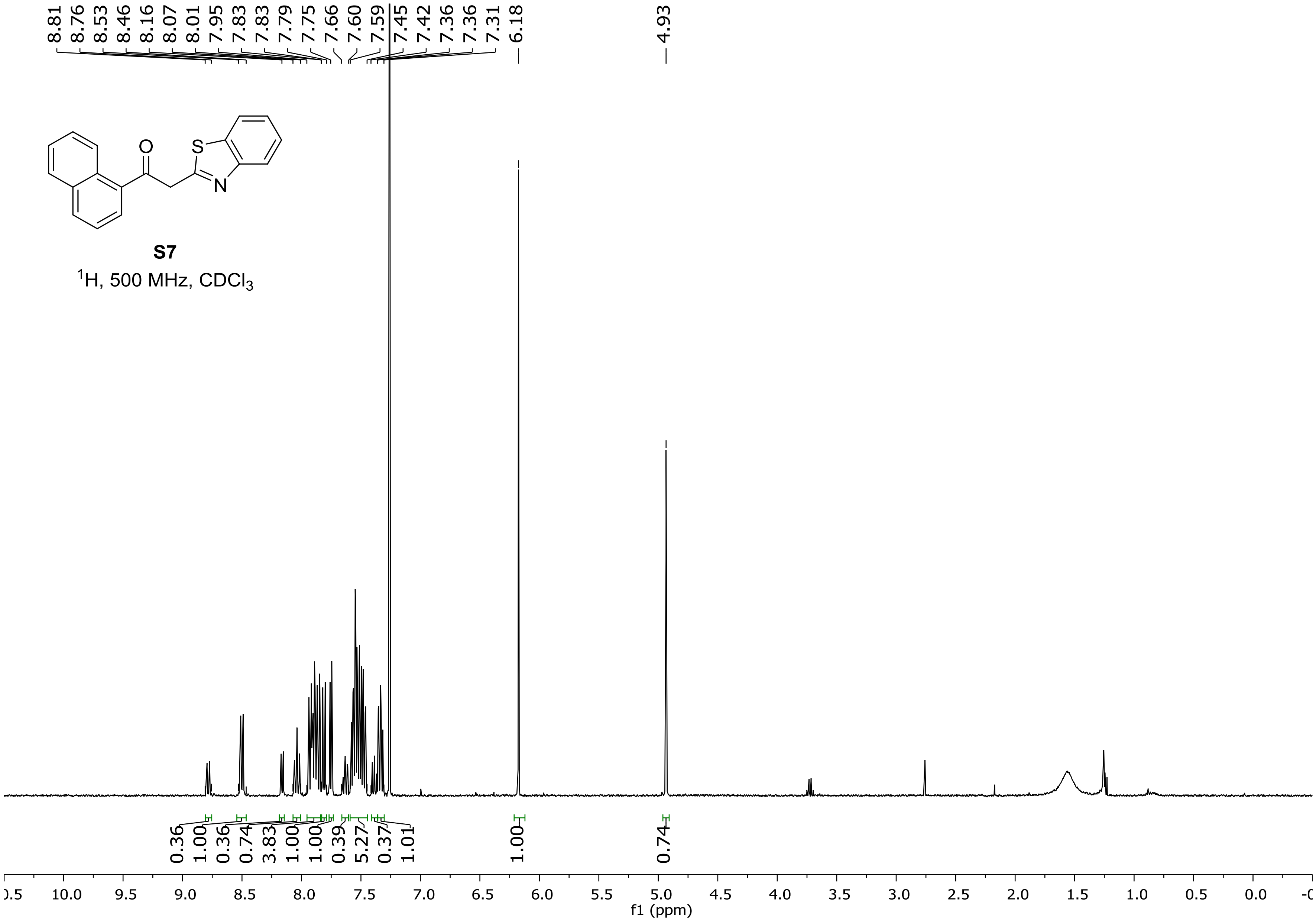

S7

^1H, 500 MHz, $CDCl_3$

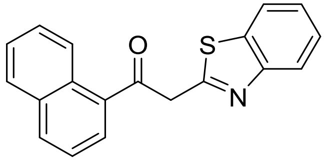

S7

$^{13}C\{^1H\}$, 126 MHz, $CDCl_3$

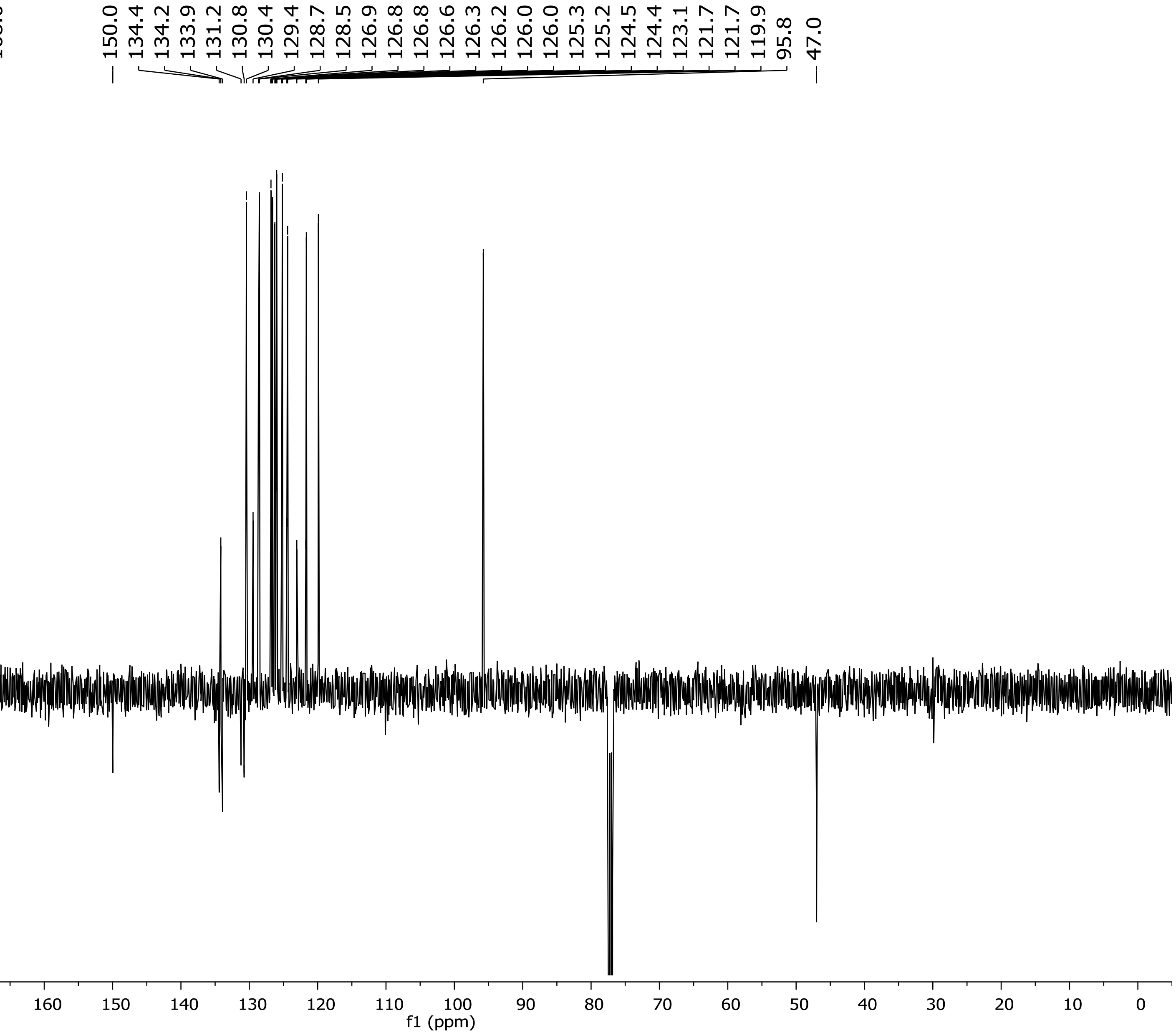

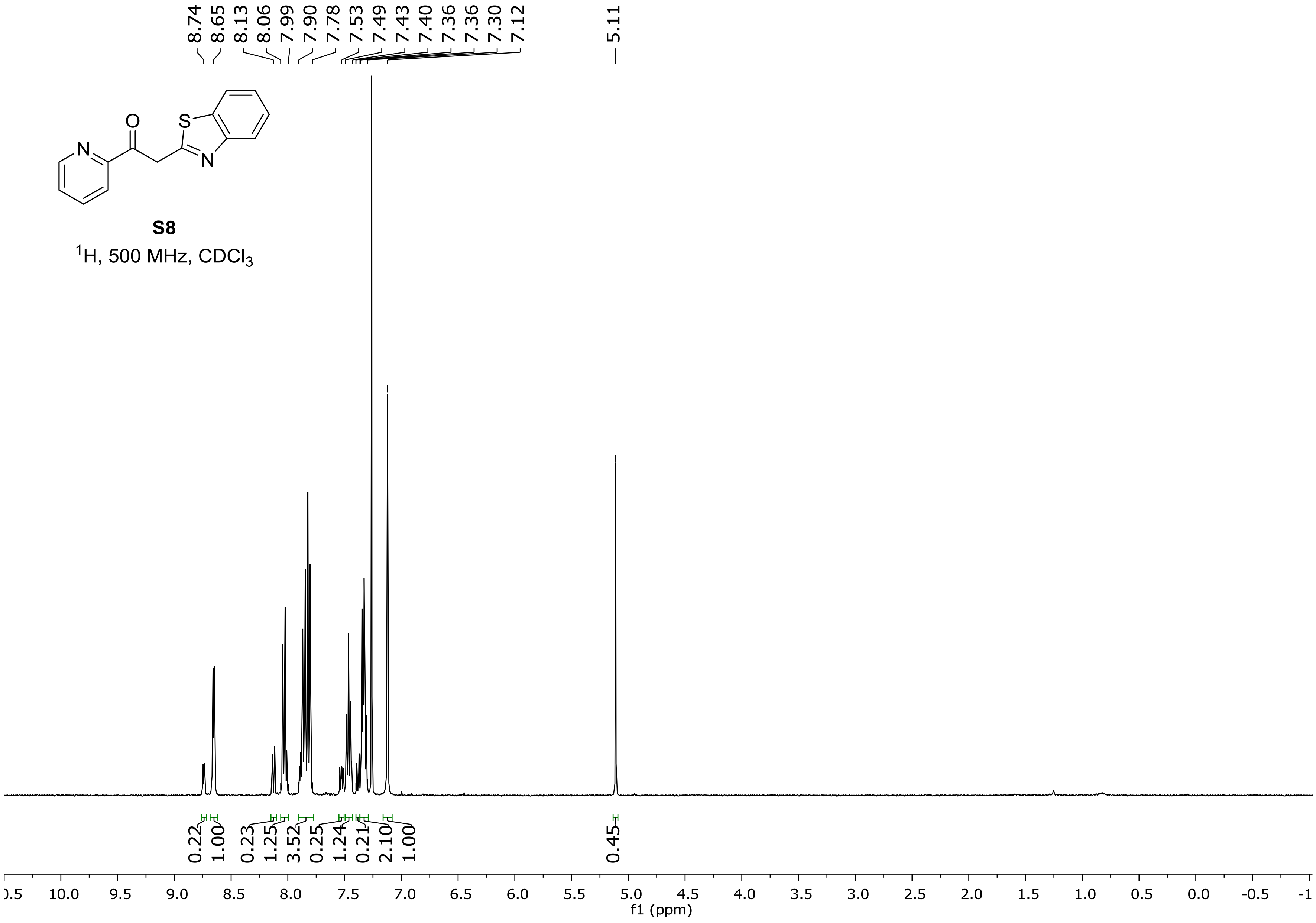
8.74
8.65
8.13
8.06
7.99
7.90
7.78
7.53
7.49
7.43
7.40
7.36
7.36
7.30
7.12
5.11
O
S
N
N
S8
^{1}H, 500 MHz, CDCl$_3$
0.22
1.00
0.23
1.25
3.52
0.25
1.24
0.21
2.10
1.00
0.45
f1 (ppm)

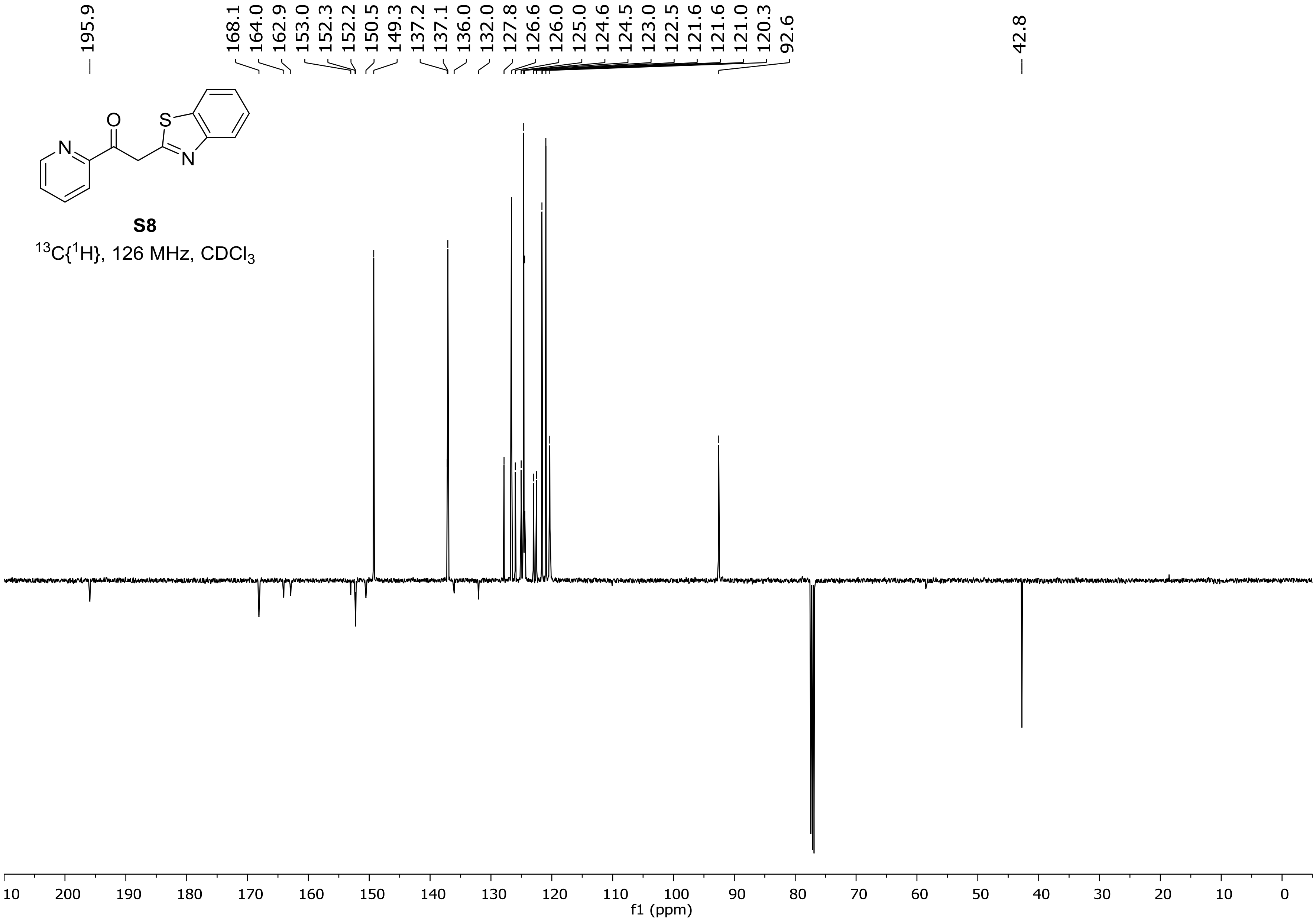
195.9
168.1
164.0
162.9
153.0
152.3
152.2
150.5
149.3
137.2
137.1
136.0
132.0
127.8
126.6
126.0
125.0
124.6
124.5
123.0
122.5
121.6
121.6
121.0
120.3
92.6
42.8
O
S
N
N
S8
13C{1H}, 126 MHz, CDCl3
10 200 190 180 170 160 150 140 130 120 110 100 90 80 70 60 50 40 30 20 10 0
f1 (ppm)

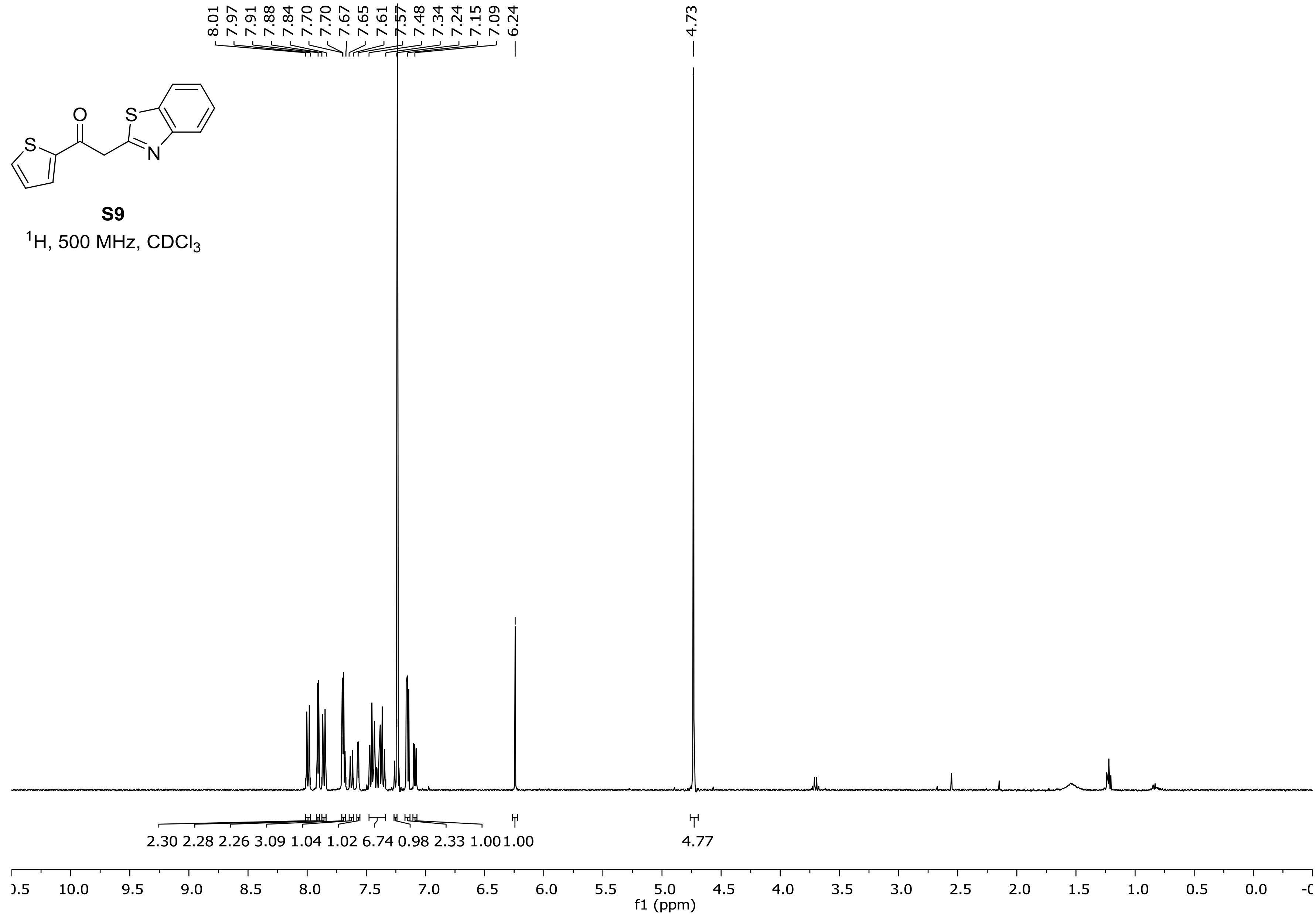

S9

^{1}H, 500 MHz, $CDCl_3$

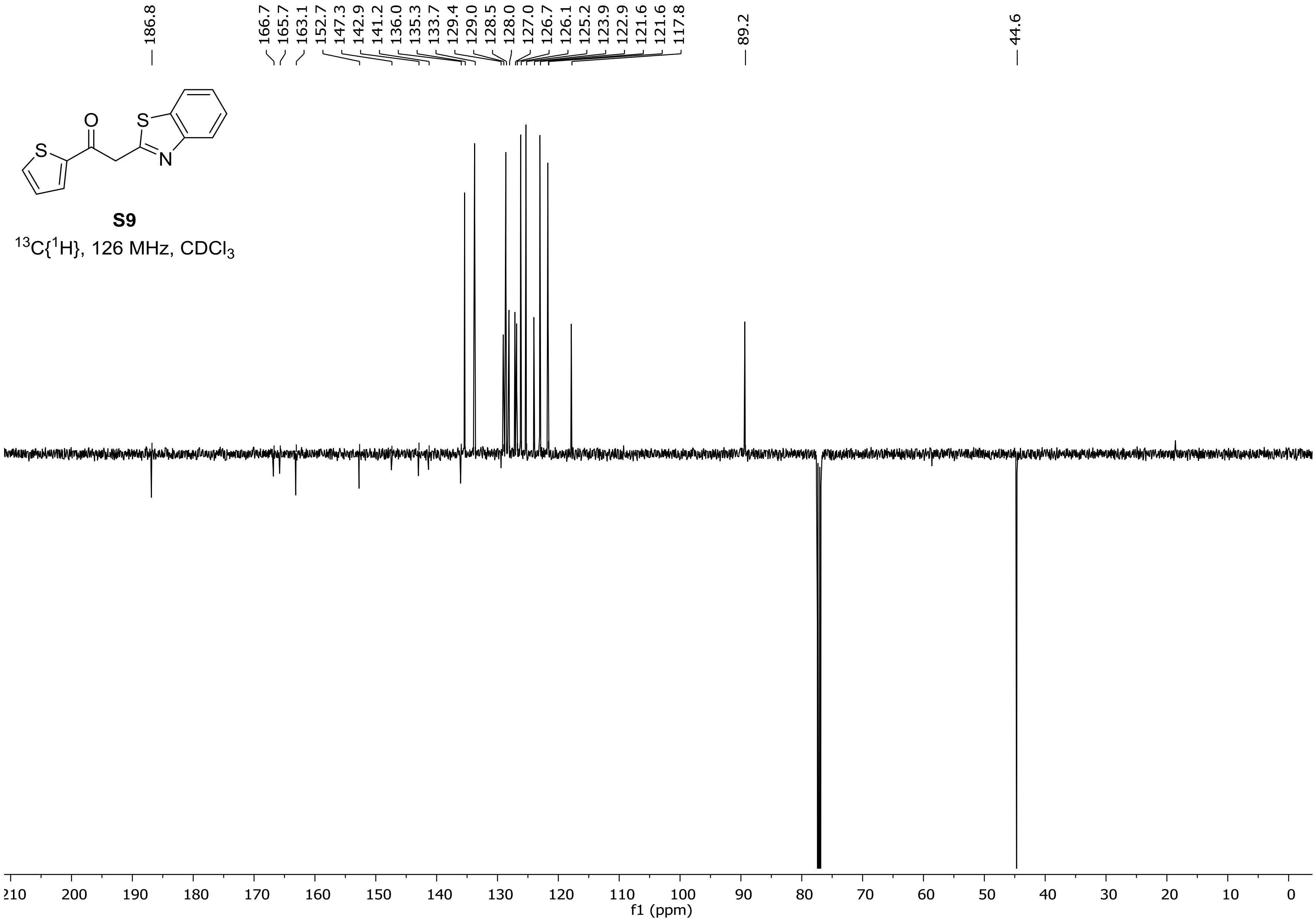

S9

$^{13}C\{^1H\}$, 126 MHz, $CDCl_3$

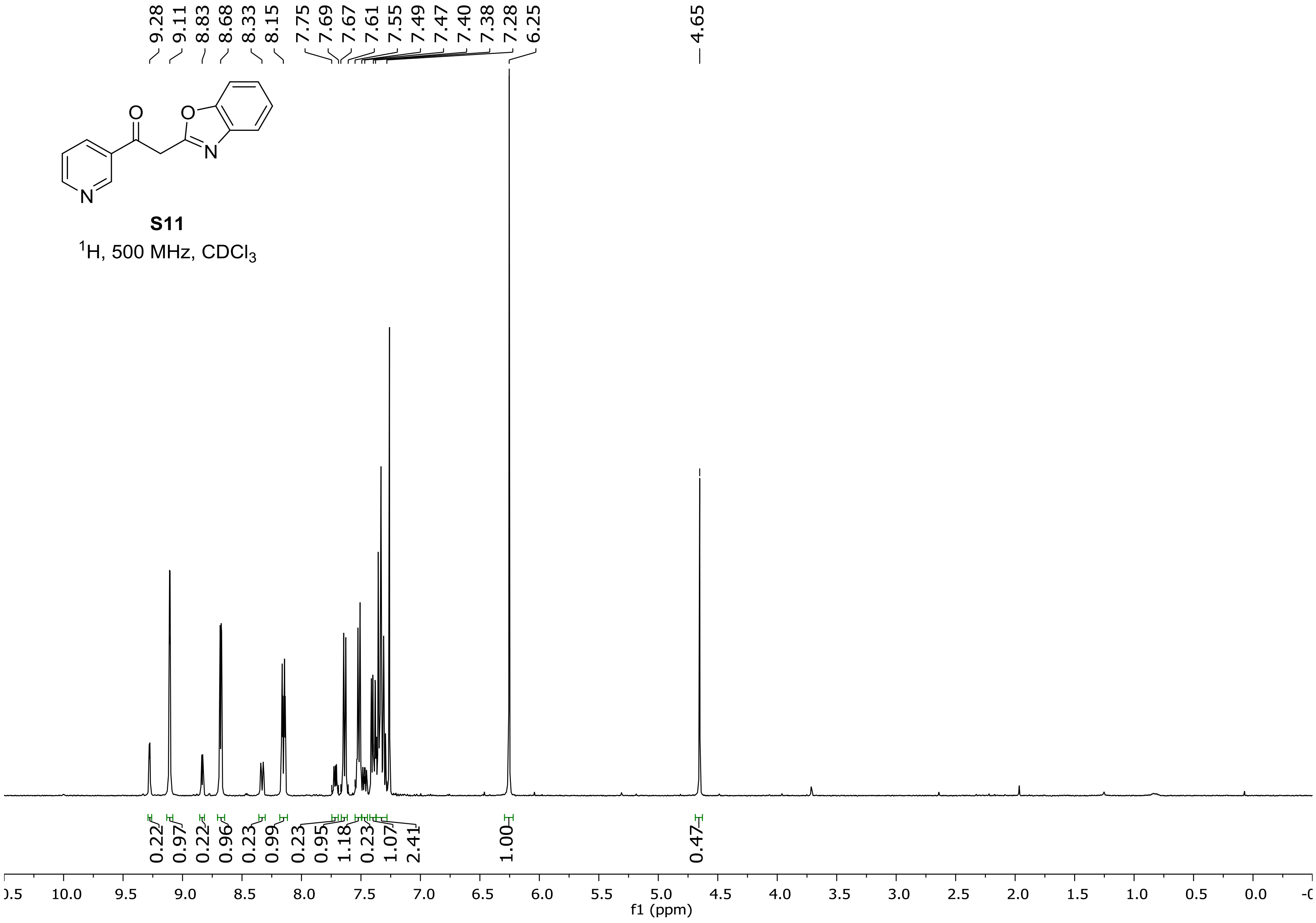

9.28
9.11
8.83
8.68
8.33
8.15
7.75
7.69
7.67
7.61
7.55
7.49
7.47
7.40
7.38
7.28
6.25
4.65
O
O
N
N
S11
¹H, 500 MHz, CDCl₃
0.22
0.97
0.22
0.96
0.23
0.99
0.23
0.95
1.18
0.23
1.07
2.41
1.00
0.47
f1 (ppm)

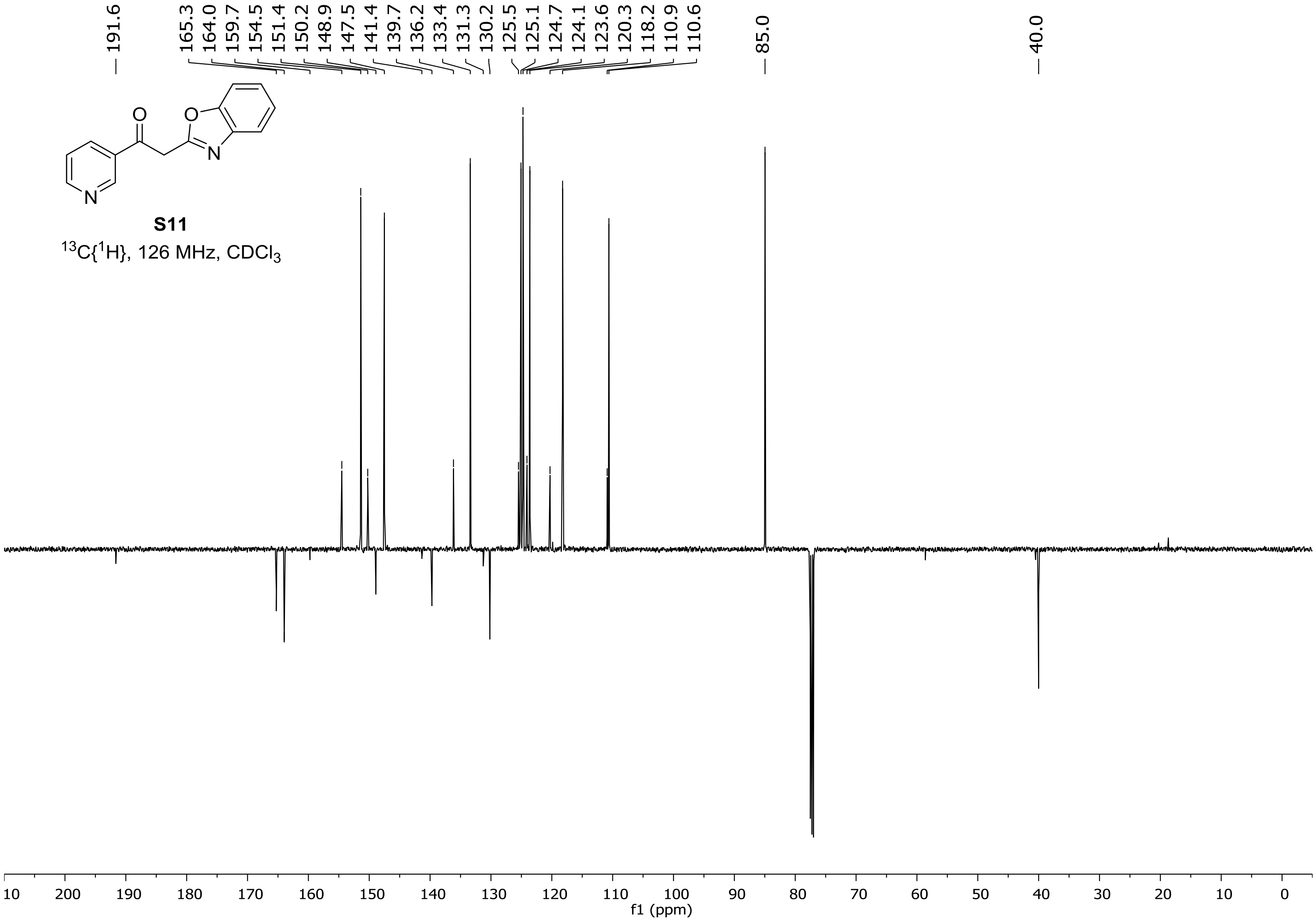

191.6
165.3
164.0
159.7
154.5
151.4
150.2
148.9
147.5
141.4
139.7
136.2
133.4
131.3
130.2
125.5
125.1
124.7
124.1
123.6
120.3
118.2
110.9
110.6
85.0
40.0
O
O
N
N
S11
$^{13}C\{^1H\}$, 126 MHz, $CDCl_3$
10 200 190 180 170 160 150 140 130 120 110 100 90 80 70 60 50 40 30 20 10 0
f1 (ppm)

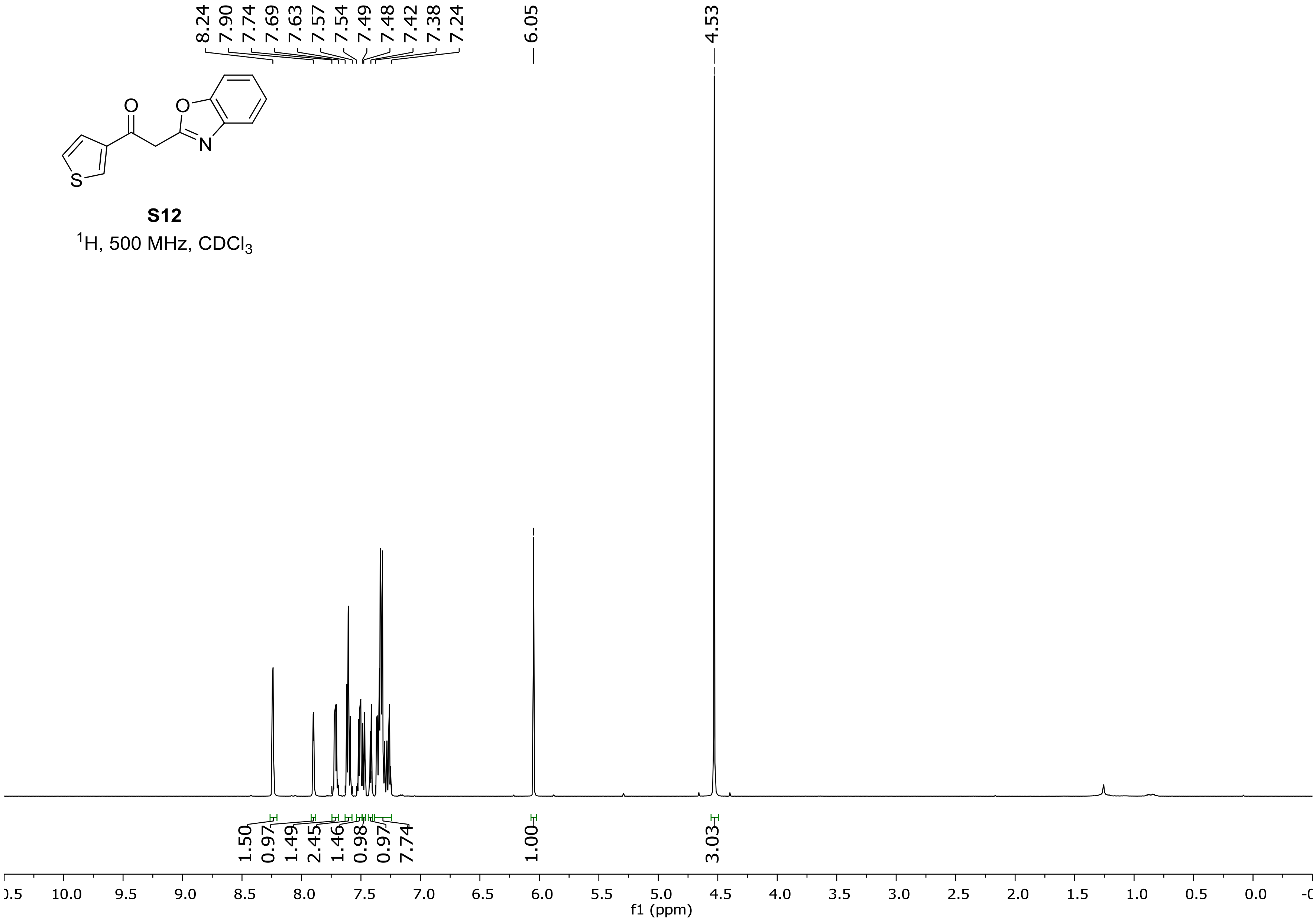

8.24
7.90
7.74
7.69
7.63
7.57
7.54
7.49
7.48
7.42
7.38
7.24
6.05
4.53
S12
1H, 500 MHz, CDCl3
1.50
0.97
1.49
2.45
1.46
0.98
0.97
7.74
1.00
3.03
f1 (ppm)

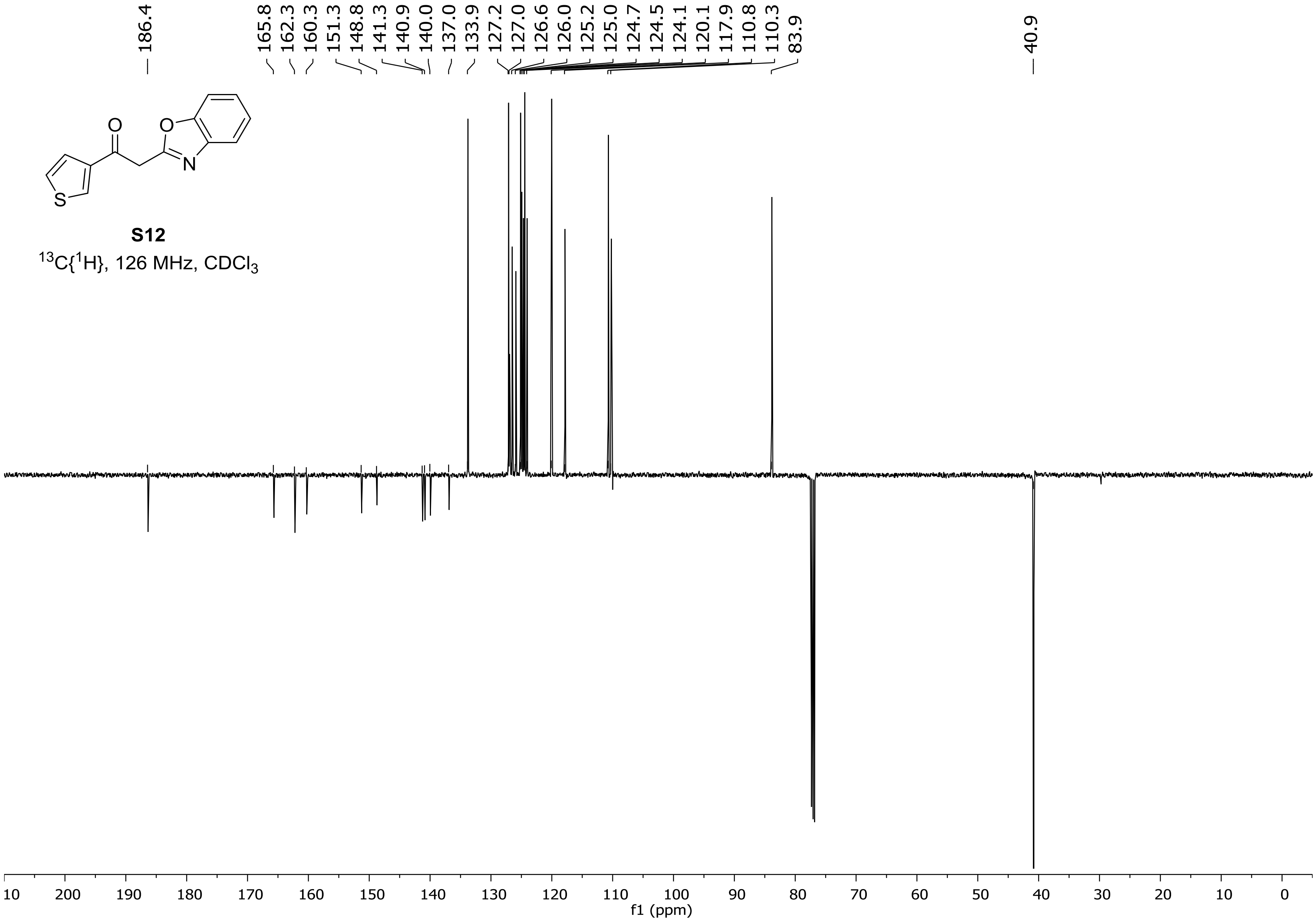

S12

$^{13}C\{^{1}H\}$, 126 MHz, $CDCl_3$

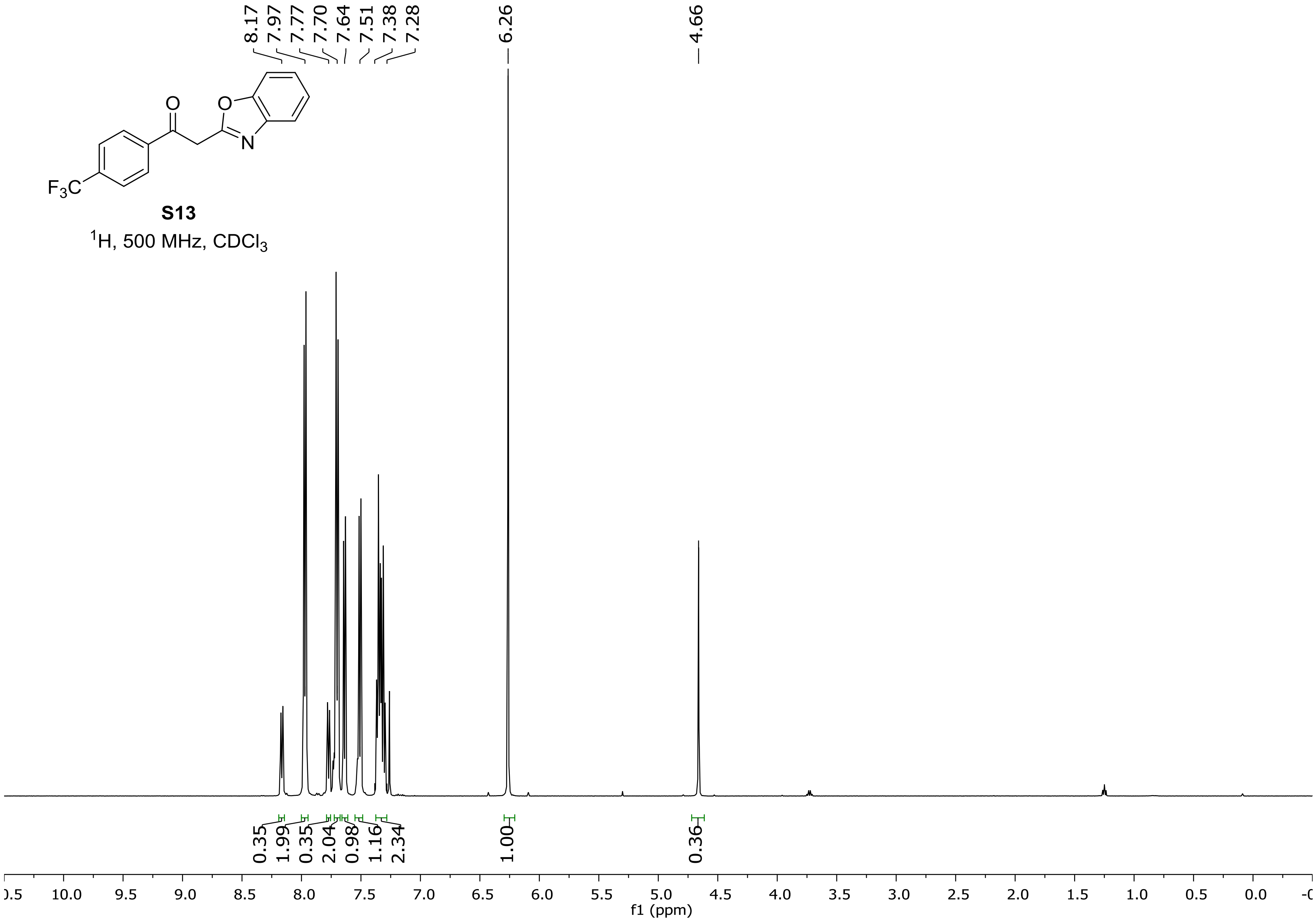
8.17
7.97
7.77
7.70
7.64
7.51
7.38
7.28
6.26
4.66
O
O
N
F3C
S13
1H, 500 MHz, CDCl3
0.35
1.99
0.35
2.04
0.98
1.16
2.34
1.00
0.36
10.0
9.5
9.0
8.5
8.0
7.5
7.0
6.5
6.0
5.5
5.0
4.5
4.0
3.5
3.0
2.5
2.0
1.5
1.0
0.5
0.0
f1 (ppm)

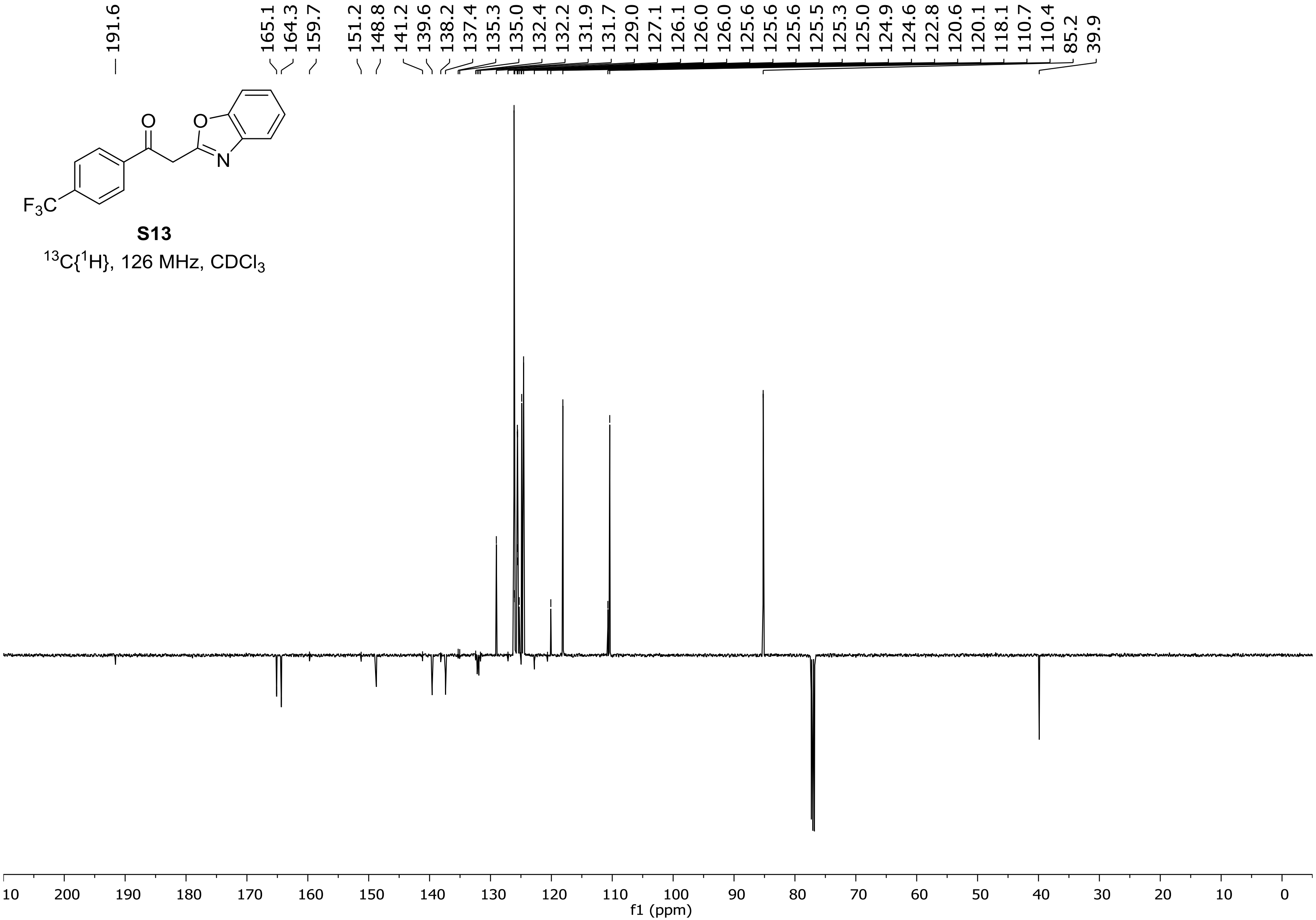
191.6
165.1
164.3
159.7
151.2
148.8
141.2
139.6
138.2
137.4
135.3
135.0
132.4
132.2
131.9
131.7
129.0
127.1
126.1
126.0
126.0
125.6
125.6
125.6
125.5
125.3
125.0
124.9
124.6
122.8
120.6
120.1
118.1
110.7
110.4
85.2
39.9
O
O
N
F3C
S13
13C{1H}, 126 MHz, CDCl3
10
200
190
180
170
160
150
140
130
120
110
100
90
80
70
60
50
40
30
20
10
0
f1 (ppm)

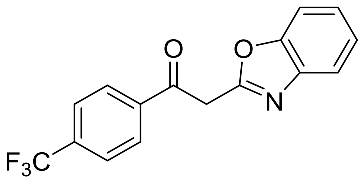

S13

$^{19}F\{^{1}H\}$, 376 MHz, $CDCl_3$

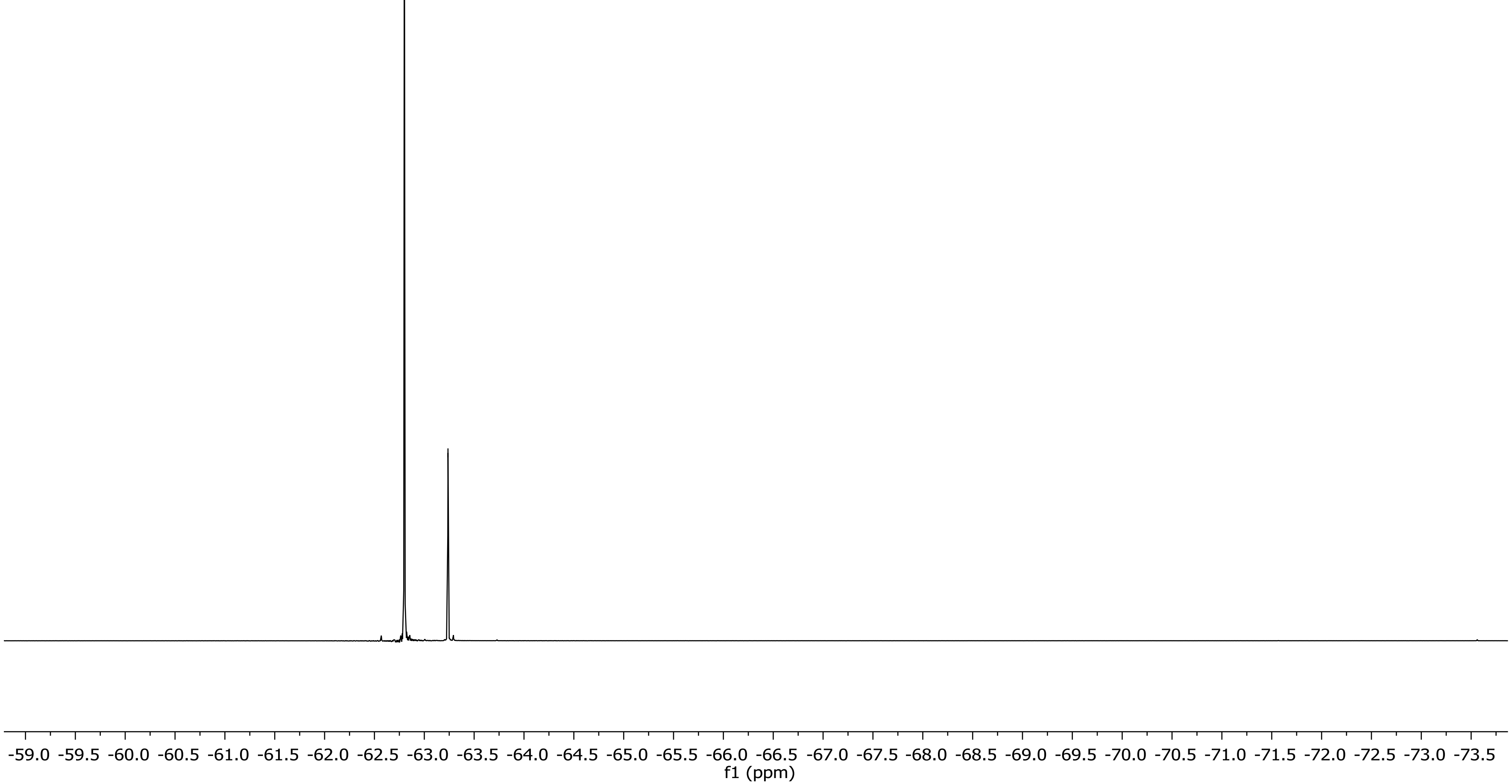

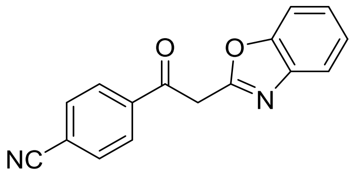

S14

^{1}H, 500 MHz, $CDCl_3$

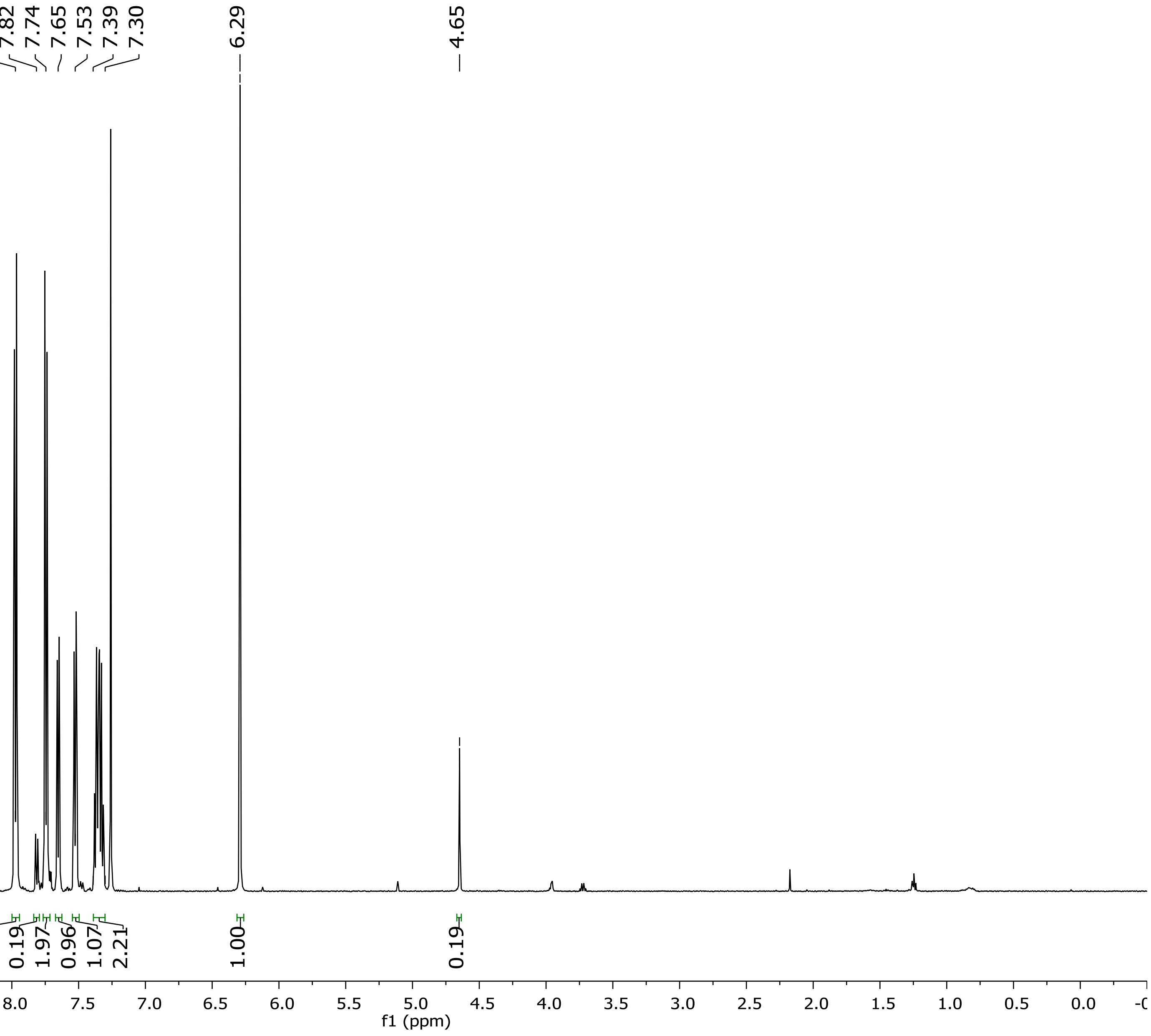

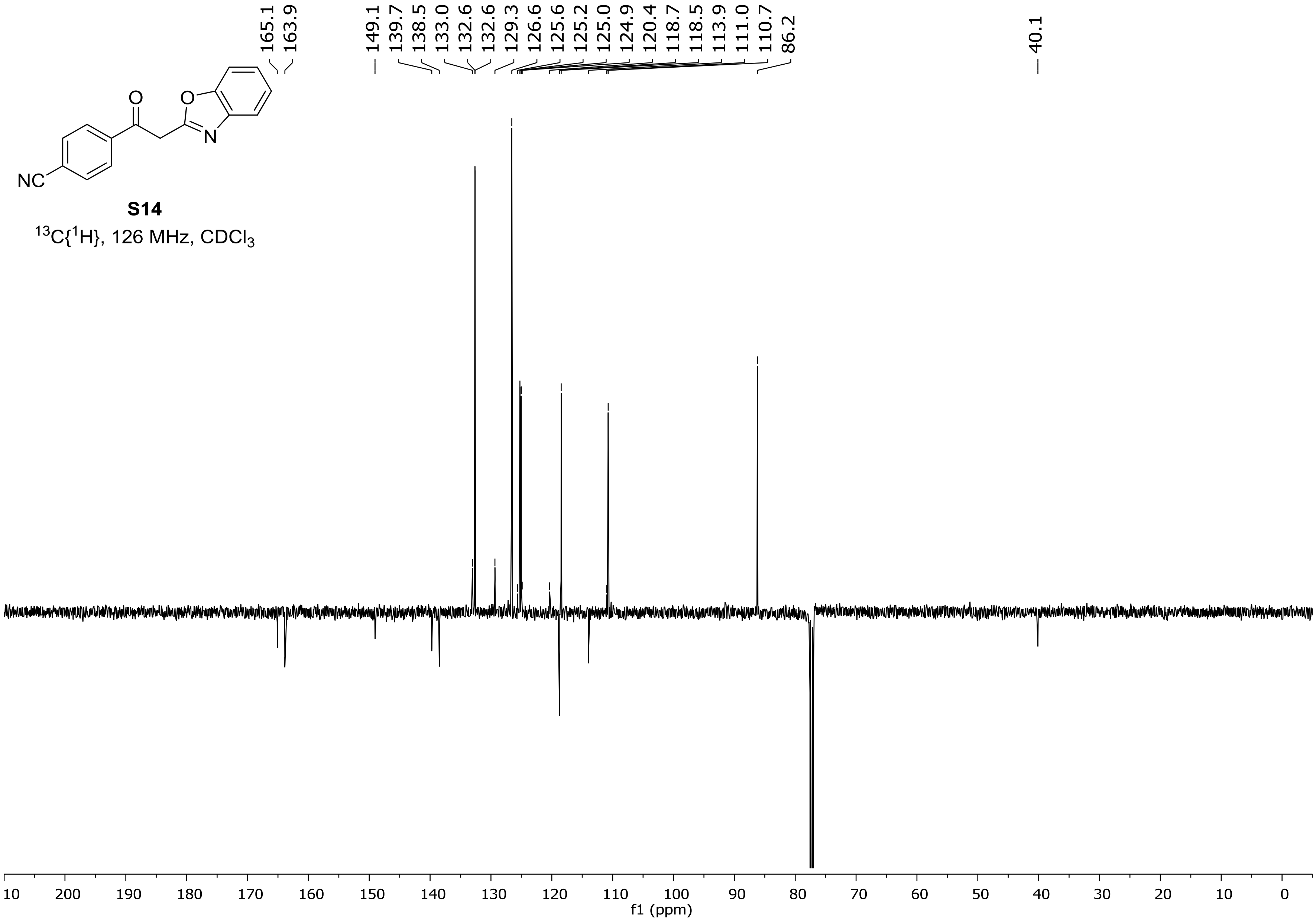
165.1
163.9
149.1
139.7
138.5
133.0
132.6
132.6
129.3
126.6
125.6
125.2
125.0
124.9
120.4
118.7
118.5
113.9
111.0
110.7
86.2
40.1
O
O
N
NC
S14
$^{13}C\{^1H\}$, 126 MHz, $CDCl_3$
10
200
190
180
170
160
150
140
130
120
110
100
90
80
70
60
50
40
30
20
10
0
f1 (ppm)

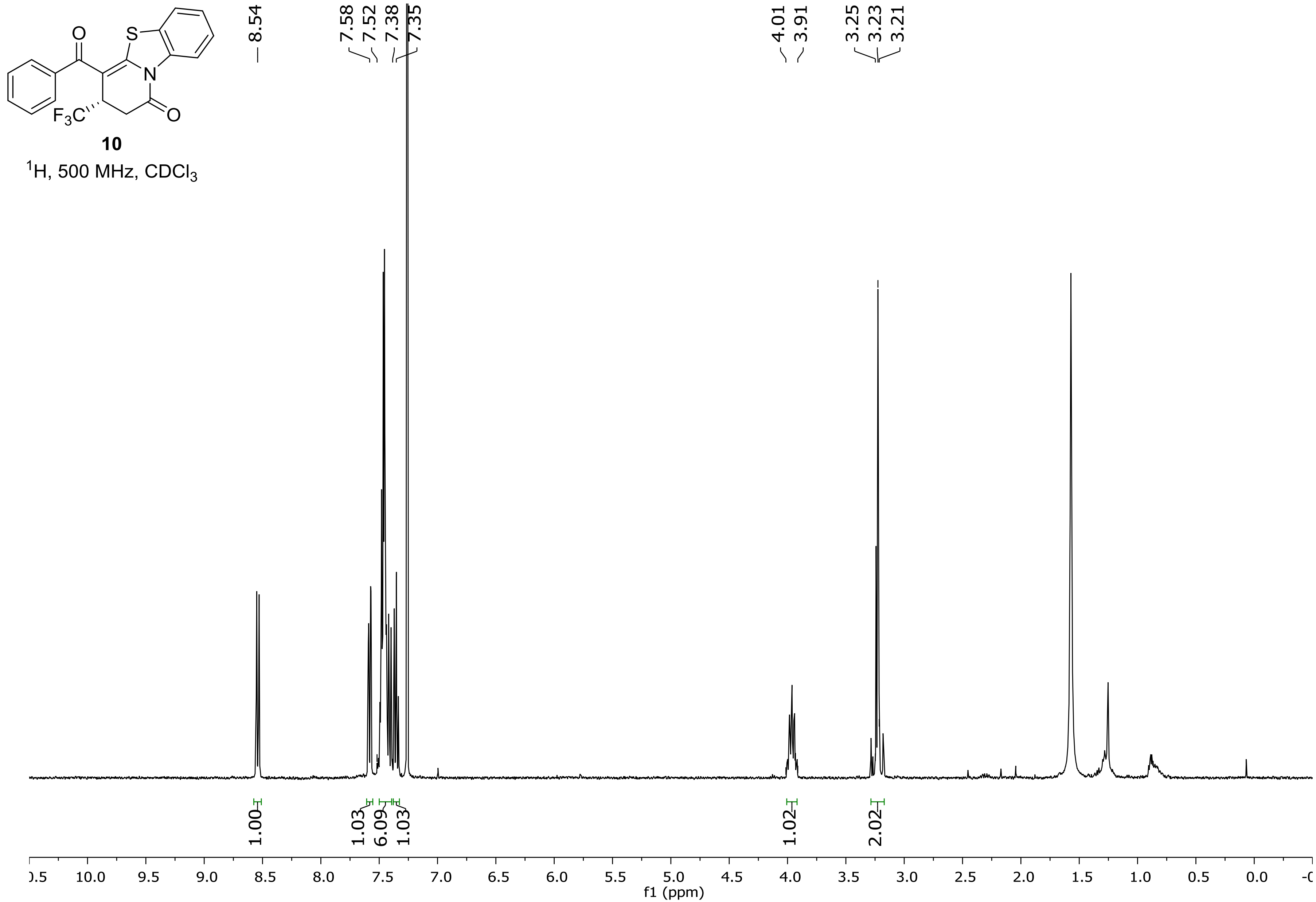

10
1H, 500 MHz, CDCl3
8.54
7.58
7.52
7.38
7.35
4.01
3.91
3.25
3.23
3.21
1.00
1.03
6.09
1.03
1.02
2.02
f1 (ppm)

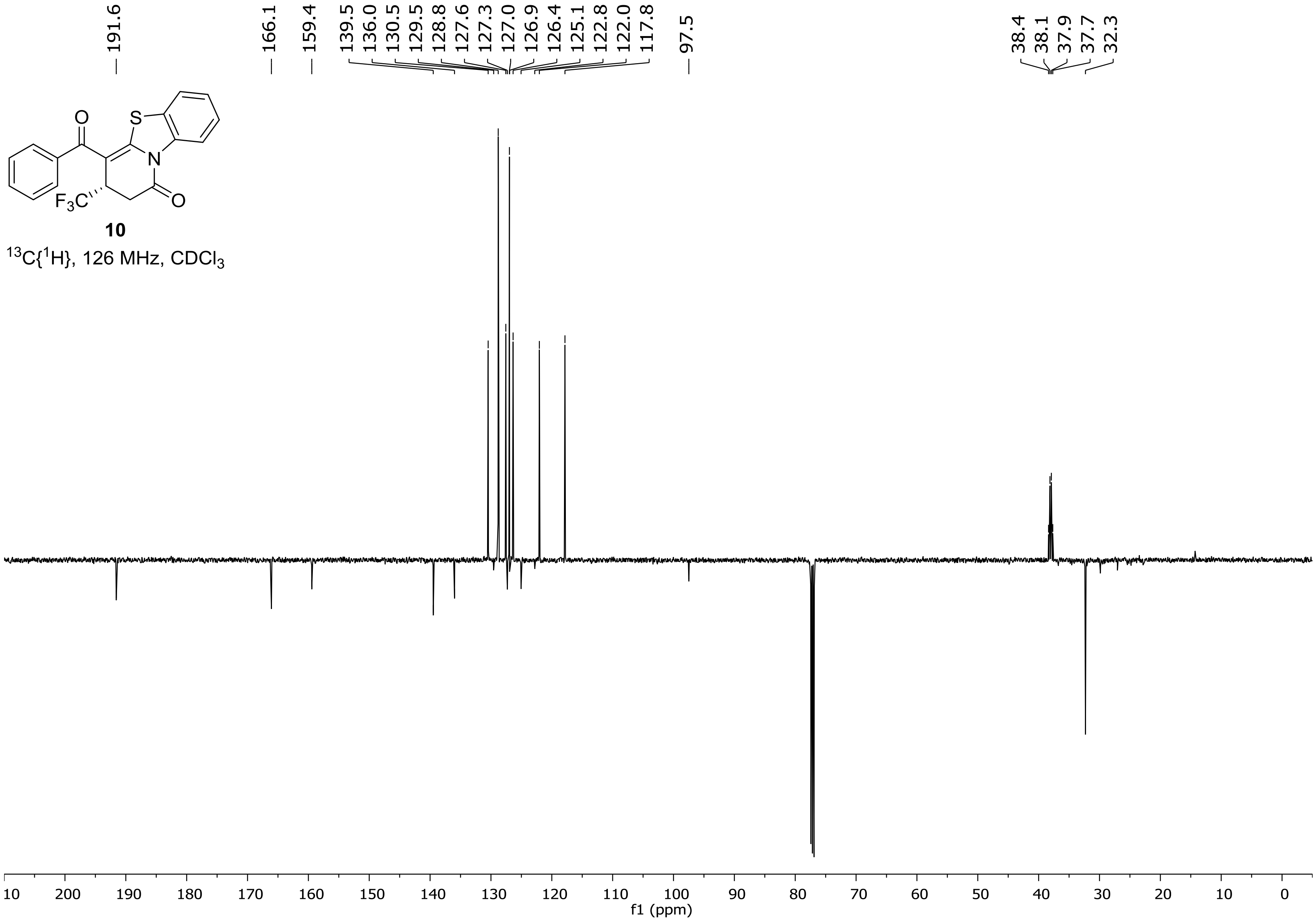

10

$^{13}C\{^{1}H\}$, 126 MHz, $CDCl_3$

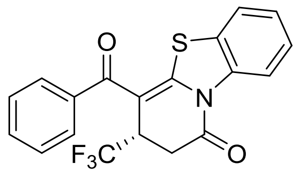

10

$^{19}F\{^1H\}$, 376 MHz, $CDCl_3$

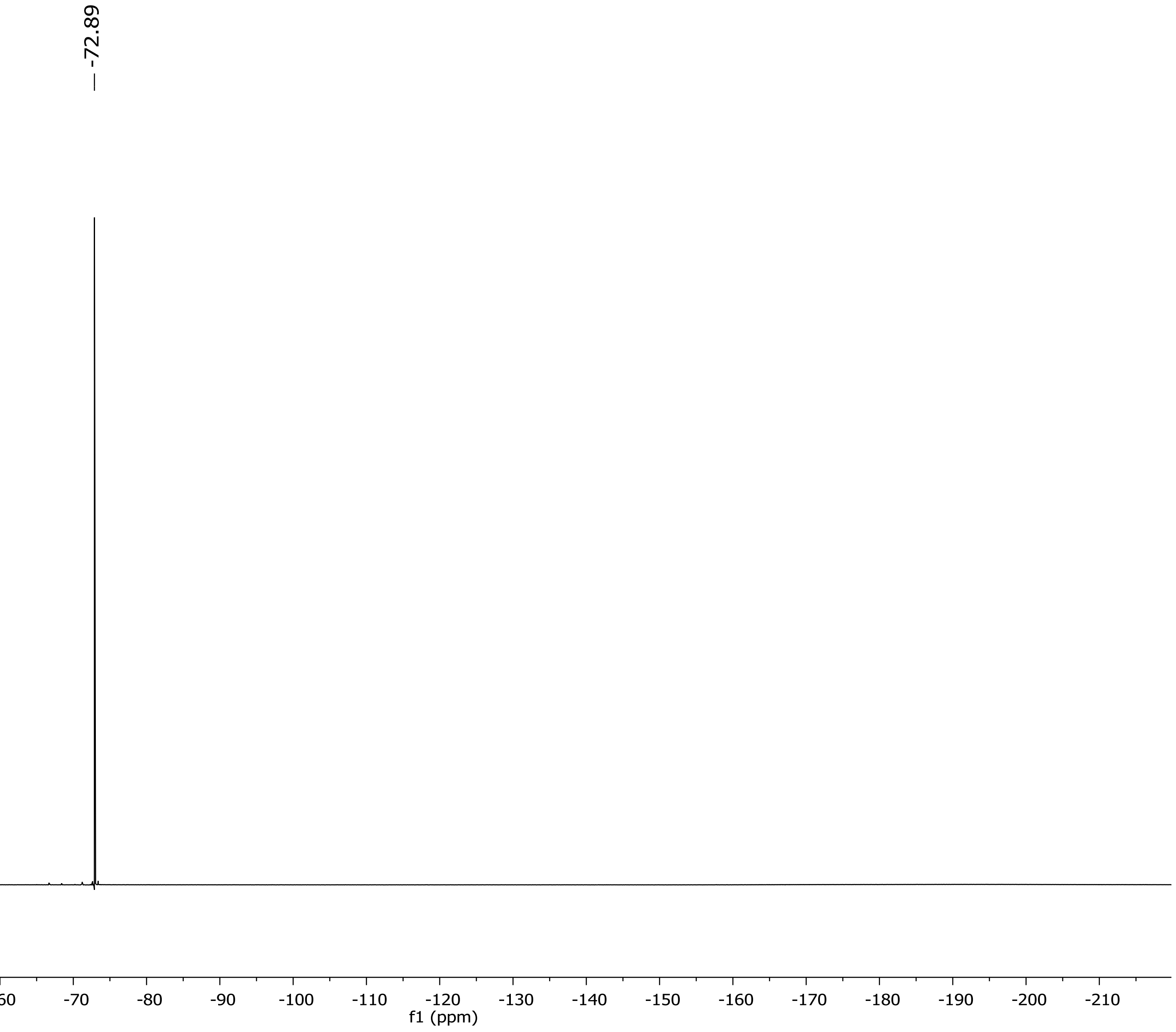

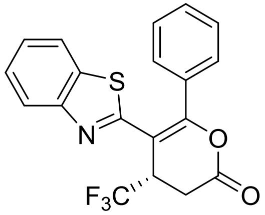

11

^1H, 500 MHz, $CDCl_3$

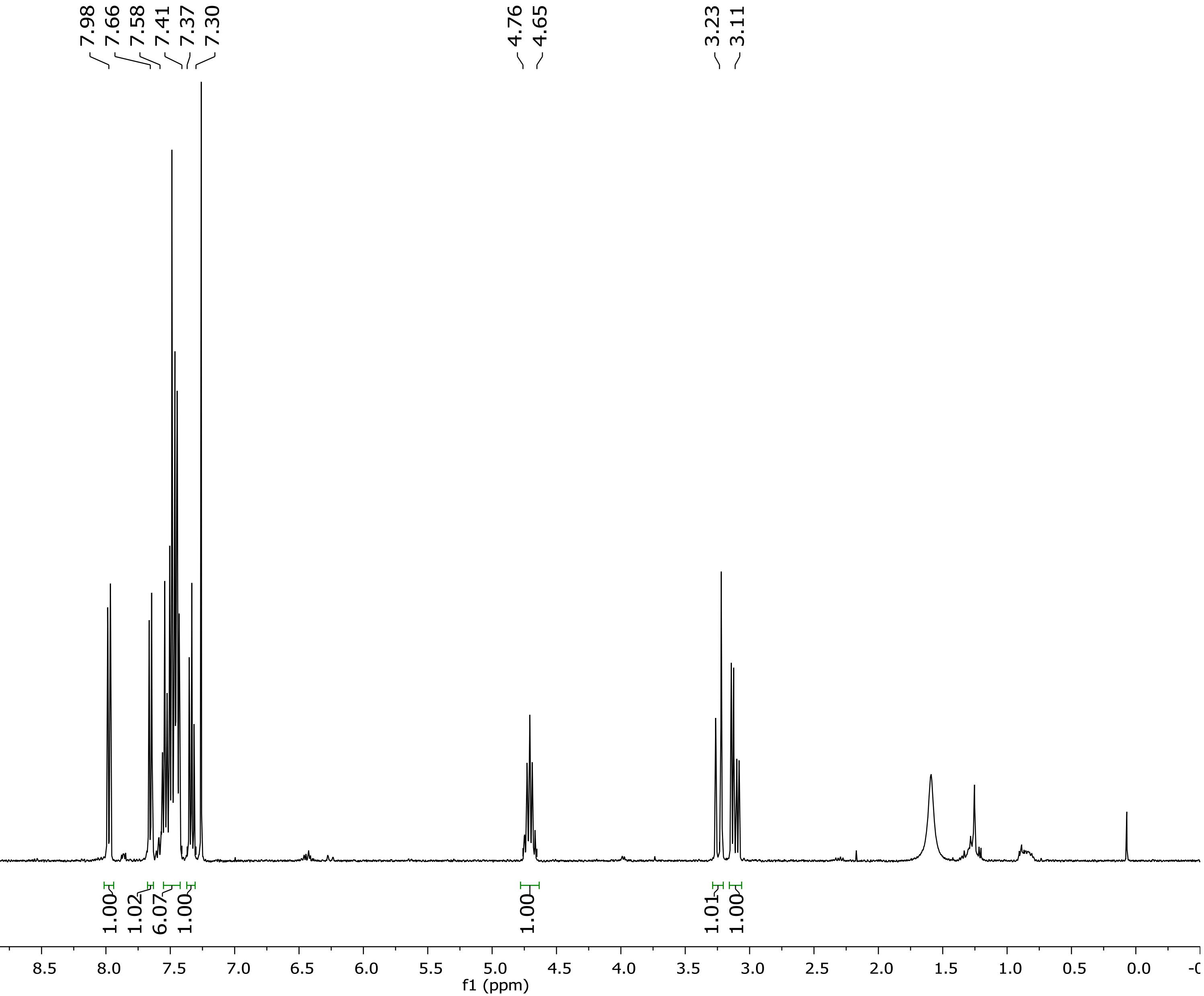

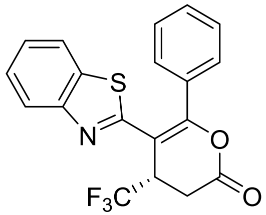

11

$^{13}C\{^{1}H\}$, 126 MHz, $CDCl_3$

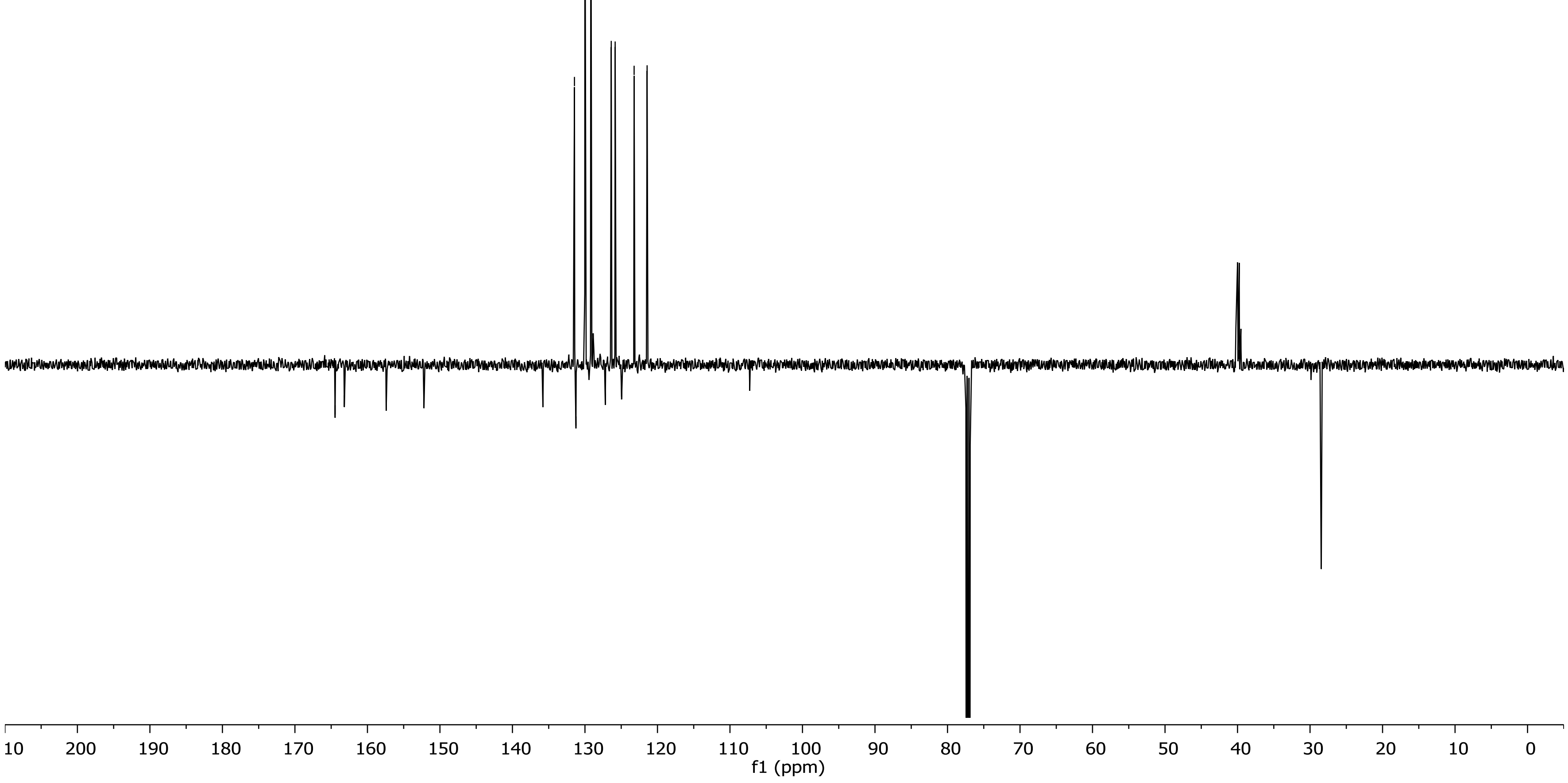

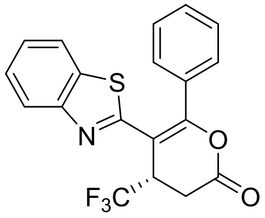

11

$^{19}F\{^{1}H\}$, 376 MHz, $CDCl_3$

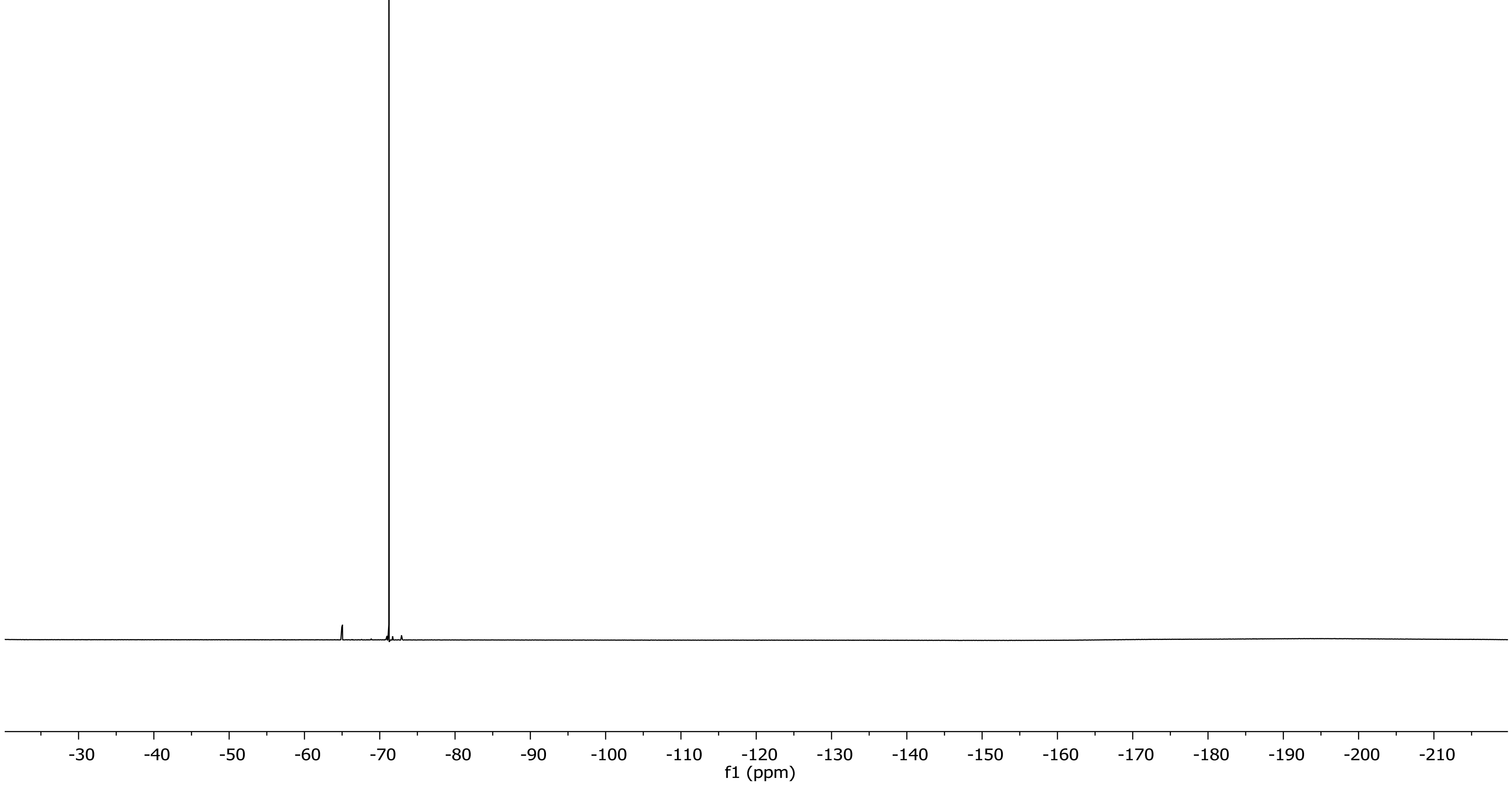

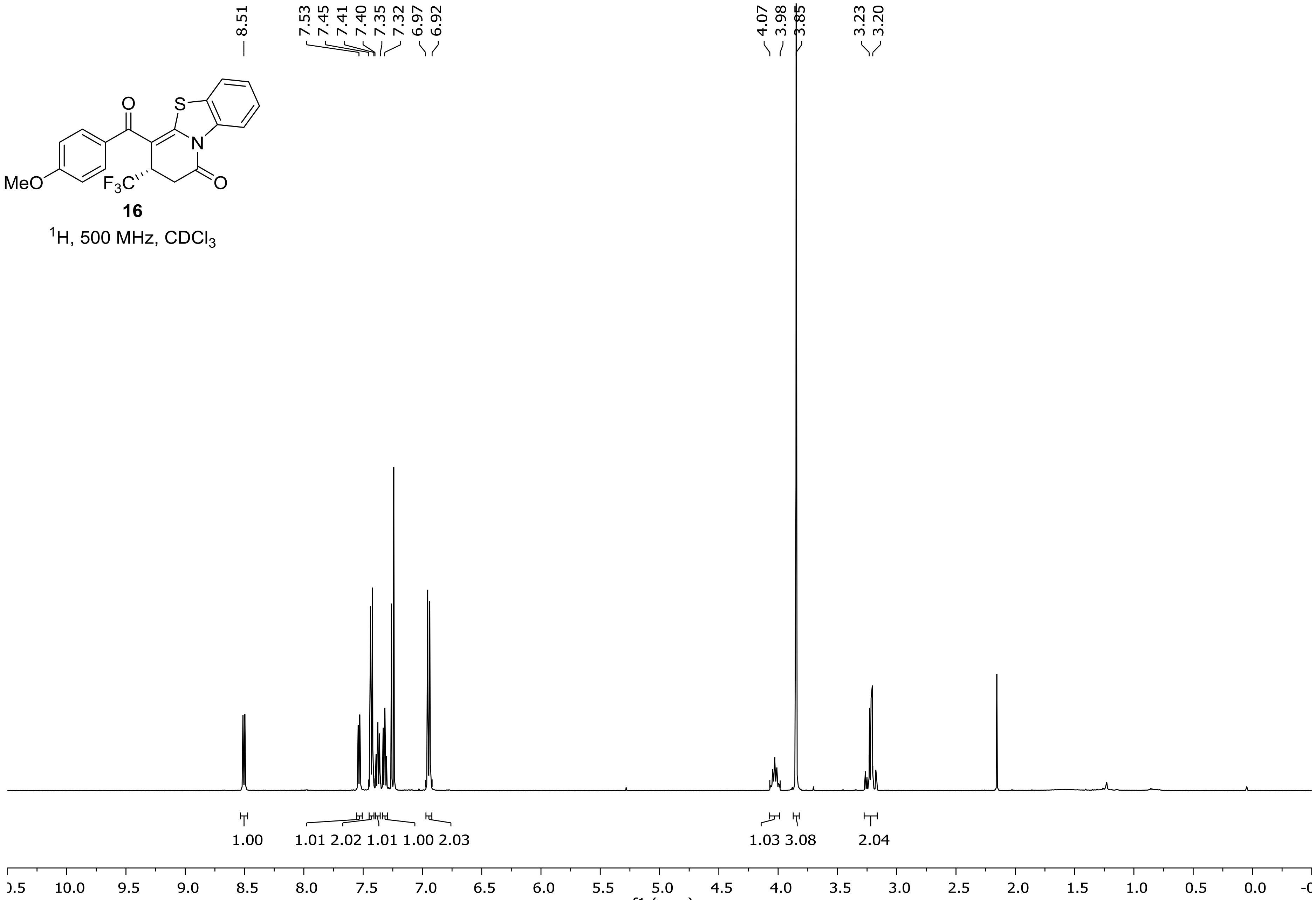

8.51
7.53
7.45
7.41
7.40
7.35
7.32
6.97
6.92
4.07
3.98
3.85
3.23
3.20
MeO
O
S
N
O
F3C
16
1H, 500 MHz, CDCl3
1.00
1.01 2.02 1.01 1.00 2.03
1.03 3.08
2.04
10.0
9.5
9.0
8.5
8.0
7.5
7.0
6.5
6.0
5.5
5.0
4.5
4.0
3.5
3.0
2.5
2.0
1.5
1.0
0.5
0.0
f1 (ppm)

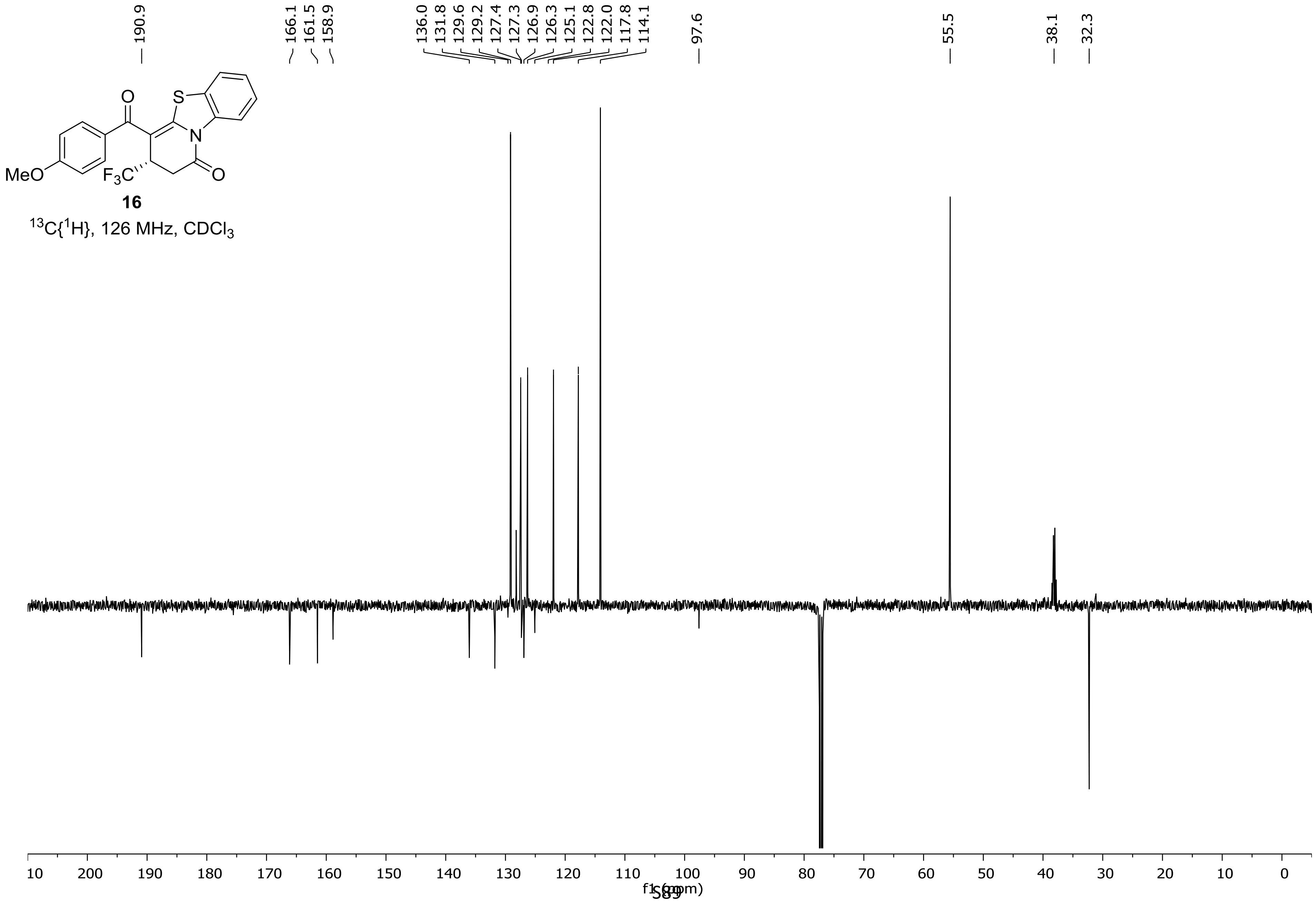

16

$^{13}C\{^{1}H\}$, 126 MHz, $CDCl_3$

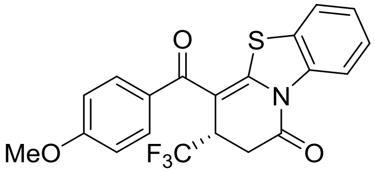

16

$^{19}F\{^1H\}$, 376 MHz, $CDCl_3$

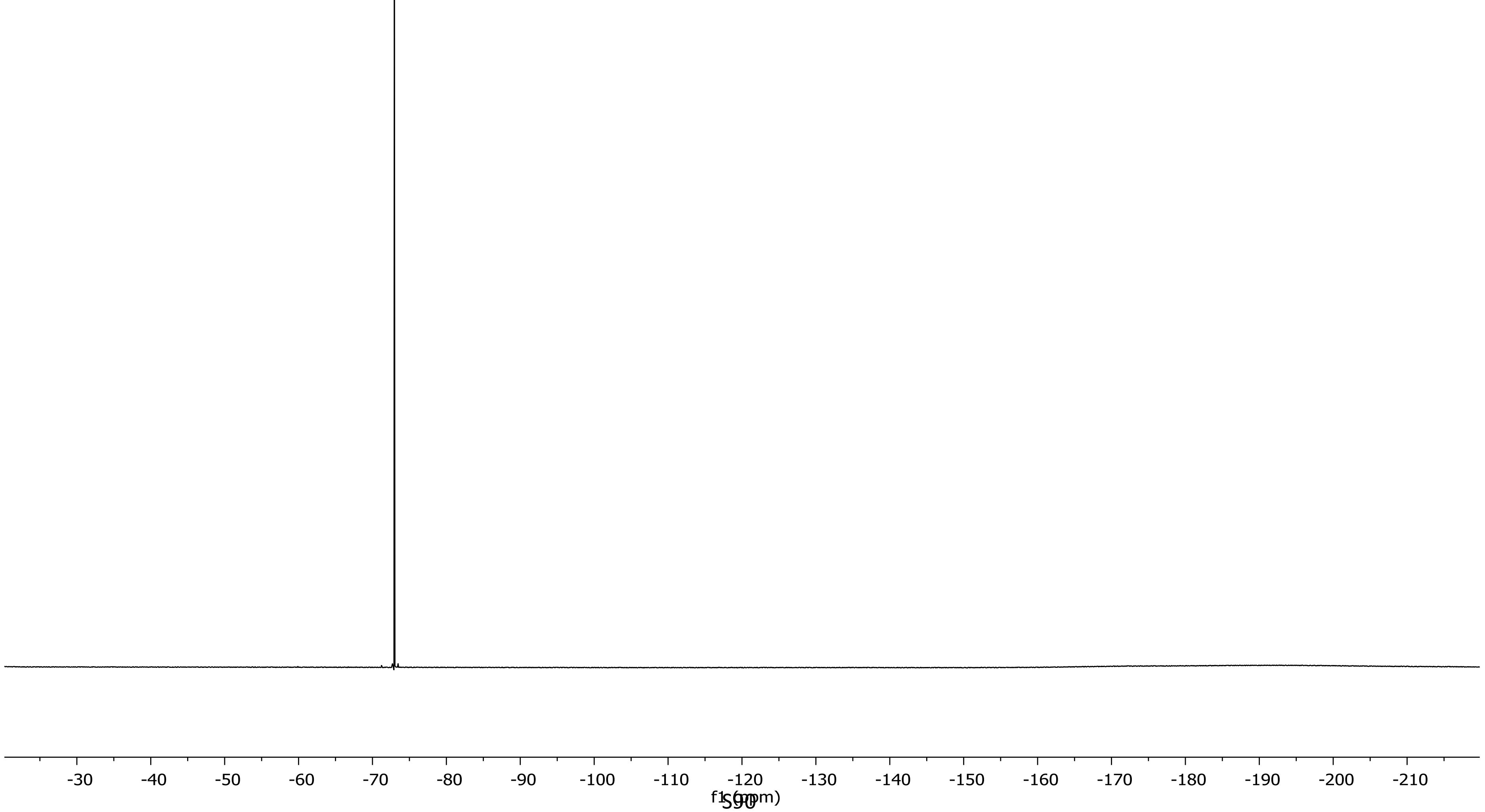

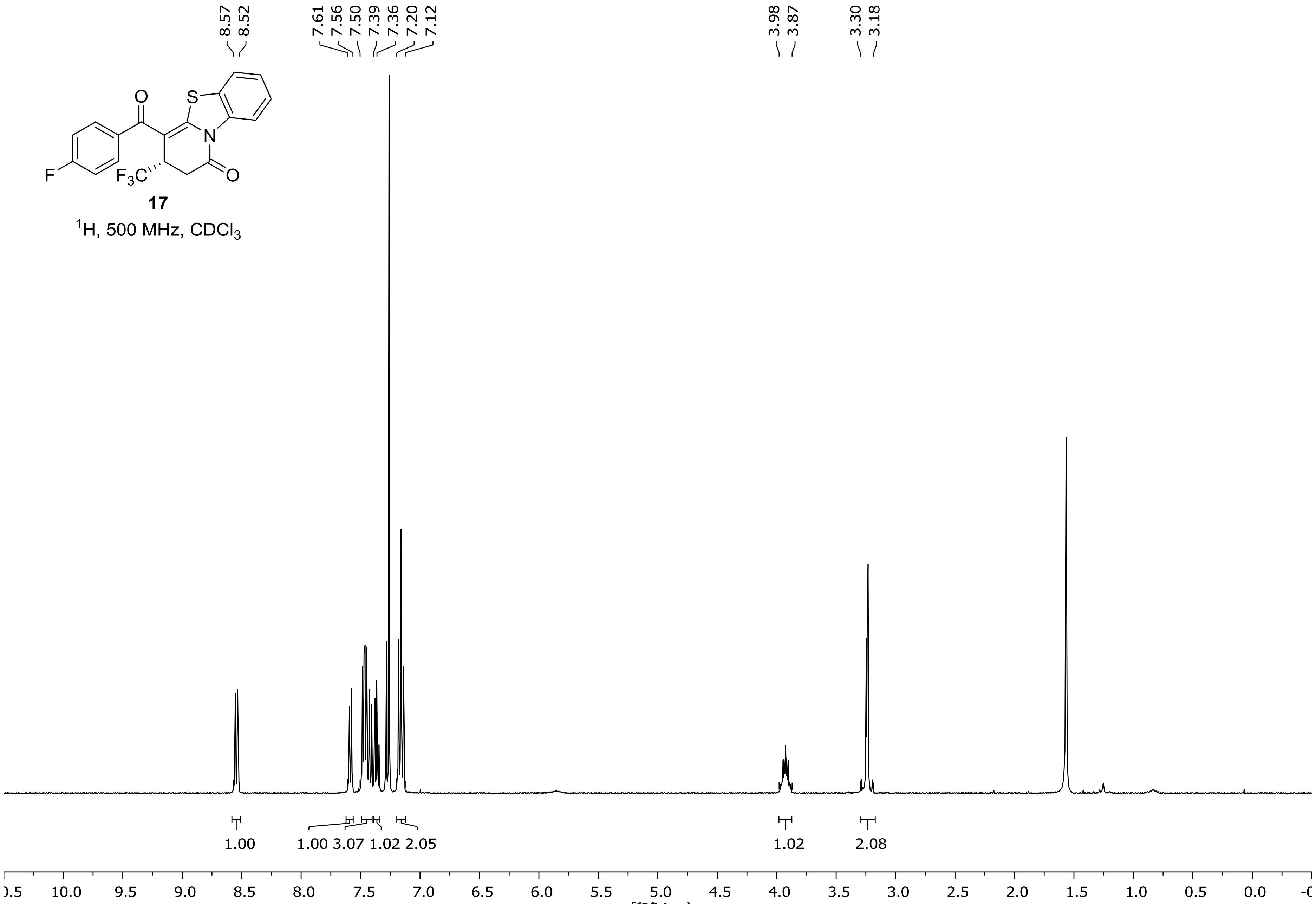
8.57
8.52
7.61
7.56
7.50
7.39
7.36
7.20
7.12
3.98
3.87
3.30
3.18
O
S
N
O
F
F_3C
17
^{1}H, 500 MHz, $CDCl_3$
1.00
1.00 3.07 1.02 2.05
1.02
2.08
10.0 9.5 9.0 8.5 8.0 7.5 7.0 6.5 6.0 5.5 5.0 4.5 4.0 3.5 3.0 2.5 2.0 1.5 1.0 0.5 0.0
f1 (ppm)

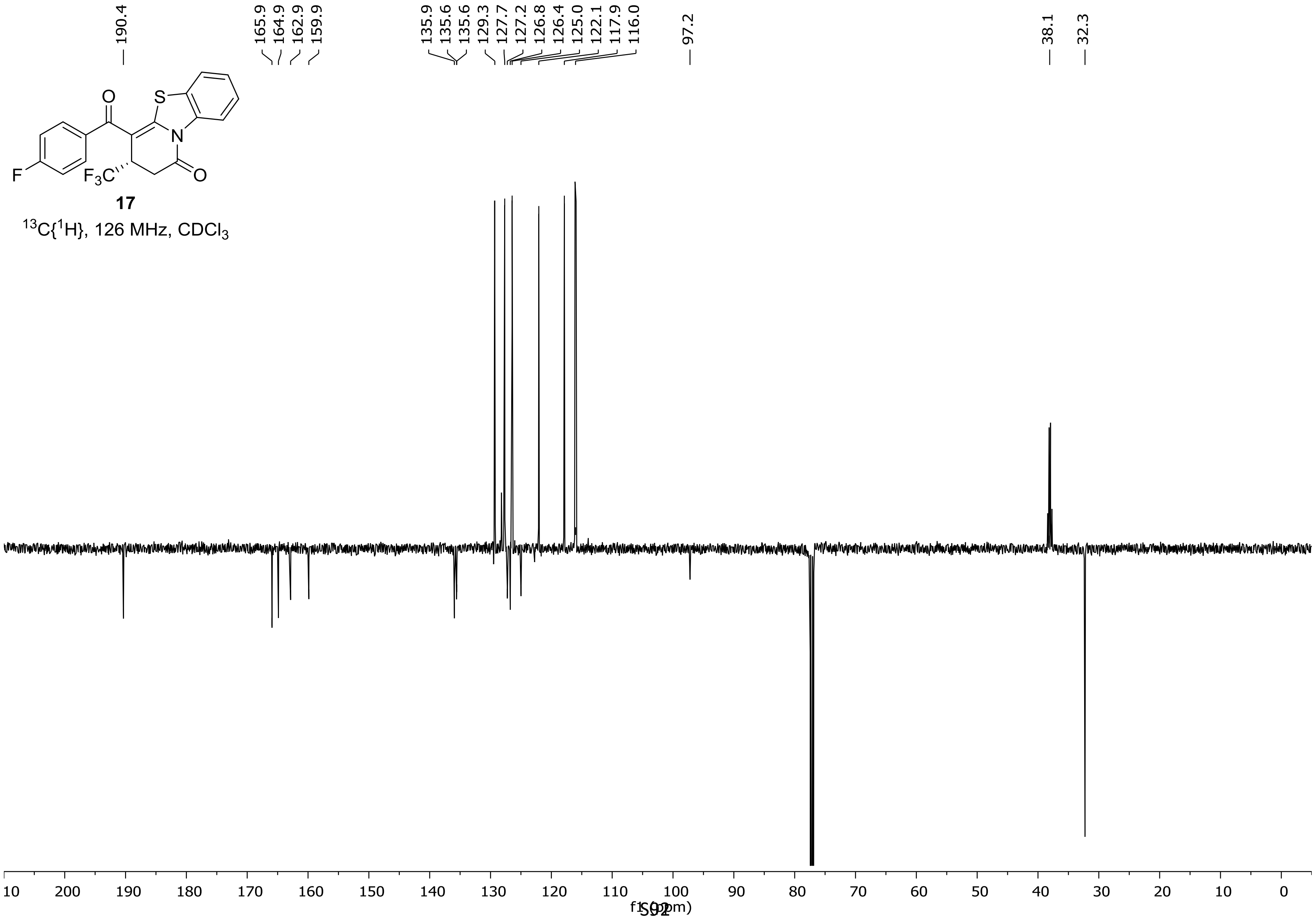

190.4
165.9
164.9
162.9
159.9
135.9
135.6
135.6
129.3
127.7
127.2
126.8
126.4
125.0
122.1
117.9
116.0
97.2
38.1
32.3
O
S
N
F
F3C
O
17
13C{1H}, 126 MHz, CDCl3
f1 (ppm)

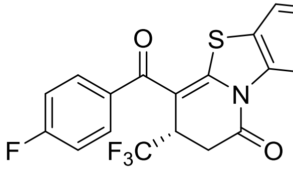

17

$^{19}F\{^{1}H\}$, 376 MHz, $CDCl_3$

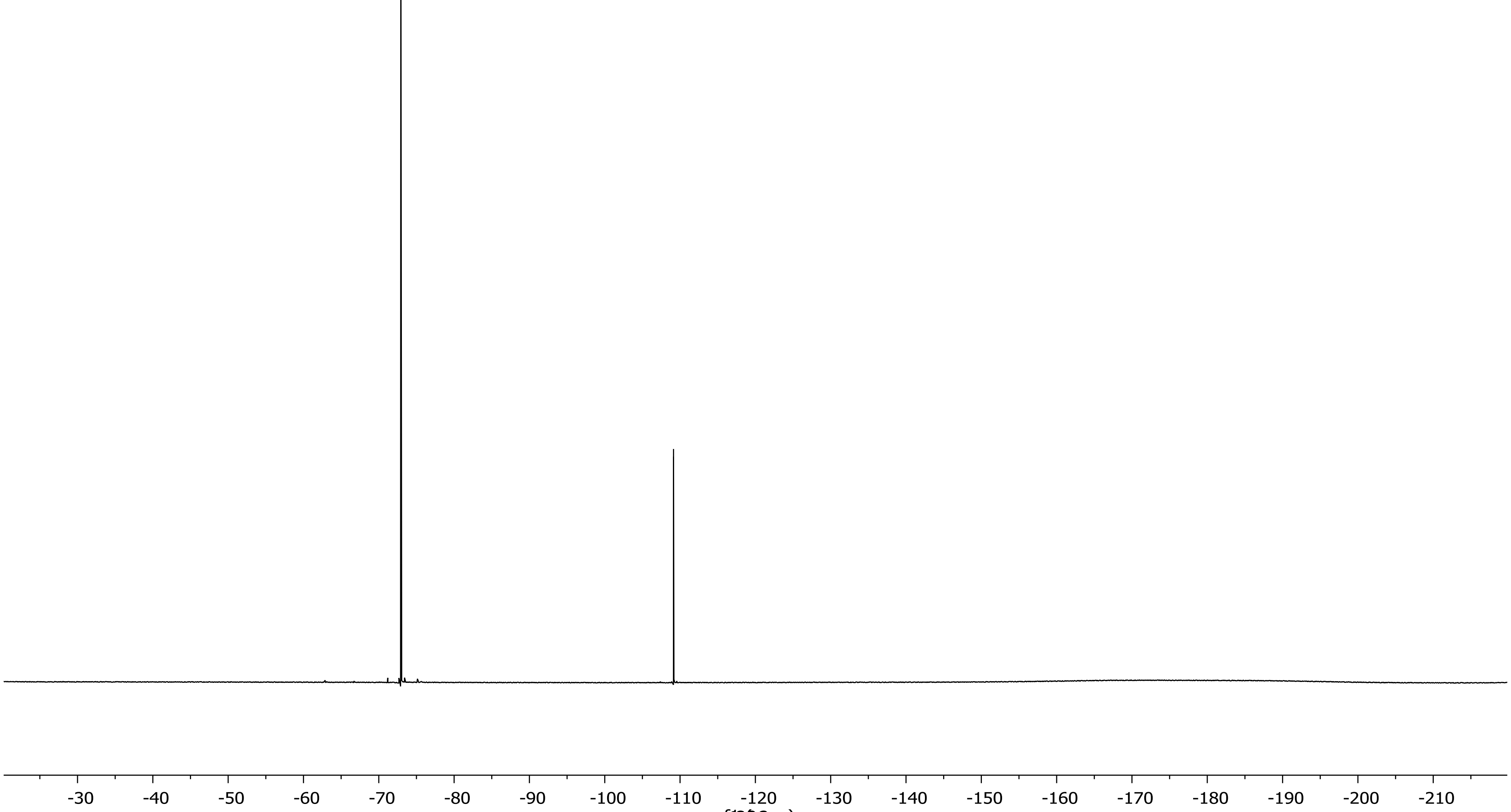

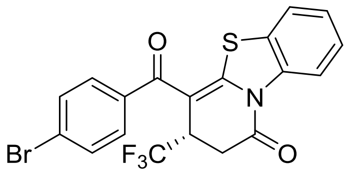

18

^{1}H, 500 MHz, $CDCl_3$

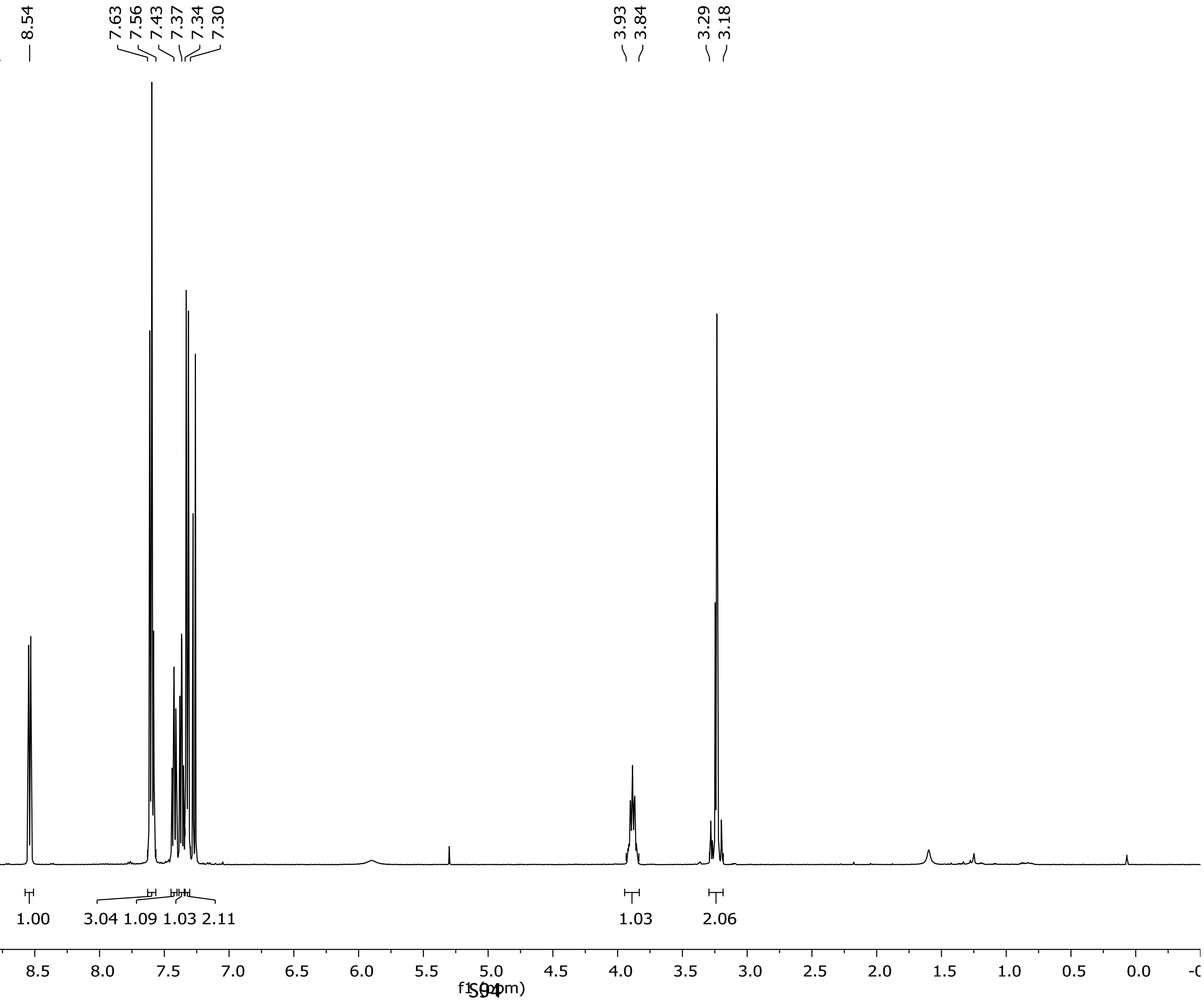

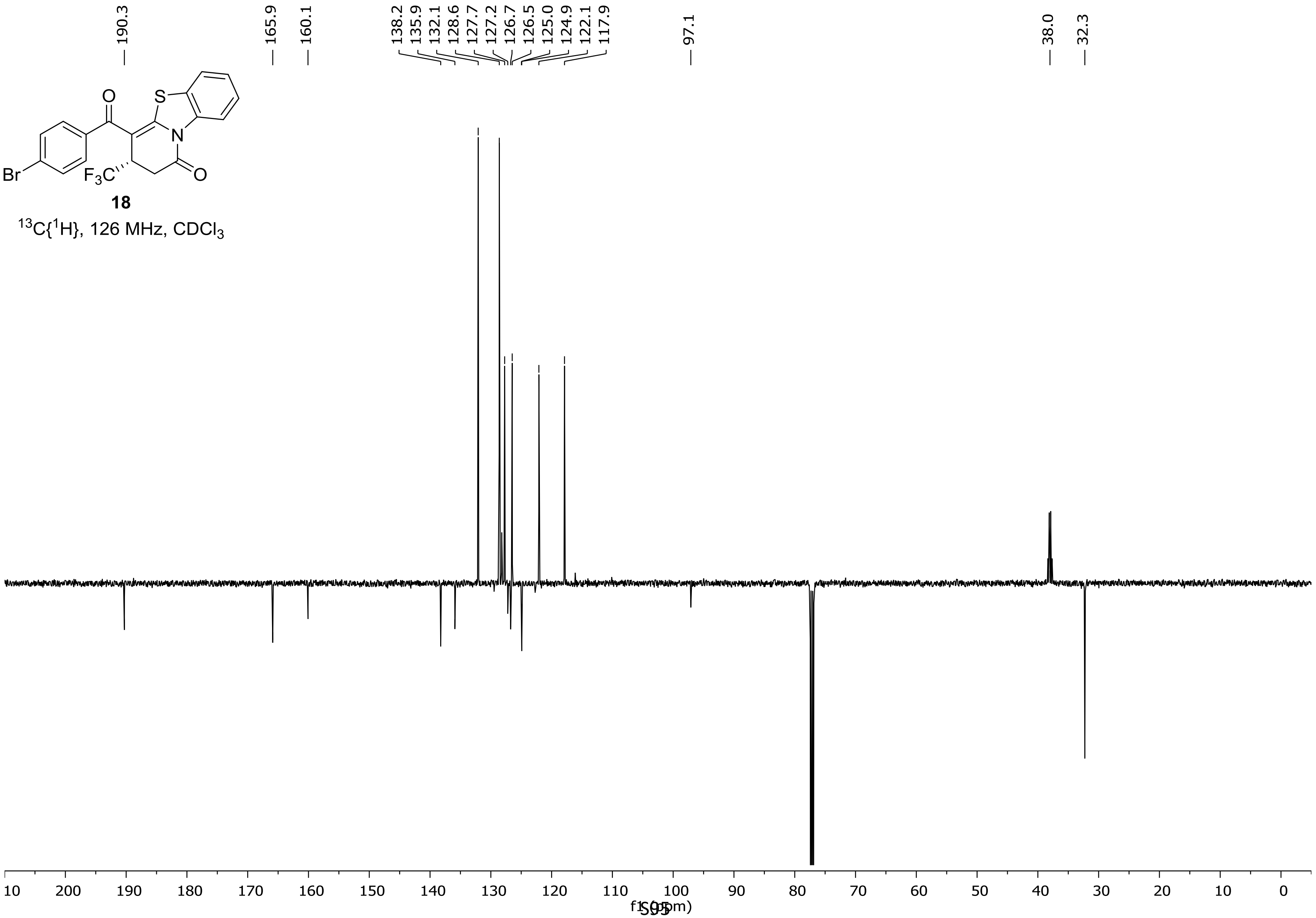
190.3
165.9
160.1
138.2
135.9
132.1
128.6
127.7
127.2
126.7
126.5
125.0
124.9
122.1
117.9
97.1
38.0
32.3
O
S
N
Br
F_3C
O
18
$^{13}C\{^1H\}$, 126 MHz, $CDCl_3$
10
200
190
180
170
160
150
140
130
120
110
100
90
80
70
60
50
40
30
20
10
0
f1 (ppm)

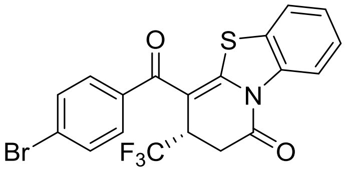

18

$^{19}F\{^{1}H\}$, 376 MHz, $CDCl_3$

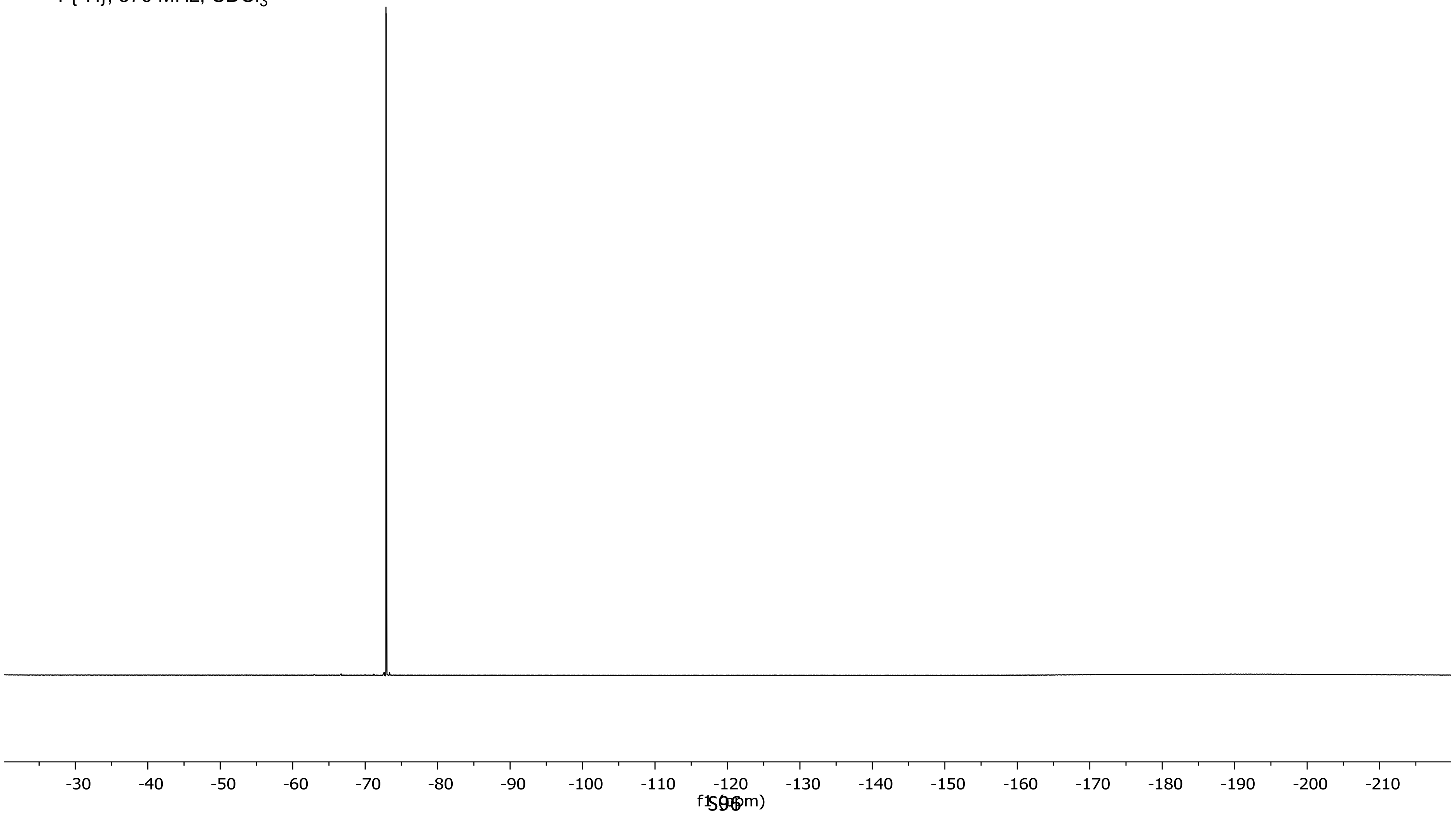

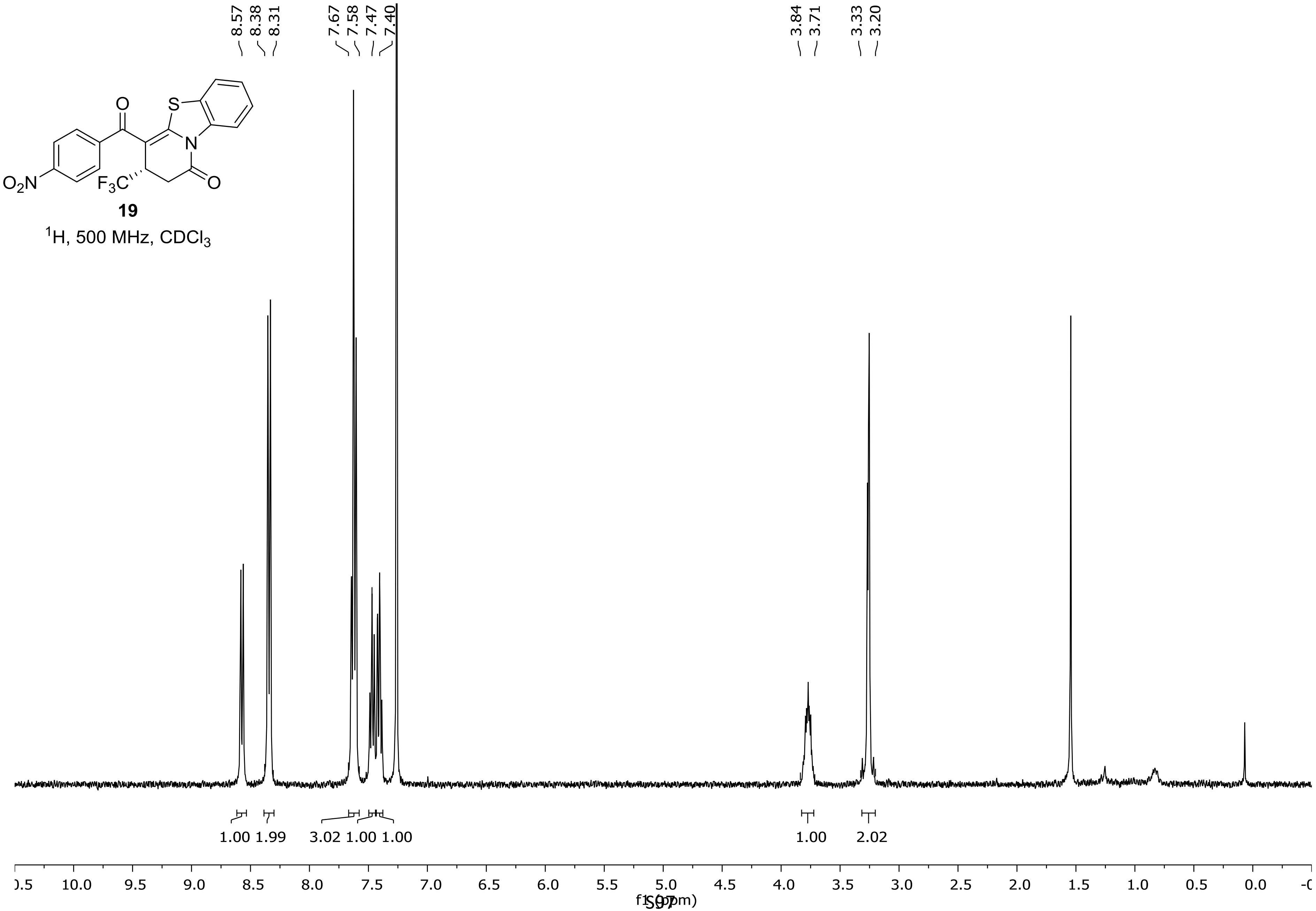

O
S
N
O
O_2N
F_3C
19
^1H, 500 MHz, $CDCl_3$
8.57
8.38
8.31
7.67
7.58
7.47
7.40
3.84
3.71
3.33
3.20
1.00
1.99
3.02
1.00
1.00
1.00
2.02
10.0
9.5
9.0
8.5
8.0
7.5
7.0
6.5
6.0
5.5
5.0
4.5
4.0
3.5
3.0
2.5
2.0
1.5
1.0
0.5
0.0
f1 (ppm)

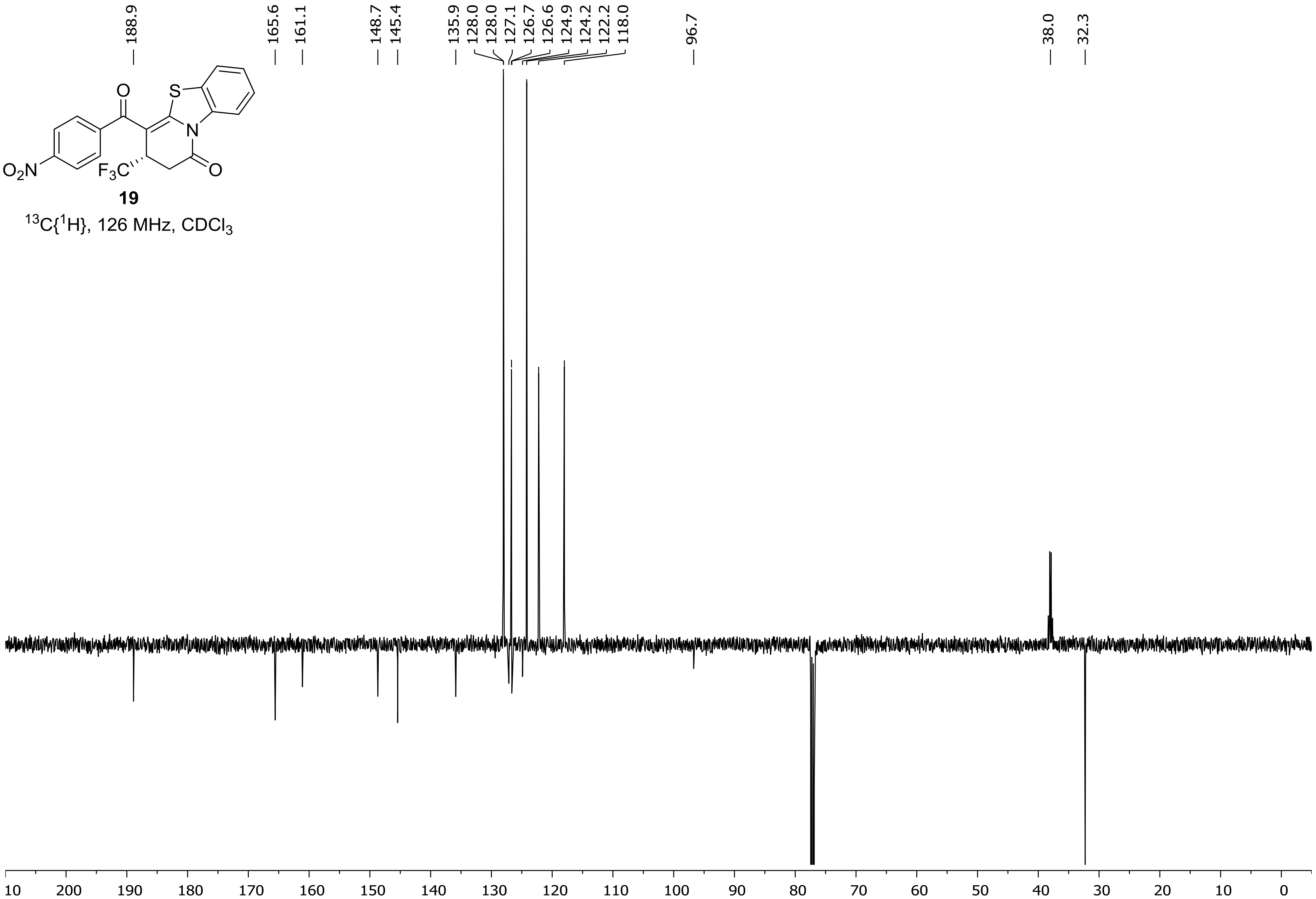

19

$^{13}C\{^{1}H\}$, 126 MHz, $CDCl_3$

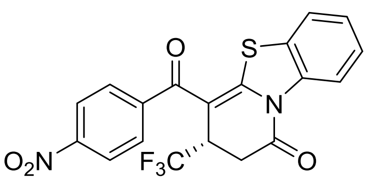

19

$^{19}F\{^{1}H\}$, 376 MHz, $CDCl_3$

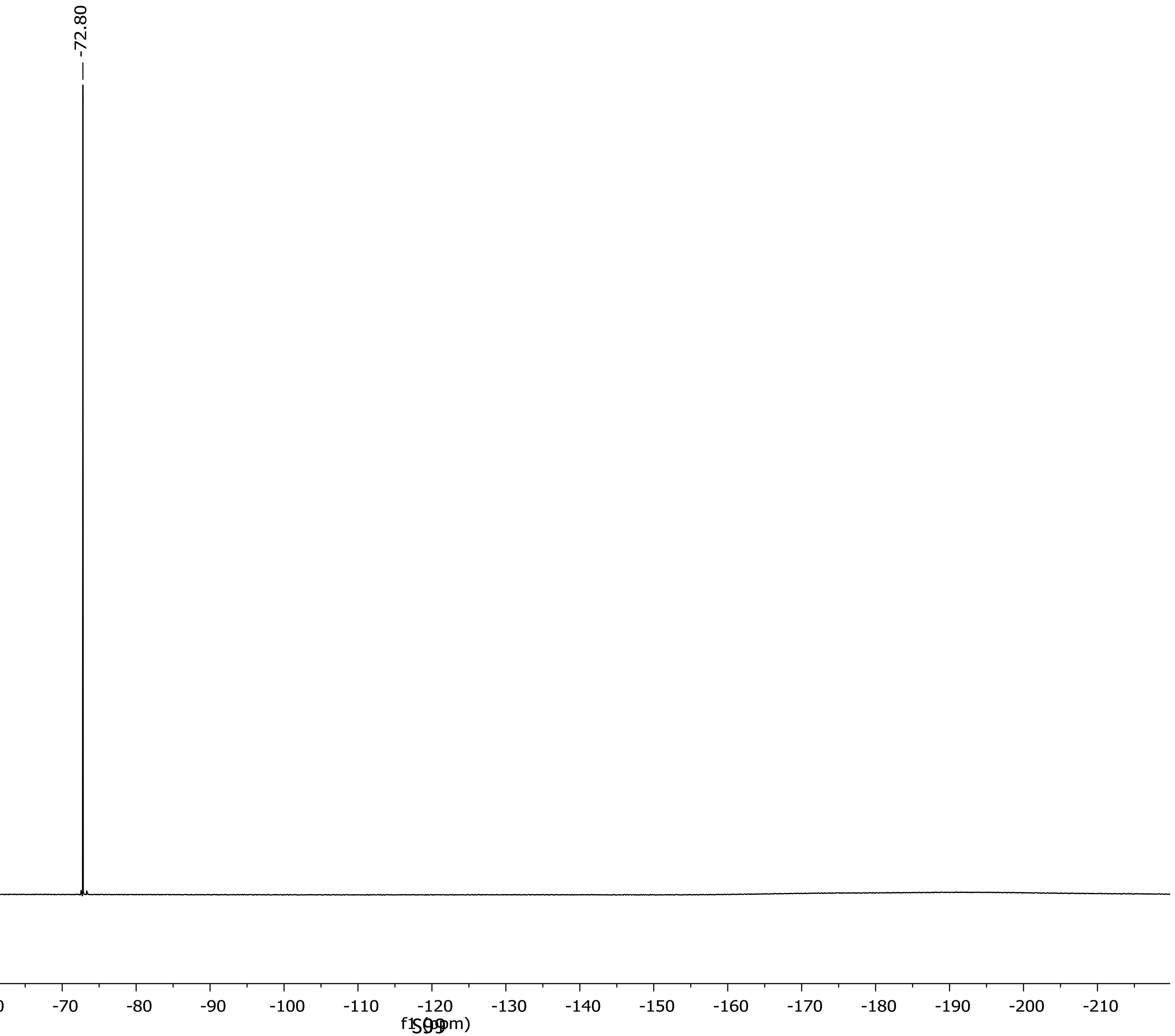

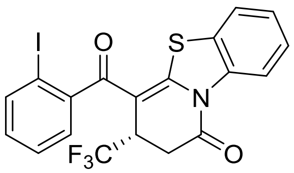

20

^{1}H, 500 MHz, $CDCl_3$

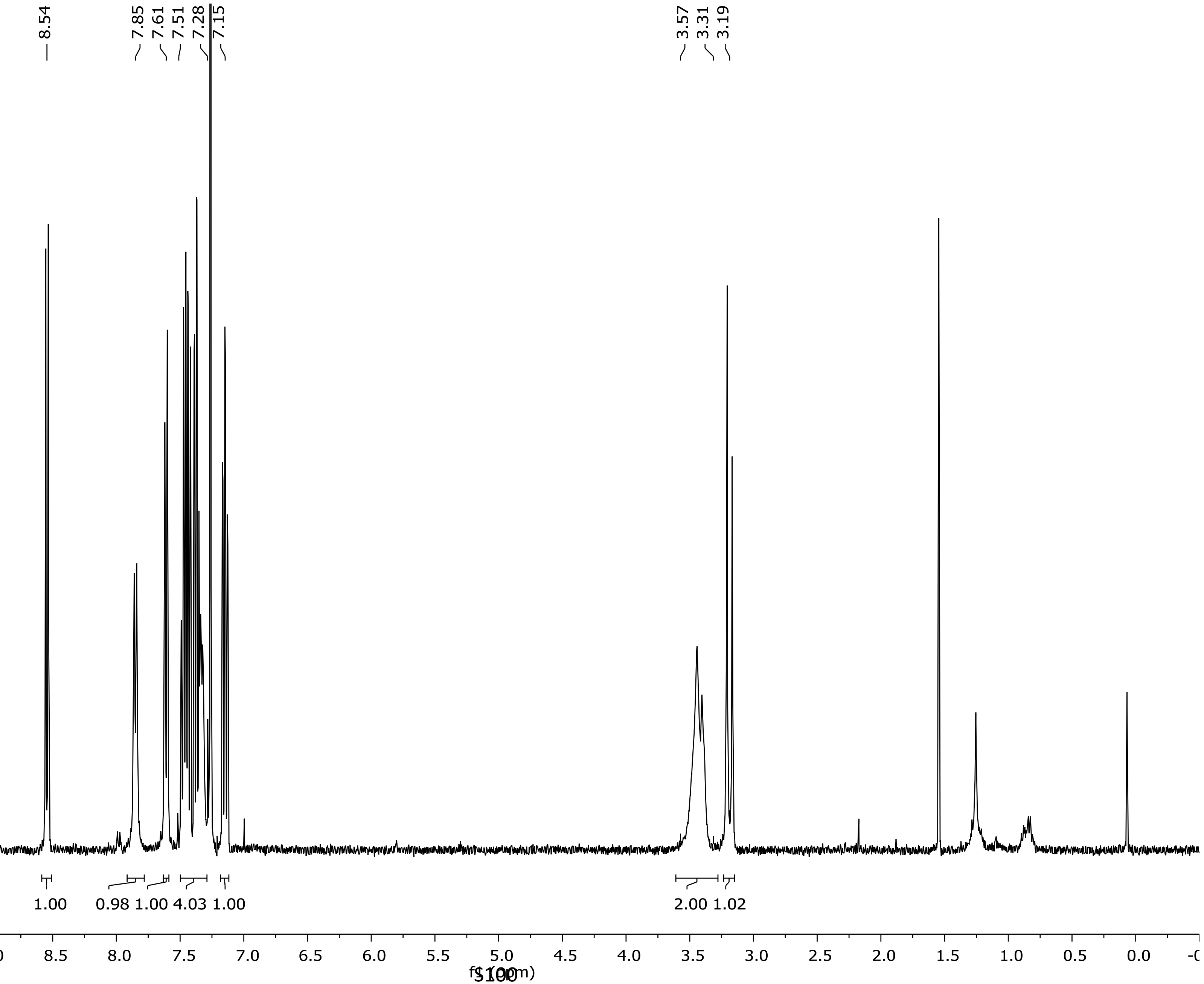

20

$^{19}C\{^{1}H\}$, 126 MHz, $CDCl_3$

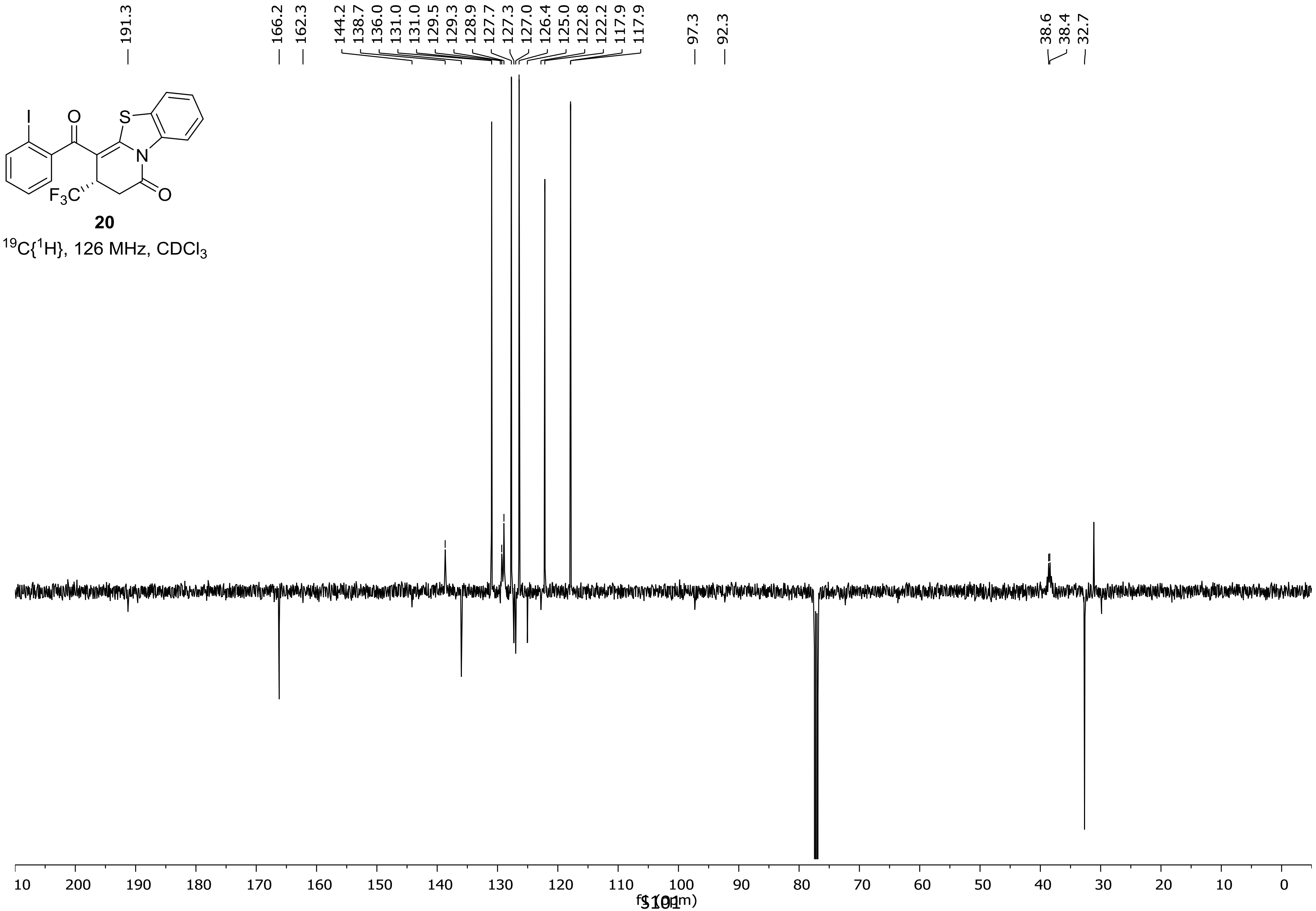

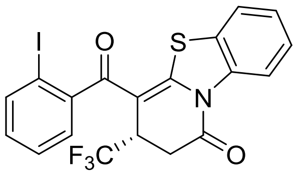

20

$^{19}F\{^{1}H\}$, 376 MHz, $CDCl_3$

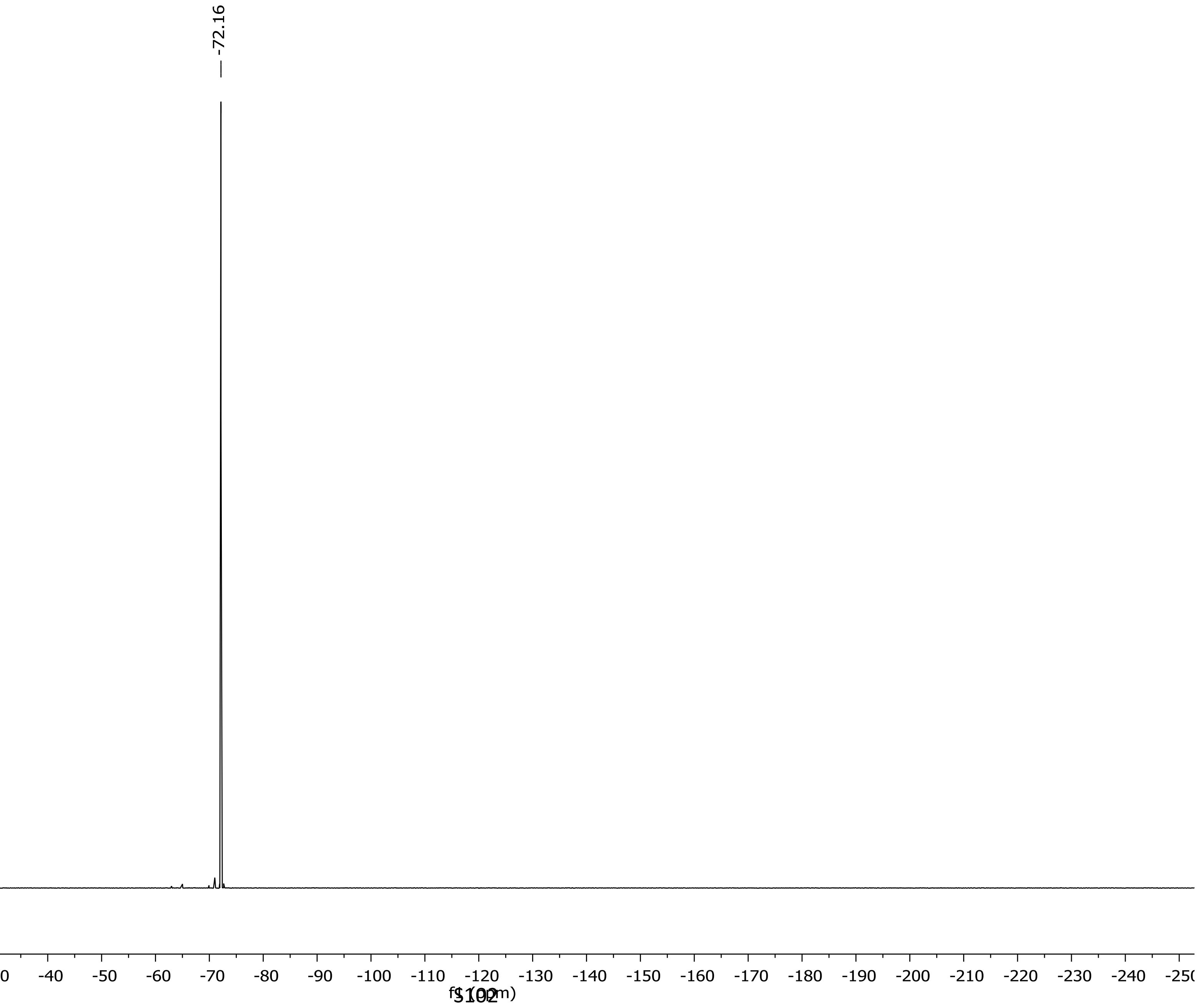

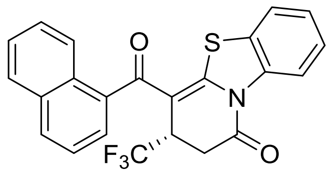

21

^1H, 500 MHz, $CDCl_3$

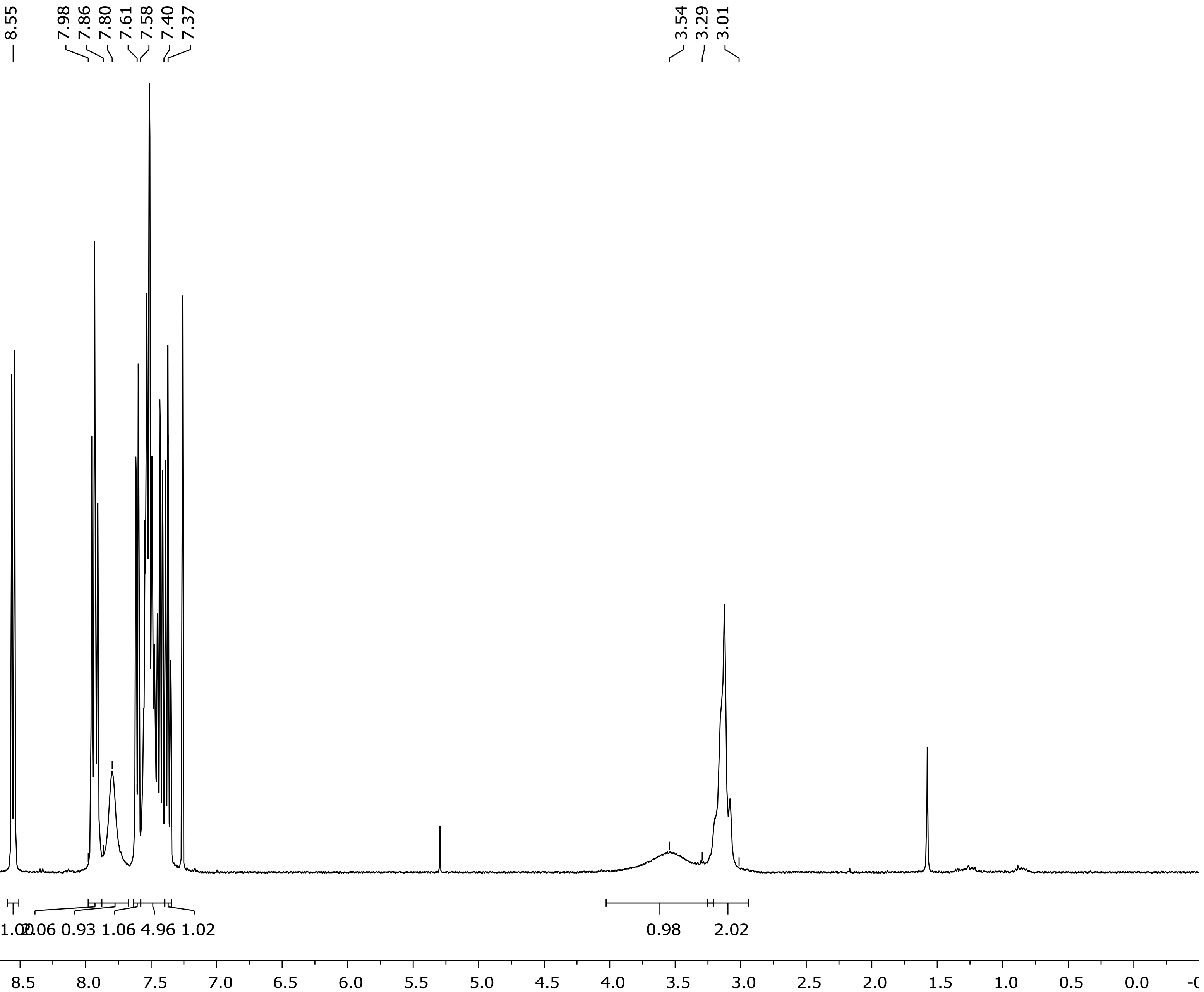

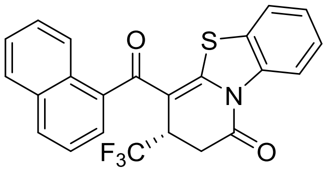

21

$^{13}C\{^{1}H\}$, 126 MHz, $CDCl_3$

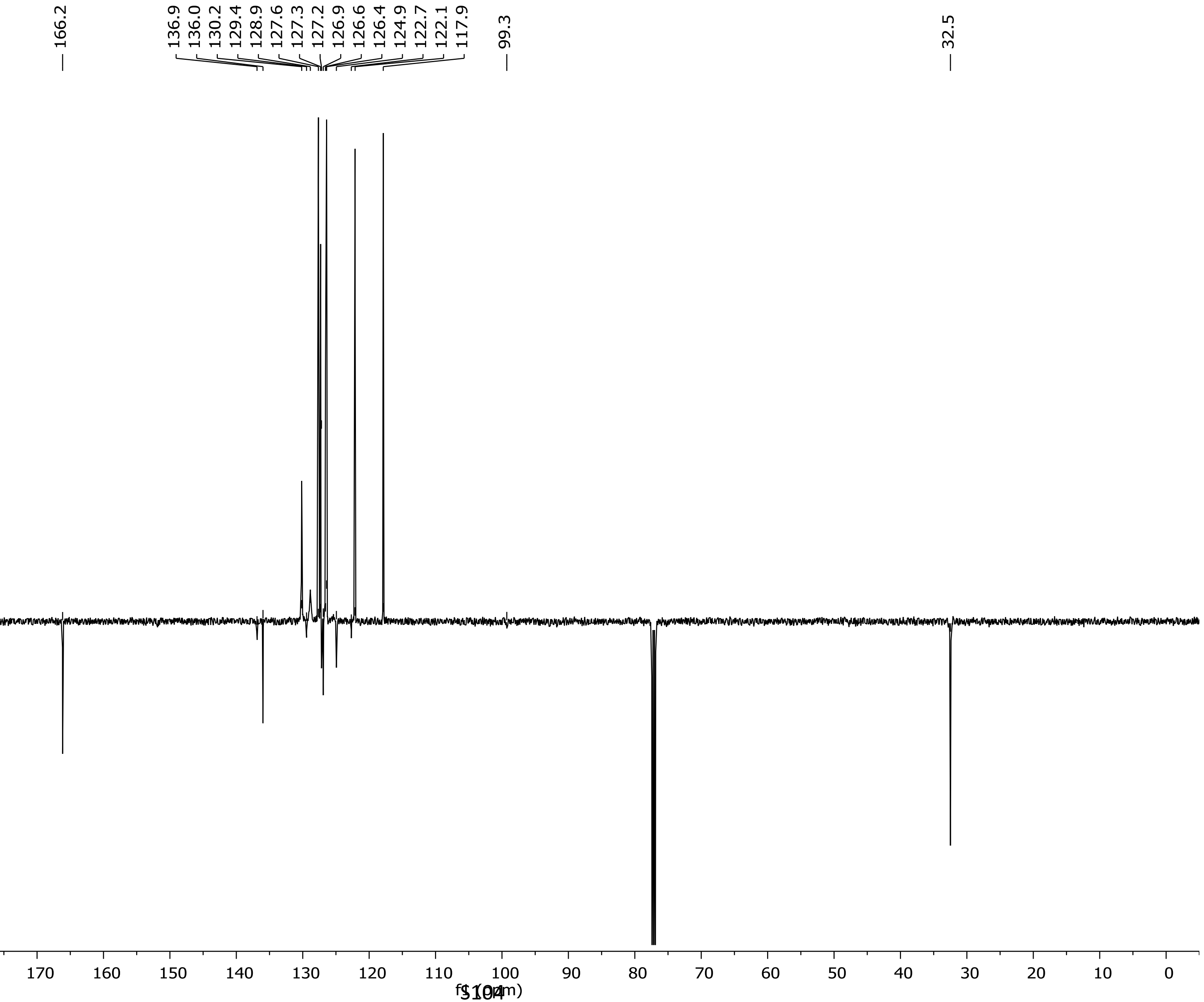

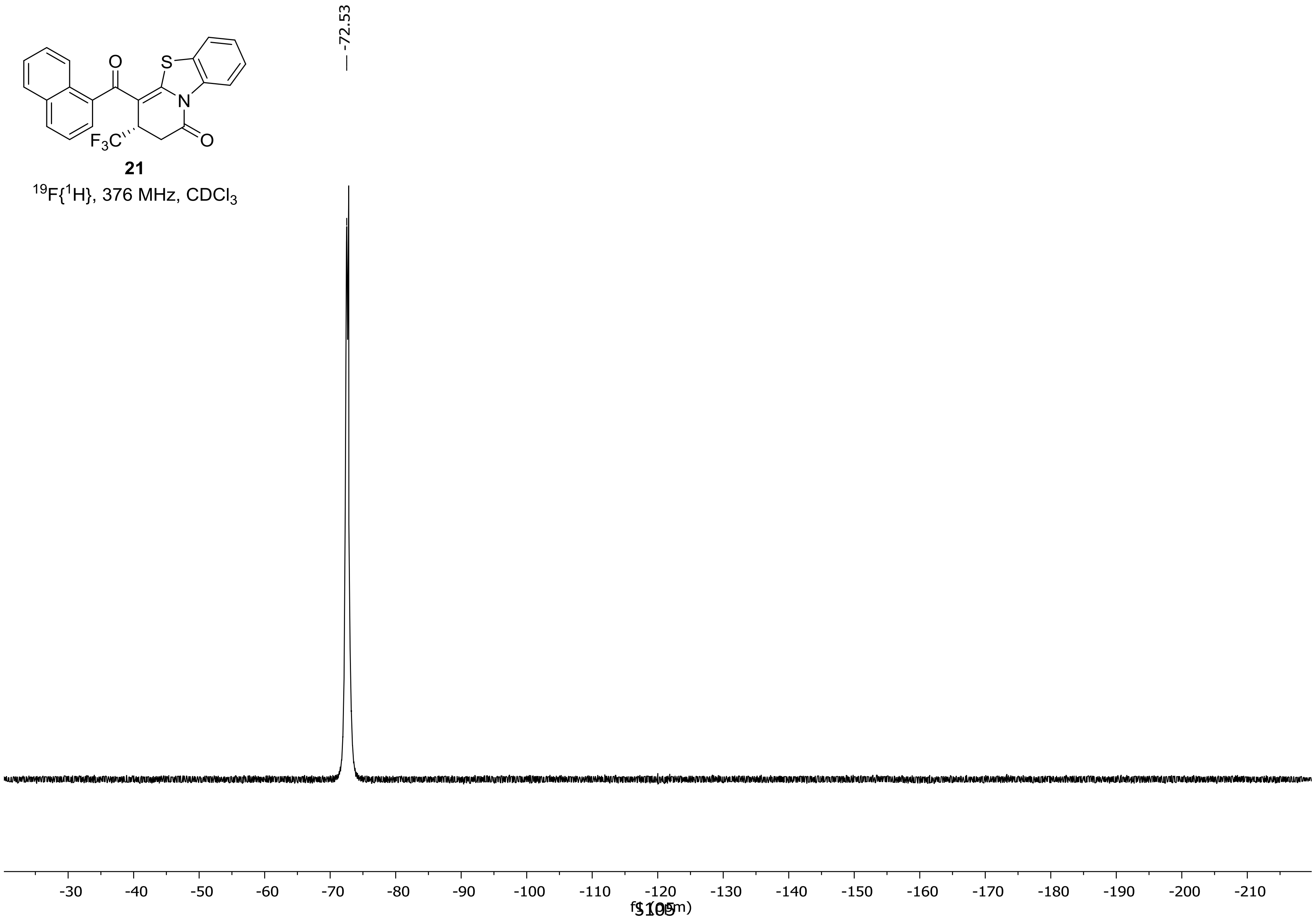
-72.53
21
$^{19}F\{^{1}H\}$, 376 MHz, $CDCl_3$
-30
-40
-50
-60
-70
-80
-90
-100
-110
-120
-130
-140
-150
-160
-170
-180
-190
-200
-210
f1 (ppm)

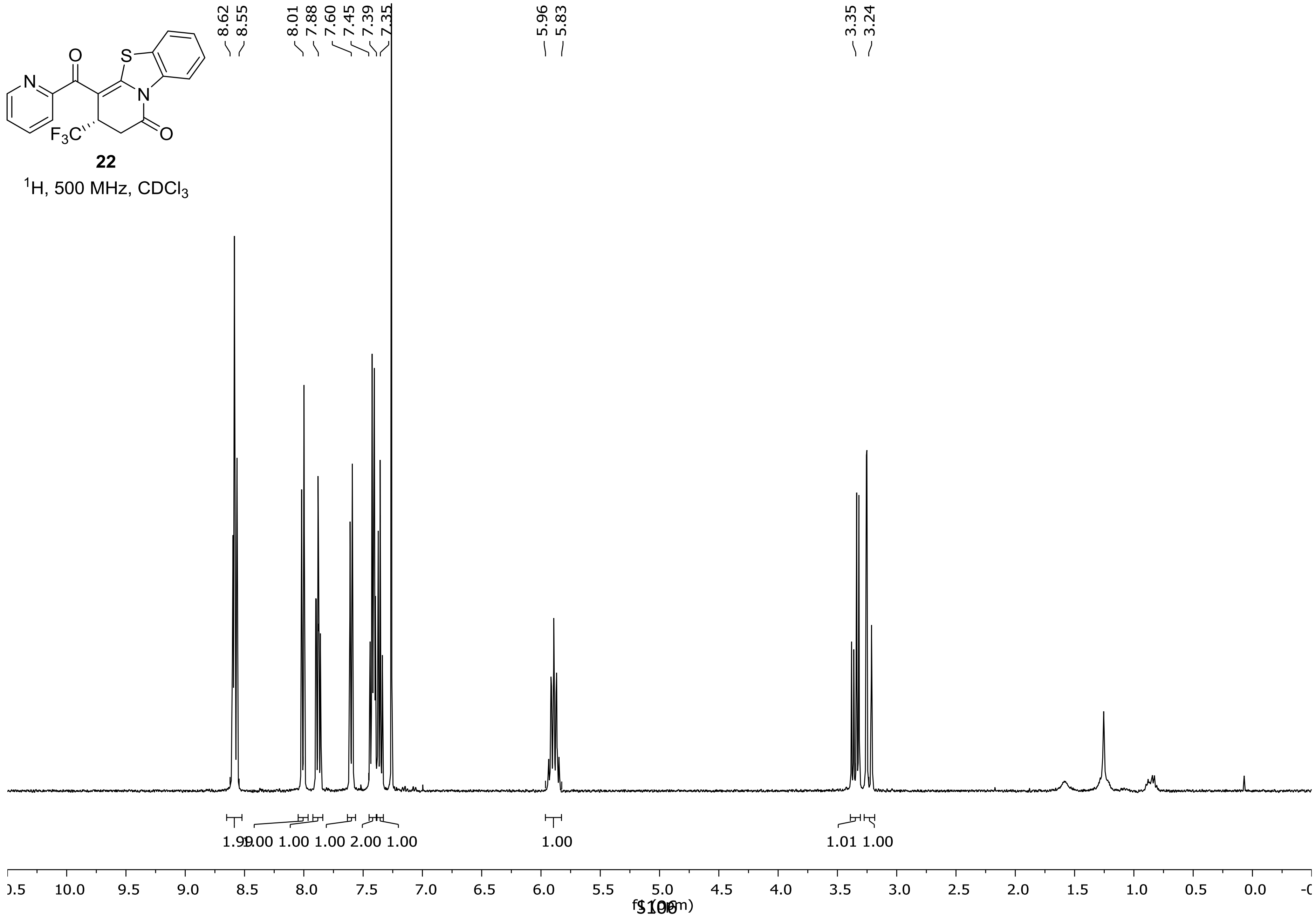

O
S
N
N
O
F3C
22
1H, 500 MHz, CDCl3
8.62
8.55
8.01
7.88
7.60
7.45
7.39
7.35
5.96
5.83
3.35
3.24
1.99
1.00 1.00 1.00 2.00 1.00
1.00
1.01 1.00
10.0 9.5 9.0 8.5 8.0 7.5 7.0 6.5 6.0 5.5 5.0 4.5 4.0 3.5 3.0 2.5 2.0 1.5 1.0 0.5 0.0
f1 (ppm)

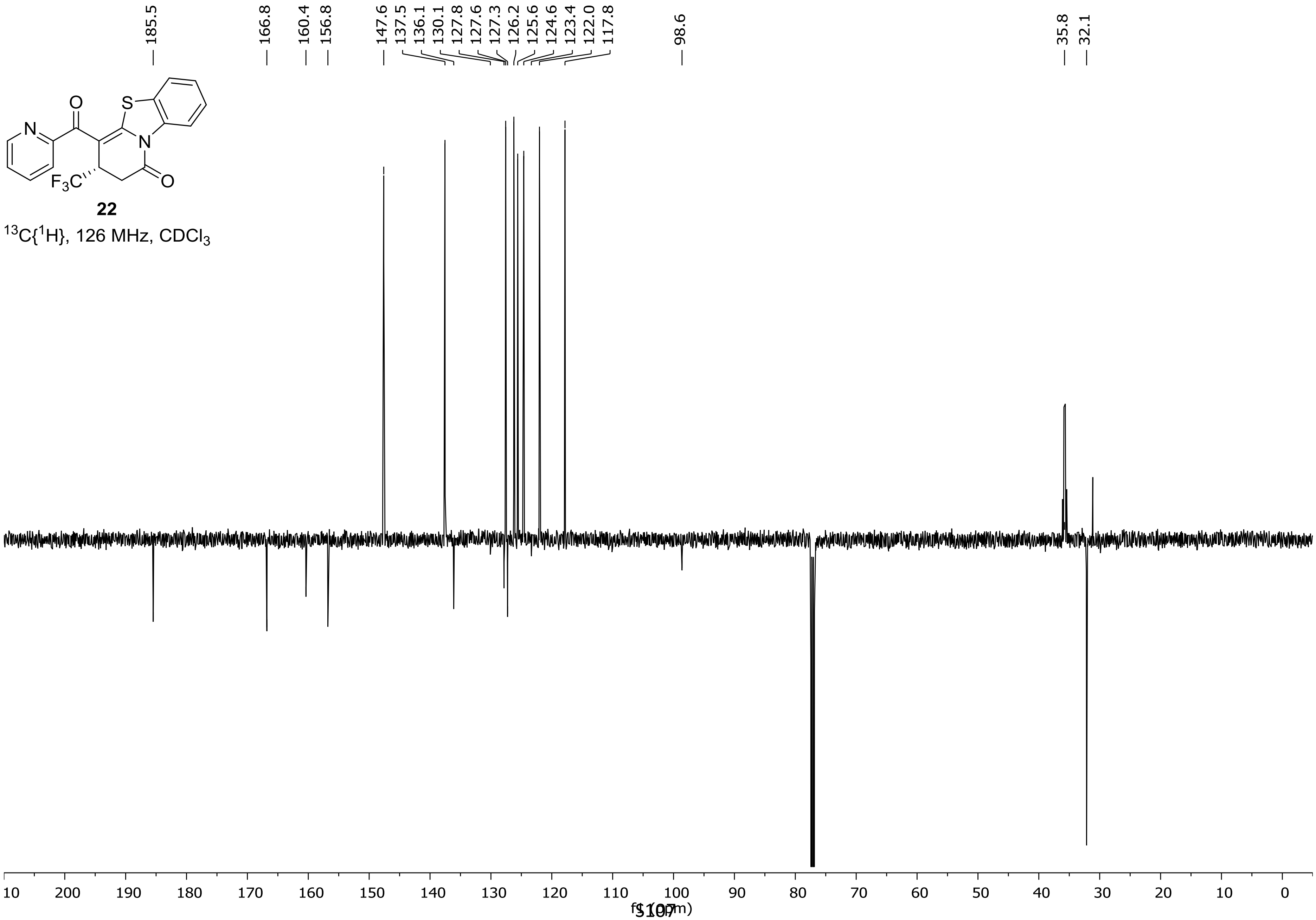
185.5
166.8
160.4
156.8
147.6
137.5
136.1
130.1
127.8
127.6
127.3
126.2
125.6
124.6
123.4
122.0
117.8
98.6
35.8
32.1
22
$^{13}C\{^{1}H\}$, 126 MHz, $CDCl_3$
f1 (ppm)

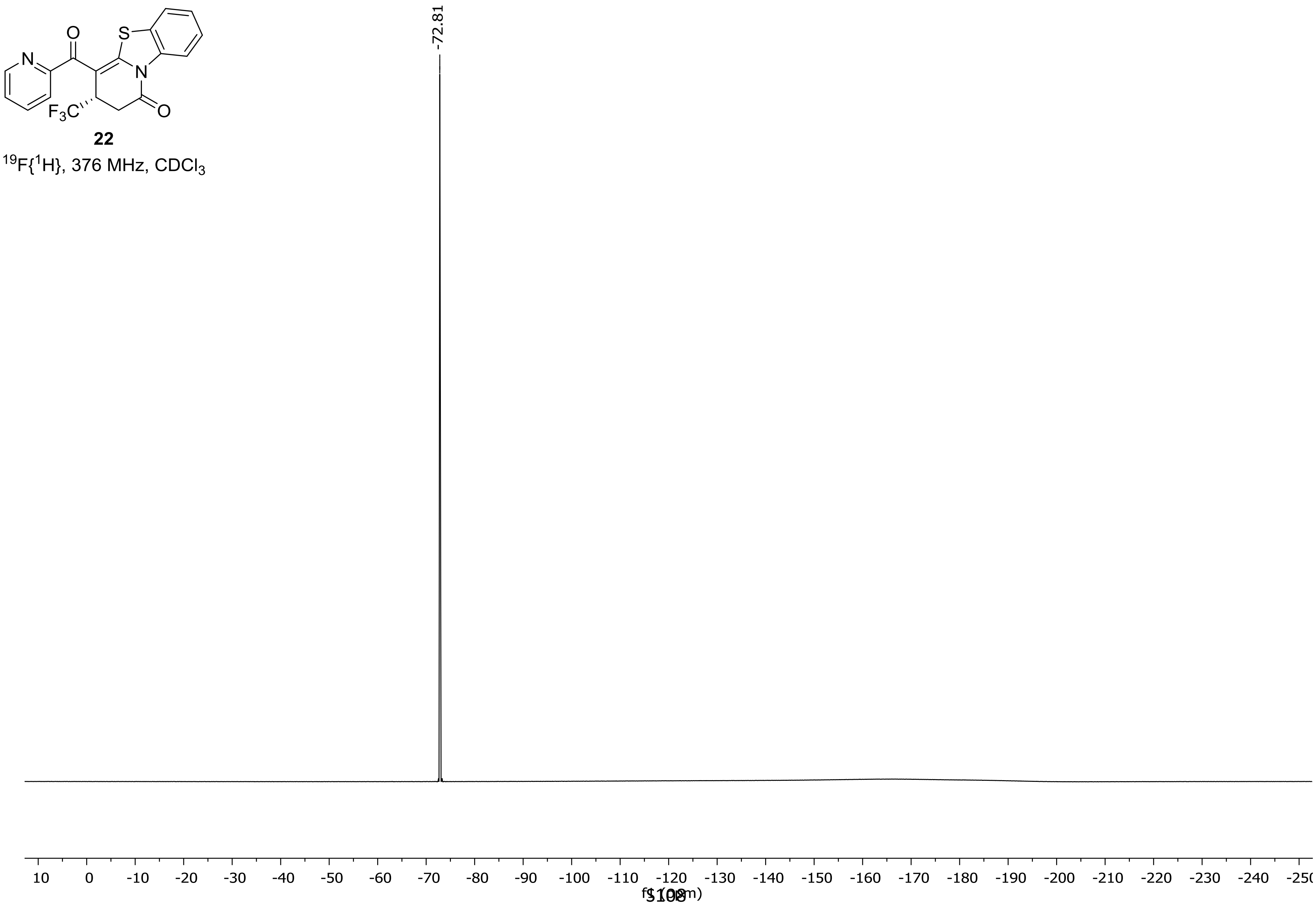
22
$^{19}F\{^{1}H\}$, 376 MHz, $CDCl_3$
-72.81
10 0 -10 -20 -30 -40 -50 -60 -70 -80 -90 -100 -110 -120 -130 -140 -150 -160 -170 -180 -190 -200 -210 -220 -230 -240 -250
f1 (ppm)

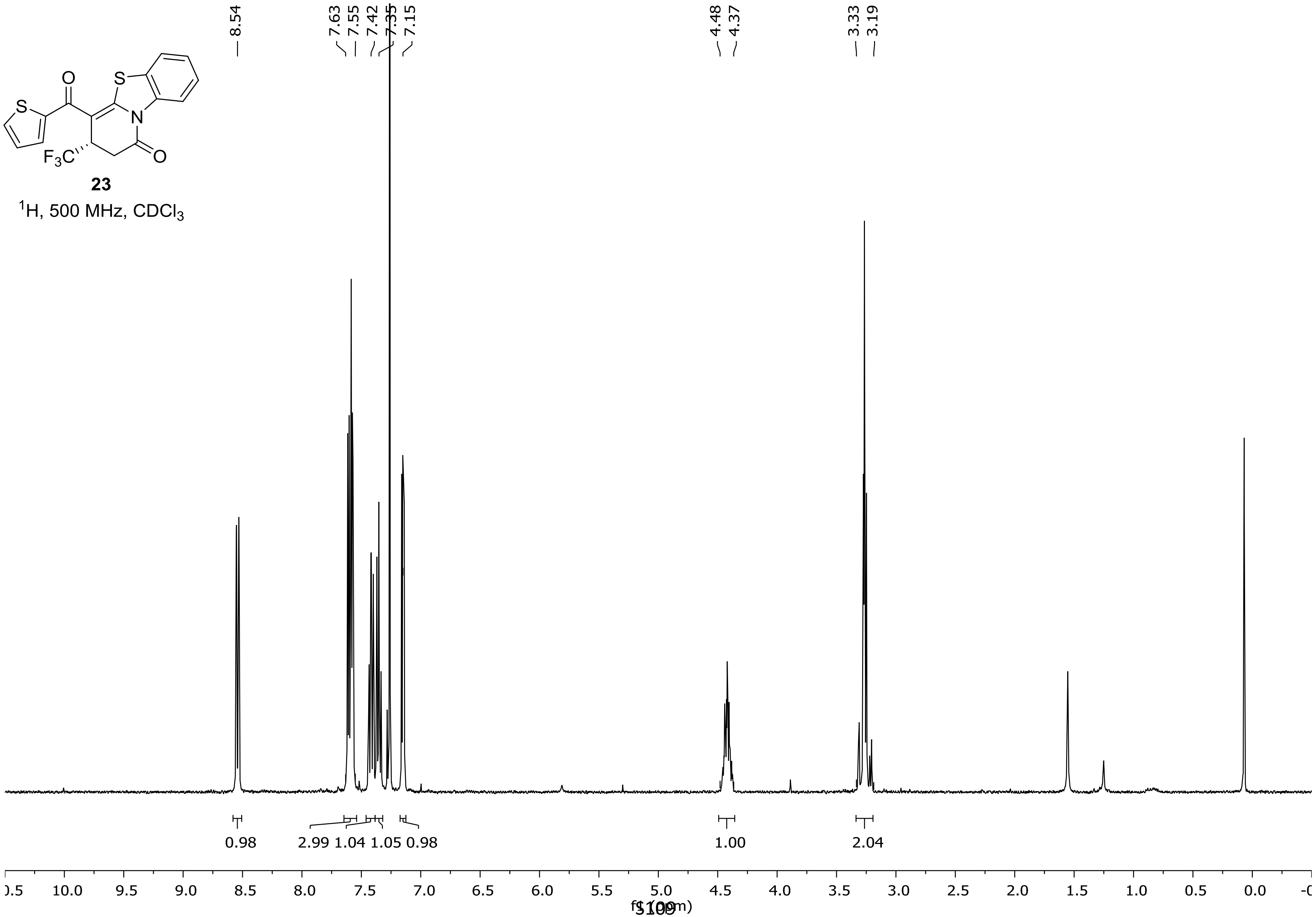
8.54
7.63
7.55
7.42
7.35
7.15
4.48
4.37
3.33
3.19
O
S
S
N
F3C
O
23
1H, 500 MHz, CDCl3
0.98
2.99
1.04
1.05
0.98
1.00
2.04
10.0
9.5
9.0
8.5
8.0
7.5
7.0
6.5
6.0
5.5
5.0
4.5
4.0
3.5
3.0
2.5
2.0
1.5
1.0
0.5
0.0
f1 (ppm)

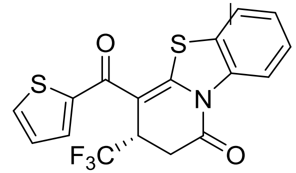

23

$^{13}C\{^1H\}$, 126 MHz, $CDCl_3$

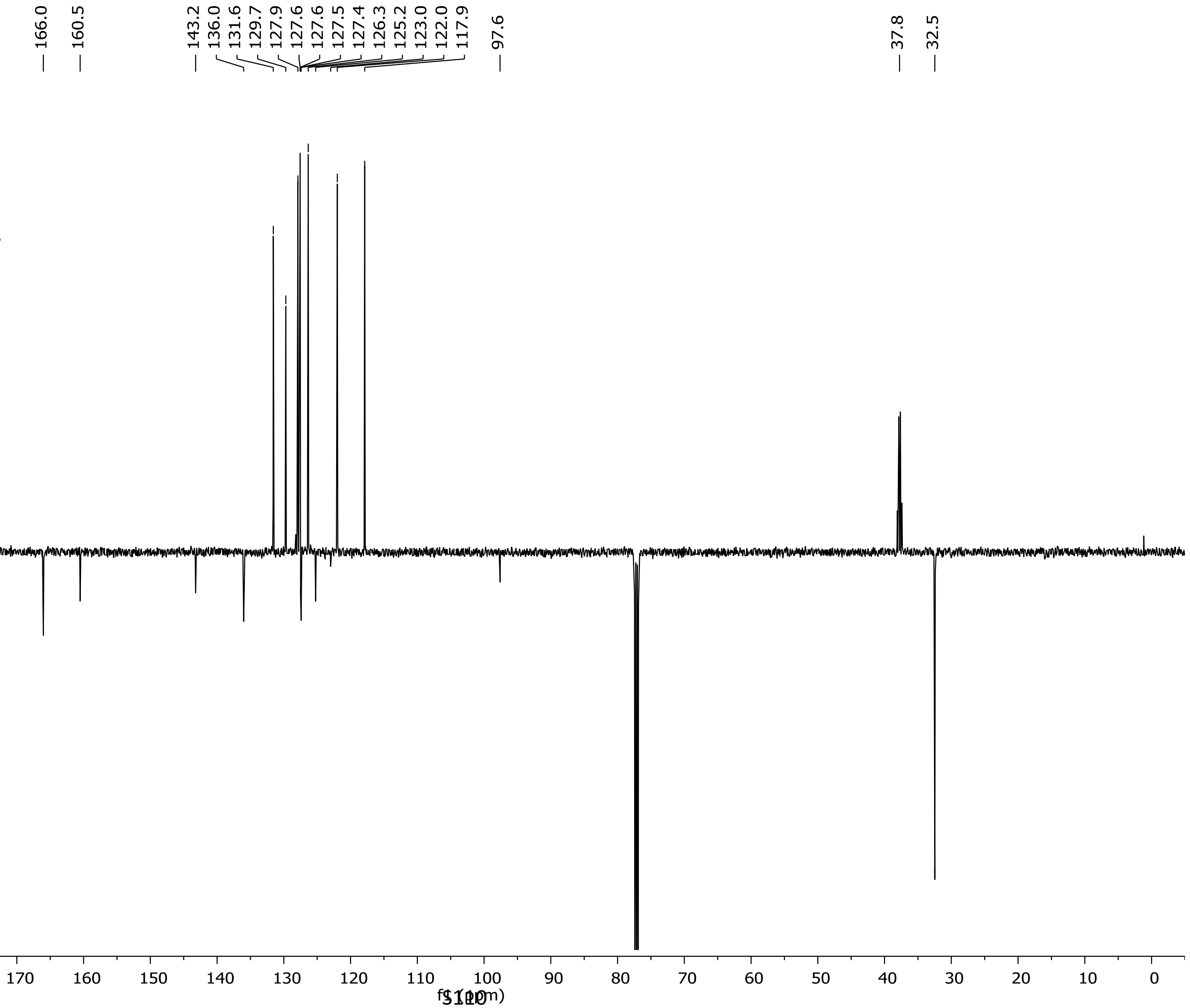

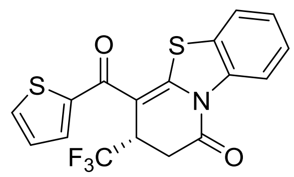

23

$^{19}F\{^1H\}$, 376 MHz, $CDCl_3$

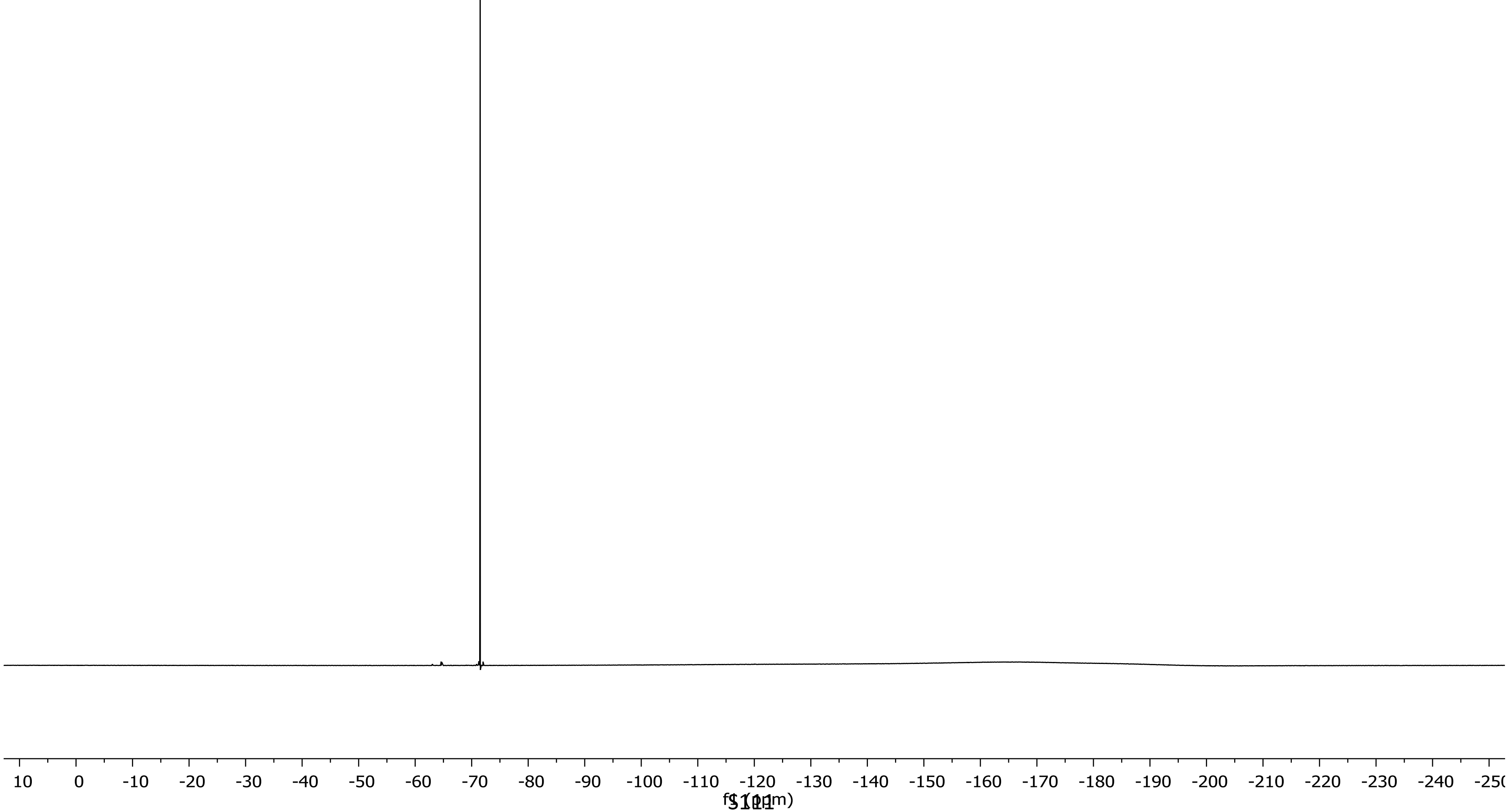

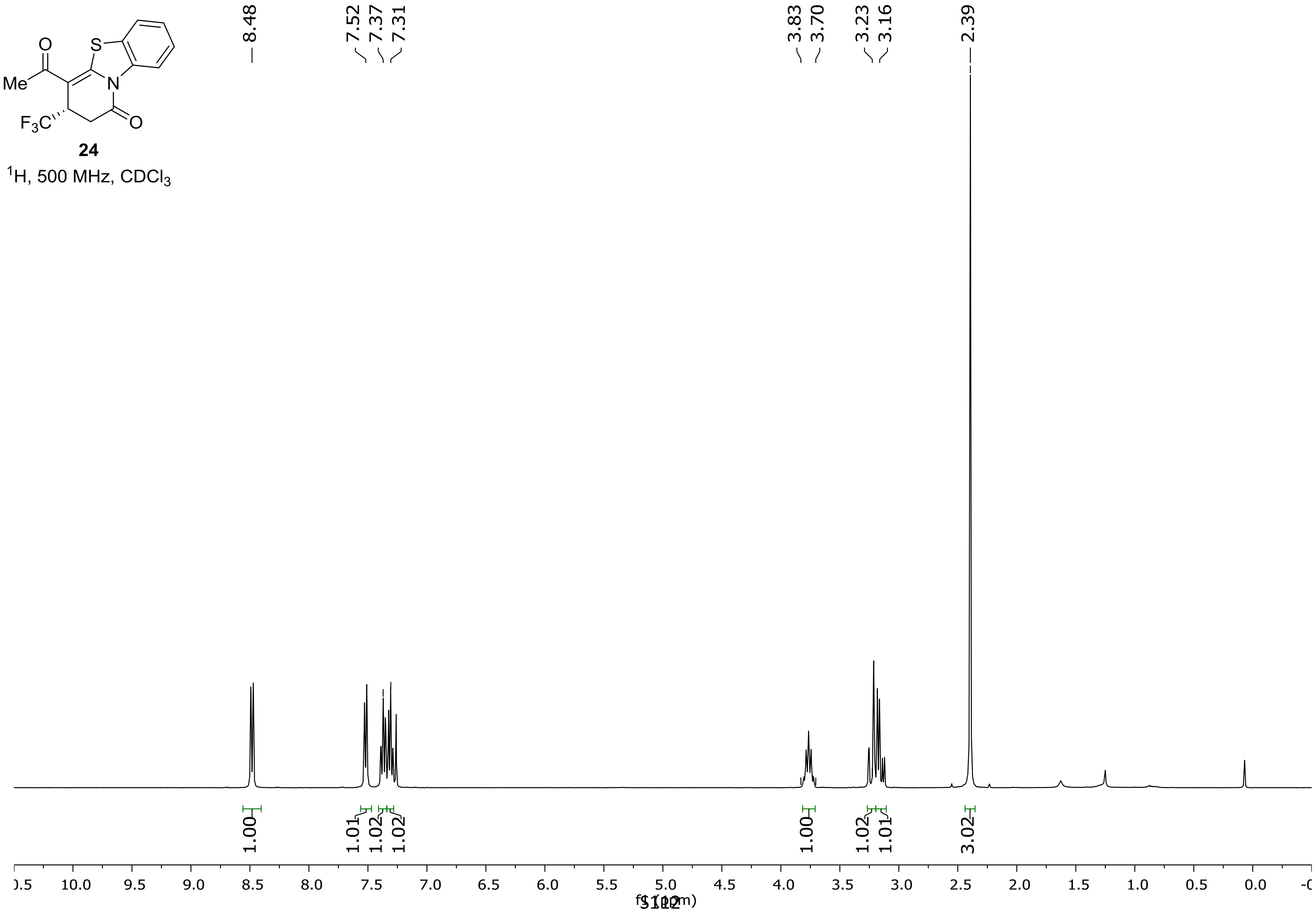

24

^{1}H, 500 MHz, $CDCl_3$

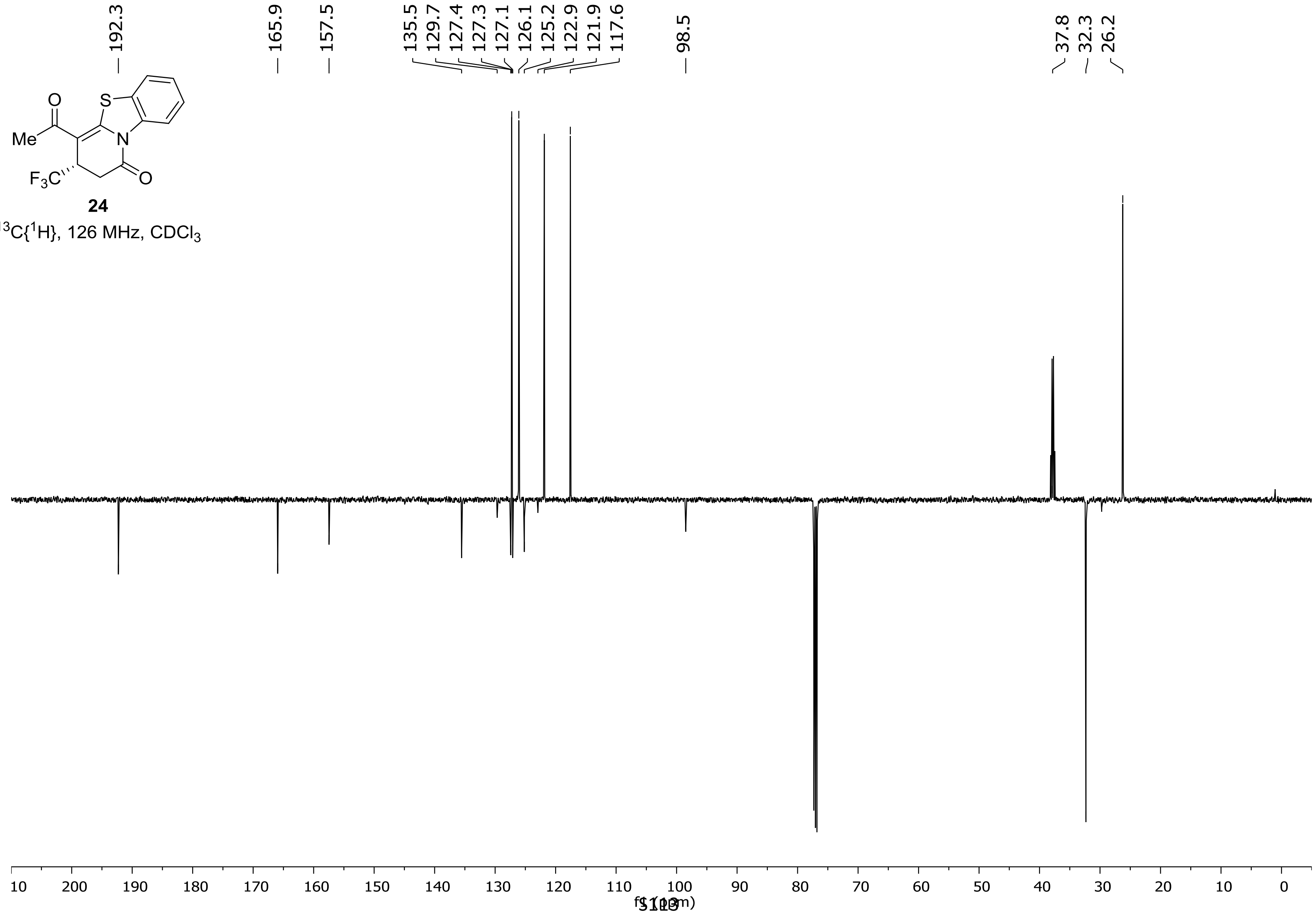

24

$^{13}C\{^{1}H\}$, 126 MHz, $CDCl_3$

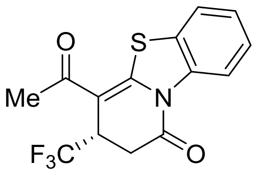

24

$^{19}F\{^{1}H\}$, 376 MHz, $CDCl_3$

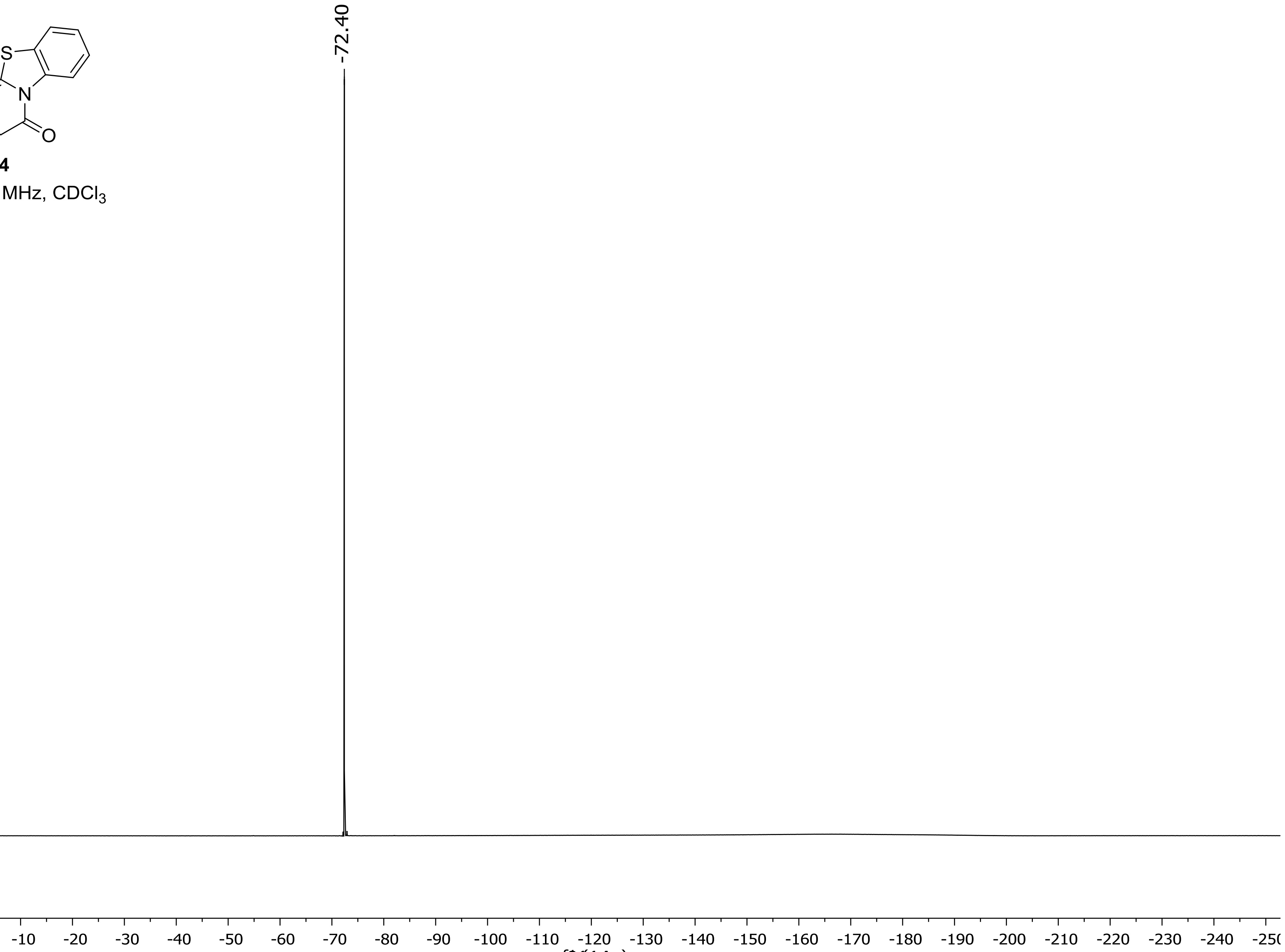

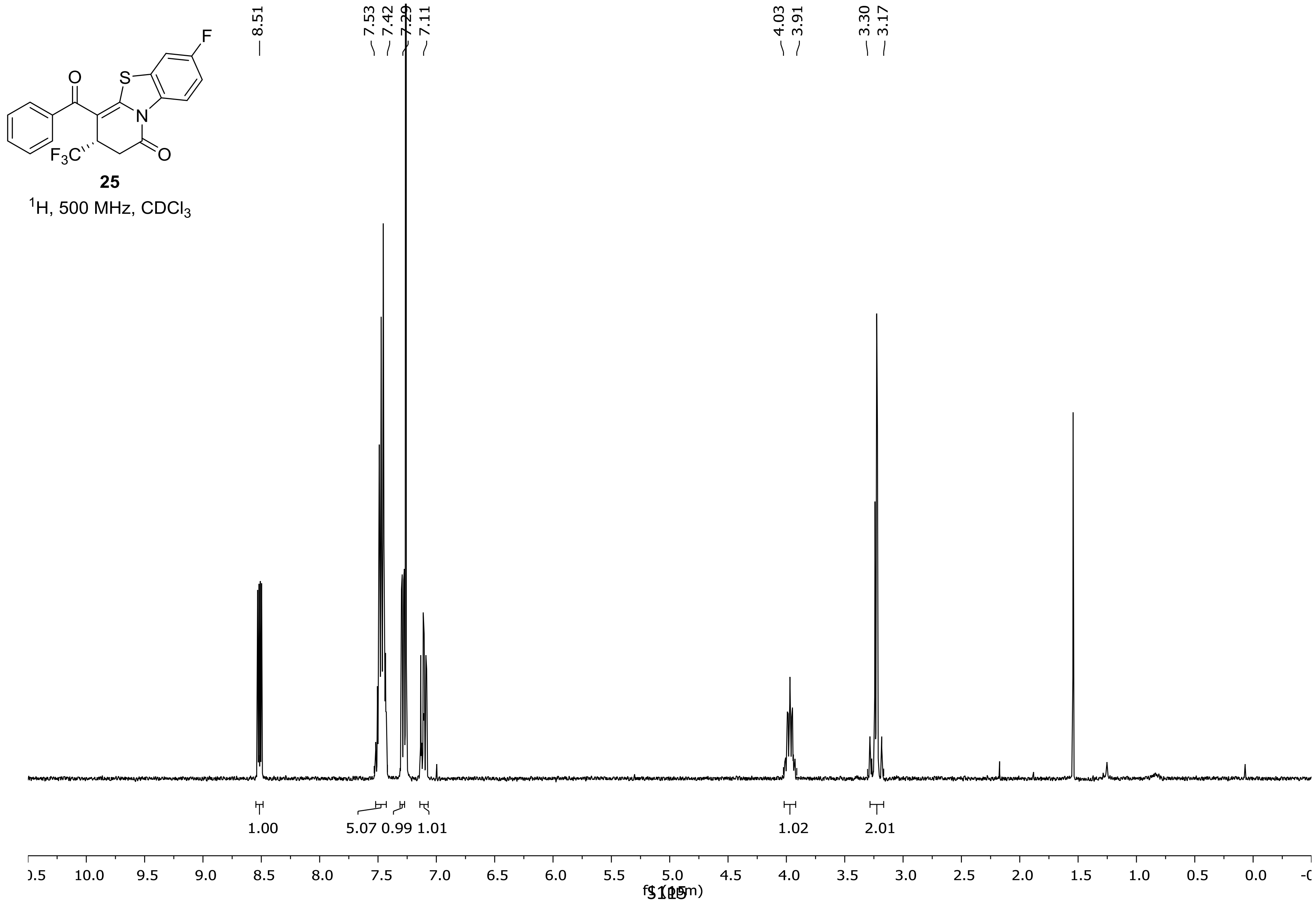

F
S
N
O
O
F_3C
25
^1H, 500 MHz, $CDCl_3$
8.51
7.53
7.42
7.29
7.11
4.03
3.91
3.30
3.17
1.00
5.07
0.99
1.01
1.02
2.01
0.5
10.0
9.5
9.0
8.5
8.0
7.5
7.0
6.5
6.0
5.5
5.0
4.5
4.0
3.5
3.0
2.5
2.0
1.5
1.0
0.5
0.0
f1 (ppm)

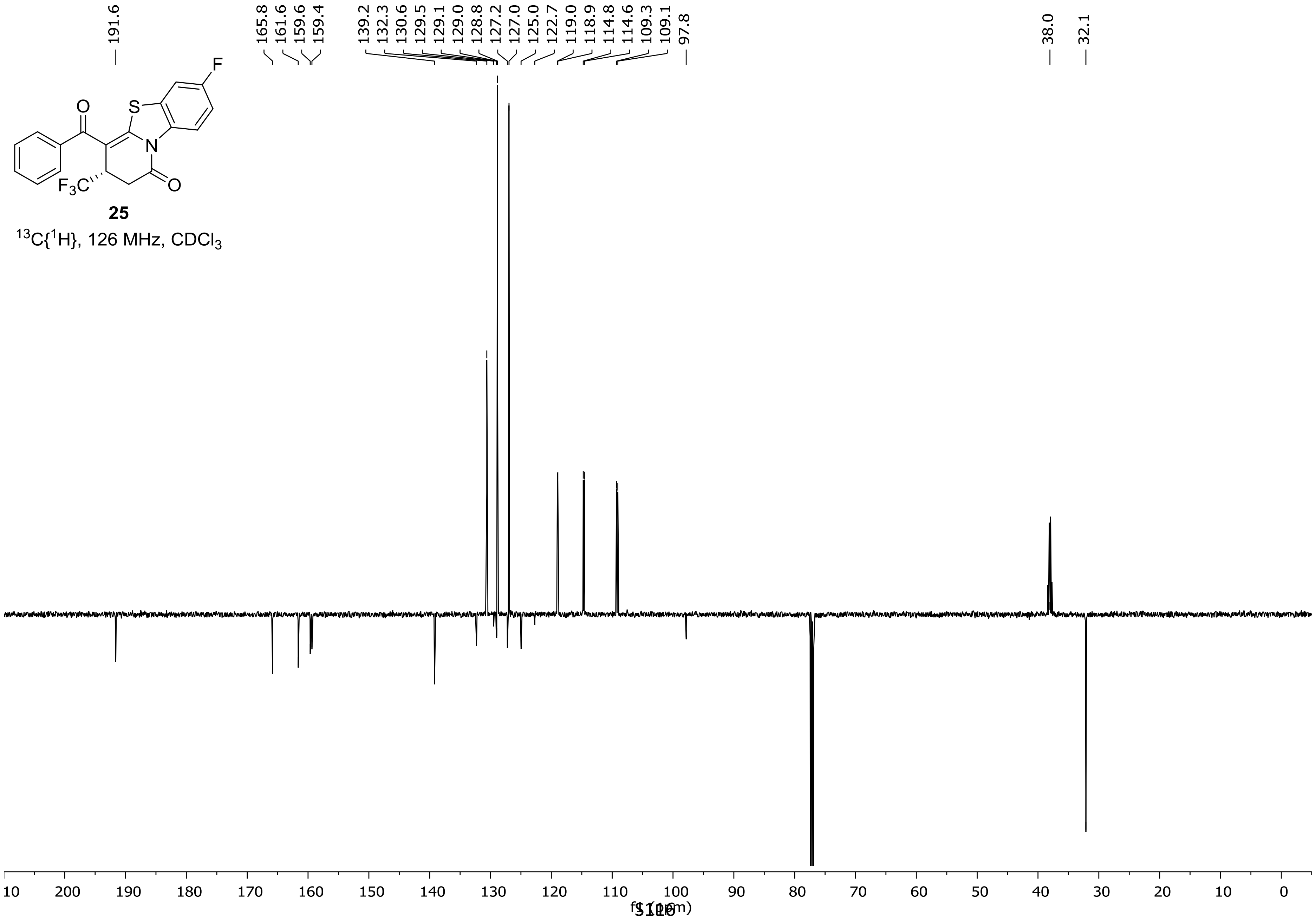

25
$^{13}C\{^1H\}$, 126 MHz, $CDCl_3$
191.6
165.8
161.6
159.6
159.4
139.2
132.3
130.6
129.5
129.1
129.0
128.8
127.2
127.0
125.0
122.7
119.0
118.9
114.8
114.6
109.3
109.1
97.8
38.0
32.1
f1 (ppm)

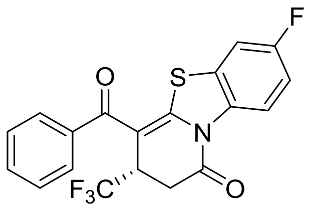

25

$^{19}F\{^{1}H\}$, 376 MHz, $CDCl_3$

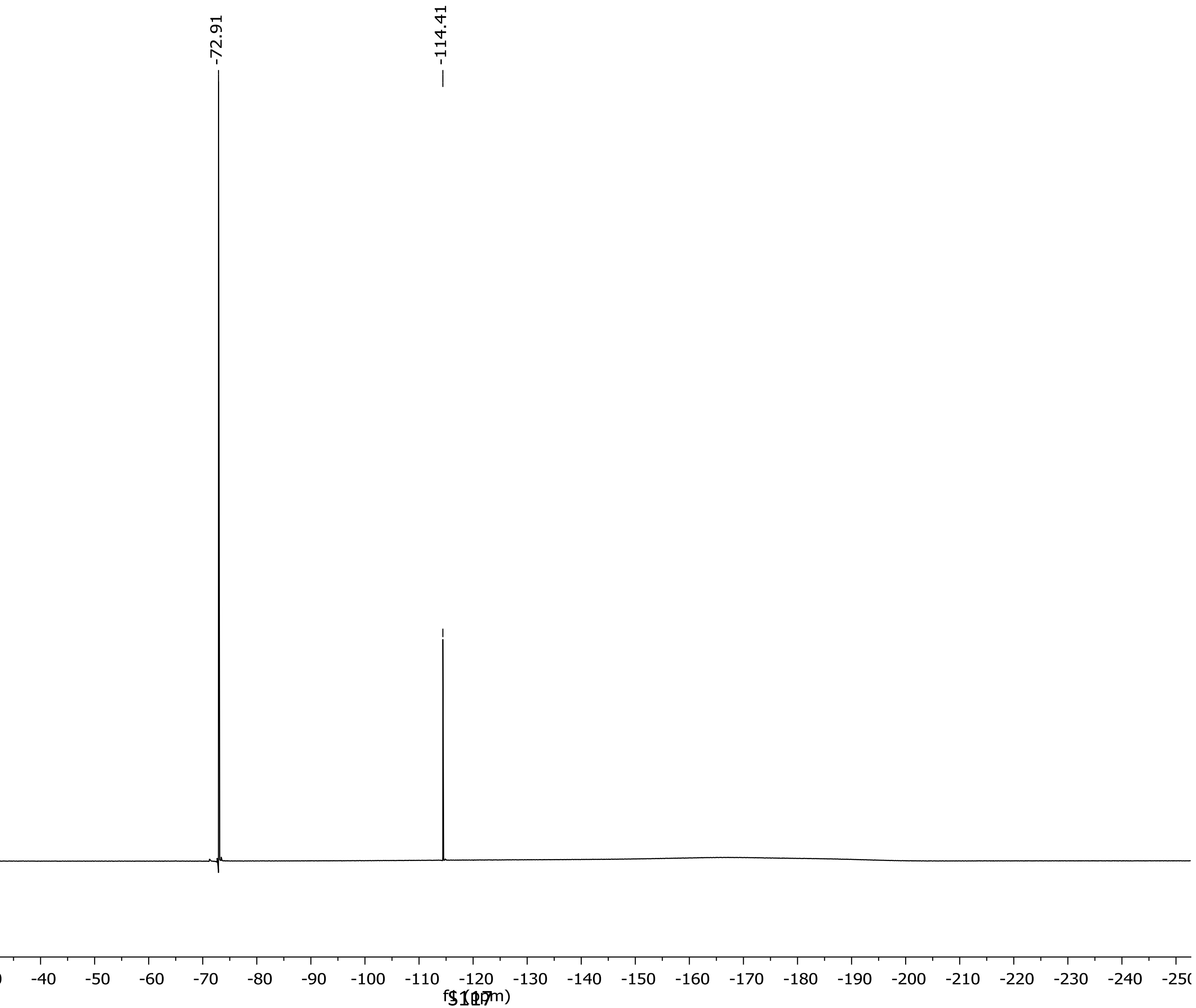

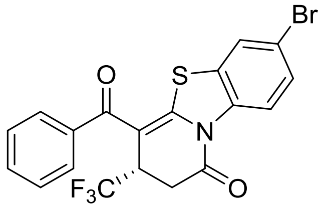

26

^{1}H, 500 MHz, $CDCl_3$

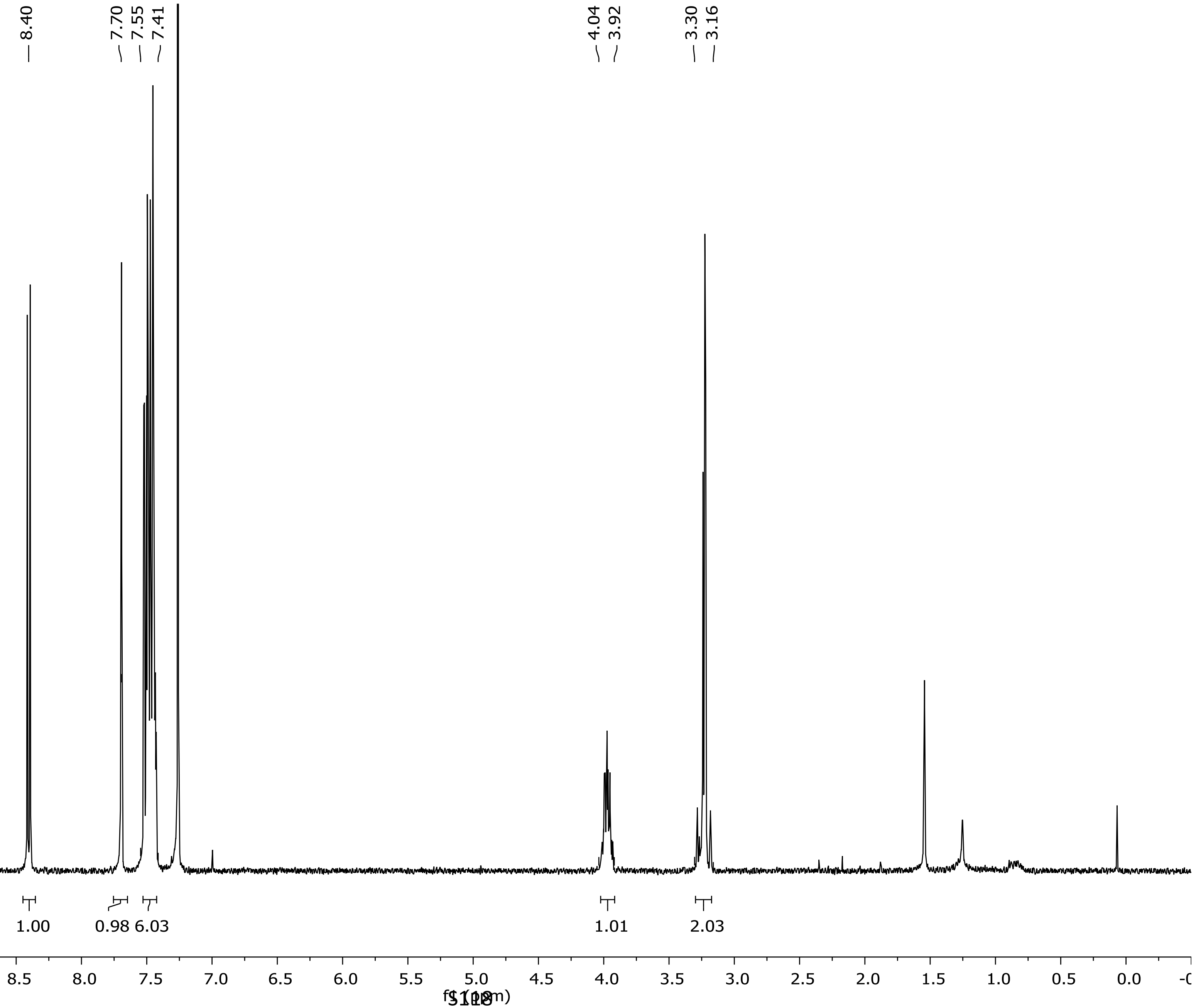

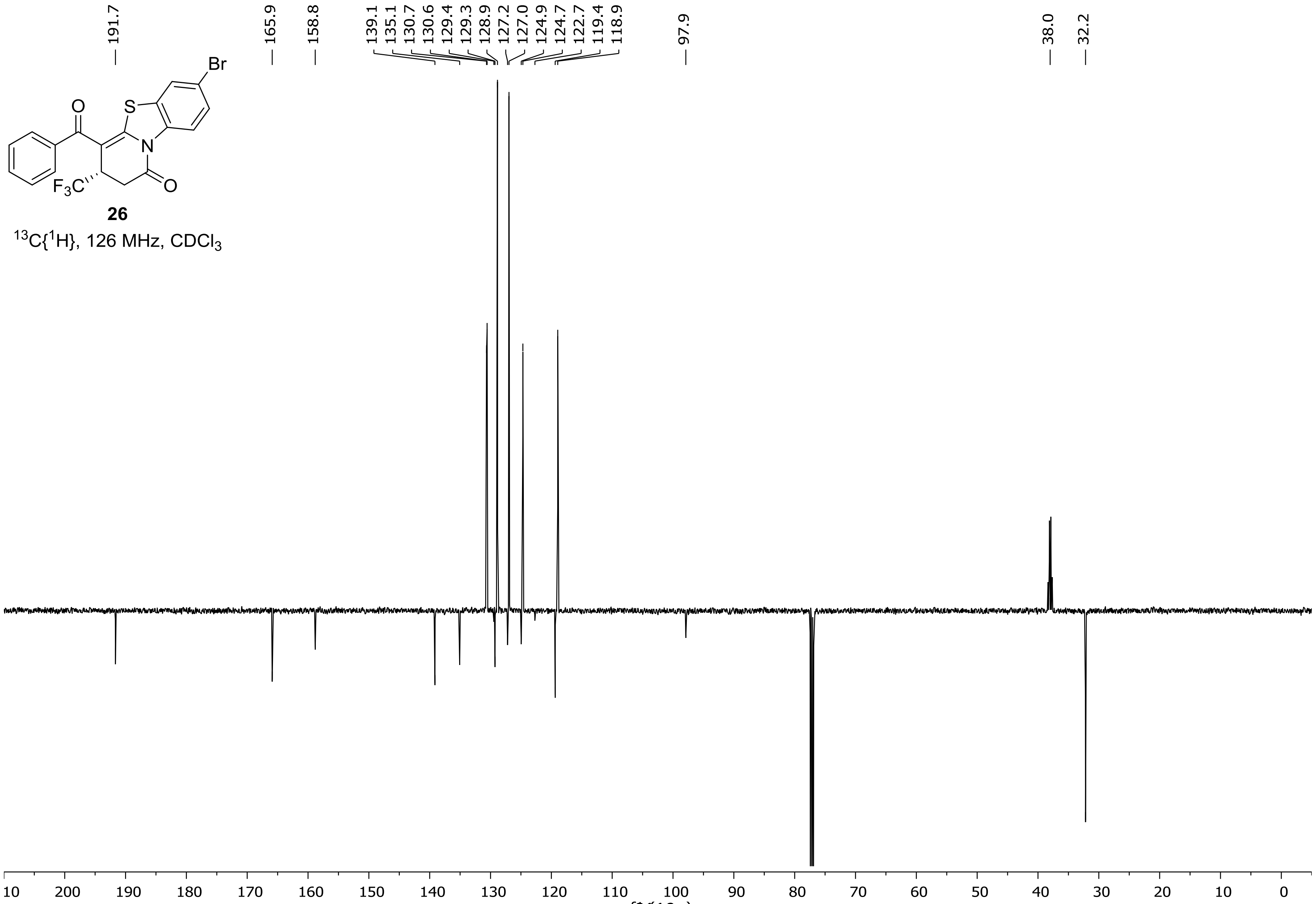

26

$^{13}C\{^{1}H\}$, 126 MHz, $CDCl_3$

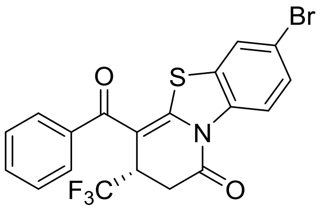

26

$^{19}F\{^{1}H\}$, 376 MHz, $CDCl_3$

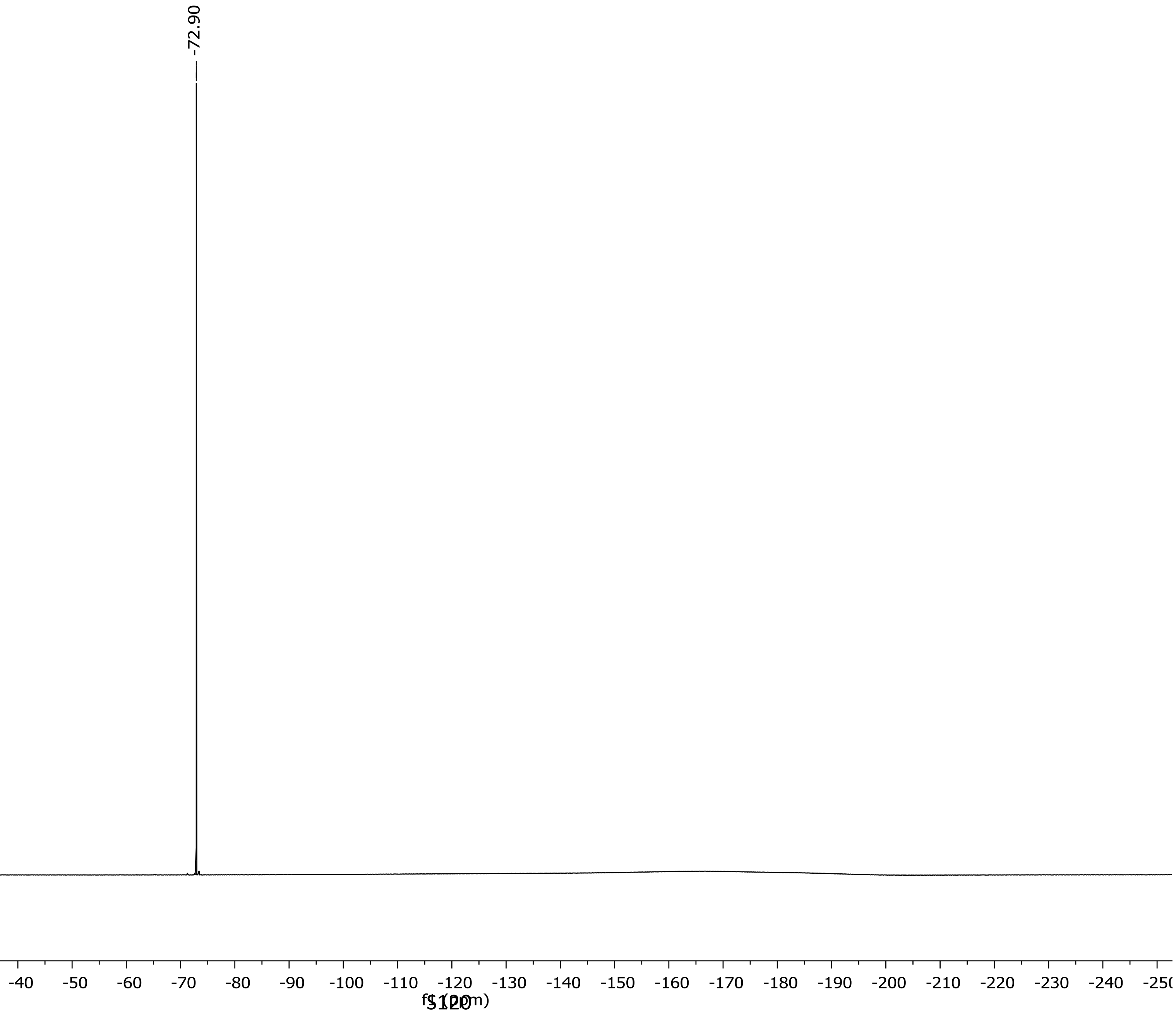

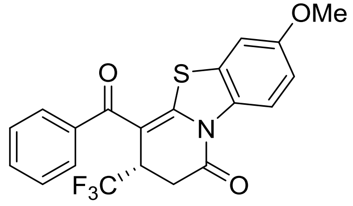

27

^{1}H, 500 MHz, $CDCl_3$

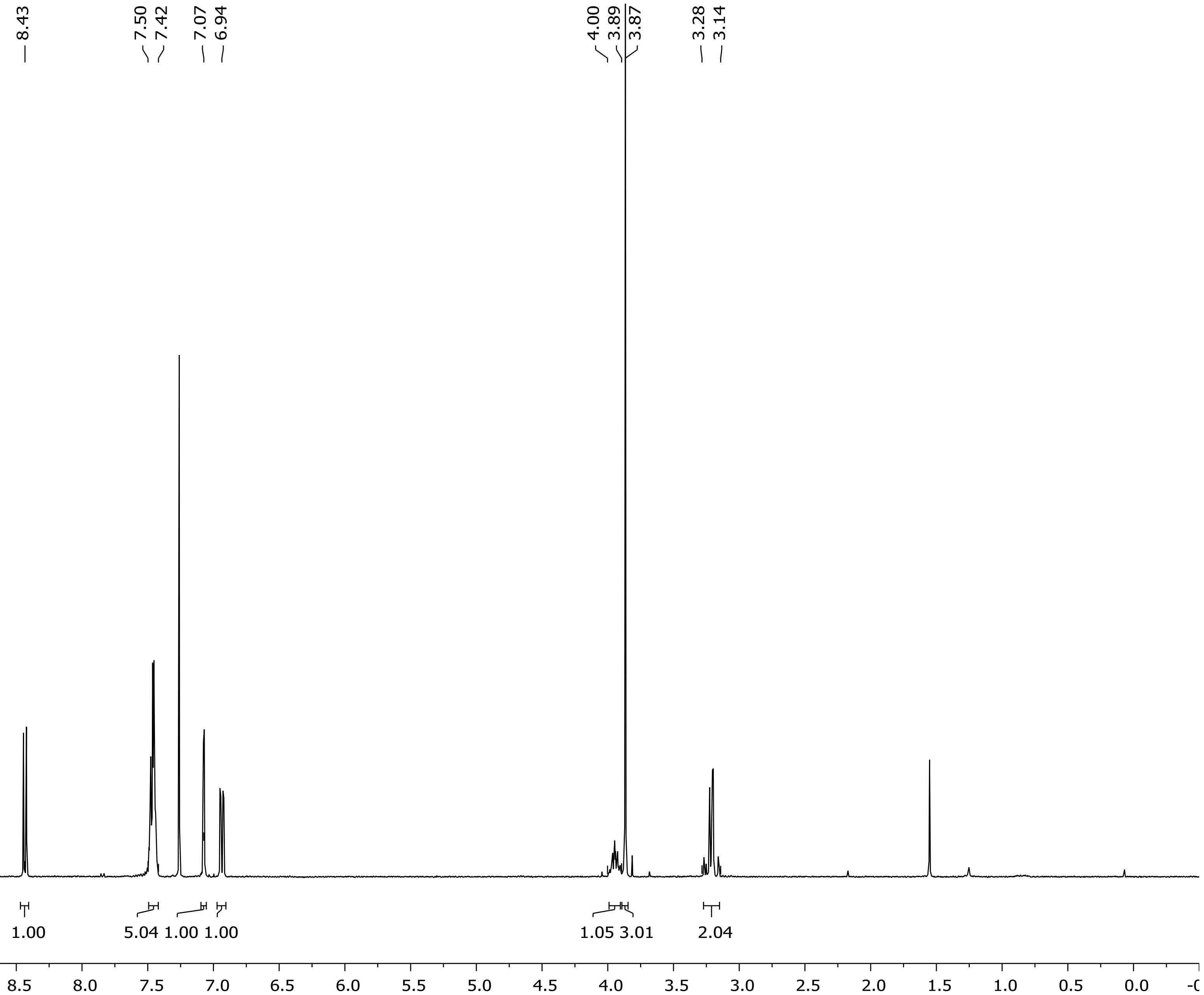

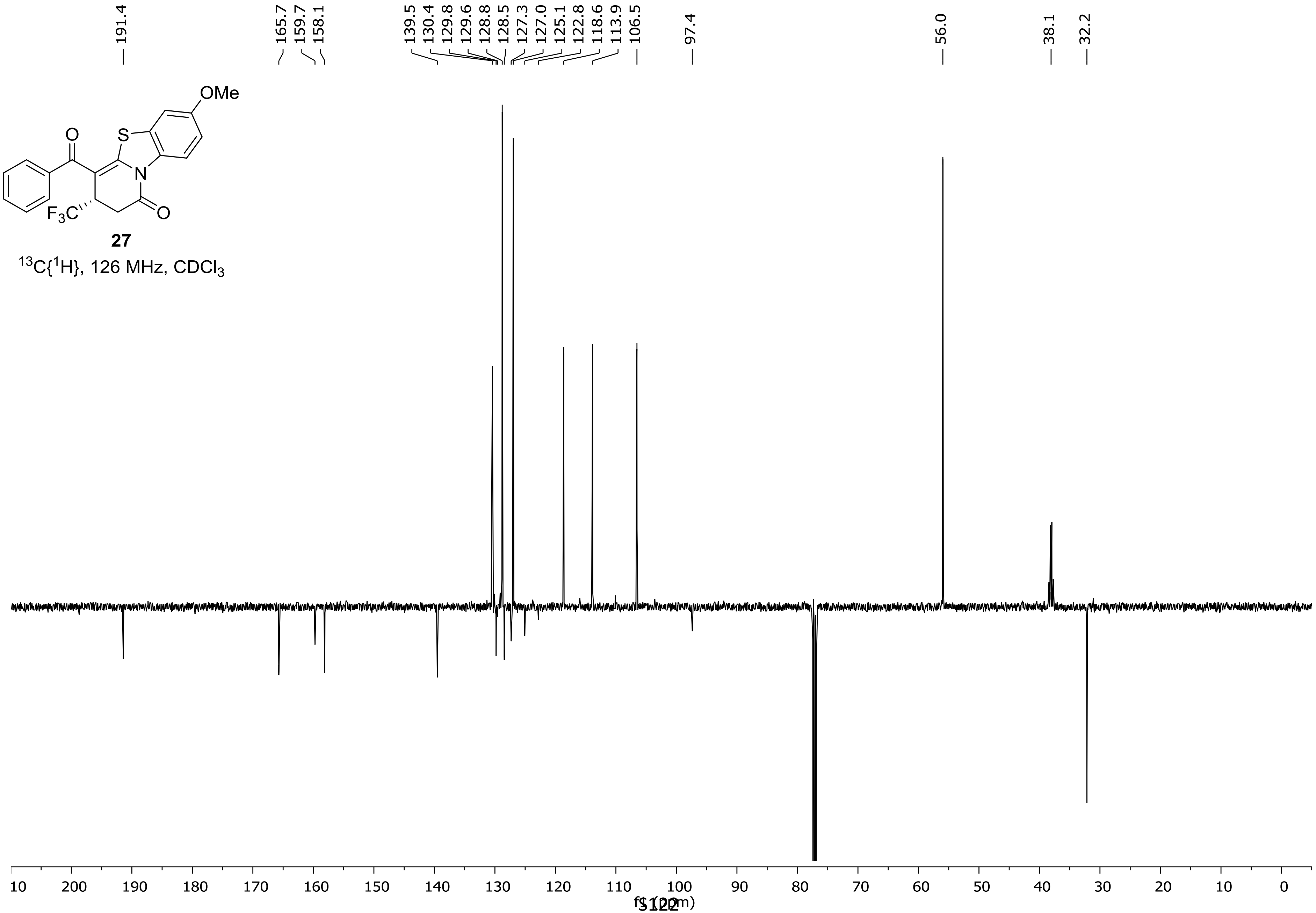

27

$^{13}C\{^{1}H\}$, 126 MHz, $CDCl_3$

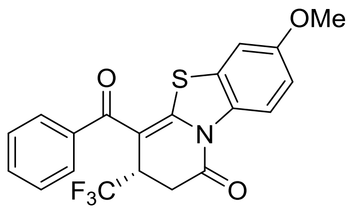

27

$^{19}F\{^1H\}$, 376 MHz, $CDCl_3$

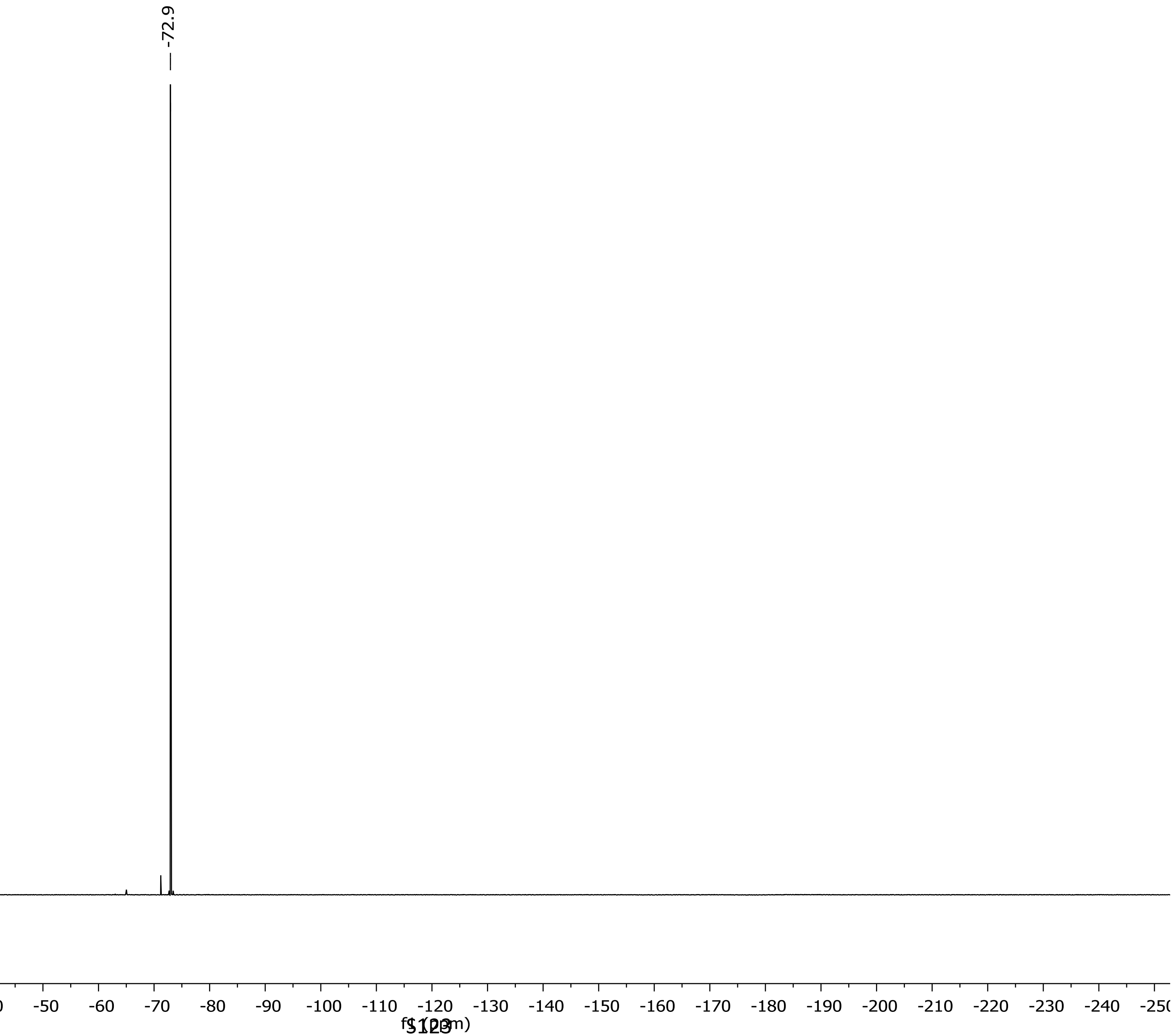

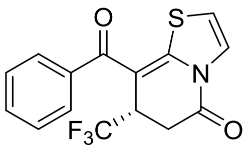

28

^1H, 500 MHz, $CDCl_3$

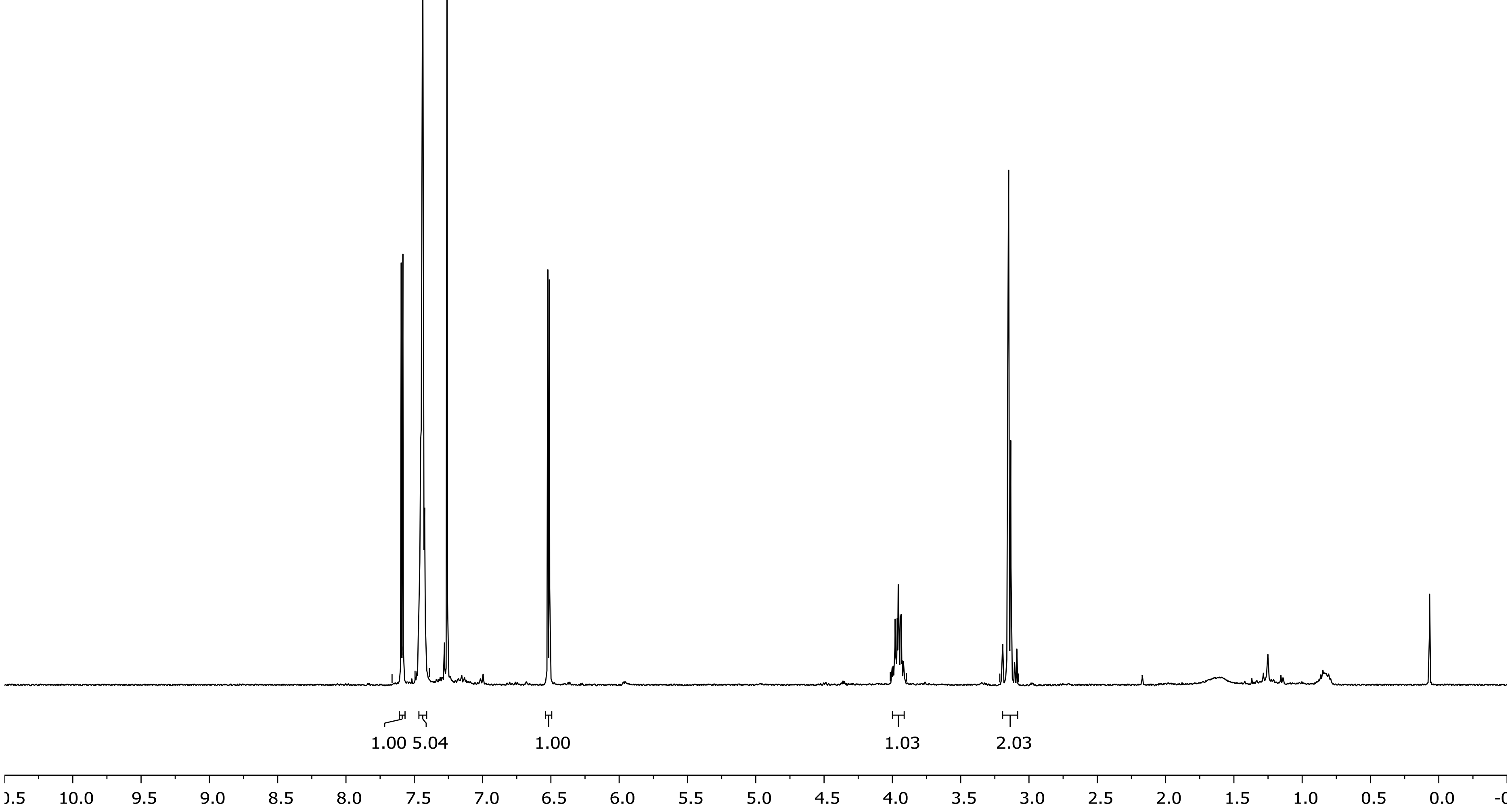

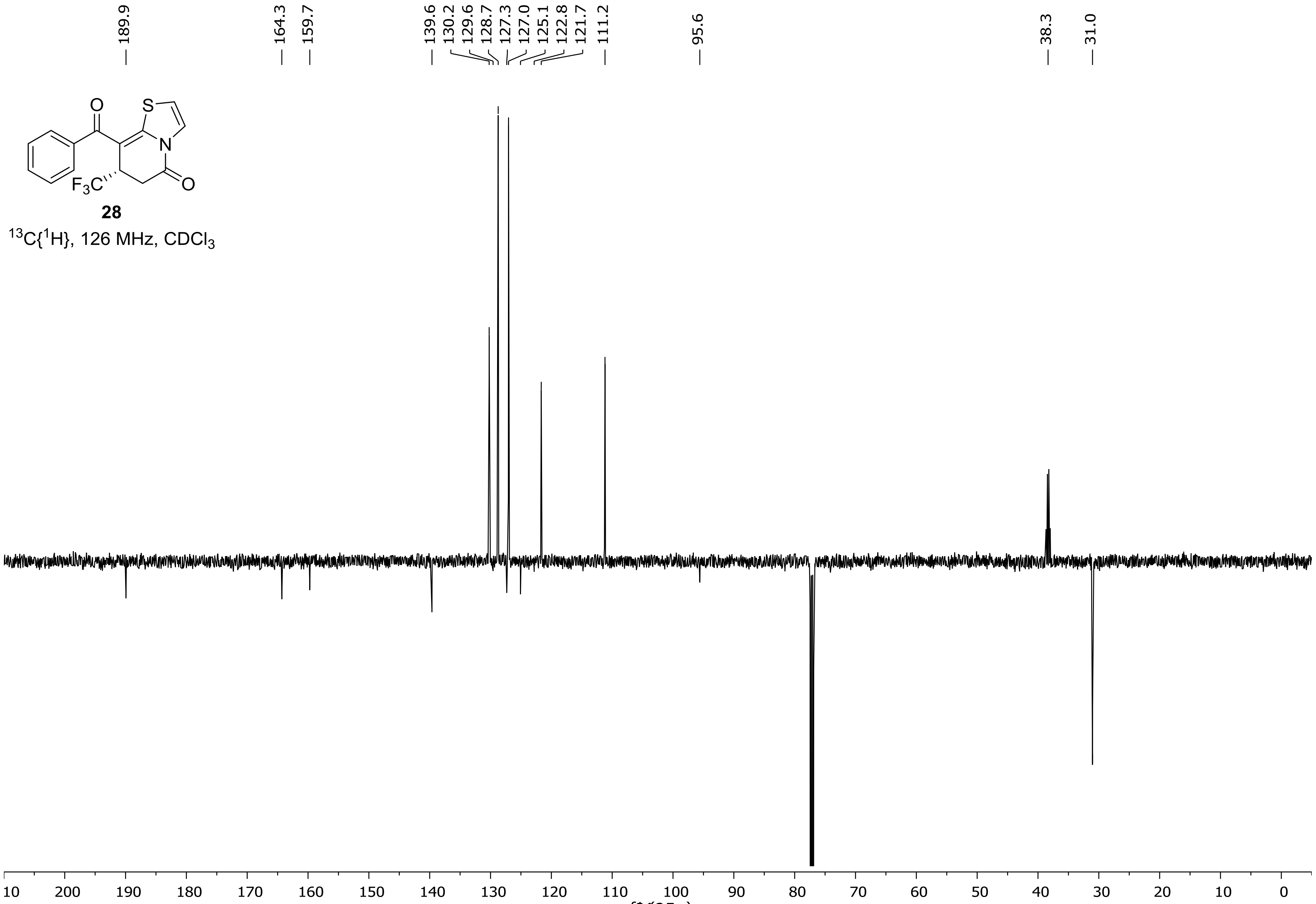

28

$^{13}C\{^{1}H\}$, 126 MHz, $CDCl_3$

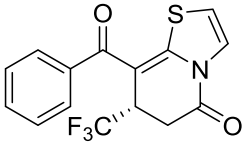

28

$^{19}F\{^{1}H\}$, 376 MHz, $CDCl_3$

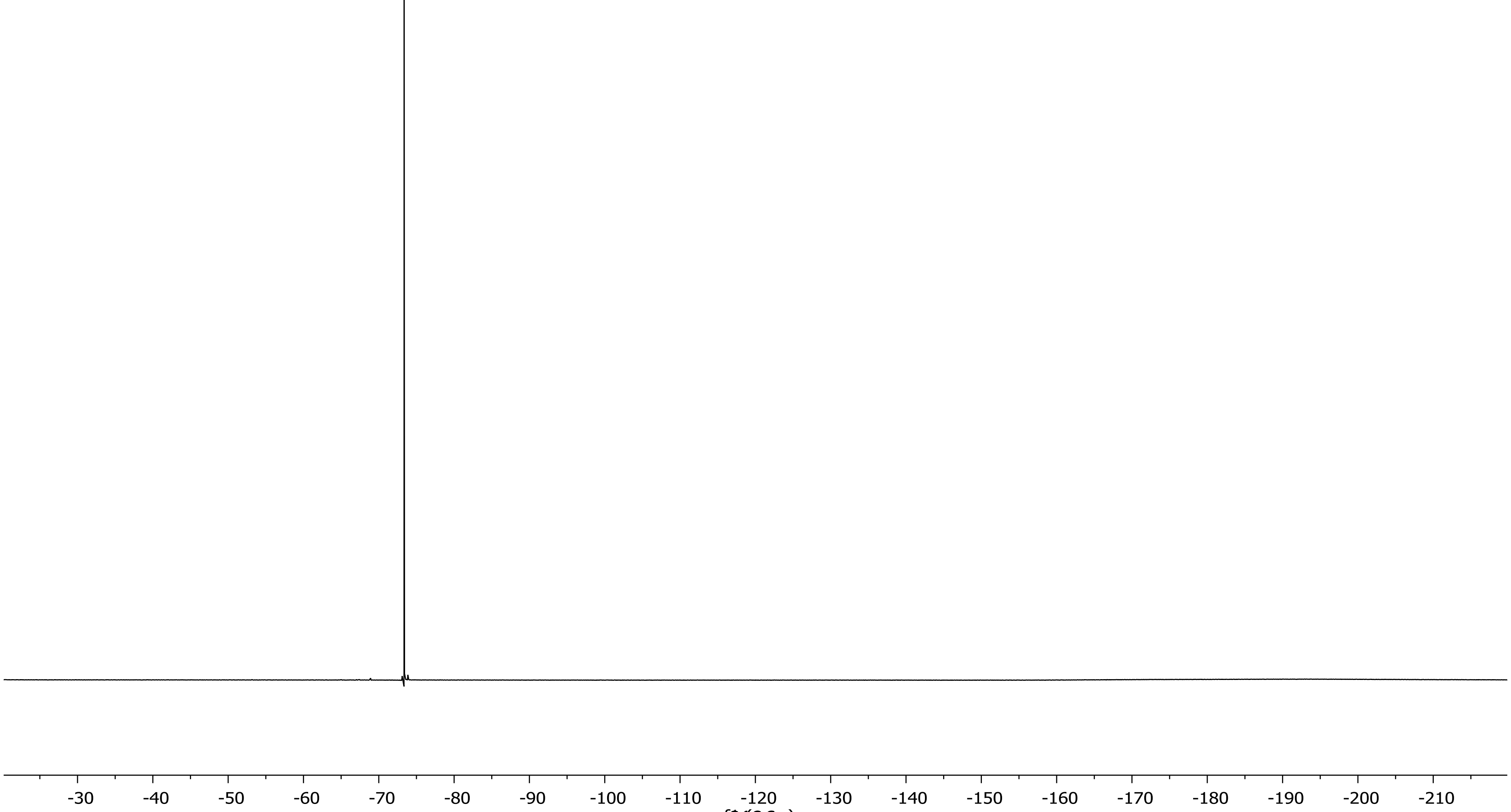

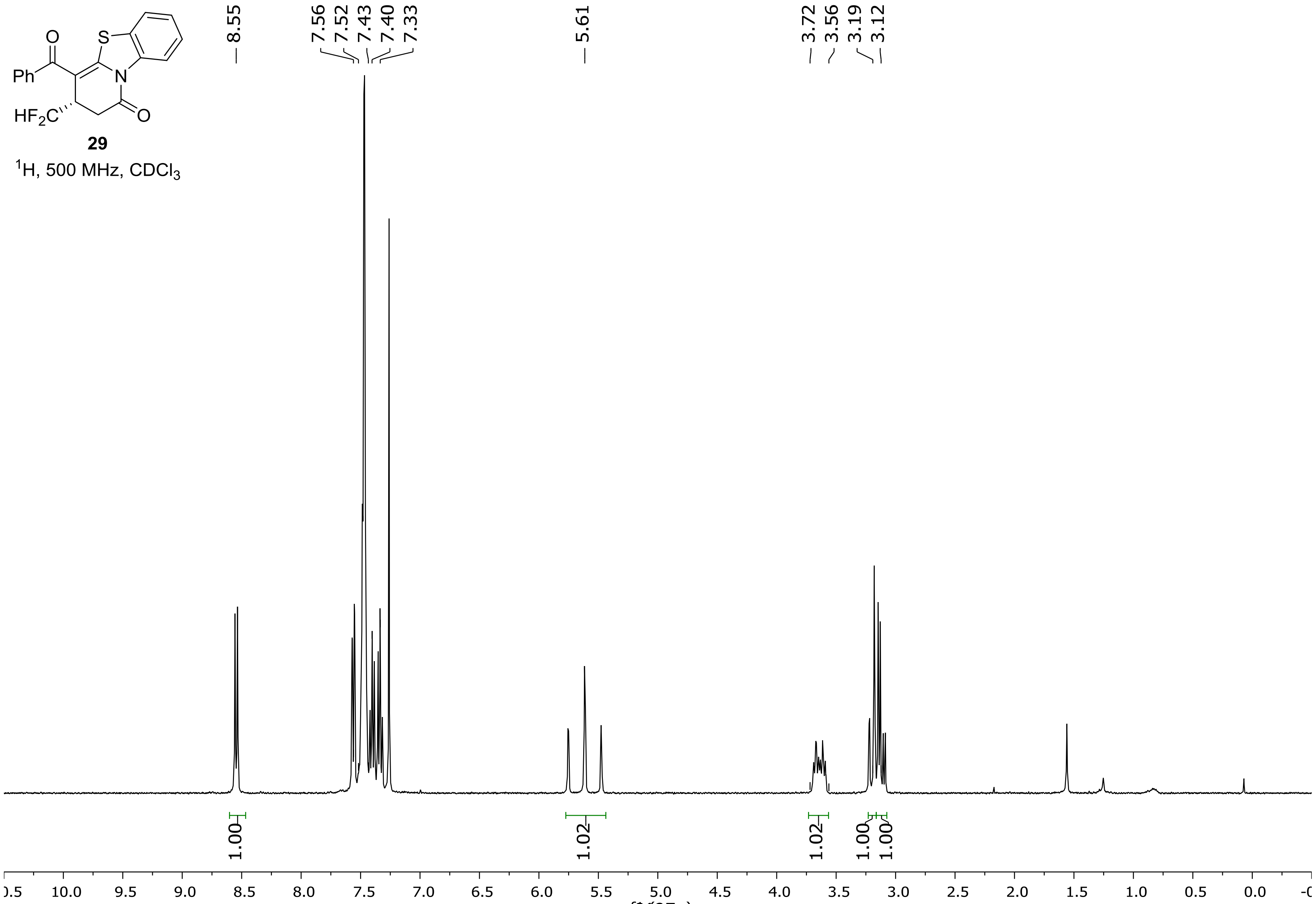

Ph
O
S
N
O
HF2C
29
1H, 500 MHz, CDCl3
8.55
7.56
7.52
7.43
7.40
7.33
5.61
3.72
3.56
3.19
3.12
1.00
1.02
1.02
1.00
1.00
10.0
9.5
9.0
8.5
8.0
7.5
7.0
6.5
6.0
5.5
5.0
4.5
4.0
3.5
3.0
2.5
2.0
1.5
1.0
0.5
0.0
f1 (ppm)

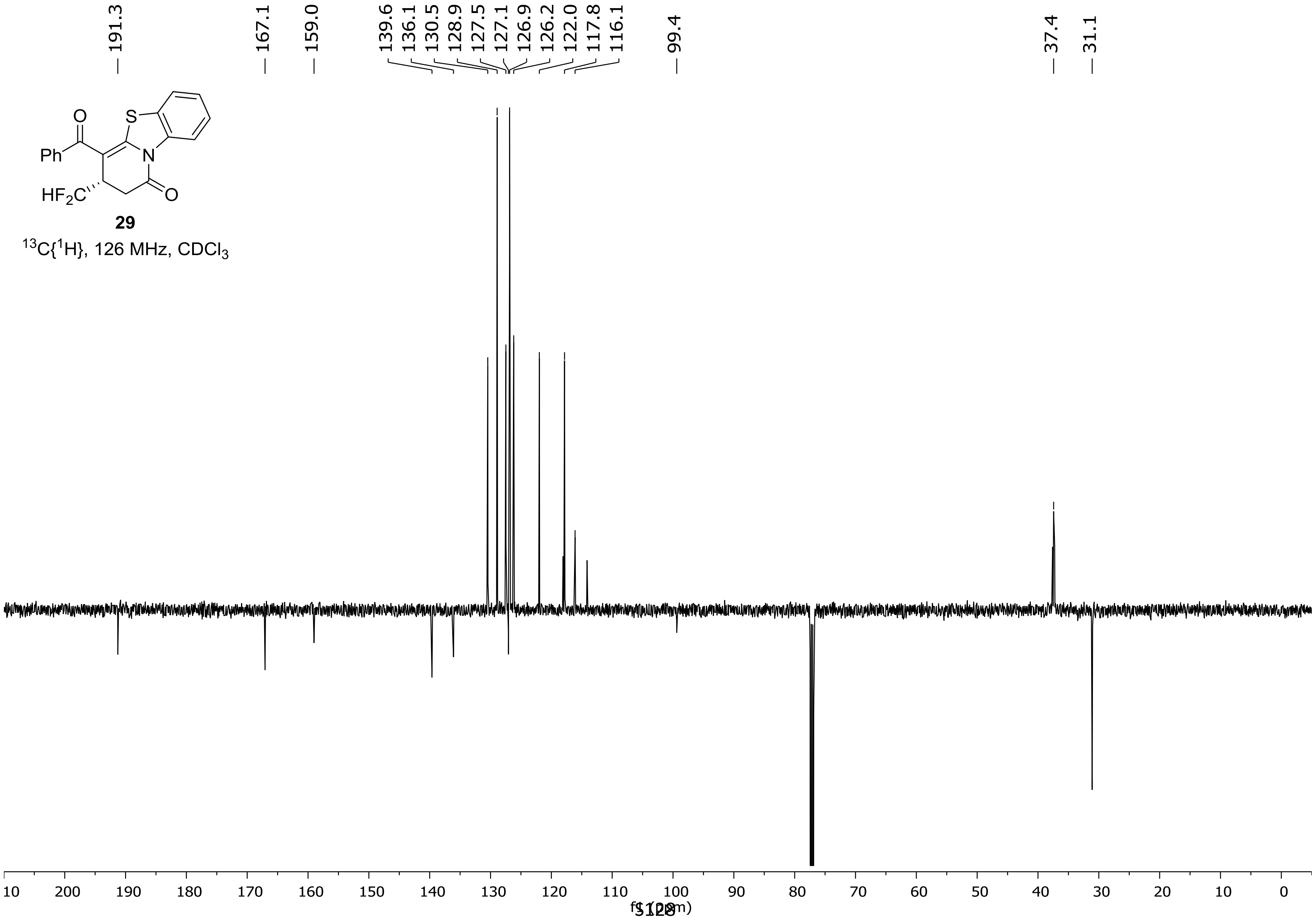
191.3
167.1
159.0
139.6
136.1
130.5
128.9
127.5
127.1
126.9
126.2
122.0
117.8
116.1
99.4
37.4
31.1
O
S
Ph
N
HF_2C
O
29
$^{13}C\{^{1}H\}$, 126 MHz, $CDCl_3$
10 200 190 180 170 160 150 140 130 120 110 100 90 80 70 60 50 40 30 20 10 0
f1 (ppm)

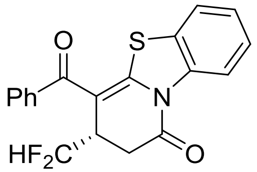

29

$^{19}F\{^{1}H\}$, 376 MHz, $CDCl_3$

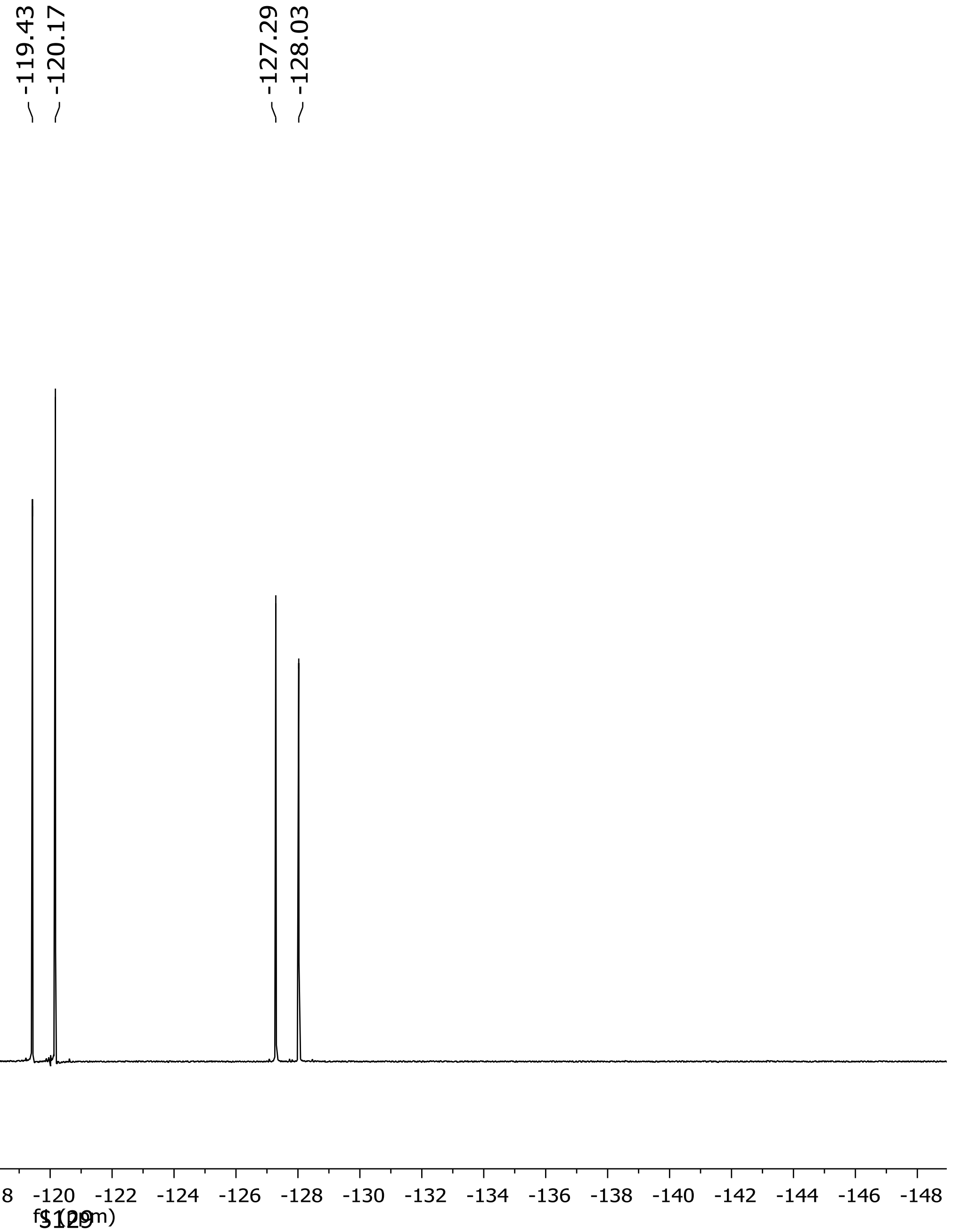

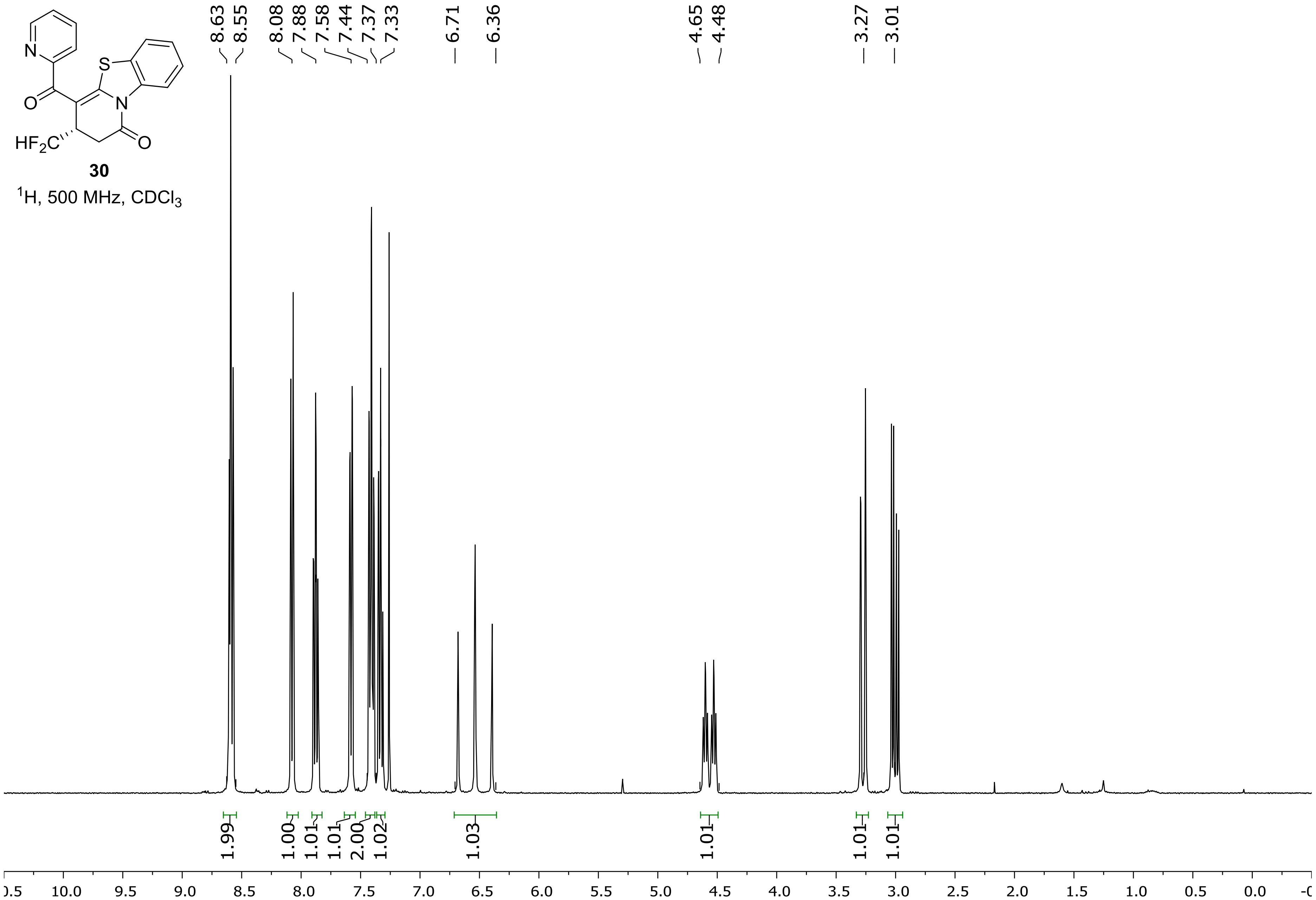

30
1H, 500 MHz, CDCl3
8.63
8.55
8.08
7.88
7.58
7.44
7.37
7.33
6.71
6.36
4.65
4.48
3.27
3.01
1.99
1.00
1.01
1.01
2.00
1.02
1.03
1.01
1.01
1.01
f1 (ppm)

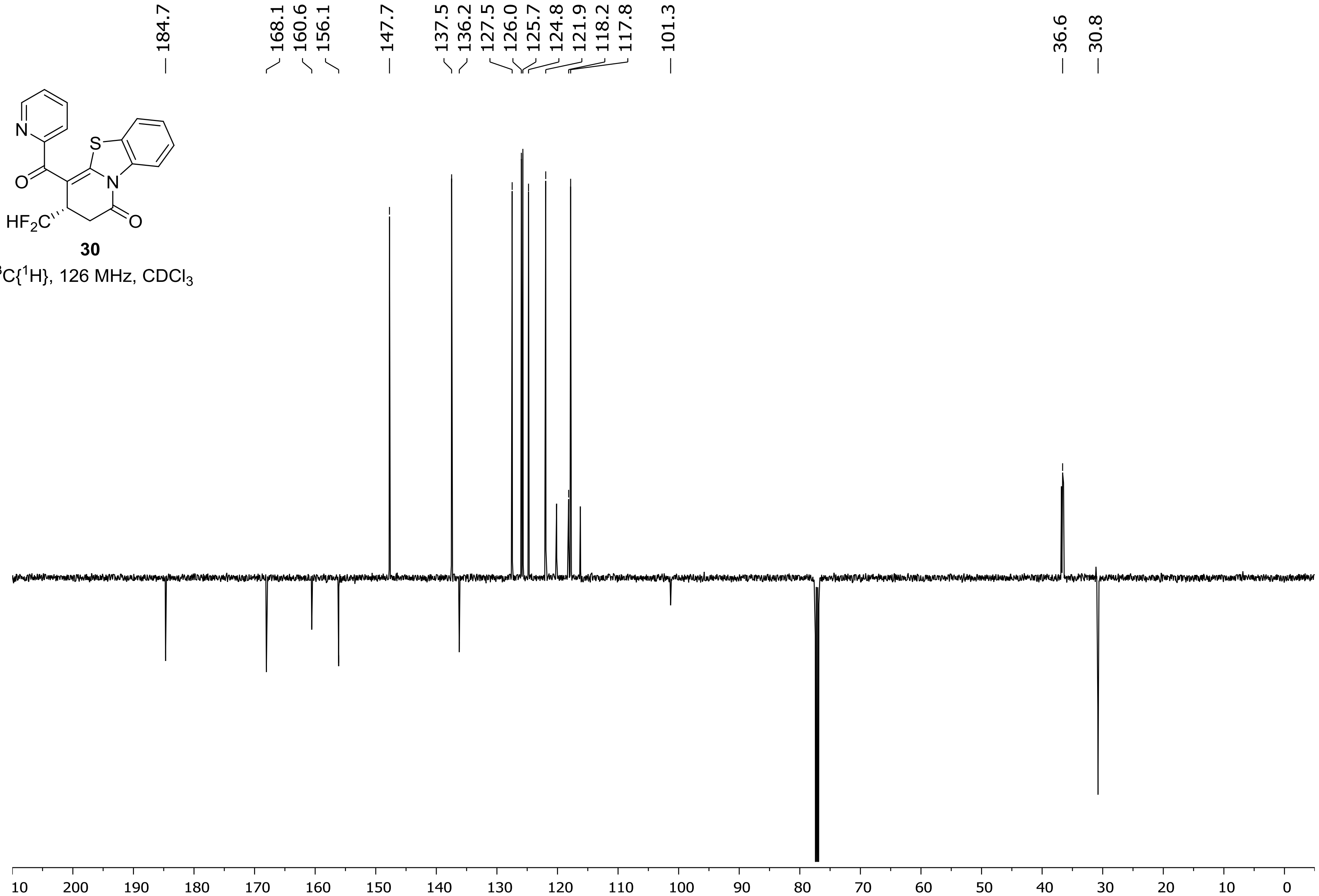

30

$^{13}C\{^1H\}$, 126 MHz, $CDCl_3$

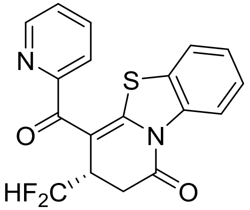

30

$^{19}F\{^1H\}$, 376 MHz, $CDCl_3$

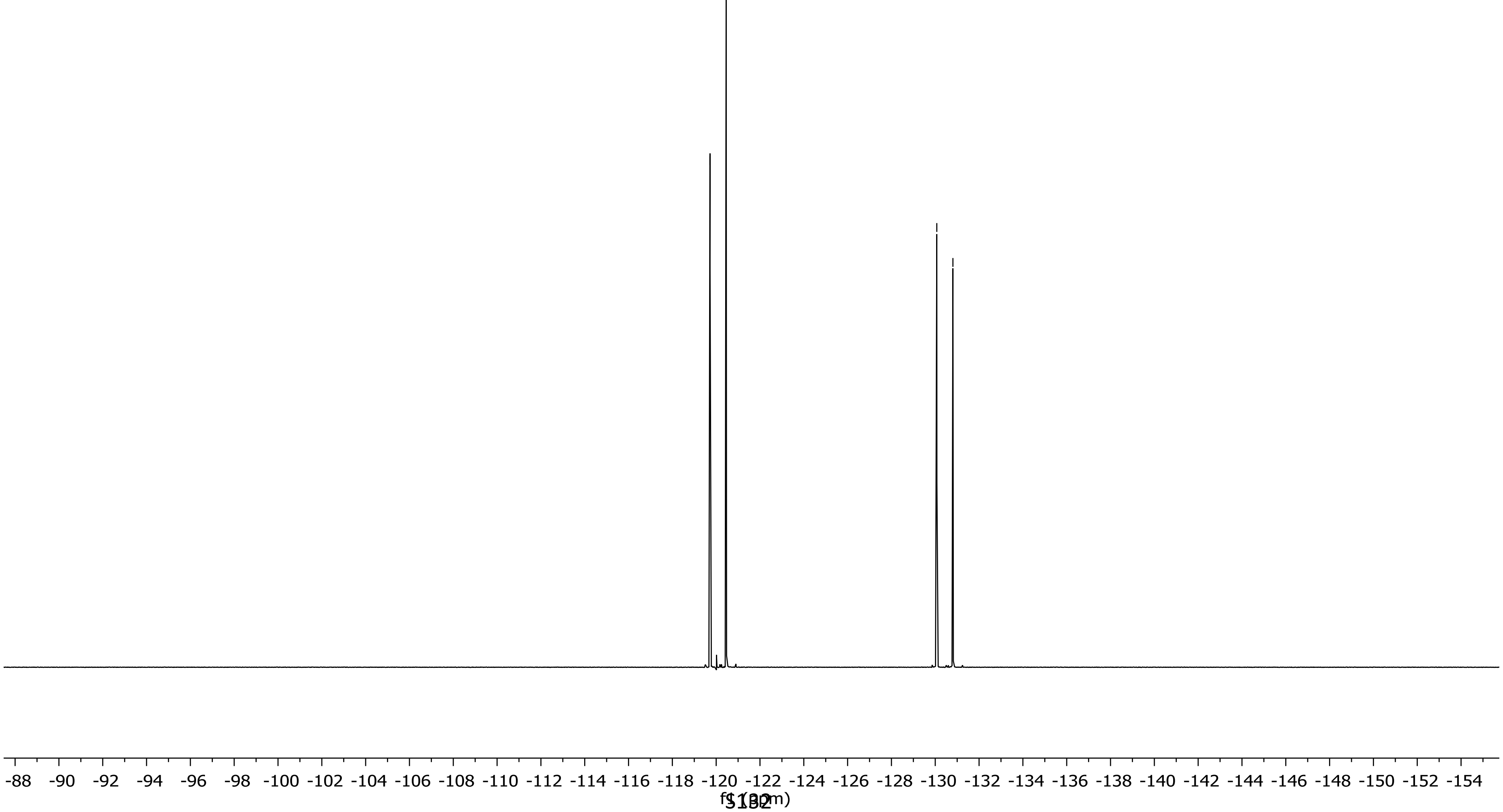

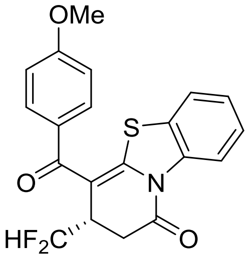

31

^{1}H, 500 MHz, $CDCl_3$

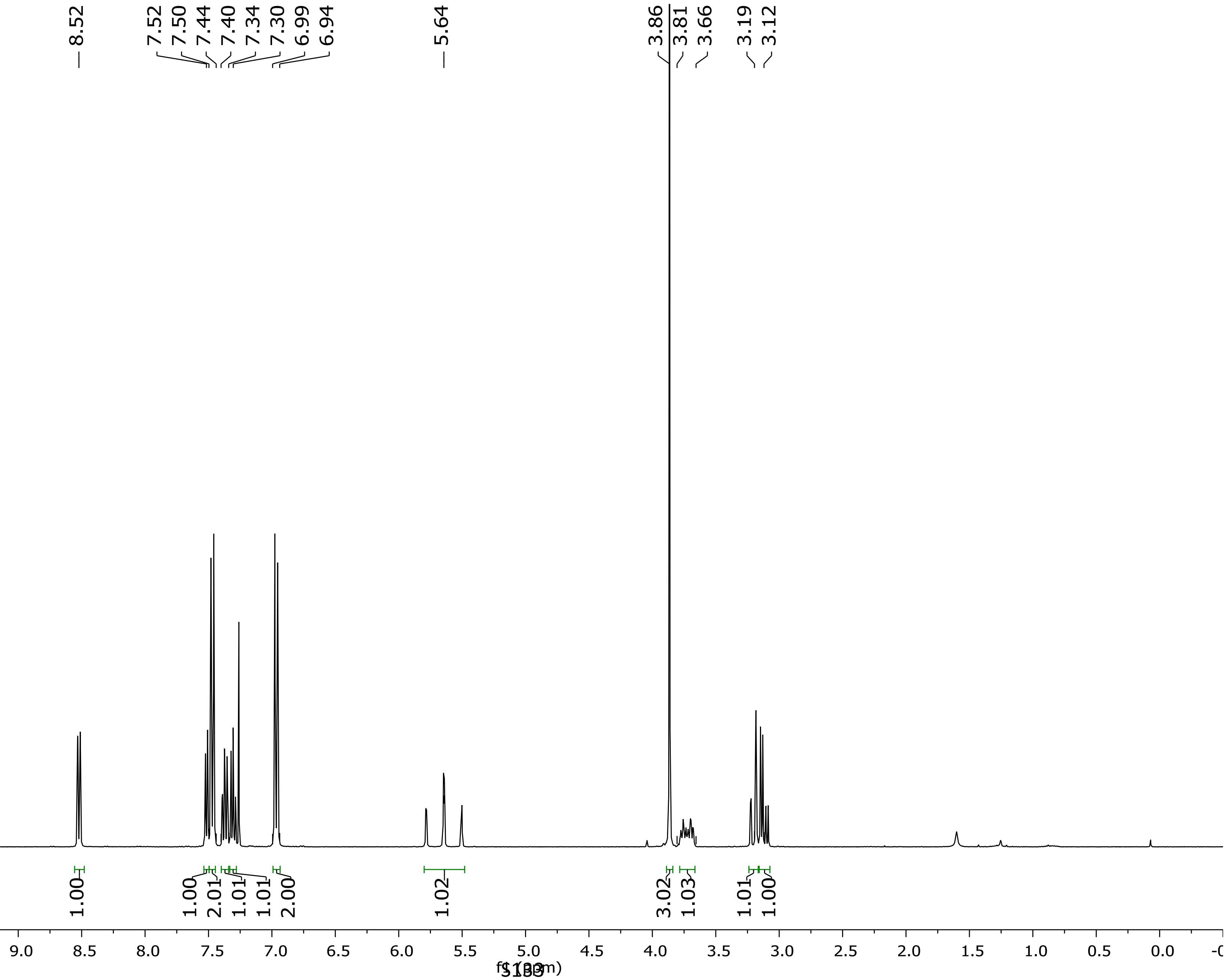

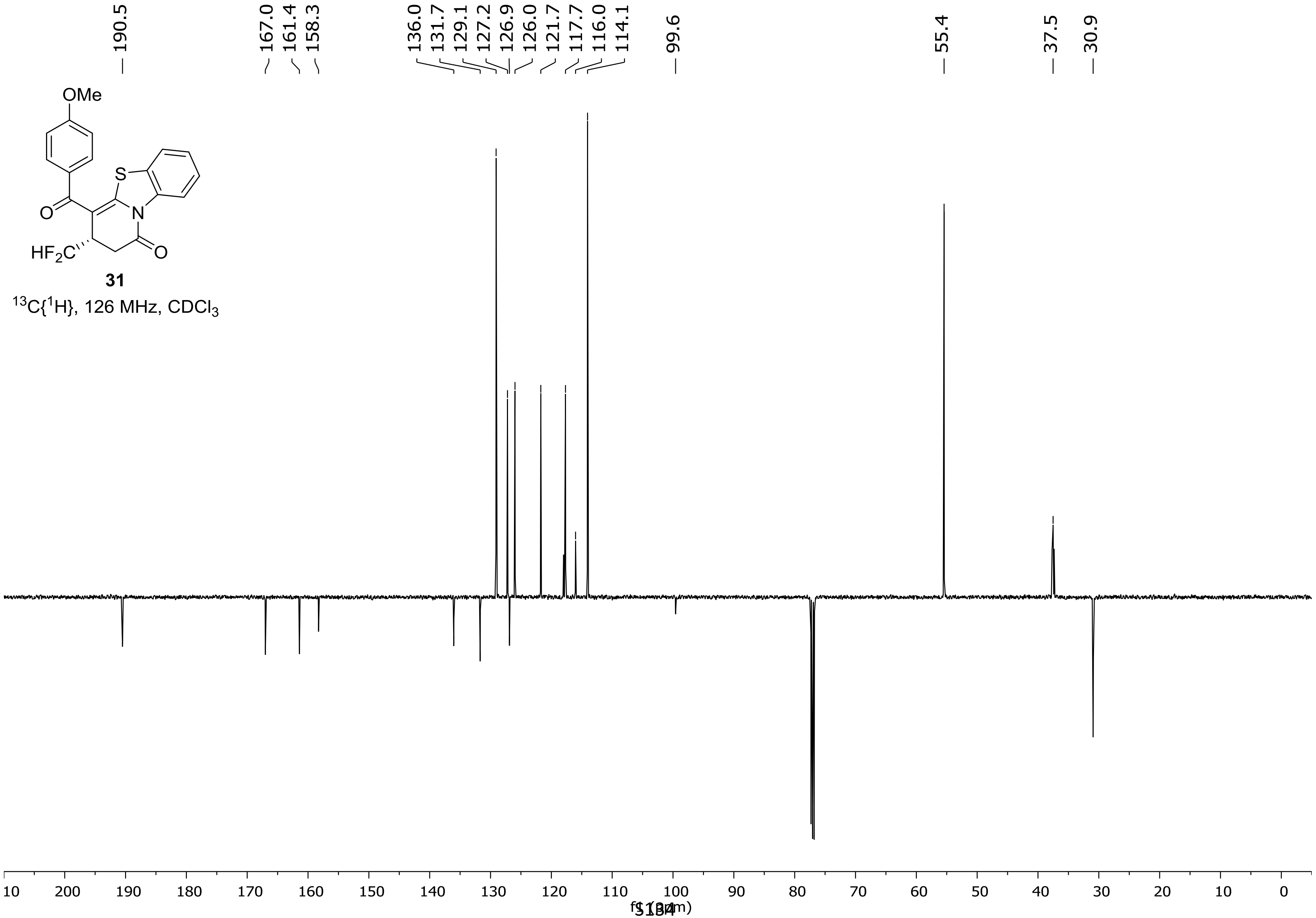
190.5
167.0
161.4
158.3
136.0
131.7
129.1
127.2
126.9
126.0
121.7
117.7
116.0
114.1
99.6
55.4
37.5
30.9
OMe
S
N
O
O
HF2C
31
13C{1H}, 126 MHz, CDCl3
10 200 190 180 170 160 150 140 130 120 110 100 90 80 70 60 50 40 30 20 10 0
f1 (ppm)

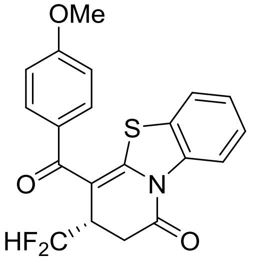

31

$^{19}F\{^{1}H\}$, 376 MHz, $CDCl_3$

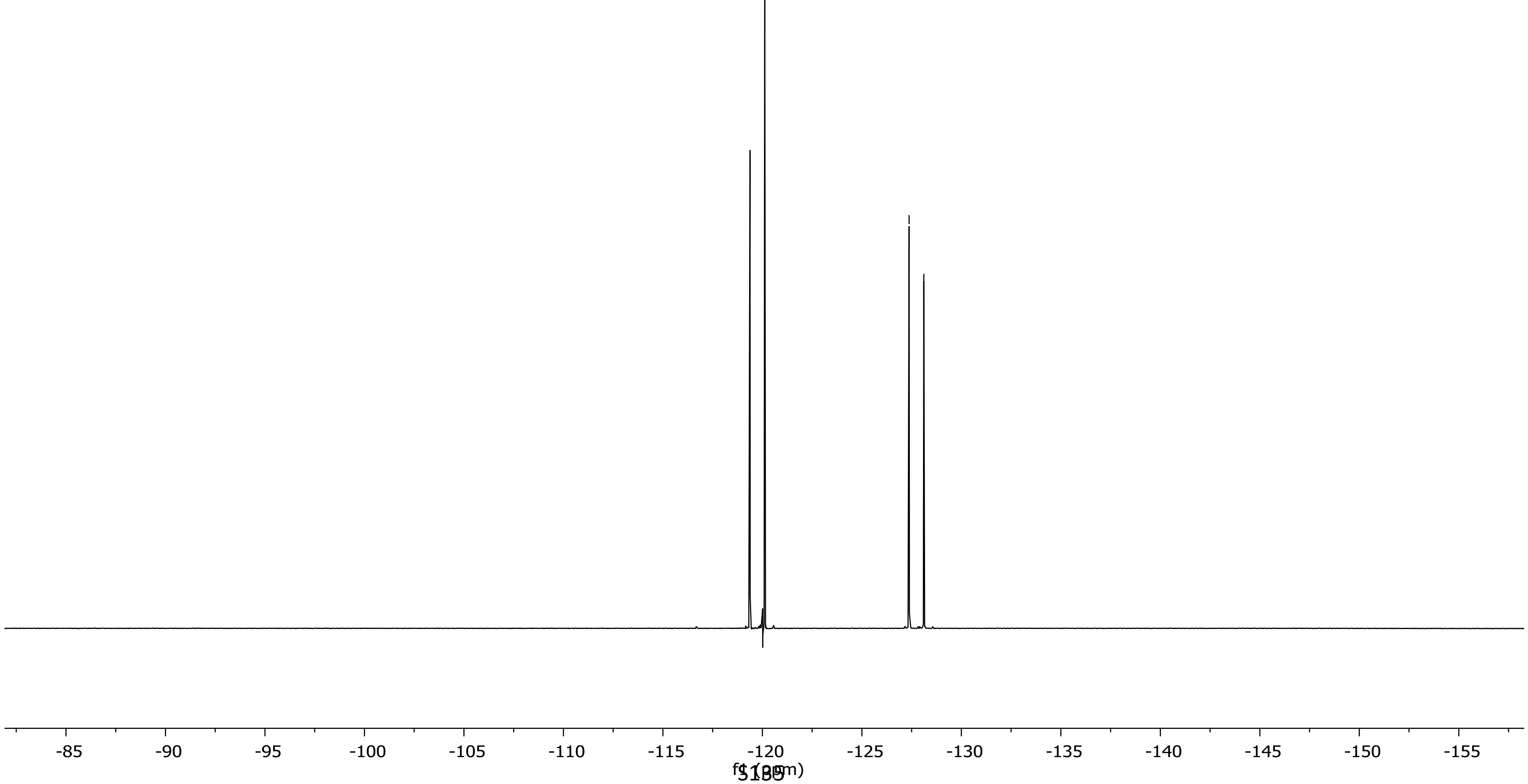

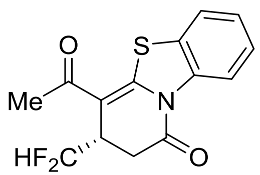

32

^{1}H, 500 MHz, $CDCl_3$

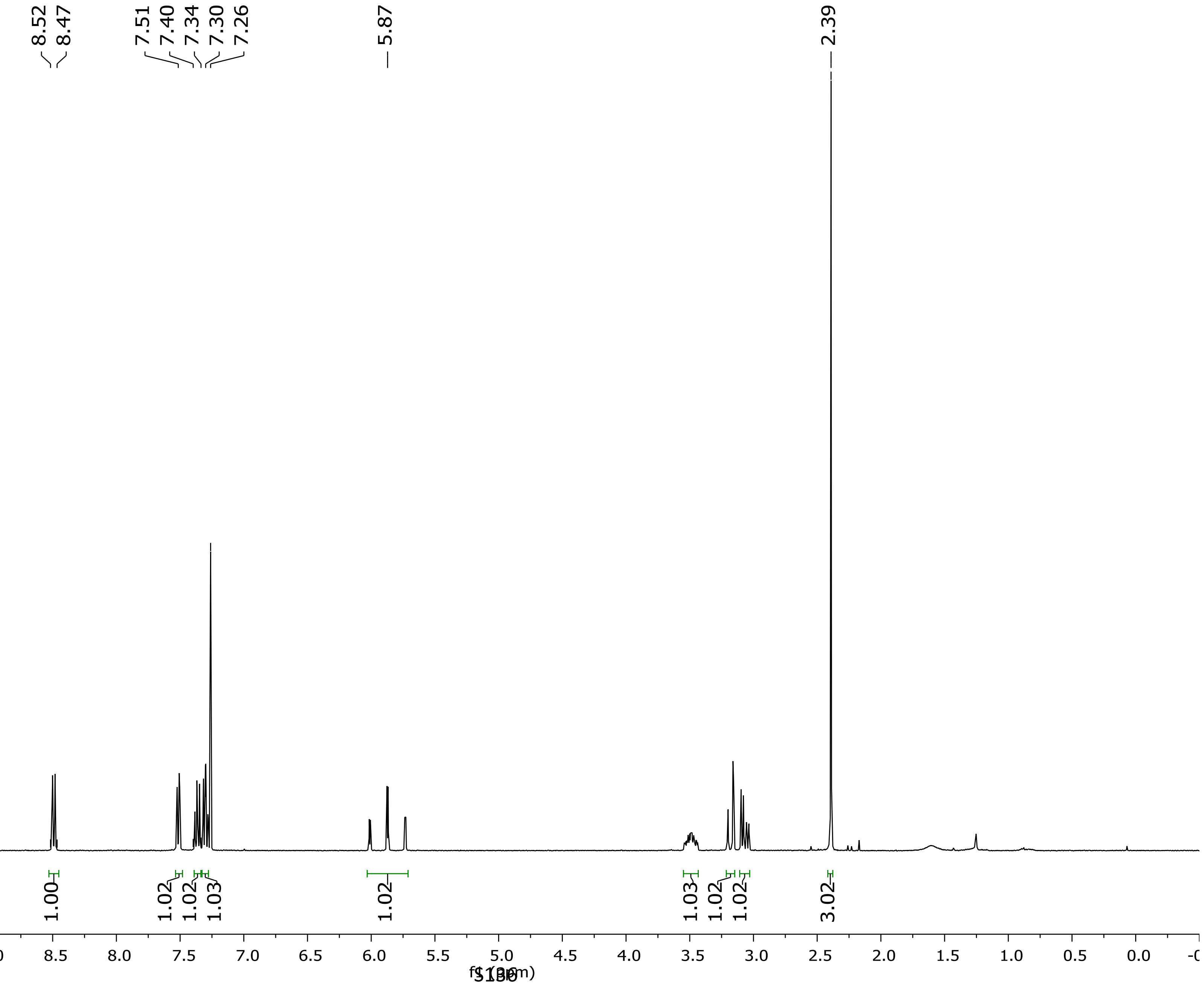

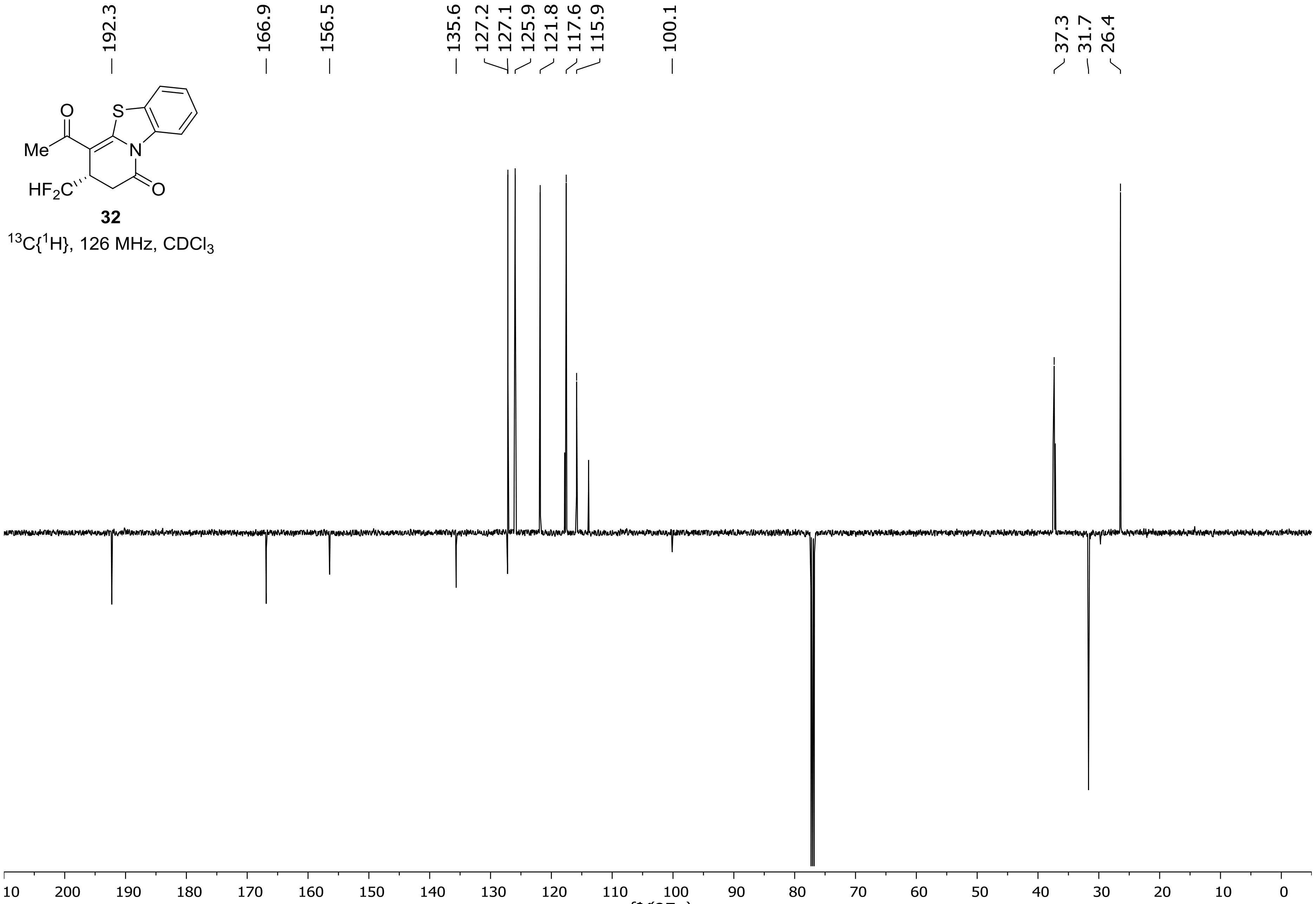

32

$^{13}C\{^{1}H\}$, 126 MHz, $CDCl_3$

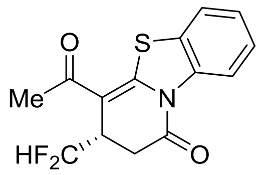

32

$^{19}F\{^{1}H\}$, 376 MHz, $CDCl_3$

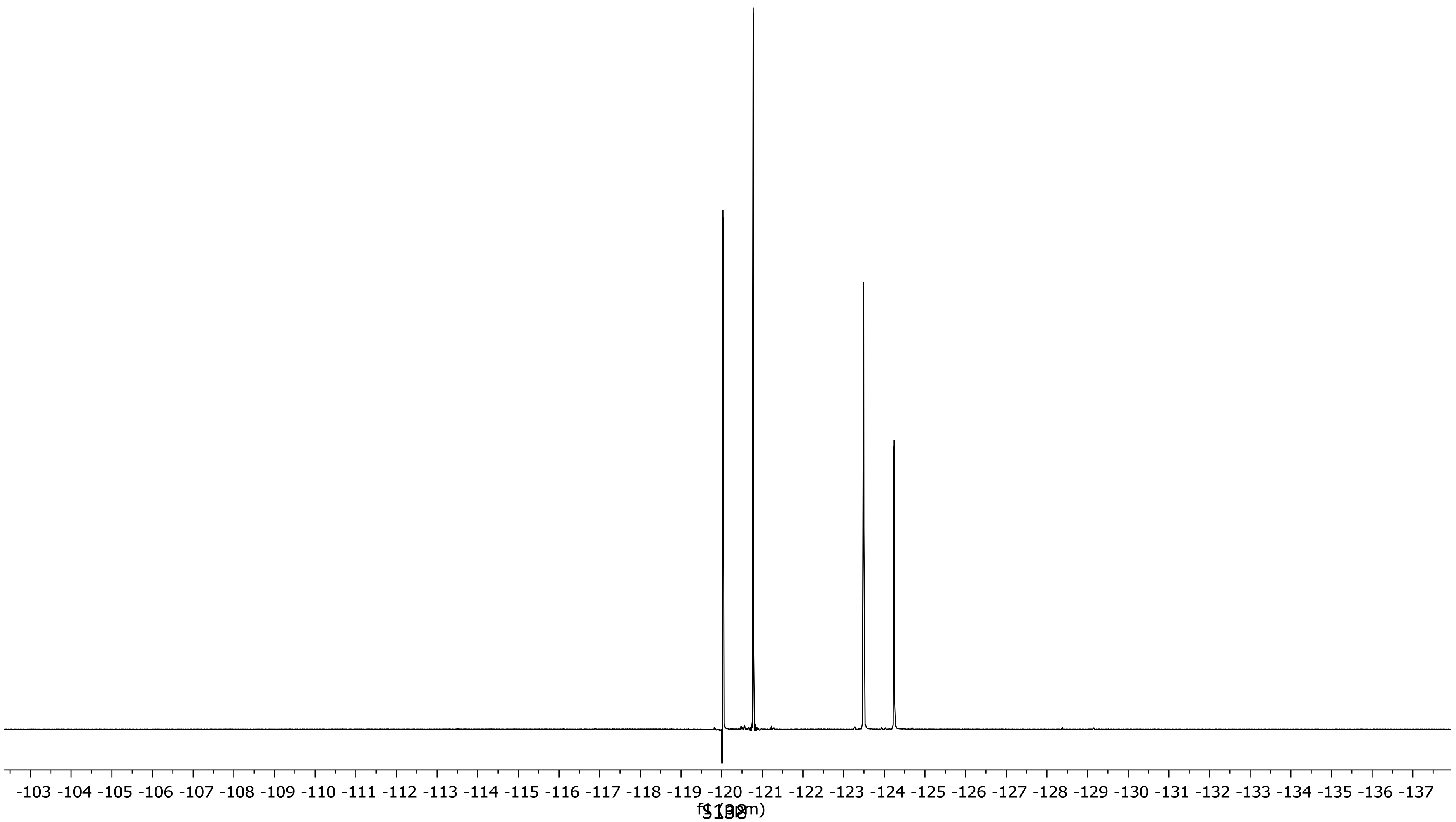

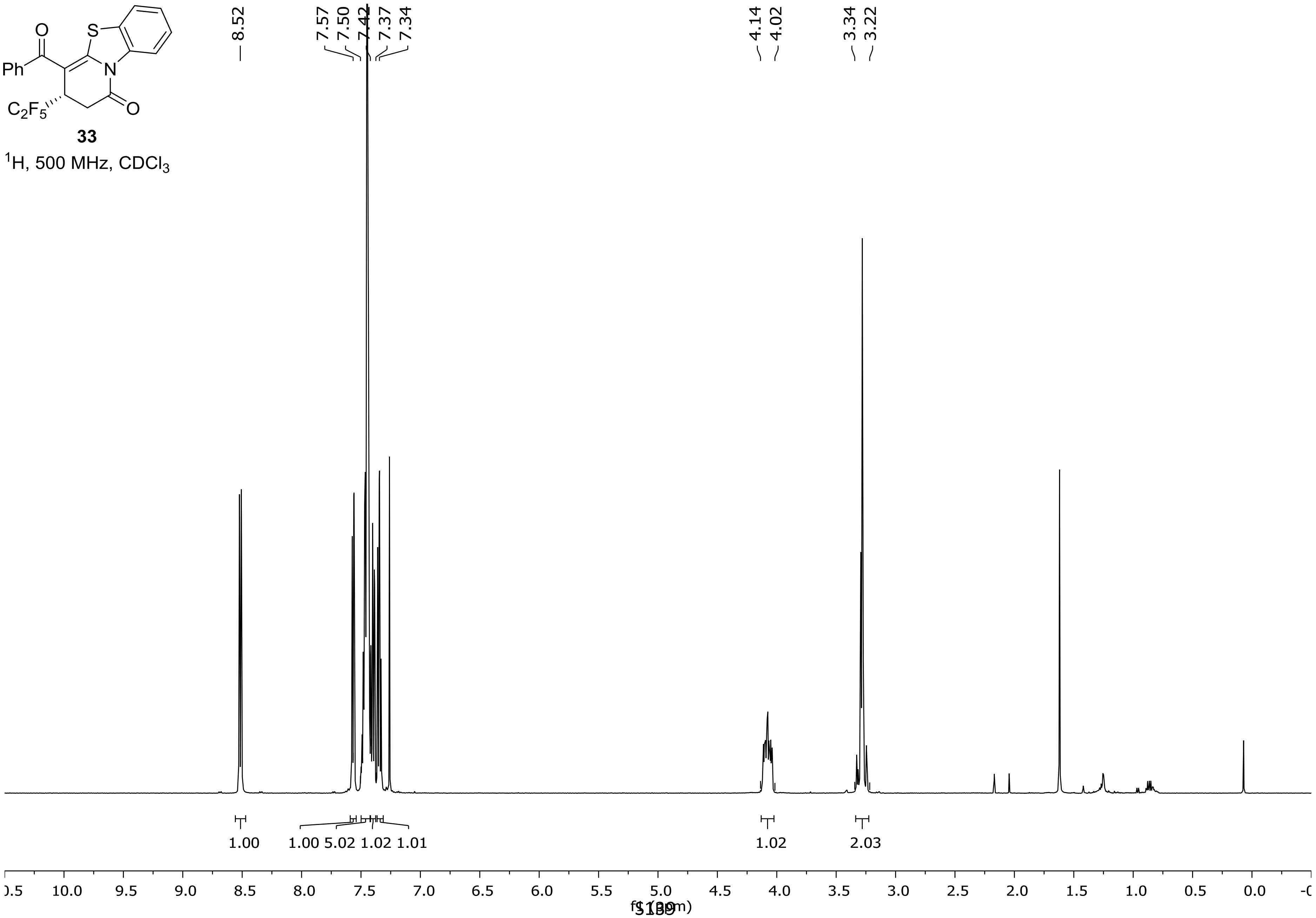
Ph
O
S
N
O
C_2F_5
33
^1H, 500 MHz, $CDCl_3$
8.52
7.57 7.50 7.42 7.37 7.34
4.14 4.02
3.34 3.22
1.00
1.00 5.02 1.02 1.01
1.02
2.03
10.0 9.5 9.0 8.5 8.0 7.5 7.0 6.5 6.0 5.5 5.0 4.5 4.0 3.5 3.0 2.5 2.0 1.5 1.0 0.5 0.0
f1 (ppm)

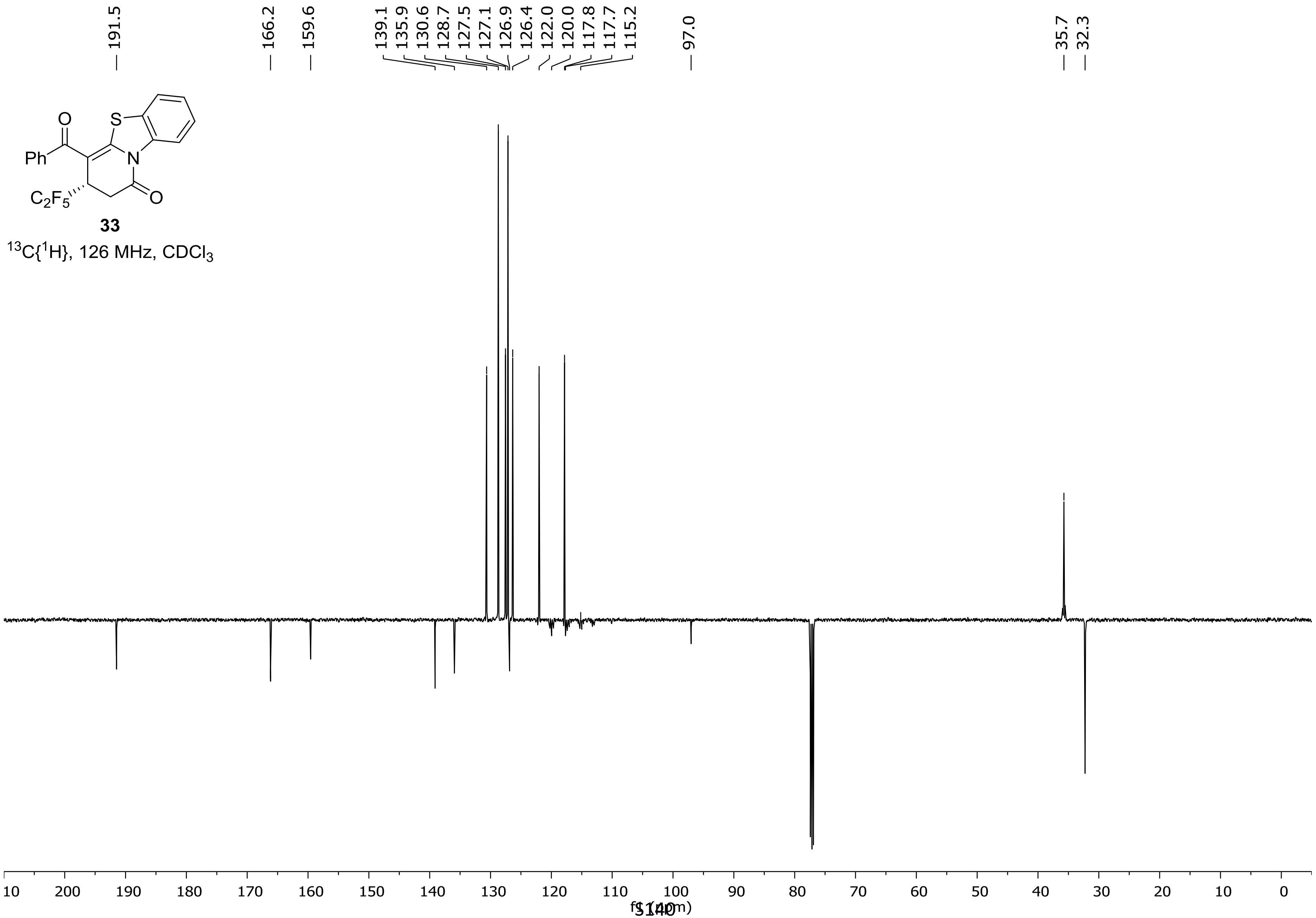
191.5
166.2
159.6
139.1
135.9
130.6
128.7
127.5
127.1
126.9
126.4
122.0
120.0
117.8
117.7
115.2
97.0
35.7
32.3
Ph
O
S
N
O
C_2F_5
33
$^{13}C\{^1H\}$, 126 MHz, $CDCl_3$
10 200 190 180 170 160 150 140 130 120 110 100 90 80 70 60 50 40 30 20 10 0
f1 (ppm)

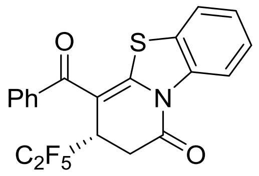

33

$^{19}F\{^{1}H\}$, 376 MHz, $CDCl_3$

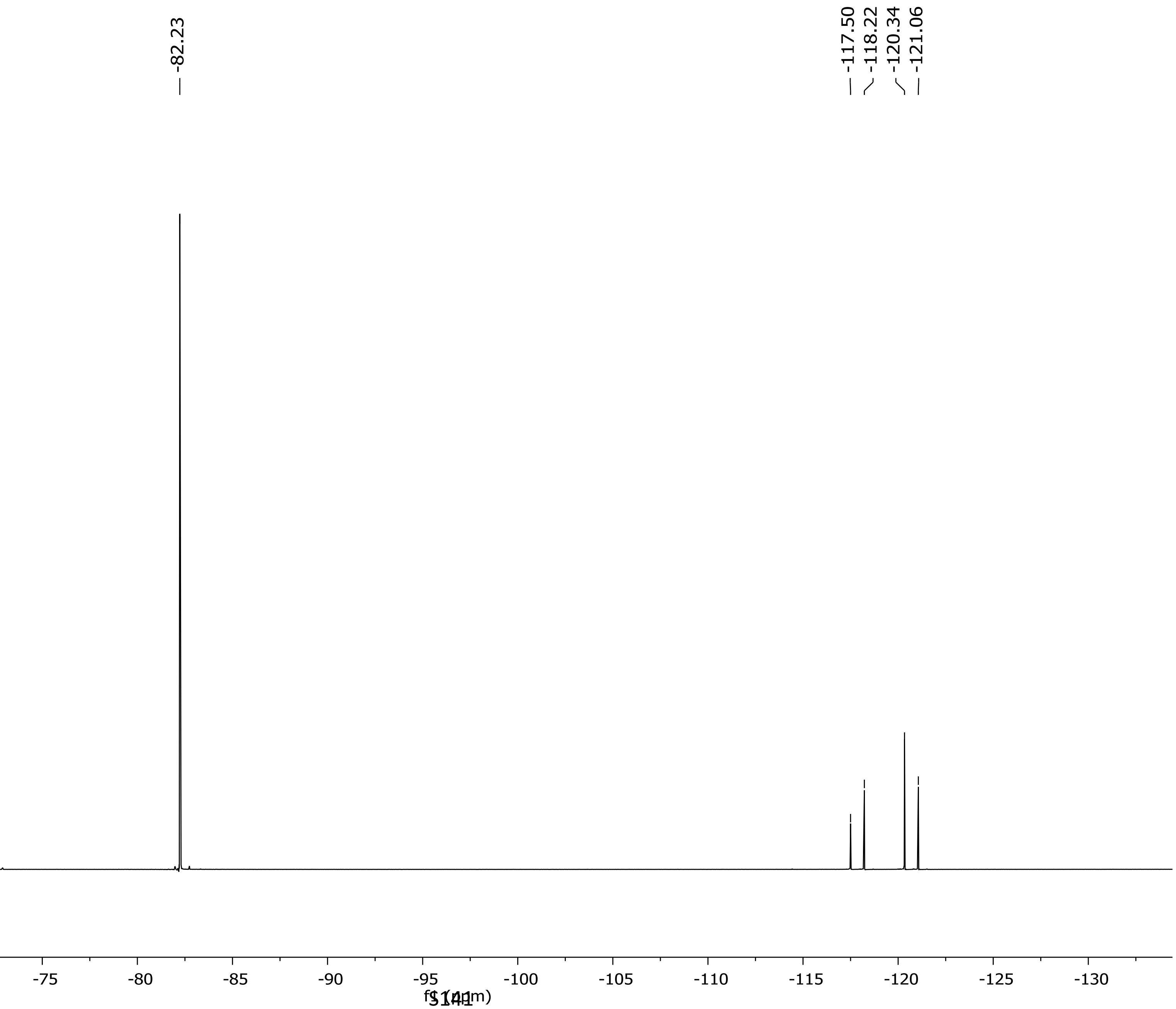

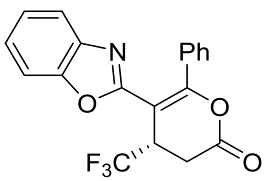

34

^1H, 500 MHz, $CDCl_3$

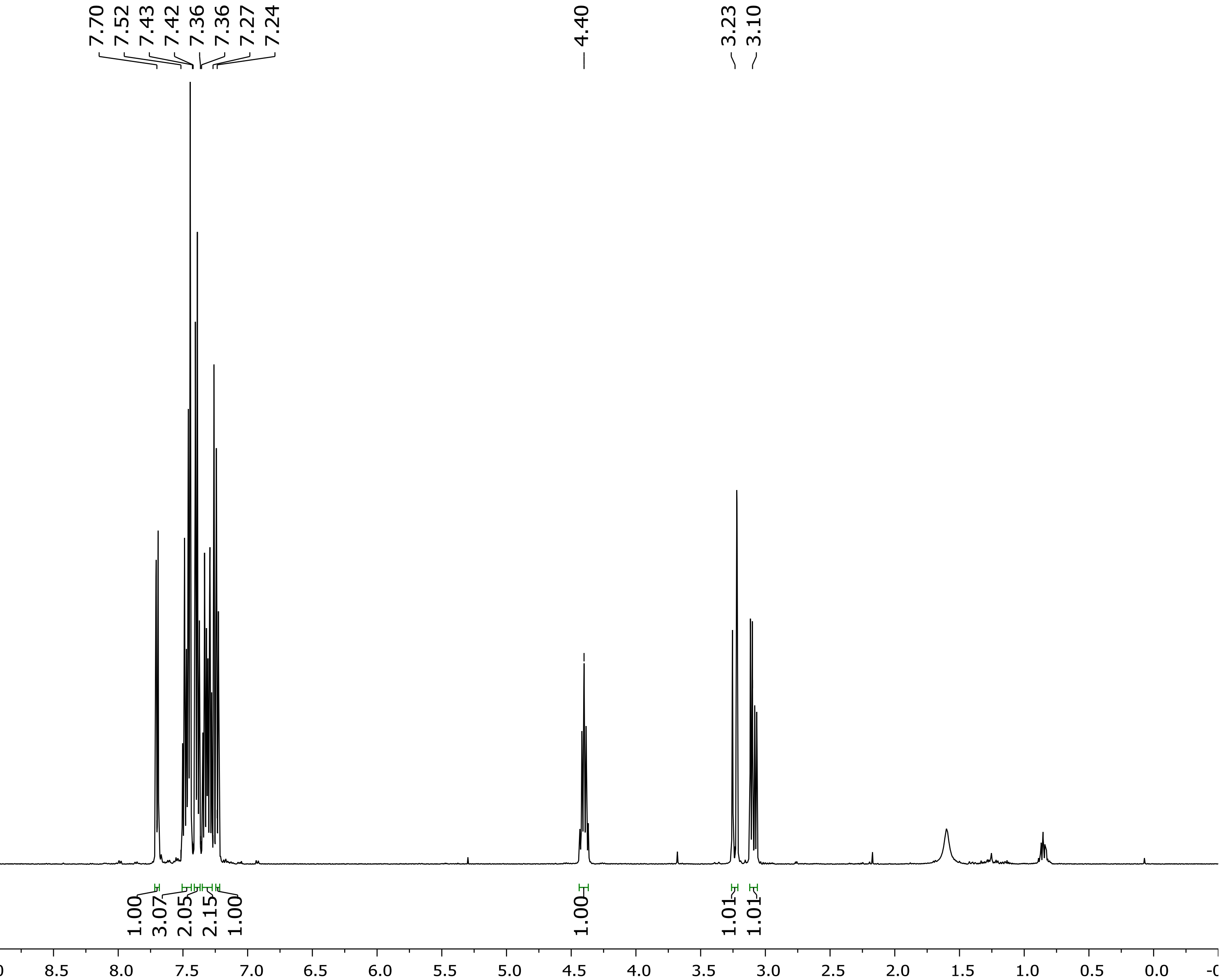

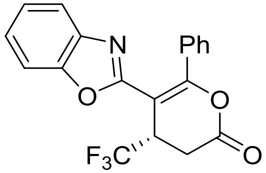

34

$^{13}C\{^{1}H\}$, 126 MHz, $CDCl_3$

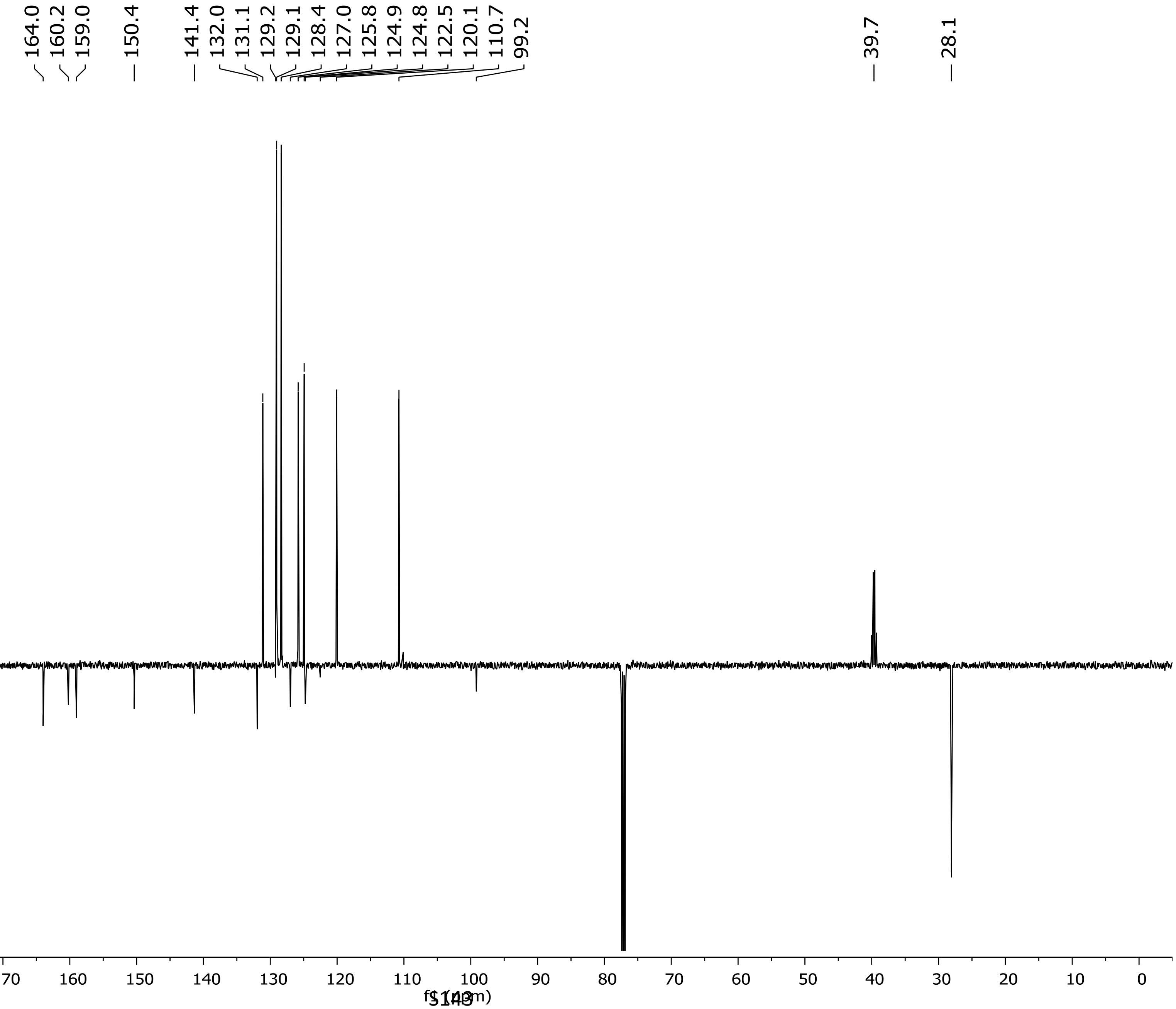

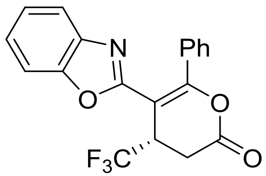

34

$^{19}F\{^{1}H\}$, 376 MHz, $CDCl_3$

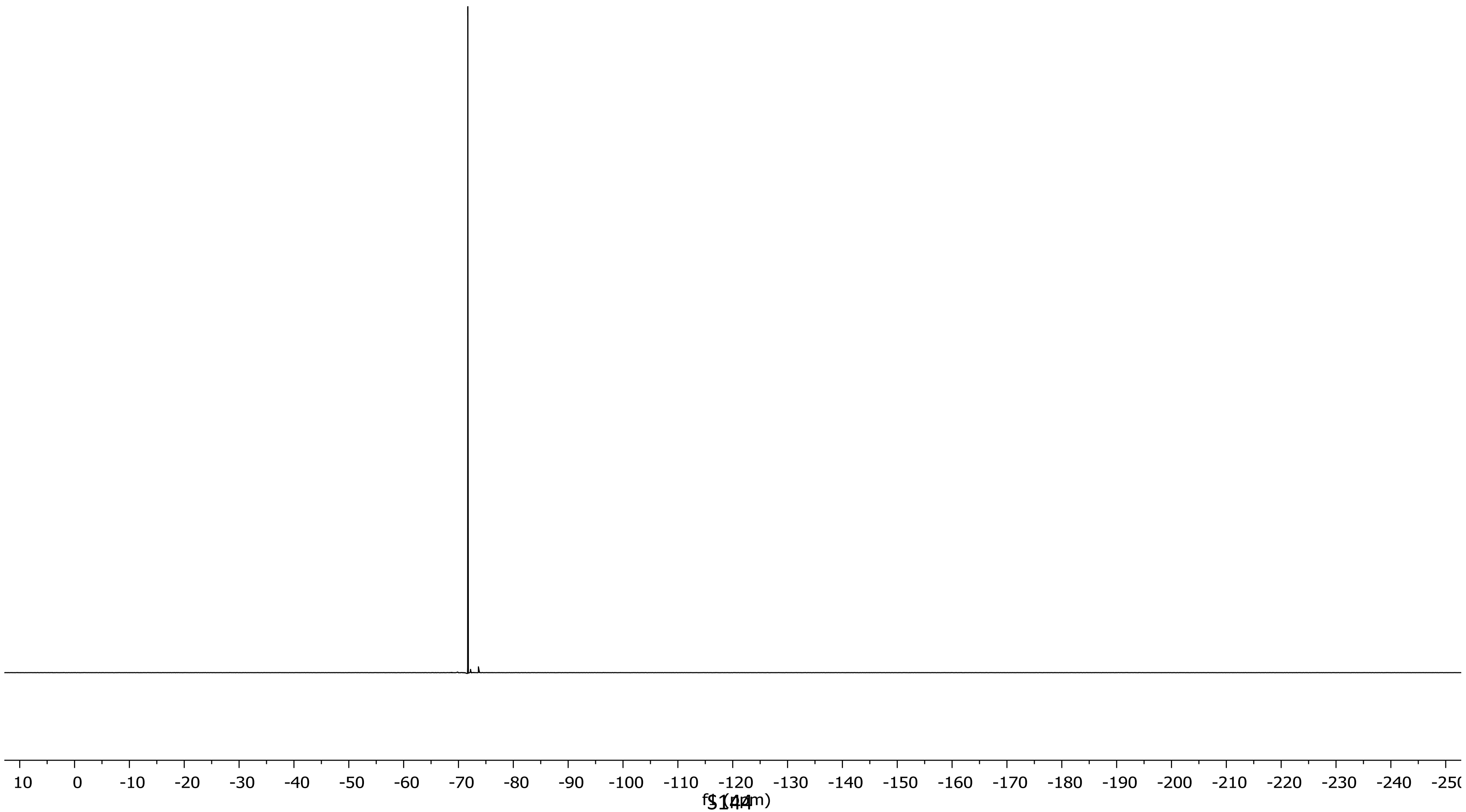

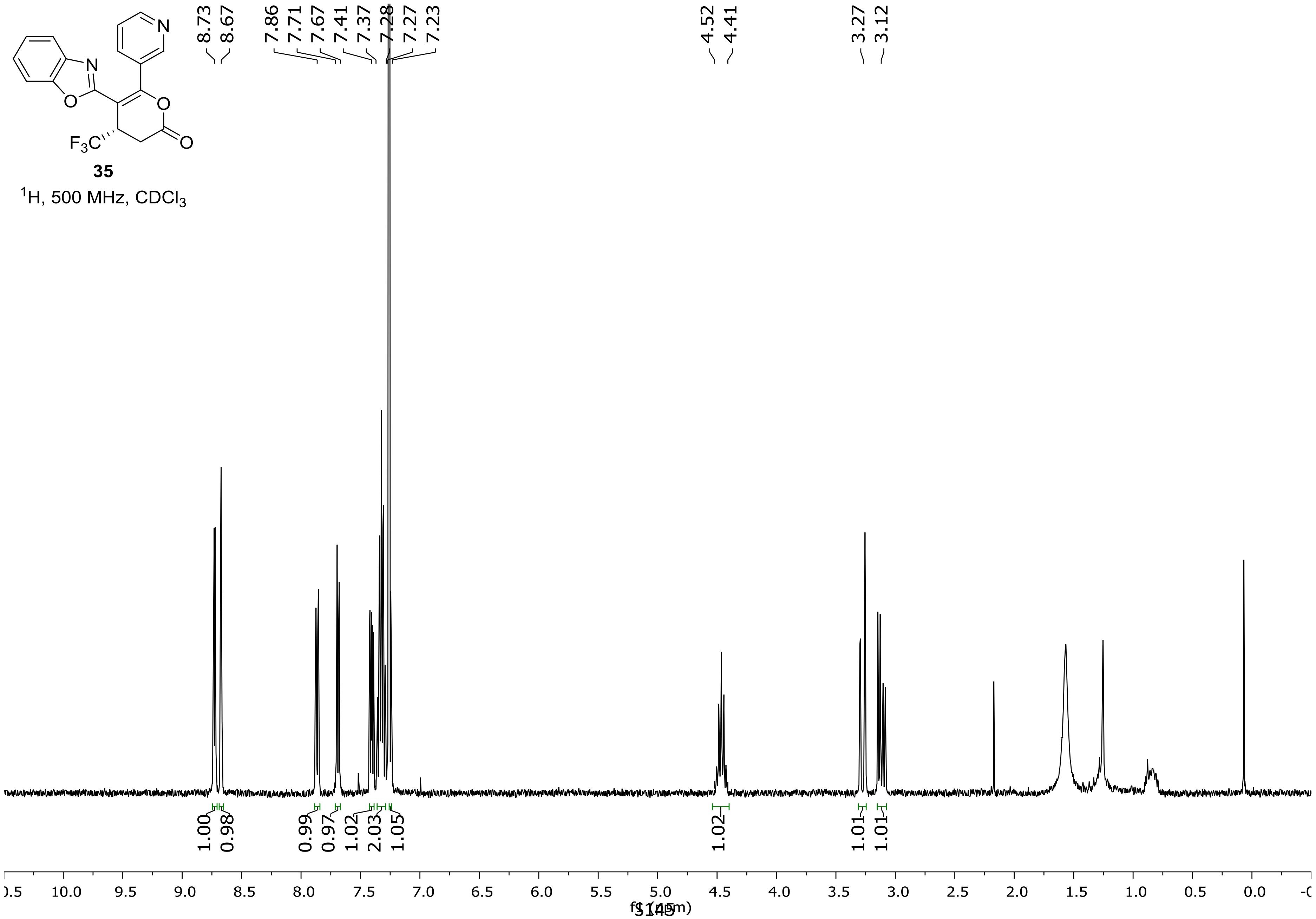

35

^{1}H, 500 MHz, $CDCl_3$

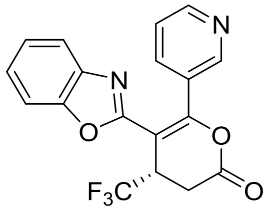

35

$^{13}C\{^1H\}$, 126 MHz, $CDCl_3$

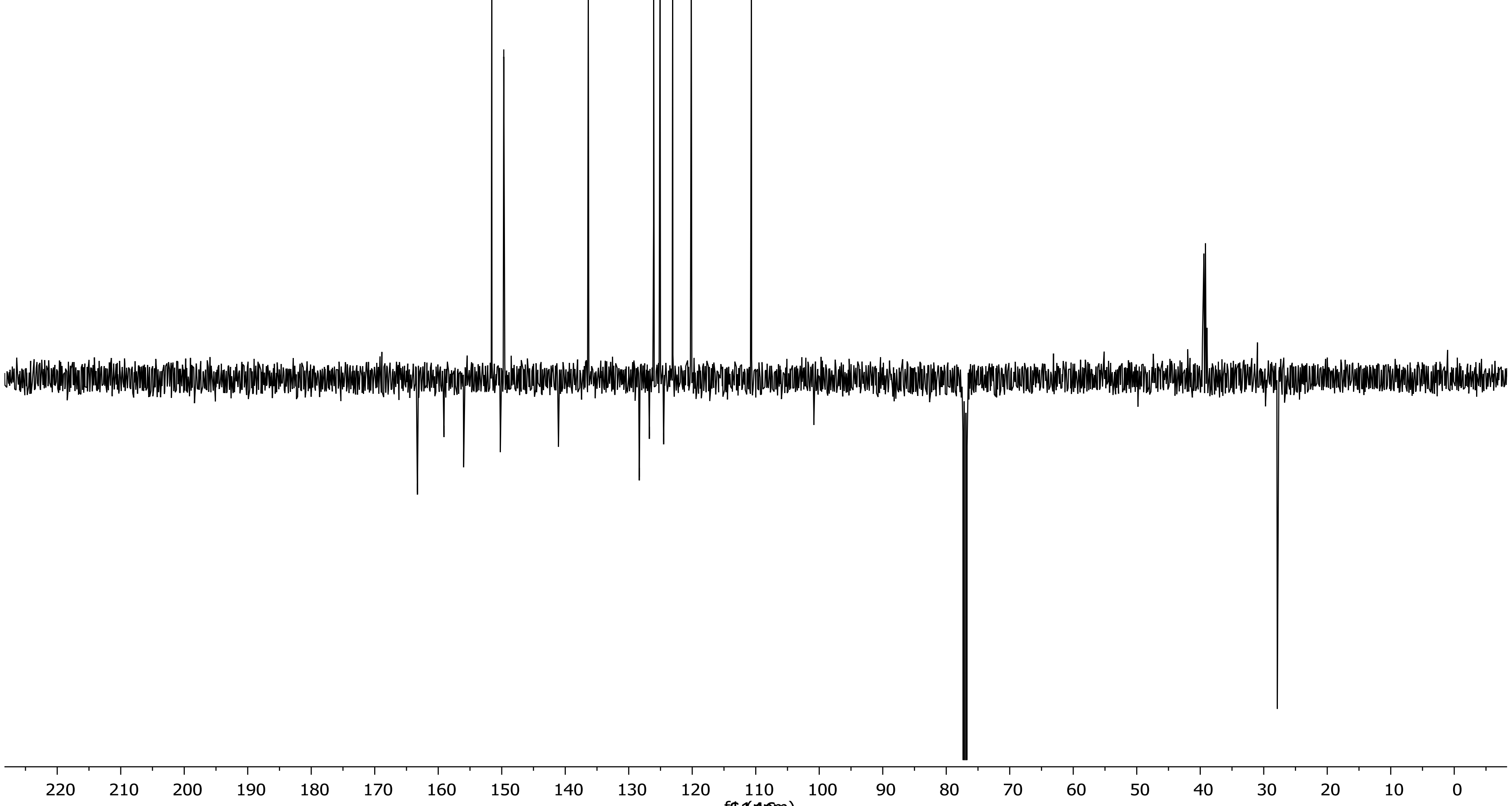

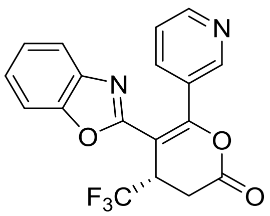

35

$^{19}F\{^1H\}$, 376 MHz, $CDCl_3$

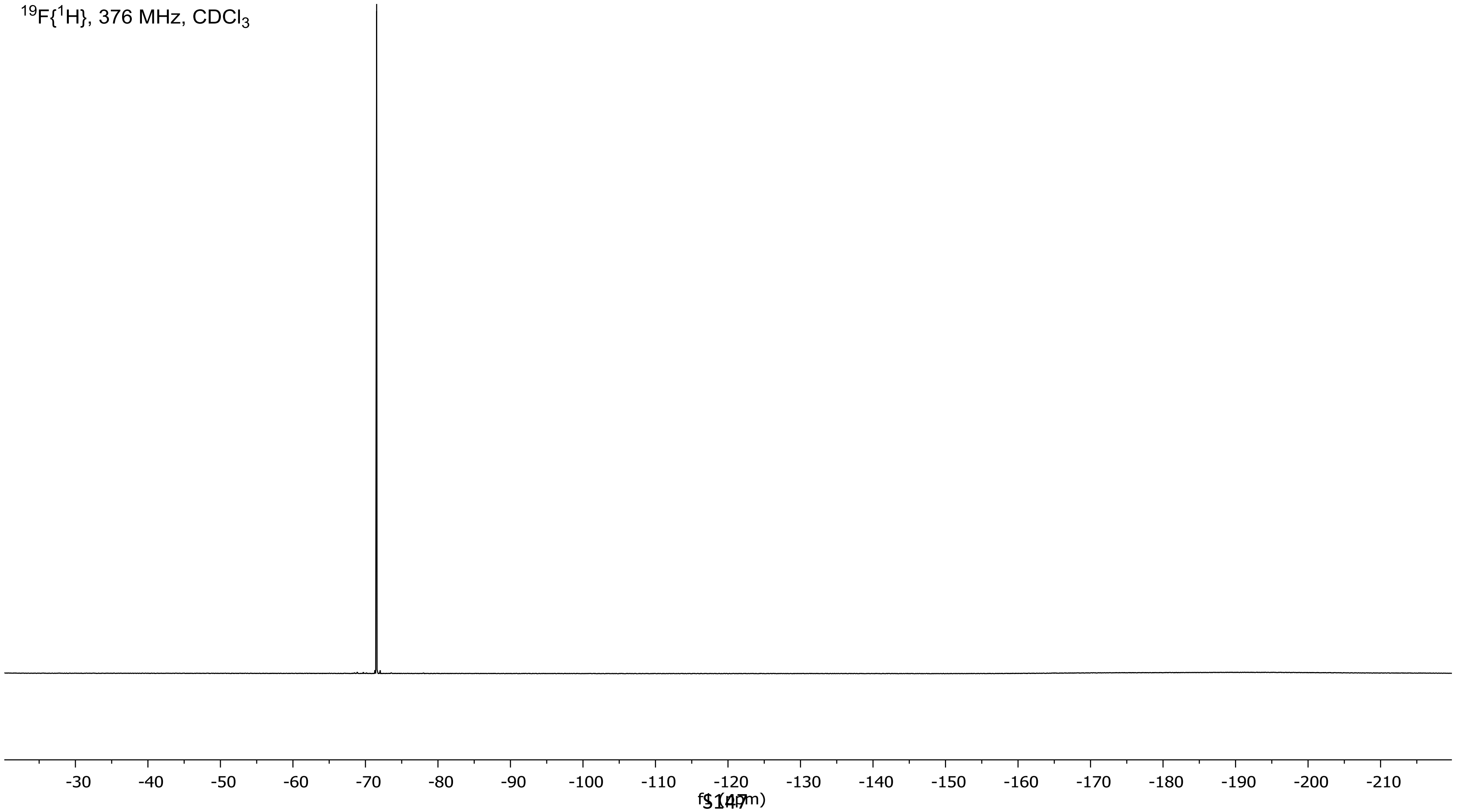

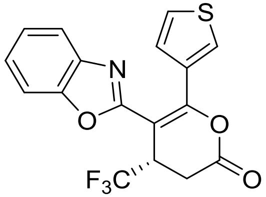

36

^{1}H, 500 MHz, $CDCl_3$

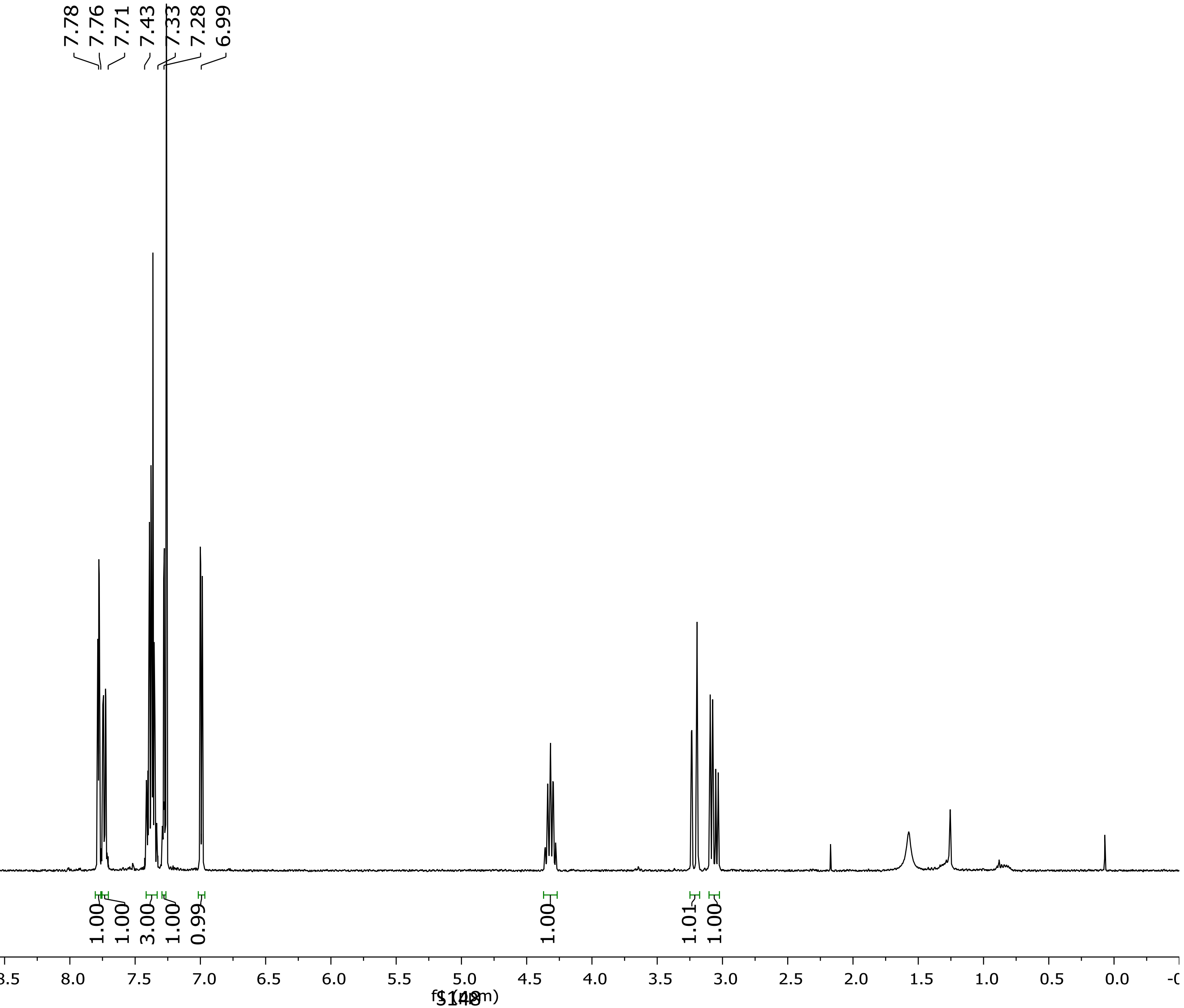

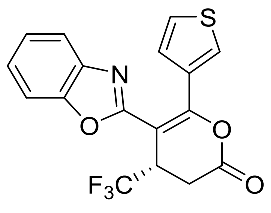

36

$^{13}C\{^{1}H\}$, 126 MHz, $CDCl_3$

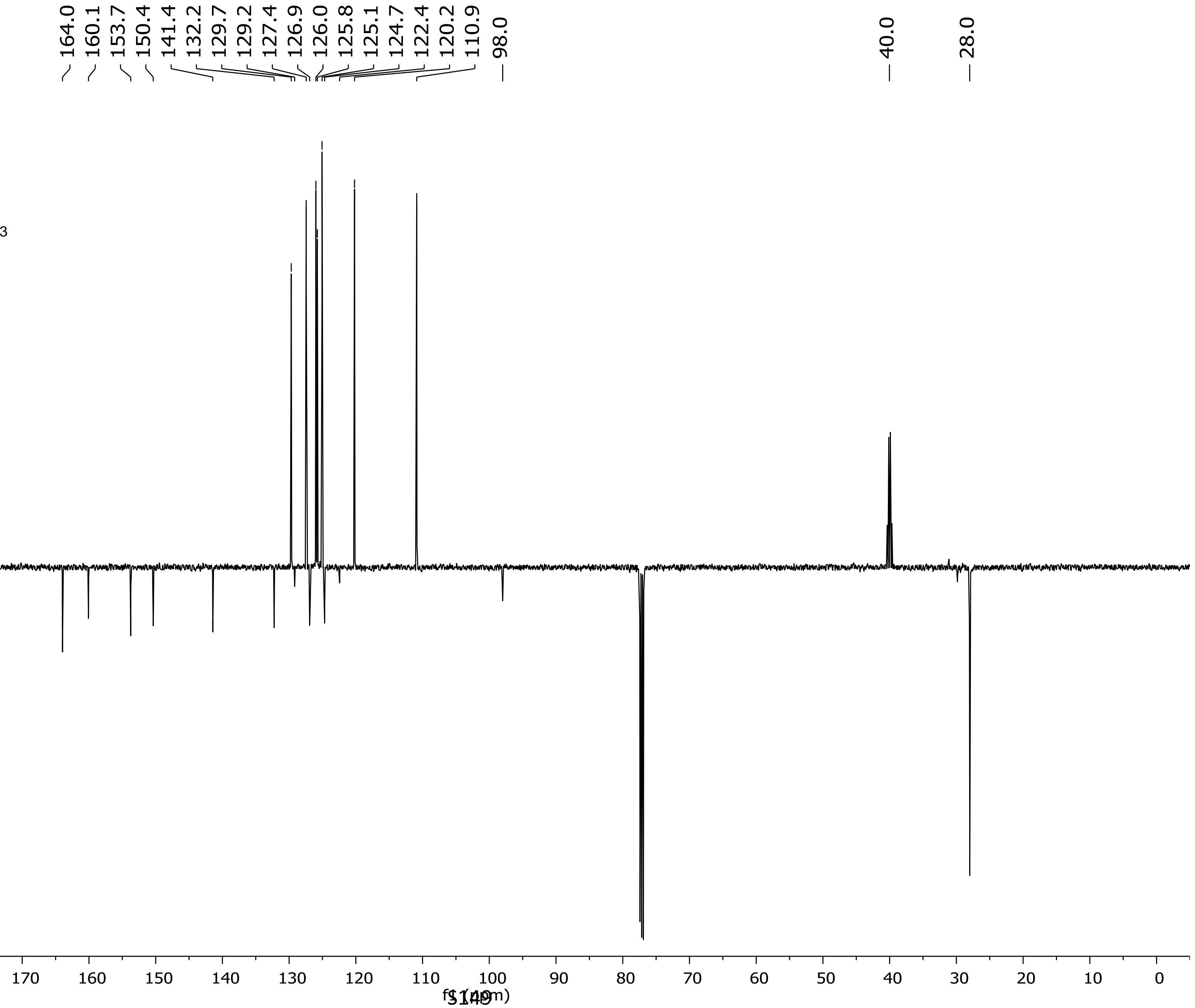

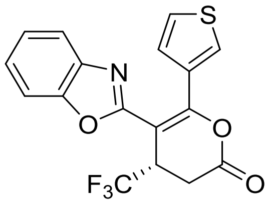

36

$^{19}F\{^{1}H\}$, 376 MHz, $CDCl_3$

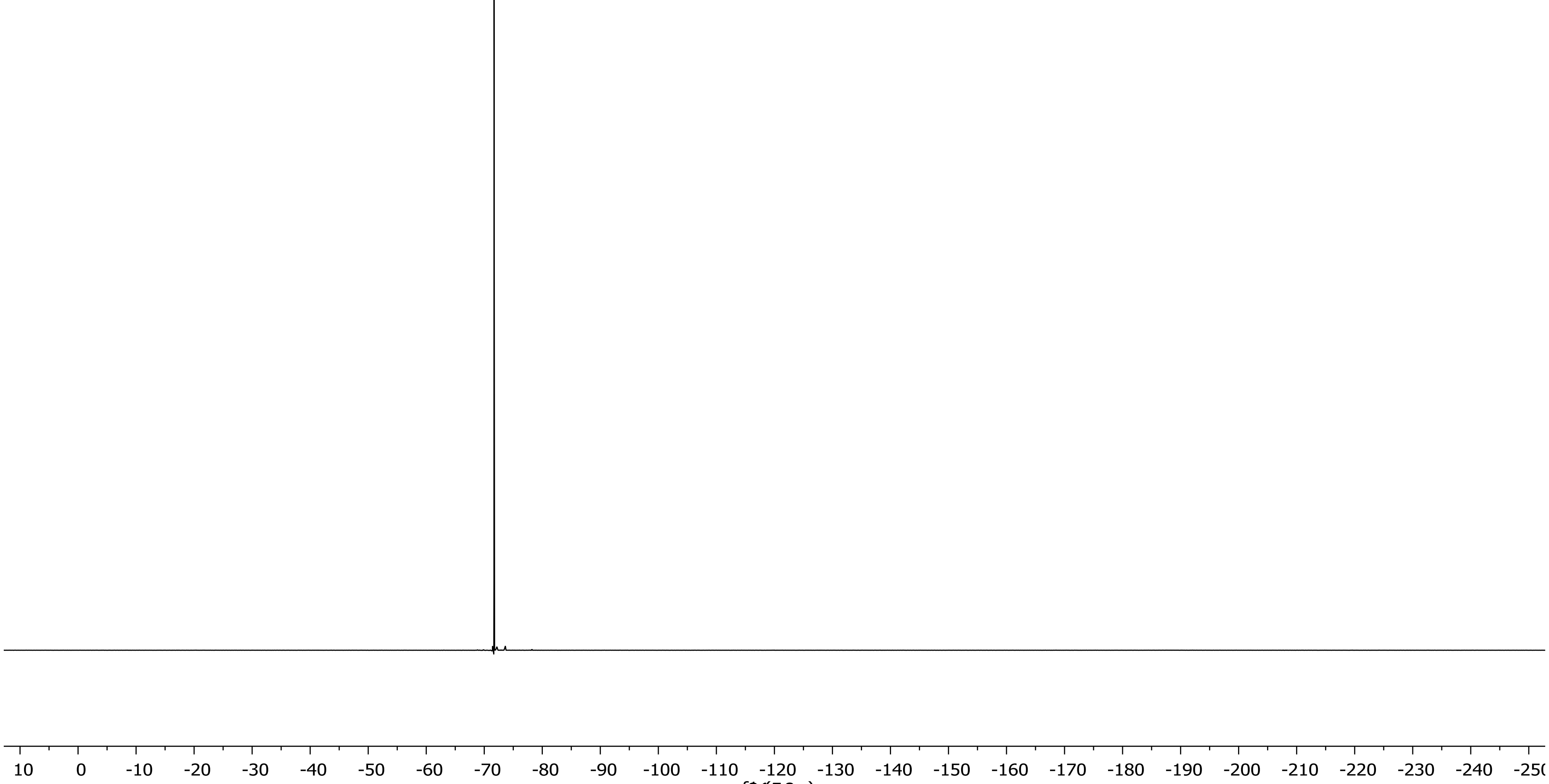

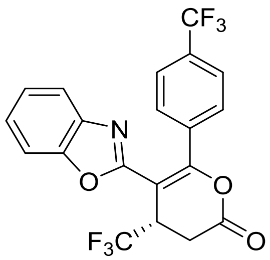

37

^{1}H, 500 MHz, $CDCl_3$

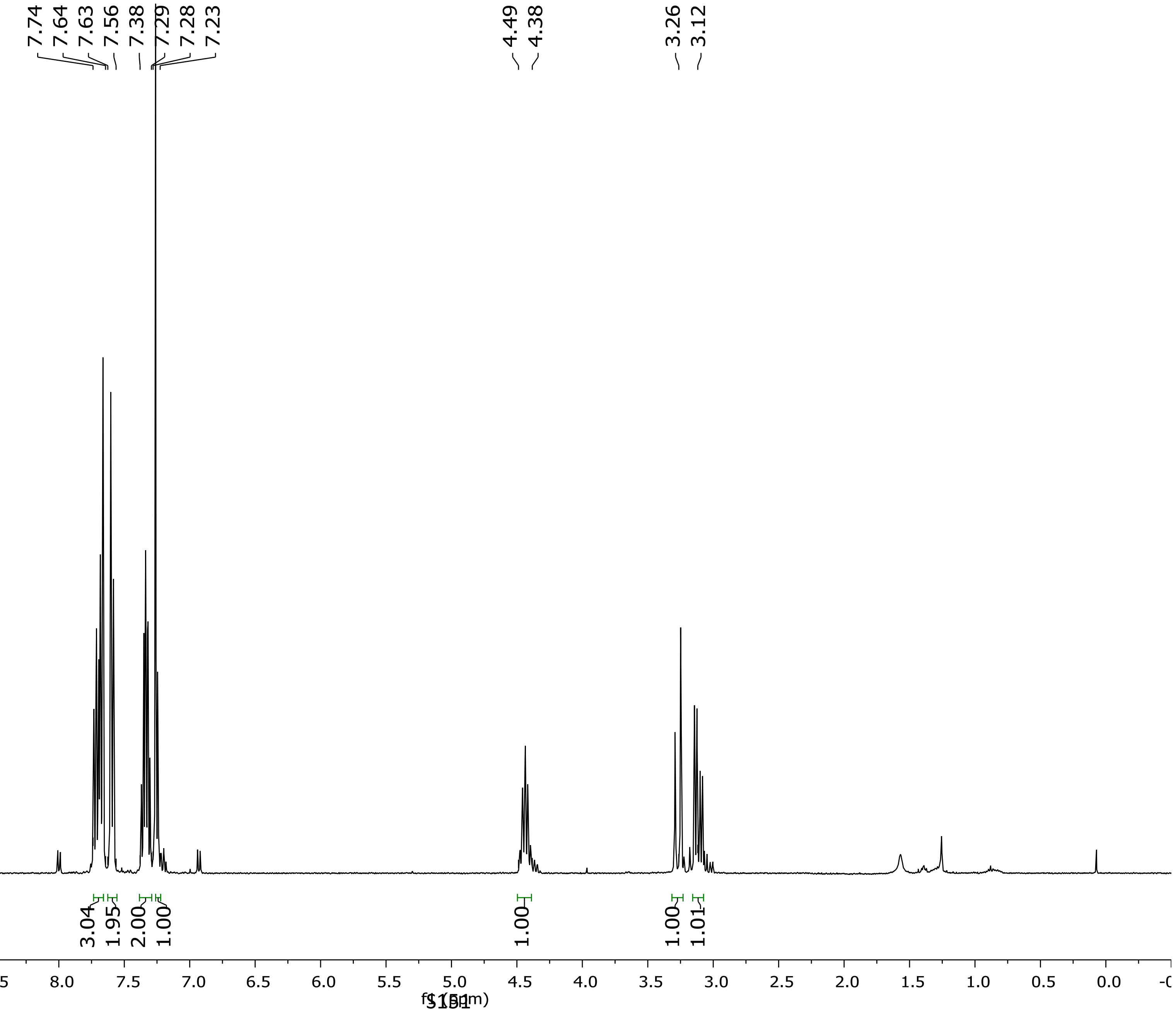

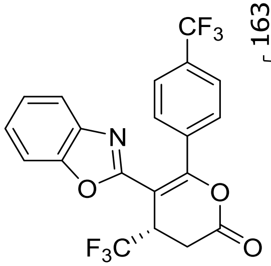

37

$^{13}C\{^{1}H\}$, 126 MHz, $CDCl_3$

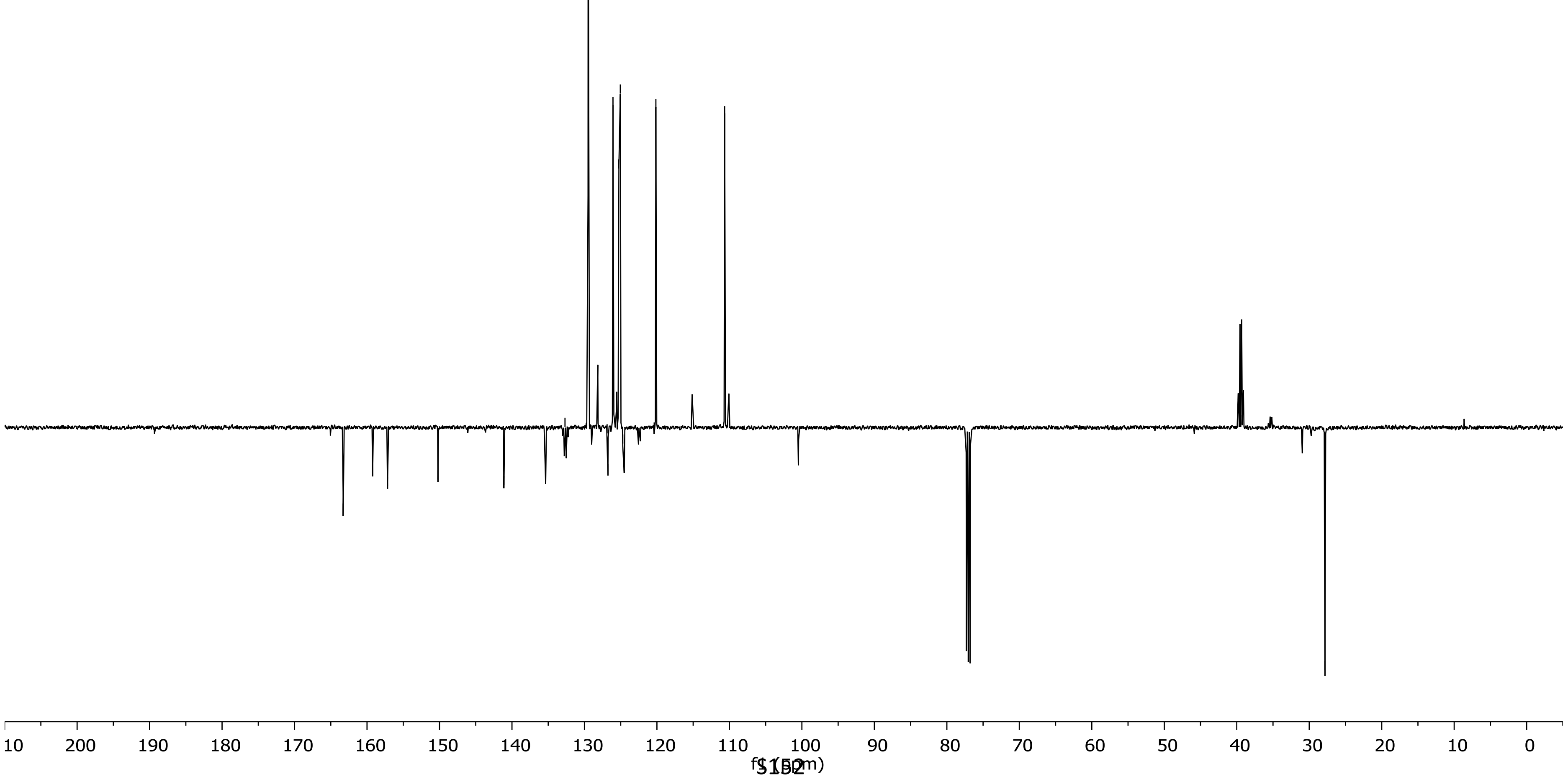

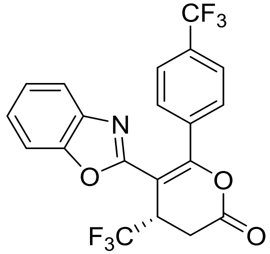

37

$^{19}F\{^{1}H\}$, 376 MHz, $CDCl_3$

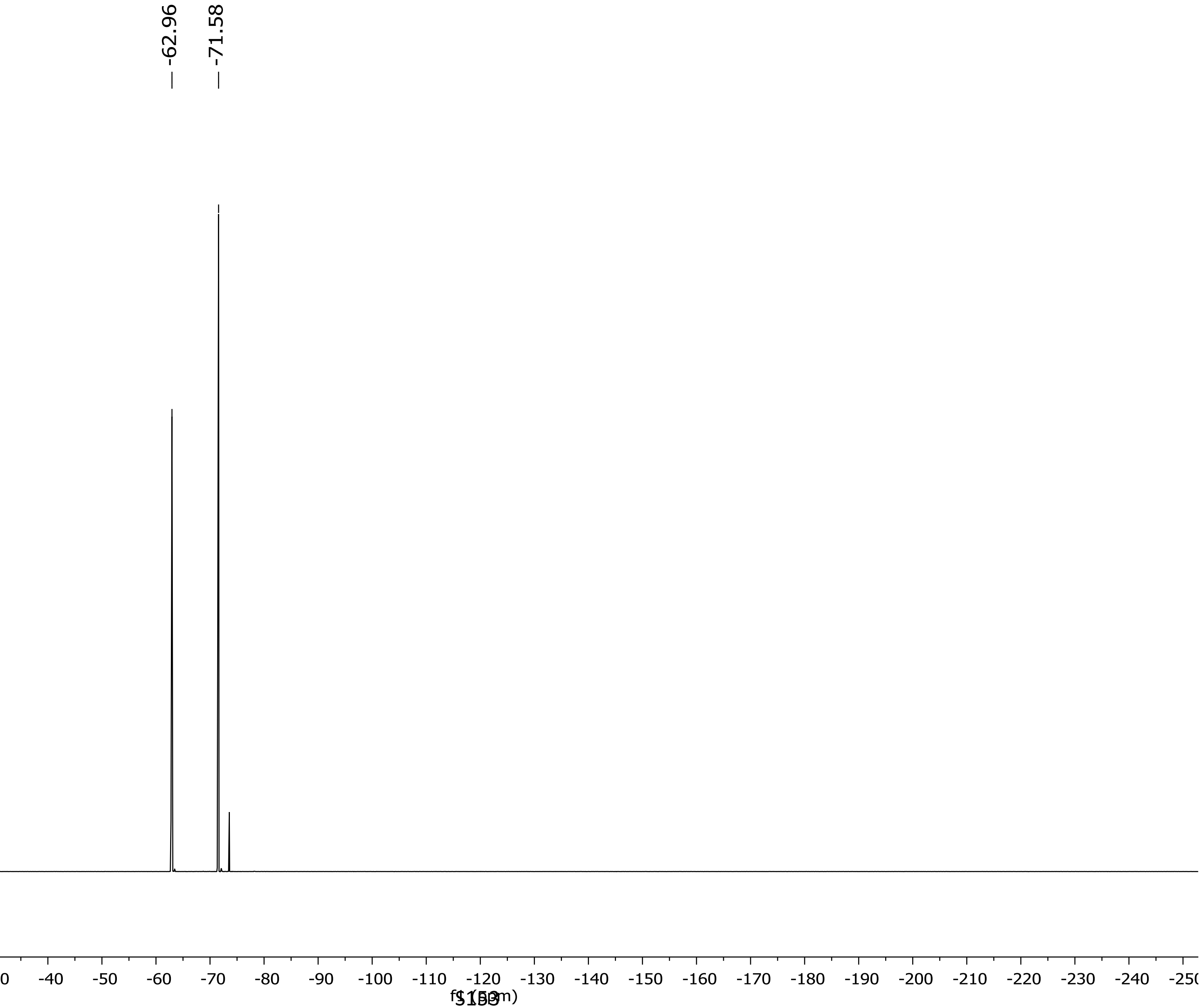

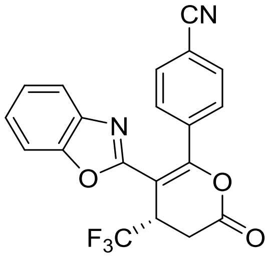

38

^1H, 500 MHz, $CDCl_3$

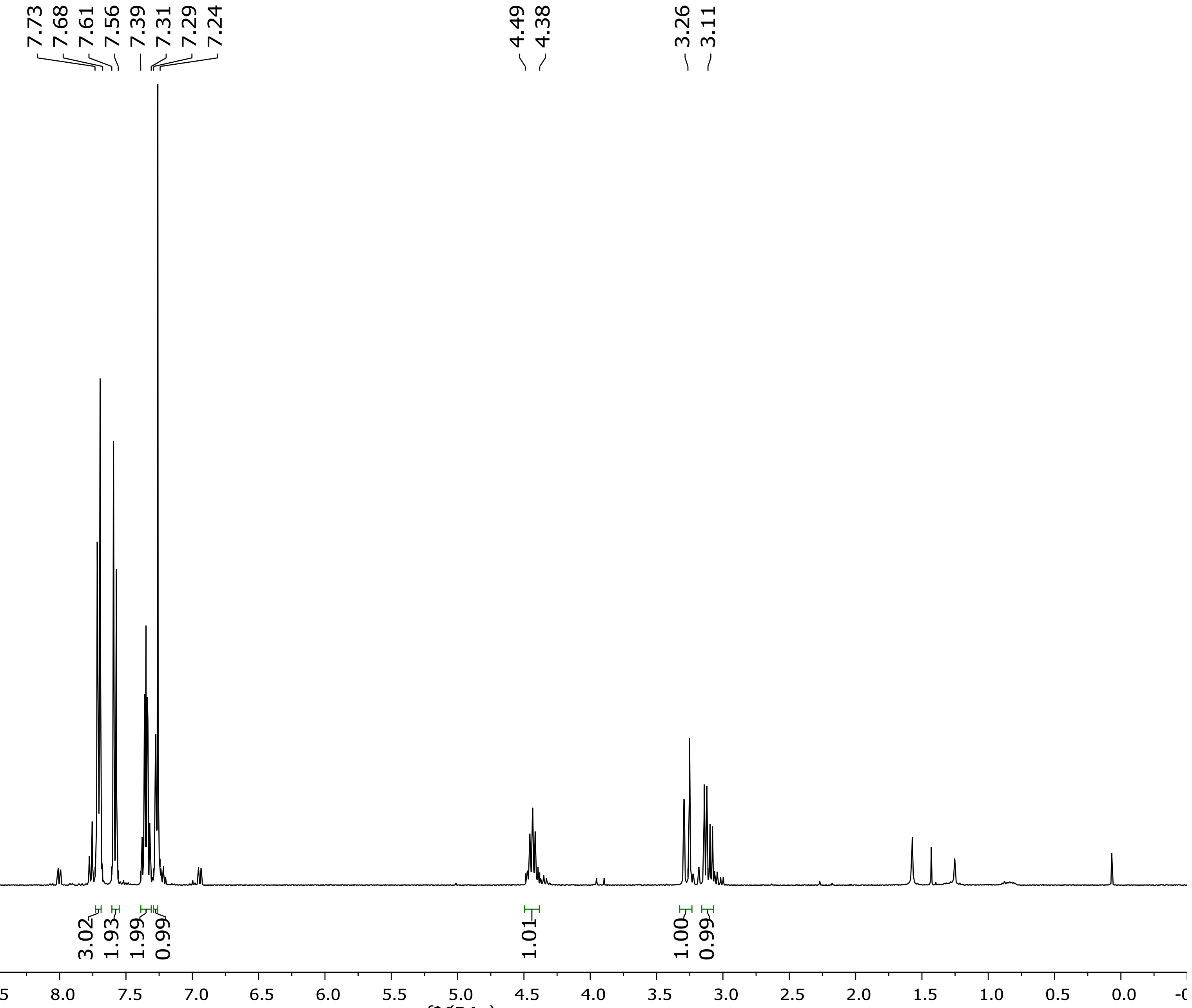

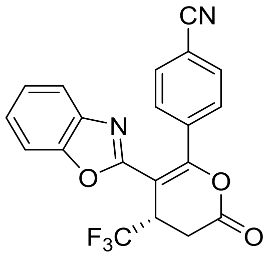

38

$^{13}C\{^{1}H\}$, 126 MHz, $CDCl_3$

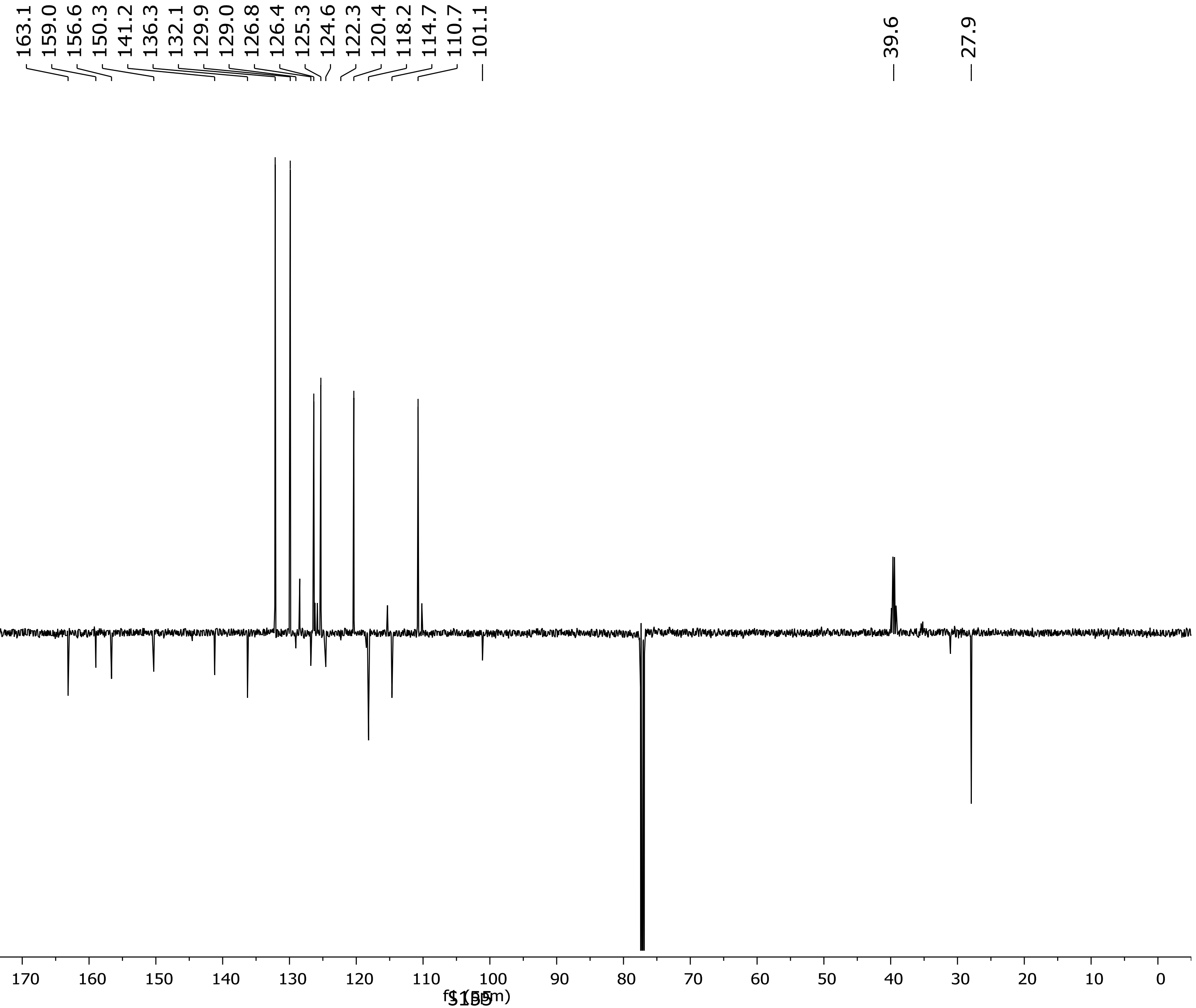

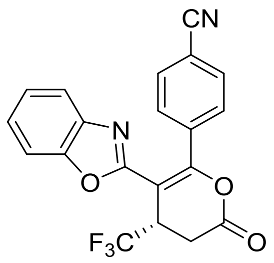

38

$^{19}F\{^{1}H\}$, 376 MHz, $CDCl_3$

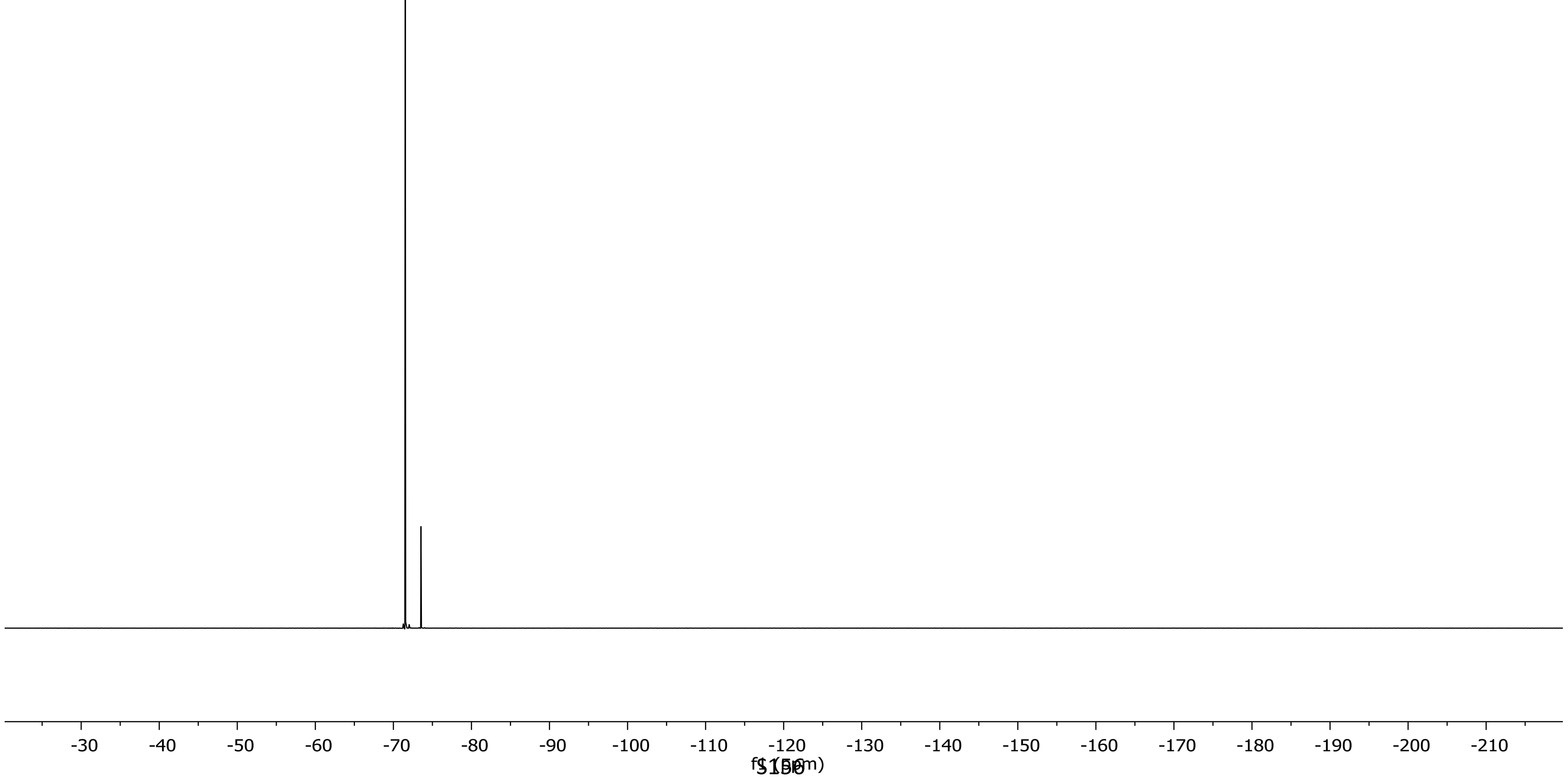

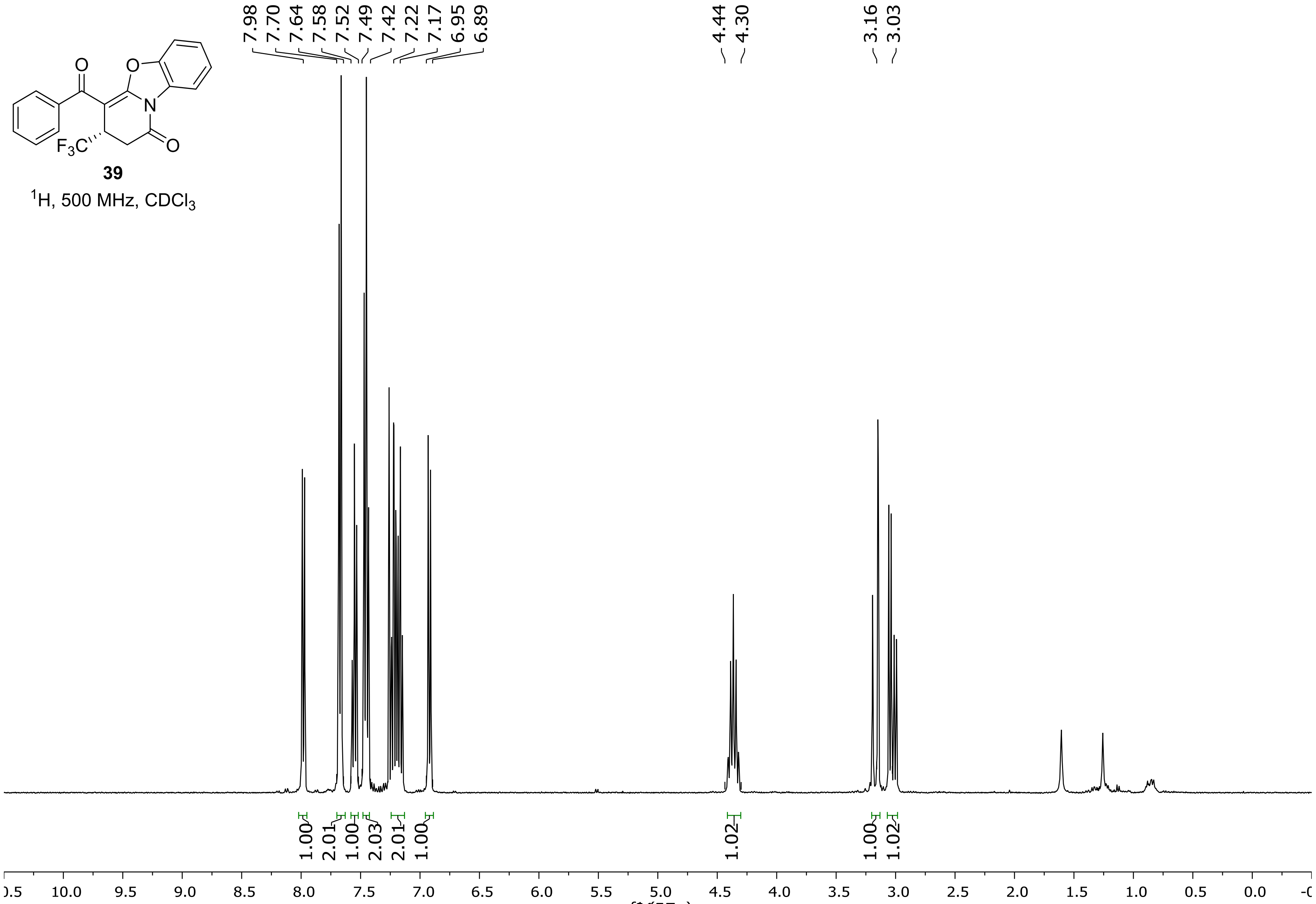

39

^{1}H, 500 MHz, $CDCl_3$

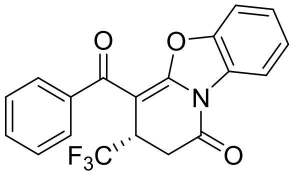

39

$^{13}C\{^{1}H\}$, 126 MHz, $CDCl_3$

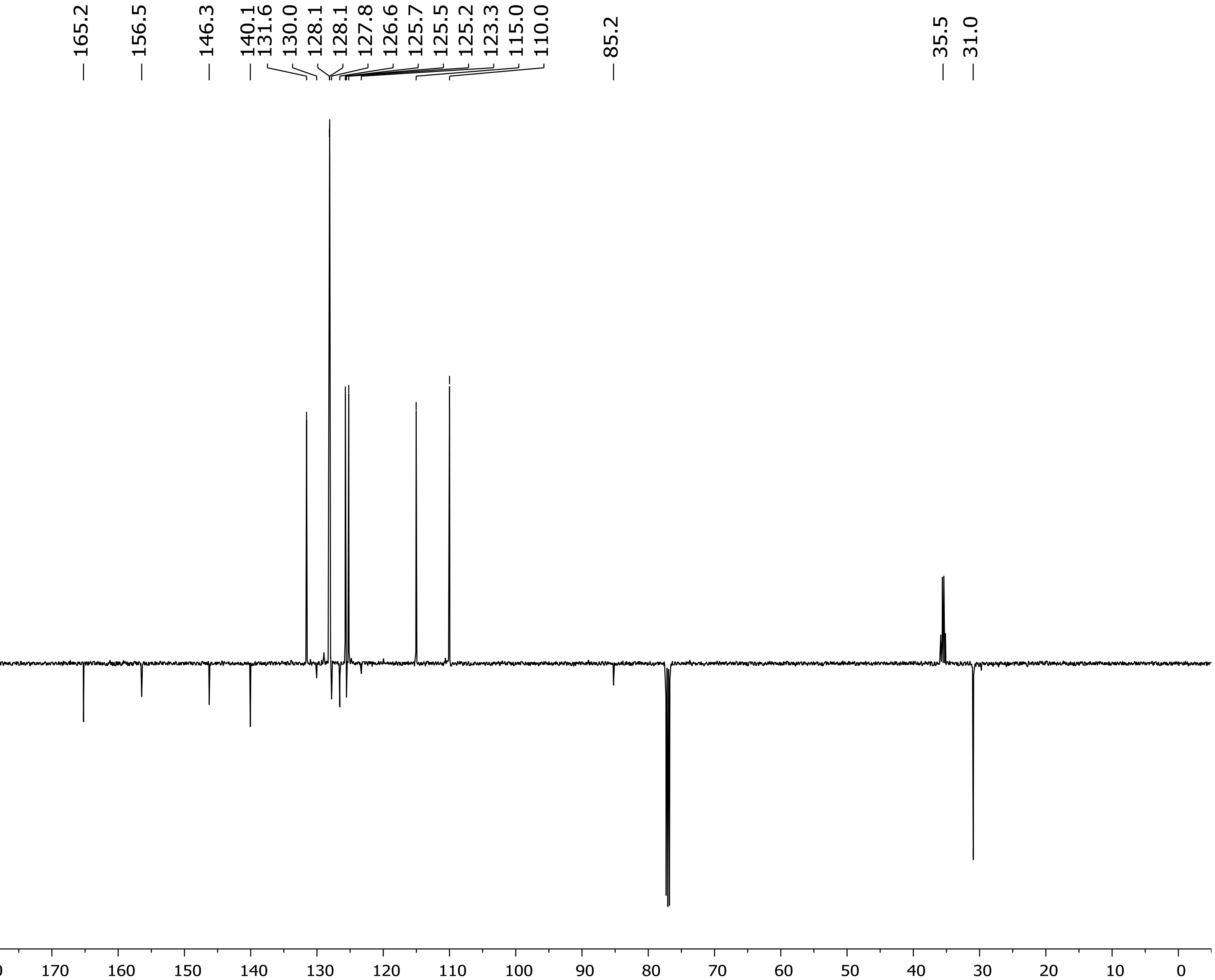

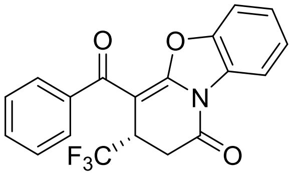

39

$^{19}F\{^1H\}$, 376 MHz, $CDCl_3$

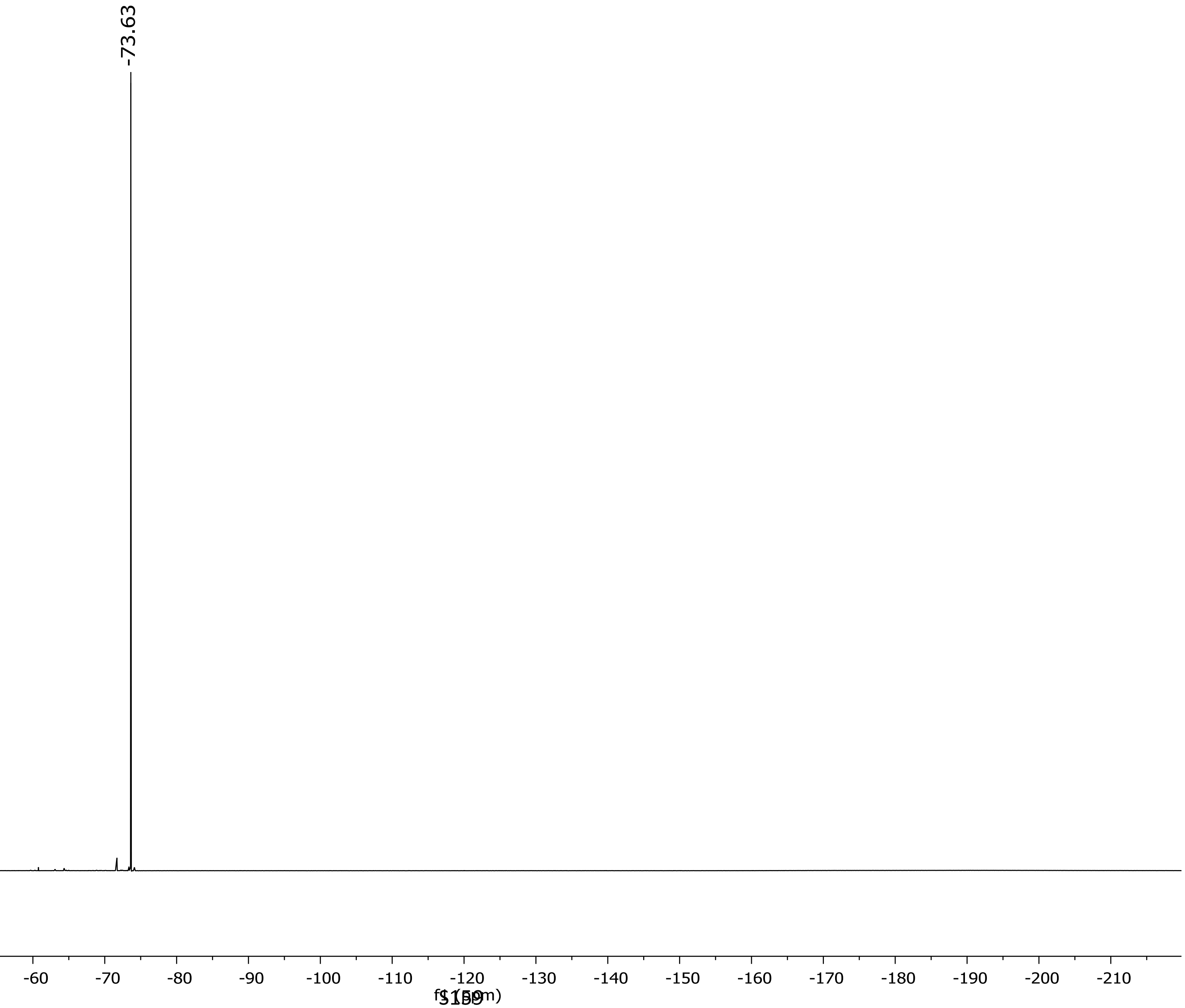

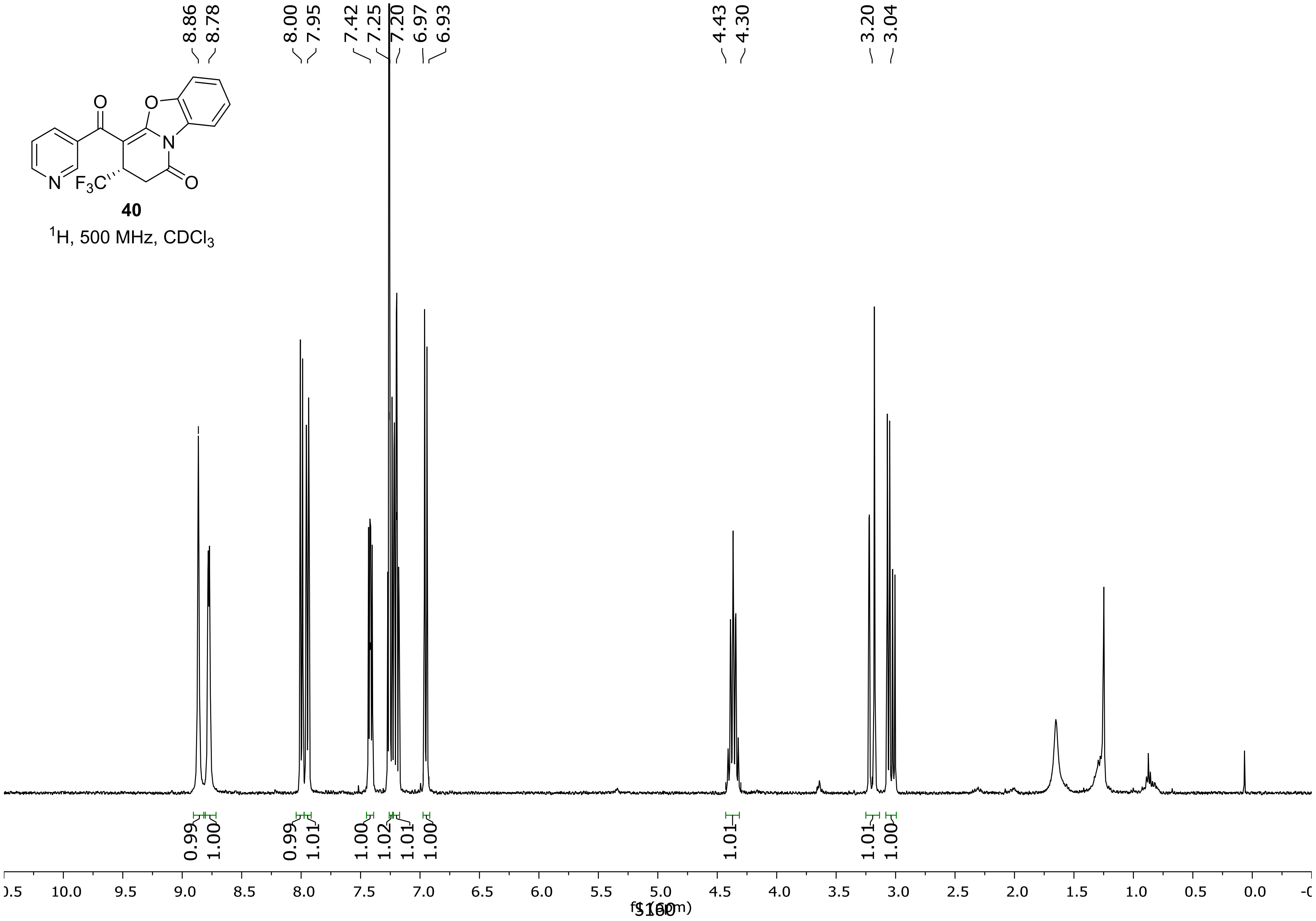
8.86
8.78
8.00
7.95
7.42
7.25
7.20
6.97
6.93
4.43
4.30
3.20
3.04
O
O
N
N
F_3C
O
40
^1H, 500 MHz, $CDCl_3$
0.99
1.00
0.99
1.01
1.00
1.02
1.01
1.00
1.01
1.01
1.00
10.0
9.5
9.0
8.5
8.0
7.5
7.0
6.5
6.0
5.5
5.0
4.5
4.0
3.5
3.0
2.5
2.0
1.5
1.0
0.5
0.0
f1 (ppm)

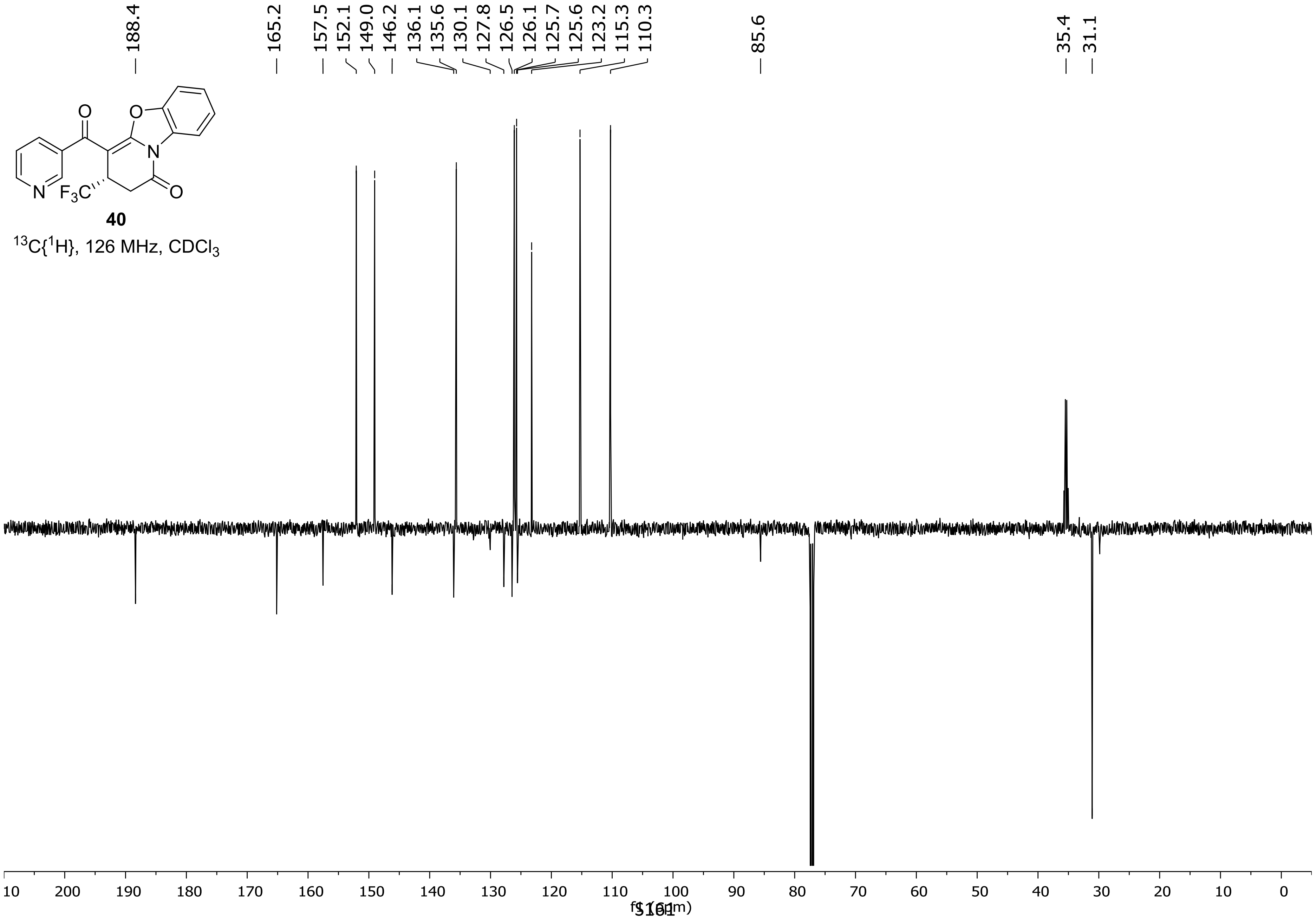
188.4
165.2
157.5
152.1
149.0
146.2
136.1
135.6
130.1
127.8
126.5
126.1
125.7
125.6
123.2
115.3
110.3
85.6
35.4
31.1
40
$^{13}C\{^{1}H\}$, 126 MHz, $CDCl_3$
f1 (ppm)

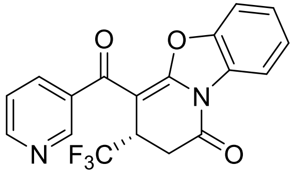

40

$^{19}F\{^1H\}$, 376 MHz, $CDCl_3$

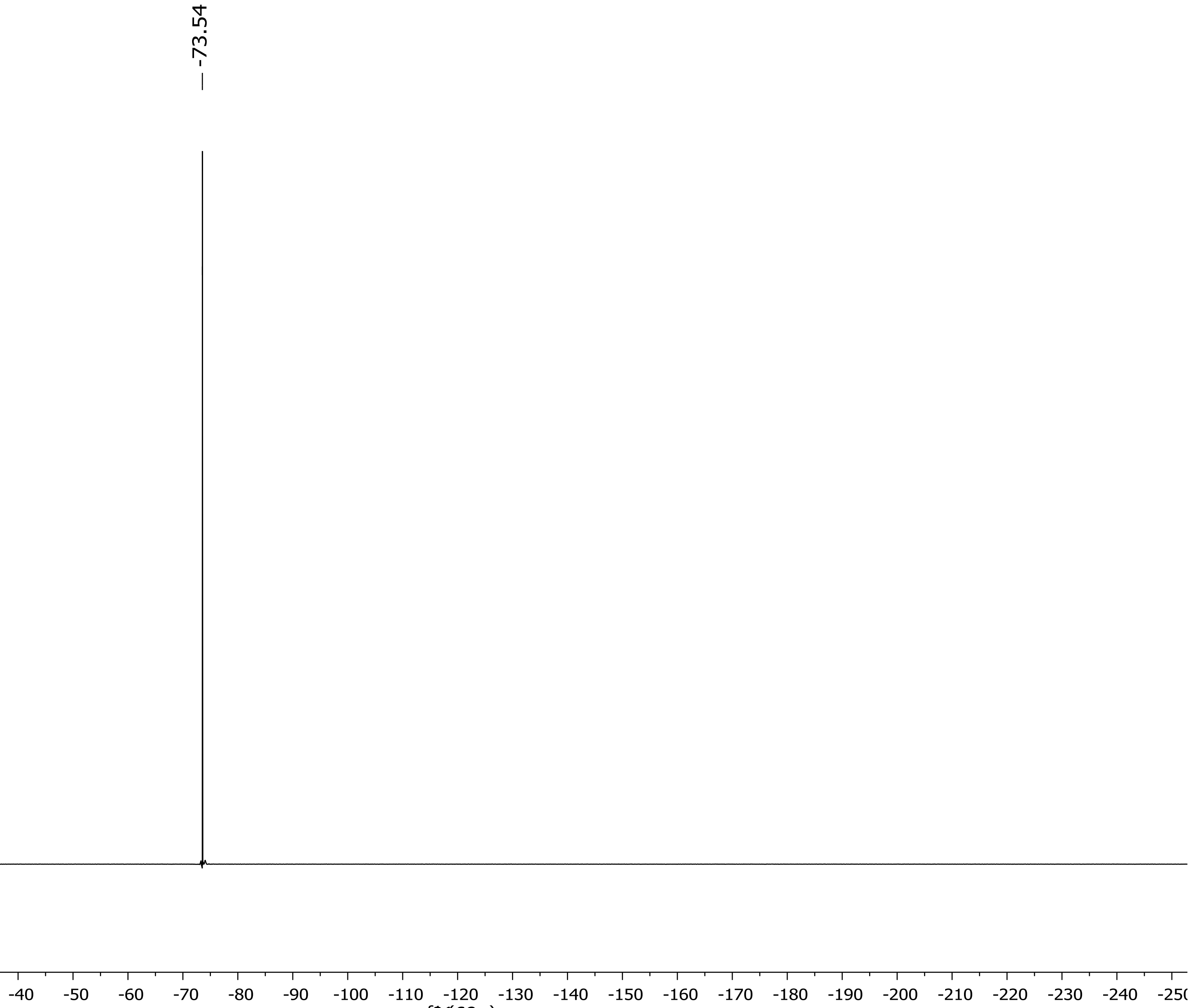

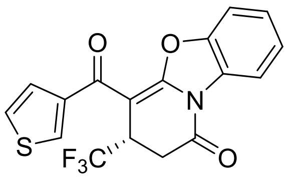

41

^{1}H, 500 MHz, $CDCl_3$

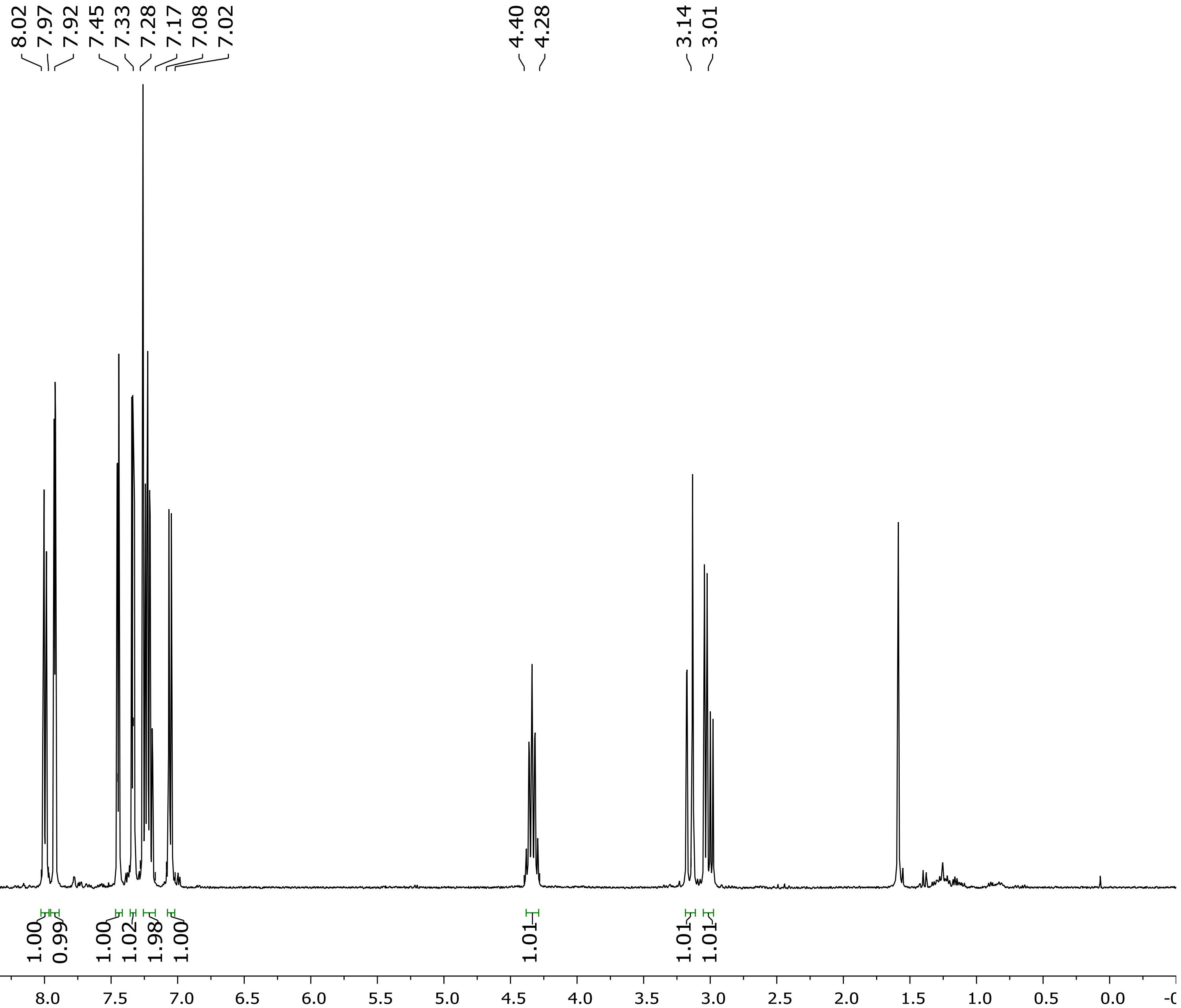

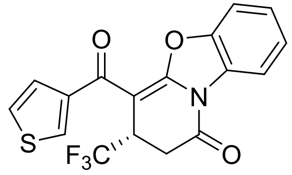

41

$^{13}C\{^1H\}$, 126 MHz, $CDCl_3$

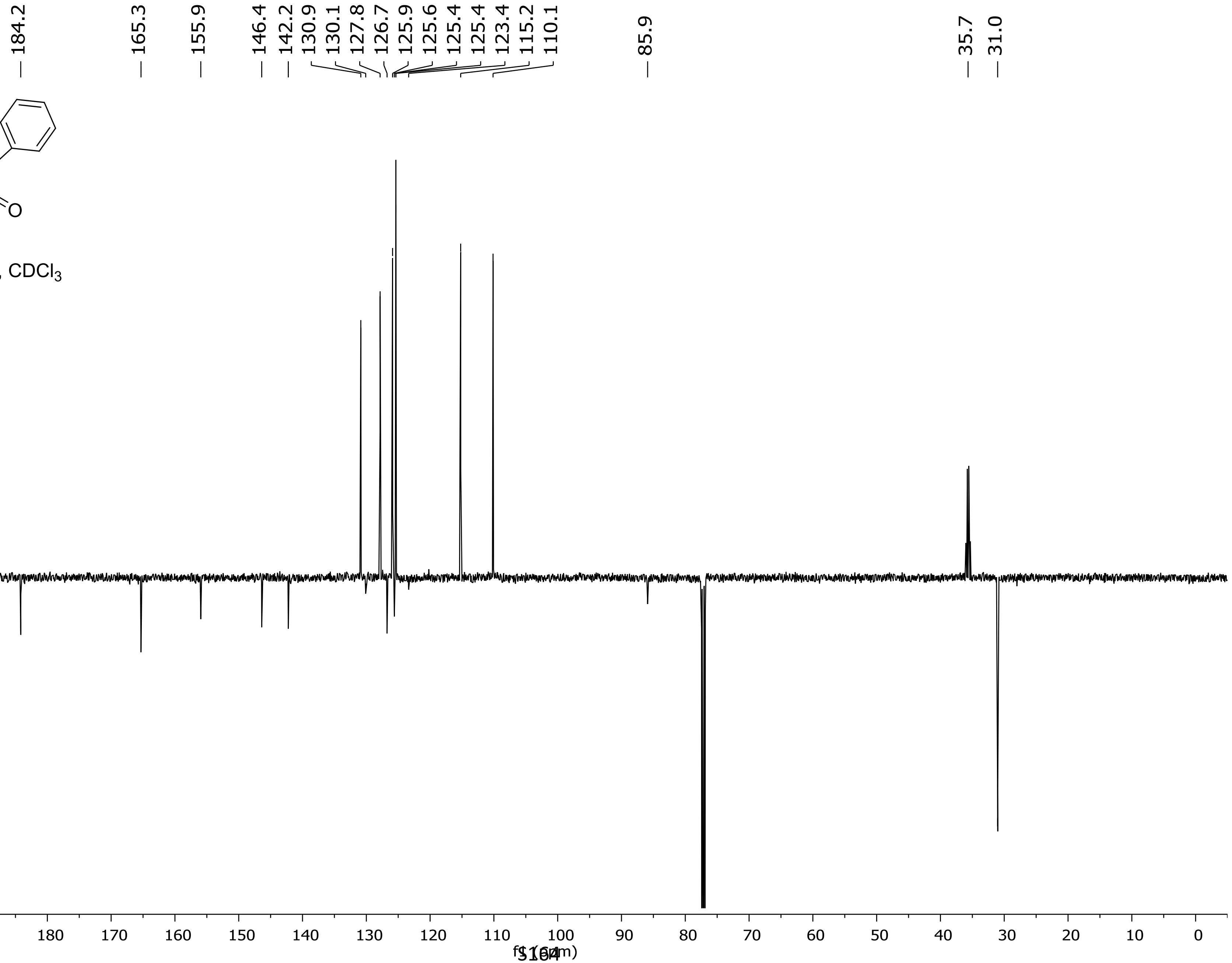

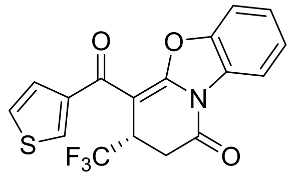

41

$^{19}F\{^{1}H\}$, 376 MHz, $CDCl_3$

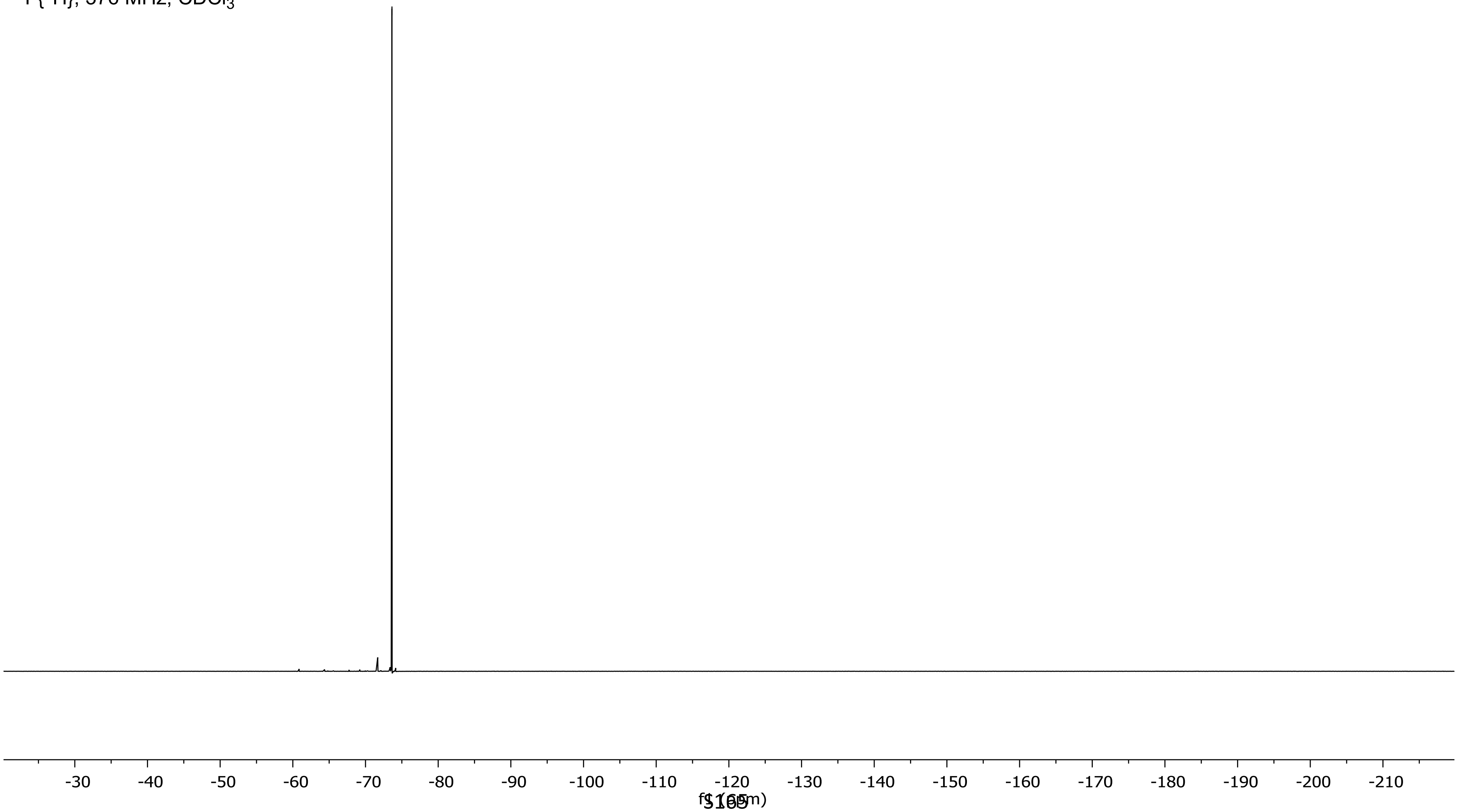

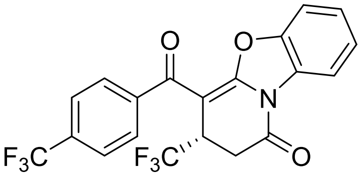

42

^{1}H, 500 MHz, $CDCl_3$

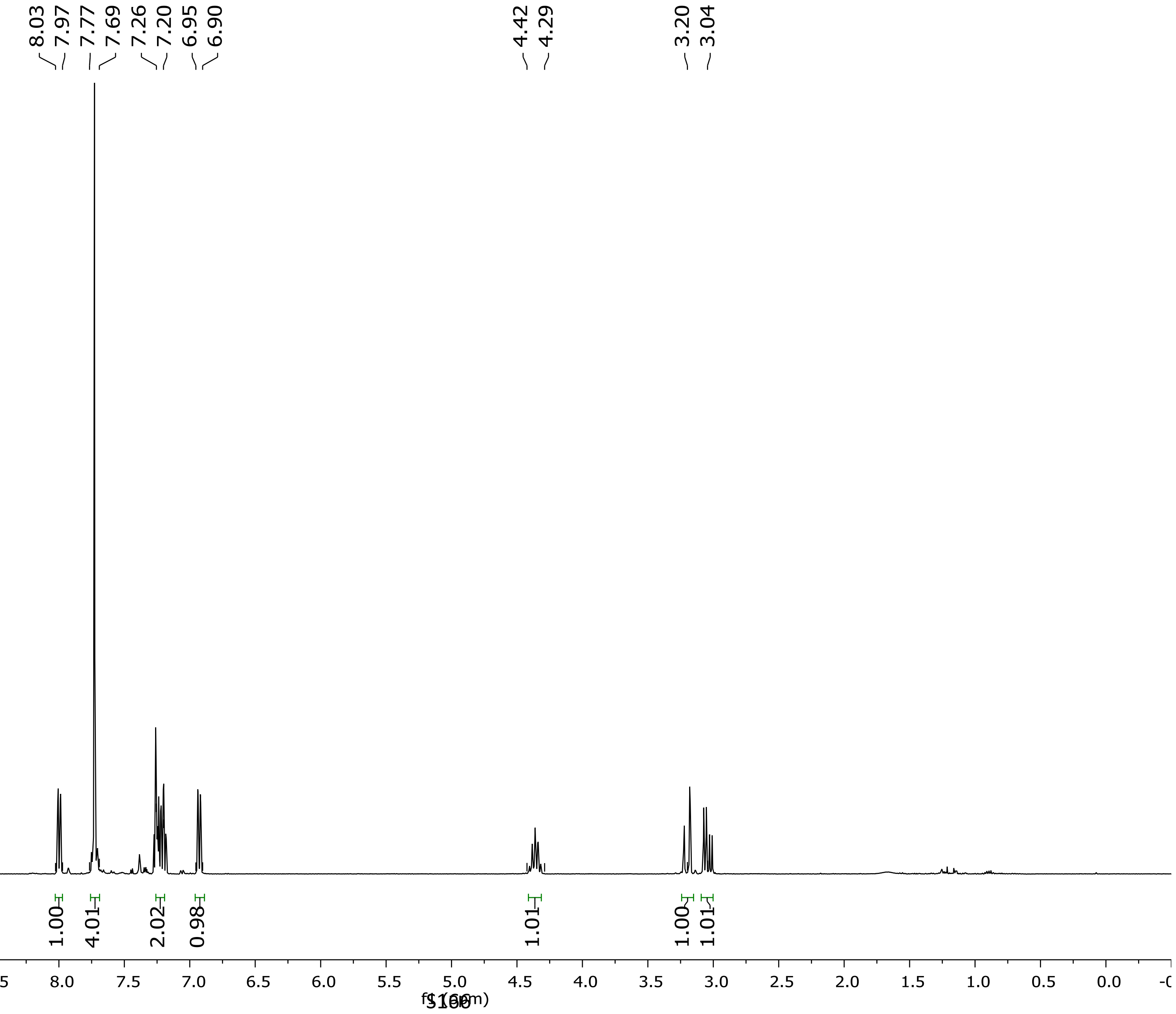

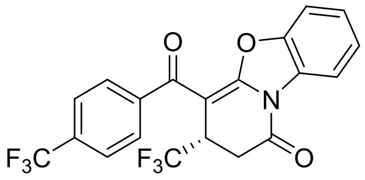

42

$^{13}C\{^{1}H\}$, 126 MHz, $CDCl_3$

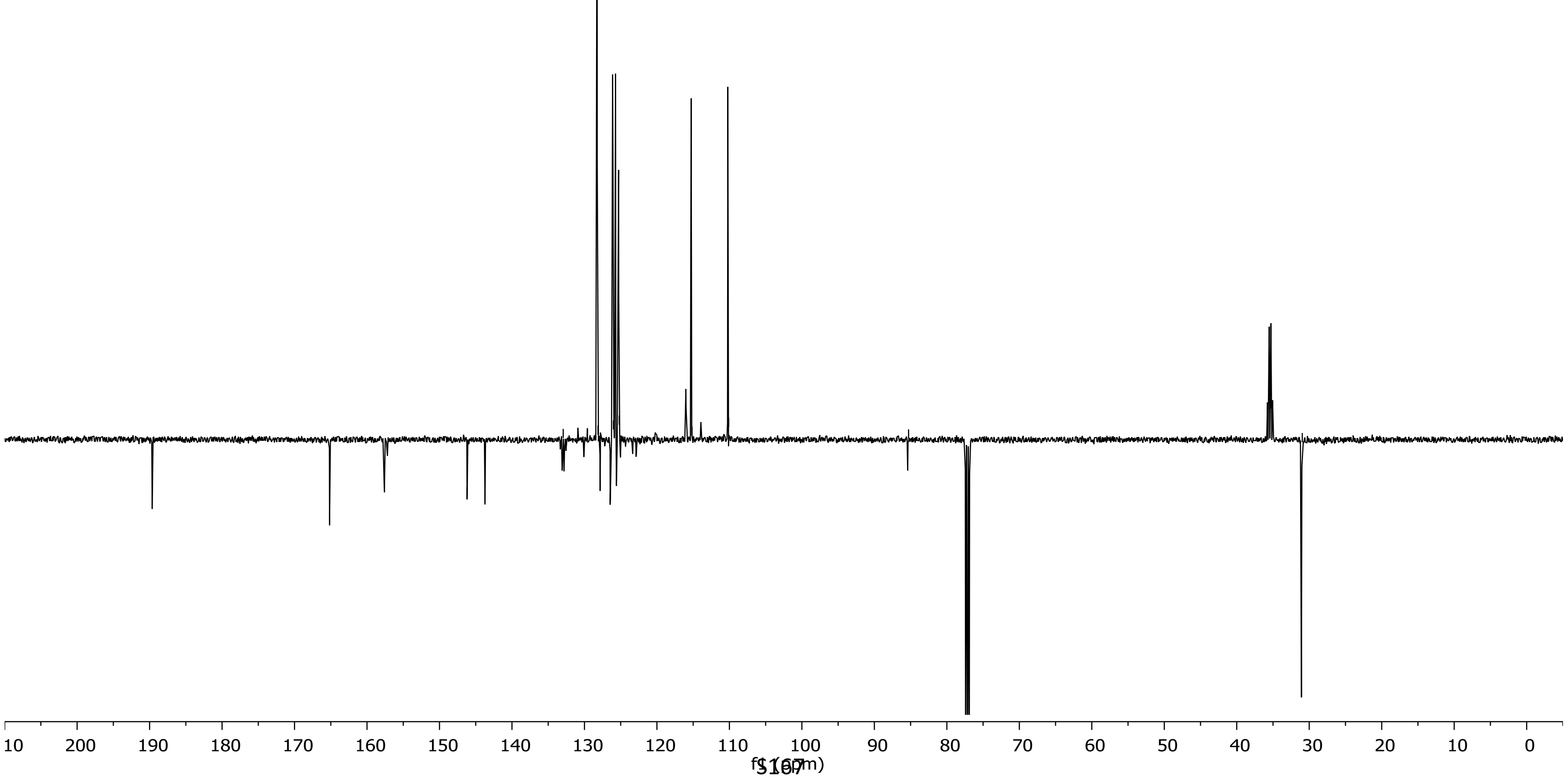

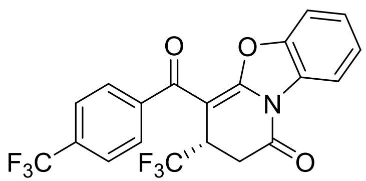

42

$^{19}F\{^{1}H\}$, 376 MHz, $CDCl_3$

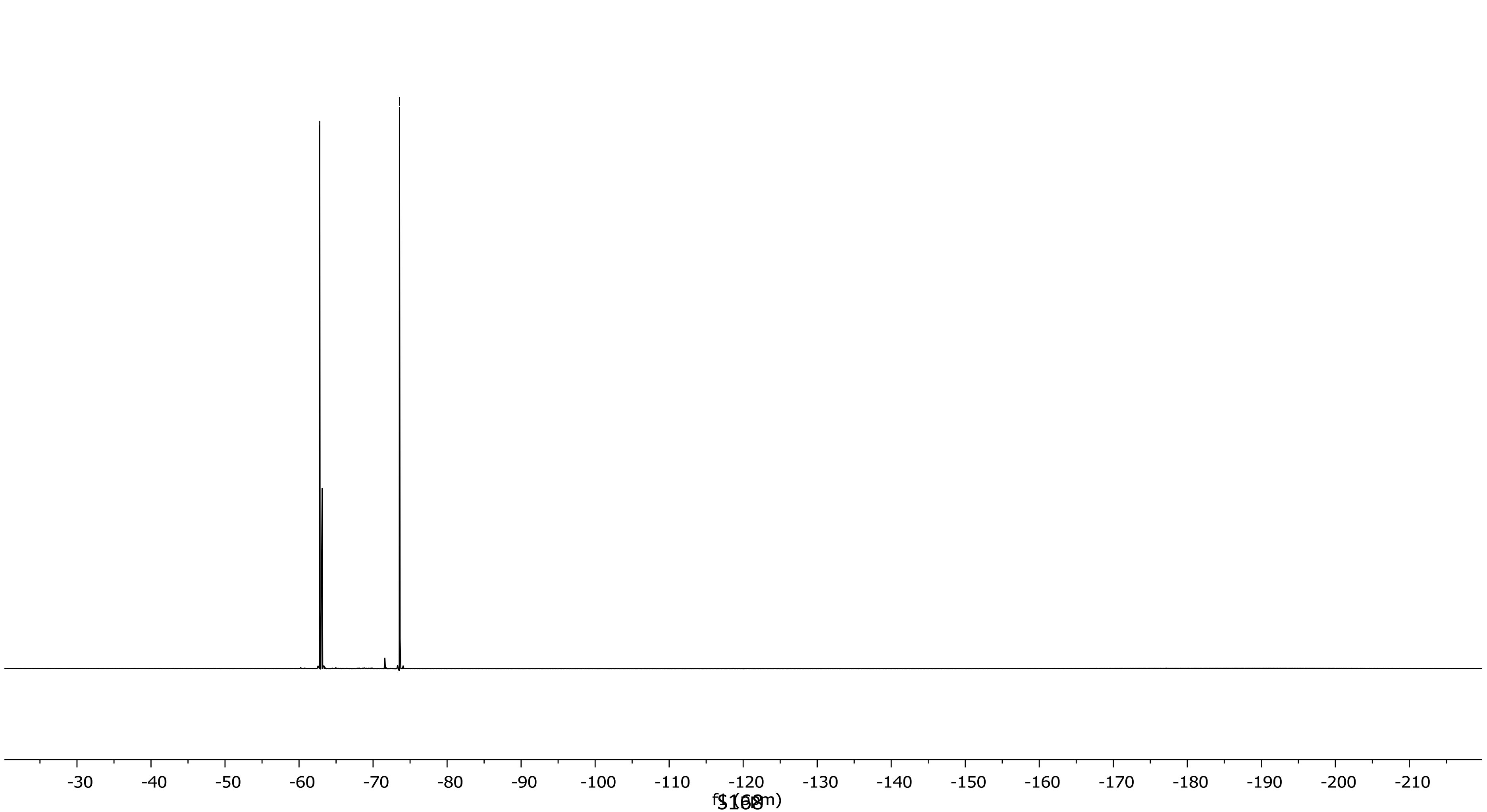

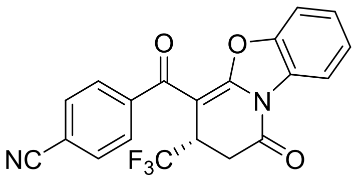

43

^1H, 500 MHz, $CDCl_3$

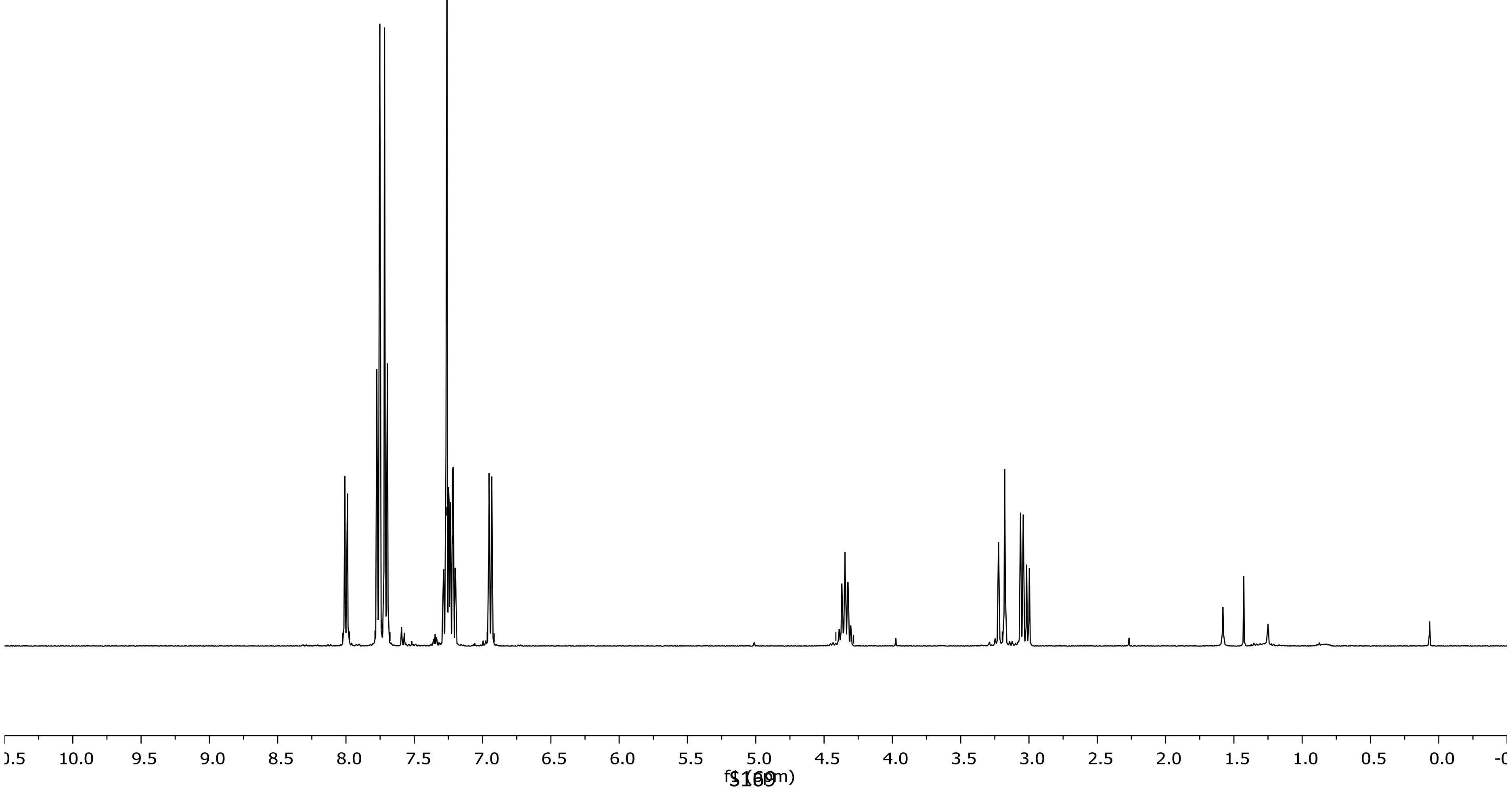

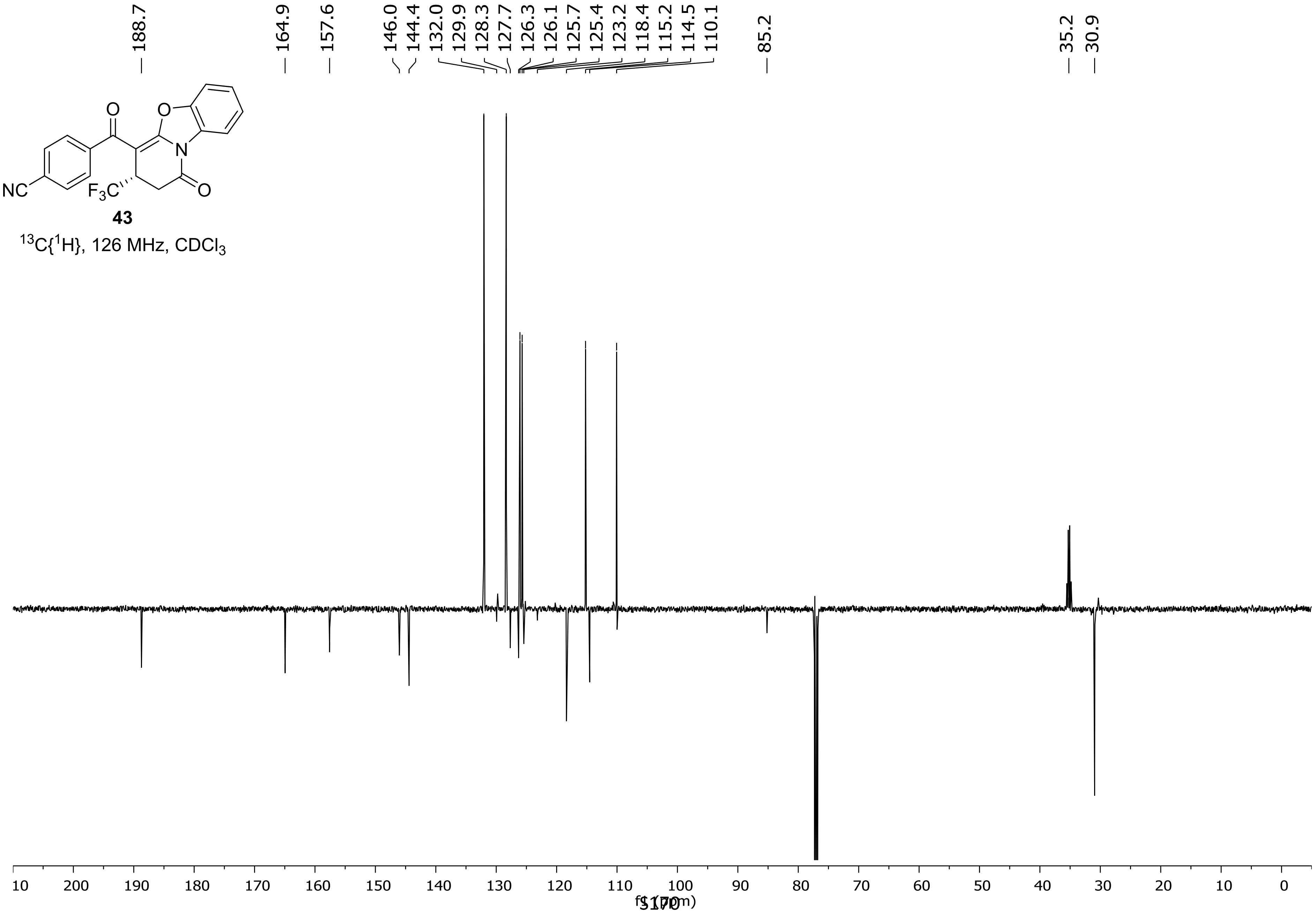
188.7
164.9
157.6
146.0
144.4
132.0
129.9
128.3
127.7
126.3
126.1
125.7
125.4
123.2
118.4
115.2
114.5
110.1
85.2
35.2
30.9
O
O
N
O
NC
F_3C
43
$^{13}C\{^{1}H\}$, 126 MHz, $CDCl_3$
210 200 190 180 170 160 150 140 130 120 110 100 90 80 70 60 50 40 30 20 10 0
f1 (ppm)

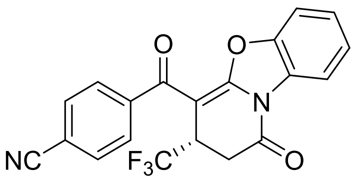

43

$^{19}F\{^{1}H\}$, 376 MHz, $CDCl_3$

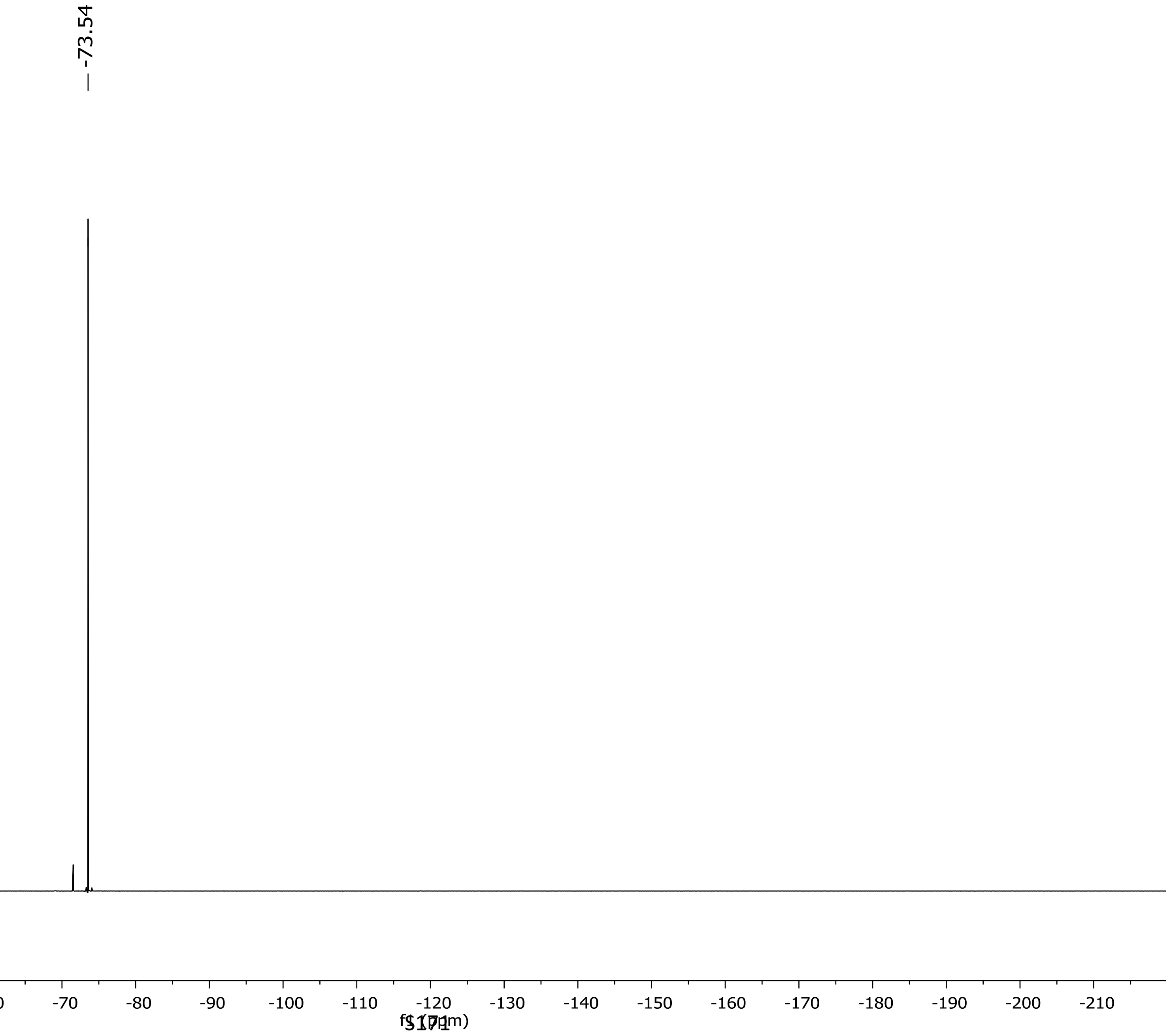

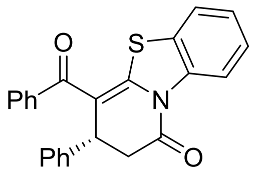

44

^{1}H, 500 MHz, $CDCl_3$

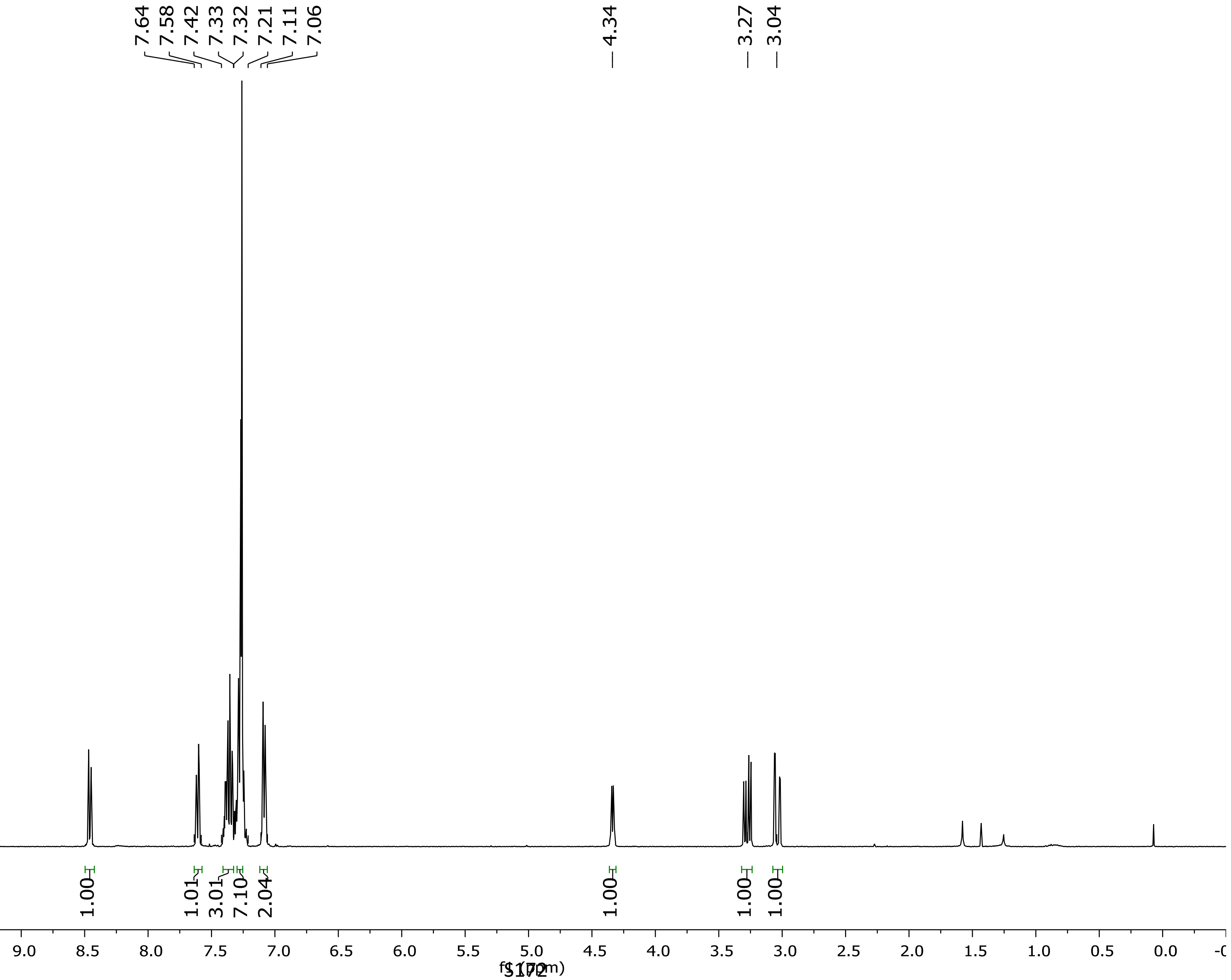

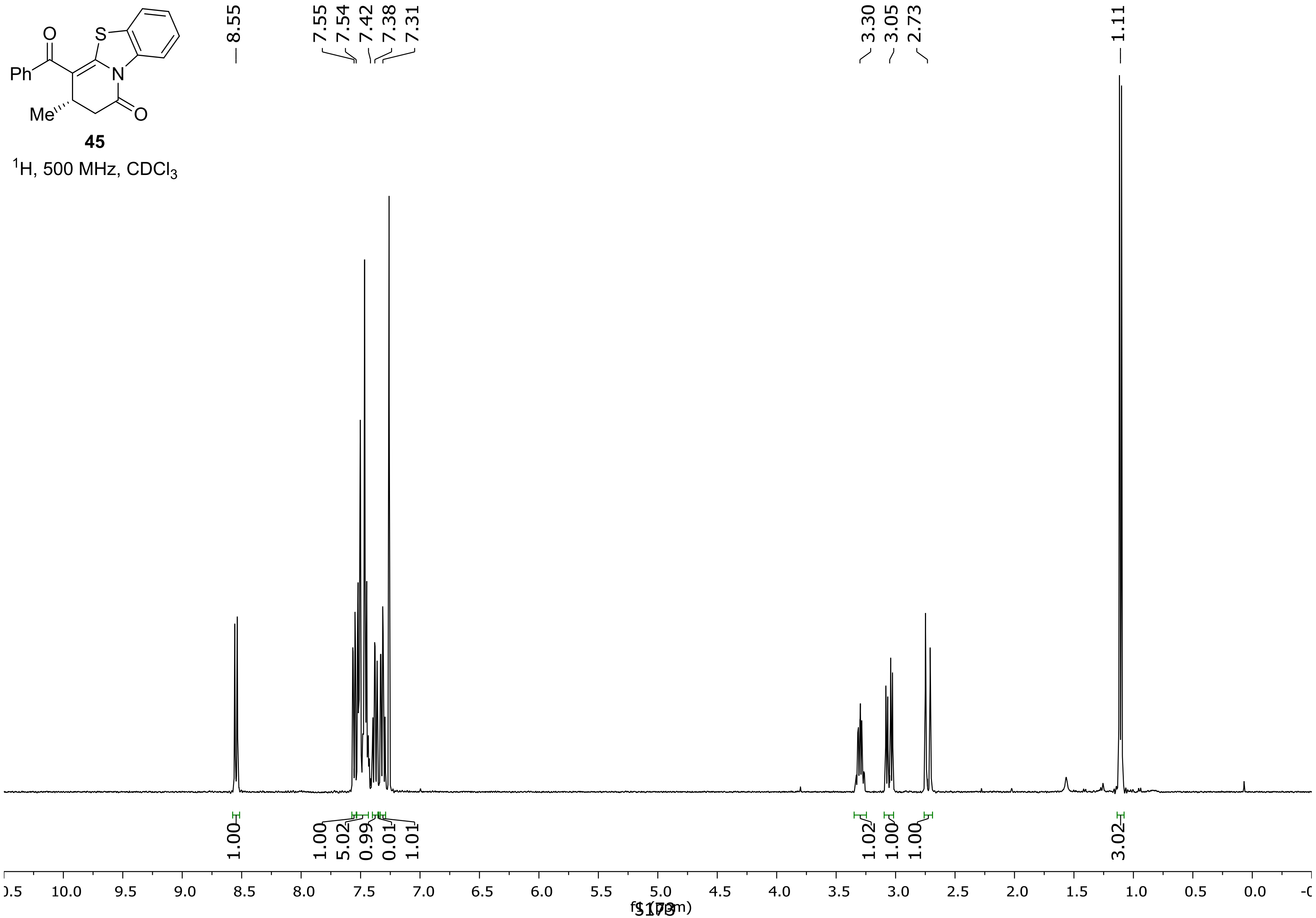

45
1H, 500 MHz, CDCl3
8.55
7.55
7.54
7.42
7.38
7.31
3.30
3.05
2.73
1.11
1.00
1.00
5.02
0.99
0.01
1.01
1.02
1.00
1.00
3.02
f1 (ppm)

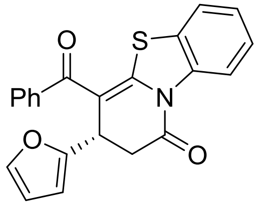

46

^{1}H, 500 MHz, $CDCl_3$

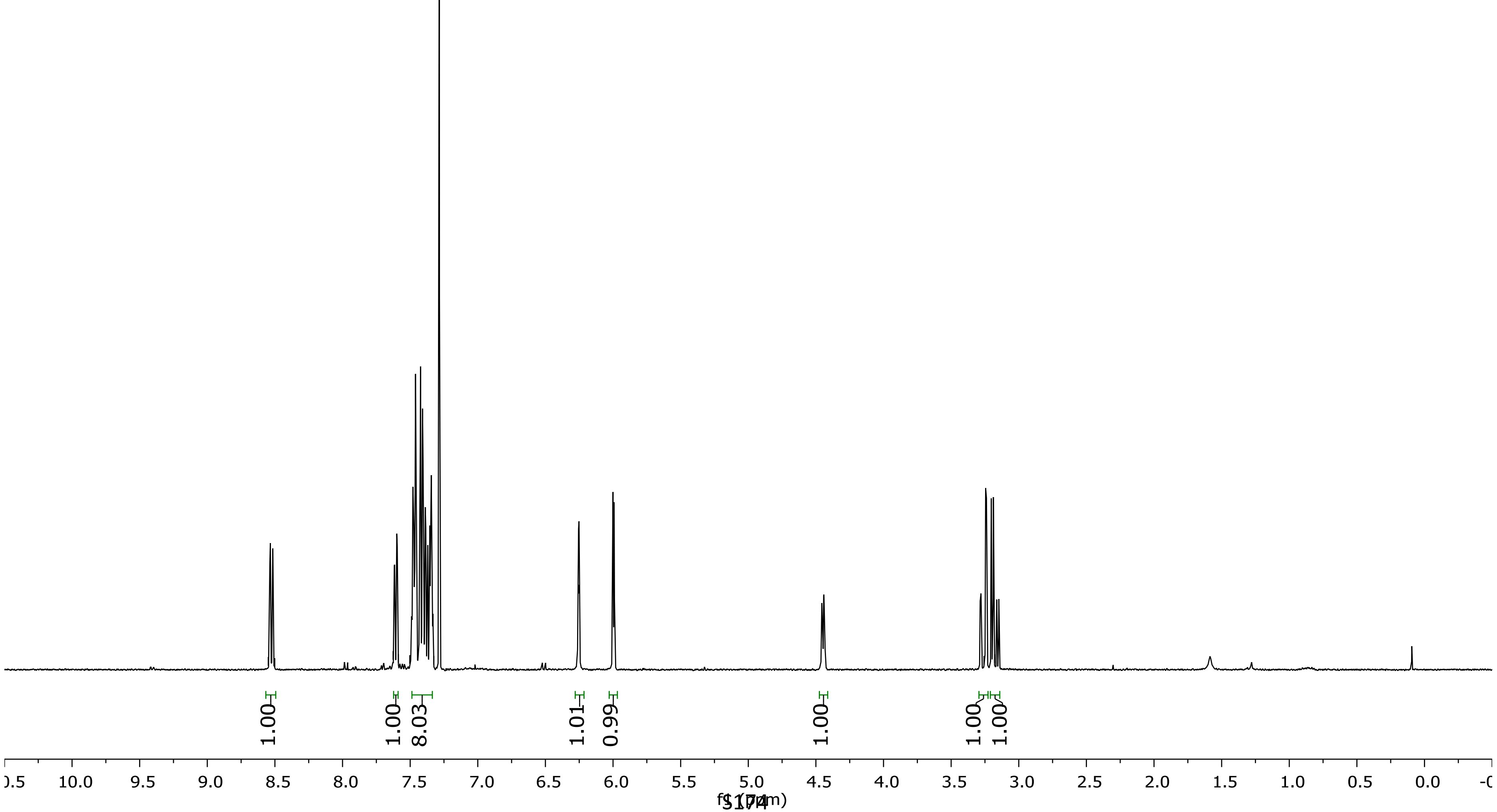

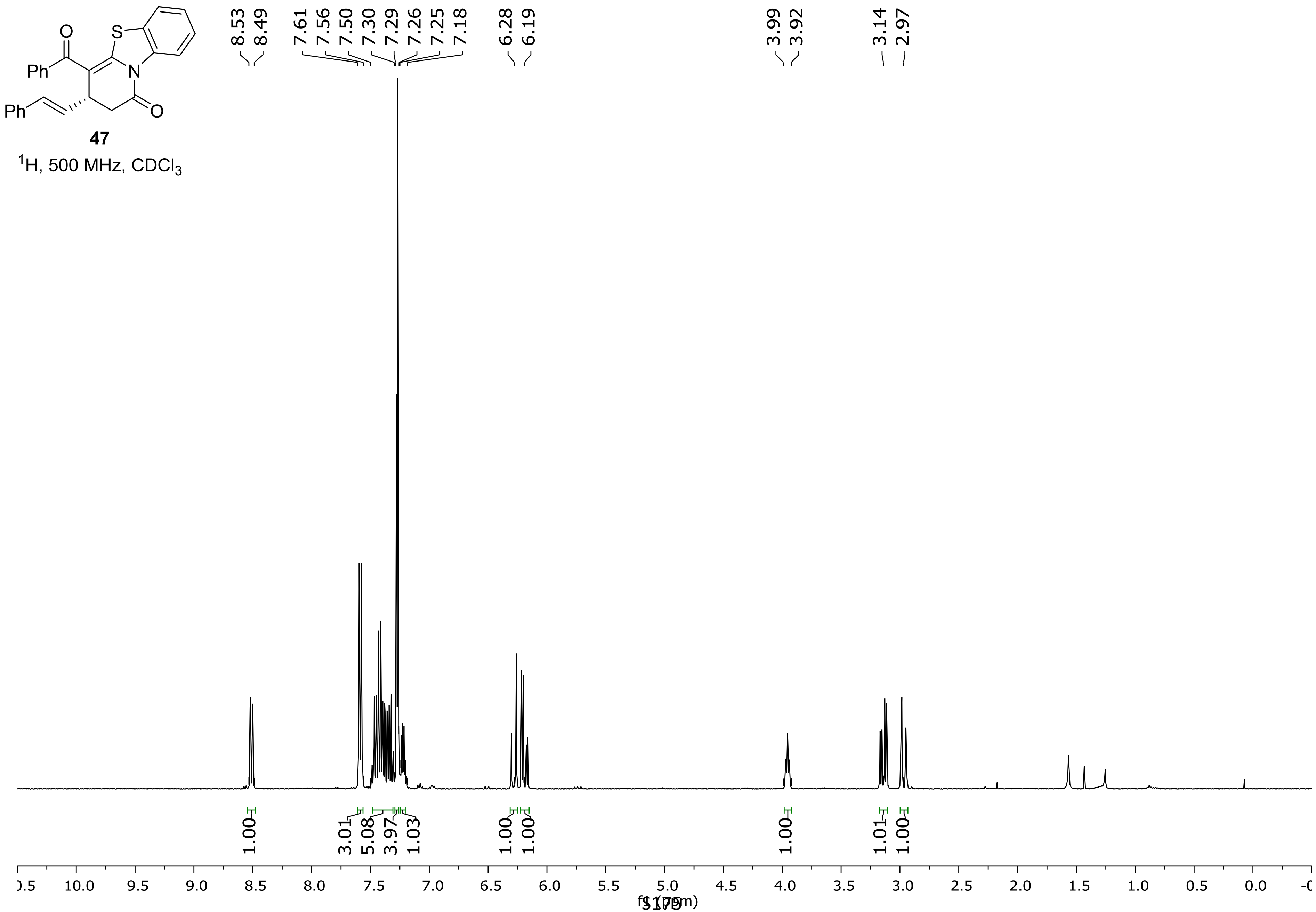

O
S
N
Ph
Ph
O
47
^{1}H, 500 MHz, $CDCl_3$
8.53
8.49
7.61
7.56
7.50
7.30
7.29
7.26
7.25
7.18
6.28
6.19
3.99
3.92
3.14
2.97
1.00
3.01
5.08
3.97
1.03
1.00
1.00
1.00
1.01
1.00
10.0 9.5 9.0 8.5 8.0 7.5 7.0 6.5 6.0 5.5 5.0 4.5 4.0 3.5 3.0 2.5 2.0 1.5 1.0 0.5 0.0
f1 (ppm)

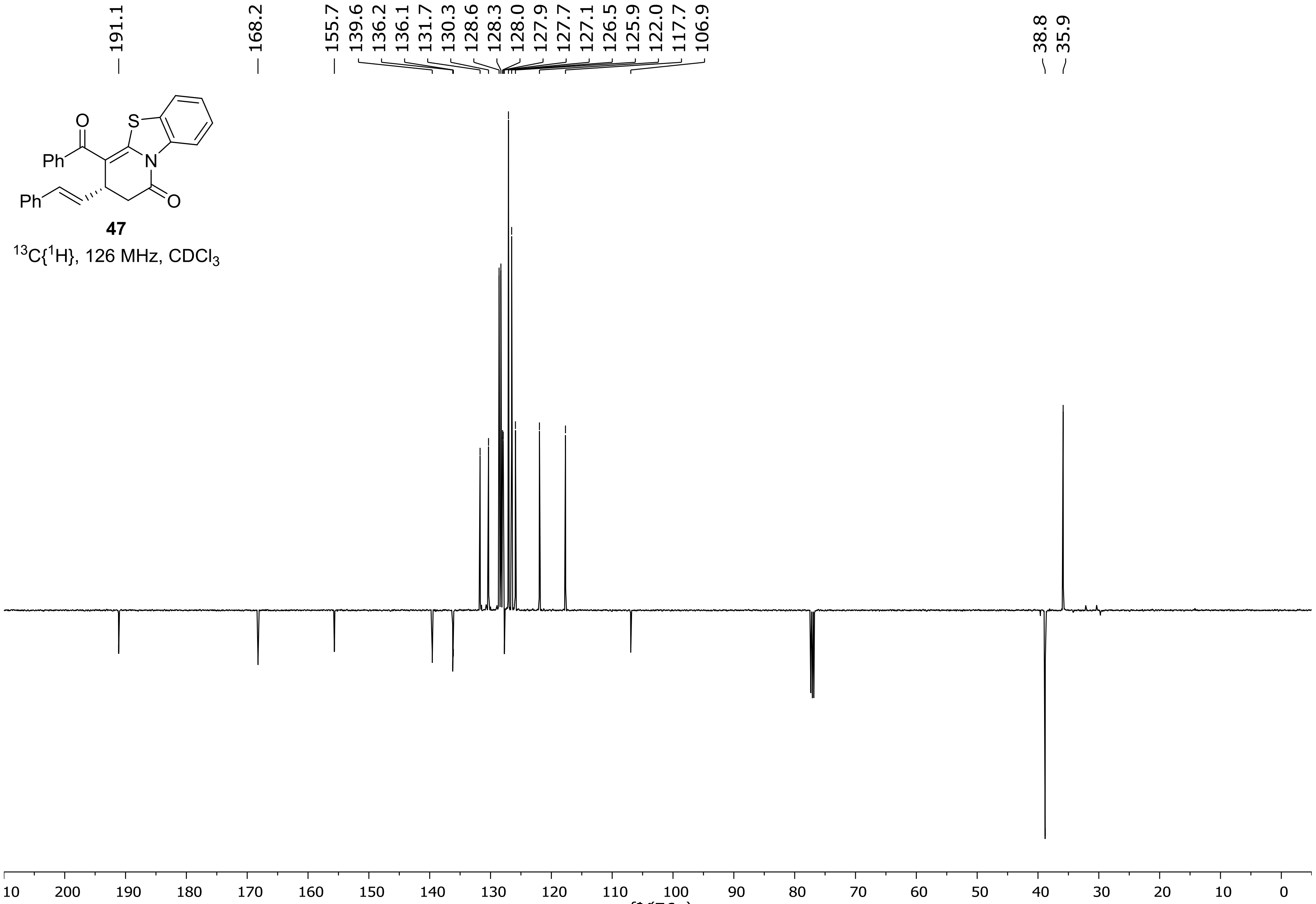
191.1
168.2
155.7
139.6
136.2
136.1
131.7
130.3
128.6
128.3
128.0
127.9
127.7
127.1
126.5
125.9
122.0
117.7
106.9
38.8
35.9
O
S
Ph
N
Ph
O
47
$^{13}C\{^1H\}$, 126 MHz, $CDCl_3$
10
200
190
180
170
160
150
140
130
120
110
100
90
80
70
60
50
40
30
20
10
0
f1 (ppm)

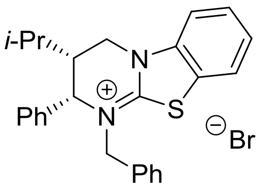

S15

^{1}H, 400 MHz, CD_2Cl_2

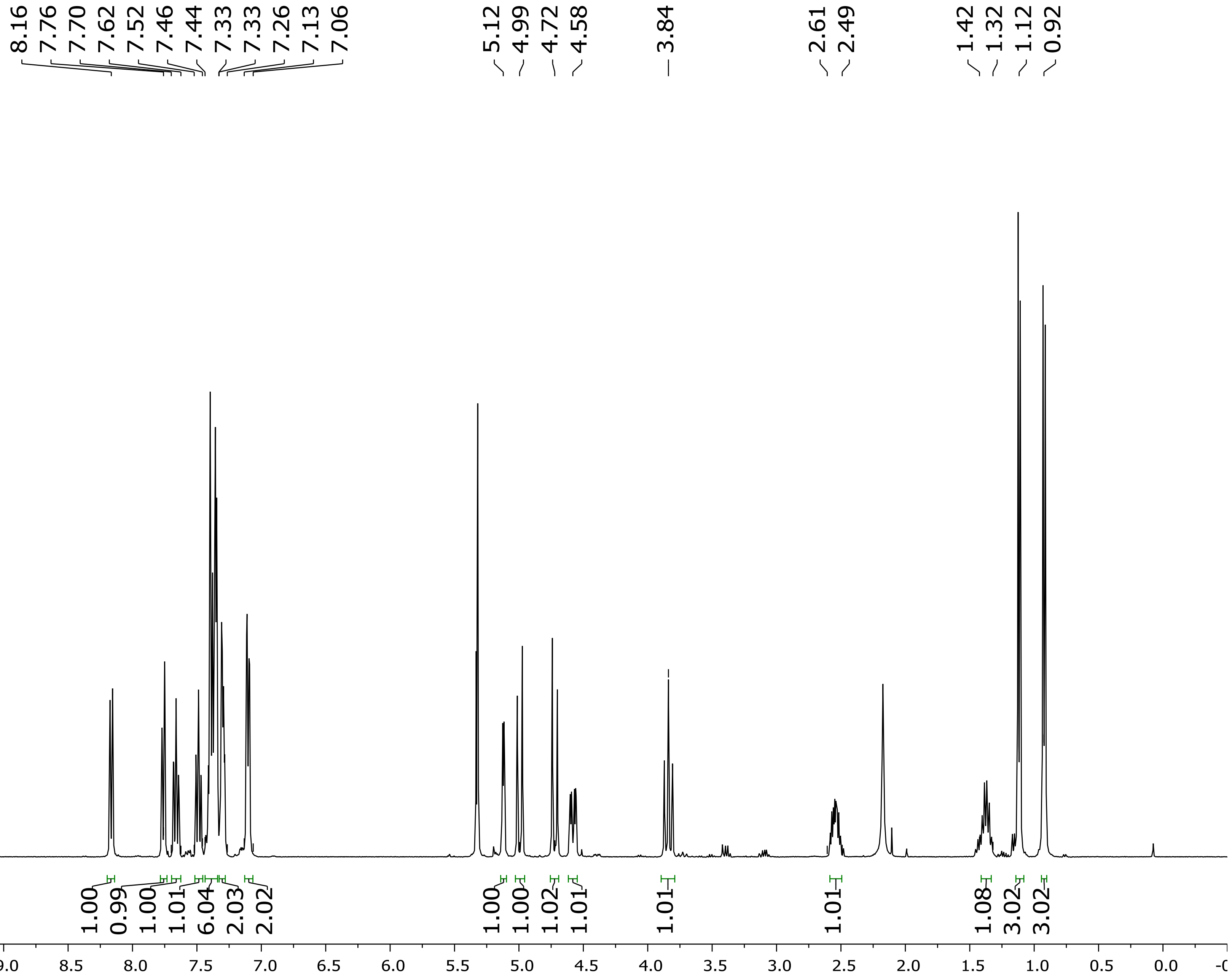

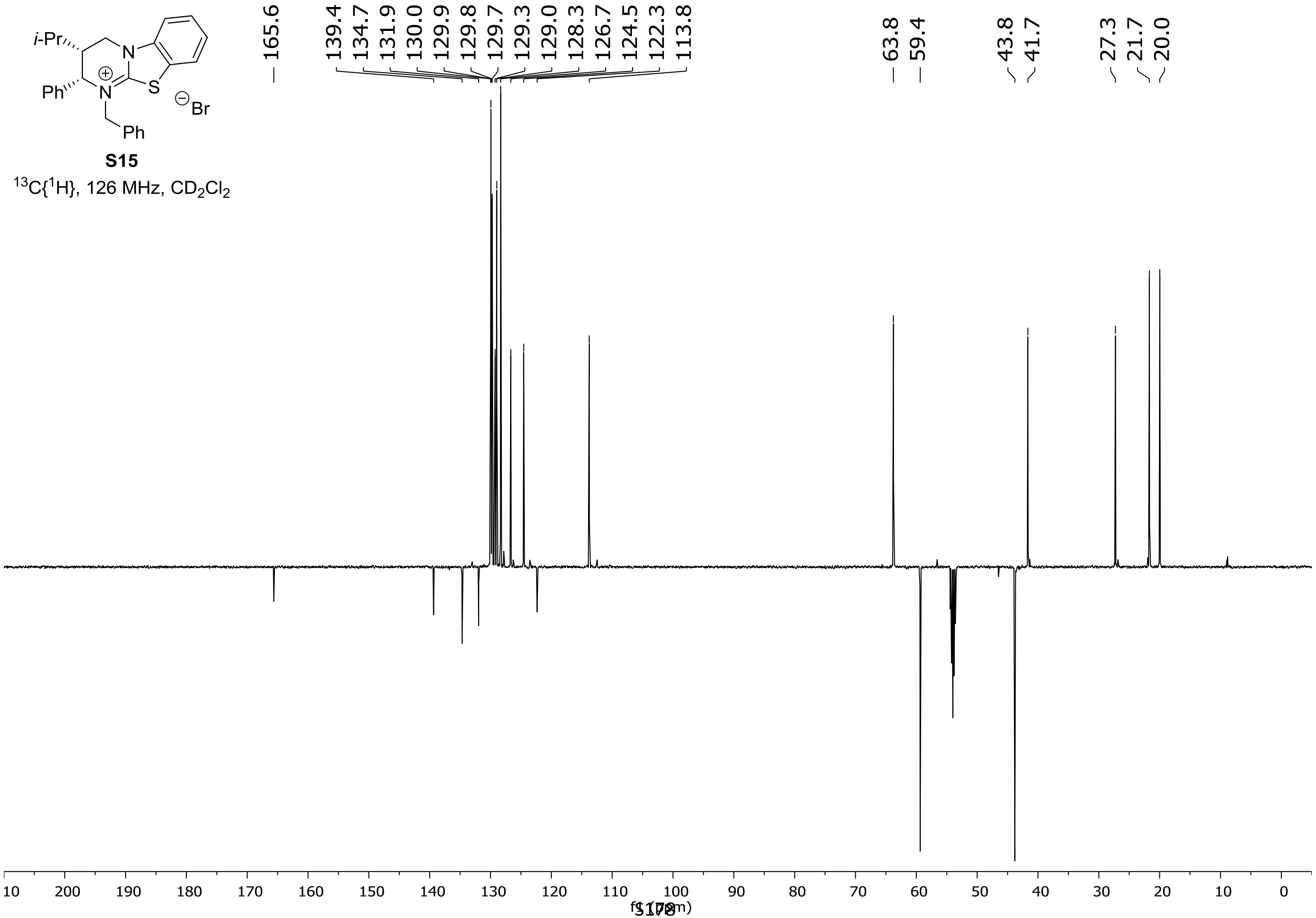
i-Pr
Ph
N
S
Br
Ph
S15
$^{13}C\{^1H\}$, 126 MHz, CD_2Cl_2
165.6
139.4
134.7
131.9
130.0
129.9
129.8
129.7
129.3
129.0
128.3
126.7
124.5
122.3
113.8
63.8
59.4
43.8
41.7
27.3
21.7
20.0
10 200 190 180 170 160 150 140 130 120 110 100 90 80 70 60 50 40 30 20 10 0
f1 (ppm)

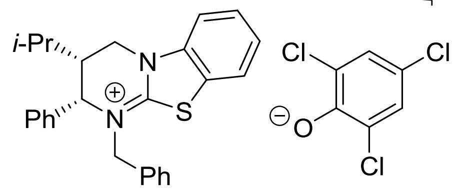

49

^{1}H, 500 MHz, $CDCl_3$

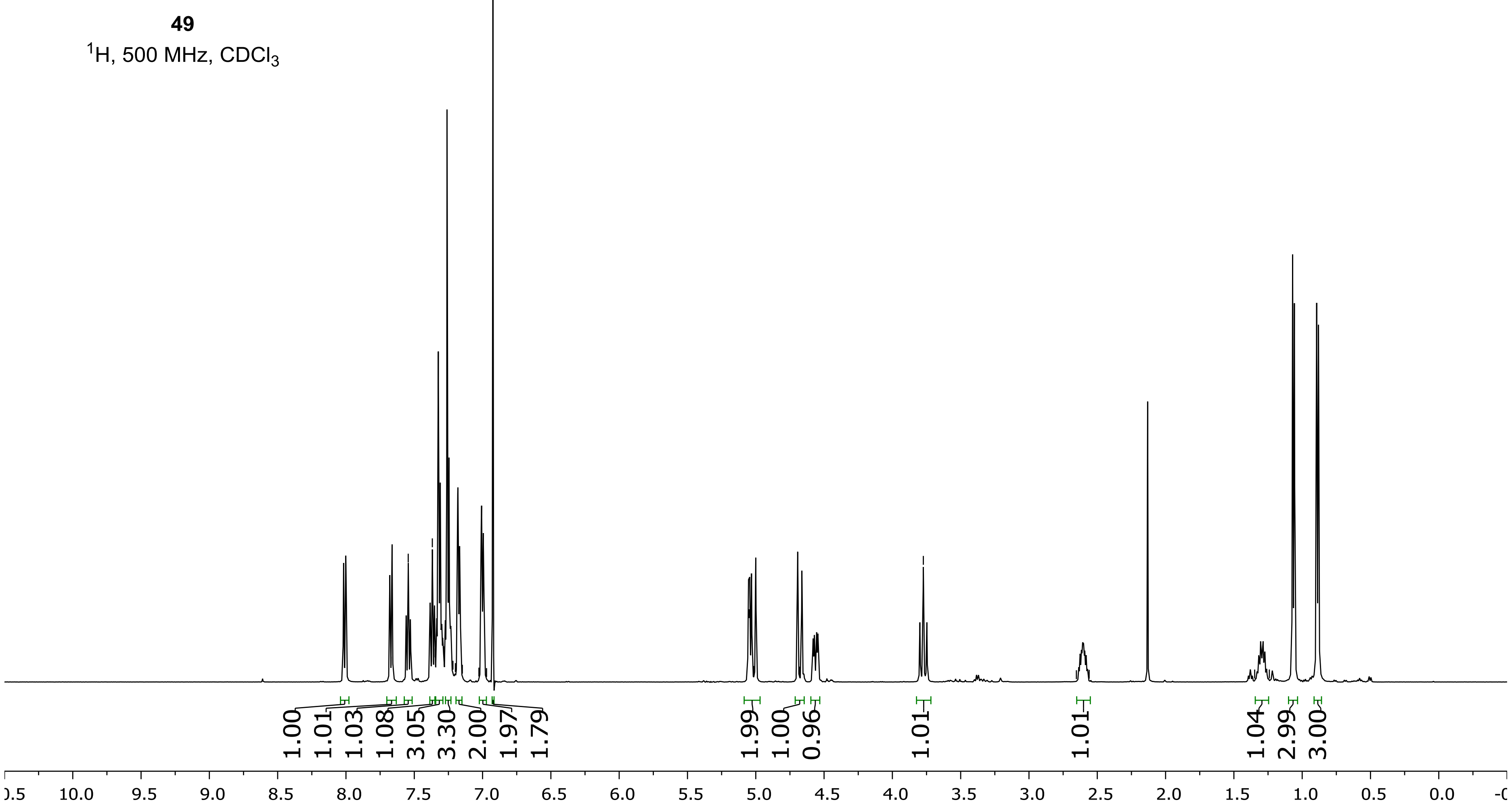

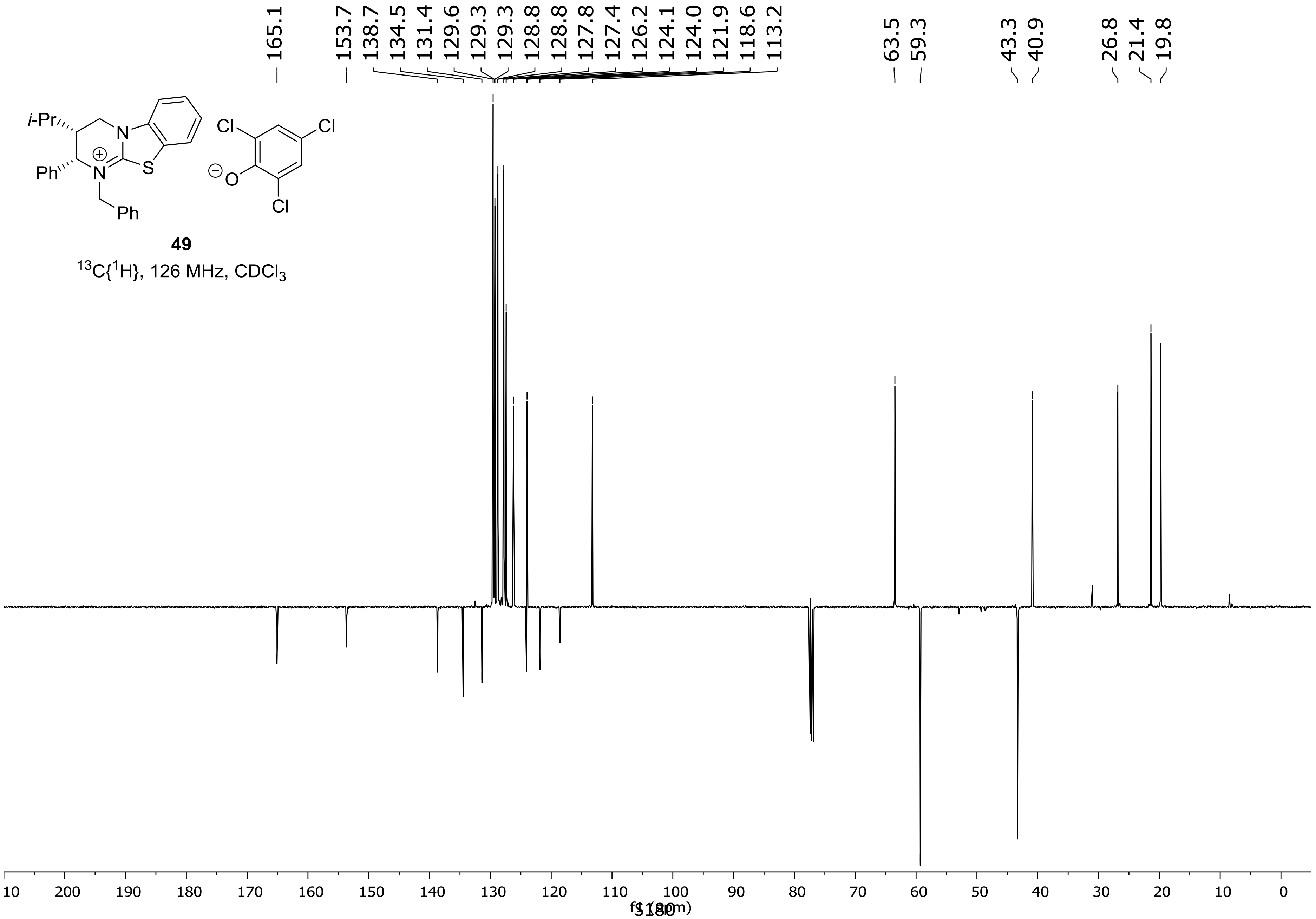

49

$^{13}C\{^1H\}$, 126 MHz, $CDCl_3$

(*S*)-4-Benzoyl-3-(trifluoromethyl)-2,3-dihydro-1*H*-benzo[4,5]thiazolo[3,2-*a*]pyridin-1-one **10**

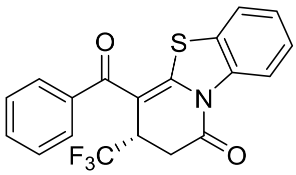

Chiral HPLC analysis: CHIRALPAK AD-H (20:80 *i*-PrOH:hexane, flow rate 1.0 mL min^{-1}, 254 nm, 30 °C), t_R (*R*): 12.3 min, t_R (*S*): 18.3 min, 96.6:3.4 (*S*:*R*) er

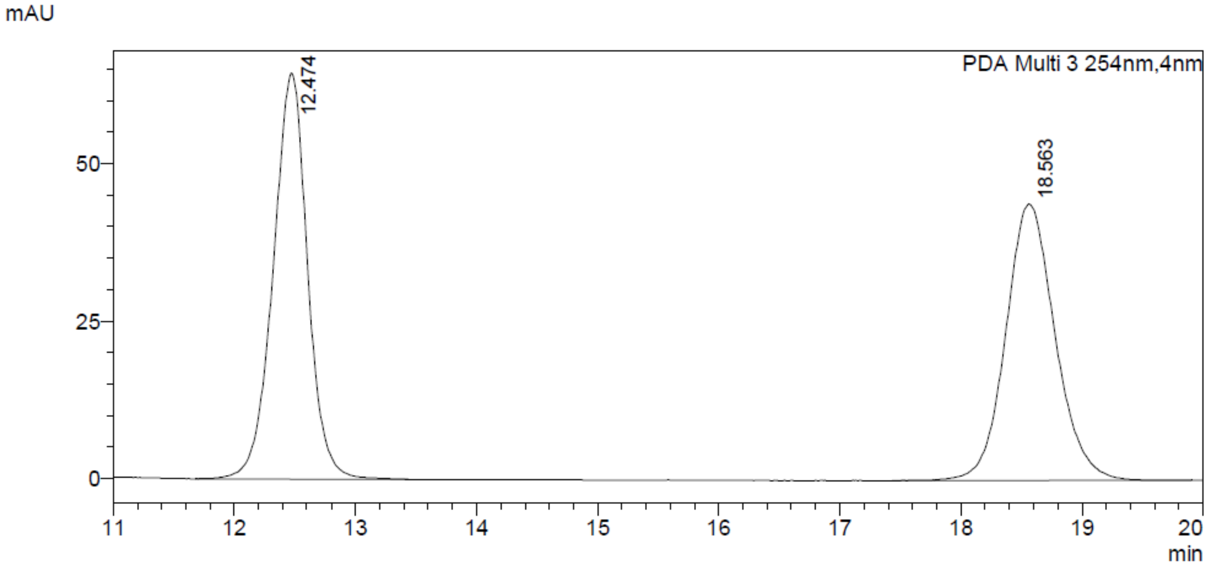

PDA Ch3 254nm

Peak#	Ret. Time	Area%
1	12.474	50.568
2	18.563	49.432
Total		100.000

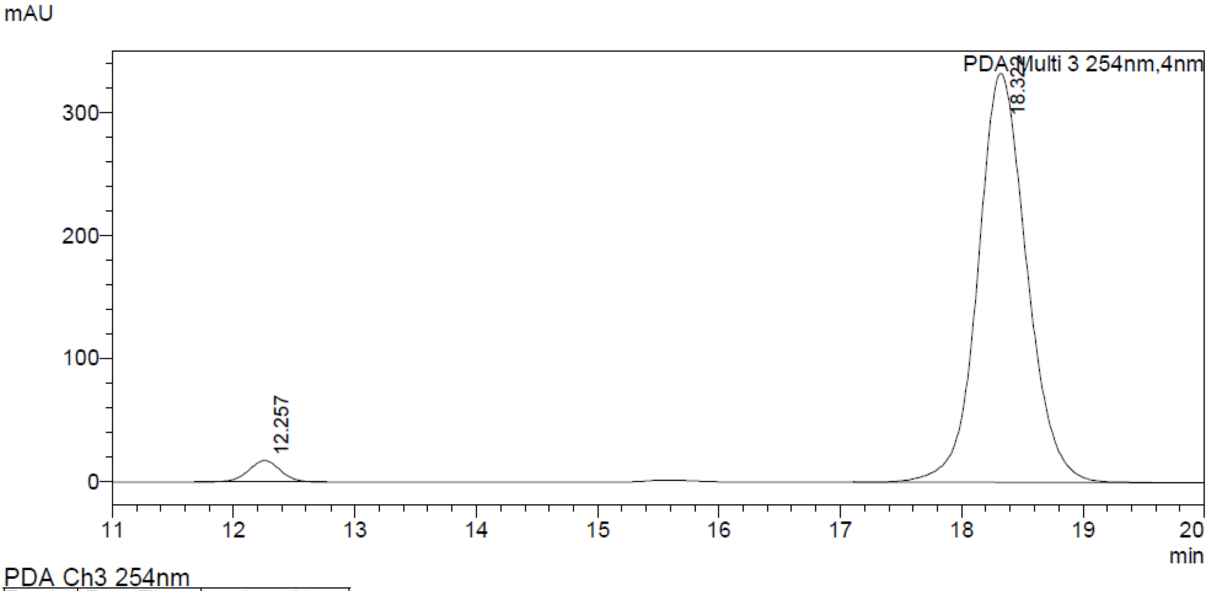

PDA Ch3 254nm

Peak#	Ret. Time	Area%
1	12.257	3.389
2	18.322	96.611
Total		100.000

(*S*)-4-(4-Methoxybenzoyl)-3-(trifluoromethyl)-2,3-dihydro-1*H*-benzo[4,5]thiazolo[3,2-*a*]pyridin-1-one **16**

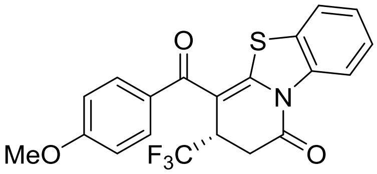

Chiral HPLC analysis: CHIRALCEL OJ-H (20:80 *i*-PrOH:hexane, flow rate 1.5 mL min^{-1}, 211 nm, 40 °C), t_R (*S*): 30.9 min, t_R (*R*): 43.9 min, 97.4:2.6 (*S*:*R*) er

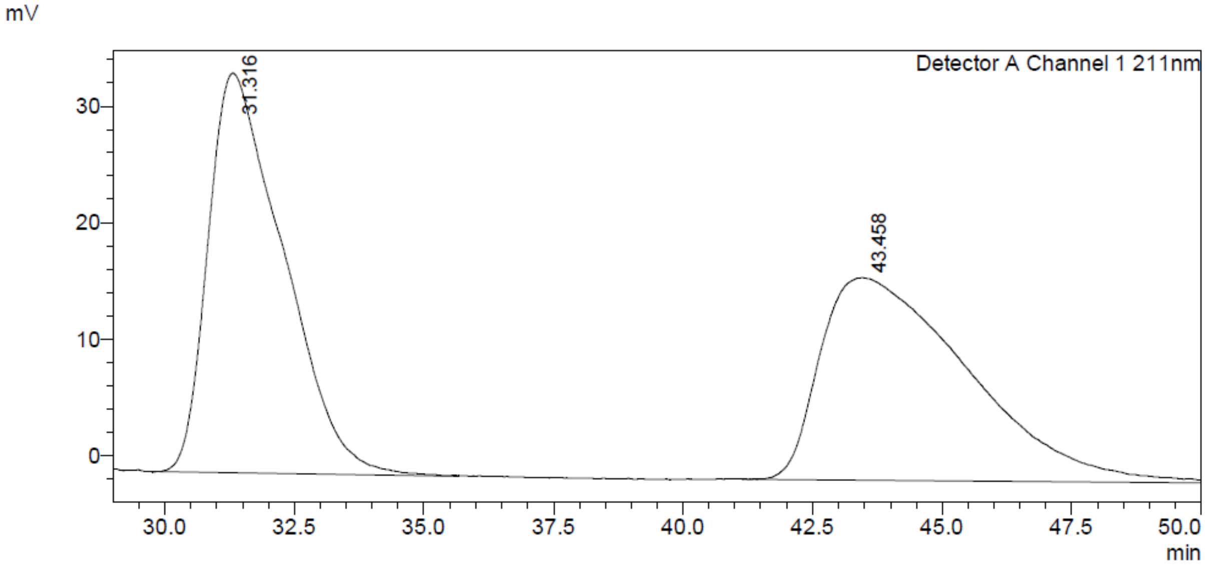

Detector A Channel 1 211nm

Peak#	Ret. Time	Area%
1	31.316	49.937
2	43.458	50.063
Total		100.000

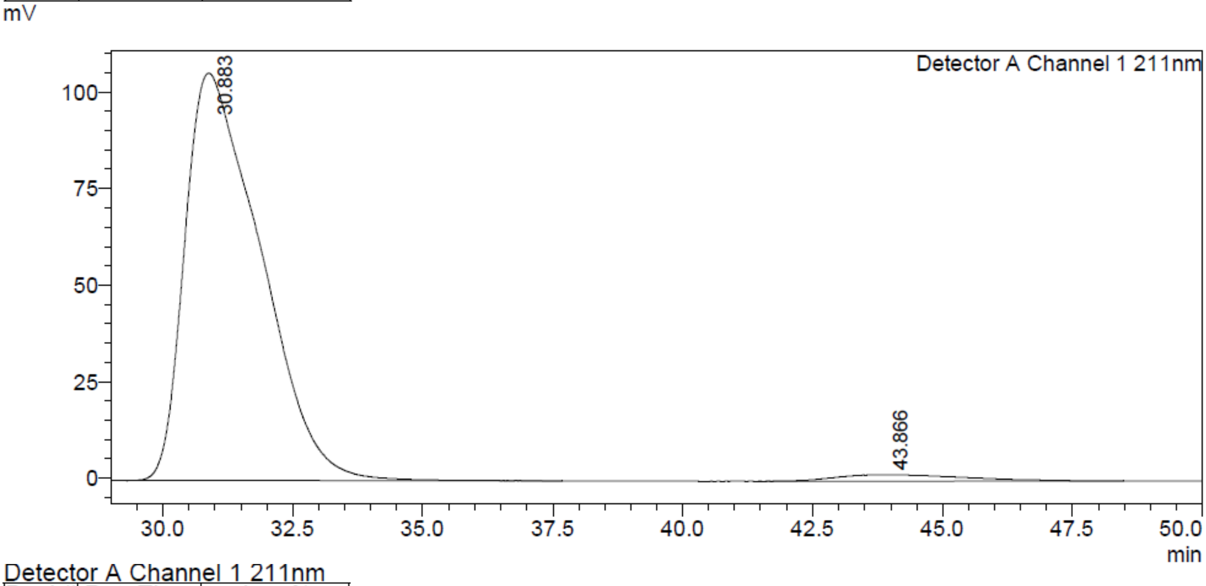

Detector A Channel 1 211nm

Peak#	Ret. Time	Area%
1	30.883	97.388
2	43.866	2.612
Total		100.000

(*S*)-4-(4-Fluorobenzoyl)-3-(trifluoromethyl)-2,3-dihydro-1*H*-benzo[4,5]thiazolo[3,2-*a*]pyridin-1-one **17**

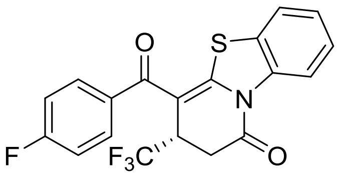

Chiral HPLC analysis: CHIRALCEL OJ-H (20:80 *i*-PrOH:hexane, flow rate 1.5 mL min^{-1}, 254 nm, 40 °C), t_R (*S*): 14.5 min, t_R (*R*): 20.1 min, 96.1:3.9 (*S*:*R*) er

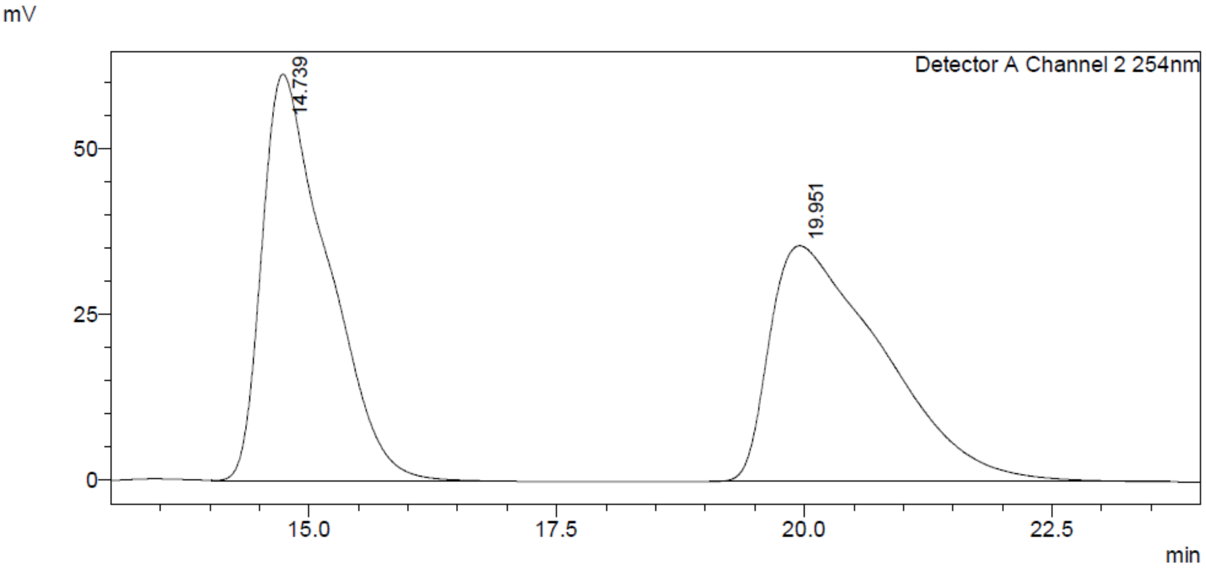

Detector A Channel 2 254nm

Peak#	Ret. Time	Area%
1	14.739	49.973
2	19.951	50.027
Total		100.000

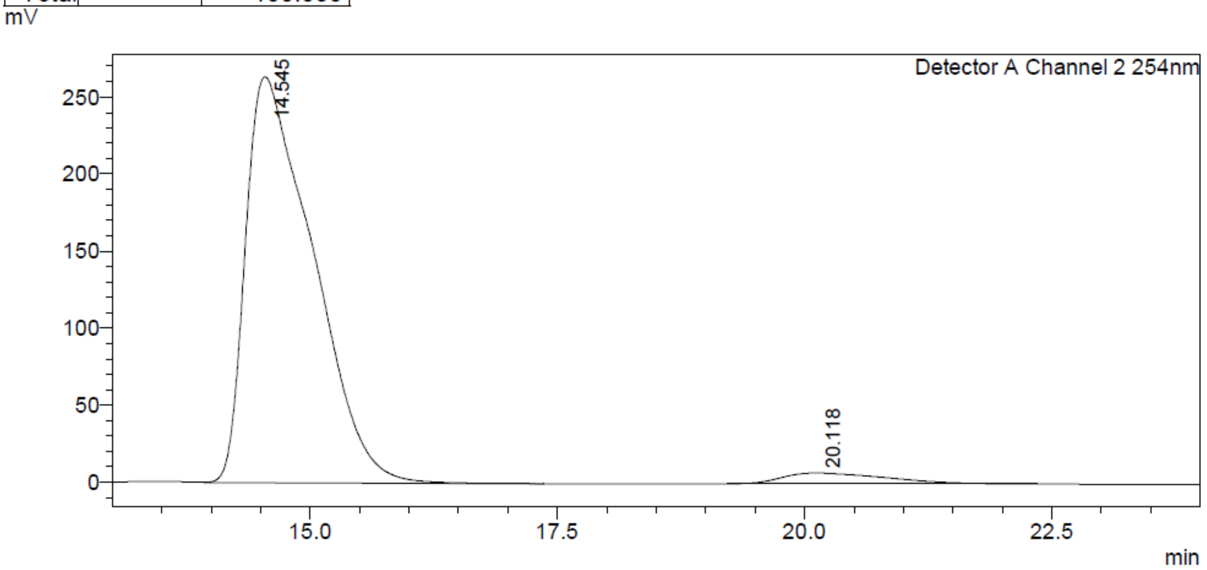

Detector A Channel 2 254nm

Peak#	Ret. Time	Area%
1	14.545	96.093
2	20.118	3.907
Total		100.000

(*S*)-4-(4-Bromobenzoyl)-3-(trifluoromethyl)-2,3-dihydro-1*H*-benzo[4,5]thiazolo[3,2-*a*]pyridin-1-one **18**

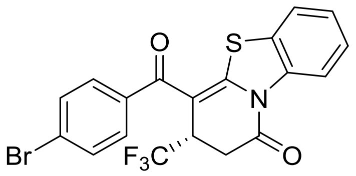

Chiral HPLC analysis: CHIRALPAK AD-H (20:80 *i*-PrOH:hexane, flow rate 1.0 mL min^{-1}, 211 nm, 30 °C), t_R (*S*): 21.0 min, t_R (*R*): 24.5 min, 95.6:4.4 (*S*:*R*) er

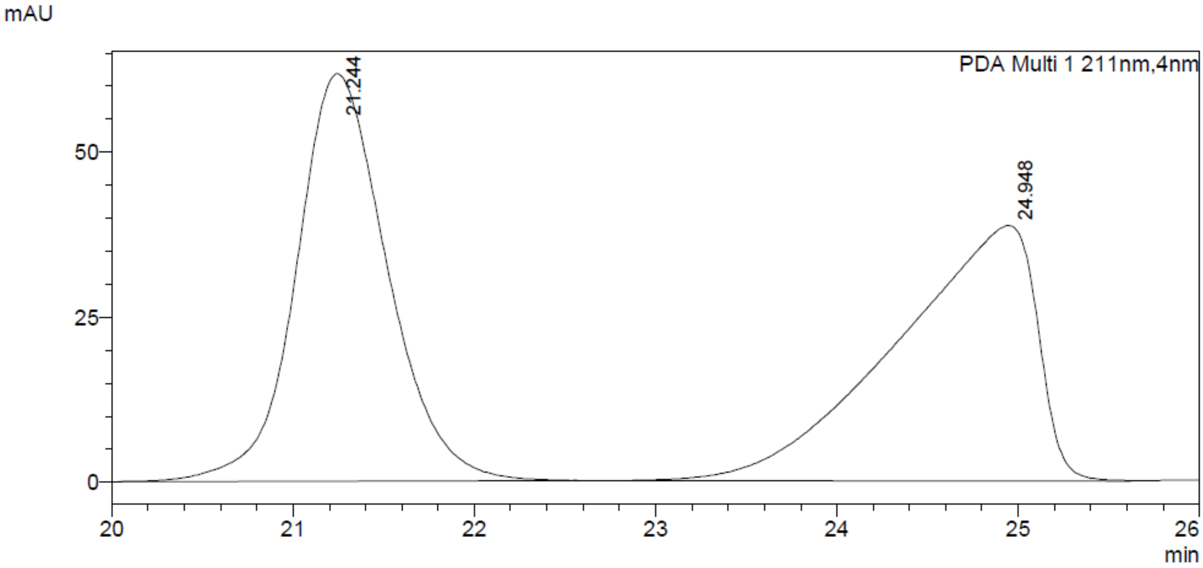

PDA Ch1 211nm

Peak#	Ret. Time	Area%
1	21.244	50.736
2	24.948	49.264
Total		100.000

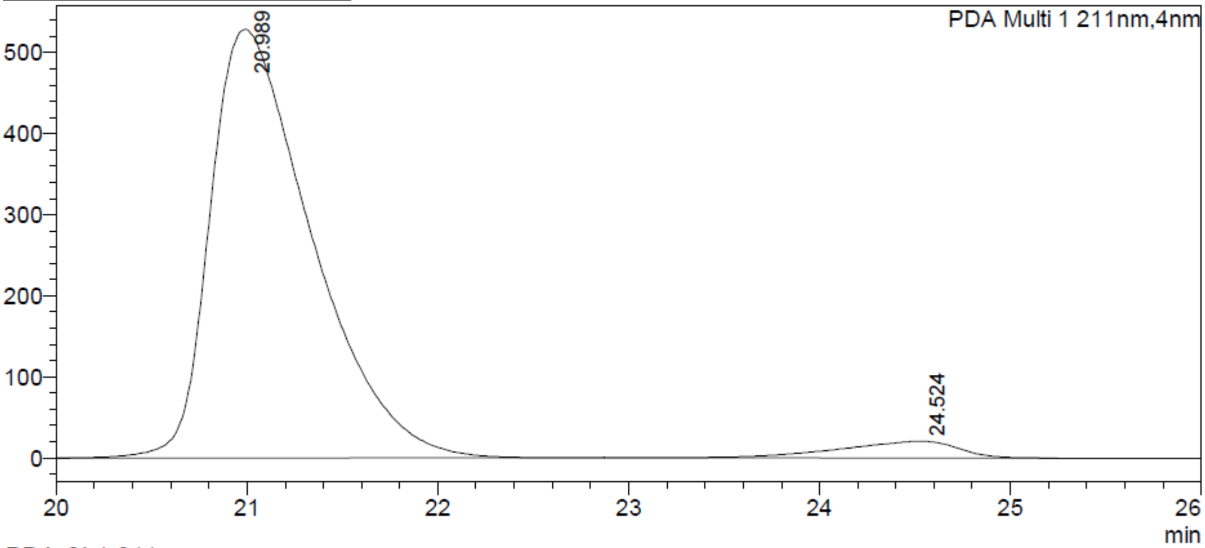

PDA Ch1 211nm

Peak#	Ret. Time	Area%
1	20.989	95.605
2	24.524	4.395
Total		100.000

Crystal recovered following absolute configuration determination by X-ray crystallographic analysis:

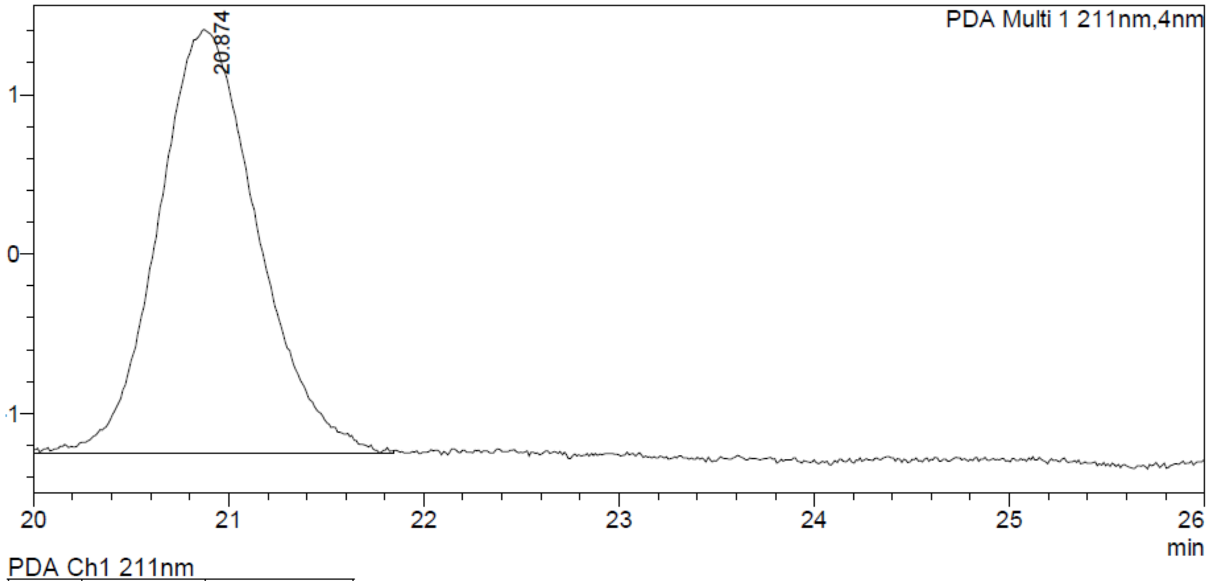

PDA Ch1 211nm

Peak#	Ret. Time	Area%
1	20.874	100.000
Total		100.000

(*S*)-4-(4-Nitrobenzoyl)-3-(trifluoromethyl)-2,3-dihydro-1*H*-benzo[4,5]thiazolo[3,2-*a*]pyridin-1-one **19**

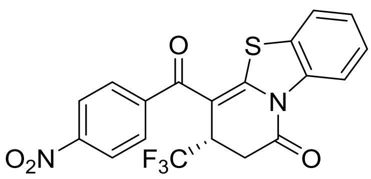

Chiral HPLC analysis: CHIRALPAK AD-H (20:80 *i*-PrOH:hexane, flow rate 1.5 mL min^{-1}, 254 nm, 40 °C), t_R (*S*): 17.6 min, t_R (*R*): 34.7 min, 87.0:13.0 (*S*:*R*) er

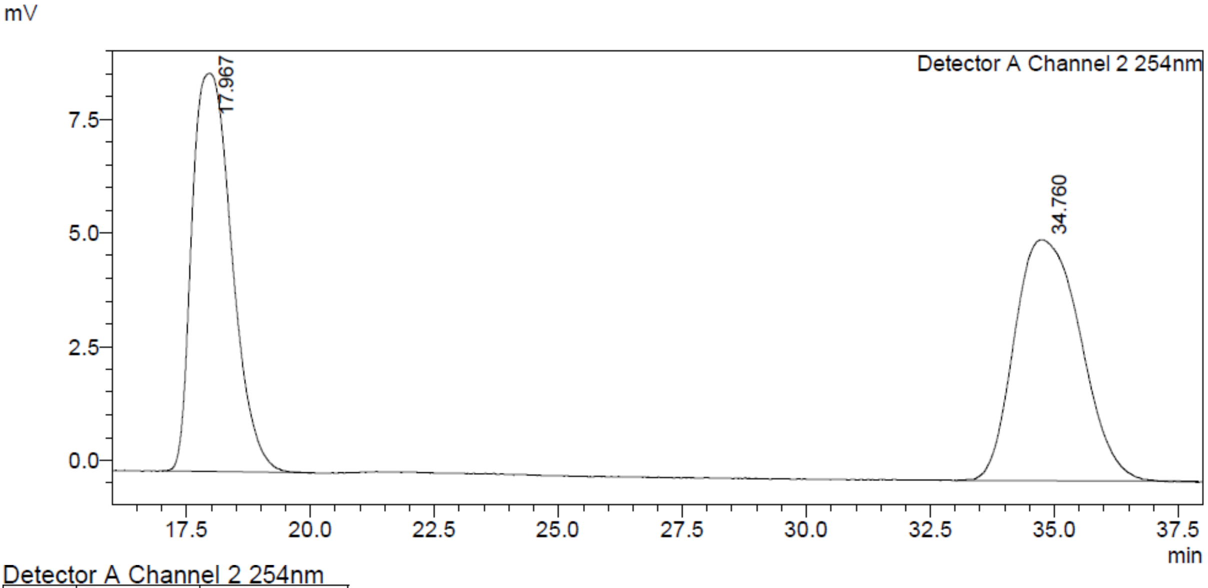

Detector A Channel 2 254nm

Peak#	Ret. Time	Area%
1	17.967	49.887
2	34.760	50.113
Total		100.000

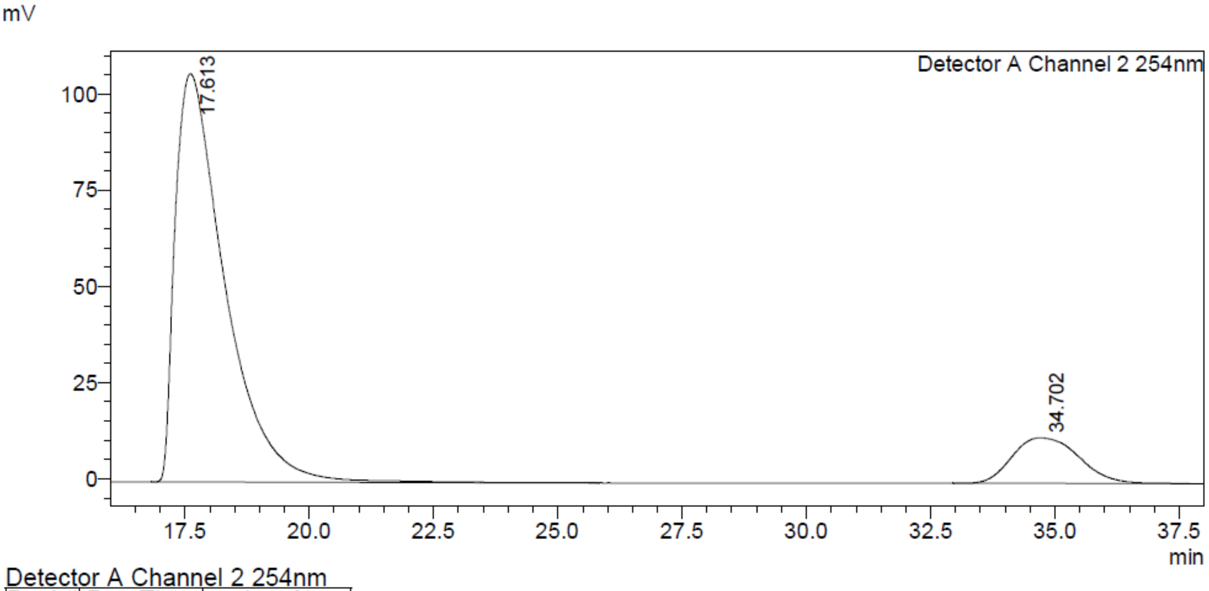

Detector A Channel 2 254nm

Peak#	Ret. Time	Area%
1	17.613	86.963
2	34.702	13.037
Total		100.000

(*S*)-4-(2-Iodobenzoyl)-3-(trifluoromethyl)-2,3-dihydro-1*H*-benzo[4,5]thiazolo[3,2-*a*]pyridin-1-one **20**

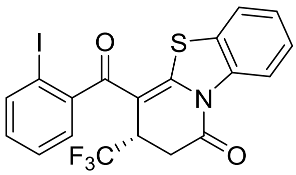

Chiral HPLC analysis: CHIRALPAK AD-H (10:90 *i*-PrOH:hexane, flow rate 1.5 mL min^{-1}, 211 nm, 40 °C), t_R (*R*): 7.2 min, t_R (*S*): 11.8 min, 96.7:3.3 (*S*:*R*) er

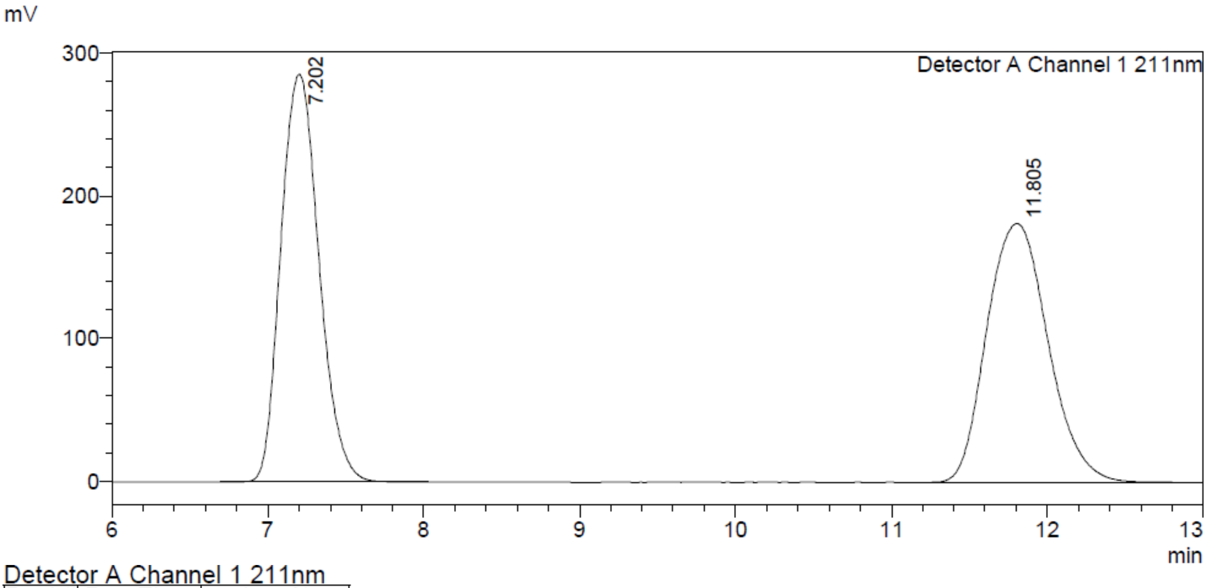

Detector A Channel 1 211nm

Peak#	Ret. Time	Area%
1	7.202	49.600
2	11.805	50.400
Total		100.000

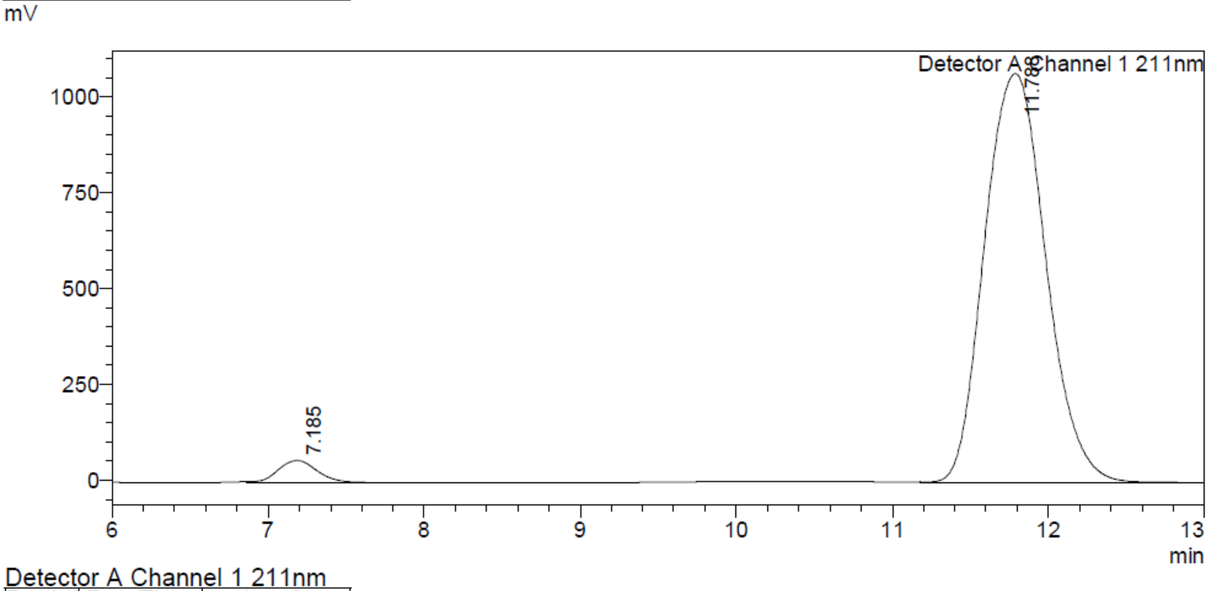

Detector A Channel 1 211nm

Peak#	Ret. Time	Area%
1	7.185	3.288
2	11.788	96.712
Total		100.000

(*S*)-4-(1-Naphthoyl)-3-(trifluoromethyl)-2,3-dihydro-1*H*-benzo[4,5]thiazolo[3,2-*a*]pyridin-1-one **21**

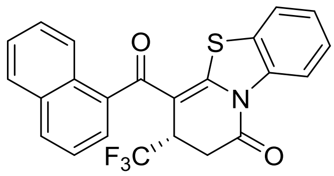

Chiral HPLC analysis: CHIRALPAK AD-H (10:90 *i*-PrOH:hexane, flow rate 1.5 mL min^{-1}, 254 nm, 40 °C), t_R (*R*): 11.4 min, t_R (*S*): 17.6 min, 96.0:4.0 (*S*:*R*) er

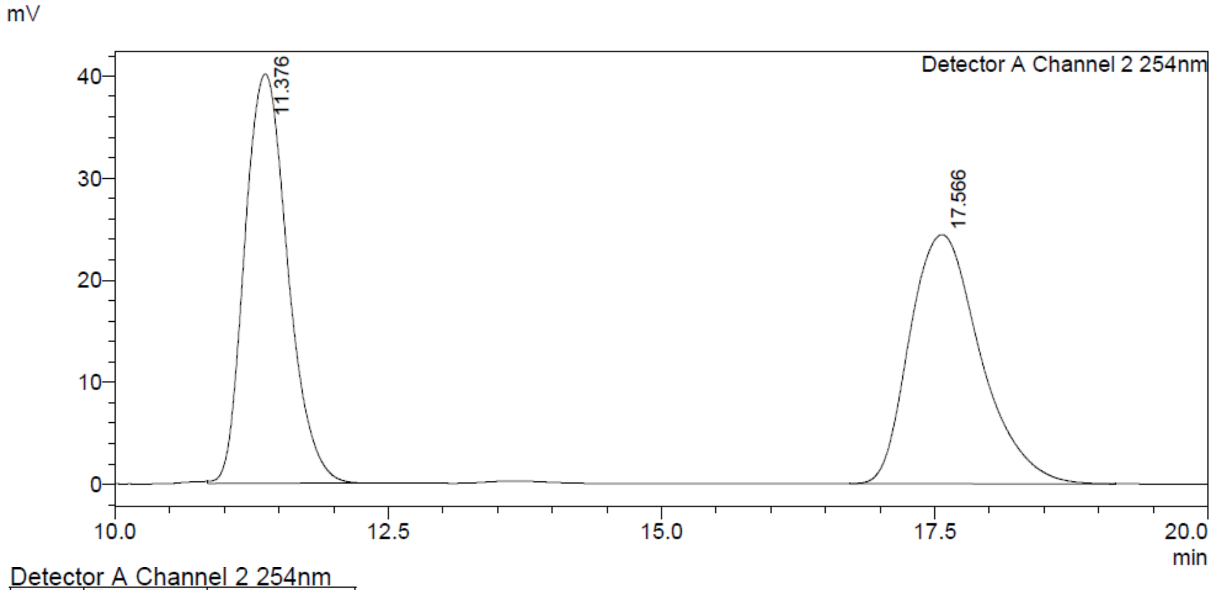

Detector A Channel 2 254nm

Peak#	Ret. Time	Area%
1	11.376	50.074
2	17.566	49.926
Total		100.000

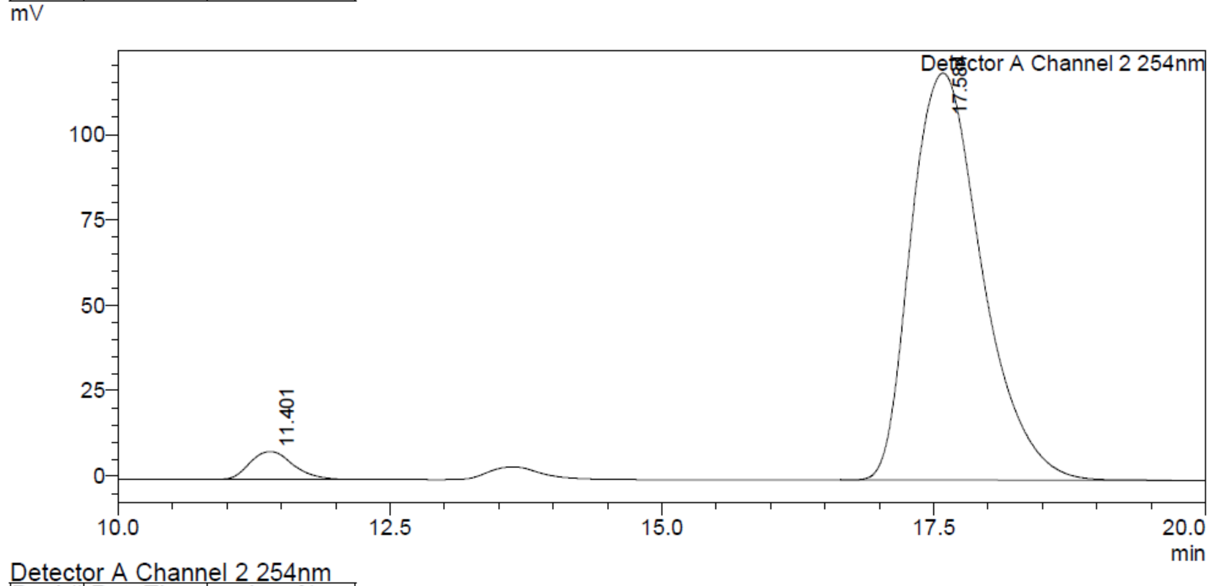

Detector A Channel 2 254nm

Peak#	Ret. Time	Area%
1	11.401	3.980
2	17.584	96.020
Total		100.000

(*S*)-4-Picolinoyl-3-(trifluoromethyl)-2,3-dihydro-1*H*-benzo[4,5]thiazolo[3,2-*a*]pyridin-1-one **22**

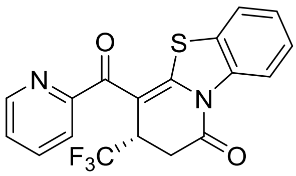

Chiral HPLC analysis: CHIRALPAK AD-H (15:85 *i*-PrOH:hexane, flow rate 1.5 mL min^{-1}, 254 nm, 40 °C), t_R (*R*): 5.4 min, t_R (*S*): 9.6 min, 96.2:3.8 (*S*:*R*) er

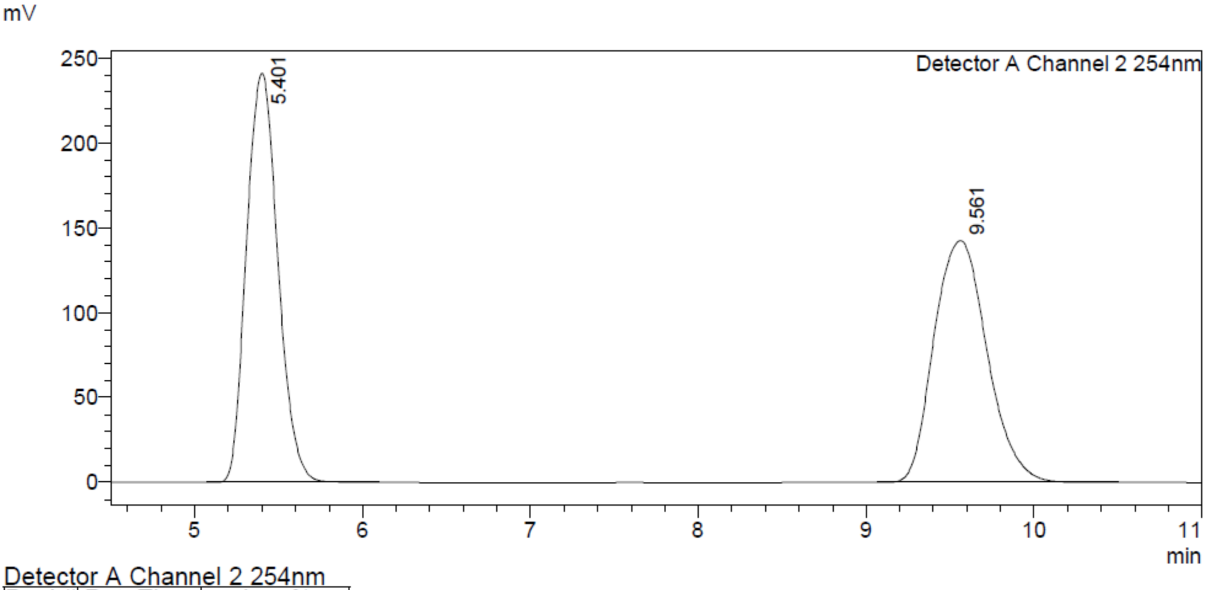

Detector A Channel 2 254nm

Peak#	Ret. Time	Area%
1	5.401	50.003
2	9.561	49.997
Total		100.000

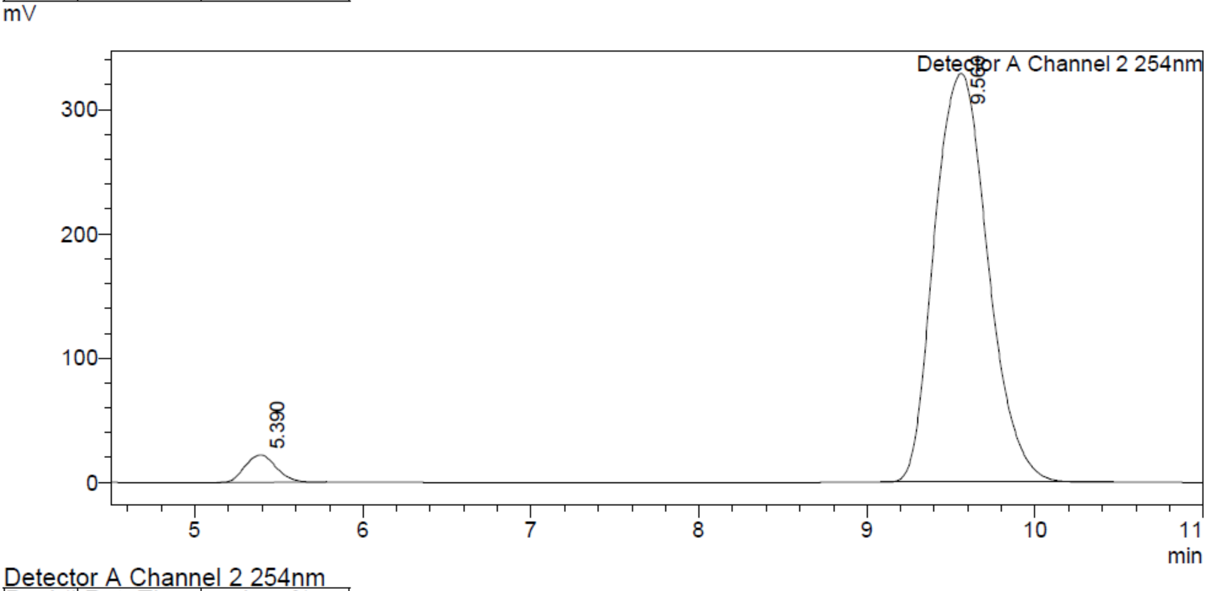

Detector A Channel 2 254nm

Peak#	Ret. Time	Area%
1	5.390	3.751
2	9.560	96.249
Total		100.000

(*S*)-4-(Thiophene-2-carbonyl)-3-(trifluoromethyl)-2,3-dihydro-1*H*-benzo[4,5]thiazolo[3,2-*a*]pyridin-1-one **23**

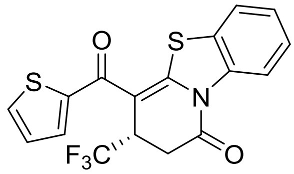

Chiral HPLC analysis: CHIRALPAK AD-H (20:80 *i*-PrOH:hexane, flow rate 1.5 mL min^{-1}, 254 nm, 40 °C), t_R (*R*): 6.0 min, t_R (*S*): 8.4 min, 97.0:3.0 (*S*:*R*) er

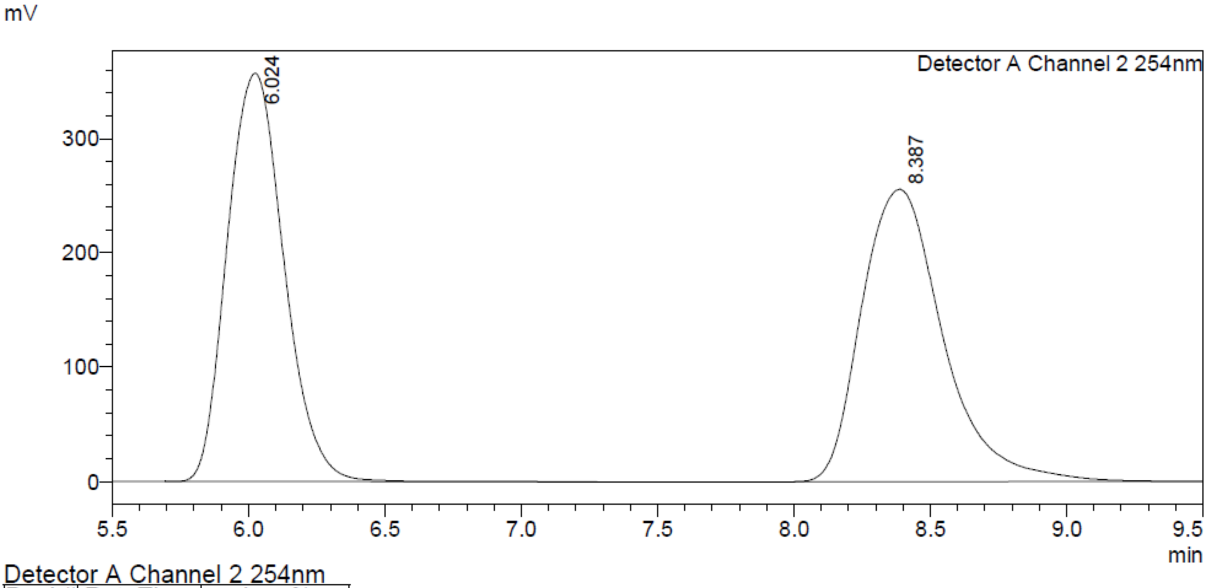

Detector A Channel 2 254nm

Peak#	Ret. Time	Area%
1	6.024	49.275
2	8.387	50.725
Total		100.000

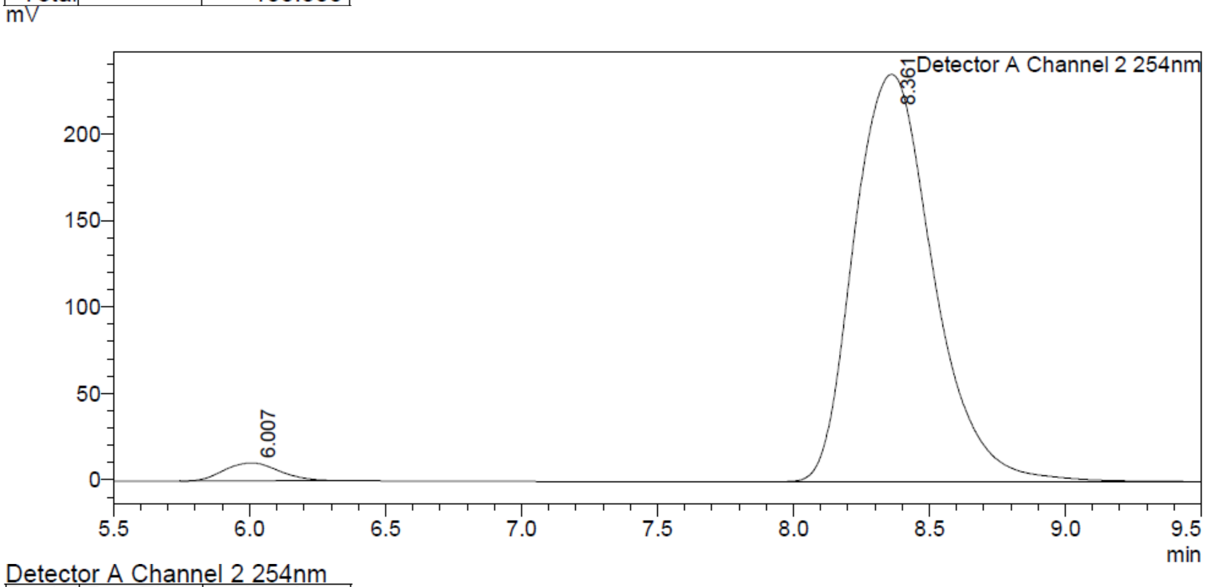

Detector A Channel 2 254nm

Peak#	Ret. Time	Area%
1	6.007	2.987
2	8.361	97.013
Total		100.000

(*S*)-4-Acetyl-3-(trifluoromethyl)-2,3-dihydro-1*H*-benzo[4,5]thiazolo[3,2-*a*]pyridin-1-one **24**

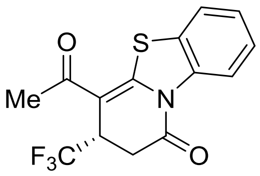

Chiral HPLC analysis: CHIRALCEL OJ-H (20:80 *i*-PrOH:hexane, flow rate 1.5 mL min^{-1}, 254 nm, 40 °C), t_R (*R*): 9.2 min, t_R (*S*): 11.0 min, 94.2:5.8 (*S*:*R*) er

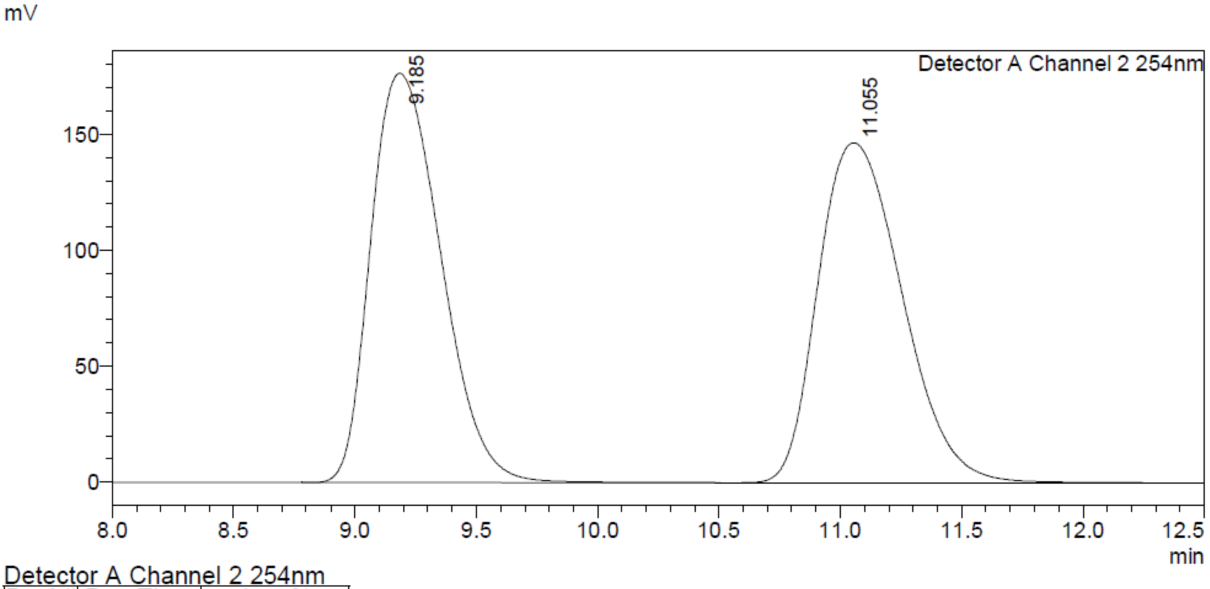

Detector A Channel 2 254nm

Peak#	Ret. Time	Area%
1	9.185	50.133
2	11.055	49.867
Total		100.000

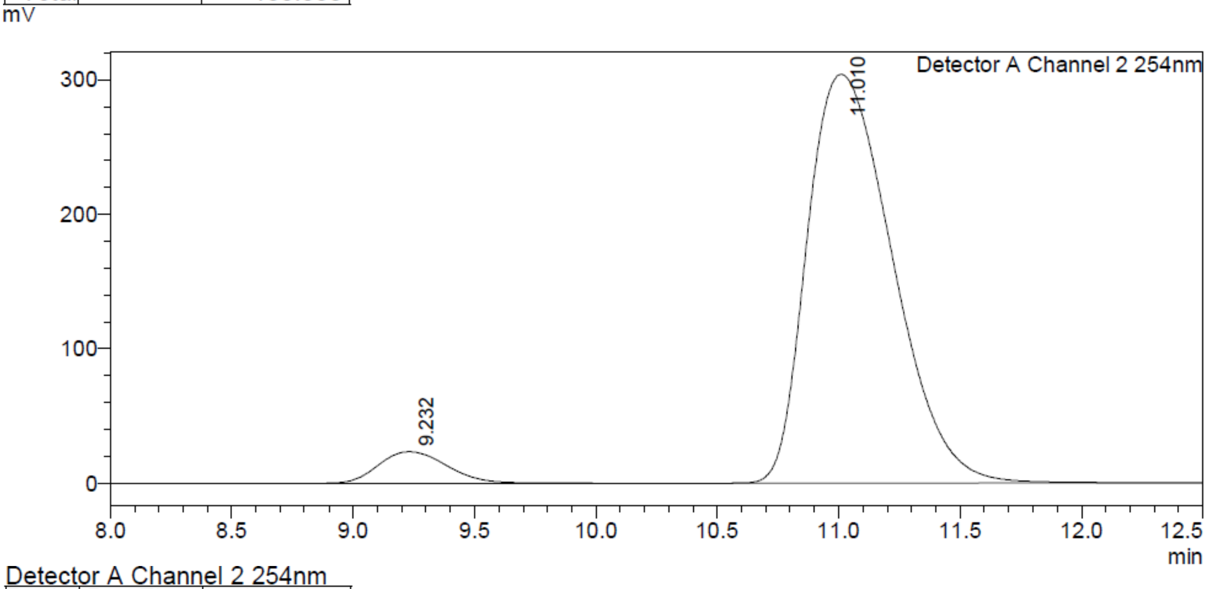

Detector A Channel 2 254nm

Peak#	Ret. Time	Area%
1	9.232	5.798
2	11.010	94.202
Total		100.000

(*S*)-4-Benzoyl-7-fluoro-3-(trifluoromethyl)-2,3-dihydro-1*H*-benzo[4,5]thiazolo[3,2-*a*]pyridin-1-one **26**

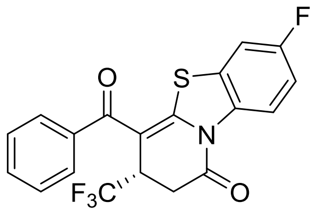

Chiral HPLC analysis: CHIRALCEL OJ-H (20:80 *i*-PrOH:hexane, flow rate 1.5 mL min^{-1}, 254 nm, 40 °C), t_R (*S*): 24.6 min, t_R (*R*): 28.7 min, 97.0:3.0 (*S*:*R*) er

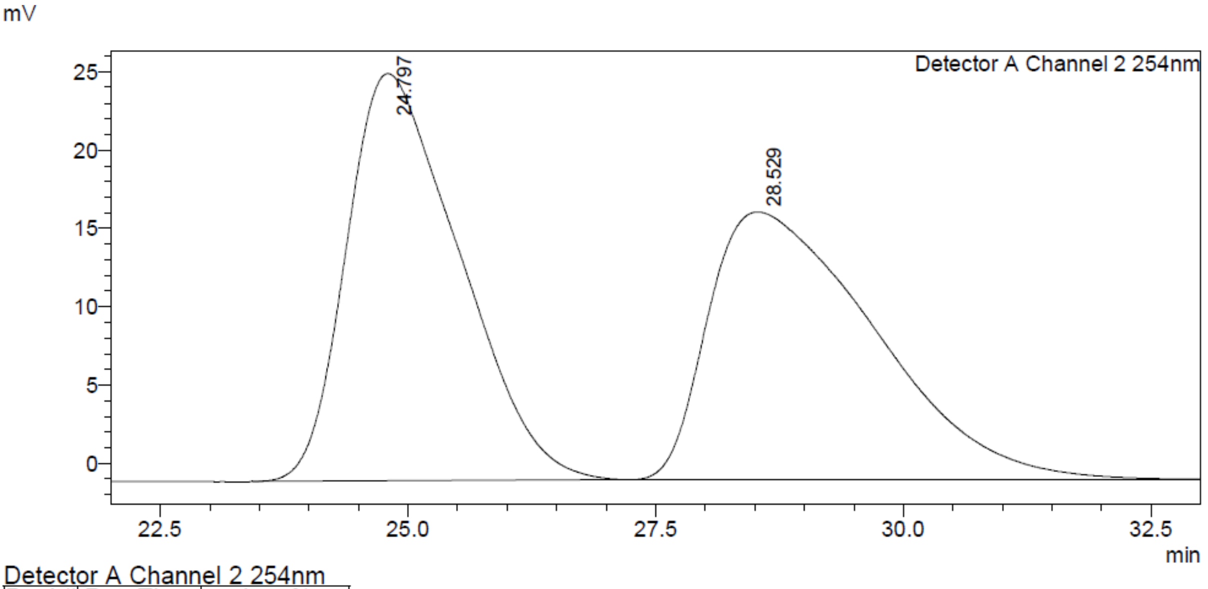

Detector A Channel 2 254nm

Peak#	Ret. Time	Area%
1	24.797	50.555
2	28.529	49.445
Total		100.000

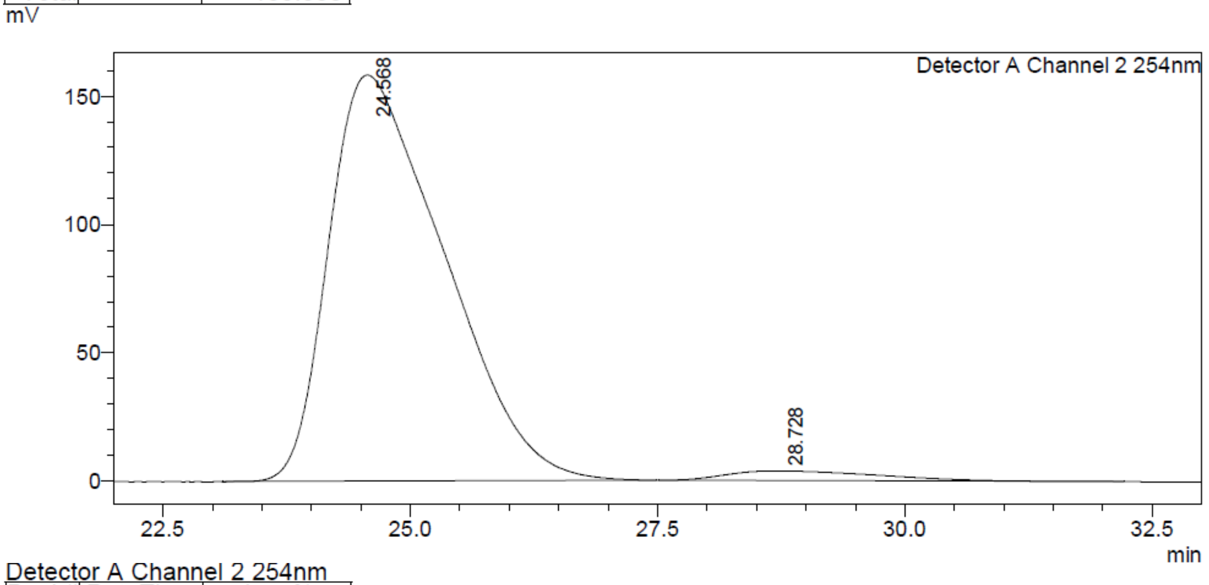

Detector A Channel 2 254nm

Peak#	Ret. Time	Area%
1	24.568	96.952
2	28.728	3.048
Total		100.000

(*S*)-4-Benzoyl-7-bromo-3-(trifluoromethyl)-2,3-dihydro-1*H*-benzo[4,5]thiazolo[3,2-*a*]pyridin-1-one **26**

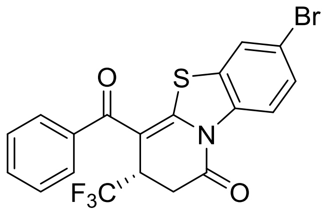

Chiral HPLC analysis: CHIRALCEL OJ-H (20:80 *i*-PrOH:hexane, flow rate 1.5 mL min^{-1}, 254 nm, 40 °C), t_R (*S*): 27.1 min, t_R (*R*): 36.2 min, 96.5:3.5 (*S*:*R*) er

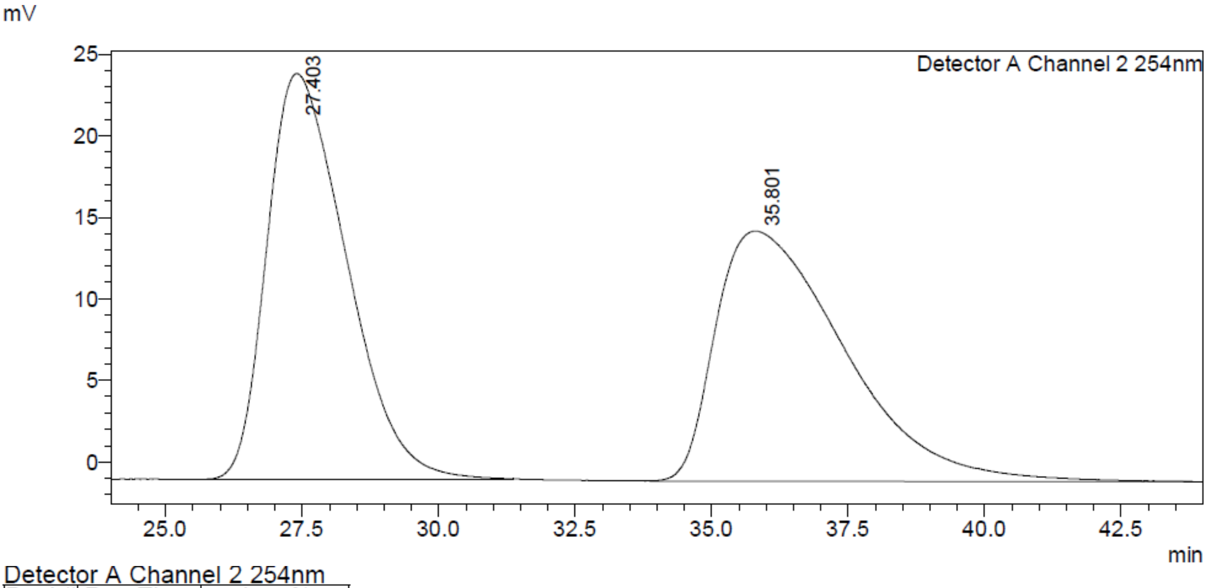

Detector A Channel 2 254nm

Peak#	Ret. Time	Area%
1	27.403	49.958
2	35.801	50.042
Total		100.000

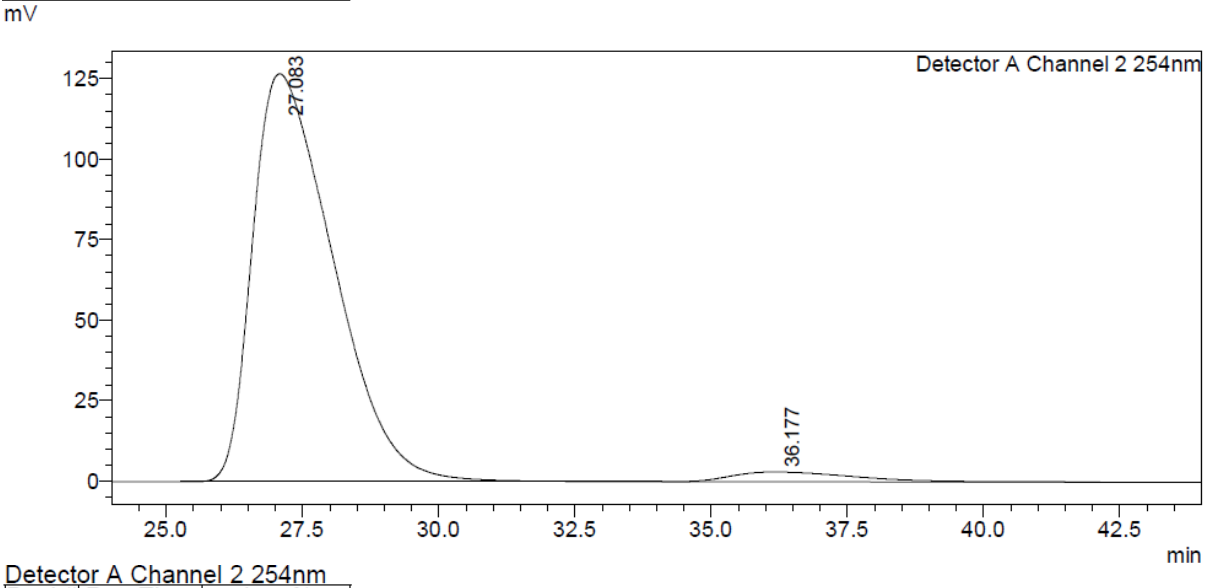

Detector A Channel 2 254nm

Peak#	Ret. Time	Area%
1	27.083	96.492
2	36.177	3.508
Total		100.000

(*S*)-4-Benzoyl-7-methoxy-3-(trifluoromethyl)-2,3-dihydro-1*H*-benzo[4,5]thiazolo[3,2-*a*]pyridin-1-one **27**

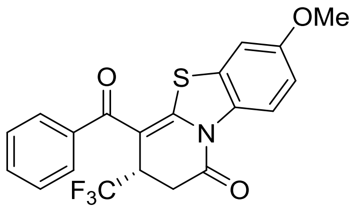

Chiral HPLC analysis: CHIRALCEL OJ-H (20:80 *i*-PrOH:hexane, flow rate 1.5 mL min^{-1}, 254 nm, 40 °C), t_R (*S*): 30.1 min, t_R (*R*): 44.7 min, 97.5:2.5 (*S*:*R*) er

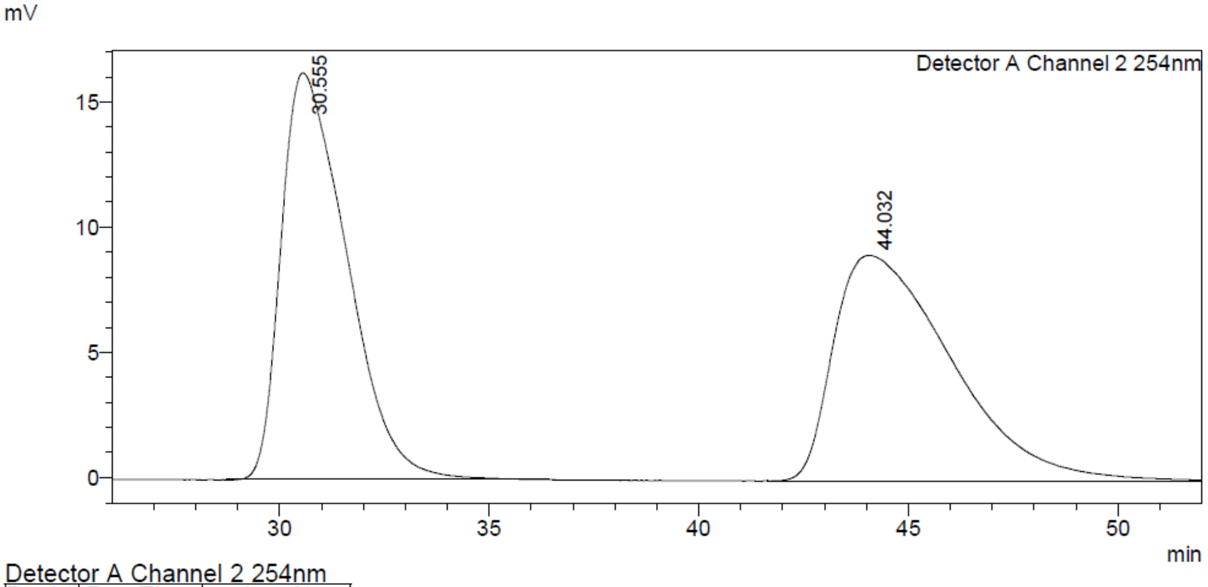

Detector A Channel 2 254nm

Peak#	Ret. Time	Area%
1	30.555	50.048
2	44.032	49.952
Total		100.000

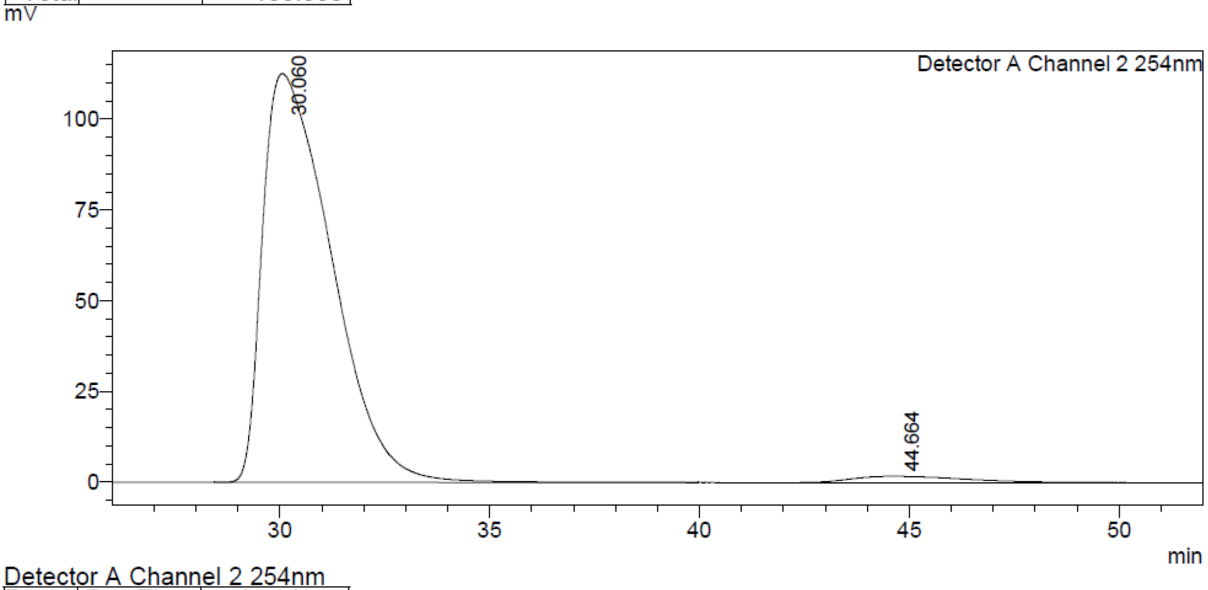

Detector A Channel 2 254nm

Peak#	Ret. Time	Area%
1	30.060	97.519
2	44.664	2.481
Total		100.000

(*S*)-8-Benzoyl-7-(trifluoromethyl)-6,7-dihydro-5*H*-thiazolo[3,2-*a*]pyridin-5-one **28**

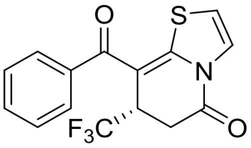

Chiral HPLC analysis: CHIRALCEL OJ-H (20:80 *i*-PrOH:hexane, flow rate 1.5 mL min^{-1}, 254 nm, 40 °C), t_R (*S*): 18.3 min, t_R (*R*): 25.3 min, 96.3:3.7 (*S*:*R*) er

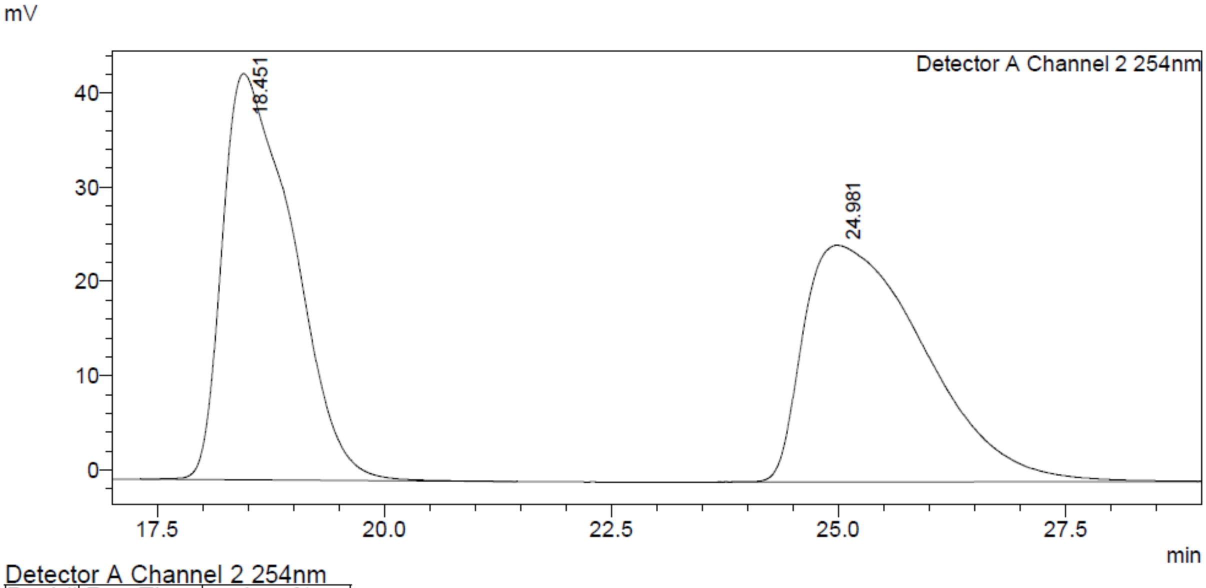

Detector A Channel 2 254nm

Peak#	Ret. Time	Area%
1	18.451	50.010
2	24.981	49.990
Total		100.000

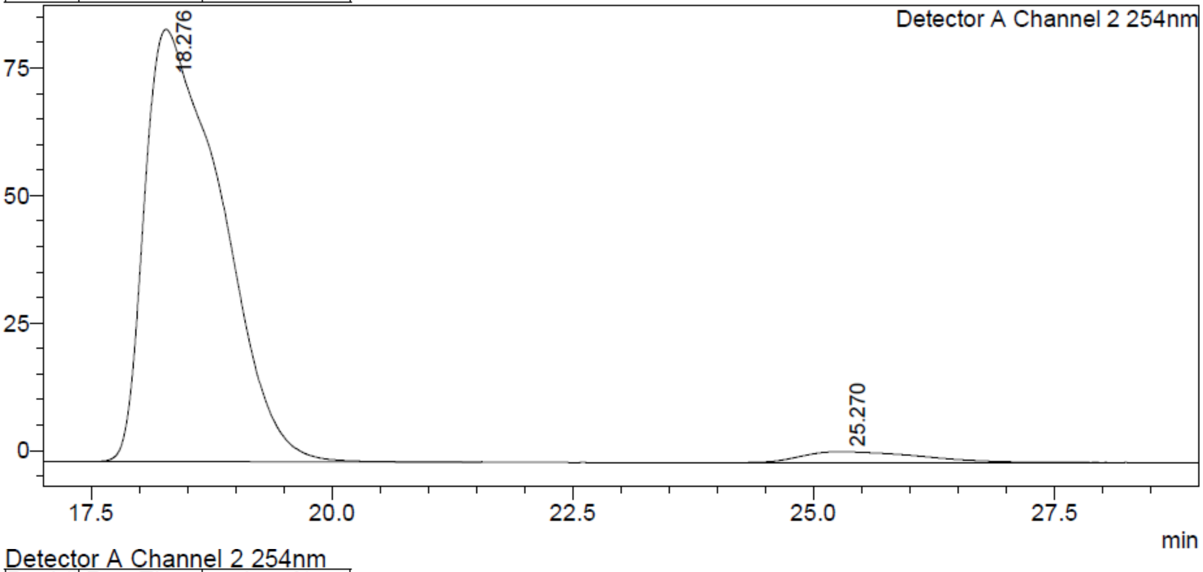

Detector A Channel 2 254nm

Peak#	Ret. Time	Area%
1	18.276	96.346
2	25.270	3.654
Total		100.000

(*S*)-4-Benzoyl-3-(difluoromethyl)-2,3-dihydro-1*H*-benzo[4,5]thiazolo[3,2-*a*]pyridin-1-one **29**

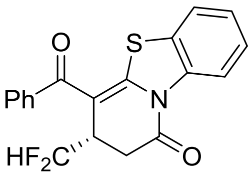

Chiral HPLC analysis: CHIRALPAK AD-H (10:90 *i*-PrOH:hexane, flow rate 1.5 mL min^{-1}, 254 nm, 40 °C), t_R (*R*): 22.1 min, t_R (*S*): 38.2 min, 89.9:10.1 (*S*:*R*) er

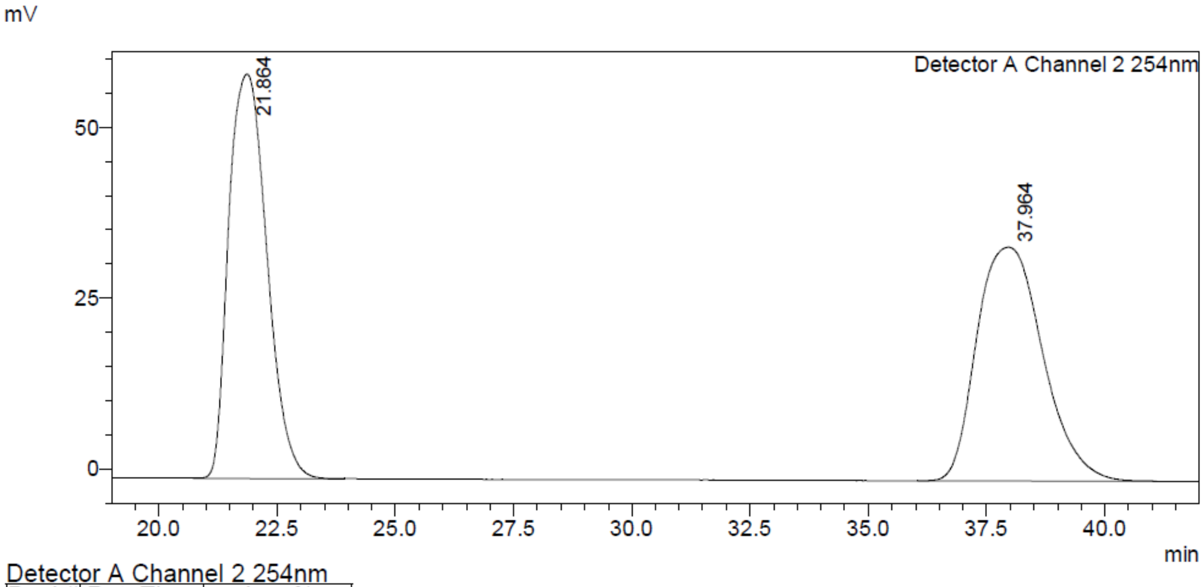

Detector A Channel 2 254nm

Peak#	Ret. Time	Area%
1	21.864	50.051
2	37.964	49.949
Total		100.000

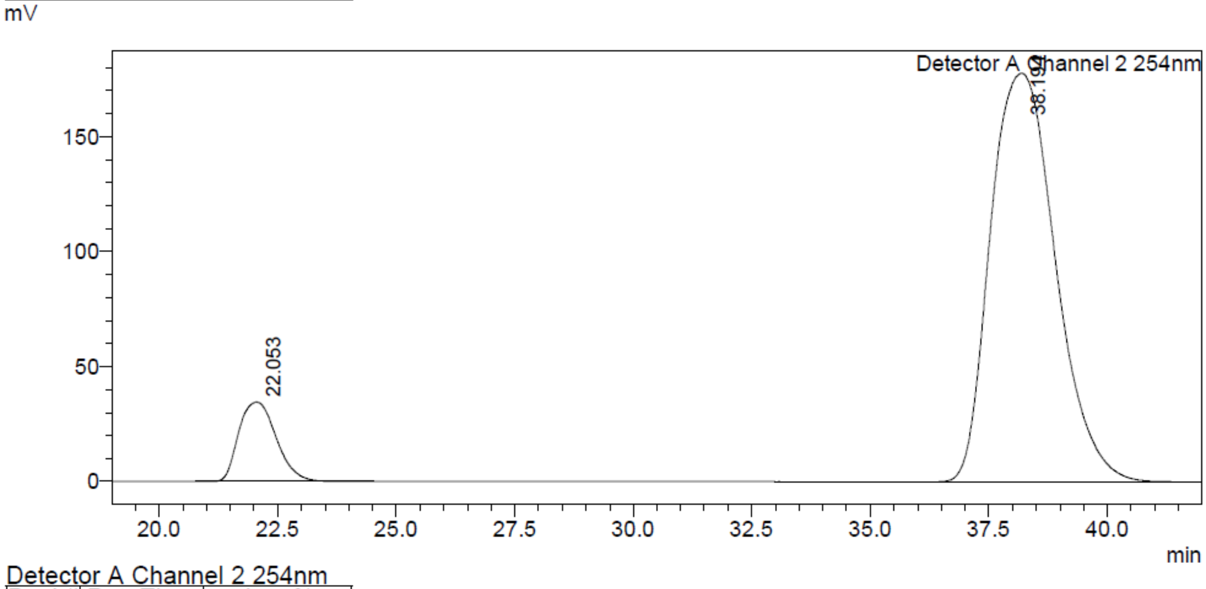

Detector A Channel 2 254nm

Peak#	Ret. Time	Area%
1	22.053	10.089
2	38.194	89.911
Total		100.000

(*S*)-3-(Difluoromethyl)-4-(4-methoxybenzoyl)-2,3-dihydro-1*H*-benzo[4,5]thiazolo[3,2-*a*]pyridin-1-one **30**

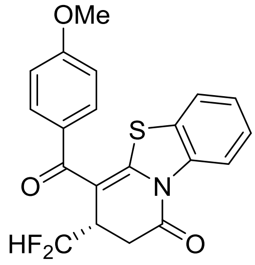

Chiral HPLC analysis: CHIRALPAK IC (20:80 *i*-PrOH:hexane, flow rate 1.5 mL min^{-1}, 254 nm, 40 °C), t_R (*R*): 19.1 min, t_R (*S*): 21.8 min, 91.1:8.9 (*S*:*R*) er

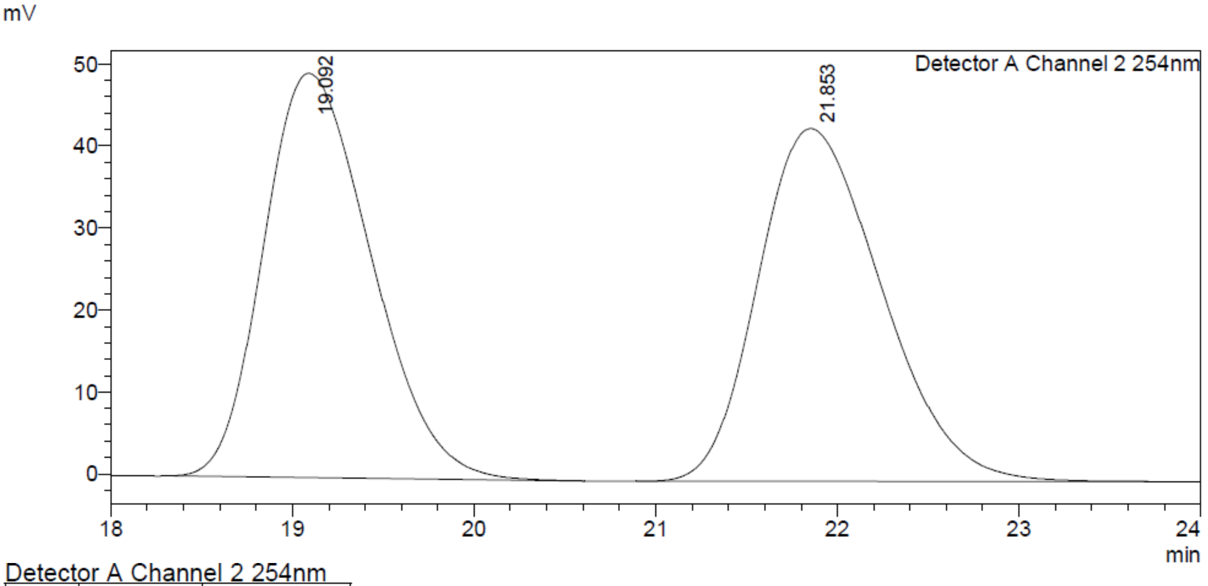

Detector A Channel 2 254nm

Peak#	Ret. Time	Area%
1	19.092	49.960
2	21.853	50.040
Total		100.000

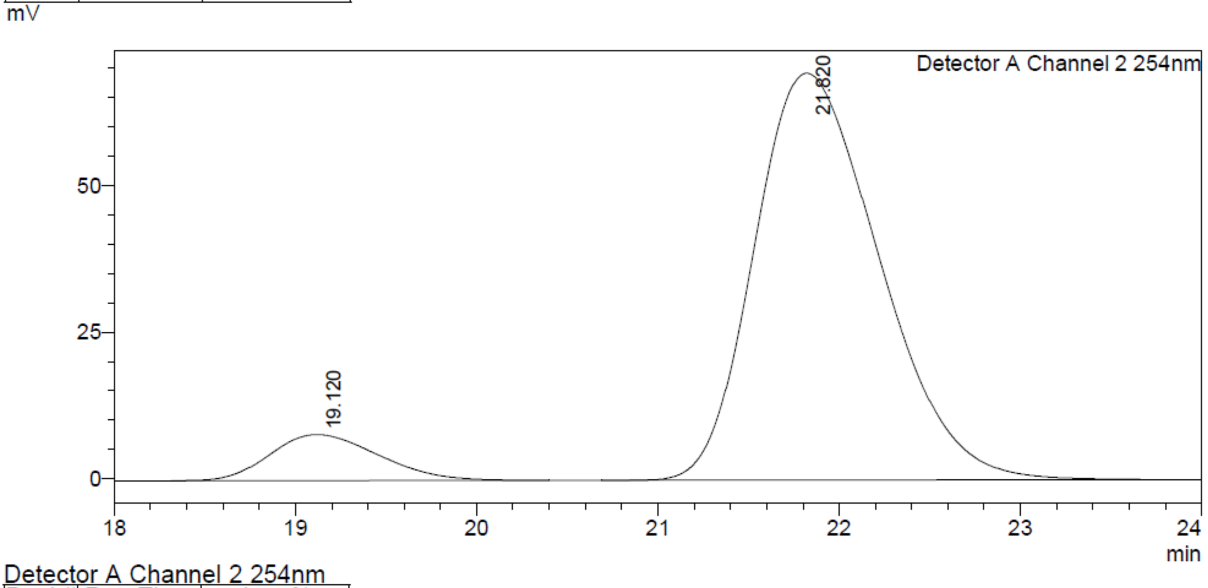

Detector A Channel 2 254nm

Peak#	Ret. Time	Area%
1	19.120	8.943
2	21.820	91.057
Total		100.000

(*S*)-3-(Difluoromethyl)-4-picolinoyl-2,3-dihydro-1*H*-benzo[4,5]thiazolo[3,2-*a*]pyridin-1-one **31**

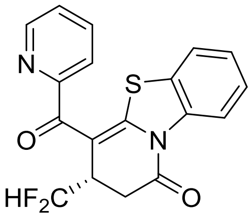

Chiral HPLC analysis: CHIRALPAK AD-H (15:85 *i*-PrOH:hexane, flow rate 1.5 mL min^{-1}, 254 nm, 40 °C), t_R (*R*): 8.8 min, t_R (*S*): 11.0 min, 88.4:11.6 (*S*:*R*) er

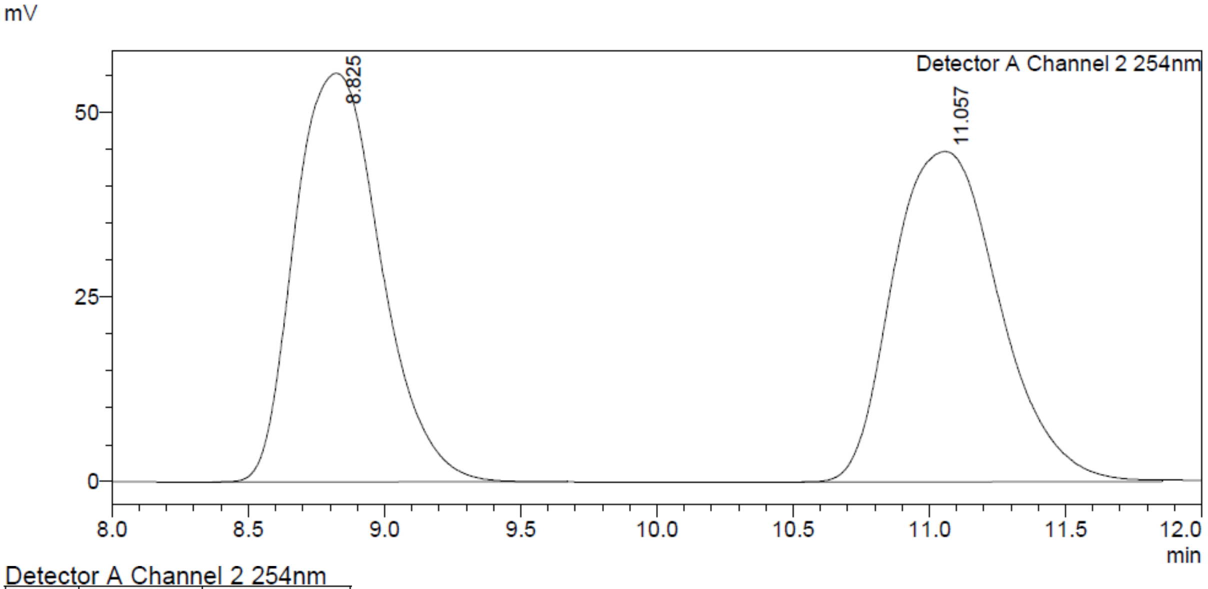

Detector A Channel 2 254nm

Peak#	Ret. Time	Area%
1	8.825	49.913
2	11.057	50.087
Total		100.000

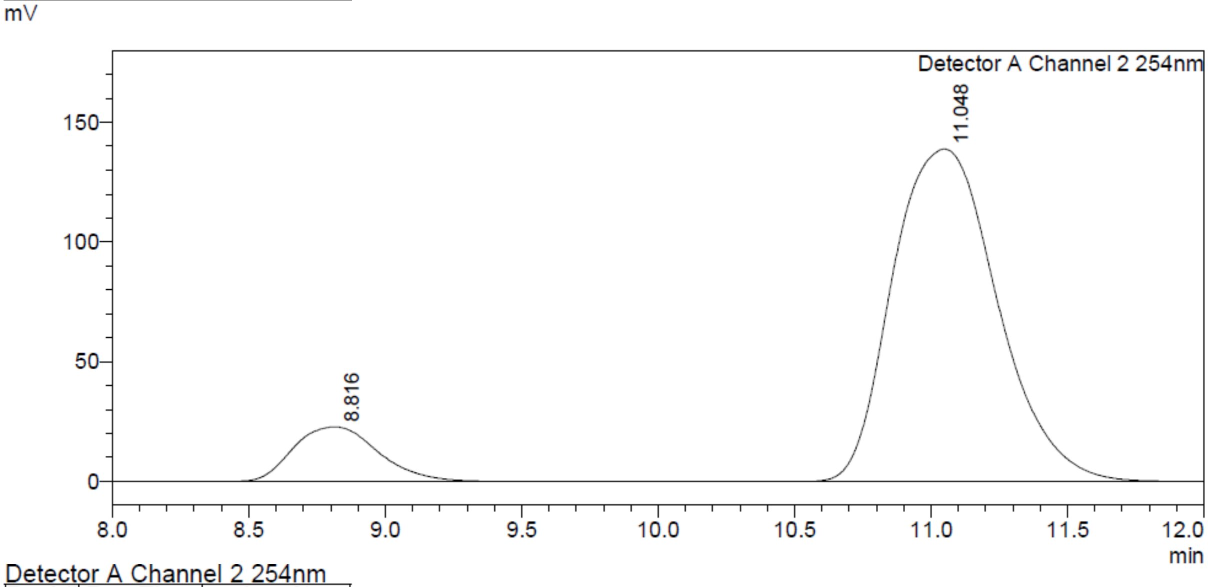

Detector A Channel 2 254nm

Peak#	Ret. Time	Area%
1	8.816	11.633
2	11.048	88.367
Total		100.000

(*S*)-4-Acetyl-3-(difluoromethyl)-2,3-dihydro-1*H*-benzo[4,5]thiazolo[3,2-*a*]pyridin-1-one **32**

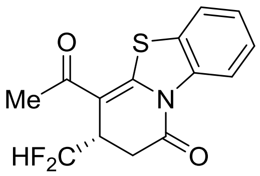

Chiral HPLC analysis: CHIRALCEL OD-H (15:85 *i*-PrOH:hexane, flow rate 1.5 mL min^{-1}, 254 nm, 40 °C), t_R (*R*): 10.9 min, t_R (*S*): 23.1 min, 86.5:13.5 (*S*:*R*) er

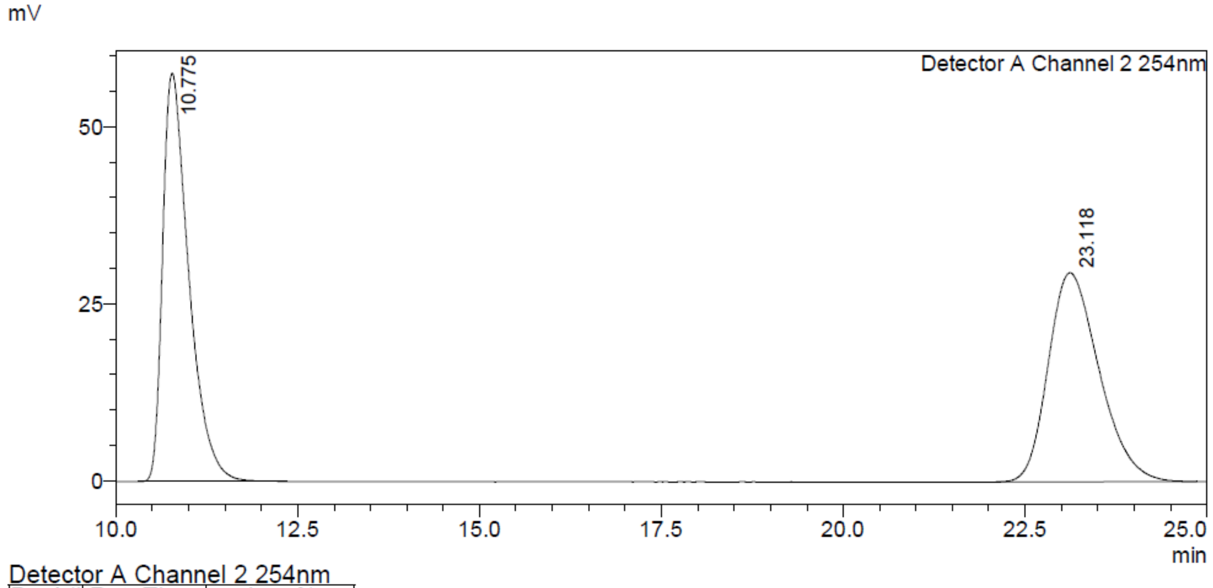

Detector A Channel 2 254nm

Peak#	Ret. Time	Area%
1	10.775	50.099
2	23.118	49.901
Total		100.000

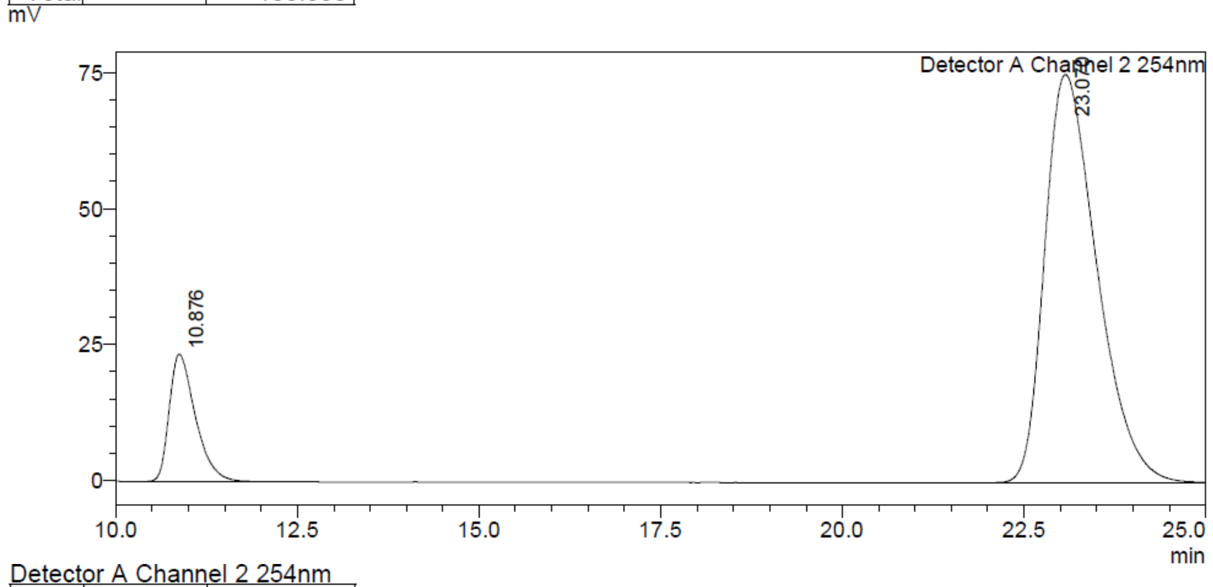

Detector A Channel 2 254nm

Peak#	Ret. Time	Area%
1	10.876	13.503
2	23.070	86.497
Total		100.000

(*S*)-4-Benzoyl-3-(perfluoroethyl)-2,3-dihydro-1*H*-benzo[4,5]thiazolo[3,2-*a*]pyridin-1-one **33**

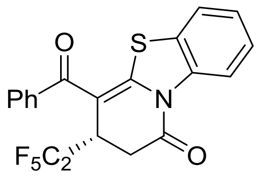

Chiral HPLC analysis: CHIRALPAK AD-H (10:90 *i*-PrOH:hexane, flow rate 1.5 mL min^{-1}, 254 nm, 40 °C), t_R (*R*): 11.0 min, t_R (*S*): 14.3 min, 97.4:2.6 (*S*:*R*) er

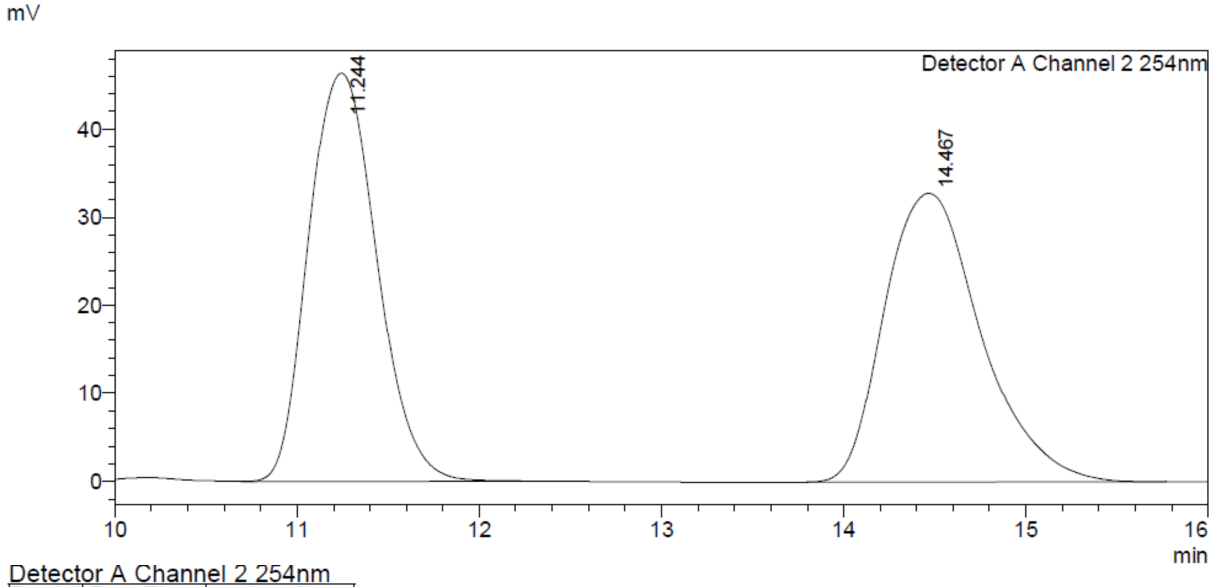

Detector A Channel 2 254nm

Peak#	Ret. Time	Area%
1	11.244	50.046
2	14.467	49.954
Total		100.000

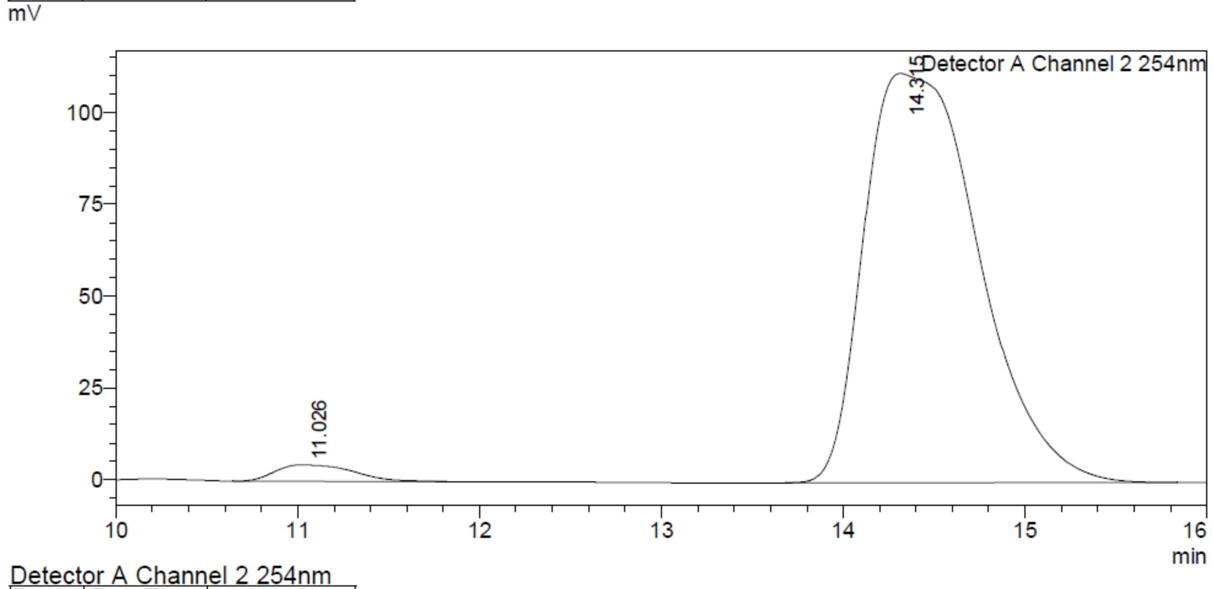

Detector A Channel 2 254nm

Peak#	Ret. Time	Area%
1	11.026	2.649
2	14.315	97.351
Total		100.000

(S)-5-(Benzo[d]oxazol-2-yl)-6-phenyl-4-(trifluoromethyl)-3,4-dihydro-2H-pyran-2-one **34**

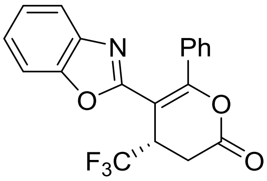

Chiral HPLC analysis: CHIRALPAK AD-H (10:90 *i*-PrOH:hexane, flow rate 1.5 mL min^{-1}, 254 nm, 40 °C), t_R (R): 5.6 min, t_R (S): 14.8 min, 99.5:0.5 (S:R) er

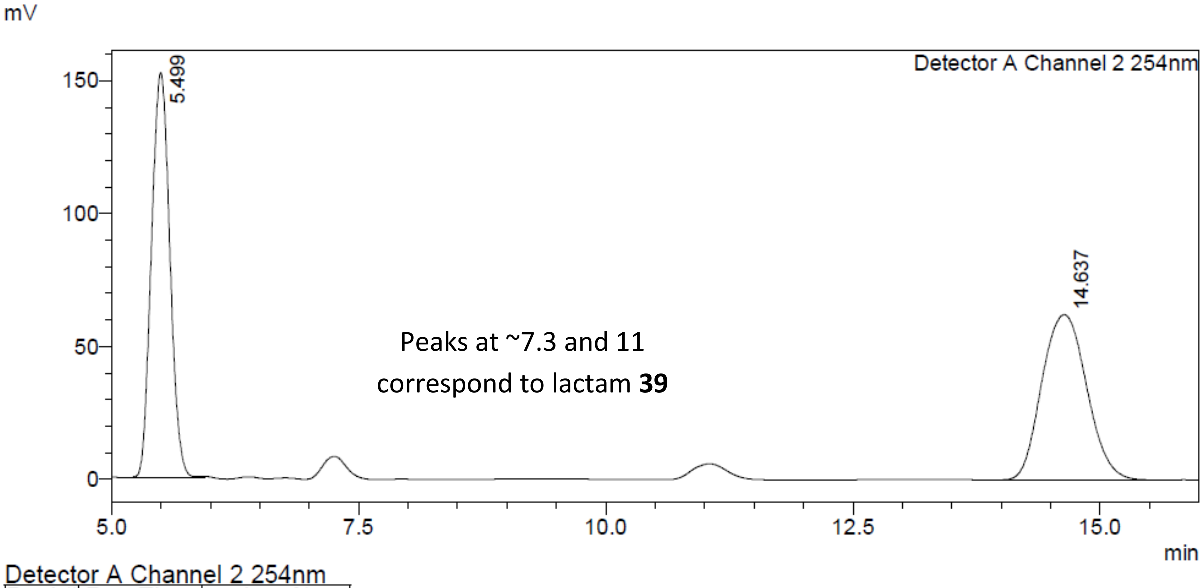

Detector A Channel 2 254nm

Peak#	Ret. Time	Area%
1	5.499	50.122
2	14.637	49.878
Total		100.000

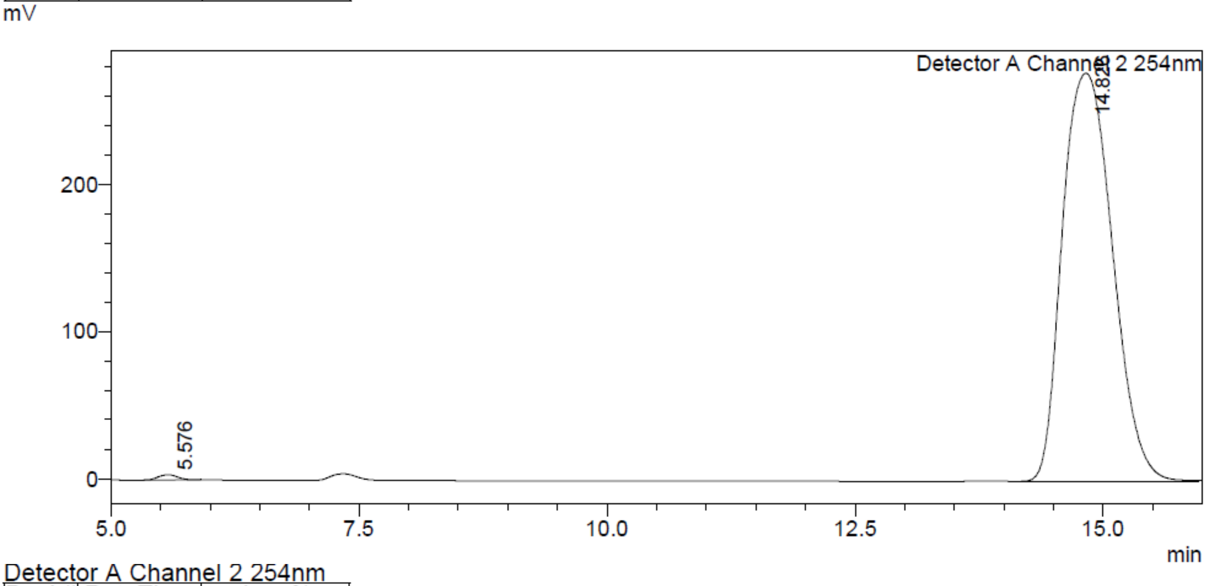

Detector A Channel 2 254nm

Peak#	Ret. Time	Area%
1	5.576	0.491
2	14.826	99.509
Total		100.000

(*S*)-5-(Benzo[*d*]oxazol-2-yl)-6-(pyridin-3-yl)-4-(trifluoromethyl)-3,4-dihydro-2*H*-pyran-2-one **35**

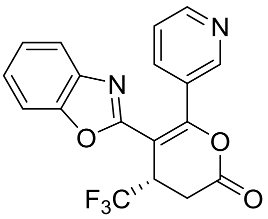

Chiral HPLC analysis: CHIRALPAK AD-H (15:85 *i*-PrOH:hexane, flow rate 1.5 mL min^{-1}, 254 nm, 40 °C), t_R (*R*): 9.0 min, t_R (*S*): 23.3 min, 99.9:0.1 (*S*:*R*) er

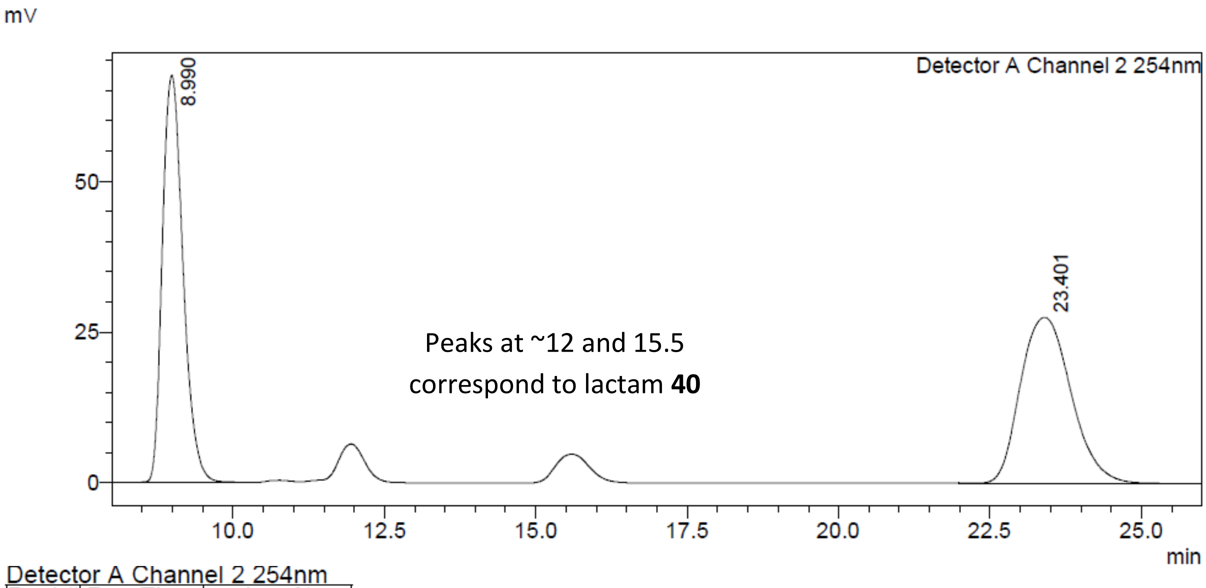

Detector A Channel 2 254nm

Peak#	Ret. Time	Area%
1	8.990	50.105
2	23.401	49.895
Total		100.000

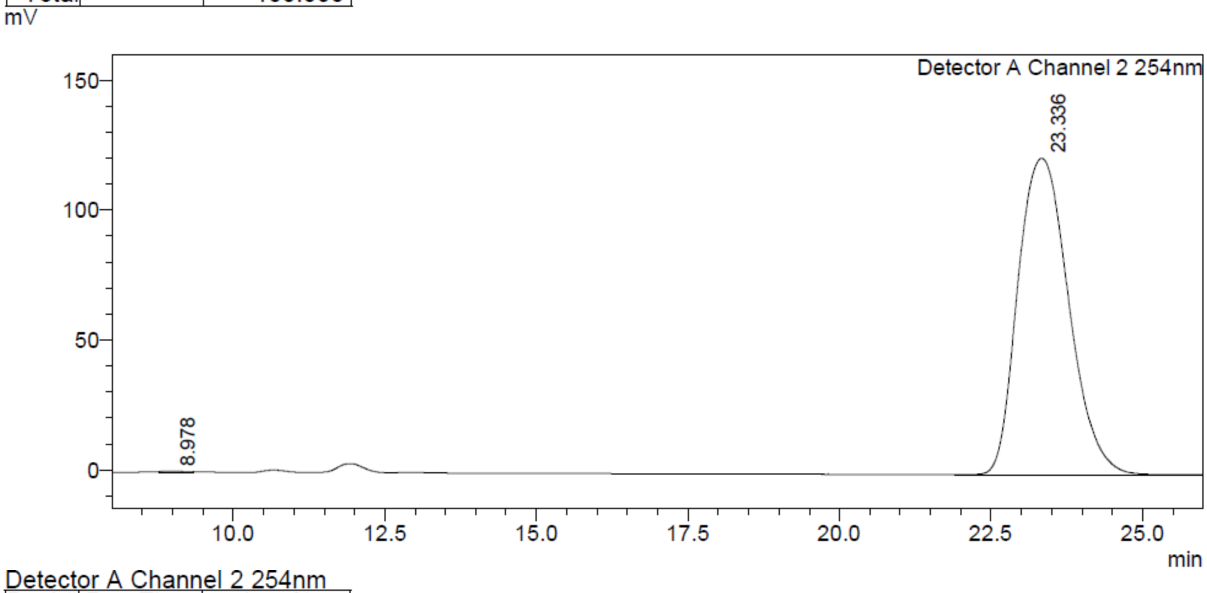

Detector A Channel 2 254nm

Peak#	Ret. Time	Area%
1	8.978	0.144
2	23.336	99.856
Total		100.000

(*S*)-5-(Benzo[*d*]oxazol-2-yl)-6-(thiophen-3-yl)-4-(trifluoromethyl)-3,4-dihydro-2*H*-pyran-2-one **36**

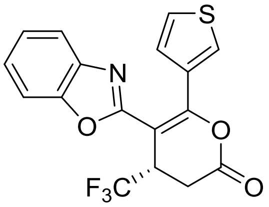

Chiral HPLC analysis: CHIRALPAK AD-H (5:95 *i*-PrOH:hexane, flow rate 1.5 mL min^{-1}, 254 nm, 40 °C), t_R (*R*): 10.0 min, t_R (*S*): 19.2 min, 99.4:0.6 (*S*:*R*) er

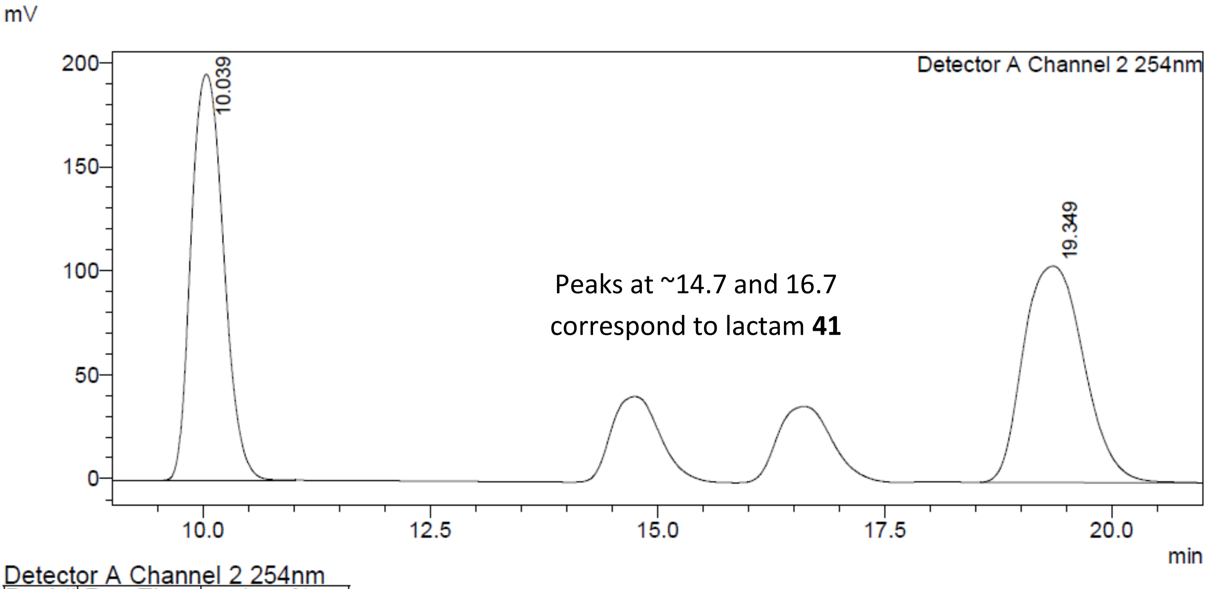

Detector A Channel 2 254nm

Peak#	Ret. Time	Area%
1	10.039	50.206
2	19.349	49.794
Total		100.000

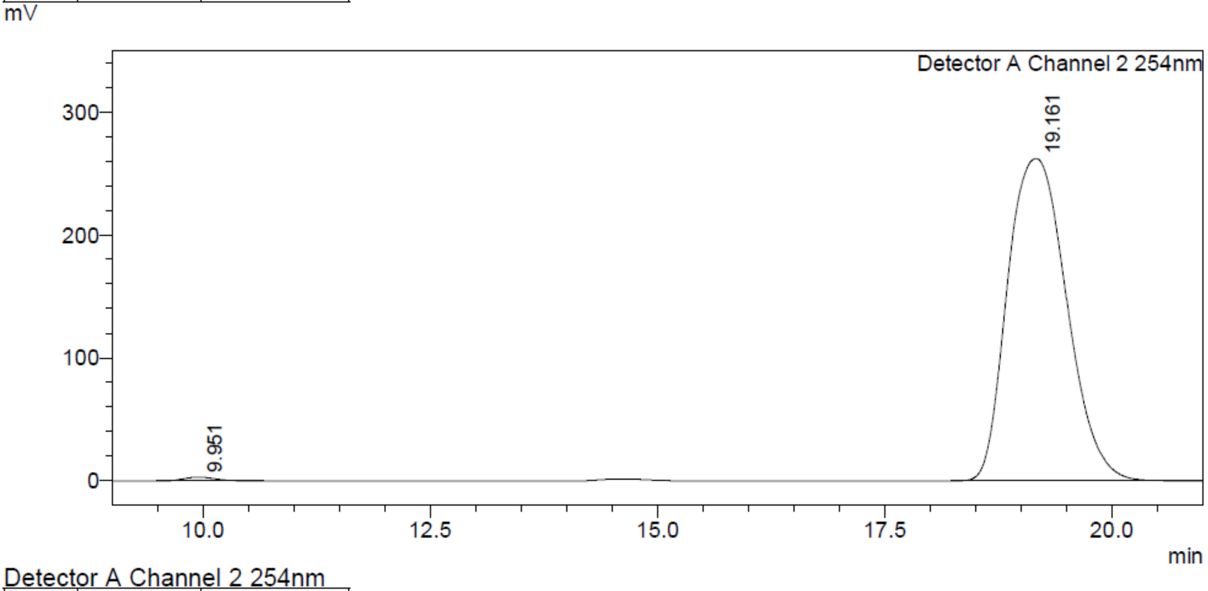

Detector A Channel 2 254nm

Peak#	Ret. Time	Area%
1	9.951	0.606
2	19.161	99.394
Total		100.000

(*S*)-5-(Benzo[*d*]oxazol-2-yl)-4-(trifluoromethyl)-6-(4-(trifluoromethyl)phenyl)-3,4-dihydro-2*H*-pyran-2-one **37**

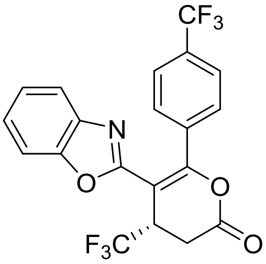

Chiral HPLC analysis: CHIRALPAK AD-H (5:95 *i*-PrOH:hexane, flow rate 1.5 mL min^{-1}, 254 nm, 40 °C), t_R (*R*): 5.7 min, t_R (*S*): 7.3 min, 99.8:0.2 (*S*:*R*) er

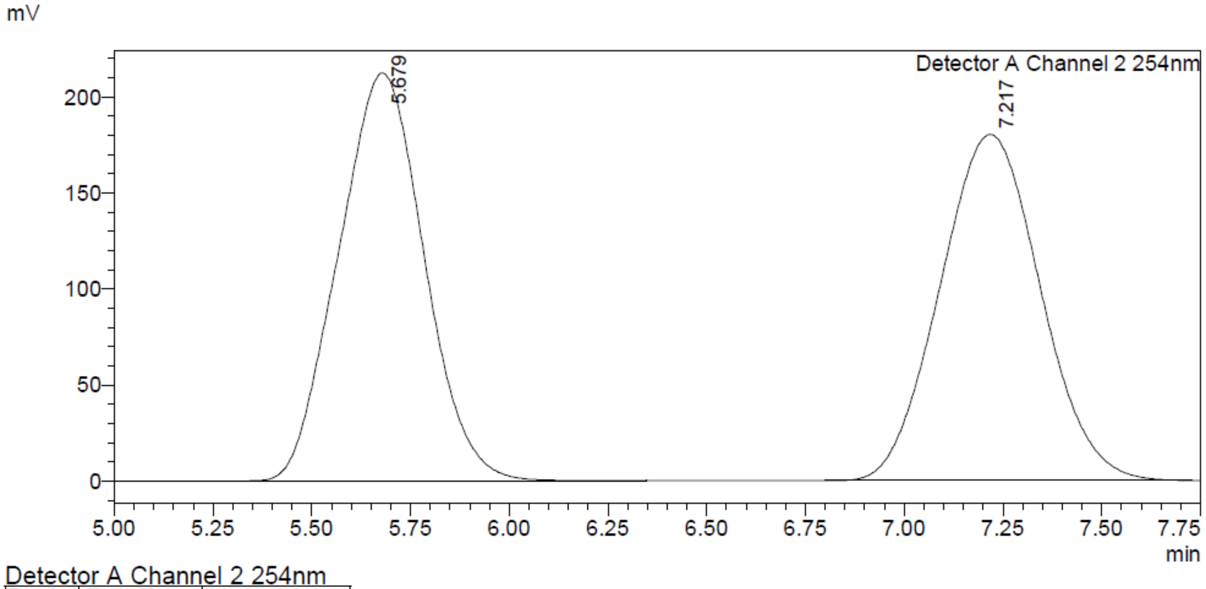

Detector A Channel 2 254nm

Peak#	Ret. Time	Area%
1	5.679	50.151
2	7.217	49.849
Total		100.000

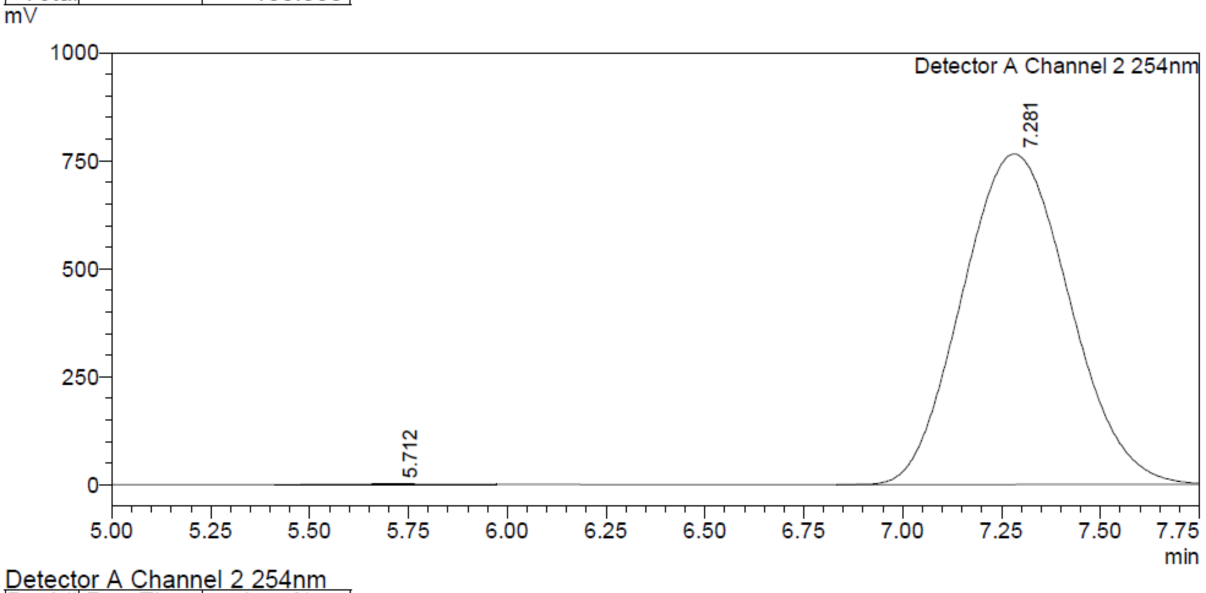

Detector A Channel 2 254nm

Peak#	Ret. Time	Area%
1	5.712	0.197
2	7.281	99.803
Total		100.000

Minor dihydropyridinone product: (*S*)-3-(trifluoromethyl)-4-(4-(trifluoromethyl)benzoyl)-2,3-dihydro-1*H*-benzo[4,5]oxazolo[3,2-*a*]pyridin-1-one **42**

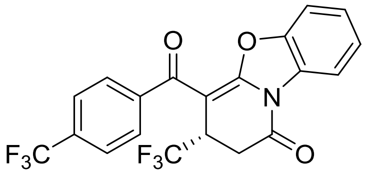

Chiral HPLC analysis: CHIRALPAK AD-H (5:95 *i*-PrOH:hexane, flow rate 1.5 mL min^{-1}, 211 nm, 40 °C), t_R (*S*): 8.2 min, t_R (*R*): 9.6 min, 97.8:2.2 (*S*:*R*) er

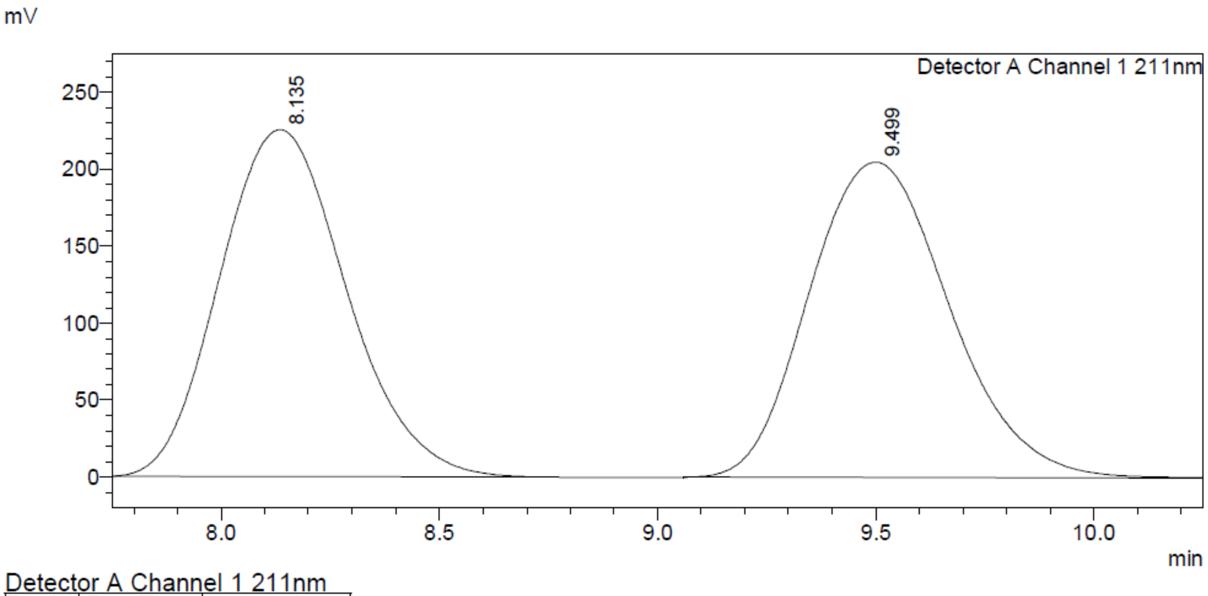

Detector A Channel 1 211nm

Peak#	Ret. Time	Area%
1	8.135	49.860
2	9.499	50.140
Total		100.000

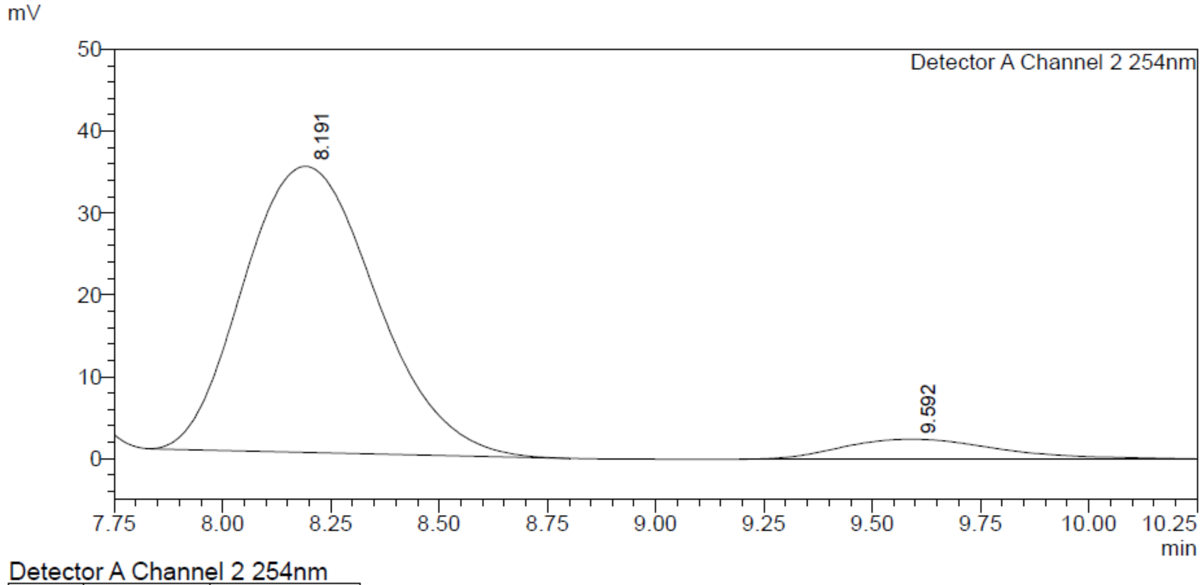

Detector A Channel 2 254nm

Peak#	Ret. Time	Area%
1	8.191	92.250
2	9.592	7.750
Total		100.000

(*S*)-4-(5-(Benzo[*d*]oxazol-2-yl)-2-oxo-4-(trifluoromethyl)-3,4-dihydro-2*H*-pyran-6-yl)benzonitrile **38**

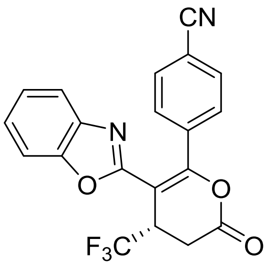

Chiral HPLC analysis: CHIRALPAK IC (20:80 *i*-PrOH:hexane, flow rate 1.5 mL min^{-1}, 254 nm, 40 °C), t_R (*S*): 5.4 min, t_R (*R*): 6.3 min, 99.8:0.2 (*S*:*R*) er

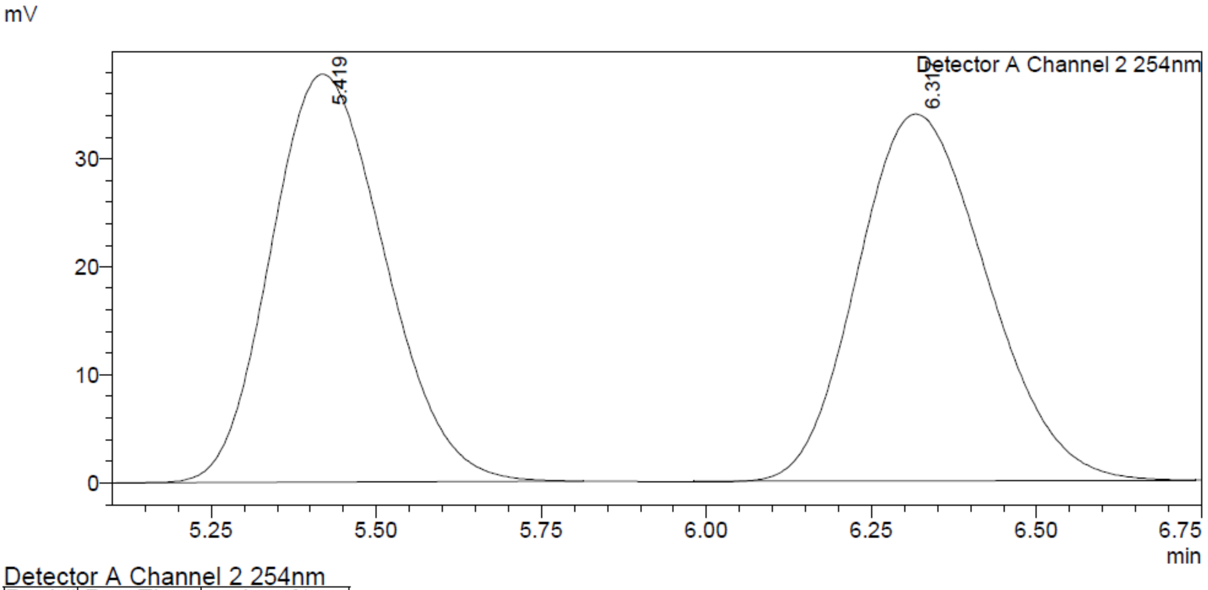

Detector A Channel 2 254nm

Peak#	Ret. Time	Area%
1	5.419	49.507
2	6.317	50.493
Total		100.000

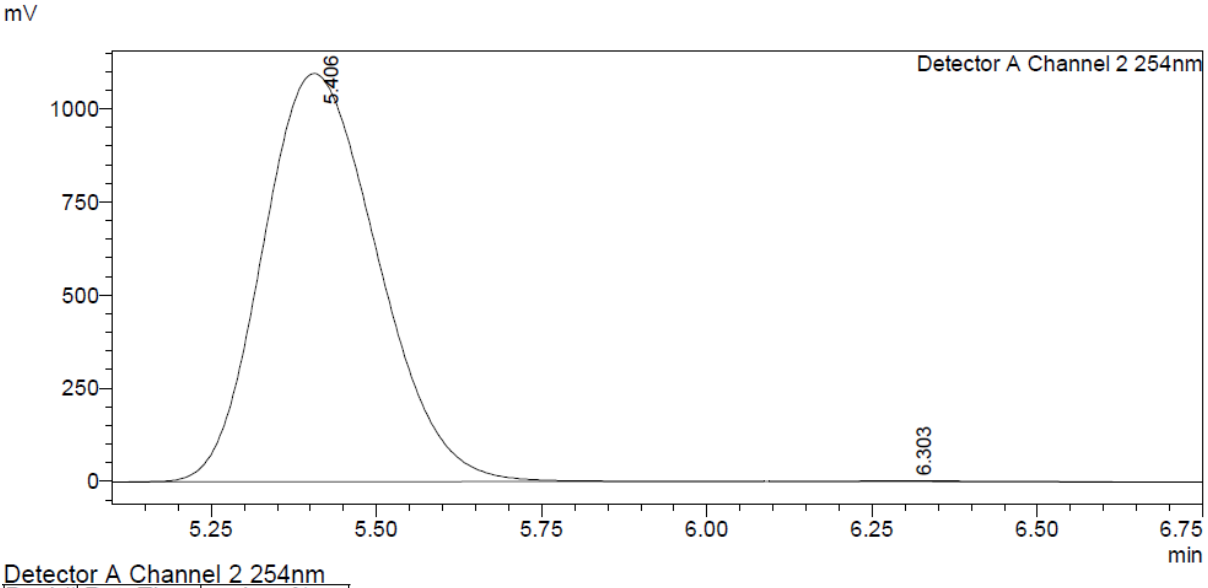

Detector A Channel 2 254nm

Peak#	Ret. Time	Area%
1	5.406	99.777
2	6.303	0.223
Total		100.000

Minor dihydropyridinone product: (*S*)-4-(1-oxo-3-(trifluoromethyl)-2,3-dihydro-1*H*-benzo[4,5]oxazolo[3,2-*a*]pyridine-4-carbonyl)benzonitrile **43**

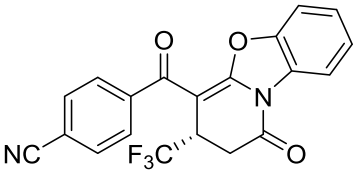

Chiral HPLC analysis: CHIRALPAK IC (20:80 *i*-PrOH:hexane, flow rate 1.5 mL min^{-1}, 211 nm, 40 °C), t_R (*S*): 9.2 min, t_R (*R*): 11.0 min, 97.5:2.5 (*S*:*R*) er

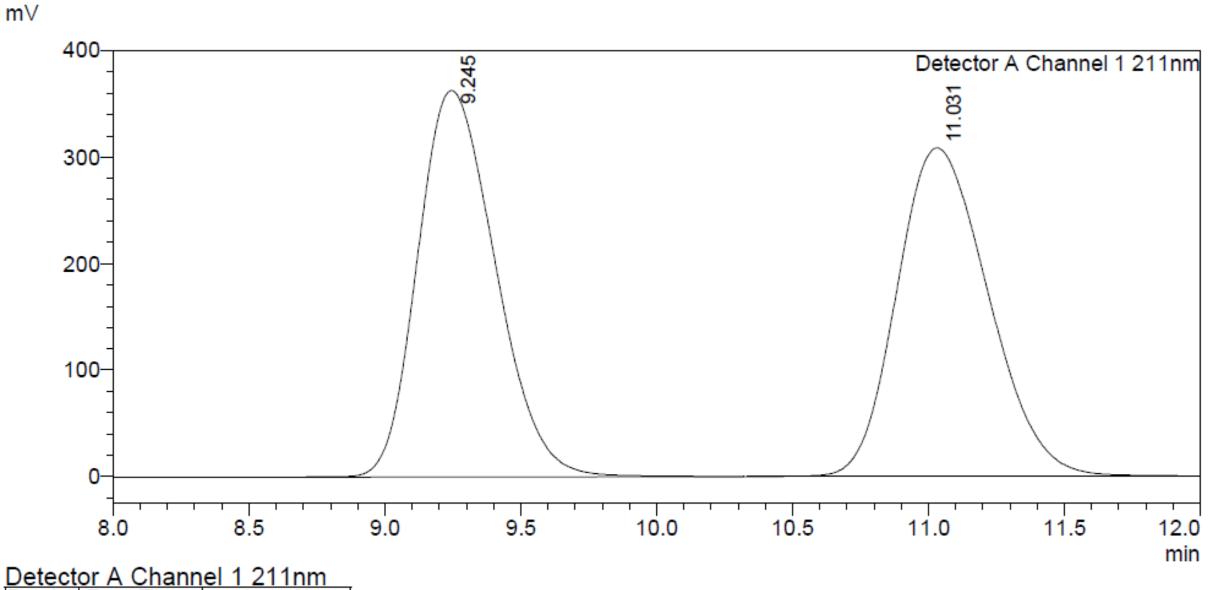

Detector A Channel 1 211nm

Peak#	Ret. Time	Area%
1	9.245	49.934
2	11.031	50.066
Total		100.000

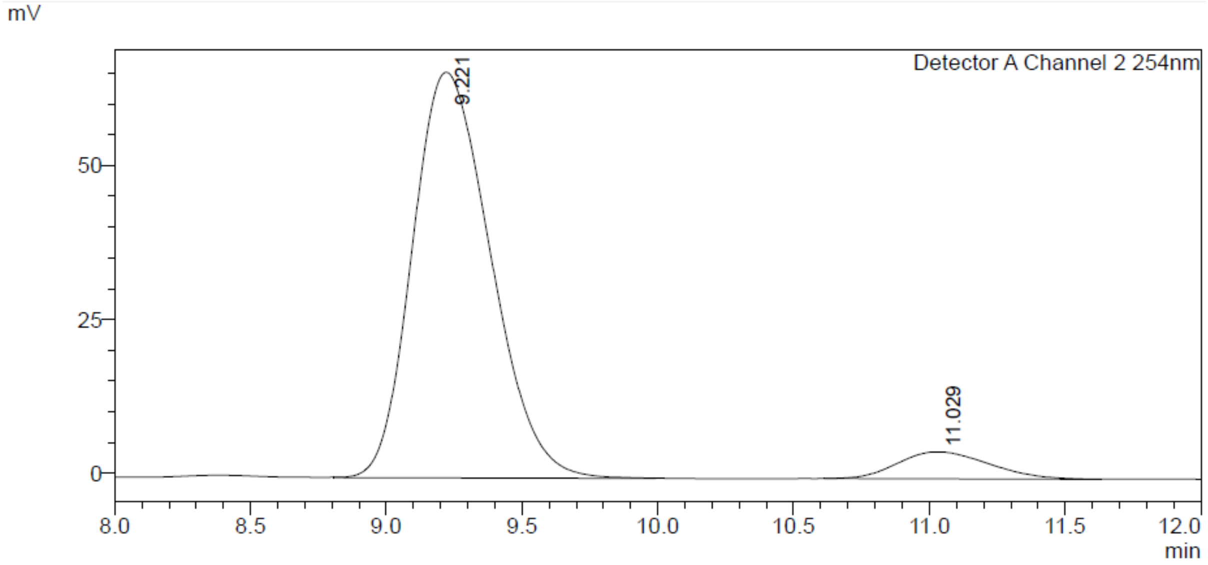

Detector A Channel 2 254nm

Peak#	Ret. Time	Area%
1	9.221	92.812
2	11.029	7.188
Total		100.000

(*S*)-4-Benzoyl-3-(trifluoromethyl)-2,3-dihydro-1*H*-benzo[4,5]oxazolo[3,2-*a*]pyridin-1-one **39**

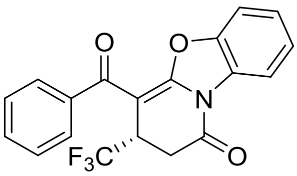

Chiral HPLC analysis: CHIRALPAK AD-H (10:90 *i*-PrOH:hexane, flow rate 1.5 mL min^{-1}, 254 nm, 40 °C), t_R (*S*): 7.3 min, t_R (*R*): 11.2 min, 97.8:2.2 (*S*:*R*) er

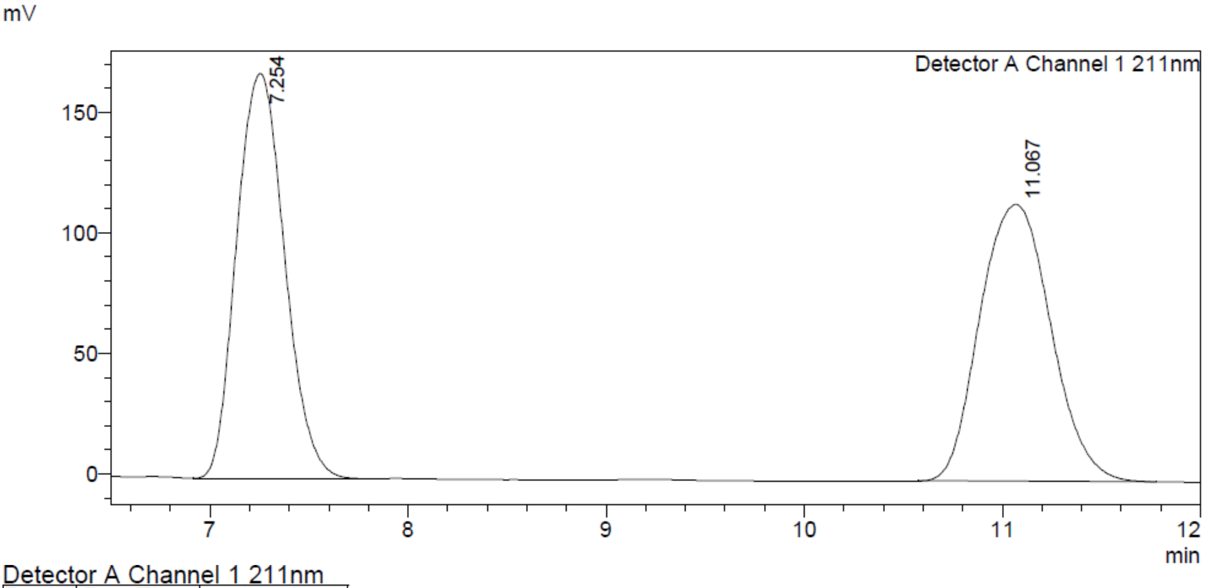

Detector A Channel 1 211nm

Peak#	Ret. Time	Area%
1	7.254	49.794
2	11.067	50.206
Total		100.000

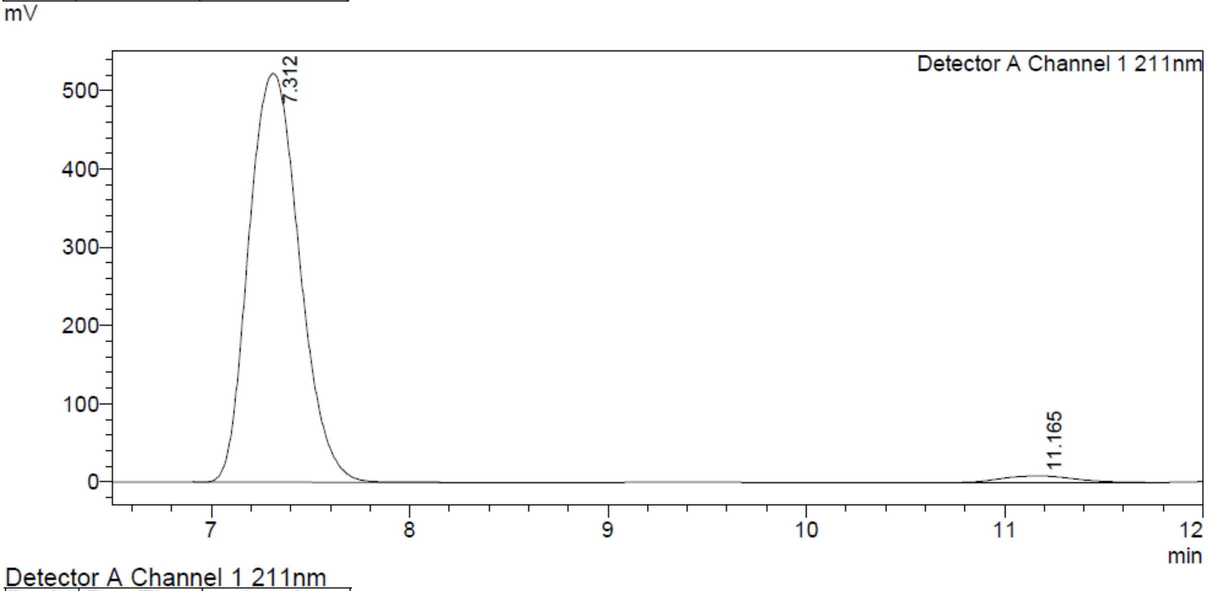

Detector A Channel 1 211nm

Peak#	Ret. Time	Area%
1	7.312	97.804
2	11.165	2.196
Total		100.000

(*S*)-4-Nicotinoyl-3-(trifluoromethyl)-2,3-dihydro-1*H*-benzo[4,5]oxazolo[3,2-*a*]pyridin-1-one **40**

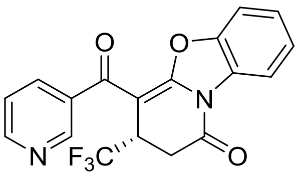

Chiral HPLC analysis: CHIRALPAK AD-H (15:85 *i*-PrOH:hexane, flow rate 1.5 mL min^{-1}, 211 nm, 40 °C), t_R (*S*): 11.5 min, t_R (*R*): 15.7 min, 99.7:0.3 (*S*:*R*) er

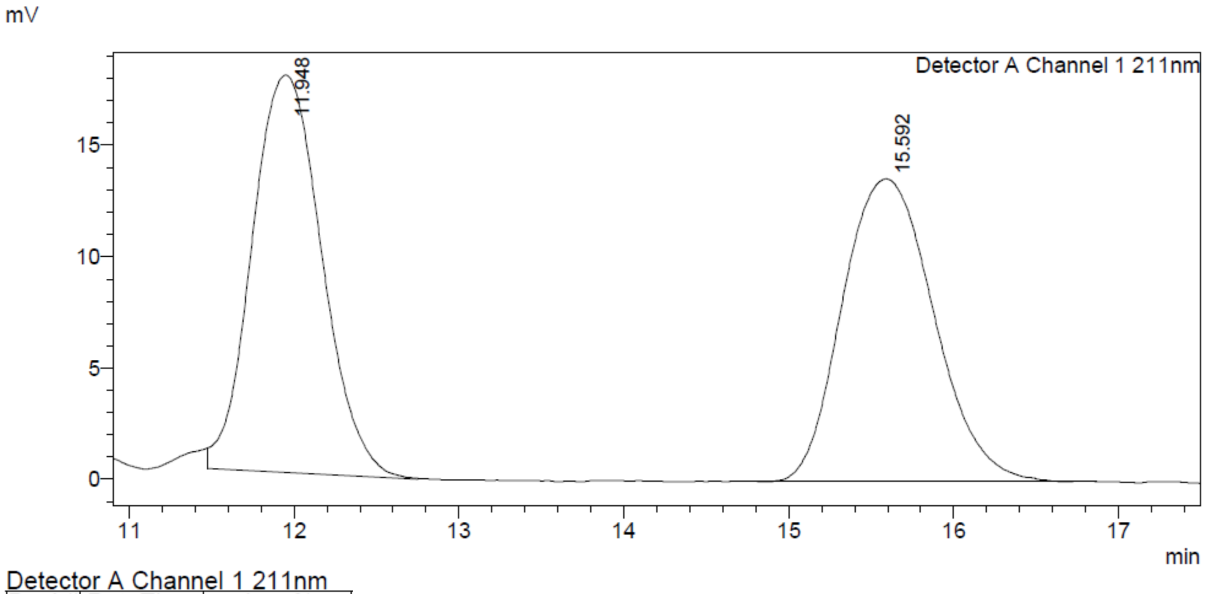

Detector A Channel 1 211nm

Peak#	Ret. Time	Area%
1	11.948	50.070
2	15.592	49.930
Total		100.000

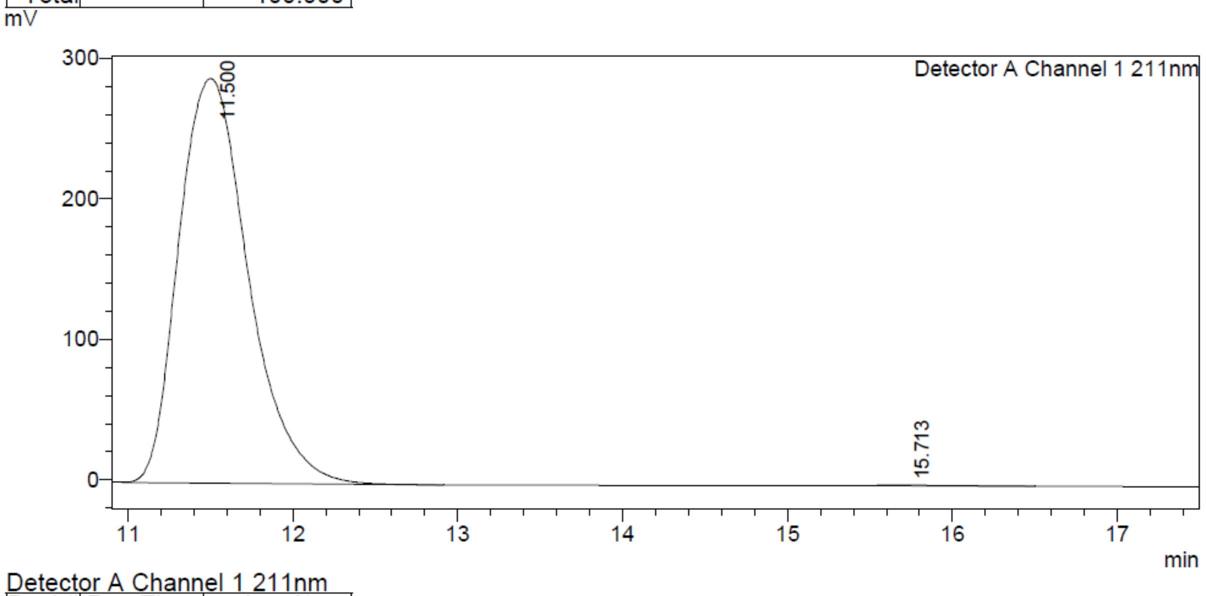

Detector A Channel 1 211nm

Peak#	Ret. Time	Area%
1	11.500	99.719
2	15.713	0.281
Total		100.000

(*S*)-4-(Thiophene-3-carbonyl)-3-(trifluoromethyl)-2,3-dihydro-1*H*-benzo[4,5]oxazolo[3,2-*a*]pyridin-1-one **41**

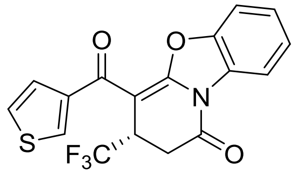

Chiral HPLC analysis: CHIRALPAK AD-H (5:95 *i*-PrOH:hexane, flow rate 1.5 mL min^{-1}, 254 nm, 40 °C), t_R (*S*): 14.6 min, t_R (*R*): 16.5 min, 98.1:1.9 (*S*:*R*) er

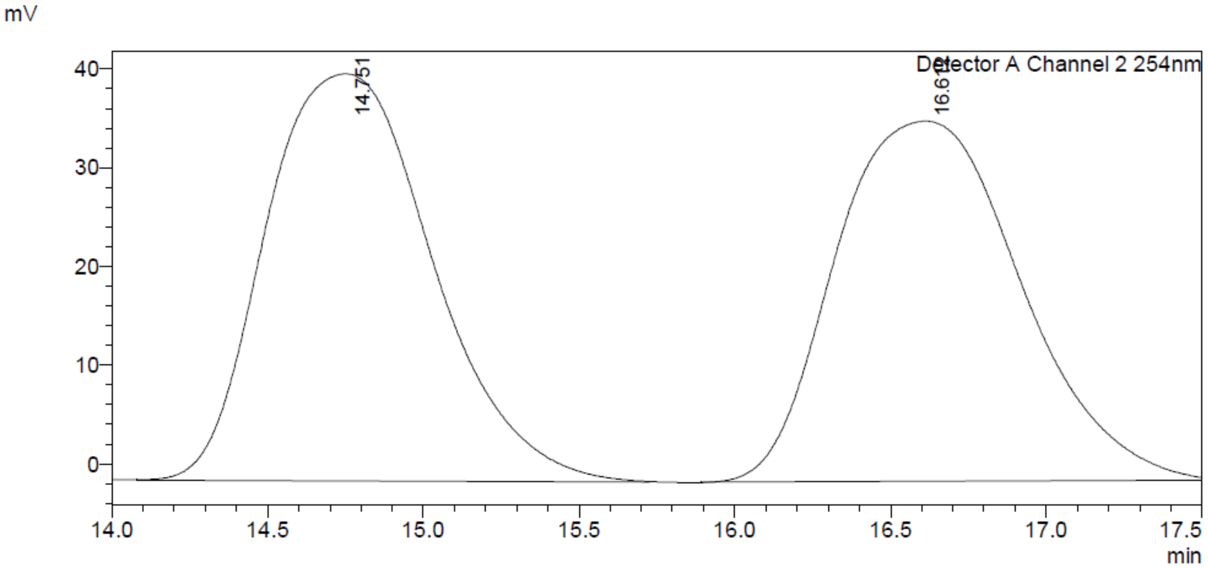

Detector A Channel 2 254nm

Peak#	Ret. Time	Area%
1	14.751	50.439
2	16.612	49.561
Total		100.000

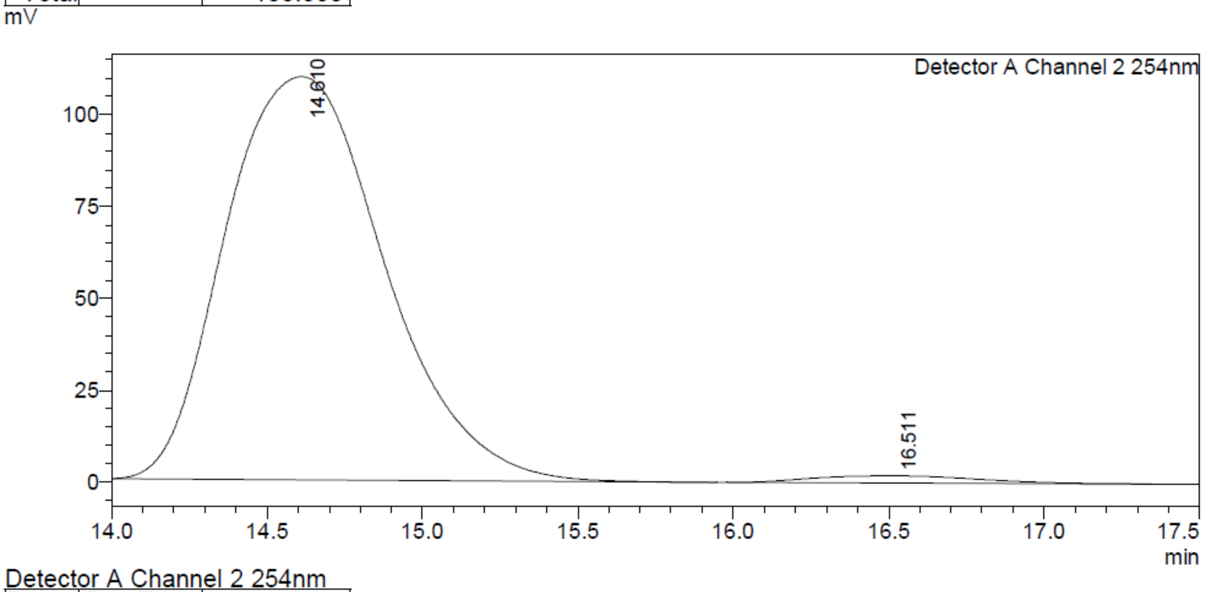

Detector A Channel 2 254nm

Peak#	Ret. Time	Area%
1	14.610	98.144
2	16.511	1.856
Total		100.000

Crystal recovered following absolute configuration determination by X-ray crystallographic analysis:

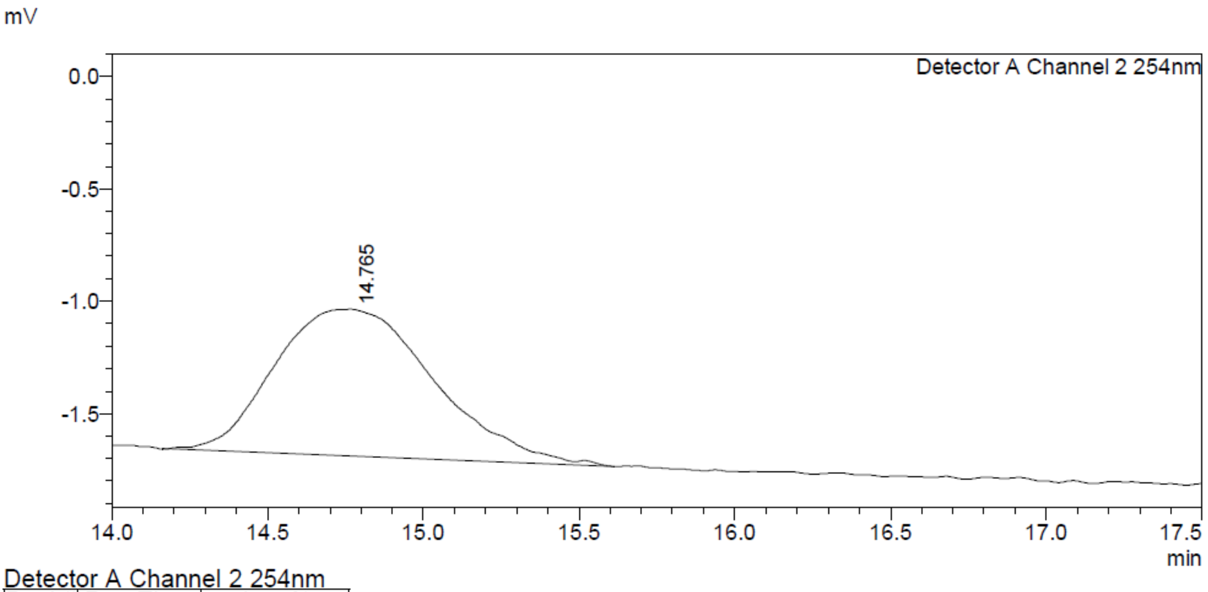

Detector A Channel 2 254nm

Peak#	Ret. Time	Area%
1	14.765	100.000
Total		100.000

(*S*)-3-(Trifluoromethyl)-4-(4-(trifluoromethyl)benzoyl)-2,3-dihydro-1*H*-benzo[4,5]oxazolo[3,2-*a*]pyridin-1-one **42**

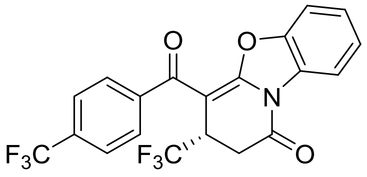

Chiral HPLC analysis: CHIRALPAK AD-H (5:95 *i*-PrOH:hexane, flow rate 1.5 mL min^{-1}, 211 nm, 40 °C), t_R (*S*): 8.2 min, t_R (*R*): 9.6 min, 97.8:2.2 (*S*:*R*) er

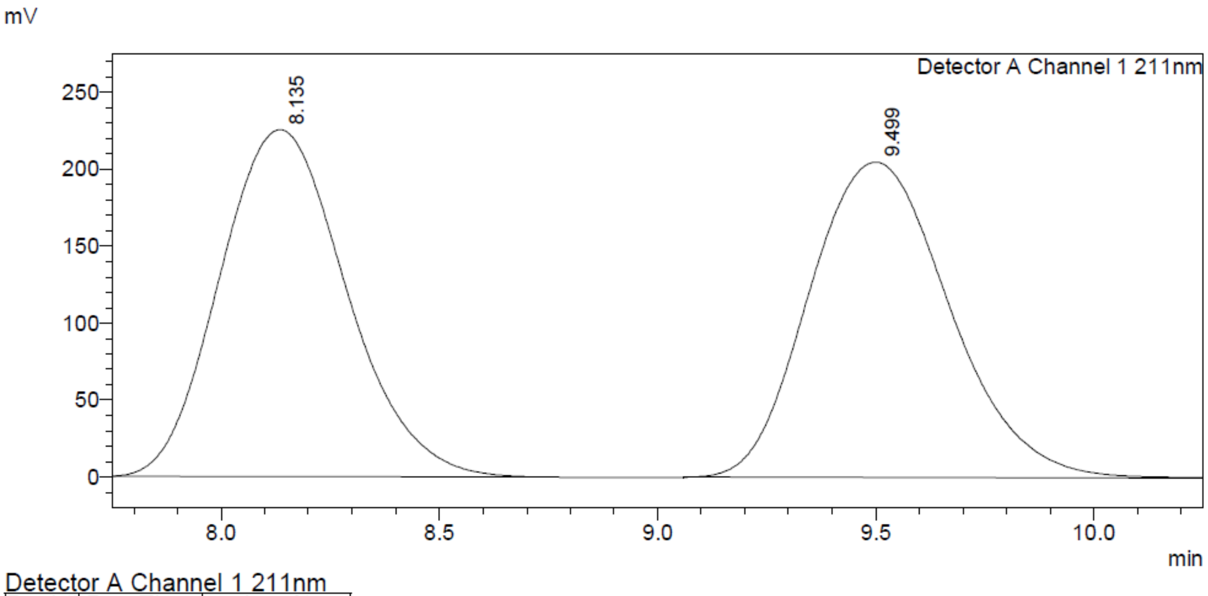

Detector A Channel 1 211nm

Peak#	Ret. Time	Area%
1	8.135	49.860
2	9.499	50.140
Total		100.000

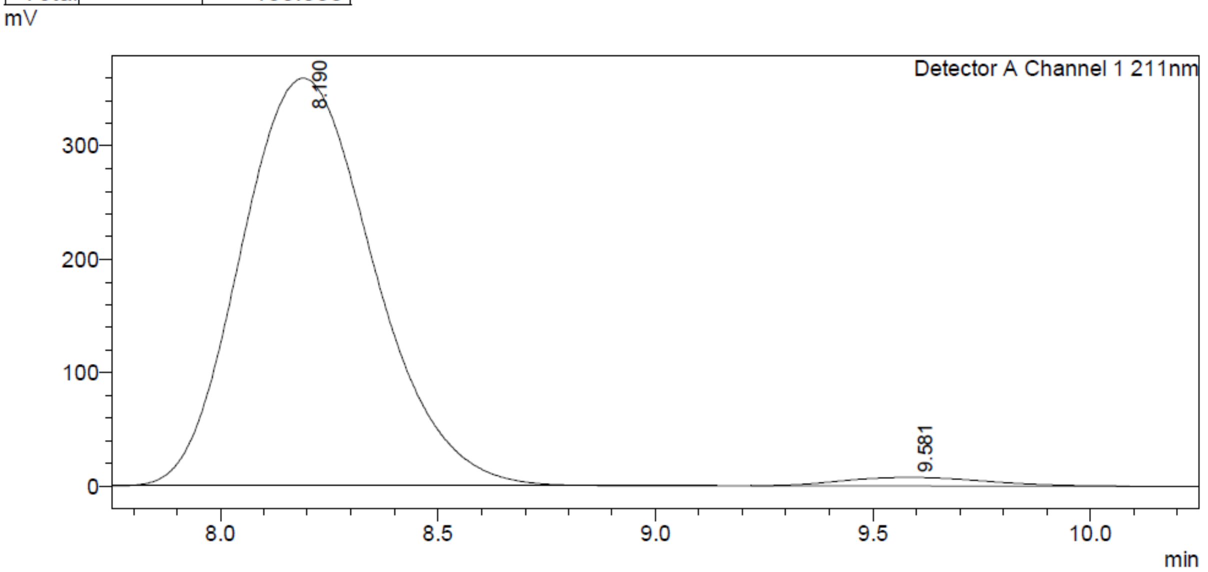

Detector A Channel 1 211nm

Peak#	Ret. Time	Area%
1	8.190	97.791
2	9.581	2.209
Total		100.000

(*S*)-4-(1-Oxo-3-(trifluoromethyl)-2,3-dihydro-1*H*-benzo[4,5]oxazolo[3,2-*a*]pyridine-4-carbonyl)benzonitrile **43**

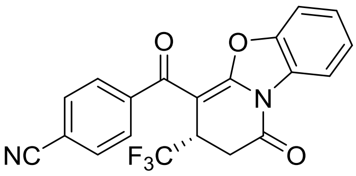

Chiral HPLC analysis: CHIRALPAK IC (20:80 *i*-PrOH:hexane, flow rate 1.5 mL min^{-1}, 211 nm, 40 °C), t_R (*S*): 9.2 min, t_R (*R*): 11.0 min, 97.5:2.5 (*S*:*R*) er

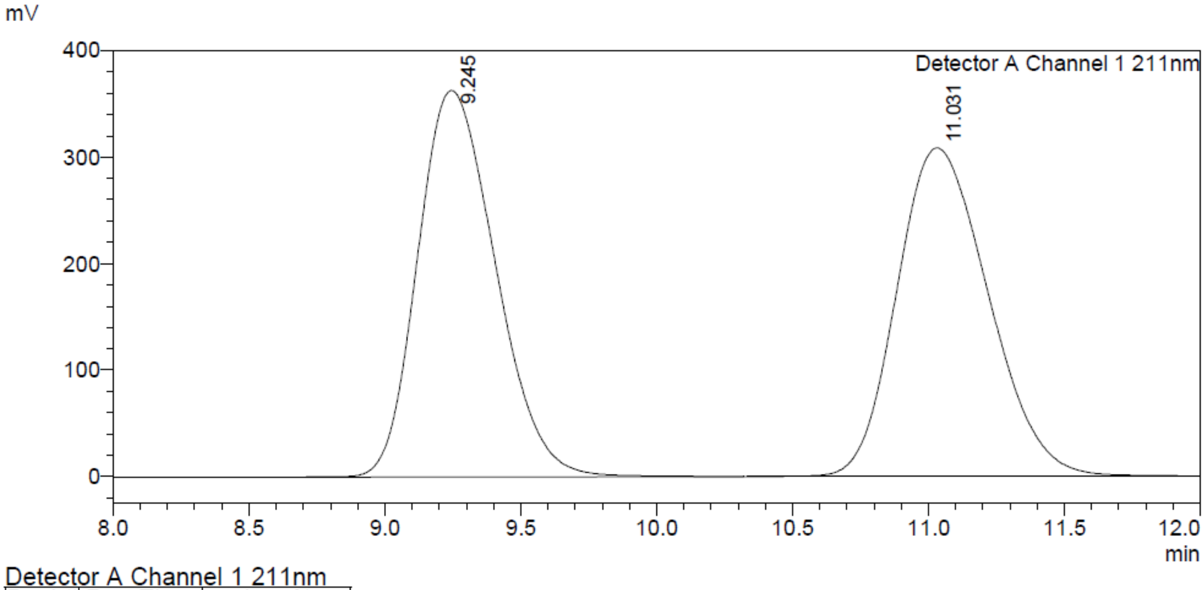

Detector A Channel 1 211nm

Peak#	Ret. Time	Area%
1	9.245	49.934
2	11.031	50.066
Total		100.000

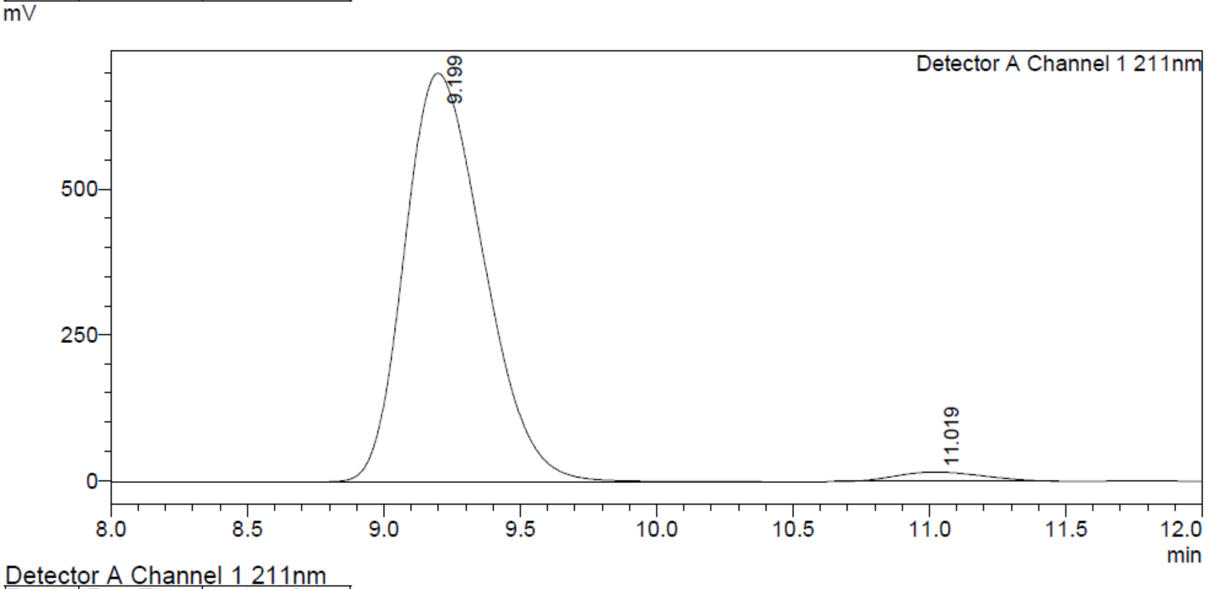

Detector A Channel 1 211nm

Peak#	Ret. Time	Area%
1	9.199	97.526
2	11.019	2.474
Total		100.000

(*R*)-4-Benzoyl-3-phenyl-2,3-dihydro-1*H*-benzo[4,5]thiazolo[3,2-*a*]pyridin-1-one **44**

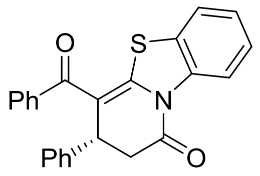

Chiral HPLC analysis: CHIRALPAK AD-H (20:80 *i*-PrOH:hexane, flow rate 1.0 mL min^{-1}, 211 nm, 30 °C), t_R (*S*): 13.2 min, t_R (*R*): 22.4 min, 92.3:7.7 (*R*:*S*) er

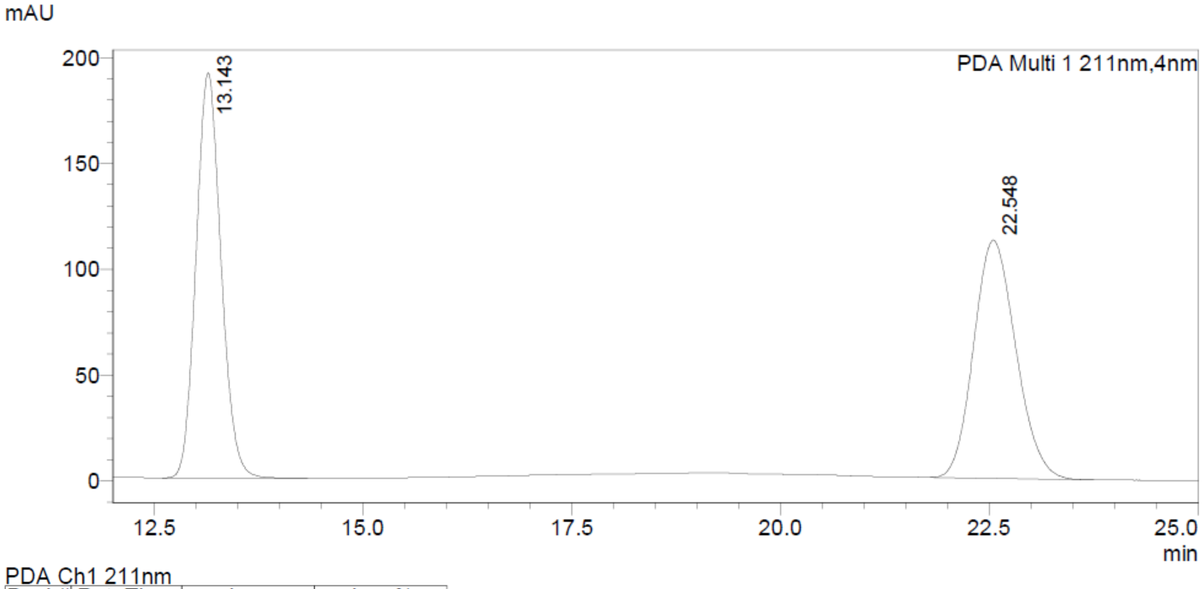

PDA Ch1 211nm

Peak#	Ret. Time	Area	Area%
1	13.143	3989307	50.198
2	22.548	3957761	49.802
Total		7947068	100.000

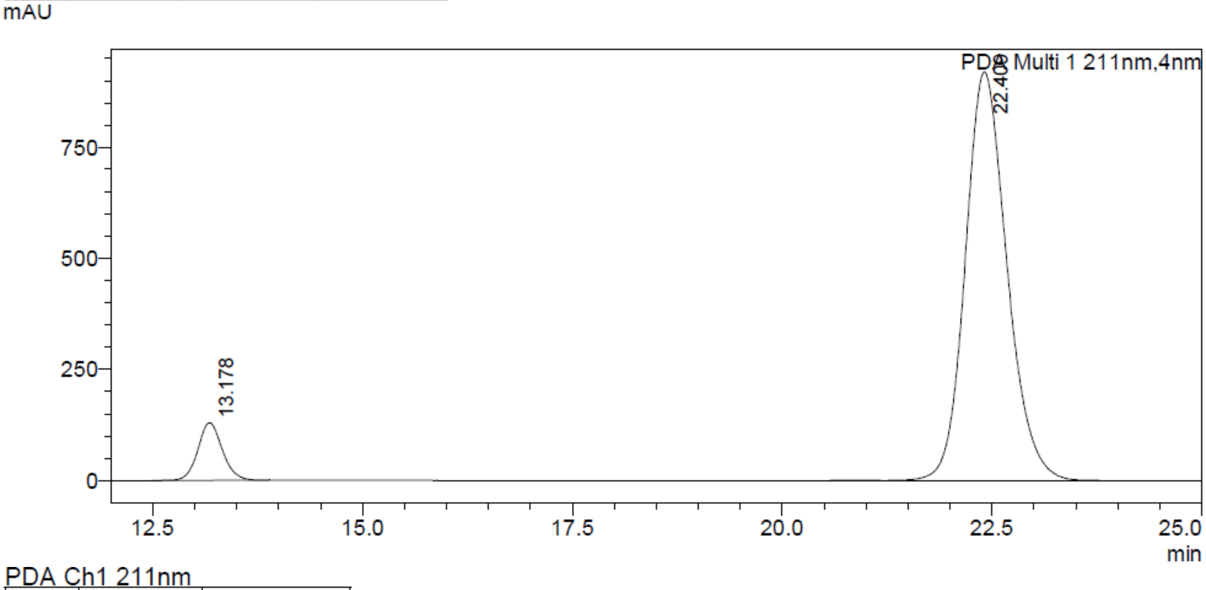

PDA Ch1 211nm

Peak#	Ret. Time	Area%
1	13.178	7.677
2	22.409	92.323
Total		100.000

(*S*)-4-Benzoyl-3-methyl-2,3-dihydro-1*H*-benzo[4,5]thiazolo[3,2-*a*]pyridin-1-one **45**

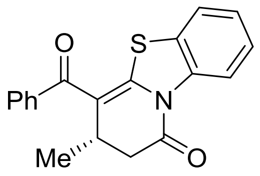

Chiral HPLC analysis: CHIRALPAK AD-H (20:80 *i*-PrOH:hexane, flow rate 1.0 mL min^{-1}, 211 nm, 30 °C), t_R (*R*): 11.4 min, t_R (*S*): 14.4 min, 93.2:6.8 (*S*:*R*) er

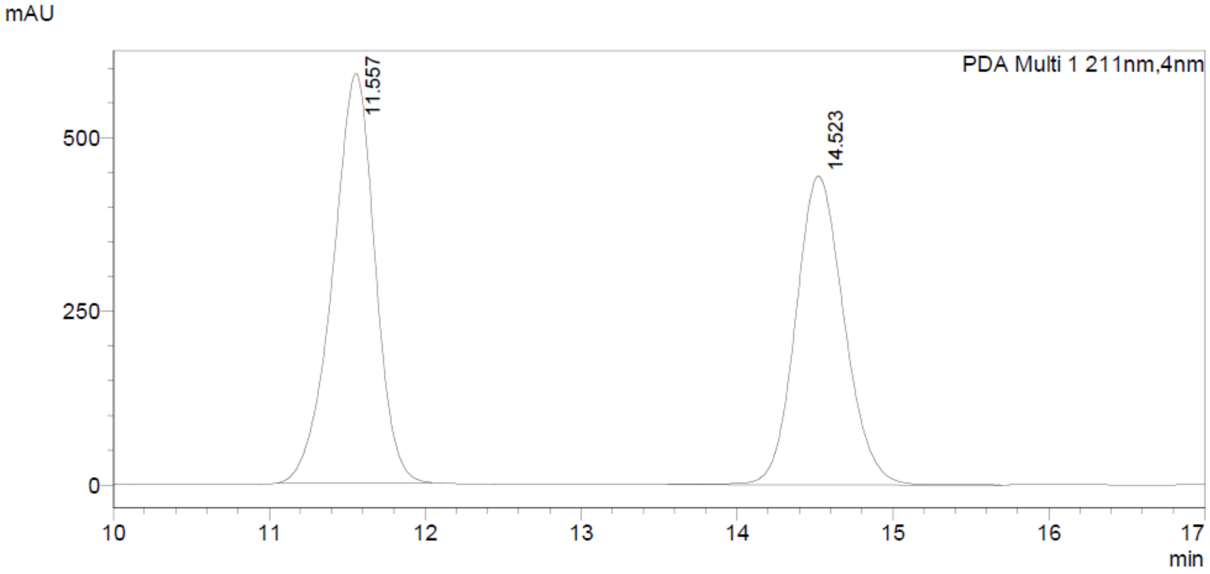

PDA Ch1 211nm

Peak#	Ret. Time	Area	Area%
1	11.557	11073949	53.664
2	14.523	9561574	46.336
Total		20635523	100.000

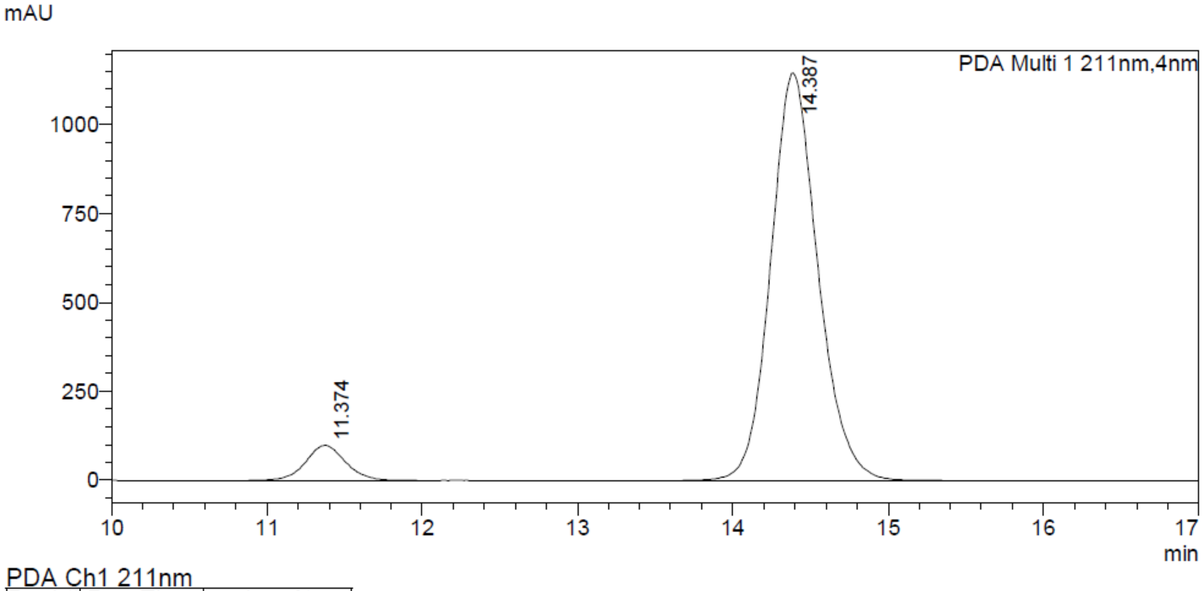

PDA Ch1 211nm

Peak#	Ret. Time	Area%
1	11.374	6.780
2	14.387	93.220
Total		100.000

(*S*)-4-Benzoyl-3-(furan-2-yl)-2,3-dihydro-1*H*-benzo[4,5]thiazolo[3,2-*a*]pyridin-1-one **46**

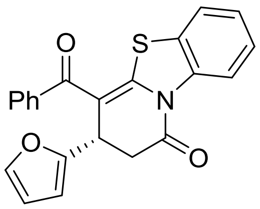

Chiral HPLC analysis: CHIRALPAK AD-H (20:80 *i*-PrOH:hexane, flow rate 1.0 mL min^{-1}, 211 nm, 30 °C), t_R (*R*): 15.0 min, t_R (*S*): 20.2 min, 93.4:6.6 (*S*:*R*) er

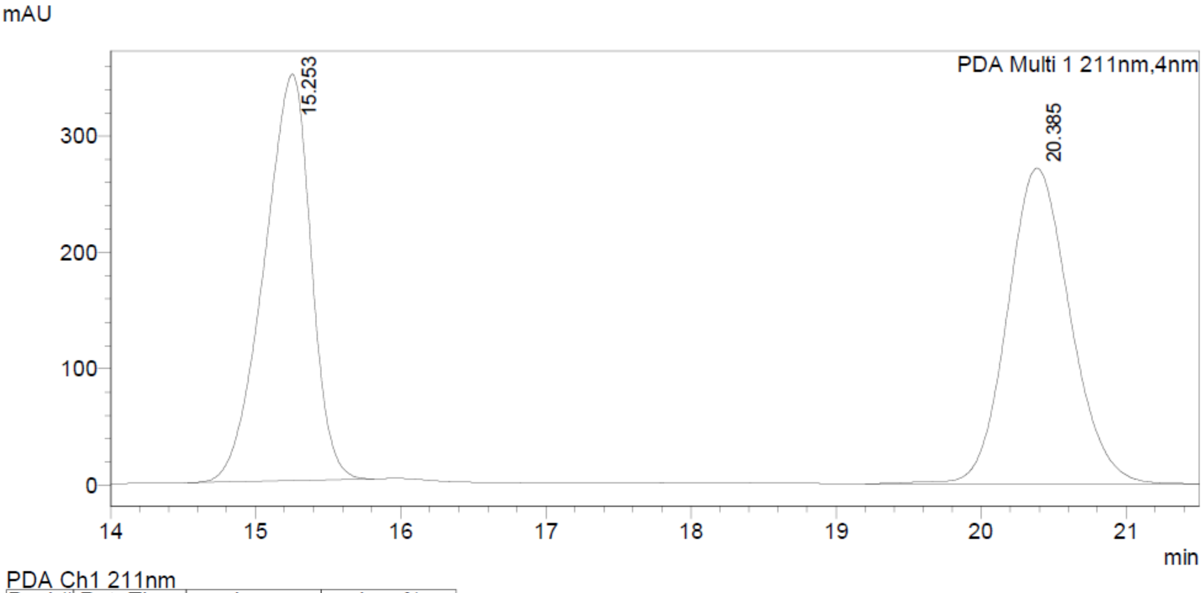

PDA Ch1 211nm

Peak#	Ret. Time	Area	Area%
1	15.253	7874774	49.261
2	20.385	8110945	50.739
Total		15985719	100.000

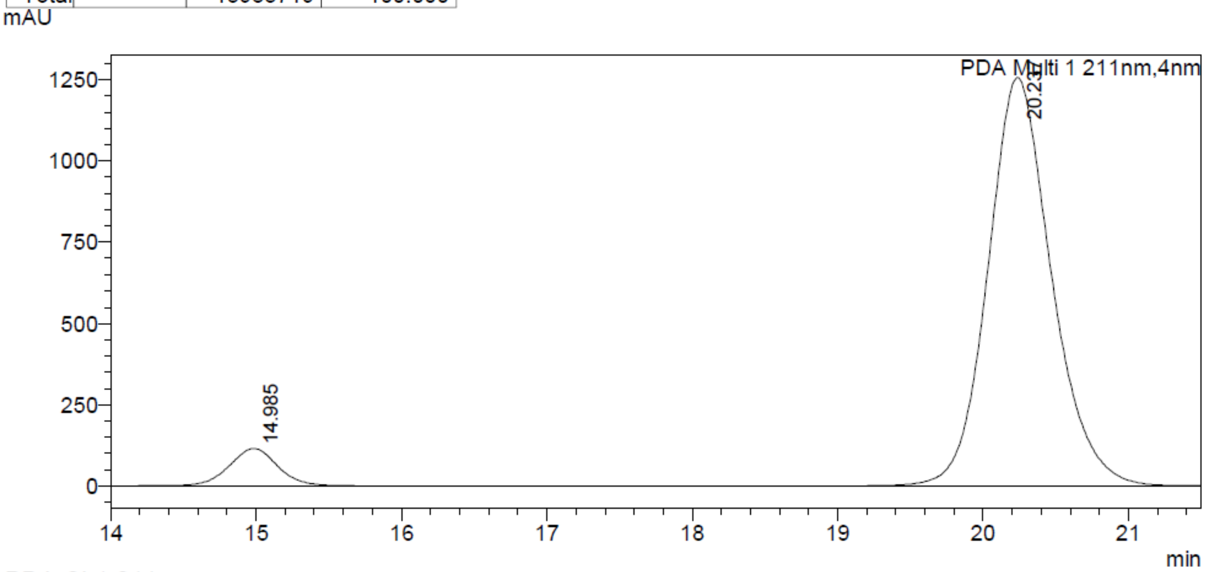

PDA Ch1 211nm

Peak#	Ret. Time	Area%
1	14.985	6.567
2	20.237	93.433
Total		100.000

(R,E)-4-Benzoyl-3-styryl-2,3-dihydro-1H-benzo[4,5]thiazolo[3,2-a]pyridin-1-one **47**

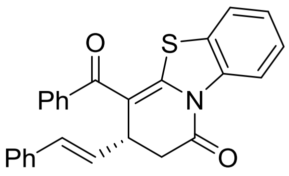

Chiral HPLC analysis: CHIRALPAK AD-H (20:80 *i*-PrOH:hexane, flow rate 1.0 mL min^{-1}, 211 nm, 30 °C), t_R (S): 13.1 min, t_R (S): 18.3 min, 91.2:8.8 (R:S) er

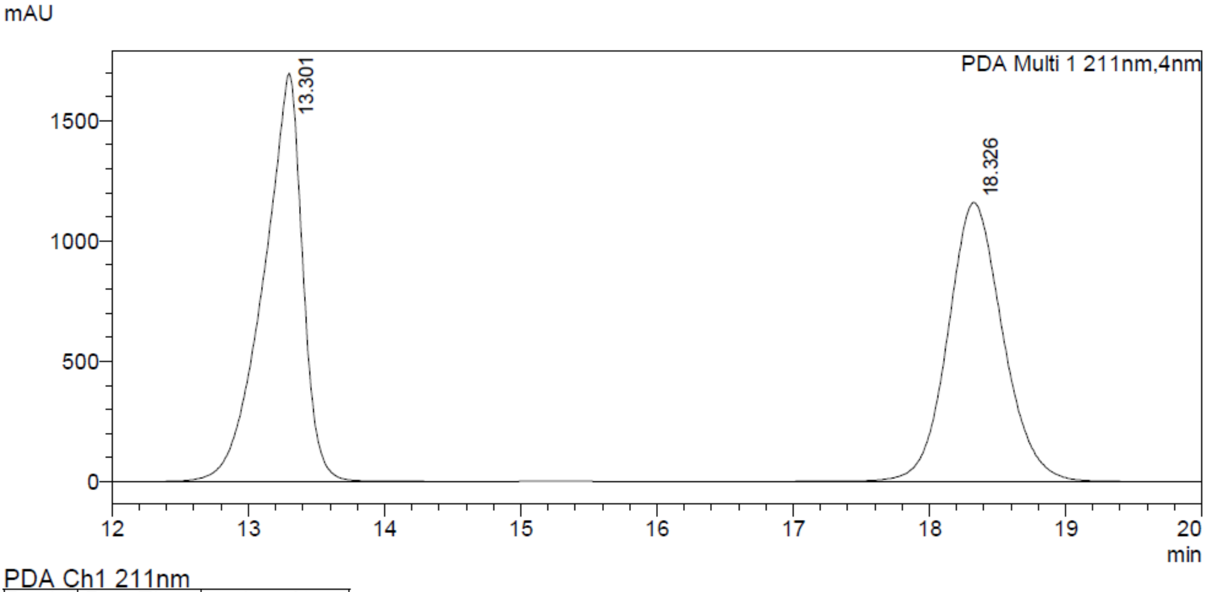

PDA Ch1 211nm

Peak#	Ret. Time	Area%
1	13.301	50.946
2	18.326	49.054
Total		100.000

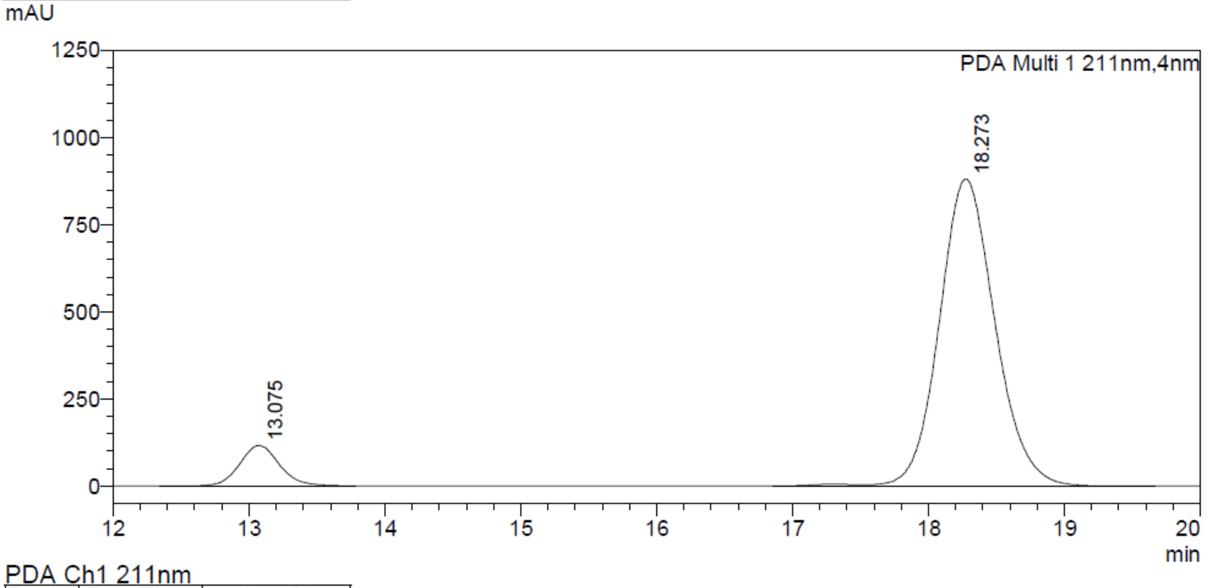

PDA Ch1 211nm

Peak#	Ret. Time	Area%
1	13.075	8.841
2	18.273	91.159
Total		100.000

References

1. H. M. Walborsky and M Schwarz, *J. Am. Chem. Soc.,* 1953, **75**, 3241–3243.
2. H. Plenkiewicz, W. Dmowski and M Lipínski, *J. Fluorine Chem.,* 2001, **111**, 227–232.
3. M. Berger, S. Veit, H. P. Niedermann, T. Kappesser and A. Stutz, Int. Pat., WO 2015/177179 A1, 2015.
4. E. R. T. Robinson, D. M. Walden, C. Fallan, M. D. Greenhalgh, P. H.-Y. Cheong and A. D. Smith, *Chem. Sci.,* 2016, **7**, 6919–6927.
5. E. R. T. Robinson, C. Fallan, C. Simal, A. M. Z. Slawin and A. D. Smith, *Chem. Sci.,* 2013, **4**, 2193–2200.
6. A. Matviitsuk, J. E. Taylor, D. B. Cordes, A. M. Z. Slawin and A. D. Smith, *Chem. Eur. J.,* 2016, **22**, 17748–17757.
7. M. Fogagnolo, P. P. Giovannini, A. Guerrini, A. Medici, P. Pedrini and N Colombi, *Tetrahedron: Asymmetry,* 1998, **9**, 2317–2327.
8. H. I. De Silva, S. Chatterjee, W. P Henry and C. U. Pittman, Jr., *Synthesis,* 2012, **44**, 3453–3464.
9. H. B. Kagan and J. C. Fiaud, in *Topics in Stereochemistry*, ed. E. L. Eliel and S. H. Wilen, John Wiley & Sons, 1988, vol. 18, pp. 249–330.
